# Photoinduced Copper‐Catalyzed Cross‐Coupling of Acylsilanes with Heteroarenes via Bimetallic Relay

**DOI:** 10.1002/advs.202409457

**Published:** 2024-10-14

**Authors:** Long Zheng, Ying‐Chao Li, Yichen Wu, Peng Wang

**Affiliations:** ^1^ State Key Laboratory of Organometallic Chemistry and Shanghai‐Hong Kong Joint Laboratory in Chemical Synthesis Shanghai Institute of Organic Chemistry University of Chinese Academy of Sciences CAS 345 Lingling Road Shanghai 200032 P. R. China; ^2^ School of Chemistry and Materials Science Hangzhou Institute for Advanced Study University of Chinese Academy of Sciences 1 Sub‐lane Xiangshan Hangzhou 310024 P. R. China; ^3^ College of Material Chemistry and Chemical Engineering Key Laboratory of Organosilicon Chemistry and Material Technology of Ministry of Education Hangzhou Normal University Hangzhou 311121 P. R. China

**Keywords:** acylsilane, azine N‐oxide, azole, coupling reaction, Fischer copper carbene

## Abstract

The transition metal‐catalyzed direct coupling reactions involving electron‐rich Fischer carbene species are largely underdeveloped and remain a big challenge. Here, a direct coupling reaction of azoles and azine *N*‐oxides is reported with Fischer copper carbene species bearing an *α*‐siloxy group i, which can be in situ generated from acylsilanes catalytically under photoirradiation and redox‐neutral conditions. This coupling reaction between electron‐rich *α*‐siloxy Fischer Cu‐carbene species with hard carbanion nucleophiles may undergo a bimetallic relay process, which is confirmed by the kinetic analysis and in situ NMR analysis. This reaction features mild conditions and remarkable heterocycle compatibility. Notably, this protocol tolerates a series of azole or azine *N*‐oxide derivatives, including benzoxazole, benzothiazole, benzoimidazole, benzoisoxazole, oxazole, oxadiazole, triazolo[4,3*‐a*]pyridine, purine, caffeine, pyridine *N*‐oxide, quinoline *N*‐oxide, pyrazine *N*‐oxide, pyridazine *N*‐oxide, etc. The synthetic value of this approach is demonstrated by the efficient synthesis of a histamine h4 receptor ligand and a marketed drug carbinoxamine.

## Graphical abstract

The coupling of azoles and azine *N*‐oxides with the Fischer‐type copper carbene species bearing a *α*‐siloxyl group which can be in situ generated from acylsilanes catalytically under photoirradiation and redox‐neutral conditions, is realized. This reaction represents the first example of Cu‐catalyzed coupling reaction of electron‐rich Fischer carbene species with hard nucleophiles. KIE studies, kinetic analysis, and in situ NMR experiments implied a bimetallic relay process.

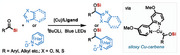

## 1 Introduction

Metal carbenes have emerged as highly versatile intermediates, involving many efficient reactions, such as cyclopropanation, C─H and X─H bond insertion reactions, tandem cyclization, etc.^[^ ^1^, ^2^, ^3^, ^4^ ^]^ In view of the great success in this context, metal carbene species with electron‐deficient (carbonyl, nitro group, etc.) and electron‐neutral (aryl, alkenyl, alkynyl group, etc.) substituents have been well established due to the ready accessibility of corresponding stable carbene precursors including diazo compounds and their analogs (*N*‐tosylhydrazones).^[^ ^1^, ^2^, ^3^, ^4^ ^]^ (For selected reviews on metal carbenes involved cyclopropanation and other cyclization, see ref. [2]) (For selected reviews on metal carbenes involved C–H and X–H insertion reactions, see ref. [3]) (For selected reviews on transition metal catalyzed coupling reactions involved metal carbene species, see ref. [4]) In sharp contrast, the investigations on catalytic reactions involving the metal carbene species bearing electron‐rich heteroatom (O, N, S, etc.) substituents are considerably limited, primarily due to the lack of readily available and stable metal carbene precursors. The corresponding diazo compounds bearing electron‐rich heteroatoms are highly unstable and explosive, and the use of stoichiometric isolable Group VI metal (Cr, Mo, or W) carbene bearing methoxy group,^[^ ^5^, ^6^ ^]^ (For selected reviews on synthetic applications of Fischer carbene complexes, see ref. [5]) normally prepared by using strictly dry anaerobic operation technique, disobeys the principles of atom‐economy and green chemistry. Only a few catalytic approaches for the access of electron‐rich Fischer carbenes with heteroatom functionalities have been mentioned,^[^ ^7^, ^8^, ^9^, ^10^, ^11^ ^]^ (For selected reviews on the formation of siloxy carbene from acylsilanes, see ref. [10]) For the reactions involved siloxy Fischer carbene species, see ref. [11]) including the attack of heteroatomic nucleophiles to tungsten vinylidene intermediate,^[^ ^7^ ^]^ hydroamination of terminal alkynes with rhodium catalyst,^[^ ^8^ ^]^ metal‐carbene bearing a leaving group,^[^ ^9^ ^]^ and in situ trap of transient siloxycarbenes with transition metal catalyst.^[^ ^10^, ^11^ ^]^ Undoubtedly, to further develop novel strategies for the generation of electron‐rich *α*‐heteroatom metal carbenes catalytically as well as their novel synthetic applications is still highly desired and remains a big challenge.

**Scheme 1:**
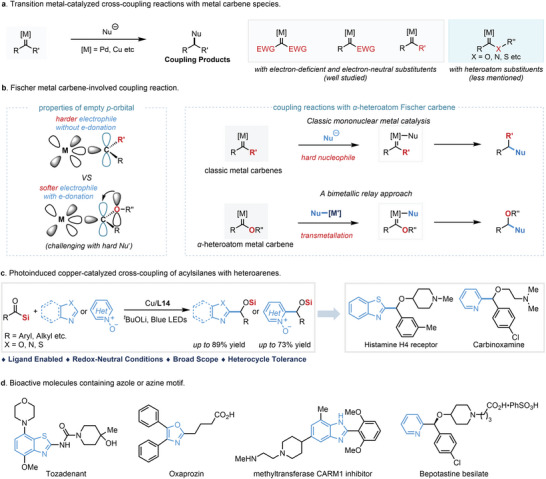
Photoinduced copper‐catalyzed cross‐coupling of acylsilanes with azoles and azine *N*‐oxides.

Transition metal‐catalyzed cross‐coupling reactions relied on metal carbene intermediates have attracted increasing attention for the efficient construction of carbon‐carbon and carbon‐heteroatom bonds (**Scheme** **1**a).^[^ ^4^ ^]^ In this context, the only example regarding the coupling of electron‐rich *α*‐siloxy Fischer copper carbenes with soft *π*‐nucleophile (alkyne) has been demonstrated recently by our group using readily accessible acylsilanes under photoirradiation in the presence of a suitable copper catalyst.^[^ ^11d^ ^]^ In principle, this approach should be compatible with a series of nucleophiles, thus rendering this approach potentially broadly applicable. However, the transformation achieved currently with soft nucleophile raises a question if hard nucleophiles could be employed in the transition metal‐catalyzed coupling reaction with *α*‐siloxy Fischer carbene species. In comparison to the well‐studied metal carbene species containing electron‐deficient or electron‐neutral groups, the *α*‐siloxy Fischer carbene species from acylsilane which contains the electronically donating *α*‐siloxy group adjacent to electron‐neutral group led to a more “softer” electrophile of the empty *p*‐orbital (Scheme 1b). As a result, the coupling of *α*‐siloxy Fischer carbene with a hard nucleophile, such as a carbanion, is more challenging due to the hard‐soft acid‐base (HSAB) principle. To overcome this challenge, we envisioned that the coupling reaction of electron‐rich *α*‐siloxy Fischer carbene with a hard nucleophile might be realized via a bimetallic relay process, in which the key intermediate could be formed via transmetalation with carbon‐metal species (Scheme 1b). Here, we demonstrate the Cu‐catalyzed coupling reaction of electron‐rich siloxy carbene species with a hard carbon nucleophile generated from azoles or azine *N*‐oxides in the presence of a strong base^[^ ^12^, ^13^ ^]^ (Scheme 1c). The kinetic analysis and in situ NMR analysis indicate a bimetallic relay process, which is the key to the success of this coupling reaction between electron‐rich *α*‐siloxy Fischer Cu‐carbene species with heteroarenes in the presence of a strong base. This method offers a general method for the preparation of various azole‐containing or azine‐containing alcohols in high efficiency under mild conditions. With the assistance of an electron‐rich bipyridine ligand, this protocol could tolerate a wide range of azoles or azine *N*‐oxides, including benzoxazole, benzothiazole, benzoimidazole, benzoisoxazole, oxazole, oxadiazole, triazolo[4,3*‐a*]pyridine, purine, caffeine, pyridine *N*‐oxide, quinoline *N*‐oxide, pyrazine *N*‐oxide, pyridazine *N*‐oxide, etc., which is unusual in Cu‐catalyzed carbene‐involved coupling reaction.^[^ ^13^ ^]^ Given the azole‐containing or azine‐containing motifs are privileged in a large number of pharmaceutically active molecules (Scheme 1d)^[^ ^14^ ^]^ this reaction might find wide applications in the pharmaceutical industry and drug discovery. The synthetic value of this approach has been strengthened by the efficient preparation of a histamine h4 receptor ligand and a marketed drug carbinoxamine. Moreover, the bimetallic relay approach might also open a new avenue for the development of novel coupling reactions of electron‐rich heteroatom Fischer carbenes with other hard nucleophiles.

## 2 Results and Discussion

Based on our experiences on the Cu‐catalyzed coupling reaction of acylsilanes with alkynes, we commenced our studies by choosing benzoylsilane (**1**) and benzoxazole (**2a**) as the model

Substrates in the presence of a catalytic amount of Cu(OTf)_2_ and a strong base *^t^* BuOLi under the irradiation of blue light. To our delight, the desired coupling product (**3a**) was observed in 35% NMR yield with the assistance of a bisoxazoline (Box) ligand **L1**. The optimization of bases indicates this novel coupling reaction could only happen in the presence of strong bases, such as *^t^* BuOLi, *^t^* BuONa, and *^t^* BuOK (See Supporting Information for more information), which could deliver the azole anion or azole copper species via deprotonation. Next, systematic ligand effects on this reaction have been evaluated using CuCl_2_ as the catalyst precursor (**Table** **1**), as CuCl_2_ gave a slight better yield than Cu(OTf)_2_. Unlike our previous reaction with soft nucleophile, the side‐arm modified mono‐benzyl *^i^* Pr‐Box **L2** could not enhance the efficiency of this coupling reaction, giving the coupling product (**3a**) in 33% NMR yield. Moreover, the coupling product **3a** is racemic, despite the use of chiral bisoxazoline ligands **L1** and **L2**. Although the use of bis‐benzothiazole ligand (**L3**), bioxazoline ligand (**L4**), and diimine ligand (**L5**) cannot further increase the reactivity of this reaction, bipyridine **L8** accelerated this reaction in some extent (46% NMR yield). On this basis, a series of modified bipyridine (**L8‐L15**) and phenanthroline (**L16**) ligands were investigated. We found that the electron‐rich bipyridine ligand (**L14**) bearing a *para*‐methoxy group gave the best outcome (50% NMR yield). The yield could be further optimized to 85% NMR yield, when the loading of benzoxazole (**2a**) increased to 2.0 equivalent. Control experiments unveiled that the use of electron‐rich bipyridine ligand is crucial, and an inferior result was obtained in the absence of **L14**.

**Table 1:** Ligand evaluation for copper‐catalyzed coupling of acylsilanes with azoles.(see Table notes).

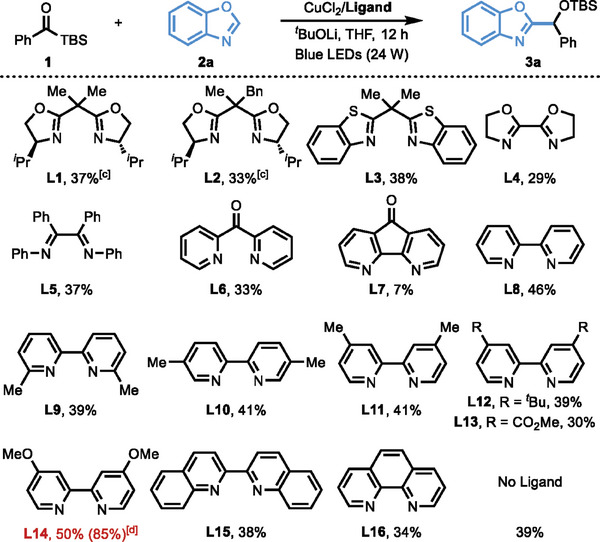

^a)^ Reaction conditions: **1** (0.1 mmol, 1.0 equiv.), **2a** (0.1 mmol, 1.0 equiv.), CuCl_2_ (10 mol%), Ligand (12 mol%), *^t^* BuOLi (0.1 mmol, 1.0 equiv.), THF (1.0 mL), Blue LEDs (450‐470 nm, 24 W), 12 h;

^b)^ Yield was determined by ^1^H NMR using CH_2_Br_2_ as the internal standard;

^c)^ product **3a** is racemic;

^d)^ **2a** (0.2 mmol, 2.0 equiv.) was used.

With the optimal reaction conditions in hand, the generality of this coupling reaction with various azole derivatives was evaluated by employing benzoylsilane (**1**) as the model substrate (**Table** **2**). A wide range of 1,3‐benzoxazoles (**2a**, **2c‐j**) bearing various functionalities are well tolerated, delivering the corresponding products in moderate to excellent yields. In general, the substituents with both electron‐rich and electron‐deficient functional groups at various positions (4‐, 5‐, 6‐, 7‐position) on the aromatic rings are all suitable for this reaction, and electron‐rich benzoxazoles normally provided higher yields than electron‐deficient ones. A series of oxazoles were also evaluated (**2k‐y**). Electron‐donating and electron‐withdrawing substituents on the aryl group of oxazole coupling partners, including methyl (**2w**), methoxy (**2m**, **2q**), fluoro (**2n**), chloro (**2o**, **2x**), bromo (**2p**, **2r**, **2t**) and trifluoromethyl (**2s**), are tolerated, furnishing coupling products in 36–72% yields. We also found that the diphenyl (**2y**), naphthyl (**2u**) and thienyl (**2v**) substituted oxazoles are suitable substrates. Notably, the reaction showed high level of compatibilities with other heterocycles, such as benzothiazole (**2b**), benzimidazole (**2z**), 1,2‐benzoxazole (**2aa**), oxadiazole (**2ab**), triazolo[4,3‐*a*]pyridine (**2ac**), purine (**2ad**) and caffeine (**2ae**), which further demonstrates the versatility of this reaction.

**Table 2:** Scope of azoles and azine *N‐*oxides.(see Table notes).

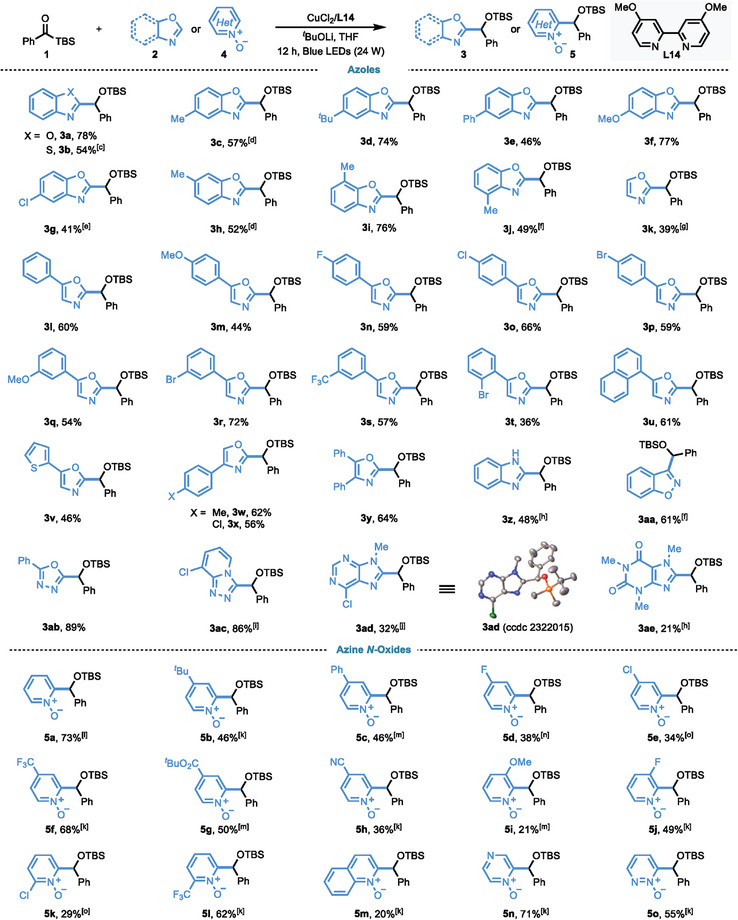

^a)^ Reaction conditions: **1** (0.1 mmol, 1.0 equiv.), **2** or **4** (0.2 mmol, 2.0 equiv.), CuCl_2_ (10 mol %), **L14** (12 mol%), LiO*^t^* Bu (0.1 mmol, 1.0 equiv.), THF (1.0 mL), Blue LEDs (24 W), 12 h;

^b)^ Isolated yield;

^c)^ **1** (0.12 mmol, 1.2 equiv.), **2** (0.1 mmol, 1.0 equiv.), LiO*^t^* Bu (0.2 mmol, 2.0 equiv.):

^d)^ LiO*^t^* Bu (0.08 mmol, 0.8 equiv.);

^e)^ **1** (0.12 mmol, 1.2 equiv.), **2** (0.1 mmol, 1.0 equiv.), CuBr (10 mol%), LiO*^t^* Bu (0.08 mmol, 0.8 equiv.);

^f)^ NaO*^t^* Bu (0.1 mmol, 1.0 equiv.);

^g)^ LiO*^t^* Bu (0.2 mmol, 2.0 equiv.), THF (0.5 mL);

^h)^ NaO*^t^* Bu (0.2 mmol, 2.0 equiv.);

^i)^ LiO*^t^* Bu (0.2 mmol, 2.0 equiv.);

^j)^ CuCl_2_ (20 mol%), **L14** (24 mol%), NaO*^t^* Bu (0.2 mmol, 2.0 equiv.);

^k)^ LiO*^t^* Bu (0.2 mmol, 2.0 equiv.), 24 h;

^l)^ CuBr_2_ (10 mol%), LiO*^t^* Bu (0.2 mmol, 2.0 equiv.), 24 h;

^m)^ LiO*^t^* Bu (0.3 mmol, 3.0 equiv.), 24 h;

^n)^ CuCl_2_ (20 mol%), **L14** (24 mol%), LiO*^t^* Bu (0.2 mmol, 2.0 equiv.), 24 h;

^o)^ **4** (0.3 mmol, 3.0 equiv.), LiO*^t^* Bu (0.2 mmol, 2.0 equiv.), 24 h.

Encouraged by the success of azoles, we next investigated the compatibility of other heteroarenes, however, the use of pyridine resulted in the decomposition of acylsilanes without the detection of any desired coupling product. Given the difficulties of deprotonation of simple pyridine derivatives, we next checked corresponding pyridine *N*‐oxide bearing a more acidic *α*‐hydrogen. To our great delight, the desired coupling product (**5a**) could be obtained in a 73% yield employing benzoylsilane (**1**) and pyridine *N*‐oxide as the model substrates. This observation further emphasized the generality of our protocol in the preparation of heterocycle‐containing secondary alcohols, which are not easy to access via nucleophilic addition with aldehydes. Next, the scope with respect to azine *N*‐oxides was evaluated. As presented in Table 2, this coupling reaction could tolerate both electron‐withdrawing groups and electron‐donating groups at various positions (2‐, 3‐, 4‐position) on the pyridine ring, providing corresponding products in moderate to good yields (**5a‐l**). Moreover, a wide range of functional groups were well tolerated, such as methoxy (**4i**), *tert*‐butyl (**4b**), phenyl (**4c**), fluoro (**4d**, **4j**), chloro (**4e**, **4k**), trifluoromethyl (**4f**, **4l**), *tert*‐butoxycarbonyl (**4** **g**) and cyanide (**4** **h**), providing corresponding coupling products in moderate to good yields. Notably, other azine *N*‐oxides, including quinoline *N*‐oxide (**4m**), pyrazine *N* ‐oxide (**4n**), and pyridazine *N* ‐oxide (**4o**), are all reactive, giving corresponding products in synthetic useful yields.

Next, we turned to investigate the substrate scope of acylsilanes (**Table** **3**). Employing 1,3‐benzoxazole (**2a**) as the model substrate, a wide range of acylsilanes are well tolerated, providing the corresponding products in moderate to excellent yields. This protocol is compatible with a variety of aryl acylsilanes bearing both electron‐rich and electron‐deficient functional groups. As

**Table 3:** Scope of acylsilanes.(see Table notes).

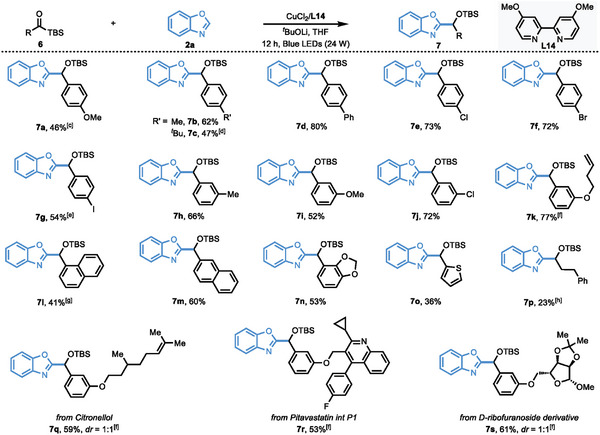

^a)^ Reaction Conditions: **6** (0.1 mmol, 1.0 equiv), **2a** (0.2 mmol, 2.0 equiv), CuCl_2_ (10 mol%), **L14** (12 mol%), LiO*^t^* Bu (0.1 mmol, 1.0 equiv), THF (1.0 mL), Blue LEDs (24 W), 12 h;

^b)^ Isolated yield;

^c)^ CuCl_2_ (20 mol%), **L14** (24 mol%), NaO*^t^* Bu (0.1 mmol, 1.0 equiv), 24 h;

^d)^ CuCl_2_ (20 mol%), **L14** (24 mol%), 24 h;

^e)^ LiO*^t^* Bu (0.06 mmol, 0.6 equiv);

^f)^ CuCl_2_ (20 mol%), **L14** (24 mol%);

^g)^ LiO*^t^* Bu (0.08 mmol, 0.8 equiv);

^h)^ NaO*^t^* Bu (0.1 mmol, 1.0 equiv), 24 h.

Listed in Table 3, functional groups such as methoxy (**6a**, **6i**), methyl (**6b**, **6h**), *tert*‐butyl (**6c**), phenyl (**6d**), chloro (**6e**, **6j**), bromo (**6f**) and iodo (**6g**) etc., are all compatible, providing corresponding coupling products in 46–80% yields. In addition, polycyclic aromatic substituted acylsilanes including 1‐naphthyl (**6l**) and 2‐naphthyl (**6m**) are also suitable substrates, delivering the corresponding products in moderate yields. Notably, this coupling reaction underwent high chemoselectivity when acylsilane substrate **6k** containing a terminal alkene was employed. The cyclopropanation of terminal alkene was not observed with electron‐rich siloxy copper carbene species under our conditions. The piperonyl substituted acylsilane **6n** coupled with benzoxazole in 53% yield. The acylsilane bearing thienyl group (**6o**) is also reactive under our conditions. To our delight, this protocol could also be compatible with aliphatic acylsilane (**6p**), albeit with a slightly lower yield (23% yield) in comparison to aryl acylsilanes. It is noteworthy that our protocol could be used in the late‐stage installation of azoles into bioactive molecules. Substrates derived from citronellol (**6q**), pitavastatin int P1 (**6r**), and D‐ribofuranoside derivative (**6s**) are all reactive, providing corresponding products in 53–61% yields.

**Scheme 2:**
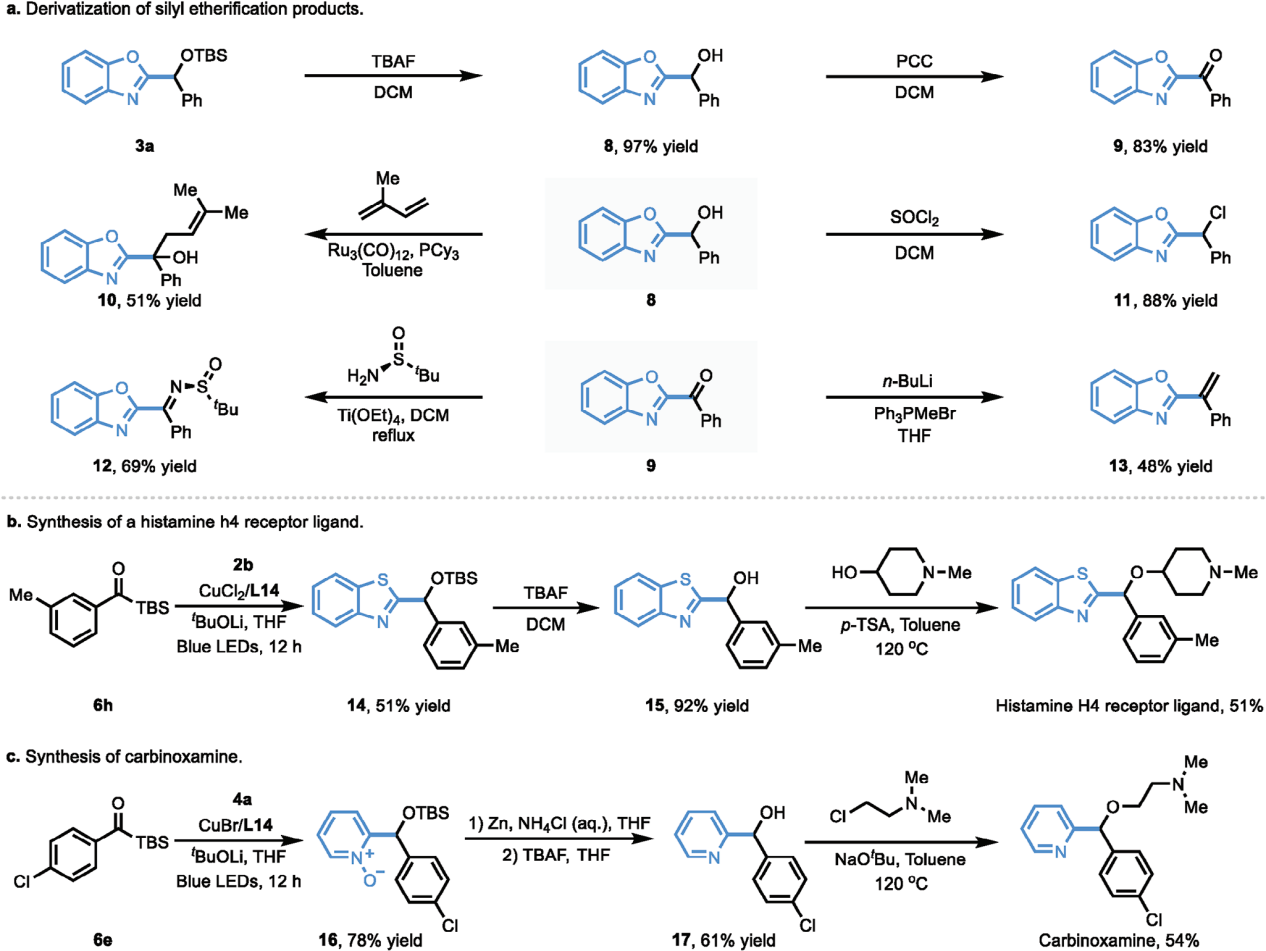
Synthetic applications.

The azole‐containing products are synthetically useful, which could be further derivatized for the preparation of valuable bioactive compounds (**Scheme** **2**a). Using our Cu‐catalyzed coupling product **3a** as substrate, the benzo[*d*]oxazol‐2‐yl(phenyl)methanol (**8**) was obtained in 97% yield with TBAF as the desilylative reagent, which could be further oxidized to corresponding ketone **9** in 83% yield by employing PCC as the oxidant. Treatment of **8** with isoprene using Ru_3_(CO)_12_ as catalyst under redox neutral conditions, 1‐(benzo[*d*]oxazol‐2‐yl)‐4‐methyl‐1‐phenylpent‐3‐en‐1‐ol (**10**) could be obtained in 51% yield. The corresponding benzyl chloride **11** could also be readily accessed in 88% yield by the reaction of **8** with SOCl_2_. In addition, the condensation reaction between benzo[*d*]oxazol‐2‐yl(phenyl)methanone (**9**) and (*S*)‐*tert*‐butanesulfinamide gave (*S*, *E*)‐*N*‐(benzo[*d*]oxazol‐2‐yl(phenyl)methylene)‐2‐methylpropane‐2‐sulfinamide (**12**) in 69% yield in the presence of Ti(OEt)_4_. This chiral sulfinamide is a potential ligand in transition metal‐catalyzed asymmetric reactions. The 2‐(1‐phenylvinyl)benzo[*d*]oxazole (**13**) could be synthesized through Wittig reaction in 48% yield. To further demonstrate the synthetic utility of this newly developed methodology, the efficient synthesis of a histamine h4 receptor ligand and carbinoxamine was carried out. The histamine h4 receptor ligand, an inflammatory mediator,^[^ ^15^ ^]^ was synthesized using our current method as the key step (Scheme 2b). The coupling of *m*‐tolyl acylsilane **6h** with benzothiazole gave the 2‐(((*tert*‐butyldimethylsilyl)oxy)(*m*‐tolyl)methyl)benzo[*d*] thiazole (**14**) in 51% yield. The corresponding alcohol **15** could be readily prepared in 92% yield upon the removal of the silyl protecting group. The histamine h4 receptor ligand was then obtained in 51% yield via etherification mediated by stoichiometric *p*‐TSA. In addition, carbinoxamine, a marketed drug molecule used for the treatment of severe allergic reactions, could be efficiently prepared in 26% total yield from pyridine *N*‐oxide (**4a**) within four steps via sequentially copper‐catalyzed coupling of acylsilane **6e** and pyridine *N*‐oxide (**4a**), reduction of *N*‐oxide, deprotection of silyl group, and S_N_2 substitution (Scheme 2c).

**Scheme 3:**
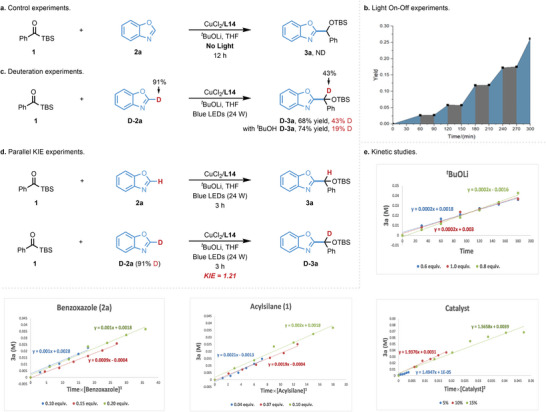
Preliminarily mechanistic studies and kinetic studies.

Preliminary mechanistic studies were conducted to shed light on the mechanism of this novel Cu‐catalyzed photoinduced coupling reaction with azoles. Control experiments indicated the reaction could not happen without photoirradiation, thus ruling out the nucleophilic addition pathway to acylsilanes by azole anion or azole‐copper nucleophiles generated in the presence of strong bases and copper catalyst (**Scheme** **3**a). The UV/VIS analysis of the individual reaction components and the reaction mixture revealed that the acylsilane was the only absorbing species in the visible range, therefore excluding the role of other species as photocatalyst (For more details, see Supporting Information). Moreover, the light on‐off experiment was conducted and the desired product **3a** formed only under continuous irradiation, which ruled out the possibility of a radical chain propagation pathway (Scheme 3b). The photochemical quantum yield (Φ = 0.019) for the current reaction is less than 1.0, which is consistent with the *α*‐siloxy copper carbene‐involved coupling mechanism^[^ ^11d^ ^]^ (For more details, see Supporting Information).

To confirm the hydrogen sources in the products, the experiment using deuterium‐labelled benzoxazole (**D‐2a**) was performed. Although only 43% deuteration rate was observed in the target product, it still revealed that the acidic proton in the benzoxazole could serve as one of the hydrogen sources for this reaction. The low deuteration might originate from the trace amount of water in the solvent or alcohol in the presence of lithium *tert*‐butoxide, as the deuteration rate was reduced to 19% by the addition of a small amount of *tert*‐butanol (Scheme 3c). The KIE experiments with deuterium‐labeled benzoxazole (**D‐2a**) unveiled that the C(sp^2^)–H cleavage of benzoxazole might not be involved in the rate‐determining step (k_H_/k_D_ = 1.21) (Scheme 3d). To further understand the reaction mechanism, the reaction progress kinetic analysis^[^ ^16^ ^]^ (RPKA) was performed (For details, see Supporting Information). The kinetic data indicated the reaction rate is second order to Cu/**L14** catalyst, first order to benzoxazole and acylsilane, which indicated the acylsilane, benzoxazole, and copper catalyst are all involved in rate determining step (Scheme 3e). The second order to Cu/**L14** catalyst disclosed a bimetallic relay process in the catalytic cycle.

**Figure 1:**
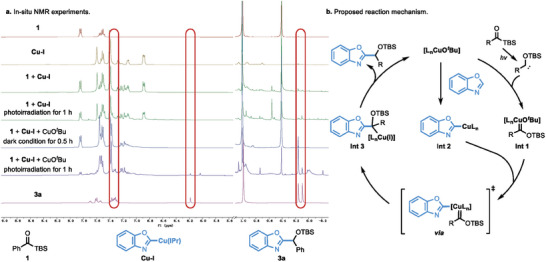
In situ NMR experiments and proposed reaction mechanism.

To validate the bimetallic relay process, we have prepared the IPr ligand stabilized benzoxazole‐Cu(I) species (**Cu‐I**), and explored its reactivity via in situ NMR experiments (**Figure** **1**a). That no reaction happened by mixing benzoylsilane (**1**) with **Cu‐I** ruled out the direct nucleophilic addition of acylsilane with azole‐Cu species. Moreover, the desired product (**3a**) was not observed under photoirradiation, which further excluded the classic mono‐nuclear Cu‐catalyzed coupling mechanism with Fischer Cu‐carbene species (For details, see Supporting Information). Notably, **3a** was observed in 10% NMR yield when one equivalent of Cu(O*^t^* Bu) was added under photoirradiation for 1.0 h, which is consistent with our proposed bimetallic relay mechanism. To further confirm this experiment observation, we have conducted the coupling reaction of benzoxazole‐Cu species **Cu‐I** with benzoylsilane (**1**) in the presence of Cu(O*^t^* Bu) under photoirradiation, giving the desired product **3a** in 44% yield (For more details, see Supporting Information). On the basis of the aforementioned kinetic analysis and experimental observations, a possible mechanism was proposed as depicted in Figure 1b. As Cu(I) catalysts are also reactive under our standard conditions, we believe that our reaction underwent a Cu(I)‐catalyzed coupling process, which could be in situ generated via the reduction of Cu(II) by carbene species.^[^ ^17^ ^]^ The ligand attached Cu(I)‐O*^t^* Bu species could be generated in the presence of LiO*^t^* Bu and siloxyl carbene species. *α*‐Siloxy Fischer Cu‐carbene species **Int 1** was then formed by trapping the in situ generated *α*‐siloxy carbene from acylsilane under photoirradiation. Meanwhile, Cu(I)‐O*^t^* Bu species was reacted with benzoxazole to reach a copper‐azole intermediate **Int 2** which was via the bimetallic relay process, transmetalation process between two organocopper species (**Int 2** and **Int 1**), delivering a key intermediate **Int 3**. Sequentially migratory insertion and protonation were followed to give the desired product. Notably, this bimetallic relay process is the rate‐determining step according to the reaction progress kinetic analysis.

## 3 Conclusion

In summary, we have demonstrated a photoinduced copper‐catalyzed coupling reaction of acylsilanes with azoles or azine *N*‐oxides, delivering a series of azole‐containing or azine‐containing secondary alcohols with broad substrate scopes and remarkable heterocycle and functional group compatibility under redox‐neutral conditions. This reaction represents the first example of a Cu‐catalyzed coupling reaction of electron‐rich Fischer carbene species with hard nucleophiles. The development of novel coupling reactions and their asymmetric versions with the in situ generated electron‐rich Fischer carbenes are ongoing in our laboratory.

## Conflict of Interest

The authors declare no conflict of interest.

## Supporting information



Supporting Information

Supporting cif

## Data Availability

The data that support the findings of this study are available in the supplementary material of this article.
