# Supplementary material for: Photoinduced Copper‐Catalyzed Cross‐Coupling of Acylsilanes with Heteroarenes via Bimetallic Relay
Source: Adv Sci (Weinh). 2024 Oct 14;11(45):2409457. doi: 10.1002/advs.202409457 (PMC11615762; doi:10.1002/advs.202409457)

ADVANCED SCIENCE
Open Access

Supporting Information

for *Adv. Sci.*, DOI 10.1002/advs.202409457

Photoinduced Copper-Catalyzed Cross-Coupling of Acylsilanes with Heteroarenes via Bimetallic Relay

Long Zheng, Ying-Chao Li, Yichen Wu and Peng Wang**

Table of Contents

1. General Information S2
2. Substrate Preparation S3
3. Experimental Optimization S6
    3.1 Base Effects S6
    3.2 Copper Sources S7
    3.3 Solvent Effects S7
    3.4 Ligand Evaluation for Coupling Reaction S8
    3.5 Evaluation of Substrate Ratio S8
    3.6 Control Experiments S9
4. General Procedure for Coupling Reactions S10
5. Scale-Up Reaction and Synthetic Applications S33
    5.1 Scale-Up Reaction S33
    5.2 Synthetic Applications S33
6. Mechanistic Study S38
    6.1 Deuteration Experiments S38
    6.2 KIE Experiments S44
    6.3 UV/Vis Absorption Spectroscopy Studies S45
    6.4 Determination of Quantum Yields S46
    6.5 Light On-Off Experiment S47
    6.6 In-Situ NMR Studies S48
    6.7 Kinetics Data S51
7. X-Ray Structure S59
8. References S60
9. NMR Spectra S61

1. General Information

$CuCl_2$ was purchased from Adamas. Other reagents were purchased from TCI, Sigma-Aldrich, Acros, Adamas-beta, J&K, 9-Ding, Bidepharm and Energy Chemical of the highest purity grade and used without further purification, unless otherwise indicated. Tetrahydrofuran (THF), acetonitrile (CH_3CN), dichloromethane (CH_2Cl_2) and *N,N*-dimethylformamide (DMF) were dried using the solvent purification system. Other anhydrous solvents were purchased from J&K. The extent of reaction was monitored by thin-layer chromatography (TLC), performed on 0.25 mm silica gel HSGF254. The TLC plates were visualized by ultraviolet light (254 nm) or treatment with potassium permanganate or phosphomolybdic acid stain followed by gentle heating.

NMR spectra were recorded on Varian 400, Bruker 400 and Agilent 400 (400 MHz for ^{1}H; 375 MHz for ^{19}F; 100 MHz for ^{13}C) spectrometer. The chemical shifts (δ) were quoted in parts per million (ppm) referenced to TMS (0.0 ppm for ^{1}H NMR), $CDCl_3$ (77.0 ppm for ^{13}C NMR) and $CFCl_3$ (0.0 ppm for ^{19}F NMR). The following abbreviations were used to explain multiplicities: s = singlet, d =doublet, t = triplet, q = quartet, m = multiplet, and br = broad. Coupling constants, *J*, were reported in Hertz unit (Hz). ^{13}C NMR spectra were fully decoupled by broad band proton decoupling. ^{19}F NMR spectra were fully decoupled by broad band proton decoupling. High-resolution mass spectra (HRMS) were recorded on an Agilent Mass spectrometer using ESI-TOF or CI/EI. UV/Vis absorption spectra were recorded on a Varian Cary 100 two-beam photospectrometer using the following parameter set: data interval 1.0 nm, bandwidth 2.0 nm, response time 0.1 s, scan speed 600 nm/min. The photoreactors are manufactured by China Wuhan Ge'ao Chemical Technology Co., Ltd. The wavelength of purple LEDs' peak intensity is 390 nm, and the broadband source is 380-390 nm. The wavelength of blue LEDs' peak intensity is 460 nm, and the broadband source is 450-470 nm. The lamp beads are connected in series by 24 W lamp beads, and the light intensity is about 30 mW/cm^2. The distance from the light source to the irradiation vessel is about 1.0 cm.

2. Substrate Preparation

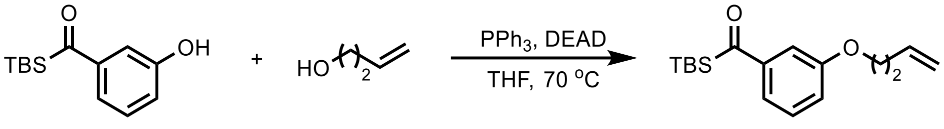

A 25 mL Schlenk flask was charged with (*tert*-butyldimethylsilyl)(3-hydroxyphenyl)methanone (118.0 mg, 0.50 mmol), but-3-en-1-ol (36.0 mg, 0.50 mmol) and PPh_3 (144.0 mg, 0.55 mmol) in THF (5.0 mL), then DEAD (88.0 µL, 0.55 mmol, 1.1 equiv.) was added dropwise at 70 °C in an oil bath. Upon completion, the reaction was quenched with water and extracted with EtOAc (3 × 10 mL). The combined organic layers were dried over Na_2SO_4, and evaporated under reduced pressure. The residue was purified by silica gel chromatography using PE/EA (100/1) as the eluent to give **6k** (0.11 g, 75% yield) as a yellow oil. ^{1}H NMR (400 MHz, $CDCl_3$) δ 7.34 (dt, J = 7.6, 1.2 Hz, 1H), 7.28 (t, J = 7.6 Hz, 1H), 7.21 (dd, J = 2.4, 1.2 Hz, 1H), 6.99 (ddd, J = 8.0, 2.8, 1.2 Hz, 1H), 5.87–5.77 (m, 1H), 5.09 (dq, J = 17.2, 1.6 Hz, 1H), 5.03 (dq, J = 10.4, 1.2 Hz, 1H), 3.97 (t, J = 6.8 Hz, 2H), 2.47 (qt, J = 6.8, 1.2 Hz, 2H), 0.88 (s, 9H), 0.29 (s, 6H); ^{13}C NMR (100 MHz, $CDCl_3$) δ 235.2, 159.1, 144.0, 134.2, 129.4, 121.3, 119.9, 117.1, 111.0, 67.3, 33.5, 26.7, 16.9, -4.7; HRMS (ESI-TOF) *m/z* Calcd for $C_{17}H_{26}O_2NaSi$ $[M+Na]^+$: 313.1594; found: 313.1597.

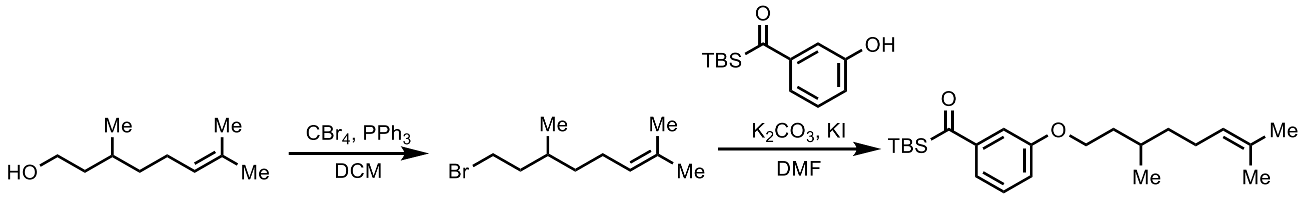

A 100 mL dry flask was charged with CBr_4 (3.65 g, 11.0 mmol), PPh_3 (3.15 g, 12.0 mmol) and anhydrous DCM (50.0 mL) in an ice water bath, then alcohol (1.56 g, 10.0 mmol) was added dropwise. The reaction mixture was stirred at room temperature for 3 hours. Upon completion, the reaction mixture was quenched with water, then extracted with DCM (3 × 20 mL). The combined organic layers were dried over Na_2SO_4, and evaporated under reduced pressure. Then the residue was purified by silica gel chromatography using PE as the eluent to give 8-bromo-2,6-dimethyloct-2-ene (1.43 g, 65% yield) as a colorless oil.

A 25 mL Schlenk flask was charged with (*tert*-butyldimethylsilyl)(3-hydroxyphenyl)methanone (0.24 g, 1.0 mmol), 8-bromo-2,6-dimethyloct-2-ene (0.26 g, 1.2 mmol), K_2CO_3 (0.28 g, 2.0 mmol) and KI (17.0 mg, 0.1 mmol) in DMF (2.0 mL) at 60 °C in an oil bath. Upon completion, the reaction was quenched with water and extracted with EtOAc (3 × 10 mL). The combined organic layers were dried over Na_2SO_4, and evaporated under reduced pressure. The residue was purified by silica gel chromatography using PE/EA (200/1) as the eluent to give **6q** (0.18 g, 47% yield) as a yellow oil. ^{1}H NMR (400 MHz, $CDCl_3$) δ 7.42 (dt, J = 7.6, 1.2 Hz, 1H), 7.36 (t, J = 8.0 Hz, 1H), 7.29 (dd, J = 2.4, 1.2 Hz, 1H), 7.07 (ddd, J = 8.0, 2.4, 1.2 Hz, 1H), 5.11 (tt, J = 6.8, 1.2 Hz, 1H), 4.08–3.99 (m, 2H), 2.09–1.93 (m, 2H), 1.89–1.81 (m, 1H), 1.74–1.65 (m, 4H), 1.63–1.57 (m, 4H), 1.45–1.36 (m, 1H), 1.28–1.18 (m, 1H), 0.97–0.95 (m, 12H), 0.37 (s, 6H); ^{13}C NMR (100 MHz, $CDCl_3$) δ 235.3, 159.4, 144.0, 131.2, 129.4, 124.6, 121.2, 119.9, 110.9, 66.4, 37.1, 36.0, 29.5, 26.7, 25.7, 25.4, 19.5, 17.6, 16.9, -4.7; HRMS (ESI-TOF) *m/z* Calcd for $C_{23}H_{38}O_2NaSi$

$[M+Na]^+$: 397.2533; found: 397.2530.

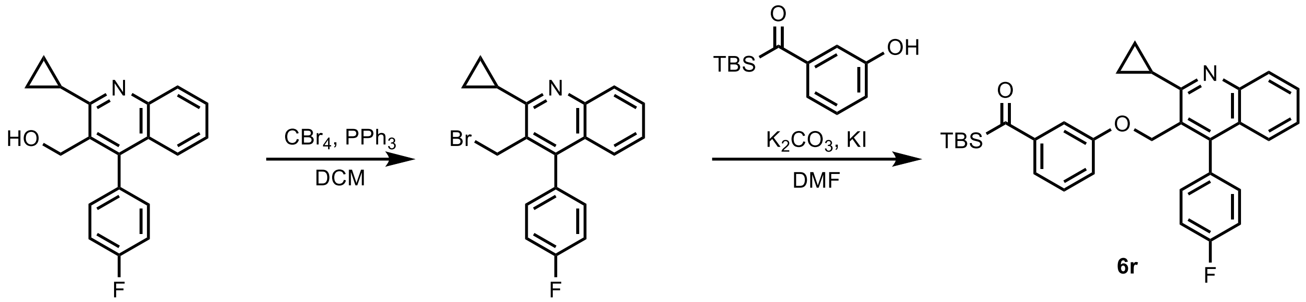

A 50 mL dry flask were charged with CBr_4 (0.73 g, 2.2 mmol), PPh_3 (0.63 g, 2.4 mmol) and anhydrous DCM (10.0 mL) in an ice water bath, then alcohol (0.59 g, 2.0 mmol) was added. The reaction mixture was stirred at room temperature for 2.0 hours. Upon completion, the reaction mixture was quenched with water, then extracted with DCM (3 × 20 mL). The combined organic layers were dried over Na_2SO_4 and evaporated under reduced pressure. Then the residue was purified by silica gel chromatography using PE/EA (50/1) as the eluent to give 3-(bromomethyl)-2-cyclopropyl-4-(4-fluorophenyl)quinoline (0.33 g, 47% yield) as a white solid.

A 10 mL Schlenk flask were charged with (*tert*-butyldimethylsilyl)(3-hydroxyphenyl)methanone (0.12 g, 0.5 mmol), 3-(bromomethyl)-2-cyclopropyl-4-(4-fluorophenyl)quinoline (0.20 g, 0.55 mmol), K_2CO_3 (0.14 g, 1.0 mmol) and KI (8.0 mg, 0.05 mmol) in DMF (1.0 mL) at 60 °C in an oil bath. Upon completion, the reaction was quenched with water and extracted with EtOAc (3 × 10 mL). The combined organic layers were dried over Na_2SO_4, and evaporated under reduced pressure. The residue was purified by silica gel chromatography using PE/EA (100/1) as the eluent to give **6r** (0.18 g, 70% yield) as a yellow oil. ^1H NMR (400 MHz, $CDCl_3$) δ 8.00 (d, J = 8.4 Hz, 1H), 7.66–7.61 (m, 1H), 7.47 (dt, J = 7.6, 1.2 Hz, 1H), 7.40–7.28 (m, 6H), 7.17–7.11 (m, 2H), 7.09 (ddd, J = 8.0, 2.8, 1.2 Hz, 1H), 5.06 (s, 2H), 2.41–2.34 (m, 1H), 1.40–1.36 (m, 2H), 1.04–1.00 (m, 2H), 0.96 (s, 9H), 0.35 (s, 6H); ^{13}C NMR (100 MHz, $CDCl_3$) δ 235.2, 162.6 (d, J = 247 Hz), 162.5, 158.9, 147.7, 147.6, 143.9, 131.9 (d, J = 4 Hz), 131.4 (d, J = 8 Hz), 129.6, 129.4, 129.0, 126.4, 125.9, 125.54, 125.49, 122.0, 120.2, 115.4 (d, J = 22 Hz), 111.2, 65.3, 26.7, 16.9, 14.6, 9.7, -4.7; ^{19}F NMR (375 MHz, $CDCl_3$) δ -113.95; HRMS (ESI-TOF) *m/z* Calcd for $C_{32}H_{35}NO_2FSi$ $[M+H]^+$: 512.2416; found: 512.2407.

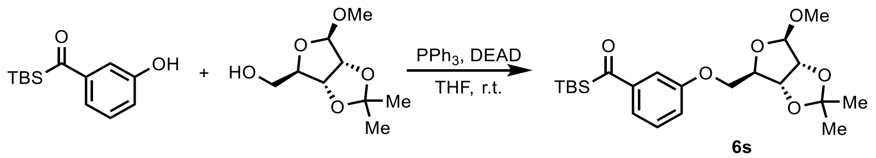

A 25 mL Schlenk flask were charged with (*tert*-butyldimethylsilyl)(3-hydroxyphenyl)methanone (0.12 g, 0.50 mmol), ((3a*R*,4*R*,6*R*,6a*R*)-6-methoxy-2,2-dimethyltetrahydrofuro[3,4-*d*][1,3]dioxol-4-yl)methanol (0.10 g, 0.50 mmol) and PPh_3 (0.14 g, 0.55 mmol) in THF (5.0 mL), then DEAD (88.0 μL, 0.55 mmol) was added dropwise at room temperature. Upon completion, the reaction was quenched with water and extracted with EtOAc (3 × 10 mL). The combined organic layers were dried over Na_2SO_4 and evaporated under reduced pressure. The residue was purified by silica gel chromatography using PE/EA (20/1) as the eluent to give **6s** (0.14 g, 66% yield) as a yellow oil. ^1H NMR (400 MHz, $CDCl_3$) δ 7.46 (dt, J = 7.6, 1.2 Hz, 1H), 7.38 (t, J = 8.0 Hz, 1H), 7.29 (dd, J = 2.8, 1.2 Hz, 1H), 7.11 (ddd,

J = 8.0, 2.8, 1.2 Hz, 1H), 5.03 (s, 1H), 4.80 (d, *J* = 6.0 Hz, 1H), 4.64 (d, *J* = 6.0 Hz, 1H), 4.55 (dd, *J* = 8.4, 6.0 Hz, 1H), 4.06 (dd, *J* = 9.6, 6.0 Hz, 1H), 3.98 (t, *J* = 9.6 Hz, 1H), 3.34 (s, 3H), 1.52 (s, 3H), 1.35 (s, 3H), 0.97 (s, 9H), 0.37 (s, 6H); ^{13}C NMR (100 MHz, $CDCl_3$) δ 235.2, 158.7, 144.0, 129.6, 121.8, 120.0, 112.5, 110.8, 109.4, 85.1, 84.4, 82.1, 68.4, 54.9, 26.7, 26.4, 24.9, 16.9, -4.7; HRMS (ESI-TOF) *m/z* Calcd for $C_{22}H_{34}O_6NaSi$ $[M+Na]^+$: 445.2017; found: 445.2018.

3. Experimental Optimization

3.1 Base Effects[a,b]

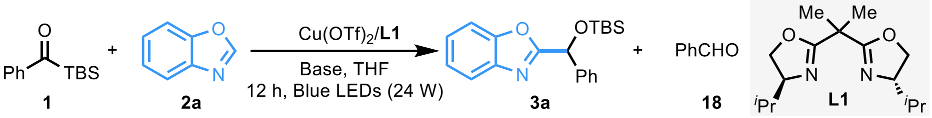

Entry	Base	Yield of **3a** (%)	Conv. of **1** (%)	Conv. of **2a** (%)	Yield of **18** (%)
1	Na_2CO_3	-	95	18	trace
2	Cs_2CO_3	-	40	8	trace
3	$CsHCO_3$	-	74	17	trace
4	NaOH	-	100	31	19
5	^{t}BuOLi	35	70	76	-
6	^{t}BuONa	17	79	77	-
7	^{t}BuOK	18	100	85	-
8	MeOLi	-	96	15	18
9	MeONa	-	93	54	6
10	MeOK	trace	71	36	12

[a]**1** (0.1 mmol, 1.0 equiv.), **2a** (0.1 mmol, 1.0 equiv.), $Cu(OTf)_2$ (10 mol %), **L1** (12 mol %), base (0.1 mmol, 1.0 equiv.), THF (1.0 mL), Blue LEDs (450-470 nm, 24 W), 12 h. [b]Yield was determined by ^{1}H NMR using CH_2Br_2 as the internal standard.

3.2 Copper Sources[a,b]

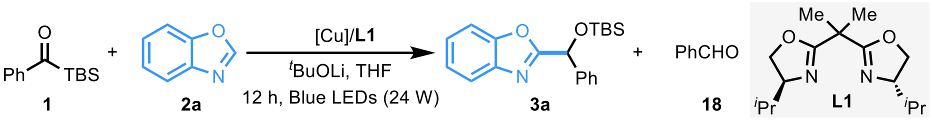

Entry	[Cu]	Yield of **3a** (%)	Conv. of **1** (%)	Conv. of **2a** (%)	Yield of **18** (%)
1	CuCl	6	9	42	-
2	CuBr	13	35	49	-
3	CuI	23	64	53	-
4	$CuPF_6$•4CH_3CN	30	90	73	-
5	Cu(OTf)•0.5Toluene	29	58	76	-
6	$CuCl_2$	37	69	87	-
7	$CuBr_2$	31	81	84	-
8	$CuCl_2$, $AgPbF_6$	3	86	46	-
9	$CuCl_2$, $AgSbF_6$	2	79	64	-
10	$CuCl_2$, NaBArF	15	100	51	6
11	$Cu(OTf)_2$	35	70	76	-

[a]**1** (0.1 mmol, 1.0 equiv.), **2a** (0.1 mmol, 1.0 equiv.), [Cu] (10 mol %), **L1** (12 mol %), tBuOLi (0.1 mmol, 1.0 equiv.), THF (1.0 mL), Blue LEDs (450-470 nm, 24 W), 12 h. [b]Yield was determined by ^1H NMR using CH_2Br_2 as the internal standard.

3.3 Solvent Effects[a,b]

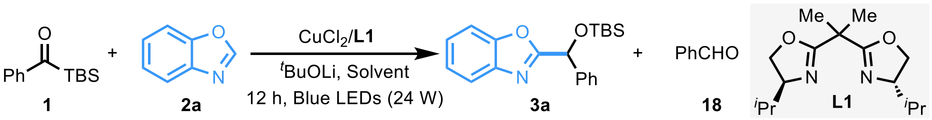

Entry	Solvent	Yield of **3a** (%)	Conv. of **1** (%)	Conv. of **2a** (%)	Yield of **18** (%)
1	DCM	2	100	35	-
2	Toluene	-	100	38	-
3	Et_2O	3	100	23	-
4	THF	37	69	87	-
5	Dioxane	22	100	51	-
6	Hexane	9	69	35	4
7	CH_3CN	8	100	28	-

[a]**1** (0.1 mmol, 1.0 equiv.), **2a** (0.1 mmol, 1.0 equiv.), $CuCl_2$ (10 mol %), **L1** (12 mol %), tBuOLi (0.1 mmol, 1.0 equiv.), Solvent (1.0 mL), Blue LEDs (450-470 nm, 24 W), 12 h. [b]Yield was determined by ^1H NMR using CH_2Br_2 as the internal standard.

3.4 Ligand Evaluation for Coupling Reaction[a,b]

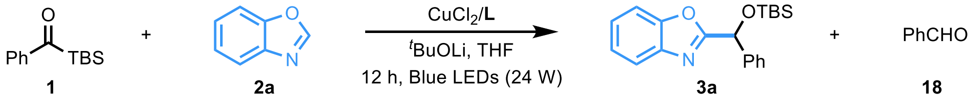

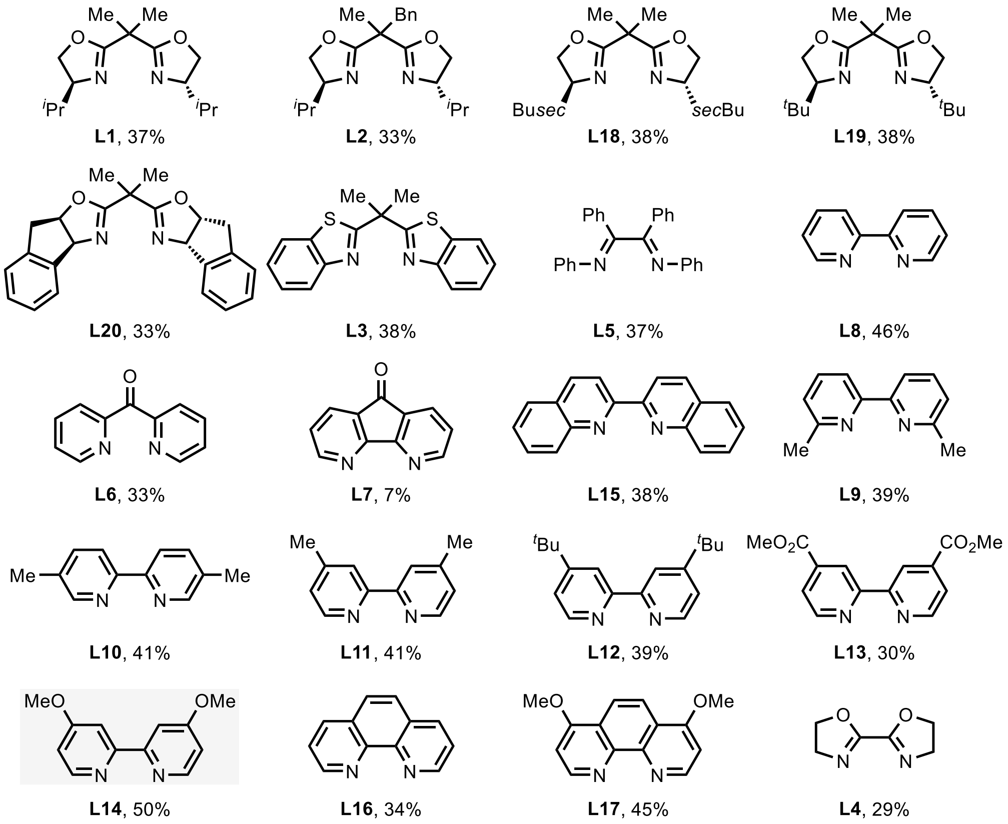

[a]**1** (0.1 mmol, 1.0 equiv.), **2a** (0.1 mmol, 1.0 equiv.), $CuCl_2$ (10 mol %), **L** (12 mol %), ^{t}BuOLi (0.1 mmol, 1.0 equiv.), THF (1.0 mL), Blue LEDs (450-470 nm, 24 W), 12 h. [b]Yield was determined by ^{1}H NMR using CH_2Br_2 as the internal standard.

3.5 Evaluation of Substrate Ratio[a,b]

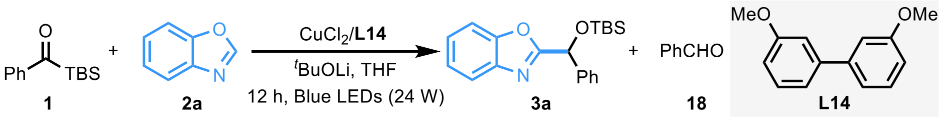

Entry	**1**/**2a** ratio	Yield of **3a** (%)	Conv. of **1** (%)	Conv. of **2a** (%)	Yield of **18** (%)
1	1.5/1.0	55	78	95	-
2	1.2/1.0	56	78	87	-
3	1.0/1.0	50	81	72	-
4	1.0/1.5	64	93	63	-
5	1.0/2.0	85 (78)[c]	100	58	-

[a]**1** , **2a** , $CuCl_2$ (10 mol %), **L14** (12 mol %), ^{t}BuOLi (0.1 mmol, 1.0 equiv.), THF (1.0 mL), Blue LEDs (450-470 nm, 24 W), 12 h. [b]Yield was determined by ^{1}H NMR using CH_2Br_2 as the internal standard. [c]Isolated yield.

3.6 Control Experiments[a,b]

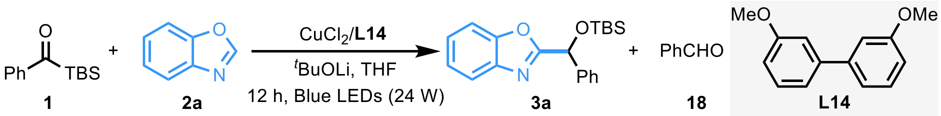

Entry	**Conditions**	Yield of **3a** (%)	Conv. of **1** (%)	Conv. of **2a** (%)	Yield of **18** (%)
1	None	85 (78)[c]	100	58	-
2	No Light	-	12	19	-
3	No Catalyst	6	27	21	-
4	No Base	-	36	-	20
5	No Ligand	65	96	62	-
6	No Catalyst and Light	trace	22	12	-

[a]**1** (0.1 mmol, 1.0 equiv.), **2a** (0.2 mmol, 2.0 equiv.), $CuCl_2$ (10 mol %), **L14** (12 mol %), ^{t}BuOLi (0.1 mmol, 1.0 equiv.), THF (1.0 mL), Blue LEDs (450-470 nm, 24 W), 12 h. [b]Yield was determined by ^{1}H NMR using CH_2Br_2 as the internal standard. [c]Isolated yield.

4. General Procedure for Coupling Reactions

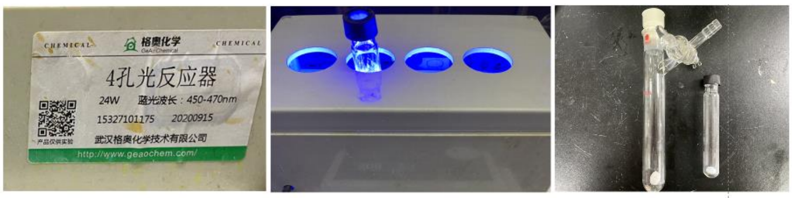

Photo reactor | Reaction In-Process | left: for **General Procedure** right: for **Scale-Up Synthesis**

Figure S1 Graphic Supporting Information for Reaction Setup

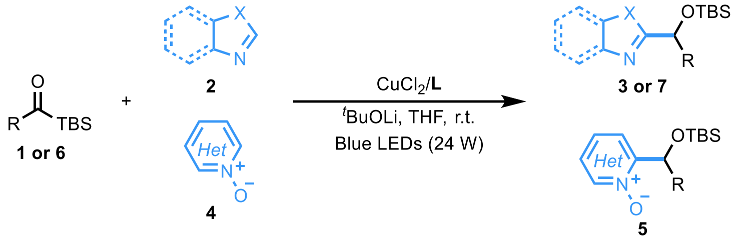

General Procedure for Coupling Reactions. A 10-mL tube were charged with $CuCl_2$ (1.3 mg, 10 mol%), **L14** (2.6 mg, 12 mol%) and ^{t}BuOLi (8.0 mg, 0.1 mmol) under N_2, followed by the addition of THF (1.0 mL). The tube was stirred at room temperature for 2.0 hours. To the above mixture were added **1** or **6** (0.1 mmol, 1.0 equiv.) and **2** or **4** (0.2 mmol, 2.0 equiv.) sequentially under N_2. The resulted reaction mixture was stirred under the irradiation of blue LEDs (24 W, 450-470 nm) for 12 hours. Upon completion, the reaction mixture was passed through a pad of silica gel with EtOAc as the eluent to remove the copper catalyst and the insoluble precipitate. The resulted solution was evaporated under reduced pressure, and the residue was purified by silica gel chromatography or preparative thin-layer chromatography as mentioned.

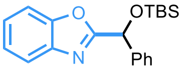

3a

2-(((*tert*-Butyldimethylsilyl)oxy)(phenyl)methyl)benzo[*d*]oxazole

3a was synthesized following the general procedure under the irradiation of blue LEDs. After purification by preparative thin-layer chromatography using PE/EA (30/1) as the eluent, **3a** was obtained in 78% yield (26.4 mg) as a white solid.

^{1}H NMR (400 MHz, $CDCl_3$) δ 7.74–7.70 (m, 1H), 7.58–7.56 (m, 2H), 7.51–7.46 (m, 1H), 7.38–7.35 (m, 2H), 7.32–7.27 (m, 3H), 6.09 (s, 1H), 0.93 (s, 9H), 0.12 (s, 3H), 0.05 (s, 3H); ^{13}C NMR (100 MHz, $CDCl_3$) δ 166.2, 150.8, 140.8, 139.6, 128.5, 128.2, 126.1, 125.1, 124.3, 120.3, 110.9, 71.2, 25.7, 18.3, -5.1, -5.2; HRMS (ESI-TOF) *m/z* Calcd for $C_{20}H_{25}NO_2NaSi$ $[M+Na]^+$: 362.1547; found: 362.1544.

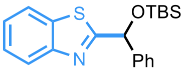

3b

2-(((*tert*-Butyldimethylsilyl)oxy)(phenyl)methyl)benzo[*d*]thiazole

3b was synthesized following the general procedure using **1** (0.12 mol, 1.2 equiv.), **2b** (0.1 mmol, 1.0 equiv.) and ^{t}BuOLi (16.0 mg, 0.2 mmol) under the irradiation of blue LEDs. After purification by preparative thin-layer

chromatography using PE/EA (30/1) as the eluent, **3b** was obtained in 54% yield (19.3 mg) as a white solid.

^{1}H NMR (400 MHz, $CDCl_3$) δ 7.94 (d, *J* = 7.6 Hz, 1H), 7.85 (d, *J* = 7.2 Hz, 1H), 7.56 (d, *J* = 7.6 Hz, 2H), 7.44–7.39 (m, 1H), 7.36–7.30 (m, 3H), 7.28–7.24 (m, 1H), 6.14 (s, 1H), 0.97 (s, 9H), 0.11 (s, 3H), 0.02 (s, 3H); ^{13}C NMR (100 MHz, $CDCl_3$) δ 177.4, 153.4, 141.7, 134.9, 128.5, 128.0, 126.3, 125.7, 124.7, 123.0, 121.7, 75.3, 25.7, 18.2, -4.9, -5.1; HRMS (ESI-TOF) *m/z* Calcd for $C_{20}H_{25}NOSNaSi$ $[M+Na]^+$: 378.1318; found: 378.1321

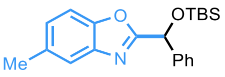

3c

2-(((*tert*-Butyldimethylsilyl)oxy)(phenyl)methyl)-5-methylbenzo[*d*]oxazole

3c was synthesized following the general procedure using ^{t}BuOLi (6.4 mg, 0.08 mmol) under the irradiation of blue LEDs. After purification by preparative thin-layer chromatography using PE/EA (30/1) as the eluent, **3c** was obtained in 57% yield (20.3 mg) as a yellow oil.

^{1}H NMR (400 MHz, $CDCl_3$) δ 7.57–7.55 (m, 2H), 7.50 (s, 1H), 7.38–7.34 (m, 3H), 7.31–7.27 (m, 1H), 7.10 (dd, *J* = 8.0, 1.6 Hz, 1H), 6.07 (s, 1H), 2.44 (s, 3H), 0.92 (s, 9H), 0.11 (s, 3H), 0.03 (s, 3H); ^{13}C NMR (100 MHz, $CDCl_3$) δ 166.2, 149.1, 141.0, 139.7, 134.1, 128.4, 128.1, 126.2, 126.1, 120.2, 110.2, 71.1, 25.7, 21.4, 18.3, -5.1, -5.2; HRMS (ESI-TOF) *m/z* Calcd for $C_{21}H_{27}NO_2NaSi$ $[M+Na]^+$: 376.1703; found: 376.1703.

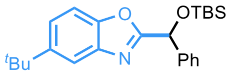

3d

5-(*tert*-Butyl)-2-(((*tert*-butyldimethylsilyl)oxy)(phenyl)methyl)benzo[*d*]oxazole

3d was synthesized following the general procedure under the irradiation of blue LEDs. After purification by preparative thin-layer chromatography using PE/EA (30/1) as the eluent, **3d** was obtained in 74% yield (29.2 mg) as a white solid.

^{1}H NMR (400 MHz, $CDCl_3$) δ 7.73 (s, 1H), 7.56 (d, *J* = 7.6 Hz, 2H), 7.41–7.33 (m, 4H), 7.29–7.25 (m, 1H), 6.07 (s, 1H), 1.35 (s, 9H), 0.93 (s, 9H), 0.12 (s, 3H), 0.05 (s, 3H); ^{13}C NMR (100 MHz, $CDCl_3$) δ 166.3, 148.8, 147.8, 140.7, 139.7, 128.4, 128.1, 126.1, 122.8, 116.7, 110.0, 71.2, 34.9, 31.7, 25.7, 18.3, -5.1; HRMS (ESI-TOF) *m/z* Calcd for $C_{24}H_{33}NO_2NaSi$ $[M+Na]^+$: 418.2173; found: 418.2171.

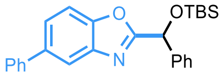

3e

2-(((*tert*-Butyldimethylsilyl)oxy)(phenyl)methyl)-5-phenylbenzo[*d*]oxazole

3e was synthesized following the general procedure under the irradiation of blue LEDs. After purification by preparative thin-layer chromatography using PE/EA (100/1) as the eluent, **3e** was obtained in 46% yield (19.2 mg) as a white solid.

^{1}H NMR (400 MHz, $CDCl_3$) δ 7.91 (s, 1H), 7.60–7.58 (m, 4H), 7.53 (d, *J* = 1.2 Hz, 2H), 7.44 (t, *J* = 8.0 Hz, 2H), 7.39–7.35 (m, 3H), 7.33–7.28 (m, 1H), 6.11 (s, 1H), 0.94 (s, 9H), 0.13 (s, 3H), 0.07 (s, 3H); ^{13}C NMR (100 MHz,

CDCl3) δ 166.8, 150.4, 141.4, 141.0, 139.6, 138.3, 128.8, 128.5, 128.2, 127.4, 127.2, 126.1, 124.8, 118.8, 110.9, 71.2, 25.7, 18.4, -5.09, -5.11; HRMS (ESI-TOF) *m/z* Calcd for $C_{26}H_{29}NO_2NaSi$ $[M+Na]^+$: 438.1860; found: 438.1861.

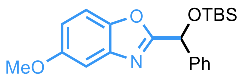

3f

2-(((*tert*-Butyldimethylsilyl)oxy)(phenyl)methyl)-5-methoxybenzo[*d*]oxazole

3f was synthesized following the general procedure under the irradiation of blue LEDs. After purification by preparative thin-layer chromatography using PE/EA (30/1) as the eluent, **3f** was obtained in 77% yield (28.4 mg) as a white solid.

^{1}H NMR (400 MHz, $CDCl_3$) δ 7.55 (d, J = 7.2 Hz, 2H), 7.38–7.34 (m, 3H), 7.31–7.27 (m, 1H), 7.20 (d, J = 2.4 Hz, 1H), 6.90 (dd, J = 8.8, 2.4 Hz, 1H), 6.05 (s, 1H), 3.82 (s, 3H), 0.93 (s, 9H), 0.11 (s, 3H), 0.04 (s, 3H); ^{13}C NMR (100 MHz, $CDCl_3$) δ 166.9, 157.1, 145.5, 141.6, 139.7, 128.5, 128.1, 126.1, 113.7, 111.0, 103.2, 71.1, 55.9, 25.7, 18.3, -5.1, -5.2; HRMS (ESI-TOF) *m/z* Calcd for $C_{21}H_{27}NO_3NaSi$ $[M+Na]^+$: 392.1652; found: 392.1658.

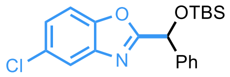

3g

2-(((*tert*-Butyldimethylsilyl)oxy)(phenyl)methyl)-5-chlorobenzo[*d*]oxazole

3g was synthesized following the general procedure using **1** (0.12 mol, 1.2 equiv.), **2g** (0.1 mmol, 1.0 equiv.), CuBr (1.4 mg, 10 mol%) and ^{t}BuOLi (6.4 mg, 0.08 mmol) under the irradiation of blue LEDs. After purification by preparative thin-layer chromatography using PE/EA (30/1) as the eluent, **3g** was obtained in 41% yield (15.2 mg) as a yellow oil.

^{1}H NMR (400 MHz, $CDCl_3$) δ 7.69 (d, J = 2.0 Hz, 1H), 7.56–7.54 (m, 2H), 7.41–7.35 (m, 3H), 7.33–7.26 (m, 2H), 6.06 (s, 1H), 0.92 (s, 9H), 0.11 (s, 3H), 0.04 (s, 3H); ^{13}C NMR (100 MHz, $CDCl_3$) δ 167.6, 149.4, 141.9, 139.3, 129.8, 128.55, 128.3, 126.1, 125.5, 120.3, 111.6, 71.1, 25.7, 18.3, -5.1, -5.2; HRMS (ESI-TOF) *m/z* Calcd for $C_{20}H_{25}NO_2ClSi$ $[M+H]^+$: 374.1338; found: 374.1336.

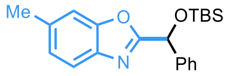

3h

2-(((*tert*-Butyldimethylsilyl)oxy)(phenyl)methyl)-6-methylbenzo[*d*]oxazole

3h was synthesized following the general procedure using ^{t}BuOLi (6.4 mg, 0.08 mmol) under the irradiation of blue LEDs. After purification by preparative thin-layer chromatography using PE/EA (30/1) as the eluent, **3h** was obtained in 52% yield (18.4 mg) as a white solid.

^{1}H NMR (400 MHz, $CDCl_3$) δ 7.58–7.54 (m, 3H), 7.36 (t, J = 7.2 Hz, 2H), 7.30–7.25 (m, 2H), 7.11 (d, J = 8.0 Hz, 1H), 6.06 (s, 1H), 2.45 (s, 3H), 0.93 (s, 9H), 0.11 (s, 3H), 0.04 (s, 3H); ^{13}C NMR (100 MHz, $CDCl_3$) δ 165.6, 151.1, 139.7, 138.6, 135.5, 128.4, 128.1, 126.1, 125.5, 119.6, 111.0, 71.1, 25.7, 21.7, 18.3, -5.1, -5.2; HRMS (ESI-TOF) *m/z* Calcd for $C_{21}H_{27}NO_2NaSi$ $[M+Na]^+$: 376.1703; found: 376.1709.

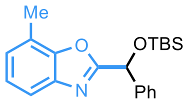

3i

2-(((*tert*-Butyldimethylsilyl)oxy)(phenyl)methyl)-7-methylbenzo[*d*]oxazole

3i was synthesized following the general procedure under the irradiation of blue LEDs. After purification by preparative thin-layer chromatography using PE/EA (30/1) as the eluent, **3i** was obtained in 76% yield (27.0 mg) as a white solid.

^{1}H NMR (400 MHz, $CDCl_3$) δ 7.57 (d, *J* = 7.6 Hz, 2H), 7.53 (d, *J* = 8.0 Hz, 1H), 7.39–7.34 (m, 2H), 7.31–7.27 (m, 1H), 7.19 (t, *J* = 8.0 Hz, 1H), 7.09 (d, *J* = 7.2 Hz, 1H), 6.08 (s, 1H), 2.48 (s, 3H), 0.94 (s, 9H), 0.11 (s, 3H), 0.05 (s, 3H); ^{13}C NMR (100 MHz, $CDCl_3$) δ 165.9, 150.0, 140.4, 139.8, 128.4, 128.1, 126.2, 126.0, 124.2, 121.4, 117.6, 71.2, 25.7, 18.3, 15.1, -5.1, -5.2; HRMS (ESI-TOF) *m/z* Calcd for $C_{21}H_{27}NO_2NaSi$ $[M+Na]^+$: 376.1703; found: 376.1695.

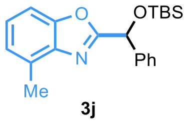

3j

2-(((*tert*-Butyldimethylsilyl)oxy)(phenyl)methyl)-4-methylbenzo[*d*]oxazole

3j was synthesized following the general procedure using ^{t}BuONa (9.6 mg, 0.10 mmol) under the irradiation of blue LEDs. After purification by preparative thin-layer chromatography using PE/EA (30/1) as the eluent, **3j** was obtained in 49% yield (17.3 mg) as a colorless oil.

^{1}H NMR (400 MHz, $CDCl_3$) δ 7.58 (d, *J* = 7.6 Hz, 2H), 7.36 (t, *J* = 8.0 Hz, 2H), 7.31–7.25 (m, 2H), 7.19 (t, *J* = 8.0 Hz, 1H), 7.10 (d, *J* = 7.2 Hz, 1H), 6.13 (s, 1H), 2.62 (s, 3H), 0.93 (s, 9H), 0.12 (s, 3H), 0.04 (s, 3H); ^{13}C NMR (100 MHz, $CDCl_3$) δ 165.2, 150.6, 140.0, 139.8, 130.7, 128.4, 128.0, 126.1, 124.8, 124.7, 108.2, 71.2, 25.71, 18.3, 16.6, -5.10, -5.13; HRMS (ESI-TOF) *m/z* Calcd for $C_{21}H_{27}NO_2NaSi$ $[M+Na]^+$: 376.1703; found: 376.1706.

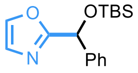

3k

2-(((*tert*-Butyldimethylsilyl)oxy)(phenyl)methyl)oxazole

3k was synthesized following the general procedure using ^{t}BuOLi (16.0 mg, 0.2 mmol) with THF (0.5 mL) under the irradiation of blue LEDs. After purification by preparative thin-layer chromatography using PE/EA (30/1) as the eluent, **3k** was obtained in 39% yield (11.3 mg) as a white solid.

^{1}H NMR (400 MHz, $CDCl_3$) δ 7.58 (d, *J* = 0.8 Hz, 1H), 7.49–7.46 (m, 2H), 7.38–7.34 (m, 2H), 7.31–7.27 (m, 1H), 7.05 (d, *J* = 0.8 Hz, 1H), 5.96 (s, 1H), 0.91 (s, 9H), 0.08 (s, 3H), 0.01 (s, 3H); ^{13}C NMR (100 MHz, $CDCl_3$) δ 164.5, 140.1, 139.0, 128.4, 127.9, 126.9, 126.0, 70.4, 25.7, 18.3, -5.2, -5.3; HRMS (ESI-TOF) *m/z* Calcd for $C_{16}H_{23}NO_2NaSi$ $[M+Na]^+$: 312.1390; found: 312.1393.

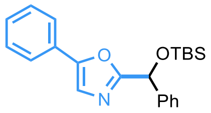

3l

2-(((*tert*-Butyldimethylsilyl)oxy)(phenyl)methyl)-5-phenyloxazole

3l was synthesized following the general procedure under the irradiation of blue LEDs. After purification by preparative thin-layer chromatography using PE/EA (30/1) as the eluent, **3l** was obtained in 60% yield (22.0 mg) as a yellow oil.

^{1}H NMR (400 MHz, $CDCl_3$) δ 7.60 (d, *J* = 7.2 Hz, 2H), 7.53 (d, *J* = 7.2 Hz, 2H), 7.40 (d, *J* = 7.6 Hz, 2H), 7.36 (d, *J* = 7.6 Hz, 2H), 7.32–7.28 (m, 2H), 7.26 (s, 1H) 5.97 (s, 1H), 0.94 (s, 9H), 0.11 (s, 3H), 0.05 (s, 3H); ^{13}C NMR (100 MHz, $CDCl_3$) δ 140.1, 128.8, 128.4, 127.9, 126.1, 124.2, 121.8, 70.7, 25.7, 18.3, -5.1, -5.2; HRMS (ESI-TOF) *m/z* Calcd for $C_{22}H_{27}NO_2NaSi$ $[M+Na]^+$: 388.1703; found: 388.1705.

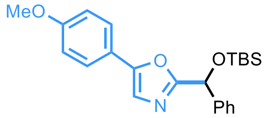

3m

2-(((*tert*-Butyldimethylsilyl)oxy)(phenyl)methyl)-5-(4-methoxyphenyl)oxazole

3m was synthesized following the general procedure under the irradiation of blue LEDs. After purification by preparative thin-layer chromatography using PE/EA (30/1) as the eluent, **3m** was obtained in 44% yield (17.5 mg) as a yellow oil.

^{1}H NMR (400 MHz, $CDCl_3$) δ 7.53–7.51 (m, 4H), 7.39–7.35 (m, 2H), 7.31–7.27 (m, 1H), 7.13 (s, 1H), 6.93–6.90 (m, 2H), 5.95 (s, 1H), 3.82 (s, 3H), 0.94 (s, 9H), 0.10 (s, 3H), 0.05 (s, 3H); ^{13}C NMR (100 MHz, $CDCl_3$) δ 163.2, 159.7, 151.6, 140.2, 128.3, 127.9, 126.1, 125.7, 120.8, 120.3, 114.3, 70.6, 55.3, 25.7, 18.3, -5.1, -5.2; HRMS (ESI-TOF) *m/z* Calcd for $C_{23}H_{29}NO_3NaSi$ $[M+Na]^+$: 418.1809; found: 418.1813.

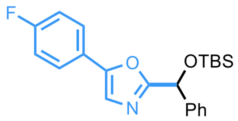

3n

2-(((*tert*-Butyldimethylsilyl)oxy)(phenyl)methyl)-5-(4-fluorophenyl)oxazole

3n was synthesized following the general procedure under the irradiation of blue LEDs. After purification by preparative thin-layer chromatography using PE/EA (30/1) as the eluent, **3n** was obtained in 59% yield (22.5 mg) as a yellow oil.

^{1}H NMR (400 MHz, $CDCl_3$) δ 7.59–7.51 (m, 4H), 7.39–7.35 (m, 2H), 7.32–7.28 (m, 1H), 7.19 (s, 1H), 7.11–7.06 (m, 2H), 5.96 (s, 1H), 0.94 (s, 9H), 0.10 (s, 3H), 0.05 (s, 3H); ^{13}C NMR (100 MHz, $CDCl_3$) δ 163.8, 162.6 (d, *J* = 247 Hz), 150.8, 140.0, 128.4, 128.0, 126.1, 126.0, 124.3 (d, *J* = 3 Hz), 121.4 (d, *J* = 1 Hz), 116.0 (d, *J* = 22 Hz), 70.6, 25.7, 18.3, -5.1, -5.2; ^{19}F NMR (375 MHz, $CDCl_3$) δ -112.80; HRMS (ESI-TOF) *m/z* Calcd for $C_{22}H_{26}NO_2FNaSi$ $[M+Na]^+$: 406.1609; found: 406.1617

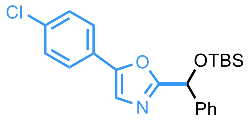

3o

(((*tert*-Butyldimethylsilyl)oxy)(phenyl)methyl)-5-(4-chlorophenyl)oxazole

3o was synthesized following the general procedure under the irradiation of blue LEDs. After purification by preparative thin-layer chromatography using PE/EA (30/1) as the eluent, **3o** was obtained in 66% yield (26.5 mg) as a white solid.

^{1}H NMR (400 MHz, $CDCl_3$) δ 7.53–7.50 (m, 4H), 7.39–7.34 (m, 4H), 7.32–7.28 (m, 1H), 7.24 (s, 1H), 5.96 (s, 1H), 0.94 (s, 9H), 0.10 (s, 3H), 0.05 (s, 3H); ^{13}C NMR (100 MHz, $CDCl_3$) δ 164.1, 150.6, 140.0, 134.1, 129.1, 128.4, 128.0, 126.4, 126.1, 125.4, 122.2, 70.6, 25.7, 18.3, -5.1, -5.2; HRMS (ESI-TOF) *m/z* Calcd for $C_{22}H_{26}NO_2NaClSi$ $[M+Na]^+$: 422.1314; found: 422.1321.

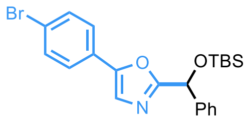

3p

5-(4-Bromophenyl)-2-(((*tert*-butyldimethylsilyl)oxy)(phenyl)methyl)oxazole

3p was synthesized following the general procedure under the irradiation of blue LEDs. After purification by preparative thin-layer chromatography using PE/EA (30/1) as the eluent, **3p** was obtained in 59% yield (26.4 mg) as a white solid.

^{1}H NMR (400 MHz, $CDCl_3$) δ 7.53–7.50 (m, 4H), 7.46–7.43 (m, 2H), 7.39–7.35 (m, 2H), 7.32–7.28 (m, 1H), 7.26 (s, 1H), 5.96 (s, 1H), 0.93 (s, 9H), 0.10 (s, 3H), 0.05 (s, 3H); ^{13}C NMR (100 MHz, $CDCl_3$) δ 164.2, 150.6, 139.9, 132.0, 128.4, 128.0, 126.8, 126.1, 125.6, 122.27, 122.26, 70.6, 25.7, 18.3, -5.1, -5.2; HRMS (ESI-TOF) *m/z* Calcd for $C_{22}H_{26}NO_2NaBrSi$ $[M+Na]^+$: 466.0808; found: 466.0803.

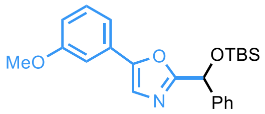

3q

2-(((*tert*-Butyldimethylsilyl)oxy)(phenyl)methyl)-5-(3-methoxyphenyl)oxazole

3q was synthesized following the general procedure under the irradiation of blue LEDs. After purification by preparative thin-layer chromatography using PE/EA (30/1) as the eluent, **3q** was obtained in 54% yield (21.2 mg) as a colorless oil.

^{1}H NMR (400 MHz, $CDCl_3$) δ 7.52 (d, J = 7.6 Hz, 2H), 7.39–7.35 (m, 2H), 7.32–7.28 (m, 2H), 7.25 (s, 1H), 7.20–7.18 (m, 1H), 7.13–7.12 (m, 1H), 6.85 (dd, J = 8.0, 2.4 Hz, 1H), 5.96 (s, 1H), 3.83 (s, 3H), 0.94 (s, 9H), 0.10 (s, 3H), 0.05 (s, 3H); ^{13}C NMR (100 MHz, $CDCl_3$) δ 163.9, 159.9, 151.4, 140.1, 130.0, 129.2, 128.4, 128.0, 126.2, 122.1, 116.7, 114.0, 109.6, 70.7, 55.3, 25.7, 18.3, -5.1, -5.2; HRMS (ESI-TOF) *m/z* Calcd for $C_{23}H_{29}NO_3NaSi$ $[M+Na]^+$: 418.1809; found: 418.1802.

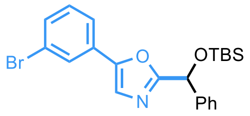

3r

5-(3-Bromophenyl)-2-(((*tert*-butyldimethylsilyl)oxy)(phenyl)methyl)oxazole

3r was synthesized following the general procedure under the irradiation of blue LEDs. After purification by preparative thin-layer chromatography using PE/EA (30/1) as the eluent, **3r** was obtained in 72% yield (32.1 mg) as a yellow oil.

^{1}H NMR (400 MHz, $CDCl_3$) δ 7.73 (t, J = 2.0 Hz, 1H), 7.54–7.49 (m, 3H), 7.43–7.36 (m, 3H), 7.33–7.22 (m, 3H), 5.96 (s, 1H), 0.94 (s, 9H), 0.11 (s, 3H), 0.05 (s, 3H); ^{13}C NMR (100 MHz, $CDCl_3$) δ 164.4, 150.0, 139.9, 131.2, 130.4, 129.8, 128.4, 128.0, 127.0, 126.1, 122.9, 122.8, 122.7, 70.6, 25.7, 18.3, -5.1, -5.2; HRMS (ESI-TOF) *m/z* Calcd for $C_{22}H_{26}NO_2NaBrSi$ $[M+Na]^+$: 466.0808; found: 466.0804.

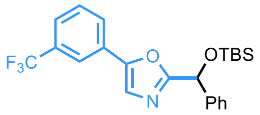

3s

2-(((*tert*-Butyldimethylsilyl)oxy)(phenyl)methyl)-5-(3-(trifluoromethyl)phenyl)oxazole

3s was synthesized following the general procedure under the irradiation of blue LEDs. After purification by preparative thin-layer chromatography using PE/EA (30/1) as the eluent, **3s** was obtained in 57% yield (24.8 mg) as a yellow oil.

^{1}H NMR (400 MHz, $CDCl_3$) δ 7.83 (s, 1H), 7.76 (d, J = 7.6 Hz, 1H), 7.56–7.49 (m, 4H), 7.40–7.29 (m, 4H), 5.98 (s, 1H), 0.95 (s, 9H), 0.11 (s, 3H), 0.06 (s, 3H); ^{13}C NMR (100 MHz, $CDCl_3$) δ 164.6, 150.1, 139.9, 131.4 (q, J = 32 Hz), 129.4, 128.7, 128.5, 128.1, 127.2, 126.1, 124.9 (q, J = 4 Hz), 123.8 (q, J = 271 Hz) 123.0, 120.9 (q, J = 7 Hz), 70.7, 25.7, 18.3, -5.1, -5.2; ^{19}F NMR (375 MHz, $CDCl_3$) δ -63.50; HRMS (ESI-TOF) *m/z* Calcd for $C_{23}H_{26}NO_2NaF_3Si$ $[M+Na]^+$: 456.1577; found: 456.1584.

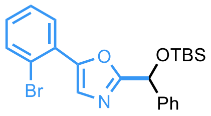

3t

5-(2-Bromophenyl)-2-(((*tert*-butyldimethylsilyl)oxy)(phenyl)methyl)oxazole

3t was synthesized following the general procedure under the irradiation of blue LEDs. After purification by preparative thin-layer chromatography using PE/EA (30/1) as the eluent, **3t** was obtained in 36% yield (16.2 mg) as a white solid.

^{1}H NMR (400 MHz, $CDCl_3$) δ 7.78 (s, 1H), 7.70–7.63 (m, 2H), 7.53 (d, J = 6.8 Hz, 2H), 7.39–7.28 (m, 4H), 7.17–7.13 (m, 1H), 5.98 (s, 1H), 0.94 (s, 9H), 0.10 (s, 3H), 0.06 (s, 3H); ^{13}C NMR (100 MHz, $CDCl_3$) δ 163.9, 149.1, 140.0, 134.0, 129.2, 128.7, 128.5, 128.4, 128.0, 127.5, 126.5, 126.2, 119.9, 70.6, 25.7, 18.3, -5.1; HRMS (ESI-TOF) *m/z* Calcd for $C_{22}H_{26}NO_2NaBrSi$ $[M+Na]^+$: 466.0808; found: 466.0809.

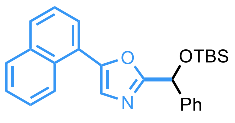

3u

2-(((*tert*-Butyldimethylsilyl)oxy)(phenyl)methyl)-5-(naphthalen-1-yl)oxazole

3u was synthesized following the general procedure under the irradiation of blue LEDs. After purification by preparative thin-layer chromatography using PE/EA (10/1) as the eluent, **3u** was obtained in 61% yield (25.3 mg) as a colorless oil.

^{1}H NMR (400 MHz, $CDCl_3$) δ 8.22–8.19 (m, 1H), 7.88–7.84 (m, 2H), 7.67 (d, *J* = 7.2 Hz, 1H), 7.58 (d, *J* = 7.6 Hz, 2H), 7.52–7.46 (m, 3H), 7.39 (t, *J* = 7.6 Hz, 2H), 7.34–7.29 (m, 2H), 6.03 (s, 1H), 0.96 (s, 9H), 0.12 (s, 3H), 0.10 (s, 3H); ^{13}C NMR (100 MHz, $CDCl_3$) δ 164.2, 150.9, 140.2, 133.8, 130.0, 129.5, 128.6, 128.4, 128.0, 126.9, 126.3, 126.22, 126.15, 125.3, 125.2, 125.1, 125.0, 70.8, 25.7, 18.3, -5.1; HRMS (ESI-TOF) *m/z* Calcd for $C_{26}H_{29}NO_2NaSi$ $[M+Na]^+$: 438.1860; found: 438.1854.

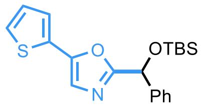

3v

2-(((*tert*-Butyldimethylsilyl)oxy)(phenyl)methyl)-5-(thiophen-2-yl)oxazole

3v was synthesized following the general procedure under the irradiation of blue LEDs. After purification by preparative thin-layer chromatography using PE/EA (10/1) as the eluent, **3v** was obtained in 46% yield (17.0 mg) as a yellow oil.

^{1}H NMR (400 MHz, $CDCl_3$) δ 7.51 (d, *J* = 7.6 Hz, 2H), 7.37 (t, *J* = 7.6 Hz, 2H), 7.32–7.25 (m, 3H), 7.10 (s, 1H), 7.04 (dd, *J* = 4.8, 3.6 Hz, 1H), 5.94 (s, 1H), 0.94 (s, 9H), 0.10 (s, 3H), 0.06 (s, 3H); ^{13}C NMR (100 MHz, $CDCl_3$) δ 163.4, 147.0, 140.0, 129.8, 128.4, 128.0, 127.7, 126.2, 125.5, 124.3, 121.5, 70.6, 25.7, 18.3, -5.1, -5.2; HRMS (ESI-TOF) *m/z* Calcd for $C_{20}H_{25}NO_2SNaSi$ $[M+Na]^+$: 394.1268; found: 394.1261.

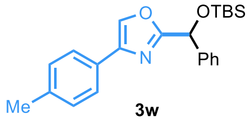

3w

2-(((*tert*-Butyldimethylsilyl)oxy)(phenyl)methyl)-4-(*p*-tolyl)oxazole

3w was synthesized following the general procedure under the irradiation of blue LEDs. After purification by preparative thin-layer chromatography using PE/EA (30/1) as the eluent, **3w** was obtained in 62% yield (23.6 mg) as a white solid.

^{1}H NMR (400 MHz, $CDCl_3$) δ 7.78 (s, 1H), 7.62–7.60 (m, 2H), 7.53–7.51 (m, 2H), 7.37–7.33 (m, 2H), 7.30–7.26 (m, 1H), 7.19 (d, *J* = 7.6 Hz, 2H), 6.02 (s, 1H), 2.36 (s, 3H), 0.93 (s, 9H), 0.11 (s, 3H), 0.03 (s, 3H); ^{13}C NMR (100 MHz, $CDCl_3$) δ 164.3, 140.5, 140.1, 137.8, 133.4, 129.4, 128.3, 128.2, 127.9, 126.0, 125.5, 70.5, 25.7, 21.3, 18.3, -5.17, -5.22; HRMS (ESI-TOF) *m/z* Calcd for $C_{23}H_{29}NO_2NaSi$ $[M+Na]^+$: 402.1860; found: 402.1866.

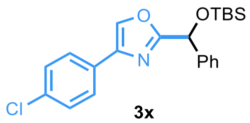

3x

2-(((*tert*-Butyldimethylsilyl)oxy)(phenyl)methyl)-4-(4-chlorophenyl)oxazole

3x was synthesized following the general procedure under the irradiation of blue LEDs. After purification by preparative thin-layer chromatography using PE/EA (30/1) as the eluent, **3x** was obtained in 56% yield (22.2 mg) as a white solid.

^{1}H NMR (400 MHz, $CDCl_3$) δ 7.81 (s, 1H), 7.66–7.63 (m, 2H), 7.52 (d, J = 7.2 Hz, 2H), 7.38–7.34 (m, 4H), 7.31–7.27 (m, 1H), 6.00 (s, 1H), 0.93 (s, 9H), 0.11 (s, 3H), 0.04 (s, 3H); ^{13}C NMR (100 MHz, $CDCl_3$) δ 164.7, 139.9, 139.6, 134.0, 133.7, 129.5, 128.9, 128.4, 128.0, 126.8, 126.0, 70.5, 25.7, 18.3, -5.15, -5.20; HRMS (ESI-TOF) *m/z* Calcd for $C_{22}H_{26}NO_2NaClSi$ $[M+Na]^+$: 422.1314; found: 422.1309.

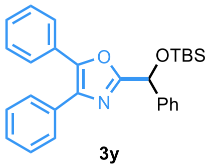

3y

2-(((*tert*-Butyldimethylsilyl)oxy)(phenyl)methyl)-4,5-diphenyloxazole

3y was synthesized following the general procedure under the irradiation of blue LEDs. After purification by preparative thin-layer chromatography using PE/EA (30/1) as the eluent, **3y** was obtained in 64% yield (28.3 mg) as a white solid.

^{1}H NMR (400 MHz, $CDCl_3$) δ 7.65–7.53 (m, 6H), 7.39–7.28 (m, 9H), 6.02 (s, 1H), 0.95 (s, 9H), 0.12 (s, 3H), 0.08 (s, 3H); ^{13}C NMR (100 MHz, $CDCl_3$) δ 162.8, 145.8, 140.2, 135.0, 132.4, 128.9, 128.6, 128.5, 128.5, 128.4, 128.1, 128.0, 127.9, 126.5, 126.2, 70.7, 25.7, 18.3, -5.1; HRMS (ESI-TOF) *m/z* Calcd for $C_{28}H_{31}NO_2NaSi$ $[M+Na]^+$: 464.2016; found: 464.2009.

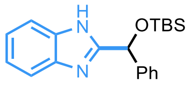

3z

2-(((*tert*-Butyldimethylsilyl)oxy)(phenyl)methyl)-1*H*-benzo[*d*]imidazole

3z was synthesized following the general procedure using ^{t}BuONa (19.2 mg, 0.2 mmol) under the irradiation of blue LEDs. After purification by preparative thin-layer chromatography using PE/EA (10/1) as the eluent, **3z** was obtained in 48% yield (16.3 mg) as a colorless oil.

^{1}H NMR (400 MHz, $CDCl_3$) δ 8.16 (s, 1H), 7.81 (d, J = 7.2 Hz, 1H), 7.40–7.30 (m, 6H), 7.27–7.18 (m, 2H), 6.89 (s, 1H), 0.91 (s, 9H), 0.13 (s, 3H), -0.14 (s, 3H); ^{13}C NMR (100 MHz, $CDCl_3$) δ 141.6, 139.4, 132.1, 128.9, 128.7, 125.6, 123.2, 122.6, 120.2, 111.5, 80.9, 25.5, 18.1, -5.2, -5.3; HRMS (ESI-TOF) *m/z* Calcd for $C_{20}H_{27}N_2OSi$ $[M+H]^+$: 339.1887; found: 339.1883.

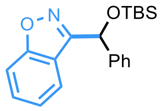

3aa

3-(((*tert*-Butyldimethylsilyl)oxy)(phenyl)methyl)benzo[*d*]isoxazole

3aa was synthesized following the general procedure using ^{t}BuONa (9.6 mg, 0.1 mmol) under the irradiation of blue LEDs. After purification by preparative thin-layer chromatography with Et_3N neutralized using PE/EA (100/1) as the eluent, **3aa** was obtained in 61% yield (20.7 mg) as a white solid.

^{1}H NMR (400 MHz, Acetone-d_6) δ 7.71–7.66 (m, 3H), 7.63–7.59 (m, 1H), 7.47–7.37 (m, 4H), 7.16 (dt, *J* = 7.6, 1.2 Hz, 1H), 6.76 (s, 1H), 0.87 (s, 9H), 0.02 (s, 6H); ^{13}C NMR (100 MHz, Acetone-d_6) δ 158.6, 140.5, 134.8, 134.2, 129.6, 129.0, 127.0, 123.1, 118.5, 116.7, 104.9, 98.4, 25.7, 18.3, -4.6, -4.8; HRMS (ESI-TOF) *m/z* Calcd for $C_{20}H_{25}NO_2NaSi$ $[M+Na]^+$: 362.1547; found: 362.1545.

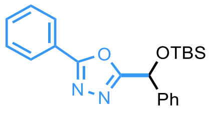

3ab

2-(((*tert*-Butyldimethylsilyl)oxy)(phenyl)methyl)-5-phenyl-1,3,4-oxadiazole

3ab was synthesized following the general procedure under the irradiation of blue LEDs. After purification by preparative thin-layer chromatography using PE/EA (30/1) as the eluent, **3ab** was obtained in 89% yield (32.6 mg) as a white solid.

^{1}H NMR (400 MHz, $CDCl_3$) δ 8.02–8.00 (m, 2H), 7.55 (d, *J* = 6.8 Hz, 2H), 7.50–7.44 (m, 3H), 7.41–7.37 (m, 2H), 7.34–7.30 (m, 1H), 6.16 (s, 1H), 0.94 (s, 9H), 0.12 (s, 3H), 0.07 (s, 3H); ^{13}C NMR (100 MHz, $CDCl_3$) δ 166.7, 165.2, 138.8, 131.7, 128.9, 128.6, 128.4, 126.9, 126.0, 123.7, 68.6, 25.6, 18.2, -5.1, -5.2; HRMS (ESI-TOF) *m/z* Calcd for $C_{21}H_{26}N_2O_2NaSi$ $[M+Na]^+$: 389.1656; found: 389.1654.

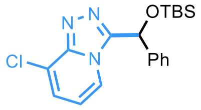

3ac

3-(((*tert*-Butyldimethylsilyl)oxy)(phenyl)methyl)-8-chloro-[1,2,4]triazolo[4,3-*a*]pyridine

3ac was synthesized following the general procedure using ^{t}BuOLi (16.0 mg, 0.2 mmol) under the irradiation of blue LEDs. After purification by preparative thin-layer chromatography using PE/EA (10/1) as the eluent, **3ac** was obtained in 86% yield (32.6 mg) as a white solid.

^{1}H NMR (400 MHz, $CDCl_3$) δ 8.08 (d, *J* = 6.8 Hz, 1H), 7.46 (d, *J* = 7.2 Hz, 2H), 7.36–7.32 (m, 2H), 7.27 (t, *J* = 6.8 Hz, 2H), 6.67 (s, 1H), 6.63 (t, *J* = 6.8 Hz, 1H), 0.91 (s, 9H), 0.19 (s, 3H), -0.12 (s, 3H); ^{13}C NMR (100 MHz, $CDCl_3$) δ 149.1, 148.6, 138.7, 128.6, 127.9, 126.2, 125.0, 122.7, 122.3, 113.0, 69.3, 25.6, 18.2, -5.4, -5.5; HRMS (ESI-TOF) *m/z* Calcd for $C_{19}H_{25}N_3OClSi$ $[M+H]^+$: 374.1450; found: 374.1452.

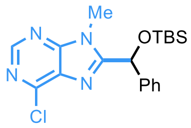

3ad

8-(((*tert*-Butyldimethylsilyl)oxy)(phenyl)methyl)-6-chloro-9-methyl-9*H*-purine

3ad was synthesized following the general procedure using $CuCl_2$ (2.6 mg, 20 mol%), **L14** (5.2 mg, 24 mol%) and ^{t}BuONa (19.2 mg, 0.2 mmol) under the irradiation of blue LEDs. After purification by preparative thin-layer chromatography using PE/EA (10/1) as the eluent, **3ad** was obtained in 32% yield (12.3 mg) as a white solid.

^{1}H NMR (400 MHz, $CDCl_3$) δ 8.70 (s, 1H), 7.48–7.45 (m, 2H), 7.39–7.34 (m, 2H), 7.32–7.28 (m, 1H), 6.40 (s, 1H), 3.69 (s, 3H), 0.97 (s, 9H), 0.20 (s, 3H), -0.02 (s, 3H); ^{13}C NMR (100 MHz, $CDCl_3$) δ 157.6, 153.8, 151.7, 150.1, 138.6, 130.4, 128.7, 128.0, 125.1, 72.2, 30.0, 25.8, 18.2, -5.1, -5.3; HRMS (ESI-TOF) *m/z* Calcd for $C_{19}H_{25}N_4ONaClSi$ $[M+Na]^+$: 411.1378; found: 411.1384.

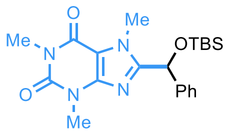

3ae

8-(((*tert*-Butyldimethylsilyl)oxy)(phenyl)methyl)-1,3,7-trimethyl-3,7-dihydro-1*H*-purine-2,6-dione

3ae was synthesized following the general procedure using ^{t}BuONa (19.2 mg, 0.2 mmol) under the irradiation of blue LEDs. After purification by preparative thin-layer chromatography using PE/EA (10/1) as the eluent, **3ae** was obtained in 21% yield (8.9 mg) as a white solid.

^{1}H NMR (400 MHz, $CDCl_3$) δ 7.38–7.33 (m, 4H), 7.31–7.28 (m, 1H), 6.16 (s, 1H), 3.76 (s, 3H), 3.62 (s, 3H), 3.38 (s, 3H), 0.96 (s, 9H), 0.16 (s, 3H), -0.02 (s, 3H); ^{13}C NMR (100 MHz, $CDCl_3$) δ 155.5, 153.2, 151.7, 147.2, 139.4, 128.5, 127.8, 125.0, 108.5, 71.3, 32.6, 29.8, 27.9, 25.7, 18.2, -5.2, -5.4; HRMS (ESI-TOF) *m/z* Calcd for $C_{21}H_{31}N_4O_3Si$ $[M+H]^+$: 415.2160; found: 415.2156.

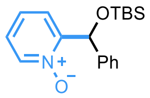

5a

2-(((*tert*-Butyldimethylsilyl)oxy)(phenyl)methyl)pyridine 1-oxide

5a was synthesized following the general procedure using CuBr (10 mol%) and ^{t}BuOLi (16.0 mg, 0.20 mmol) under the irradiation of blue LEDs for 24 hours. After purification by preparative thin-layer chromatography using DCM/MeOH (20/1) as the eluent, **5a** was obtained in 73% yield (23.1 mg) as a white solid.

^{1}H NMR (400 MHz, $CDCl_3$) δ 8.12 (d, J = 6.4 Hz, 1H), 7.75 (d, J = 8.0 Hz, 1H), 7.55 (d, J = 7.6 Hz, 2H), 7.31–7.20 (m, 4H), 7.13 (t, J = 7.6 Hz, 1H), 6.43 (s, 1H), 0.90 (s, 9H), 0.04 (s, 3H), -0.05 (s, 3H); ^{13}C NMR (100 MHz, $CDCl_3$) δ 154.7, 141.1, 139.2, 128.0, 127.7, 127.1, 125.7, 123.9, 123.3, 69.8, 25.7, 18.1, -4.9, -5.1; HRMS (ESI-TOF) *m/z* Calcd for $C_{18}H_{25}NO_2NaSi$ $[M+Na]^+$: 338.1547; found: 338.1541.

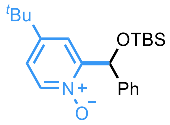

5b

4-(*tert*-Butyl)-2-(((*tert*-butyldimethylsilyl)oxy)(phenyl)methyl)pyridine 1-oxide

5b was synthesized following the general procedure using ^{t}BuOLi (16.0 mg, 0.20 mmol) under the irradiation of blue LEDs for 24 hours. After purification by preparative thin-layer chromatography using DCM/MeOH (20/1) as the

eluent, **5b** was obtained in 46% yield (17.2 mg) as a white solid.

^{1}H NMR (400 MHz, $CDCl_3$) δ 8.05 (d, *J* = 6.8 Hz, 1H), 7.77 (d, *J* = 2.8 Hz, 1H), 7.56 (d, *J* = 6.8 Hz, 2H), 7.29 (t, *J* = 7.2 Hz, 2H), 7.24–7.20 (m, 1H), 7.12 (dd, *J* = 6.8, 3.2 Hz, 1H), 6.43 (s, 1H), 1.32 (s, 9H), 0.92 (s, 9H), 0.03 (s, 3H), -0.04 (s, 3H); ^{13}C NMR (10 MHz, $CDCl_3$) δ 153.4, 150.4, 141.3, 138.4, 128.1, 127.6, 127.0, 121.0, 120.2, 69.9, 34.6, 30.5, 25.7, 18.1, -5.0, -5.2; HRMS (ESI-TOF) *m/z* Calcd for $C_{22}H_{34}NO_2Si$ $[M+H]^+$: 372.2353; found: 372.2347.

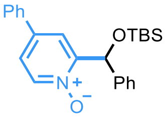

5c

2-(((*tert*-Butyldimethylsilyl)oxy)(phenyl)methyl)-4-phenylpyridine 1-oxide

5c was synthesized following the general procedure using ^{t}BuOLi (24.0 mg, 0.30 mmol) under the irradiation of blue LEDs for 24 hours. After purification by preparative thin-layer chromatography using DCM/MeOH (20/1) as the eluent, **5c** was obtained in 46% yield (18.1 mg) as a white solid.

^{1}H NMR (400 MHz, $CDCl_3$) δ 8.17 (d, *J* = 6.8 Hz, 1H), 8.01 (d, *J* = 2.8 Hz, 1H), 7.62–7.59 (m, 4H), 7.49 (t, *J* = 8.0 Hz, 2H), 7.45–7.41 (m, 1H), 7.37 (dd, *J* = 6.8, 2.8 Hz, 1H), 7.30 (t, *J* = 7.6 Hz, 2H), 7.25–7.21 (m, 1H), 6.48 (s, 1H), 0.94 (s, 9H), 0.08 (s, 3H), -0.01 (s, 3H); ^{13}C NMR (100 MHz, $CDCl_3$) δ 154.5, 141.1, 139.3, 138.5, 136.8, 129.3, 128.9, 128.1, 127.7, 127.1, 126.4, 121.6, 120.8, 69.9, 25.8, 18.2, -4.9, -5.0; HRMS (ESI-TOF) *m/z* Calcd for $C_{24}H_{30}NO_2Si$ $[M+H]^+$: 392.2040; found: 392.2037.

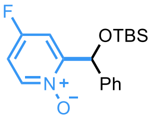

5d

2-(((*tert*-butyldimethylsilyl)oxy)(phenyl)methyl)-4-fluoropyridine 1-oxide

5d was synthesized following the general procedure using $CuCl_2$ (2.6 mg, 20 mol%), **L14** (5.2 mg, 24 mol%) and ^{t}BuOLi (16.0 mg, 0.20 mmol) under the irradiation of blue LEDs for 24 hours. After purification by preparative thin-layer chromatography using DCM/MeOH (20/1) as the eluent, **5d** was obtained in 38% yield (12.7 mg) as a yellow solid.

^{1}H NMR (400 MHz, $CDCl_3$) δ 8.09 (dd, *J* = 7.2, 5.2 Hz, 1H), 7.56–7.50 (m, 3H), 7.32–7.23 (m, 3H), 6.92–6.88 (m, 1H), 6.34 (s, 1H), 0.90 (s, 9H), 0.06 (s, 3H), -0.06 (s, 3H); ^{13}C NMR (100 MHz, $CDCl_3$) δ 159.7 (d, *J* = 257 Hz), 157.0 (d, *J* = 8 Hz), 140.9 (d, *J* = 8 Hz), 140.2, 128.1, 128.0, 127.2, 112.3 (d, *J* = 24 Hz), 110.9 (d, *J* = 25 Hz), 70.15, 25.69, 18.07, -4.90, -5.14. ^{19}F NMR (375 MHz, $CDCl_3$) δ -110.43; HRMS (ESI-TOF) *m/z* Calcd for $C_{18}H_{24}NO_2FNaSi$ $[M+Na]^+$: 356.1453; found: 356.1450.

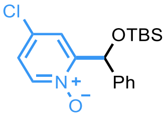

5e

2-(((*tert*-Butyldimethylsilyl)oxy)(phenyl)methyl)-4-chloropyridine 1-oxide

5e was synthesized following the general procedure using **4e** (38.9 mg, 0.30 mmol) and ^{t}BuOLi (16.0 mg, 0.20 mmol) under the irradiation of blue LEDs for 24 hours. After purification by preparative thin-layer chromatography using

DCM/MeOH (20/1) as the eluent, **5e** was obtained in 34% yield (11.8 mg) as a white solid.

^{1}H NMR (400 MHz, CDCl$_3$) δ 8.03 (d, *J* = 7.2 Hz, 1H), 7.72 (d, *J* = 2.8 Hz, 1H), 7.54 (d, *J* = 7.2 Hz, 2H), 7.32–7.23 (m, 3H), 7.11 (dd, *J* = 6.8, 2.8 Hz, 1H), 6.33 (s, 1H), 0.90 (s, 9H), 0.06 (s, 3H), -0.06 (s, 3H); ^{13}C NMR (100 MHz, CDCl$_3$) δ 155.7, 140.3, 140.0, 132.1, 128.2, 128.0, 127.2, 124.3, 123.5, 69.9, 25.7, 18.1, -4.9, -5.1; HRMS (ESI-TOF) *m/z* Calcd for $C_{18}H_{24}NO_2ClNaSi$ [M+Na]$^+$: 372.1157; found: 372.1155.

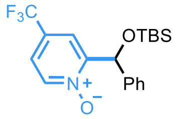

5f

2-(((*tert*-Butyldimethylsilyl)oxy)(phenyl)methyl)-4-(trifluoromethyl)pyridine 1-oxide

5f was synthesized following the general procedure using ^{t}BuOLi (16.0 mg, 0.20 mmol) under the irradiation of blue LEDs for 24 hours. After purification by preparative thin-layer chromatography using PE/EA (2/1) as the eluent, **5e** was obtained in 68% yield (26.0 mg) as a white solid.

^{1}H NMR (400 MHz, CDCl$_3$) δ 8.17 (d, *J* = 6.8 Hz, 1H), 8.01 (d, *J* = 2.8 Hz, 1H), 7.53 (d, *J* = 8.0 Hz, 2H), 7.37–7.24 (M, 4H), 6.30 (s, 1H), 0.90 (s, 9H), 0.05 (s, 3H), -0.06 (s, 3H); ^{13}C NMR (100 MHz, CDCl$_3$) δ 155.6, 139.9, 139.8, 128.2, 128.1, 127.2, 126.7 (q, *J* = 35 Hz), 122.6 (q, *J* = 270 Hz), 120.5 (q, *J* = 4 Hz), 120.2 (q, *J* = 3 Hz), 70.0, 25.6, 18.1, -4.9, -5.2; ^{19}F NMR (375 MHz, CDCl$_3$) δ -64.13; HRMS (ESI-TOF) *m/z* Calcd for $C_{19}H_{24}NO_2F_3NaSi$ [M+Na]$^+$: 406.1421; found: 406.1421.

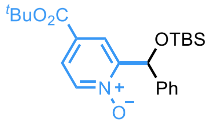

5g

2-(((*tert*-Butyldimethylsilyl)oxy)(phenyl)methyl)-4-(pivaloyloxy)pyridine 1-oxide

5g was synthesized following the general procedure using ^{t}BuOLi (24.0 mg, 0.30 mmol) under the irradiation of blue LEDs for 24 hours. After purification by preparative thin-layer chromatography using DCM/MeOH (20/1) as the eluent, **5g** was obtained in 50% yield (20.7 mg) as a white solid.

^{1}H NMR (400 MHz, CDCl$_3$) δ 8.34 (d, *J* = 2.8 Hz, 1H), 8.11 (d, *J* = 6.8 Hz, 1H), 7.69 (dd, *J* = 6.8, 2.4 Hz, 1H), 7.53 (d, *J* = 6.8 Hz, 2H), 7.32–7.22 (m, 3H), 6.29 (s, 1H), 1.60 (s, 9H), 0.93 (s, 9H), 0.05 (s, 3H), -0.07 (s, 3H); ^{13}C NMR (100 MHz, CDCl$_3$) δ 162.8, 154.5, 140.5, 139.3, 128.5, 128.1, 127.9, 127.3, 123.9, 123.6, 82.4, 70.1, 28.0, 25.7, 18.1, -4.9, -5.2; HRMS (ESI-TOF) *m/z* Calcd for $C_{23}H_{33}NO_4NaSi$ [M+Na]$^+$: 438.2071; found: 438.2075.

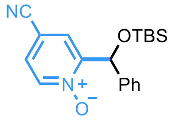

5h

2-(((*tert*-Butyldimethylsilyl)oxy)(phenyl)methyl)-4-cyanopyridine 1-oxide

5h was synthesized following the general procedure using ^{t}BuOLi (16.0 mg, 0.20 mmol) under the irradiation of blue LEDs for 24 hours. After purification by preparative thin-layer chromatography using DCM/MeOH (20/1) as the eluent, **5h** was obtained in 36% yield (12.1 mg) as a white solid.

^{1}H NMR (400 MHz, CDCl$_3$) δ 8.12 (d, *J* = 6.4 Hz, 1H), 8.00 (d, *J* = 2.8 Hz, 1H), 7.50 (d, *J* = 7.6 Hz, 2H), 7.37 (d, *J* = 6.8,

2.4 Hz, 1H), 7.33–7.25 (m, 3H), 6.23 (s, 1H), 0.90 (s, 9H), 0.06 (s, 3H), -0.07 (s, 3H); ^{13}C NMR (100 MHz, $CDCl_3$) δ 156.2, 140.2, 139.6, 128.3, 128.3, 127.3, 126.3, 126.2, 116.4, 107.8, 70.0, 25.7, 18.1, -4.9, -5.1; HRMS (ESI-TOF) *m/z* Calcd for $C_{19}H_{24}N_2O_2NaSi$ $[M+Na]^+$: 363.1499; found: 363.1495.

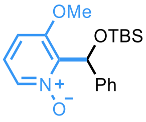

5i

2-(((*tert*-Butyldimethylsilyl)oxy)(phenyl)methyl)-3-methoxypyridine 1-oxide

5i was synthesized following the general procedure using ^{t}BuOLi (24.0 mg, 0.30 mmol) under the irradiation of blue LEDs for 24 hours. After purification by preparative thin-layer chromatography using DCM/MeOH (20/1) as the eluent, **5i** was obtained in 21% yield (7.4 mg) as a white solid.

^{1}H NMR (400 MHz, $CDCl_3$) δ 8.21 (dd, *J* = 4.0, 2.0 Hz, 1H), 7.47 (d, *J* = 7.2 Hz, 2H), 7.27 (t, *J* = 7.2 Hz, 2H), 7.20–7.10 (m, 3H), 6.37 (s, 1H), 3.83 (s, 3H), 0.88 (s, 9H), 0.04 (s, 3H), 0.00, -0.07 (s. 3H); ^{13}C NMR (100 MHz, $CDCl_3$) δ 152.6, 152.1, 143.5, 141.2, 127.8, 126.6, 126.1, 122.8, 117.8, 71.4, 55.3, 25.9, 18.4, -4.9, -5.0; HRMS (ESI-TOF) *m/z* Calcd for $C_{19}H_{27}NO_2NaSi$ $[M+Na-O]^+$: 352.1703; found: 352.1702.

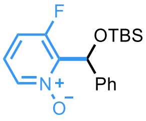

5j

2-(((*tert*-Butyldimethylsilyl)oxy)(phenyl)methyl)-3-fluoropyridine 1-oxide

5j was synthesized following the general procedure using ^{t}BuOLi (16.0 mg, 0.20 mmol) under the irradiation of blue LEDs for 24 hours. After purification by preparative thin-layer chromatography using DCM/MeOH (20/1) as the eluent, **5j** was obtained in 49% yield (16.4 mg) as a white solid.

^{1}H NMR (400 MHz, $CDCl_3$) δ 8.10 (d, *J* = 6.4 Hz, 1H), 7.61 (d, *J* = 7.6 Hz, 2H), 7.32 (t, *J* = 7.6 Hz, 2H), 7.27–7.23 (m, 1H), 7.12–7.06 (m, 1H), 7.00 (s, 1H), 6.94 (d, *J* = 8.4 Hz, 1H), 0.92 (s, 9H), 0.07 (s, 3H), -0.01 (s, 3H); ^{13}C NMR (100 MHz, $CDCl_3$) δ 159.4 (d, *J* = 257 Hz), 145.6 (d, *J* = 21 Hz), 141.0, 135.7, 128.2, 127.6, 125.5 (d, *J* = 2 Hz), 123.4 (d, *J* = 10 Hz), 114.3 (d, *J* = 22 Hz), 67.4 (d, *J* = 2 Hz), 25.6, 18.1, -5.3, -5.4; ^{19}F NMR (375 MHz, $CDCl_3$) δ -116.99; HRMS (ESI-TOF) *m/z* Calcd for $C_{18}H_{24}NO_2FNaSi$ $[M+Na]^+$: 356.1453; found: 356.1448.

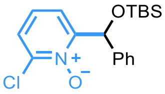

5k

2-(((*tert*-Butyldimethylsilyl)oxy)(phenyl)methyl)-6-chloropyridine 1-oxide

5k was synthesized following the general procedure using **4k** (38.9 mg, 0.30 mmol) and ^{t}BuOLi (16.0 mg, 0.20 mmol) under the irradiation of blue LEDs for 24 hours. After purification by preparative thin-layer chromatography using PE/Acetone (3/1) as the eluent, **5k** was obtained in 29% yield (10.0 mg) as a white solid.

^{1}H NMR (400 MHz, $CDCl_3$) δ 7.73 (d, *J* = 8.0 Hz, 1H), 7.56 (d, *J* = 7.2 Hz, 2H), 7.38 (d, *J* = 8.0 Hz, 1H), 7.31–7.21 (M, 4H), 6.36 (s, 1H), 0.89 (s, 9H), 0.04 (s, 3H), -0.08 (s, 3H); ^{13}C NMR (100 MHz, $CDCl_3$) δ 156.3, 141.8, 140.5, 128.1, 127.8, 127.4, 125.1, 124.9, 121.0, 70.7, 25.7, 18.1, -4.9, -5.1; HRMS (ESI-TOF) *m/z* Calcd for $C_{18}H_{24}NO_2ClNaSi$ $[M+Na]^+$: 372.1157; found: 372.1161.

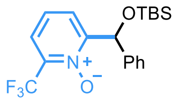

5l

2-(((*tert*-Butyldimethylsilyl)oxy)(phenyl)methyl)-6-(trifluoromethyl)pyridine 1-oxide

5l was synthesized following the general procedure using ^{t}BuOLi (16.0 mg, 0.20 mmol) under the irradiation of blue LEDs for 24 hours. After purification by preparative thin-layer chromatography using PE/EA (2/1) as the eluent, **5l** was obtained in 62% yield (23.7 mg) as a white solid.

^{1}H NMR (400 MHz, $CDCl_3$) δ 7.96 (dd, J = 8.0, 2.0 Hz, 1H), 7.59–7.53 (m, 3H), 7.38–7.22 (m, 4H), 6.35 (s, 1H), 0.89 (s, 9H), 0.05 (s, 3H), -0.08 (s, 3H); ^{13}C NMR (100 MHz, $CDCl_3$) δ 156.7, 140.2, 138.3 (q, J = 33 Hz), 128.2, 127.9, 127.4, 126.0, 124.1, 123.2 (q, J = 4 Hz), 120.0 (q, J = 271 Hz), 69.9, 25.7, 18.1, -4.9, -5.2; ^{19}F NMR (375 MHz, $CDCl_3$) δ -69.22; HRMS (ESI-TOF) *m/z* Calcd for $C_{19}H_{24}NO_2F_3NaSi$ $[M+Na]^+$: 406.1421; found: 406.1417.

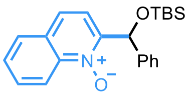

5m

2-(((*tert*-Butyldimethylsilyl)oxy)(phenyl)methyl)quinoline 1-oxide

5m was synthesized following the general procedure using ^{t}BuOLi (16.0 mg, 0.20 mmol) under the irradiation of blue LEDs for 24 hours. After purification by preparative thin-layer chromatography using DCM/MeOH (20/1) as the eluent, **5m** was obtained in 20% yield (7.3 mg) as a white solid.

^{1}H NMR (400 MHz, $CDCl_3$) δ 8.72 (d, J = 8.4 Hz, 2H), 7.86–7.81 (m, 2H), 7.77–7.69 (m, 2H), 7.64 (d, J = 7.2 Hz, 2H), 7.59 (t, J = 7.6 Hz, 1H), 7.30 (t, J = 7.6 Hz, 2H), 7.22 (t, J = 7.6 Hz, 2H), 6.68 (s, 1H), 0.92 (S, 9h), 0.05 (s, 3H), -0.01 (s, 3H); ^{13}C NMR (100 MHz, $CDCl_3$) δ 151.1, 150.3, 141.3, 141.2, 130.2, 129.3, 128.2, 128.0, 127.7, 127.1, 125.6, 119.7, 119.2, 70.4, 25.8, 18.2, -4.9, -5.1; HRMS (ESI-TOF) *m/z* Calcd for $C_{22}H_{27}NO_2NaSi$ $[M+Na]^+$: 388.1703; found: 388.1701.

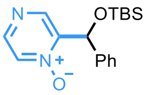

5n

2-(((*tert*-Butyldimethylsilyl)oxy)(phenyl)methyl)pyrazine 1-oxide

5n was synthesized following the general procedure using ^{t}BuOLi (16.0 mg, 0.20 mmol) under the irradiation of blue LEDs for 24 hours. After purification by preparative thin-layer chromatography using PE/EA (2/1) as the eluent, **5n** was obtained in 71% yield (22.4 mg) as a white solid.

^{1}H NMR (400 MHz, $CDCl_3$) δ 8.94 (s, 1H), 8.34 (s, 1H), 7.99 (d, J = 4.0 Hz, 1H), 7.53 (d, J = 7.2 Hz, 2H), 7.34–7.24 (m, 3H), 6.28 (s, 1H), 0.91 (s, 9H), 0.06 (s, 3H), -0.07 (s, 3H); ^{13}C NMR (100 MHz, $CDCl_3$) δ 149.2, 146.0, 145.4, 139.9, 133.4, 128.3, 128.2, 127.2, 69.2, 25.7, 18.1, -4.9, -5.2; HRMS (ESI-TOF) *m/z* Calcd for $C_{17}H_{25}N_2O_2Si$ $[M+H]^+$: 317.1680; found: 317.1673.

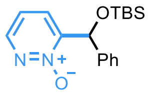

5o

6-(((*tert*-Butyldimethylsilyl)oxy)(phenyl)methyl)pyridazine 1-oxide

5o was synthesized following the general procedure using ^{t}BuOLi (16.0 mg, 0.20 mmol) under the irradiation of blue LEDs for 24 hours. After purification by preparative thin-layer chromatography using PE/EA (2/1) as the eluent, **5o** was obtained in 55% yield (17.3 mg) as a white solid.

^{1}H NMR (400 MHz, $CDCl_3$) δ 8.35 (dd, *J* = 5.2, 2.4 Hz, 1H), 8.07 (dd, *J* = 8.0, 2.4 Hz, 1H), 7.53 (d, *J* = 6.8 Hz, 2H), 7.32–7.23 (m, 3H), 7.11 (dd, *J* = 8.0, 5.2 Hz, 1H), 6.20 (s, 1H), 0.90 (s, 9H), 0.05 (s, 3H), -0.07 (s, 3H); ^{13}C NMR (100 MHz, $CDCl_3$) δ 149.4, 148.7, 139.7, 131.0, 128.2, 128.1, 127.2, 116.4, 70.3, 25.7, 18.1, -4.9, -5.1; HRMS (ESI-TOF) *m/z* Calcd for $C_{17}H_{24}N_2O_2NaSi$ $[M+H]^+$: 339.1499; found: 339.1497.

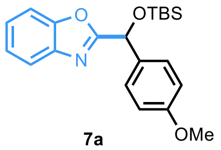

7a

2-(((*tert*-Butyldimethylsilyl)oxy)(4-methoxyphenyl)methyl)benzo[*d*]oxazole

7a was synthesized following the general procedure using $CuCl_2$ (2.6 mg, 20 mol%), **L14** (5.2 mg, 24 mol%) and ^{t}BuONa (9.6 mg, 0.10 mmol) under the irradiation of blue LEDs. After purification by preparative thin-layer chromatography using PE/EA (30/1) as the eluent, **7a** was obtained in 46% yield (17.0 mg) as a white solid.

^{1}H NMR (400 MHz, $CDCl_3$) δ 7.73–7.69 (m, 1H), 7.50–7.47 (m, 3H), 7.32–7.28 (m, 2H), 6.91–6.88 (m, 2H), 6.03 (s, 1H), 3.79 (s, 3H), 0.92 (s, 9H), 0.10 (s, 3H), 0.05 (s, 3H); ^{13}C NMR (100 MHz, $CDCl_3$) δ 166.4, 159.5, 150.8, 140.8, 131.8, 127.5, 125.0, 124.2, 120.3, 113.9, 110.8, 70.9, 55.2, 25.7, 18.3, -5.07, -5.14; HRMS (ESI-TOF) *m/z* Calcd for $C_{21}H_{27}NO_3NaSi$ $[M+Na]^+$: 392.1652; found: 392.1655.

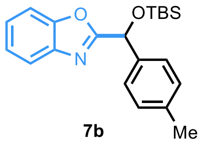

7b

2-(((*tert*-Butyldimethylsilyl)oxy)(*p*-tolyl)methyl)benzo[*d*]oxazole

7b was synthesized following the general procedure under the irradiation of blue LEDs. After purification by preparative thin-layer chromatography using PE/EA (30/1) as the eluent, **7b** was obtained in 62% yield (22.0 mg) as a white solid.

^{1}H NMR (400 MHz, $CDCl_3$) δ 7.72–7.69 (m, 1H), 7.50–7.44 (m, 3H), 7.32–7.27 (m, 2H), 7.17 (d, *J* = 8.0 Hz, 2H), 6.05 (s, 1H), 2.33 (s, 3H), 0.92 (s, 9H), 0.11 (s, 3H), 0.05 (s, 3H); ^{13}C NMR (100 MHz, $CDCl_3$) δ 166.3, 150.8, 140.8, 137.9, 136.7, 129.2, 126.1, 125.0, 124.2, 120.3, 110.9, 71.1, 25.7, 21.2, 18.3, -5.08, -5.14; HRMS (ESI-TOF) *m/z* Calcd for $C_{21}H_{27}NO_2NaSi$ $[M+Na]^+$: 376.1703; found: 376.1699.

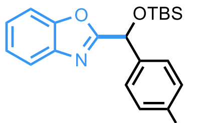

7c

2-((4-(*tert*-Butyl)phenyl)((*tert*-butyldimethylsilyl)oxy)methyl)benzo[*d*]oxazole

7c was synthesized following the general procedure using $CuCl_2$ (2.6 mg, 20 mol%), **L14** (5.2 mg, 24 mol%) under the irradiation of blue LEDs for 24 hours. After purification by preparative thin-layer chromatography using PE/EA (30/1) as the eluent, **7c** was obtained in 47% yield (18.6 mg) as a white solid.

^{1}H NMR (400 MHz, $CDCl_3$) δ 7.74–7.69 (m, 1H), 7.51–7.47 (m, 3H), 7.37 (d, J = 8.4 Hz, 2H), 7.32–7.28 (m, 2H), 6.07 (s, 1H), 1.29 (s, 9H), 0.93 (s, 9H), 0.11 (s, 3H), 0.03 (s, 3H); ^{13}C NMR (100 MHz, $CDCl_3$) δ 166.4, 151.0, 150.8, 140.8, 136.6, 125.7, 125.4, 125.0, 124.2, 120.3, 110.9, 71.0, 34.5, 31.3, 25.7, 18.3, -5.1, -5.2; HRMS (ESI-TOF) *m/z* Calcd for $C_{24}H_{33}NO_2NaSi$ $[M+Na]^+$: 418.2173; found: 418.2173.

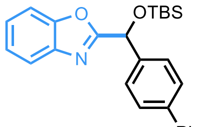

7d

2-([1,1'-Biphenyl]-4-yl((*tert*-butyldimethylsilyl)oxy)methyl)benzo[*d*]oxazole

7d was synthesized following the general procedure under the irradiation of blue LEDs. After purification by preparative thin-layer chromatography using PE/EA (30/1) as the eluent, **7d** was obtained in 80% yield (33.2 mg) as a white solid.

^{1}H NMR (400 MHz, $CDCl_3$) δ 7.75–7.71 (m 1H), 7.65–7.55 (m, 6H), 7.52–7.48 (m, 1H), 7.43–7.39 (m, 2H), 7.34–7.28 (m, 3H), 6.14 (s, 1H), 0.95 (s, 9H), 0.14 (s, 3H), 0.07 (s, 3H); ^{13}C NMR (100 MHz, $CDCl_3$) δ 166.1, 150.9, 141.1, 140.8, 140.7, 138.6, 128.7, 127.3, 127.3, 127.1, 126.6, 125.1, 124.3, 120.3, 110.9, 71.0, 25.7, 18.4, -5.07, -5.12; HRMS (ESI-TOF) *m/z* Calcd for $C_{26}H_{29}NO_2NaSi$ $[M+Na]^+$: 438.1860; found: 438.1865.

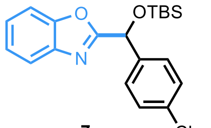

7e

2-(((*tert*-Butyldimethylsilyl)oxy)(4-chlorophenyl)methyl)benzo[*d*]oxazole

7e was synthesized following the general procedure under the irradiation of blue LEDs. After purification by preparative thin-layer chromatography using PE/EA (30/1) as the eluent, **7e** was obtained in 73% yield (27.4 mg) as a white solid.

^{1}H NMR (400 MHz, $CDCl_3$) δ 7.74–7.69 (m, 1H), 7.53–7.48 (m, 3H), 7.36–7.29 (m, 4H), 6.05 (s, 1H), 0.92 (s, 9H), 0.12 (s, 3H), 0.05 (s, 3H); ^{13}C NMR (100 MHz, $CDCl_3$) δ 165.7, 150.8, 140.7, 138.2, 134.0, 128.7, 127.6, 125.3, 124.4, 120.4, 110.9, 70.6, 25.7, 18.3, -5.1, -5.2; HRMS (ESI-TOF) *m/z* Calcd for $C_{20}H_{24}NO_2NaClSi$ $[M+Na]^+$: 396.1157; found: 396.1155.

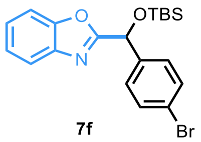

7f

2-((4-Bromophenyl)((*tert*-butyldimethylsilyl)oxy)methyl)benzo[*d*]oxazole

7f was synthesized following the general procedure under the irradiation of blue LEDs. After purification by preparative thin-layer chromatography using PE/EA (30/1) as the eluent, **7f** was obtained in 72% yield (30.0 mg) as a white solid.

^{1}H NMR (400 MHz, $CDCl_3$) δ 7.73–7.69 (m, 1H), 7.51–7.44 (m, 5H), 7.34–7.29 (m, 2H), 6.03 (s, 1H), 0.92 (s, 9H), 0.11 (s, 3H), 0.05 (s, 3H); ^{13}C NMR (100 MHz, $CDCl_3$) δ 165.6, 150.8, 140.7, 138.7, 131.7, 127.9, 125.3, 124.4, 122.2, 120.4, 110.9, 70.6, 25.7, 18.3, -5.1, -5.2; HRMS (ESI-TOF) *m/z* Calcd for $C_{20}H_{24}NO_2NaBrSi$ $[M+Na]^+$: 440.0652; found: 440.0652.

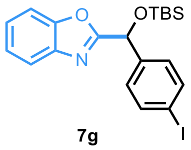

7g

2-(((*tert*-Butyldimethylsilyl)oxy)(4-iodophenyl)methyl)benzo[*d*]oxazole

7g was synthesized following the general procedure using ^{t}BuOLi (4.8 mg, 0.06 mmol) under the irradiation of blue LEDs. After purification by preparative thin-layer chromatography using PE/EA (30/1) as the eluent, **7g** was obtained in 54% yield (25.0 mg) as a white solid.

^{1}H NMR (400 MHz, $CDCl_3$) δ 7.72–7.69 (m, 3H), 7.51–7.47 (m, 1H), 7.33–7.30 (m, 4H), 6.01 (s, 1H), 0.92 (s, 9H), 0.11 (s, 3H), 0.05 (s, 3H); ^{13}C NMR (100 MHz, $CDCl_3$) δ 165.5, 150.8, 140.7, 139.4, 137.6, 128.1, 125.3, 124.4, 120.4, 110.9, 94.0, 70.7, 25.7, 18.3, -5.1, -5.2; HRMS (ESI-TOF) *m/z* Calcd for $C_{20}H_{24}NO_2NaISi$ $[M+Na]^+$: 488.0513; found: 488.0516.

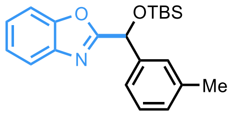

7h

2-(((*tert*-Butyldimethylsilyl)oxy)(*m*-tolyl)methyl)benzo[*d*]oxazole

7h was synthesized following the general procedure under the irradiation of blue LEDs. After purification by preparative thin-layer chromatography using PE/EA (30/1) as the eluent, **7h** was obtained in 66% yield (23.5 mg) as a white solid.

^{1}H NMR (400 MHz, $CDCl_3$) δ 7.74–7.70 (m, 1H), 7.51–7.47 (m, 1H), 7.39–7.36 (m, 2H), 7.32–7.23 (m, 3H), 7.10 (d, *J* = 7.6 Hz, 1H), 6.05 (s, 1H), 2.34 (s, 3H), 0.93 (s, 9H), 0.11 (s, 3H), 0.05 (s, 3H); ^{13}C NMR (100 MHz, $CDCl_3$) δ 166.3, 150.8, 140.8, 139.5, 138.1, 128.9, 128.4, 126.7, 125.0, 124.2, 123.2, 120.3, 110.9, 71.2, 25.7, 21.5, 18.4, -5.1, -5.2; HRMS (ESI-TOF) *m/z* Calcd for $C_{21}H_{27}NO_2NaSi$ $[M+Na]^+$: 376.1703; found: 376.1695.

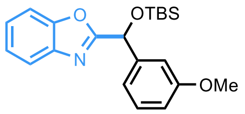

7i

2-(((*tert*-Butyldimethylsilyl)oxy)(3-methoxyphenyl)methyl)benzo[*d*]oxazole

7i was synthesized following the general procedure under the irradiation of blue LEDs. After purification by preparative thin-layer chromatography using PE/EA (30/1) as the eluent, **7i** was obtained in 52% yield (19.4 mg) as a white solid.

^{1}H NMR (400 MHz, $CDCl_3$) δ 7.74–7.70 (m, 1H), 7.51–7.47 (m, 1H), 7.33–7.29 (m, 2H), 7.27–7.25 (m, 1H), 7.16 (t, *J* = 2.0 Hz, 1H), 7.11 (d, *J* = 7.6 Hz, 1H), 6.85–6.82 (m, 1H), 6.07 (m, 1H), 3.80 (s, 3H), 0.93 (s, 9H), 0.13 (s, 3H), 0.05 (s, 3H); ^{13}C NMR (100 MHz, $CDCl_3$) δ 166.0, 159.7, 150.8, 141.2, 140.8, 129.5, 125.1, 124.3, 120.3, 118.4, 113.8, 111.4, 110.9, 71.0, 55.2, 25.7, 18.3, -5.1, -5.2; HRMS (ESI-TOF) *m/z* Calcd for $C_{21}H_{27}NO_3NaSi$ $[M+Na]^+$: 392.1652; found: 392.1653.

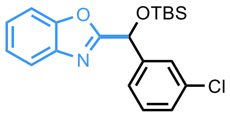

7j

2-(((*tert*-Butyldimethylsilyl)oxy)(3-chlorophenyl)methyl)benzo[*d*]oxazole

7j was synthesized following the general procedure under the irradiation of blue LEDs. After purification by preparative thin-layer chromatography using PE/EA (30/1) as the eluent, **7j** was obtained in 72% yield (27.1 mg) as a white solid.

^{1}H NMR (400 MHz, $CDCl_3$) δ 7.74–7.70 (m, 1H), 7.58 (brs, 1H), 7.53–7.48 (m, 1H), 7.43 (dt, *J* = 6.4, 2.4 Hz, 1H), 7.35–7.26 (m, 4H), 6.05 (s, 1H), 0.93 (s, 9H), 0.13 (s, 3H), 0.05 (s, 3H); ^{13}C NMR (100 MHz, $CDCl_3$) δ 165.5, 150.8, 141.6, 140.7, 134.5, 129.8, 128.4, 126.3, 125.3, 124.4, 124.3, 120.4, 110.9, 70.5, 25.7, 18.3, -5.1, -5.2; HRMS (ESI-TOF) *m/z* Calcd for $C_{20}H_{24}NO_2NaClSi$ $[M+Na]^+$: 396.1157; found: 396.1161.

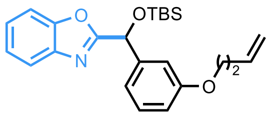

7k

2-((3-(But-3-en-1-yloxy)phenyl)((*tert*-butyldimethylsilyl)oxy)methyl)benzo[*d*]oxazole

7k was synthesized following the general procedure using $CuCl_2$ (2.6 mg, 20 mol%), **L14** (5.2 mg, 24 mol%) under the irradiation of blue LEDs. After purification by preparative thin-layer chromatography using PE/EA (30/1) as the eluent, **7k** was obtained in 74% yield (30.5 mg) as a colorless oil.

^{1}H NMR (400 MHz, $CDCl_3$) δ 7.74–7.69 (m, 1H), 7.51–7.47 (m, 1H), 7.33–7.27 (m, 2H), 7.25 (t, *J* = 7.2 Hz, 1H), 7.15–7.11 (m, 2H), 6.83 (ddd, *J* = 8.4, 2.8, 1.2 Hz, 1H), 6.05 (s, 1H), 5.95–5.84 (m, 1H), 5.15 (dq, *J* = 17.2, 1.6 Hz, 1H), 5.09 (dq, *J* = 10.4, 1.6 Hz, 1H), 4.06–3.97 (m, 2H), 2.53 (qt, *J* = 6.8, 1.2 Hz, 2H), 0.93 (s, 9H), 0.12 (s, 3H), 0.05 (s, 3H); ^{13}C NMR (100 MHz, $CDCl_3$) δ 166.1, 159.0, 150.8, 141.1, 140.8, 134.4, 129.5, 125.1, 124.3, 120.3,

118.4, 117.0, 114.5, 112.1, 110.9, 71.0, 67.1, 33.6, 25.7, 18.3, -5.1, -5.2; HRMS (ESI-TOF) *m/z* Calcd for $C_{24}H_{31}NO_3NaSi$ $[M+Na]^+$: 432.1965; found: 432.1962.

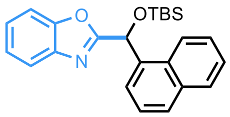

7l

2-(((*tert*-Butyldimethylsilyl)oxy)(naphthalen-1-yl)methyl)benzo[*d*]oxazole

7l was synthesized following the general procedure using ^{t}BuOLi (6.4 mg, 0.08 mmol) under the irradiation of blue LEDs. After purification by preparative thin-layer chromatography using PE/EA (30/1) as the eluent, **7l** was obtained in 41% yield (16.0 mg) as a white solid.

^{1}H NMR (400 MHz, $CDCl_3$) δ 8.26 (d, *J* = 7.6 Hz, 1H), 8.06 (d, *J* = 7.2 Hz, 1H), 7.85–7.83 (m, 2H), 7.72–7.68 (m, 1H), 7.58 (t, *J* = 7.2 Hz, 1H), 7.50–7.41 (m, 3H), 7.29–7.24 (m, 2H), 6.73 (s, 1H), 0.94 (s, 9H), 0.12 (s, 3H), 0.08 (s, 3H); ^{13}C NMR (100 MHz, $CDCl_3$) δ 165.9, 150.7, 140.8, 135.0, 133.6, 130.1, 128.9, 128.8, 126.3, 125.6, 125.5, 125.1, 124.6, 124.2, 123.3, 120.3, 110.9, 69.1, 25.7, 18.4, -5.08, -5.11; HRMS (ESI-TOF) *m/z* Calcd for $C_{24}H_{27}NO_2NaSi$ $[M+Na]^+$: 412.1703; found: 412.1707.

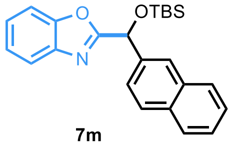

7m

2-(((*tert*-Butyldimethylsilyl)oxy)(naphthalen-2-yl)methyl)benzo[*d*]oxazole

7m was synthesized following the general procedure under the irradiation of blue LEDs. After purification by preparative thin-layer chromatography using PE/EA (30/1) as the eluent, **7m** was obtained in 60% yield (23.3 mg) as a white solid.

^{1}H NMR (400 MHz, $CDCl_3$) δ 8.04 (s, 1H), 7.87–7.80 (m, 3H), 7.74–7.67 (m, 2H), 7.49–7.43 (m, 3H), 7.31–7.27 (m, 2H), 6.25 (s, 1H), 0.95 (s, 9H), 0.14 (s, 3H), 0.09 (s, 3H); ^{13}C NMR (100 MHz, $CDCl_3$) δ 166.1, 150.9, 140.8, 137.1, 133.2, 133.1, 128.4, 128.2, 127.7, 126.19, 126.16, 125.1, 124.3, 124.0, 120.3, 110.9, 71.4, 25.7, 18.4, -5.0, -5.1; HRMS (ESI-TOF) *m/z* Calcd for $C_{24}H_{27}NO_2NaSi$ $[M+Na]^+$: 412.1703; found: 412.1710.

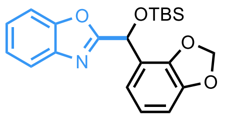

7n

2-(Benzo[*d*][1,3]dioxol-4-yl((*tert*-butyldimethylsilyl)oxy)methyl)benzo[*d*]oxazole

7n was synthesized following the general procedure under the irradiation of blue LEDs. After purification by preparative thin-layer chromatography using PE/EA (30/1) as the eluent, **7n** was obtained in 53% yield (20.3 mg) as a white solid.

^{1}H NMR (400 MHz, $CDCl_3$) δ 7.75–7.71 (m, 1H), 7.54–7.50 (m, 1H), 7.34–7.29 (m, 2H), 7.25 (d, *J* = 8.0 Hz, 1H), 6.89 (t, *J* = 8.0 Hz, 1H), 6.78 (d, *J* = 8.0 Hz, 1H), 6.22 (s, 1H), 5.94 (ABd, *J* = 4.0, 1.2 Hz, 2H), 0.91 (s, 9H), 0.12 (s,

3H), 0.07 (s, 3H); ^{13}C NMR (100 MHz, $CDCl_3$) δ 165.2, 150.8, 147.2, 144.2, 140.9, 125.1, 124.2, 121.8, 121.3, 120.5, 119.8, 110.9, 108.3, 101.2, 65.9, 25.7, 18.3, -5.16, -5.19; HRMS (ESI-TOF) *m/z* Calcd for $C_{21}H_{25}NO_4NaSi$ $[M+Na]^+$: 406.1445; found: 406.1441.

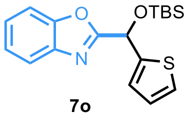

7o

2-(((*tert*-Butyldimethylsilyl)oxy)(thiophen-2-yl)methyl)benzo[*d*]oxazole

7o was synthesized following the general procedure under the irradiation of blue LEDs. After purification by preparative thin-layer chromatography using PE/EA (30/1) as the eluent, **7o** was obtained in 36% yield (12.6 mg) as a yellow oil.

^{1}H NMR (400 MHz, $CDCl_3$) δ 7.76–7.72 (m, 1H), 7.56–7.52 (m, 1H), 7.37–7.31 (m, 2H), 7.29 (dd, *J* = 4.8, 1.2 Hz, 1H), 7.05 (dt, *J* = 3.6, 1.2 Hz, 1H), 6.96 (dd, *J* = 5.2, 3.6 Hz, 1H), 6.33 (s, 1H), 0.93 (s, 9H), 0.14 (s, 3H), 0.07 (s, 3H); ^{13}C NMR (100 MHz, $CDCl_3$) δ 165.2, 150.8, 143.6, 140.7, 126.7, 125.7, 125.3, 124.6, 124.4, 120.4, 111.0, 67.9, 25.6, 18.3, -5.18, -5.21; HRMS (ESI-TOF) *m/z* Calcd for $C_{18}H_{23}NO_2SNaSi$ $[M+Na]^+$: 368.1111; found: 368.1111.

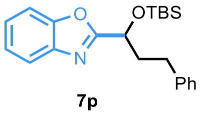

7p

2-(1-((*tert*-butyldimethylsilyl)oxy)-3-phenylpropyl)benzo[*d*]oxazole

7p was synthesized following the general procedure using NaO^{t}Bu (19.2 mg, 0.20 mmol) under the irradiation of blue LEDs for 24 hours. After purification by preparative thin-layer chromatography using PE/EA (30/1) as the eluent, **7p** was obtained in 23% yield (8.5 mg) as a yellow oil.

^{1}H NMR (400 MHz, $CDCl_3$) δ 7.74–7.71 (m, 1H), 7.55–7.53 (m 1H), 7.37–7.31 (m, 2H), 7.29–7.25 (m, 2H), 7.20–7.15 (m, 3H), 5.00 (t, *J* = 7.2 Hz, 1H), 2.85–2.67 (m, 2H), 2.37–2.24 (m 2H), 0.91 (s, 9H), 0.11 (s, 3H), -0.03 (s, 3H); ^{13}C NMR (100 MHz, $CDCl_3$) δ 166.8, 150.6, 141.2, 140.8, 128.4, 126.0, 125.1, 124.3, 120.2, 110.8, 68.5, 38.1, 31.4, 25.7, 18.2, -5.0, -5.2; HRMS (ESI-TOF) *m/z* Calcd for $C_{22}H_{29}NO_2NaSi$ $[M+Na]^+$: 390.1860; found: 390.1860.

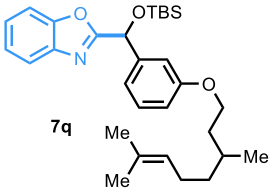

7q

2-(((*tert*-Butyldimethylsilyl)oxy)(3-((3,7-dimethyloct-6-en-1-yl)oxy)phenyl)methyl)benzo[*d*]oxazole

7q was synthesized following the general procedure using $CuCl_2$ (2.6 mg, 20 mol%), **L14** (5.2 mg, 24 mol%) under the irradiation of blue LEDs. The *dr* of **7q** was determined by ^{13}C NMR, after purification by preparative thin-layer chromatography using PE/EA (30/1) as the eluent, **7q** was obtained in 59% yield (29.0 mg) as a white solid.

^{1}H NMR (400 MHz, $CDCl_3$) δ 7.74–7.70 (m, 1H), 7.51–7.47 (m, 1H), 7.33–7.28 (m, 2H), 7.27–7.23 (m, 1H), 7.14–7.09 (m, 2H), 6.82 (dd, *J* = 8.0, 1.6 Hz, 1H), 6.05 (s, 1H), 5.10 (tt, *J* = 7.2, 1.6 Hz, 1H), 4.04–3.93 (m, 2H), 2.08–1.94 (m, 2H), 1.86–1.78 (m, 1H), 1.70–1.64 (m, 4H), 1.60–1.55 (m, 4H), 1.43–1.34 (m, 1H), 1.26–1.16 (m, 1H), 0.95–0.93 (m, 12H), 0.12 (s, 3H), 0.05 (s, 3H); ^{13}C NMR (100 MHz, $CDCl_3$) δ 166.1, 159.3, 150.8, 141.1, 140.8,

131.3, 129.4, 125.1, 124.7, 124.3, 120.3, 118.2, 114.4, 112.0, 110.9, 71.1, 66.2, 37.1, 36.1, 29.5, 25.7, 25.4, 19.5, 18.4, 17.6, -5.1, -5.2; HRMS (ESI-TOF) *m/z* Calcd for $C_{30}H_{43}NO_3NaSi$ $[M+Na]^+$: 516.2904; found: 516.2907.

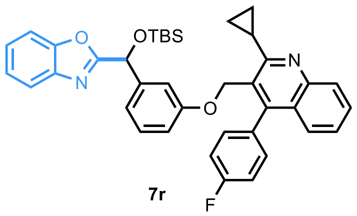

7r

2-(((*tert*-Butyldimethylsilyl)oxy)(3-((2-cyclopropyl-4-(4-fluorophenyl)quinolin-3-yl)methoxy)phenyl)methyl)-benzo[*d*]oxazole

7r was synthesized following the general procedure using $CuCl_2$ (2.6 mg, 20 mol%), **L14** (5.2 mg, 24 mol%) under the irradiation of blue LEDs. After purification by preparative thin-layer chromatography using PE/EA (10/1) as the eluent, **7r** was obtained in 53% yield (33.4 mg) as a white oil.

^1H NMR (400 MHz, $CDCl_3$) δ 8.00 (d, *J* = 8.4 Hz, 1H), 7.73–7.69 (m, 1H), 7.66–7.61 (m, 1H), 7.51–7.47 (m, 1H), 7.38–7.29 (m, 6H), 7.27–7.23 (m, 1H), 7.18 (brs, 1H), 7.15–7.09 (m, 3H), 6.84 (dd, *J* = 8.4 Hz, 2.4 Hz, 1H), 6.04 (s, 1H), 5.02 (ABd, *J* = 15.6, 10.0 Hz, 2H), 2.42–2.35 (m, 1H), 1.38–1.35 (m, 2H), 1.03–0.99 (m, 2H), 0.91 (s, 9H), 0.09 (s, 3H), 0.04 (s, 3H); ^{13}C NMR (100 MHz, $CDCl_3$) δ 165.9, 162.64, 162.55 (d, *J* = 246 Hz), 158.8, 150.8, 147.7, 147.5, 141.3, 140.7, 132.0 (d, *J* = 4 Hz), 131.5 (dd, *J* = 8, 2 Hz), 131.44, 131.42, 129.5, 129.4, 129.0, 126.5, 126.0, 125.7, 125.5, 125.2, 124.3, 120.3, 119.0, 115.4 (dd, *J* = 21, 3 Hz), 114.9, 112.2, 110.9, 70.9, 65.2, 25.7, 18.3, 14.6, 9.73, 9.66, -5.2; ^{19}F NMR (375 MHz, $CDCl_3$) δ -114.03; HRMS (ESI-TOF) *m/z* Calcd for $C_{39}H_{40}N_2O_3FSi$ $[M+H]^+$: 631.2787; found: 631.2779.

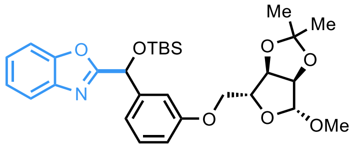

7s

2-(((*tert*-Butyldimethylsilyl)oxy)(3-(((3a*R*,4*R*,6*R*,6a*R*)-6-methoxy-2,2-dimethyltetrahydrofuro[3,4-*d*][1,3]-dioxol-4-yl)methoxy)phenyl)methyl)benzo[*d*]oxazole

7s was synthesized following the general procedure using $CuCl_2$ (2.6 mg, 20 mol%), **L14** (5.2 mg, 24 mol%) under the irradiation of blue LEDs. The *dr* of **7s** was determined by ^{13}C NMR, after purification by preparative thin-layer chromatography using PE/EA (10/1) as the eluent, **7s** was obtained in 61% yield (32.9 mg) as a colorless oil.

^1H NMR (400 MHz, $CDCl_3$) δ 7.73–7.71 (m, 1H), 7.51–7.49 (m, 1H), 7.33–7.29 (m, 2H), 7.25 (d, *J* = 7.6 Hz, 1H), 7.18–7.16 (m, 1H), 7.12 (d, *J* = 7.6 Hz, 1H), 6.85 (dd, *J* = 8.0, 2.4 Hz, 1H), 6.06 (s, 1H), 5.01 (s, 1H), 4.80 (d, *J* = 6.0 Hz, 1H), 4.62 (dd, *J* = 6.0, 2.4 Hz, 1H), 4.55–4.52 (m, 1H), 4.06–3.89 (m, 2H), 3.32 (s, 3H), 1.51 (s, 3H), 1.34 (s, 3H), 0.93 (s, 9H), 0.13 (s, 3H), 0.05 (s, 3H); ^{13}C NMR (100 MHz, $CDCl_3$) δ 165.9, 158.6, 150.8, 141.2, 140.7, 129.5, 125.1, 124.3, 120.3, 118.8, 114.6, 114.5, 112.5, 111.9, 111.8, 110.9, 109.4, 85.10, 85.08, 84.6, 82.10, 82.08, 70.9, 68.2, 54.9, 26.4, 25.7, 24.9, 18.3, -5.1, -5.2; HRMS (ESI-TOF) *m/z* Calcd for $C_{29}H_{39}NO_7NaSi$ $[M+Na]^+$: 564.2388; found: 564.2386.

Unsuccessful Substrates

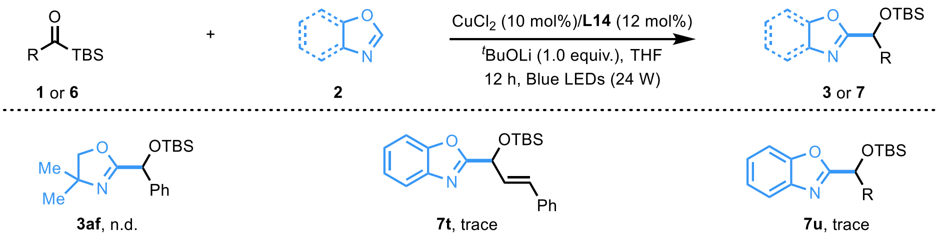

5. Scale-Up Reaction and Synthetic Applications

5.1 Scale-Up Reaction

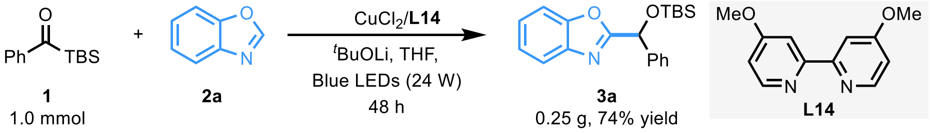

A 100-mL Schlenk flask were charged with $CuCl_2$ (13.4 mg, 0.10 mmol), **L14** (25.9 mg, 0.12 mmol) and ^{t}BuOLi (80.0 mg, 1.0 mmol) under N_2, followed by the addition of THF (3.0 mL). The tube was stirred at room temperature for 4 hours. To the above mixture were added **1** (0.22 g, 1.0 mmol), **2a** (0.24 g, 2.0 mmol) and THF (2.0 mL) subsequently under N_2. The resulted reaction mixture was stirred under the irradiation of blue LEDs (24 W, 450-470 nm) for 48 hours. Upon completion, the reaction mixture was passed through a pad of silica gel with EtOAc as the eluent to remove the copper catalyst and the insoluble precipitate. The resulting solution was evaporated under reduced pressure and the residue was purified by silica gel chromatography using PE/EA (150/1) as the eluent to give **3a** (0.25 g, 74% yield) as a yellow oil.

5.2 Synthetic Applications

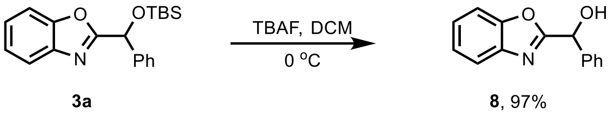

A 50 mL Schlenk flask was charged with **3a** (0.34 g, 1.0 mmol) in dry DCM (10.0 mL). TBAF (3.0 mL, 1.0 mol/L in THF) was added dropwise to the above mixture at 0 °C and stirred at same temperature for 2.0 hours. Upon completion, the reaction was quenched with water and extracted with DCM. The combined organic layers were dried over Na_2SO_4 and evaporated under reduced pressure. The mixture was purified by silica gel chromatography using PE/EA (10/1) as the eluent to give **8**[1] (218.3 mg, 97% yield) as a white solid. ^{1}H NMR (400 MHz, $CDCl_3$) δ 7.74–7.72 (m, 1H), 7.55–7.52 (m, 2H), 7.51–7.47 (m, 1H), 7.43–7.31 (m, 5H), 6.03 (s, 1H), 1.67 (brs, 1H).

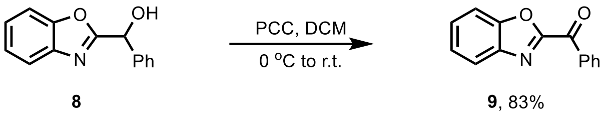

9 was synthesized following the known procedure.[2] A 50 mL Schlenk flask was charged with **8** (113.0 mg, 0.5 mmol) in dry DCM (5.0 mL). PCC (216.0 mg, 1.0 mmol) was added to the above mixture at 0 °C, then the mixture was warmed to room temperature stirred for 2.0 hours. Upon completion, the reaction was filtered through celite and eluted with DCM. The combined organic layers were evaporated under reduced pressure. The mixture was purified by silica gel chromatography using PE/EA (20/1) as the eluent to give **9** (92.4 mg, 83% yield) as a white solid. ^{1}H NMR (400 MHz, $CDCl_3$) δ 8.58–8.55 (m, 2H), 7.96 (d, J = 8.0 Hz, 1H), 7.74–7.68 (m, 2H), 7.60–7.55 (m, 3H), 7.49 (td, J = 8.0, 1.2 Hz, 1H).

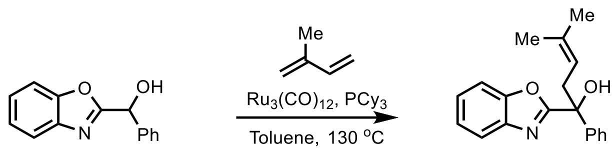

8 **10**, 51%

10 was synthesized following the known procedure.[3] A 4 mL sealed tube were charged with **8** (45.0 mg, 0.2 mmol), isoprene (40.0 μL, 0.4 mmol), $Ru_3(CO)_{12}$ (2.6 mg, 4.0 mol%) and PCy_3 (5.6 mg, 20 mol%) in dry toluene (0.1 mL). The reaction mixture was stirred at 130 °C for 24 hours. Upon completion, the reaction was quenched with water and extracted with EtOAc. The combined organic layers were dried over Na_2SO_4 and evaporated under reduced pressure. The mixture was purified by preparative thin-layer chromatography using PE/EA (20/1) as the eluent to give **10** (30.2 mg, 51% yield) as a white solid. ^{1}H NMR (400 MHz, $CDCl_3$) δ 7.75–7.71 (m, 1H), 7.63 (d, J = 7.6 Hz, 2H), 7.51–7.47 (m, 1H), 7.37–7.25 (m, 5H), 5.14 (t, J = 7.2 Hz, 1H), 3.60 (s, 1H), 3.28 (dd, J = 14.8, 6.8 Hz, 1H), 3.02 (dd, J = 14.8, 7.6 Hz, 1H), 1.66 (s, 3H), 1.64 (s, 3H).

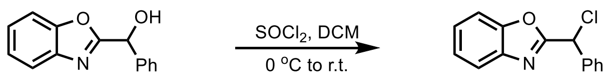

8 **11**, 88%

11 was synthesized following the known procedure.[4] A 10 mL Schlenk flask was charged with **8** (45.0 mg, 0.2 mmol) in dry DCM (1.0 mL). $SOCl_2$ (22 μL, 0.3 mmol) was added dropwise to the above mixture at 0 °C, then warmed to room temperature stirred for 2.0 hours. Upon completion, the reaction was quenched with saturated sodium bicarbonate solution and extracted with DCM. The combined organic layers were dried over Na_2SO_4 and evaporated under reduced pressure. The mixture was purified by silica gel chromatography using PE/EA (30/1) as the eluent to give **11** (42.8 mg, 88% yield) as a colorless oil. ^{1}H NMR (400 MHz, $CDCl_3$) δ 7.76–7.72 (m, 1H), 7.66–7.63 (m, 2H), 7.54–7.49 (m, 1H), 7.43–7.30 (m, 5H), 6.22 (s, 1H).

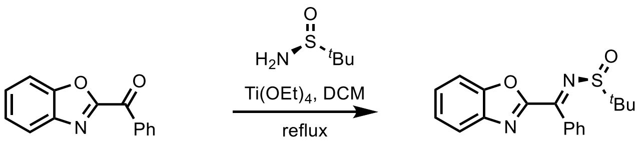

9 **12**, 69%

A 10 mL Schlenk flask were charged with **9** (44.6 mg, 0.2 mmol) and (*S*)-2-methylpropane-2-sulfinamide (29.0 mg, 0.24 mmol) in dry DCM (1.0 mL). Ti(Oet)$_4$ (137.0 mg, 0.6 mmol) was added dropwise to above mixture and the reaction was then refluxed for 24 hours. Upon completion, the reaction was quenched with saturated sodium bicarbonate solution and extracted with DCM. The combined organic layers were dried over Na_2SO_4 and evaporated under reduced pressure. The mixture was purified by silica gel chromatography using PE/EA (20/1) as the eluent to give **12** (45.1 mg, 69% yield) as a yellow solid. ^{1}H NMR (600 MHz, DMSO-d_6, 80 °C) δ 7.86 (dq, J = 7.8, 0.6 Hz, 1H), 7.80 (dq, J = 7.8, 0.6 Hz, 1H), 7.68–7.67 (m, 2H), 7.63–7.60 (m, 1H), 7.54–7.51 (m, 3H), 7.49–7.46 (m, 1H), 1.30 (s, 9H); ^{13}C NMR (150 MHz, DMSO-*d*6, 80 °C) δ 149.7, 140.0, 134.5, 132.3, 128.5, 128.0, 126.4, 124.9, 120.5, 111.1, 58.8, 22.1; HRMS (ESI-TOF) *m/z* Calcd for $C_{18}H_{18}N_2O_2S$na $[M+Na]^+$: 349.0981; found: 349.0982.

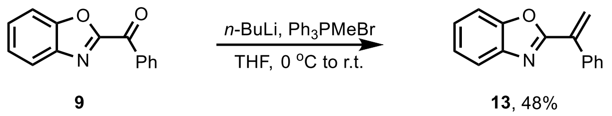

A 10 mL Schlenk flask was charged with Ph_3PMeBr (107.2 mg, 0.3 mmol) in dry THF (1.0 mL). *n*-BuLi (96.0 µL, 2.5 mol/L in hexane) was added dropwise to the above mixture at 0 °C and the mixture was then stirred at same temperature for 1 hour. After that, **9** (44.6 mg, 0.2 mmol) was added to the above mixture at 0 °C, then warmed to room temperature stirred for 1.0 hour. Upon completion, the reaction was quenched with water and extracted with EtOAc. The combined organic layers were dried over Na_2SO_4 and evaporated under reduced pressure. The mixture was purified by silica gel chromatography using PE/EA (40/1) as the eluent to give **13** (21.3 mg, 48% yield) as a yellow oil. ^{1}H NMR (400 MHz, Acetone-*d*$_6$) δ 7.74–7.66 (m, 4H), 7.49–7.38 (m, 5H), 6.51 (d, J = 0.8 Hz, 1H), 6.04 (d, J = 0.8 Hz, 1H).

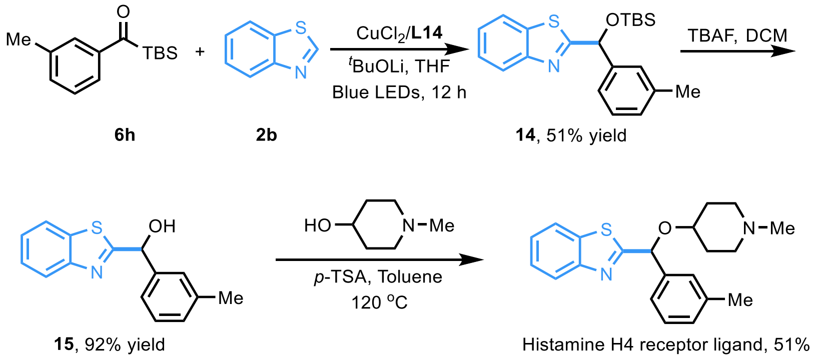

A 10 mL tube were charged with $CuCl_2$ (1.3 mg, 10 mol%), **L14** (2.6 mg, 12 mol%) and ^{t}BuOLi (16.0 mg, 0.2 mmol) under N_2, followed by the addition of THF (1.0 mL). The tube was stirred at room temperature for 2.0 hours. To the above mixture were added **6h** (28.1 mg, 0.12 mmol) and **2b** (13.5 mg, 0.1 mmol) sequentially under N_2. The resulted reaction mixture was stirred under the irradiation of blue LEDs (24 W, 450-470 nm) for 12 hours. Upon completion, the reaction mixture was passed through a pad of silica gel with EtOAc as the eluent to remove the copper catalyst and the insoluble precipitate. The resulted solution was evaporated under reduced pressure, and the residue was purified by preparative thin-layer chromatography using PE/EA (50/1) as the eluent to give **14** (18.8 mg, 51% yield) as a colorless oil. ^{1}H NMR (400 MHz, $CDCl_3$) δ 7.95–7.93 (m, 1H), 7.86–7.83 (m, 1H), 7.43–7.39 (m, 1H), 7.37–7.30 (m, 3H), 7.25–7.20 (m, 1H), 7.07 (d, J = 7.6 Hz, 1H), 6.10 (s, 1H), 2.33 (s, 3H), 0.97 (s, 9H), 0.10 (s, 3H), 0.02 (s, 3H); ^{13}C NMR (100 MHz, $CDCl_3$) δ 177.6, 153.4, 141.6, 138.1, 135.0, 128.8, 128.4, 127.0, 125.7, 124.7, 123.4, 123.0, 121.7, 75.4, 25.7, 21.5, 18.2, -4.9, -5.1; HRMS (ESI-TOF) *m/z* Calcd for $C_{21}H_{28}NOSSi$ $[M+H]^+$: 370.1655; found: 370.1656.

A 10 mL Schlenk flask was charged with **14** (18.8 mg, 0.05 mmol) in dry DCM (1.0 mL). TBAF (0.15 mL, 1.0 mol/L in THF) was added dropwise to the above mixture at 0 °C and stirred at same temperature for 2.0 hours. Upon completion, the reaction was quenched with water and extracted with DCM. The combined organic layers were dried over Na_2SO_4 and evaporated under reduced pressure. The mixture was purified by preparative thin-layer chromatography using PE/EA (10/1) as the eluent to give **15** (11.8 mg, 92% yield) as a white solid. ^{1}H NMR (400 MHz, $CDCl_3$) δ 7.97 (d, J = 8.0 Hz, 1H), 7.83 (d, J = 8.0 Hz, 1H), 7.44 (t, J = 8.0 Hz, 1H), 7.37–7.24 (m, 4H), 7.14 (d, J = 7.6 Hz, 1H), 6.09 (s, 1H), 4.10 (s, 1H), 2.34 (s, 3H); ^{13}C NMR (100 MHz, $CDCl_3$) δ 175.1, 152.6, 140.9, 138.6,

135.2, 129.5, 128.7, 127.4, 126.1, 125.1, 123.8, 123.0, 121.7, 74.4, 21.4; HRMS (ESI-TOF) *m/z* Calcd for $C_{15}H_{14}NOS$ $[M+H]^+$: 256.0791; found: 256.0790.

A 10 mL Schlenk flask were charged with **15** (51.0 mg, 0.2 mmol) and *p*-TSA (190.0 mg, 1.0 mmol) in dry toluene (1.0 mL). 1-methylpiperidin-4-ol (23.0 mg, 0.2 mmol) was added dropwise to the above mixture at 120 °C and the mixture was then stirred at same temperature for 4.0 hours. Upon completion, the reaction was quenched with water and extracted with EtOAc. The combined organic layers were dried over Na_2SO_4, and evaporated under reduced pressure. The mixture was purified by by preparative thin-layer chromatography using DCM/MeOH (10/1) as the eluent to give histamine H4 receptor ligand (35.6 mg, 51% yield) as a yellow oil. ^1H NMR (400 MHz, $CDCl_3$) δ 7.97 (d, *J* = 8.0 Hz, 1H), 7.86 (d, *J* = 8.0 Hz, 1H), 7.44 (t, *J* = 8.0 Hz, 1H), 7.37–7.31 (m, 3H), 7.25 (t, *J* = 7.6 Hz, 1H), 7.11 (d, *J* = 7.6 Hz, 1H), 5.86 (s, 1H), 3.73 (brs, 1H), 2.90–2.82 (m, 2H), 2.58–2.42 (m, 5H), 2.34 (s, 3H), 2.13–2.07 (m, 2H), 1.99–1.84 (m, 2H); ^{13}C NMR (100 MHz, $CDCl_3$) δ 174.4, 153.0, 139.3, 138.3, 135.0, 129.2, 128.5, 127.4, 125.9, 125.0, 123.8, 123.1, 121.7, 78.9, 52.0, 45.3, 29.9, 21.4; HRMS (ESI-TOF) *m/z* Calcd for $C_{21}H_{25}N_2OS$ $[M+H]^+$: 353.1682; found: 353.1687.

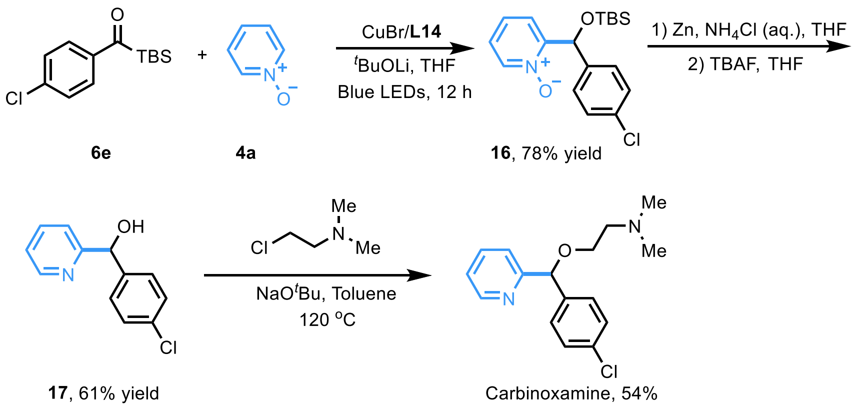

A 10 mL tube were charged with CuBr (1.4 mg, 10 mol%), **L14** (2.6 mg, 12 mol%) and ᵗBuOLi (16.0 mg, 0.2 mmol) under N_2, followed by the addition of THF (1.0 mL). The tube was stirred at room temperature for 2.0 hours. To the above mixture were added **6e** (25.5 mg, 0.10 mmol) and **4a** (19.0 mg, 0.20 mmol) sequentially under N_2. The resulted reaction mixture was stirred under the irradiation of blue LEDs (24 W, 450-470 nm) for 12 hours. Upon completion, the reaction mixture was passed through a pad of silica gel with DCM/MeOH (10/1) as the eluent to remove the copper catalyst and the insoluble precipitate. The resulted solution was evaporated under reduced pressure, and the residue was purified by preparative thin-layer chromatography using DCM/MeOH (20/1) as the eluent to give **16** (27.4 mg, 78% yield) as a white solid. ^1H NMR (400 MHz, $CDCl_3$) δ 8.12 (d, *J* = 6.4 Hz, 1H), 7.74 (dd, *J* = 8.0, 2.0 Hz, 1H), 7.51 (d, *J* = 8.4 Hz, 2H), 7.31 (t, *J* = 8.0 Hz, 1H), 7.26 (d, *J* = 8.4 Hz, 2H), 7.15 (td, *J* = 6.4, 2.0 Hz, 1H), 6.37 (s, 1H), 0.90 (s, 9H), 0.043 (s, 3H), -0.038 (s, 3H); ^{13}C NMR (100 MHz, $CDCl_3$) δ 154.2, 139.6, 139.3, 133.4, 128.5, 128.2, 125.9, 124.1, 123.1, 69.3, 25.7, 18.1, -4.9, -5.1; HRMS (ESI-TOF) *m/z* Calcd for $C_{18}H_{24}NO_2ClNaSi$ $[M+Na]^+$: 372.1157; found: 372.1152.

A 10 mL Schlenk flask was charged with **16** (17.5 mg, 0.05 mmol) in THF (0.5 mL), 30% aqueous solution of NH_4Cl (0.5 mL) and zinc dust (32.7 mg, 0.50 mmol) were added. The reaction mixture was stirred at room temperature for overnight. Upon completion, the reaction was quenched with water and extracted with EtOAc. The combined organic layers were dried over Na_2SO_4 and evaporated under reduced pressure. Then the mixture was dissolved in THF (1.0

mL), TBAF (0.15 mL, 1.0 mol/L in THF) was added dropwise to the above mixture at 0 °C and stirred at same temperature for 30.0 minutes. Upon completion, the reaction was quenched with water and extracted with DCM. The combined organic layers were dried over Na_2SO_4 and evaporated under reduced pressure. The mixture was purified by by preparative thin-layer chromatography using PE/EA (5/1) as the eluent to give **17** (6.7 mg, 61% yield) as a white solid. ^1H NMR (400 MHz, $CDCl_3$) δ 8.57 (d, J = 4.8 Hz, 1H), 7.63 (td, J = 7.6, 1.6 Hz, 1H), 7.34–7.29 (m, 4H), 7.21 (dd, J = 8.0, 4.4 Hz, 1H), 7.12 (d, J = 8.0 Hz, 1H), 5.72 (s, 1H), 5.30 (brs, 1H).

A 10 mL Schlenk flask were charged with **17** (22.0 mg, 0.10 mmol) and 2-chloro-N,N-dimethylethan-1-amine (10.8 mg, 0.10 mmol) in dry toluene (1.0 mL). NaO^{t}Bu (10.6 mg, 0.11 mmol) was added to the above mixture at 120 °C and the mixture was then stirred at same temperature for 5.0 hours. Upon completion, the reaction was quenched with water and extracted with EtOAc. The combined organic layers were dried over Na_2SO_4, and evaporated under reduced pressure. The mixture was purified by by preparative thin-layer chromatography using PE/EA (1/2, TEA: 2%) as the eluent to give Carbinoxamine (15.7 mg, 54% yield) as a yellow oil. ^1H NMR (400 MHz, $CDCl_3$) δ 8.52 (d, J = 4.8 Hz, 1H), 7.68 (t, J = 8.4 Hz, 1H), 7.52 (d, J = 8.0 Hz, 1H), 7.37 (d, J = 8.0 Hz, 2H), 7.28 (d, J = 8.8 Hz, 2H), 7.17–7.14 (m, 1H), 5.47 (s, 1H), 3.66–3.57 (m, 2H), 2.63 (t, J = 6.0 Hz, 2H), 2.29 (s, 6H).

6. Mechanistic Study

6.1 Deuteration Experiments

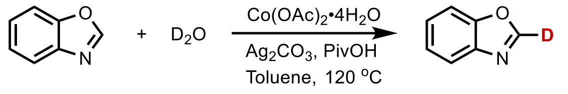

2a **D-2a**, 69%, 91% D

D-2a was synthesized following the known procedure.[5] A 100 mL Schlenk flask were charged with **2a** (1.19 g, 10.0 mmol), D_2O (3.6 mL, 200.0 mmol), Co(Oac)$_2$•4H_2O (0.15 g, 6.0 mol%), Ag_2CO_3 (4.14 g, 15.0 mmol), and PivOH (1.02 g, 10.0 mmol) in dry Toluene (30.0 mL). The reaction mixture was stirred at 120 °C for 3.0 hours. Upon completion, the reaction was filtered through celite and eluted with DCM. The combined organic layers were dried over Na_2SO_4 and evaporated under reduced pressure. The mixture was purified by silica gel chromatography using PE/EA (50/1) as the eluent to give **D-2a** (0.83 g, 69% yield, 91% D) as a colorless oil. ^{1}H NMR (400 MHz, $CDCl_3$) δ 8.11 (s, 0.09H), 7.83–7.78 (m, 1H), 7.62–7.58 (m, 1H), 7.43–7.36 (m, 2H).

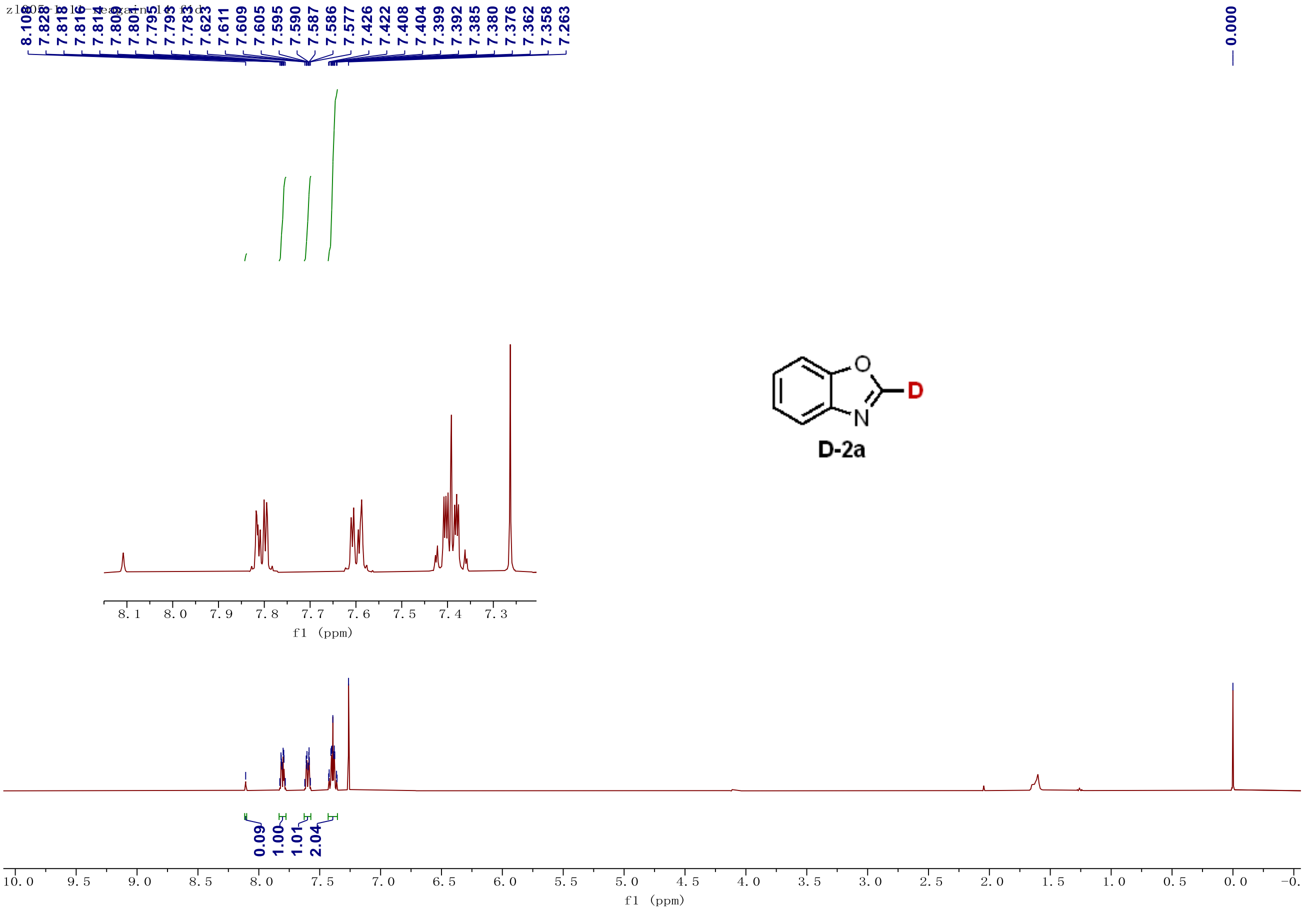

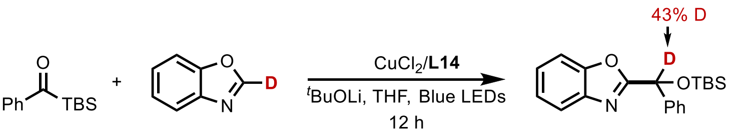

1 **D-2a** (91% D) **D-3a**, 68% yield

A 10 mL tube were charged with $CuCl_2$ (1.3 mg, 10 mol%), **L14** (2.6 mg, 12 mol%) and ^{t}BuOLi (8.0 mg, 0.1 mmol) under N_2, followed by the addition of THF (1.0 mL). The tube was stirred at room temperature for 2.0 hours. To the above mixture were added **1** (22.0 mg, 0.1 mmol) and **D-2a** (24.0 mg, 0.2 mmol) sequentially under N_2. The resulted reaction mixture was stirred under the irradiation of blue LEDs (24 W, 450-470 nm) for 12 hours. Upon completion, the reaction mixture was passed through a pad of silica gel with EtOAc as the eluent to remove the copper catalyst

and the insoluble precipitate. The resulted solution was evaporated under reduced pressure, and the residue was purified by preparative thin-layer chromatography to give **D-3a** (23.1 mg, 68% yield, 43% D) as a colorless oil. ^{1}H NMR (400 MHz, $CDCl_3$) δ 7.74–7.70 (m, 1H), 7.58–7.56 (m, 2H), 7.51–7.47 (m, 1H), 7.39–7.34 (m, 2H), 7.32–7.27 (m, 3H), 6.09 (s, 0.57H), 0.93 (s, 9H), 0.12 (s, 3H), 0.05 (s, 3H).

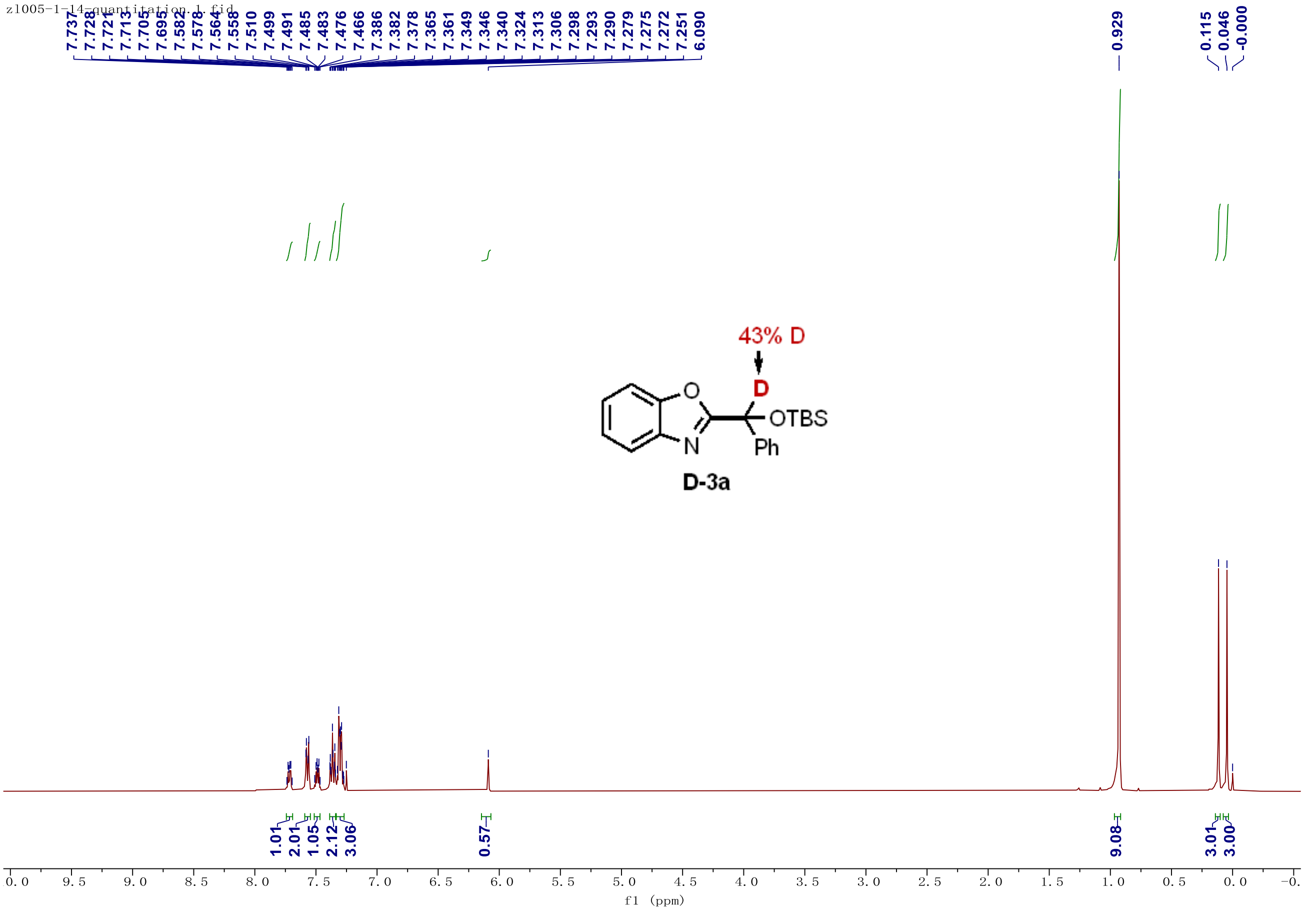

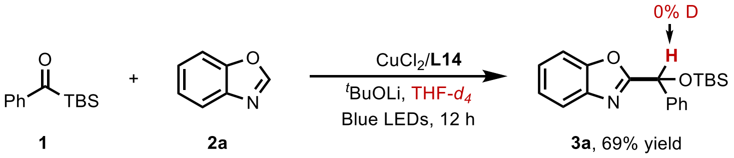

A 10 mL tube were charged with $CuCl_2$ (1.3 mg, 10 mol%), **L14** (2.6 mg, 12 mol%) and ^{t}BuOLi (8.0 mg, 0.1 mmol) under N_2, followed by the addition of THF-d_4 (1.0 mL). The tube was stirred at room temperature for 2.0 hours. To the above mixture were added **1** (22.0 mg, 0.1 mmol) and **2a** (23.8 mg, 0.2 mmol) sequentially under N_2. The resulted reaction mixture was stirred under the irradiation of blue LEDs (24 W, 450-470 nm) for 12 hours. Upon completion, the reaction mixture was passed through a pad of silica gel with EtOAc as the eluent to remove the copper catalyst and the insoluble precipitate. The resulted solution was evaporated under reduced pressure and the residue was purified by preparative thin-layer chromatography to give **3a** (23.5 mg, 69% yield, 0% D).

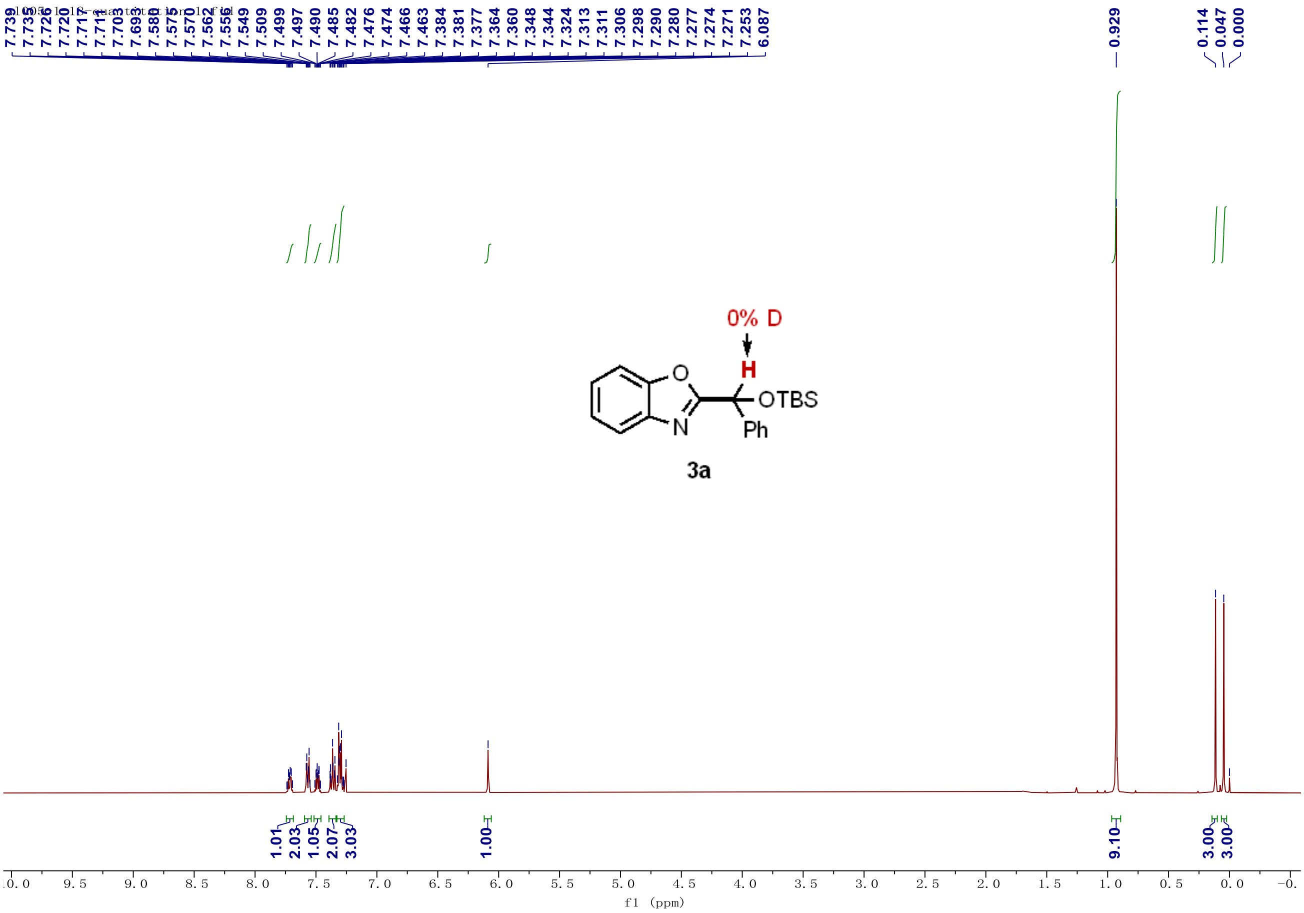

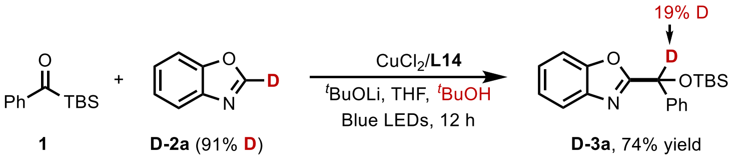

A 10 mL tube were charged with $CuCl_2$ (1.3 mg, 10 mol%), **L14** (2.6 mg, 12 mol%) and ^{t}BuOLi (8.0 mg, 0.1 mmol) under N_2, followed by the addition of THF (1.0 mL). The tube was stirred at room temperature for 2.0 hours. To the above mixture were added **1** (22.0 mg, 0.1 mmol), **D-2a** (24.0 mg, 0.2 mmol) and ^{t}BuOH (7.4 mg, 0.1 mmol) sequentially under N_2. The resulted reaction mixture was stirred under the irradiation of blue LEDs (24 W, 450-470 nm) for 12 hours. Upon completion, the reaction mixture was passed through a pad of silica gel with EtOAc as the eluent to remove the copper catalyst and the insoluble precipitate. The resulted solution was evaporated under reduced pressure, and the residue was purified by preparative thin-layer chromatography to give **D-3a** (25.1 mg, 74% yield, 19% D).

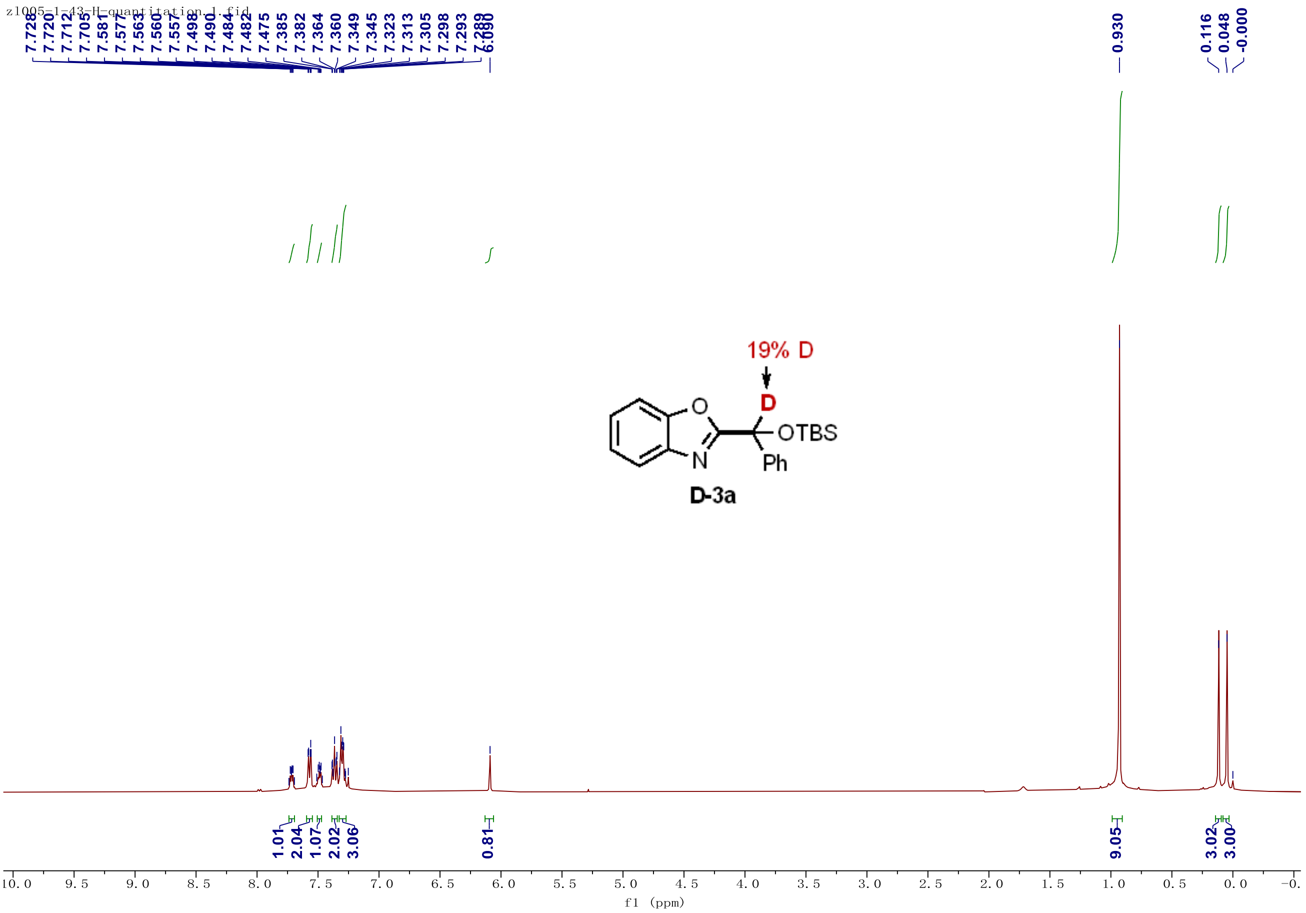

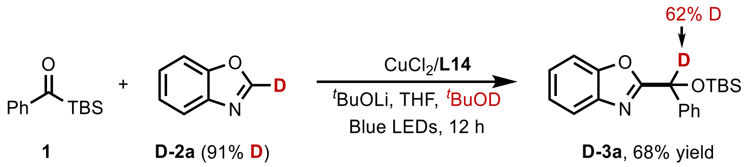

A 10 mL tube were charged with $CuCl_2$ (1.3 mg, 10 mol%), **L14** (2.6 mg, 12 mol%) and ^{t}BuOLi (8.0 mg, 0.1 mmol) under N_2, followed by the addition of THF (1.0 mL). The tube was stirred at room temperature for 2.0 hours. To the above mixture were added **1** (22.0 mg, 0.1 mmol), **D-2a** (24.0 mg, 0.2 mmol) and ^{t}BuOD (7.5 mg, 0.1 mmol) sequentially under N_2. The resulted reaction mixture was stirred under the irradiation of blue LEDs (24 W, 450-470 nm) for 12 hours. Upon completion, the reaction mixture was passed through a pad of silica gel with EtOAc as the eluent to remove the copper catalyst and the insoluble precipitate. The resulted solution was evaporated under reduced pressure, and the residue was purified by preparative thin-layer chromatography to give **D-3a** (23.2 mg, 68% yield, 62% D).

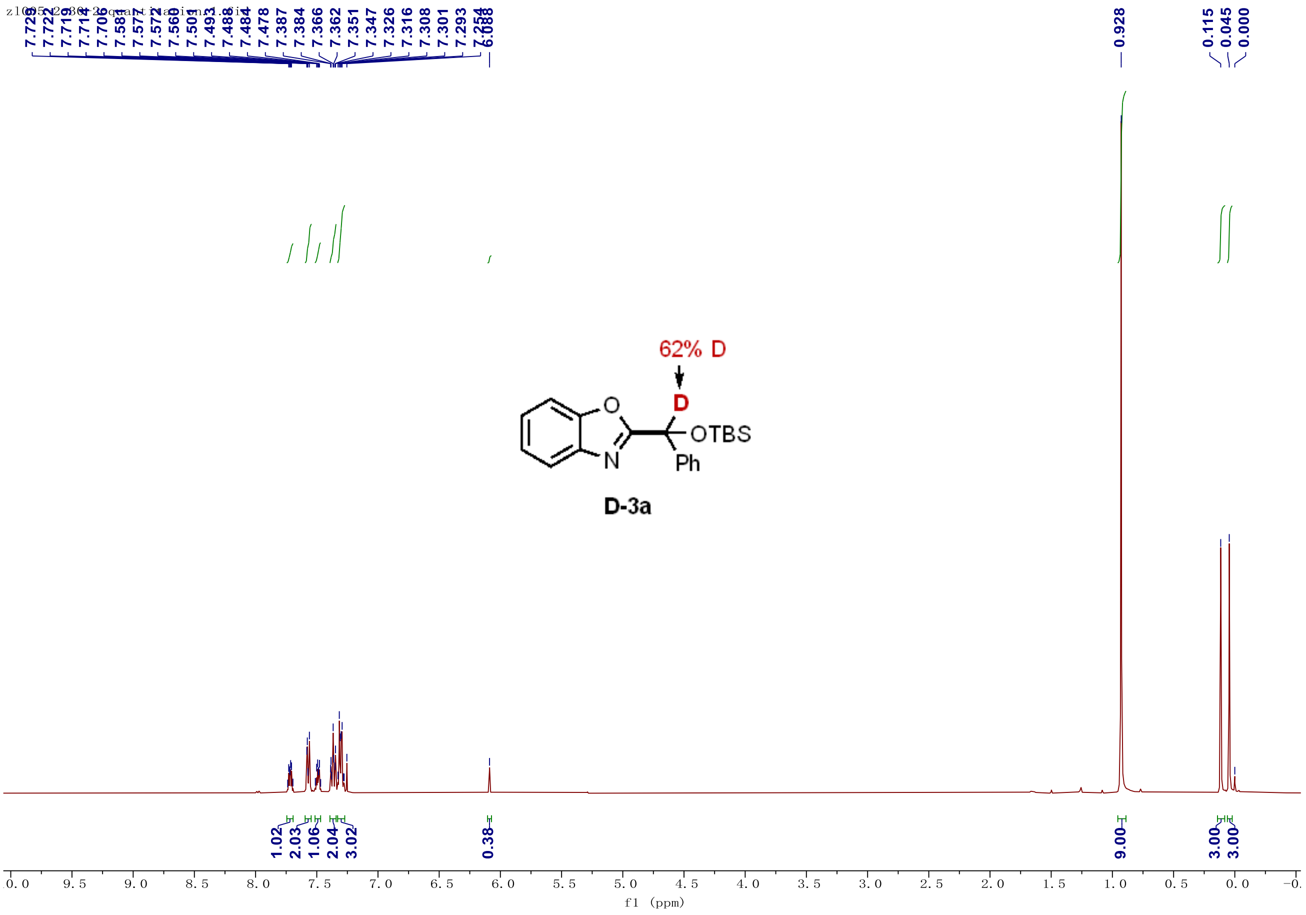

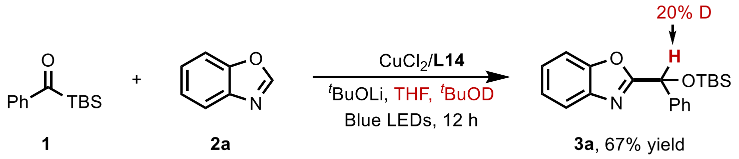

A 10 mL tube were charged with $CuCl_2$ (1.3 mg, 10 mol%), **L14** (2.6 mg, 12 mol%) and ^{t}BuOLi (8.0 mg, 0.1 mmol) under N_2, followed by the addition of THF (1.0 mL). The tube was stirred at room temperature for 2.0 hours. To the above mixture were added **1** (22.0 mg, 0.1 mmol), **2a** (23.8 mg, 0.2 mmol) and ^{t}BuOD (7.5 mg, 0.1 mmol) sequentially under N_2. The resulted reaction mixture was stirred under the irradiation of blue LEDs (24 W, 450-470 nm) for 12 hours. Upon completion, the reaction mixture was passed through a pad of silica gel with EtOAc as the eluent to remove the copper catalyst and the insoluble precipitate. The resulted solution was evaporated under reduced pressure, and the residue was purified by preparative thin-layer chromatography to give D-**3a** (22.9 mg, 67% yield, 20% D).

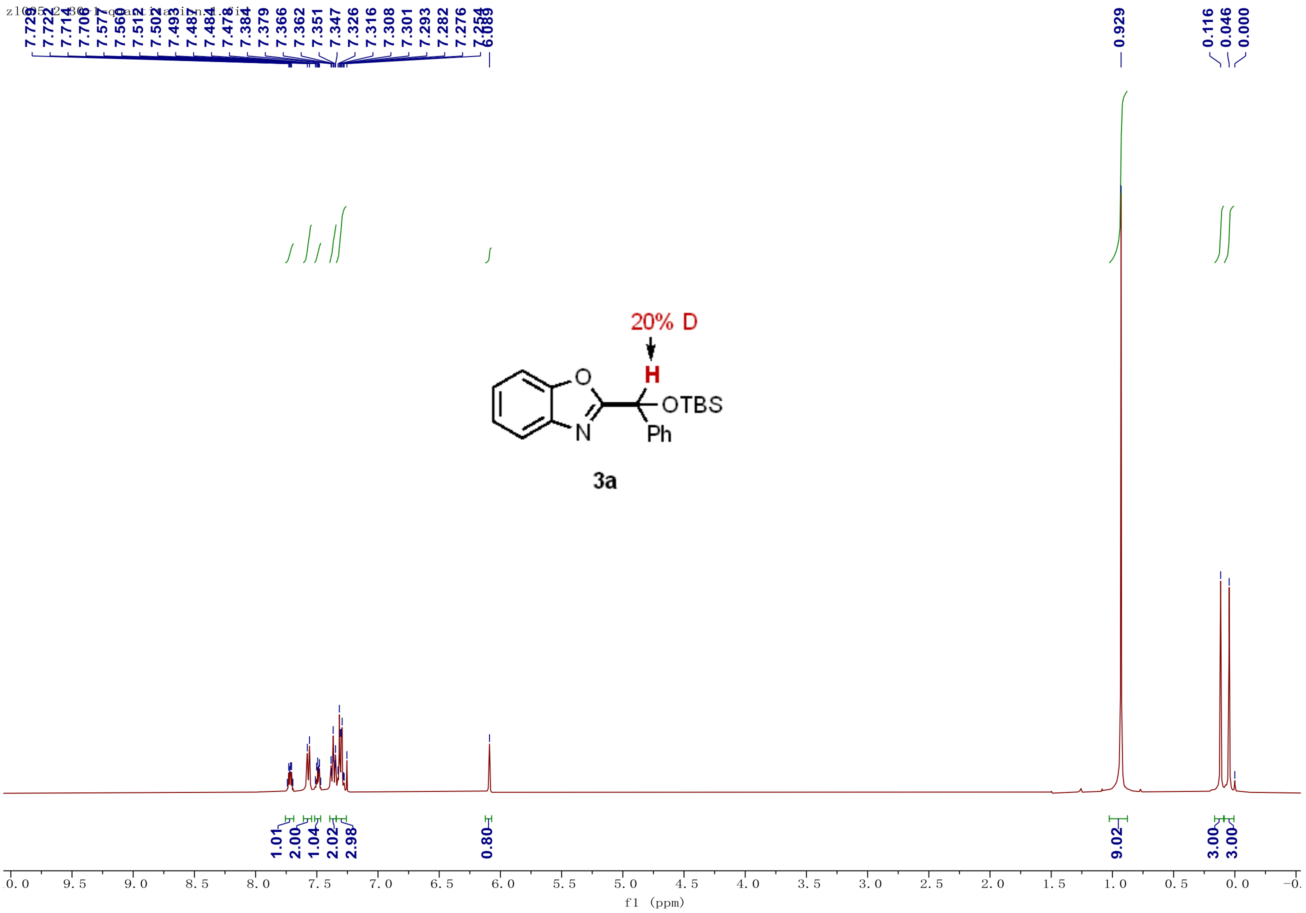

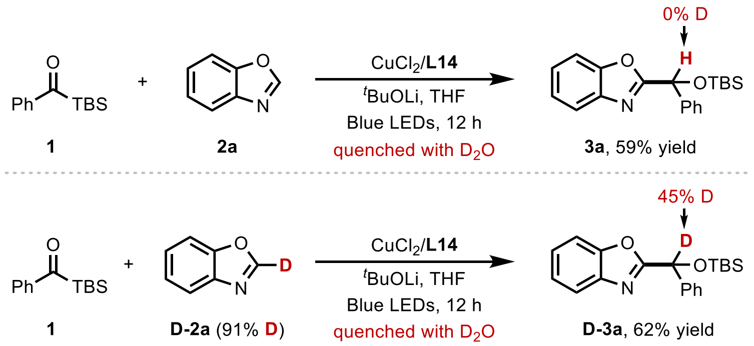

A 10 mL tube were charged with $CuCl_2$ (1.3 mg, 10 mol%), **L14** (2.6 mg, 12 mol%) and ^{t}BuOLi (8.0 mg, 0.1 mmol) under N_2, followed by the addition of THF (1.0 mL). The tube was stirred at room temperature for 2.0 hours. To the above mixture were added **1** (0.1 mmol, 1.0 equiv.) and **2a**/**D-2a** (0.2 mmol, 2.0 equiv.) sequentially under N_2. The resulted reaction mixture was stirred under the irradiation of blue LEDs (24 W, 450-470 nm) for 12 hours. Upon completion, the reaction mixture was quenched with D_2O (1.0 mL), then passed through a pad of silica gel with EtOAc as the eluent to remove the copper catalyst and the insoluble precipitate. The resulted solution was evaporated under reduced pressure, and the yield of product was determined by ^{1}H NMR with CH_2Br_2 as an internal standard.

6.2 KIE Experiments

Two 10 mL tubes were charged with $CuCl_2$ (1.3 mg, 10 mol%), **L14** (2.6 mg, 12 mol%) and ^{t}BuOLi (8.0 mg, 0.1 mmol) under N_2, followed by the addition of THF (1.0 mL). The tube was stirred at room temperature for 2.0 hours. To the above mixture were added **1** (0.1 mmol, 1.0 equiv.) and **2a**/**D-2a** (0.2 mmol, 2.0 equiv.) subsequently under N_2. The resulted reaction mixture was stirred under the irradiation of blue LEDs (24 W, 450-470 nm) for 3 hours. After irradiation, the yield of product was determined by ^{1}H NMR with CH_2Br_2 as an internal standard.

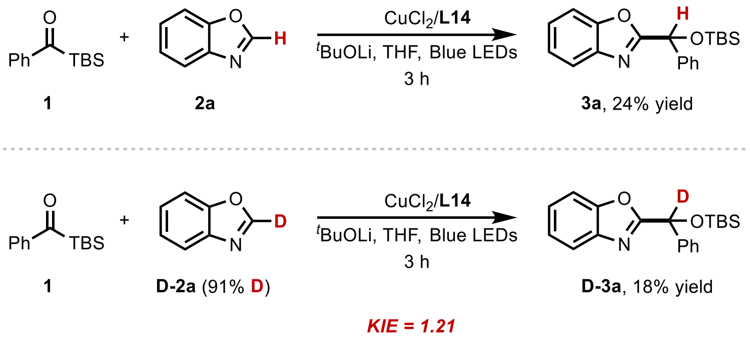

6.3 UV/Vis Absorption Spectroscopy Studies

In order to investigate possible adduct formation between **1** ((*tert*-butyldimethylsilyl)(phenyl)methanone) and **2a** benzo[*d*]oxazole, ^{t}BuOLi and $CuCl_2$/**L14**, UV/vis absorption studies were performed. All absorption spectra were recorded on a Varian Cary 100 two-beam photospectrometer using the following parameter set: data interval 1.0 nm, bandwidth 2.0 nm, response time 0.1 s, scan speed 600 nm/min, baseline correction.

Absorption spectra of all reaction components [(**1** (0.1 mmol), **2a** (0.2 mmol), ^{t}BuOLi (0.1 mmol), and $CuCl_2$/**L14** (10 mol%/12 mol%) in THF (10.0 mL), Figure S2).

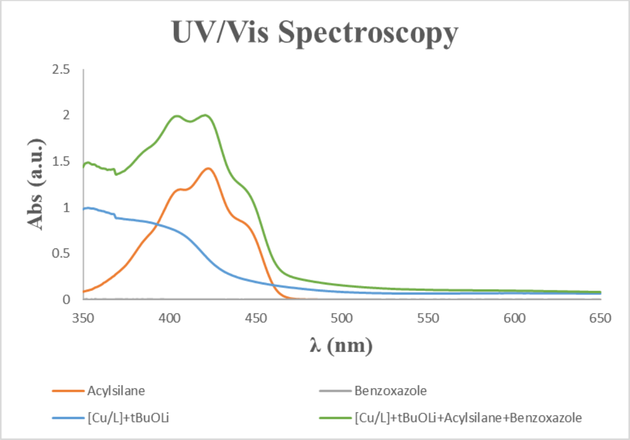

Figure S2 Absorption spectra of all reaction components.

6.4 Determination of Quantum Yields

Determination of the light intensity for 460 nm blue LEDs

The photon flux of the blue LEDs (λ = 460 nm) was determined by standard ferrioxalate actinometry. On one hand, a 50 mL flask was charged with potassium ferrioxalate hydrate (1.8 g) and dissolved in H_2SO_4 (25.0 mL, 0.05 M) to prepare a 0.15 M solution of ferrioxalate. On the other hand, a buffered solution of 1,10-phenanthroline was prepared by dissolving sodium acetate (11.2 g) and phenanthroline (50.0 mg) in H_2SO_4 (50.0 mL, 0.5 M). Both solutions were stored in the dark. To determine the photon flux of the blue LEDs, 4.0 mL of the ferrioxalate solution was placed in a 25 mL Schlenk tube and irradiated for 60 seconds at 460 nm. After irradiation, the irradiated ferrioxalate solution (1.0 mL), the buffered phenanthroline solution (1.0 mL), and water (4.0 mL) were added to the 25 mL flask and the mixture were stirred in the dark for 1 hour to allow the ferrous ions to completely coordinate to the phenanthroline. The absorbance of the solution was measured at 510 nm. A non-irradiated sample was also prepared and the absorbance at 510 nm was measured. Conversion was calculated using eq S1.

$$\text{Mol } Fe^{2+} = V * \Delta A / l * \varepsilon \qquad (S1)$$

$$= 0.6 \text{ L} * 4 * 2.97 / 1.0 \text{ cm} * 11100 \text{ L mol}^{-1}\text{cm}^{-1} = 6.42\times10^{-6} \text{ mol}$$

V = final volume of the solution; ΔA = difference in absorbance between the irradiated and non-irradiated solutions at 510 nm; l = path length (1.0 cm); ε = molar absorptivity of ferrioxalate at 510 nm (11100 L $mol^{-1}cm^{-1}$).

The photon flux can be calculated according to eq S2.

$$\text{Photon flux} = \text{mol } Fe^{2+} / \Phi * t * f \qquad (S2)$$

$$= 6.42\times10^{-6} \text{ mol} / 0.89 * 60 \text{ s} * 1.0 = 1.2 \text{ x } 10^{-7} \text{ einsteins s}^{-1}$$

Φ = quantum yield of ferrioxalate actinometer (0.89 at 460 nm); t = time; f = fraction of light absorbed.

Determination of Quantum Yield for reaction system

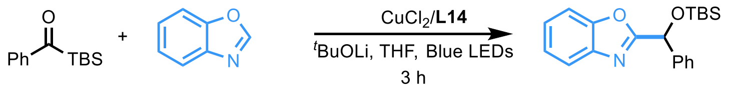

A 10 mL tube were charged with $CuCl_2$ (1.3 mg, 10 mol%), **L14** (2.6 mg, 12 mol%) and ^{t}BuOLi (8.0 mg, 0.1 mmol) under N_2, followed by the addition of THF (1.0 mL). The tube was stirred at room temperature for 2.0 hours. To the above mixture were added **1** (22.0 mg, 0.1 mmol) and **2a** (23.8 mg, 0.2 mmol) sequentially under N_2. The resulted reaction mixture was stirred under the irradiation of blue LEDs (λ = 460 nm, slit width = 3.0 mm, slit height 5.0 mm with intensity of 0.98 mW•cm^{-2}) for 10800 s. After irradiation, the yield of product was determined by ^{1}H NMR analysis to be 24% with CH_2Br_2 as an internal standard. The quantum yield was determined as follows.

$$\Phi = \text{moles of product formed / Photon flux} * t * f$$

$$= 0.0001 \text{ mol} * 0.24 / 1.2 \text{ x } 10^{-7} \text{ einsteins s}^{-1} * 10800 \text{ s} * 0.99 = 0.019$$

This result reveals that the radical chain process is not the main pathway.

6.5 Light On-Off Experiment

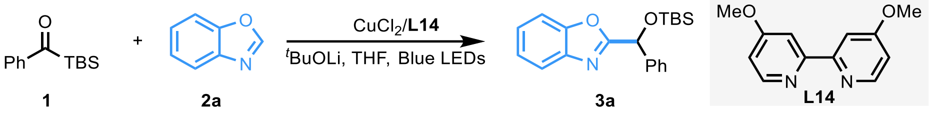

A 10 mL tube were charged with $CuCl_2$ (1.3 mg, 10 mol%), **L14** (2.6 mg, 12 mol%) and ^{t}BuOLi (8.0 mg, 0.1 mmol) under N_2, followed by the addition of THF (1.0 mL). The tube was stirred at room temperature for 2.0 hours. To the above mixture were added **1** (22.0 mg, 0.10 mmol) and **2a** (23.8 mg, 0.20 mmol) subsequently under N_2. The resulted reaction mixture was stirred under the irradiation of blue LEDs (24 W, 450-470 nm). The reaction progress was monitored by removing aliquots (~10.0 μL) from the reaction mixture via syringe under N_2, then filtered with a filter head into 2.0 mL GC vial and analyzed by gas chromatography with dodecane as the internal standard.

Data of light on-off experiment

Entry	**Conditions**	Yield of **3a** (%)
1	Light On 1.0 h	3
2	1.0 + Light Off 0.5 h	3
3	1.5 + Light On 0.5 h	6
4	2.0 + Light Off 0.5 h	6
5	2.5 + Light On 0.5 h	12
6	3.0 + Light Off 0.5 h	12
7	3.5 + Light On 0.5 h	17
8	4.0 + Light Off 0.5 h	18
9	4.5 + Light On 0.5 h	26

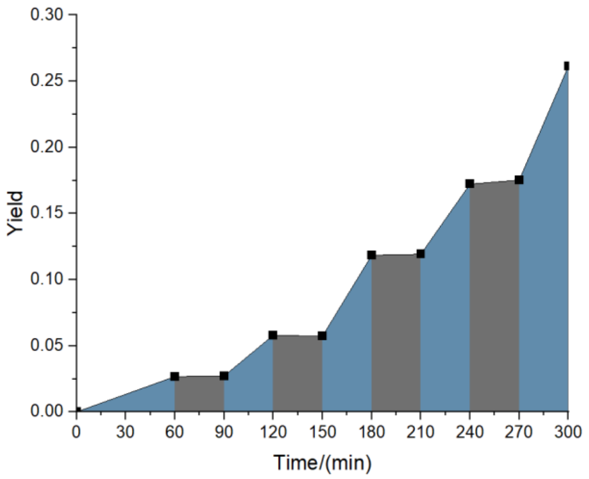

Figure S3 Light on-off experiment.

6.6 In-Situ NMR Studies

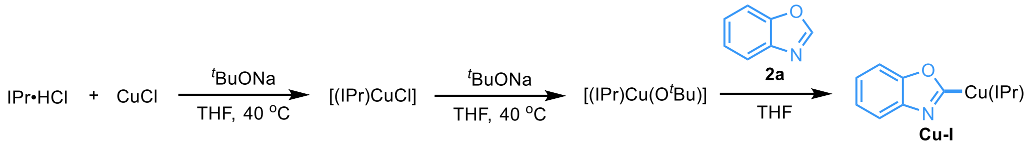

In the glovebox, a 25.0 mL tube were charged with IPr•HCl (425.0 mg, 1.0 mmol) and sodium *tert*-butoxide (96.1 mg, 1.0 mmol), followed by the addition of THF (5.0 mL). The tube was stirred at 40 °C for 4.0 hours. Upon completion, the reaction mixture was filtered through Celite, and concentrated in vacuo, affording [(IPr)CuCl] (285.4 mg, 59% yield) as a colorless powder.

A 25.0 mL tube were charged with [(IPr)CuCl] (195.0 mg, 0.4 mmol) and sodium *tert*-butoxide (38.4 mg, 0.4 mmol), followed by the addition of THF (3.0 mL). The tube was stirred at 40 °C for 4.0 hours. Upon completion, the reaction mixture was filtered through Celite, and concentrated in vacuo, affording [(IPr)Cu(O^{t}Bu)] as a colorless powder. Then, the observed product and benzoxazole (47.6 mg, 0.4 mmol) were dissolved in THF (5.0 mL), stirred at room temperature for 1.0 hour. Upon completion, the reaction mixture was concentrated in vacuo. The resulting solid was washed with hexane to give **Cu-I** (185.1mg, 81% yield) as a purple powder[6].

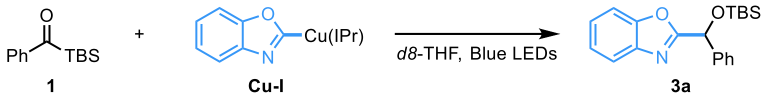

A Schlenk NMR tube was charged with **Cu-I** (11.4 mg, 0.02 mmol) and **1** (4.4 mg, 0.02 mmol) under N_2, followed by the addition of *d8*-THF (0.3 mL). The tube was swinged at room temperature under the irradiation of blue LEDs (24 W, 450-470 nm). The reaction mixture was determined by ^{1}H NMR with toluene (1.1 µL, 0.01 mmol) as an internal standard.

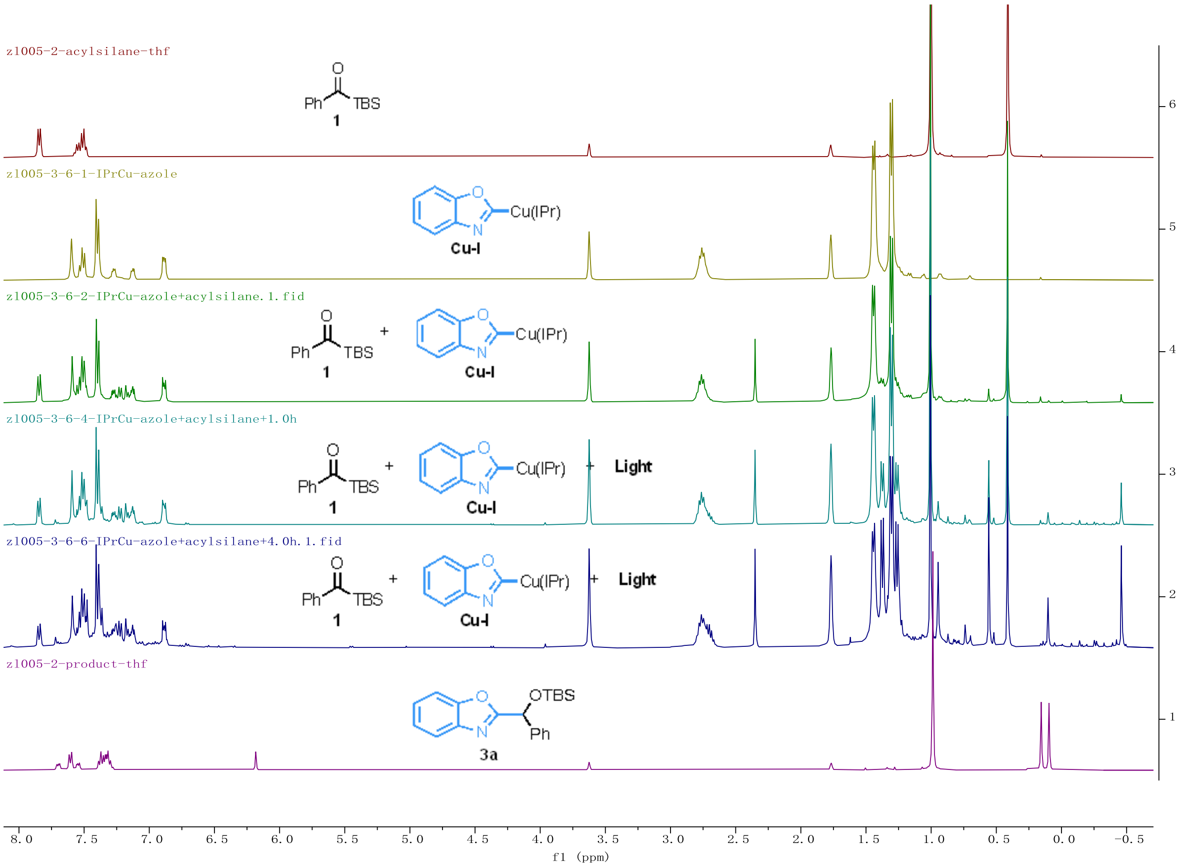

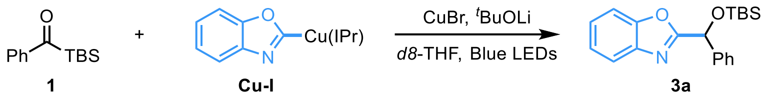

A Schlenk NMR tube was charged with CuBr (2.9 mg, 0.02 mmol) and ^{t}BuOLi (0.8 mg, 0.01 mmol) under N_2, followed by the addition of *d8*-THF (0.3 mL). The tube was swinged at room temperature for for 2.0 hours. To the above mixture were added **Cu-I** (11.4 mg, 0.02 mmol) and **1** (4.4 mg, 0.02 mmol) sequentially under N_2. The tube was swinged at room temperature under the irradiation of blue LEDs (24 W, 450-470 nm) for 1 hour. The yield of product **3a** was determined by ^{1}H NMR analysis to be 10% with toluene (1.1 μL, 0.01 mmol) as an internal standard.

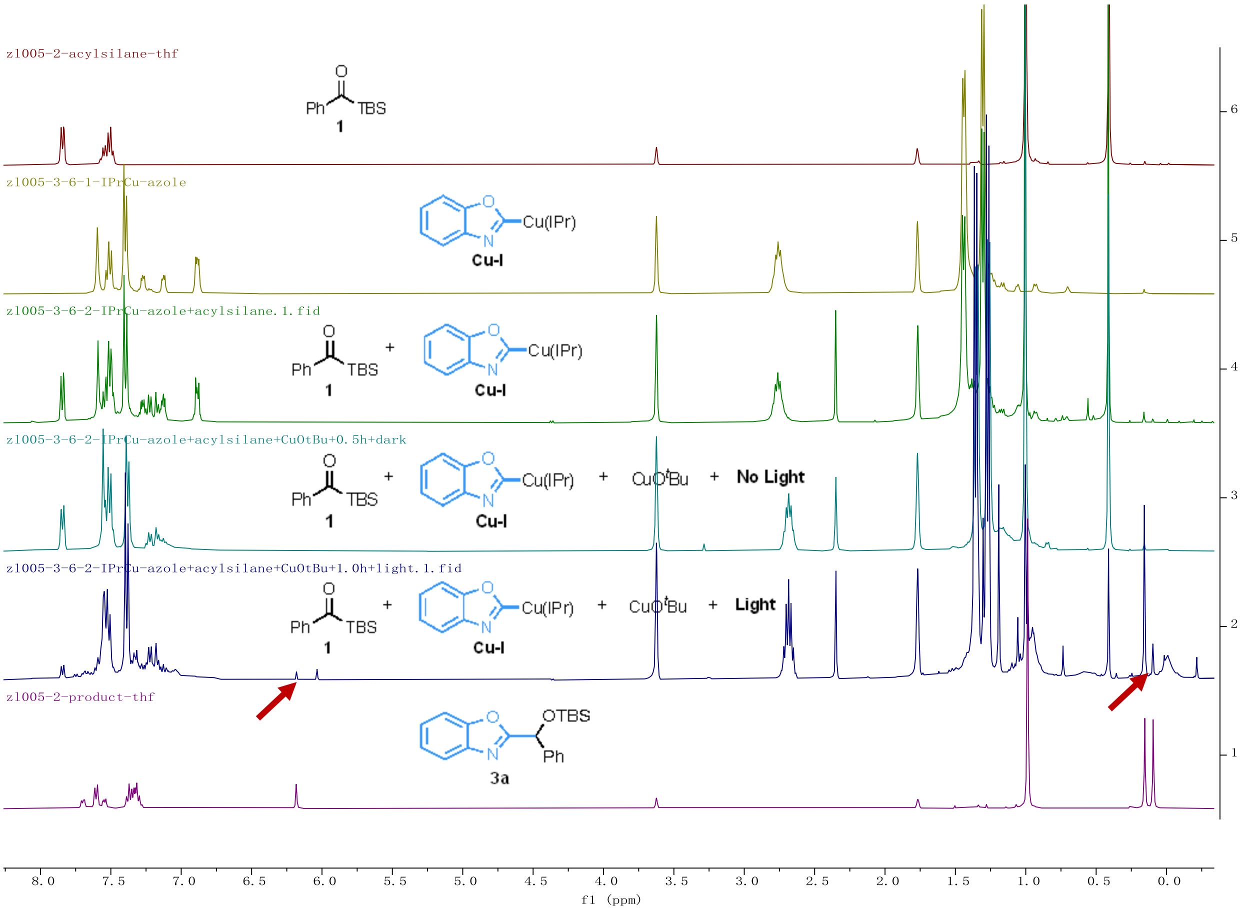

The new appeared peaks belong to product **3a**.

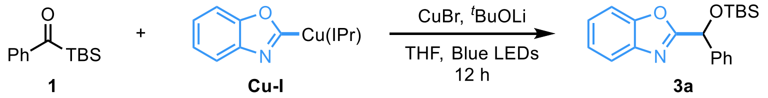

A 10-mL tube were charged with CuBr (7.2 mg, 0.050 mmol) and ^{t}BuOLi (2.0 mg, 0.025 mmol) under N_2, followed by the addition of THF (1.0 mL). The tube was stirred at room temperature for 2.0 hours. To the above mixture were added **Cu-I** (28.5 mg, 0.050 mmol) and **1** (5.5 mg, 0.025 mmol) sequentially under N_2. The resulted reaction mixture was stirred under the irradiation of blue LEDs (24 W, 450-470 nm) for 12 hours. Upon completion, the reaction mixture was passed through a pad of silica gel with EtOAc as the eluent to remove the copper catalyst and the insoluble precipitate. The yield of product was determined by ^{1}H NMR analysis to be 44% with CH_2Br_2 (3.5 μL, 0.050 mmol) as an internal standard. Then the residue was purified by preparative thin-layer chromatography to give **3a** (3.7 mg, 44% yield).

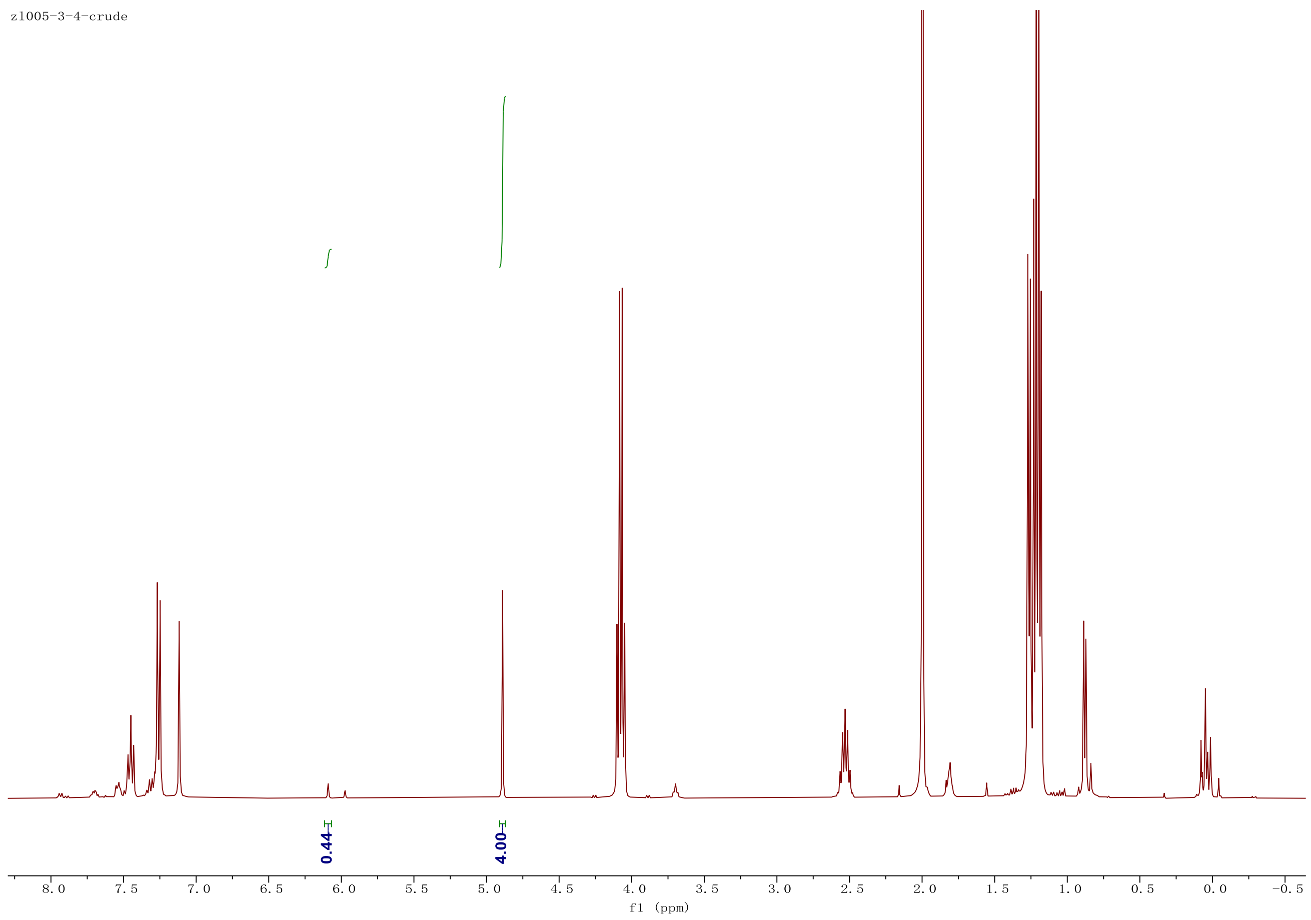
z1005-3-4-crude
0.44
4.00
8.0
7.5
7.0
6.5
6.0
5.5
5.0
4.5
4.0
3.5
3.0
2.5
2.0
1.5
1.0
0.5
0.0
-0.5
f1 (ppm)

6.7 Kinetics Data

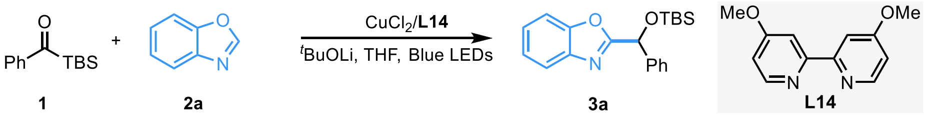

A 10 mL tube were charged with $CuCl_2$, **L14** and ^{t}BuOLi under N_2, followed by the addition of THF (1.0 mL). The tube was stirred at room temperature for 2.0 hours. To the above mixture were added **1** and **2a** subsequently under N_2. The resulted reaction mixture was stirred under the irradiation of blue LEDs (24 W, 450-470 nm). The reaction progress was monitored by removing aliquots (~10.0 μL) from the reaction mixture via syringe under N_2, then filtered with a filter head into 2.0 mL GC vial and analyzed by gas chromatography with dodecane as the internal standard.

Kinetic Plots with Different Concentration of [Cu/L14]

The study of kinetic orders in the catalyst was completed following the general procedure described above.

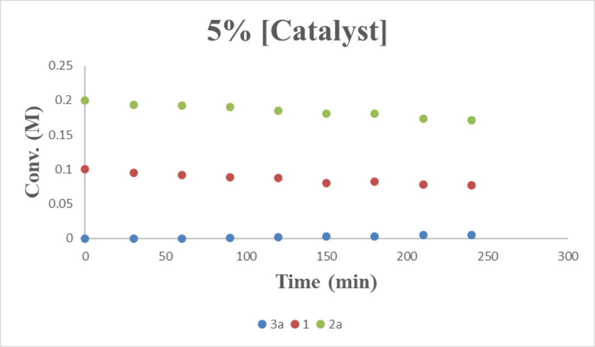

Figure S4 Time-course for the reaction with 5 % [Cu/**L14**] loading. Reaction conditions: [Cu/**L14**] = 0.005 M, (**1**) = 0.10 M, (**2a**) = 0.20 M, solvent = THF.

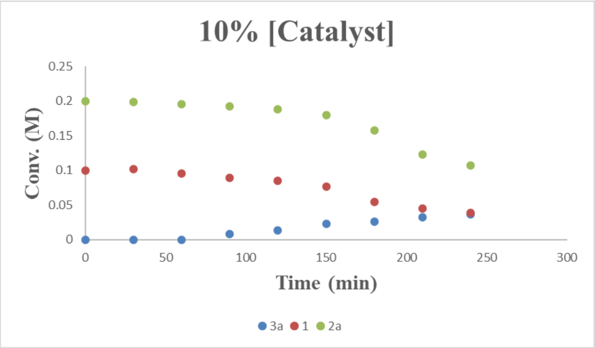

Figure S5 Time-course for the reaction with 10 % [Cu/**L14**] loading. Reaction conditions: [Cu/**L14**] = 0.010 M, (**1**) = 0.10 M, (**2a**) = 0.20 M, solvent = THF.

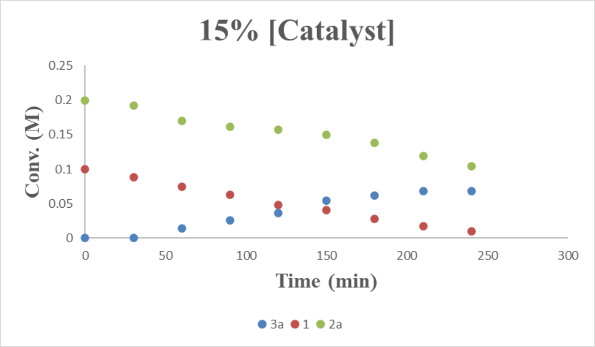

Figure S6 Time-course for the reaction with 15 % [Cu/**L14**] loading. Reaction conditions: [Cu/**L14**] = 0.015 M, (**1**) = 0.10 M, (**2a**) = 0.20 M, solvent = THF.

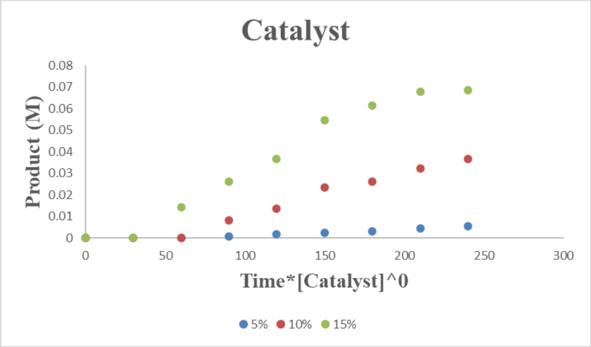

Figure S7 Various normalization analysis of product vs. [Cu/**L14**]^{0}·(time). The lack of overlay fitting suggests that the catalyst order is not 0.

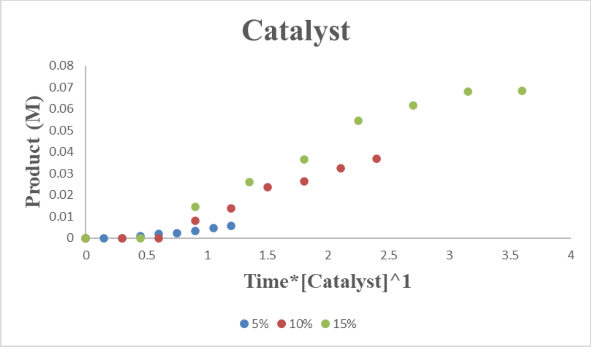

Figure S8 Various normalization analysis of product vs. [Cu/**L14**]^{1}·(time). The lack of overlay fitting suggests that the catalyst order is not 1.

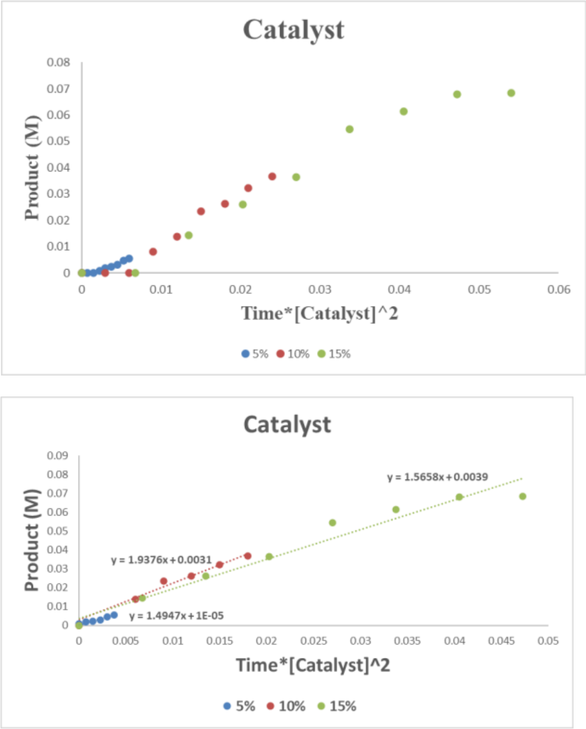

Figure S9 Various normalization analysis of product vs. [Cu/**L14**]^2·(time). The overlay fitting suggests that the catalyst order is 2.

Kinetic Plots with Different (^{t}BuOLi) Loading

The study of kinetic orders with (^{t}**BuOLi**) was completed following the general procedure described above.

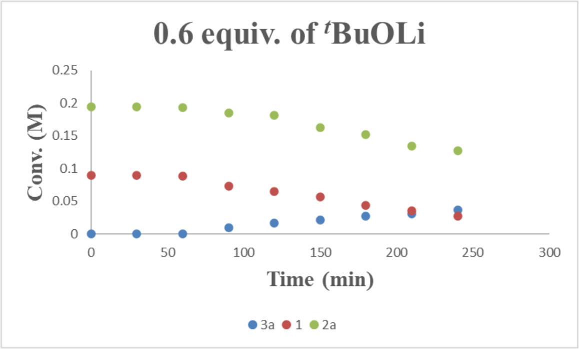

Figure S10 Time-course for the reaction with (^{t}**BuOLi**) = 0.06 mmol loading. Reaction conditions: [Cu/**L14**] = 0.01 M, (**1**) = 0.10 M, **(2a)** = 0.20 M, solvent = THF.

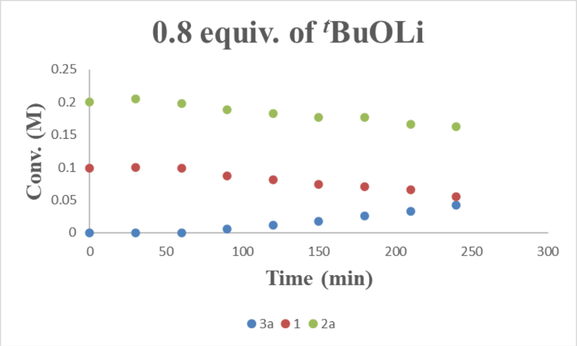

Figure S11 Time-course for the reaction with (^{t}**BuOLi**) = 0.08 mmol loading. Reaction conditions: [Cu/**L14**] = 0.01 M, (**1**) = 0.10 M, **(2a)** = 0.20 M, solvent = THF.

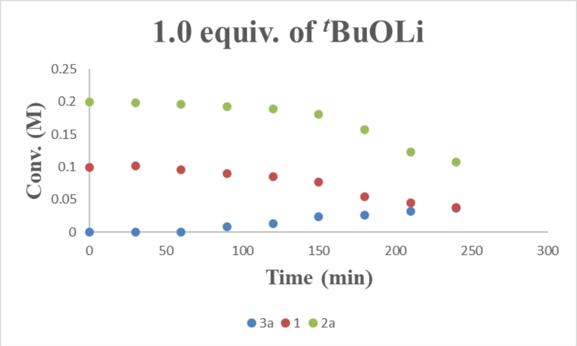

Figure S12 Time-course for the reaction with (^{t}**BuOLi**) = 0.10 mmol loading. Reaction conditions: [Cu/**L14**] = 0.01 M, (**1**) = 0.10 M, **(2a)** = 0.20 M, solvent = THF.

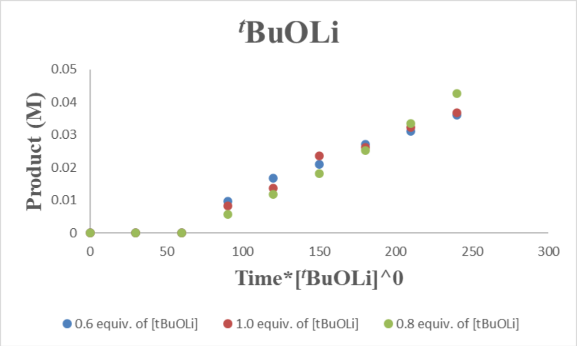

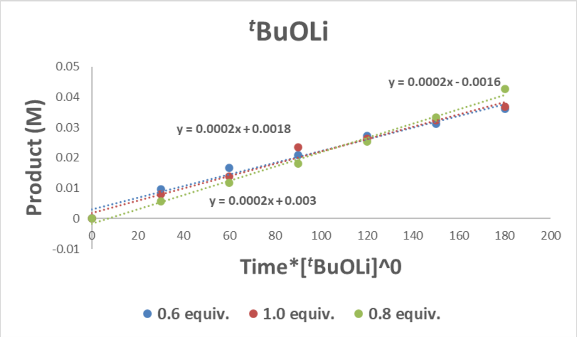

Figure S13 Various normalization analysis of product vs. (^{t}**BuOLi**)^0·(time). The overlay fitting suggests that the benzoylsilane (^{t}**BuOLi**) order is 0.

Kinetic Plots with Different (1) Loading

The study of kinetic orders with (**1**) was completed following the general procedure described above.

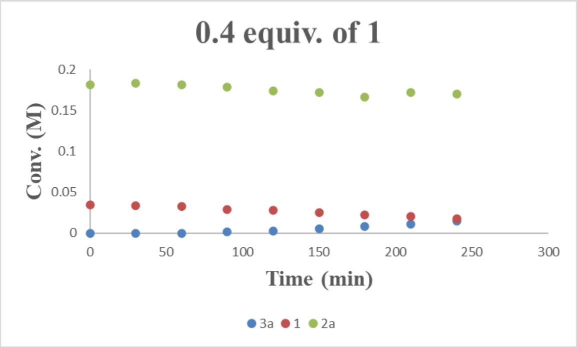

Figure S14 Time-course for the reaction with (**1**) = 0.04 mmol loading. Reaction conditions: [Cu/**L14**] = 0.01 M, (**1**) = 0.04 M, **(2a)** = 0.20 M, solvent = THF.

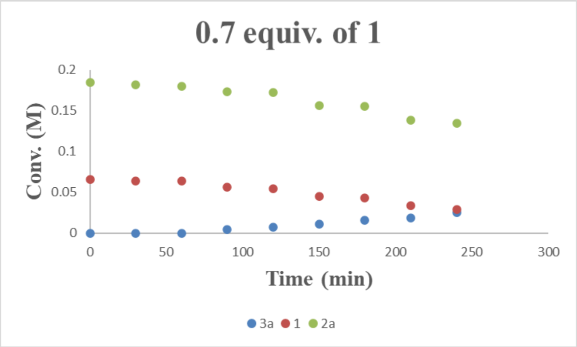

Figure S15 Time-course for the reaction with (**1**) = 0.07 mmol loading. Reaction conditions: [Cu/**L14**] = 0.01 M, (**1**) = 0.07 M, **(2a)** = 0.20 M, solvent = THF.

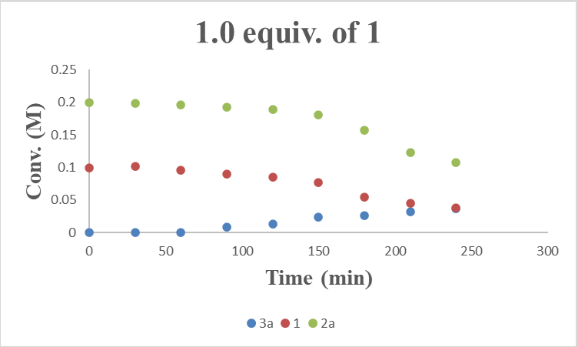

Figure S16 Time-course for the reaction with (**1**) = 0.10 mmol loading. Reaction conditions: [Cu/**L14**] = 0.01 M, (**1**) = 0.10 M, **(2a)** = 0.20 M, solvent = THF.

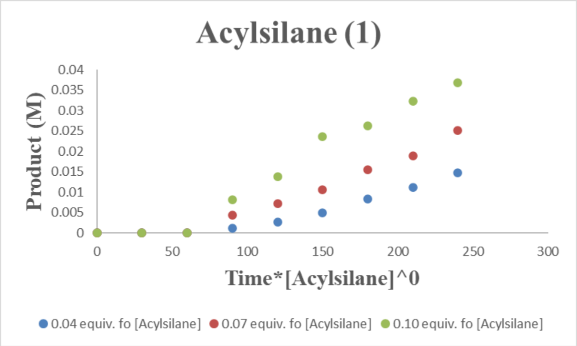

Figure S17 Various normalization analysis of product vs. $(\mathbf{1})^{0}\cdot$(time). The lack of overlay fitting suggests that the benzoylsilane (**1**) order is not 0.

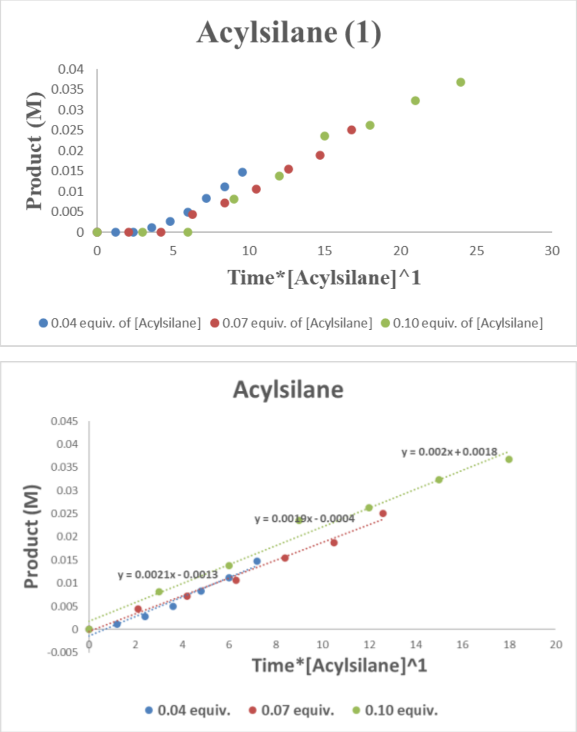

Figure S18 Various normalization analysis of product vs. $(\mathbf{1})^{1}\cdot$(time). The overlay fitting suggests that the benzoylsilane (**1**) order is 1.

Kinetic Plots with Different Benzoxazole (2a) Loading

The study of kinetic orders with benzoxazole **(2a)** was completed following the general procedure described above.

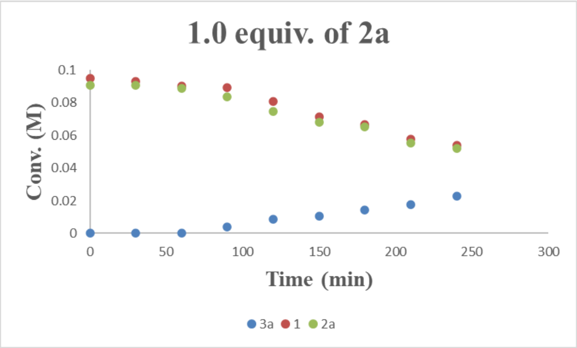

Figure S19 Time-course for the reaction with (**2a**) = 0.10 mmol loading. Reaction conditions: [Cu/**L14**] = 0.01 M, (**1**) = 0.10 M, (**2a**) = 0.10 M, solvent = THF.

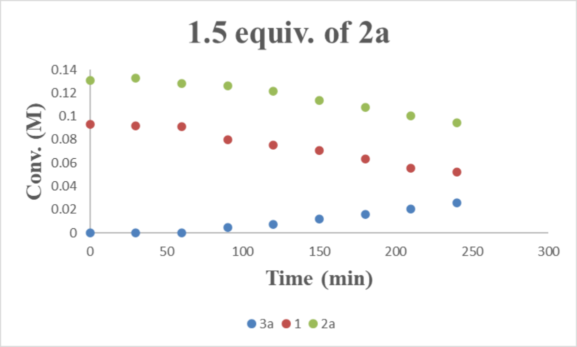

Figure S20 Time-course for the reaction with (**2a**) = 0.15 mmol loading. Reaction conditions: [Cu/**L14**] = 0.01 M, (**1**) = 0.10 M, (**2a**) = 0.15 M, solvent = THF.

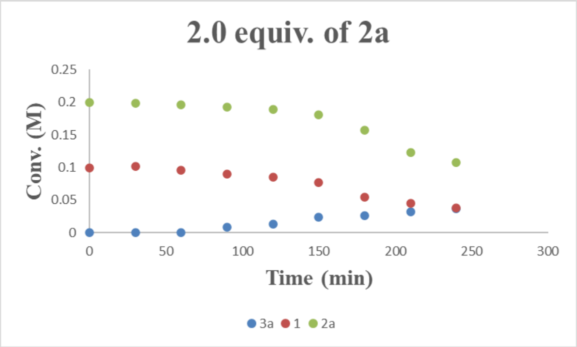

Figure S21 Time-course for the reaction with (**2a**) = 0.20 mmol loading. Reaction conditions: [Cu/**L14**] = 0.01 M, (**1**) = 0.10 M, (**2a**) = 0.20 M, solvent = THF.

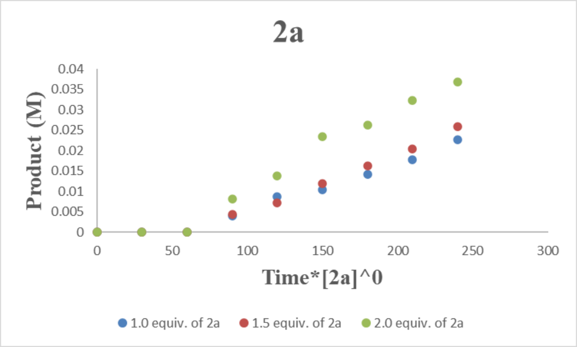

Figure S22 Various normalization analysis of product vs. (**2a**)^0·(time). The lack of overlay fitting suggests that the ethynylbenzene (**2a**) order is not 0.

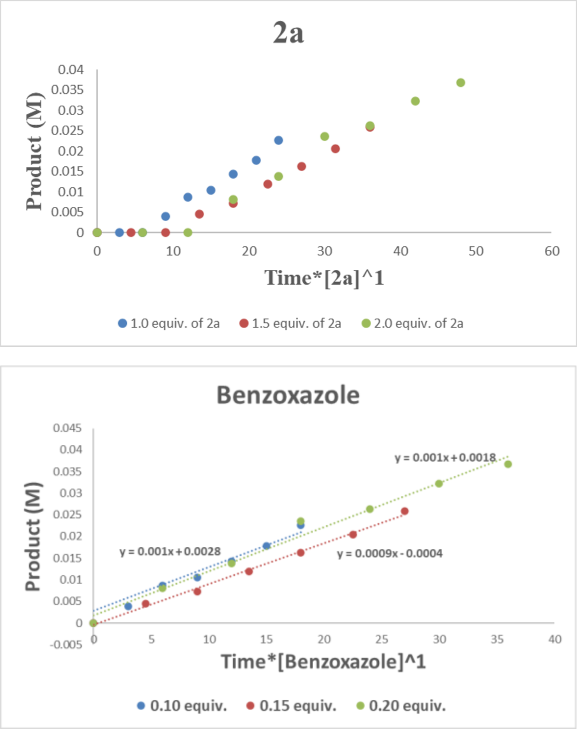

Figure S23 Various normalization analysis of product vs. (**2a**)^1·(time). The overlay fitting suggests that the ethynylbenzene (**2a**) order is 1.

7. X-Ray Structure

Single crystal of **3ad** was obtained by recrystallization from PE/DCM. The molecular structure and X-ray diffraction data/refinement of **3ad** was shown below.

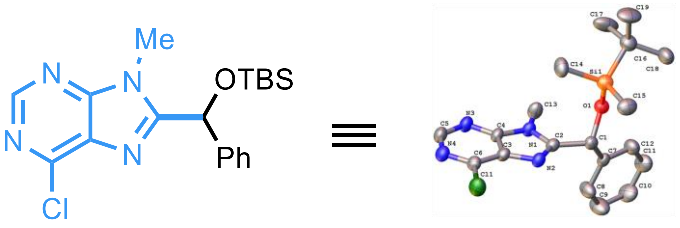

3ad X-Ray structure (CCDC 2322015)

Bond precision:	C-C = 0.0073 A			Wavelength = 1.34139
Cell:	a = 7.0461(5)	b = 23.7846(15)	c =12.7294(8)	
	Alpha = 90	beta =101.868(3)	gamma = 90	
Temperature: 213 K				

	Calculated	Reported
Volume	2087.7(2)	2087.7(2)
Space group	P 21/n	P 1 21/n 1
Hall group	-P 2yn	-P 2yn
Moiety formula	C19H25ClN4OSi	C19H25ClN4OSi
Sum formula	C19H25ClN4OSi	C19H25ClN4OSi
Mr	388.97	388.97
Dx,g cm-3	1.237	1.238
Z	4	4
Mu (mm-1)	1.503	1.503
F000	824.0	824.0
F000’	827.60	
h,k,lmax	8,29,15	8,28,15
Nref	3978	3968
Tmin,Tmax	0.900, 0.928	0.500, 0.751
Tmin’	0.900	

Correction method = # Reported T Limits: Tmin = 0.500 Tmax = 0.751 AbsCorr = MULTI-SCAN

Data completeness = 0.997	Theta(max) = 54.896
R(reflections) = 0.0914(2690)	wR2(reflections) = 0.3027(3968)
S = 1.071	Npar = 309

8. References

[1] Karthik, S.; Gandhi, T. Palladium(II)/N-Heterocyclic Carbene-Catalyzed Direct C−H Acylation of Hetero-arenes with N-Acylsaccharins. *Org. Lett*. **2017**, *19*, 5486−5489.

[2] Skinner, W. A.; Gualtiere, F.; Brody, G.; Fieldsteel, A. H. Antiviral agents. 1. Benzothiazole and benzoxazole analogs of 2-(.alpha.-hydroxybenzyl)benzimidazole. *J. Med. Chem*. **1971**, *14*, 546−549.

[3] Park, B. Y.; Montgomery, T. P.; Garza, V.; Krische, M. J. Ruthenium Catalyzed Hydrohydroxy alkylation of Isoprene with Heteroaromatic Secondary Alcohols: Isolation and Reversible Formation of the Putative Metallacycle Intermediate. *J. Am.Chem.Soc*. **2013**, *135*, 16320−16323.

[4] Heynderickx, A.; Guglielmetti, R.; Dubest, R.; Aubard, J.; Samat, A. Sulfinyl- and Sulfonyl-Substituted 2-Benzylbenzoxazoles and 2-Benzylbenzothiazoles as Potential Photochromic Compounds. *Synthesis*, **2003**, *7*, 1112−1116.

[5] Tan, G.; He, S.; Huang, X.; Liao, X.; Cheng, Y.; You, J. Cobalt-Catalyzed Oxidative C−H/C−H Cross-Coupling Between Two Heteroarenes. *Angew. Chem. Int. Ed*. **2016**, *55*, 10414−10418.

[6] Zhang, L.; Cheng, J.; Ohishi, T.; Hou, Z. *Angew. Chem. Int. Ed*. **2010**, *49*, 8670–8673.

9. NMR Spectra

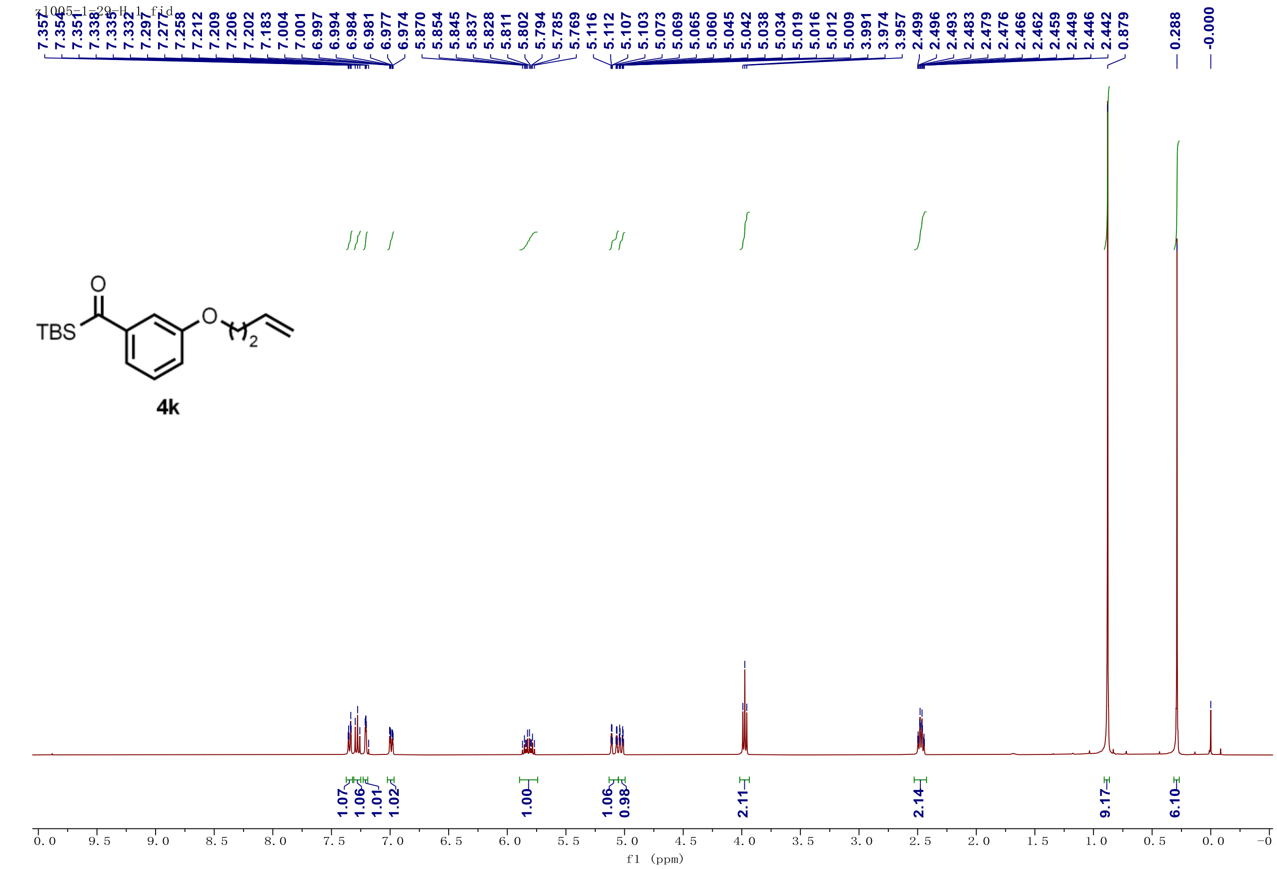

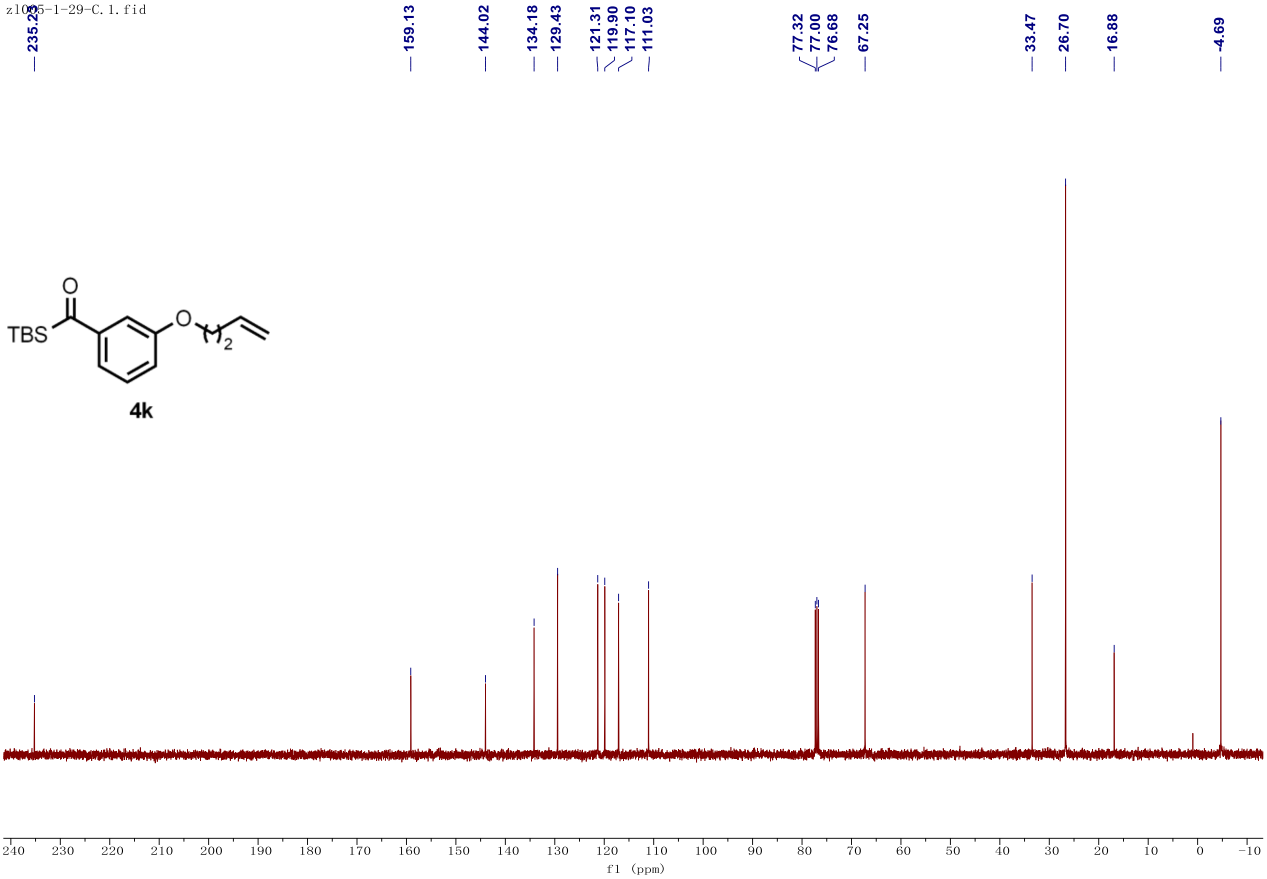

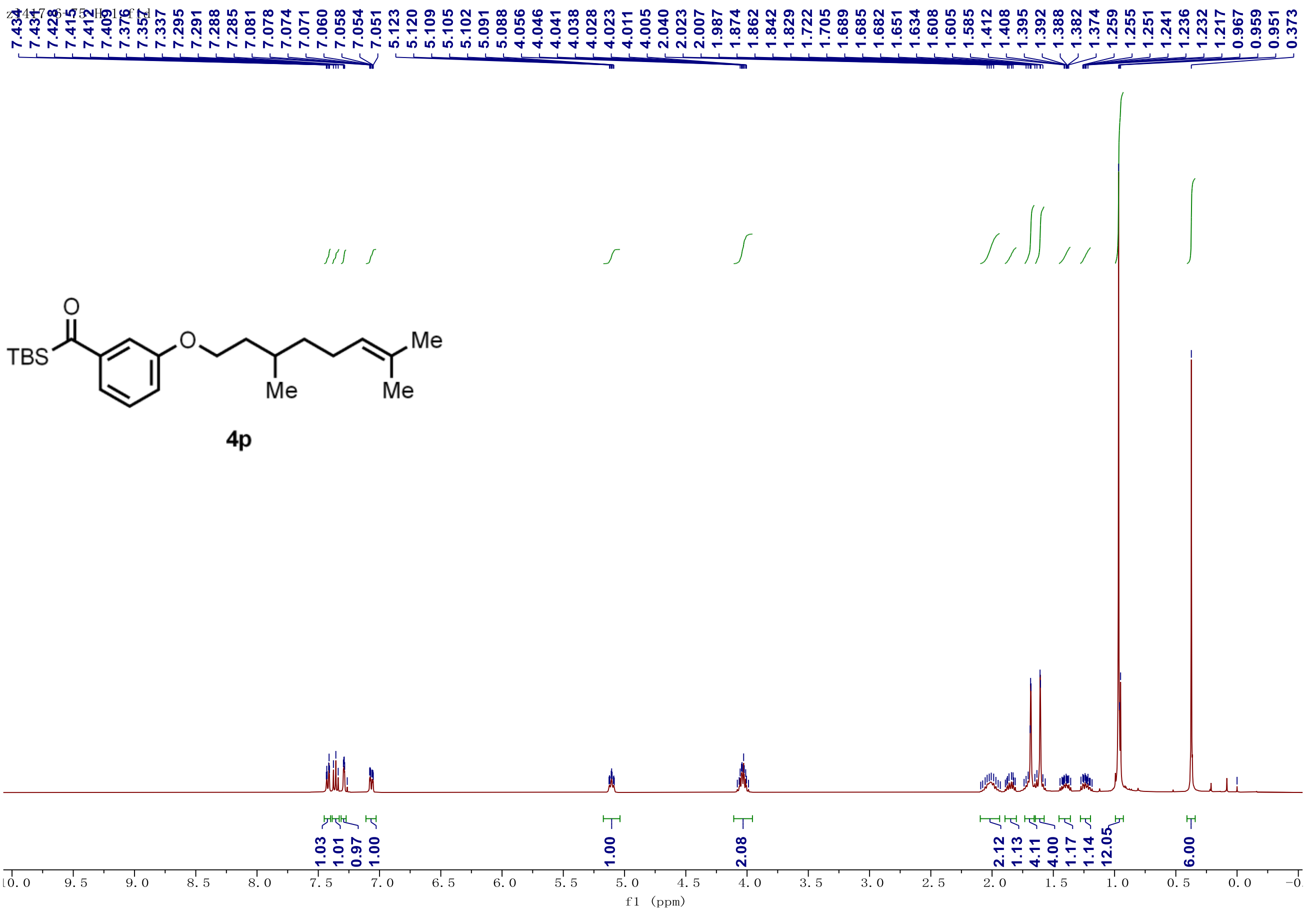
7.434 7.431 7.428 7.415 7.412 7.409 7.376 7.357 7.337 7.295 7.291 7.288 7.285 7.081 7.078 7.074 7.071 7.060 7.058 7.054 7.051 5.123 5.120 5.109 5.105 5.102 5.091 5.088 4.056 4.046 4.041 4.038 4.028 4.023 4.011 4.005 2.040 2.023 2.007 1.987 1.874 1.862 1.842 1.829 1.722 1.705 1.689 1.685 1.682 1.651 1.634 1.608 1.605 1.585 1.412 1.408 1.395 1.392 1.388 1.382 1.374 1.259 1.255 1.251 1.241 1.236 1.232 1.217 0.967 0.959 0.951 0.373
TBS
O
Me
Me
Me
4p
1.03 1.01 0.97 1.00 1.00 2.08 2.12 1.13 4.11 4.00 1.17 1.14 12.05 6.00
f1 (ppm)

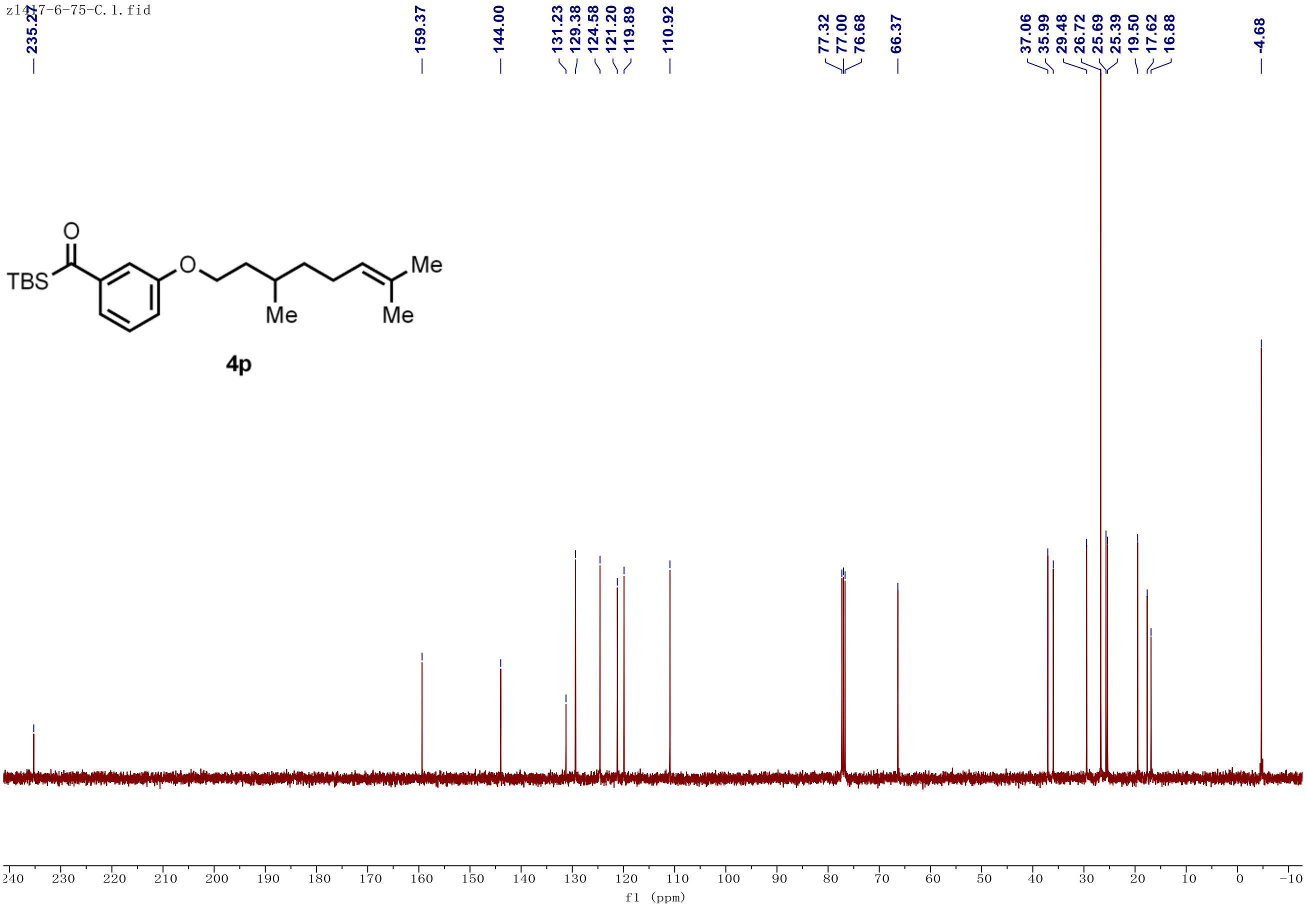
235.27 159.37 144.00 131.23 129.38 124.58 121.20 119.89 110.92 77.32 77.00 76.68 66.37 37.06 35.99 29.48 26.72 25.69 25.39 19.50 17.62 16.88 -4.68
TBS
O
Me
Me
Me
4p
f1 (ppm)

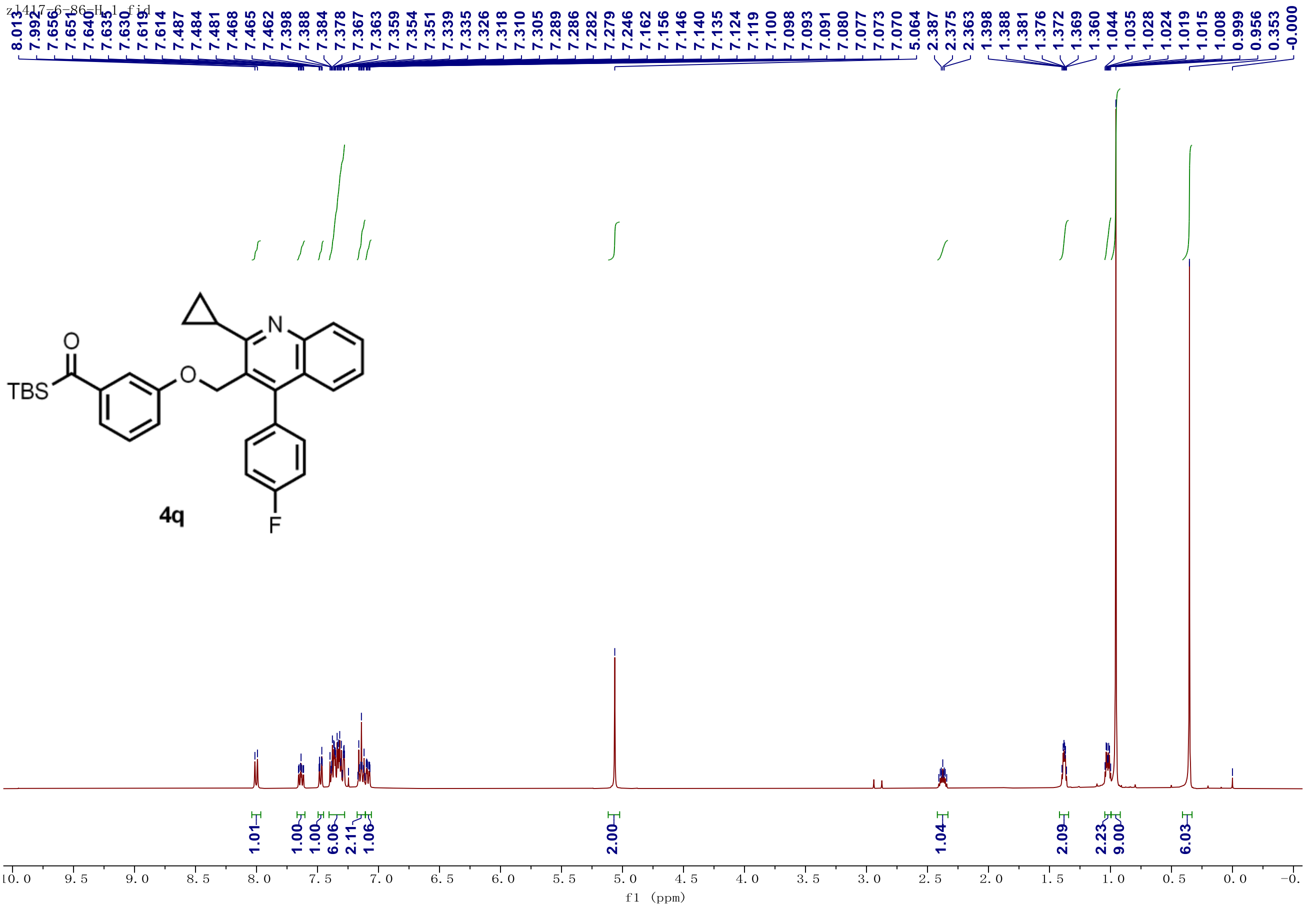
zl417-6-86-H.1.fid
8.013
7.992
7.656
7.651
7.640
7.635
7.630
7.619
7.614
7.487
7.484
7.481
7.468
7.465
7.462
7.398
7.388
7.384
7.378
7.367
7.363
7.359
7.354
7.351
7.339
7.335
7.326
7.318
7.310
7.305
7.289
7.286
7.282
7.279
7.246
7.162
7.156
7.146
7.140
7.135
7.124
7.119
7.100
7.098
7.093
7.091
7.080
7.077
7.073
7.070
5.064
2.387
2.375
2.363
1.398
1.388
1.381
1.376
1.372
1.369
1.360
1.044
1.035
1.028
1.024
1.019
1.015
1.008
0.999
0.956
0.353
-0.000
TBS
O
N
F
4q
1.01
1.00
1.00
6.06
2.11
1.06
2.00
1.04
2.09
2.23
9.00
6.03
f1 (ppm)

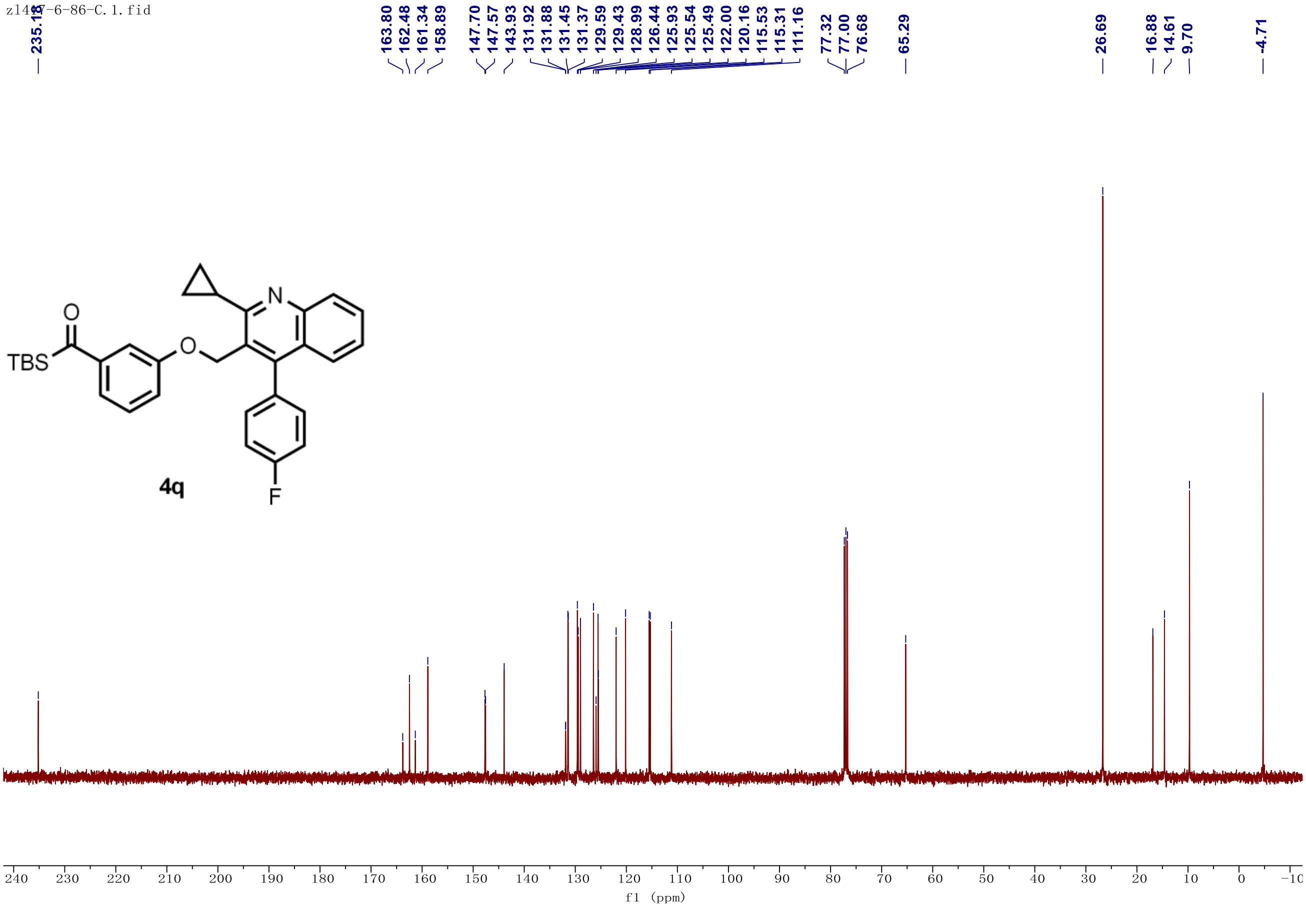
zl417-6-86-C.1.fid
235.18
163.80
162.48
161.34
158.89
147.70
147.57
143.93
131.92
131.88
131.45
131.37
129.59
129.43
128.99
126.44
125.93
125.54
125.49
122.00
120.16
115.53
115.31
111.16
77.32
77.00
76.68
65.29
26.69
16.88
14.61
9.70
-4.71
TBS
O
N
F
4q
f1 (ppm)

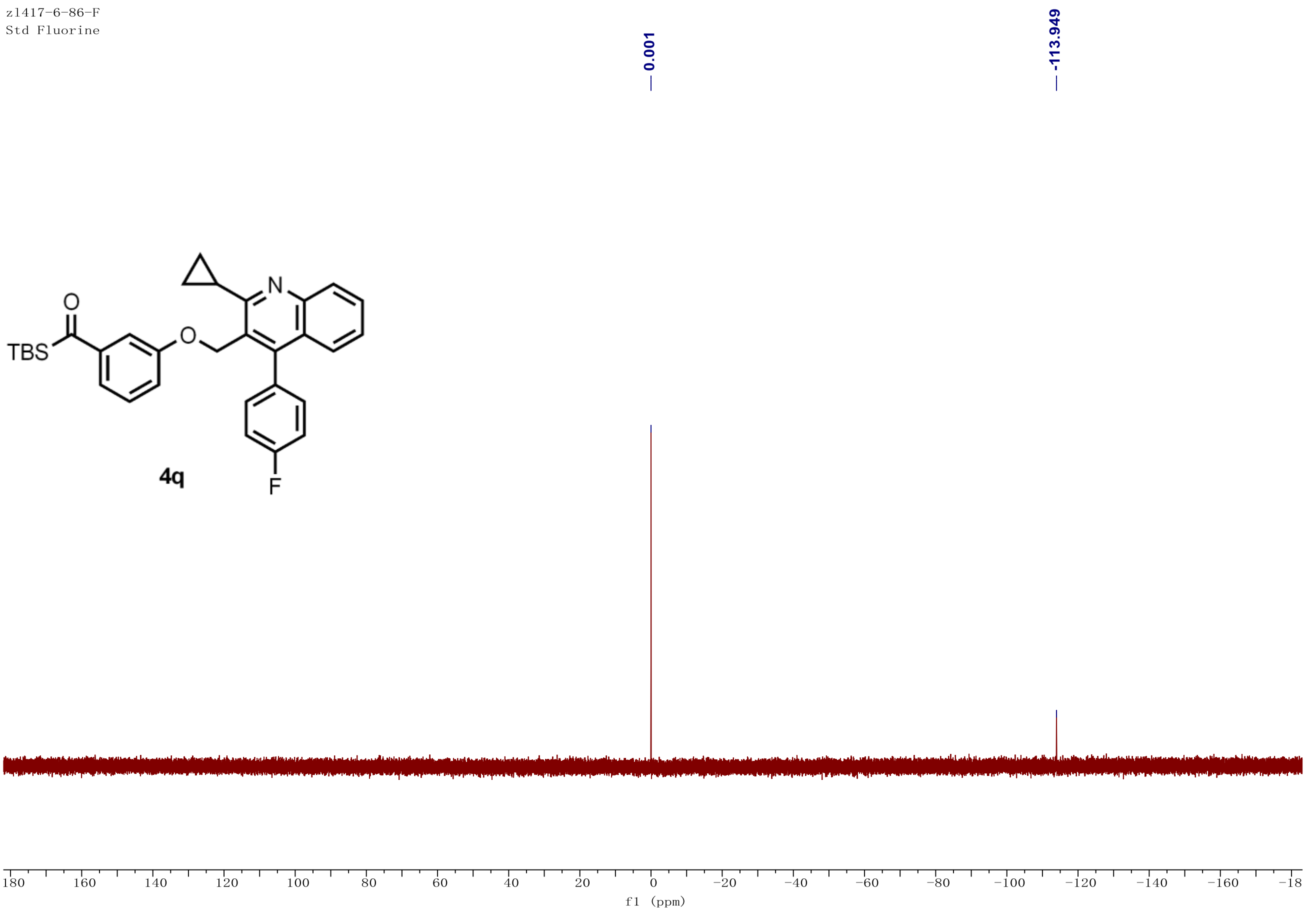
zl417-6-86-F
Std Fluorine
0.001
-113.949
TBS
O
N
F
4q
180 160 140 120 100 80 60 40 20 0 -20 -40 -60 -80 -100 -120 -140 -160
f1 (ppm)

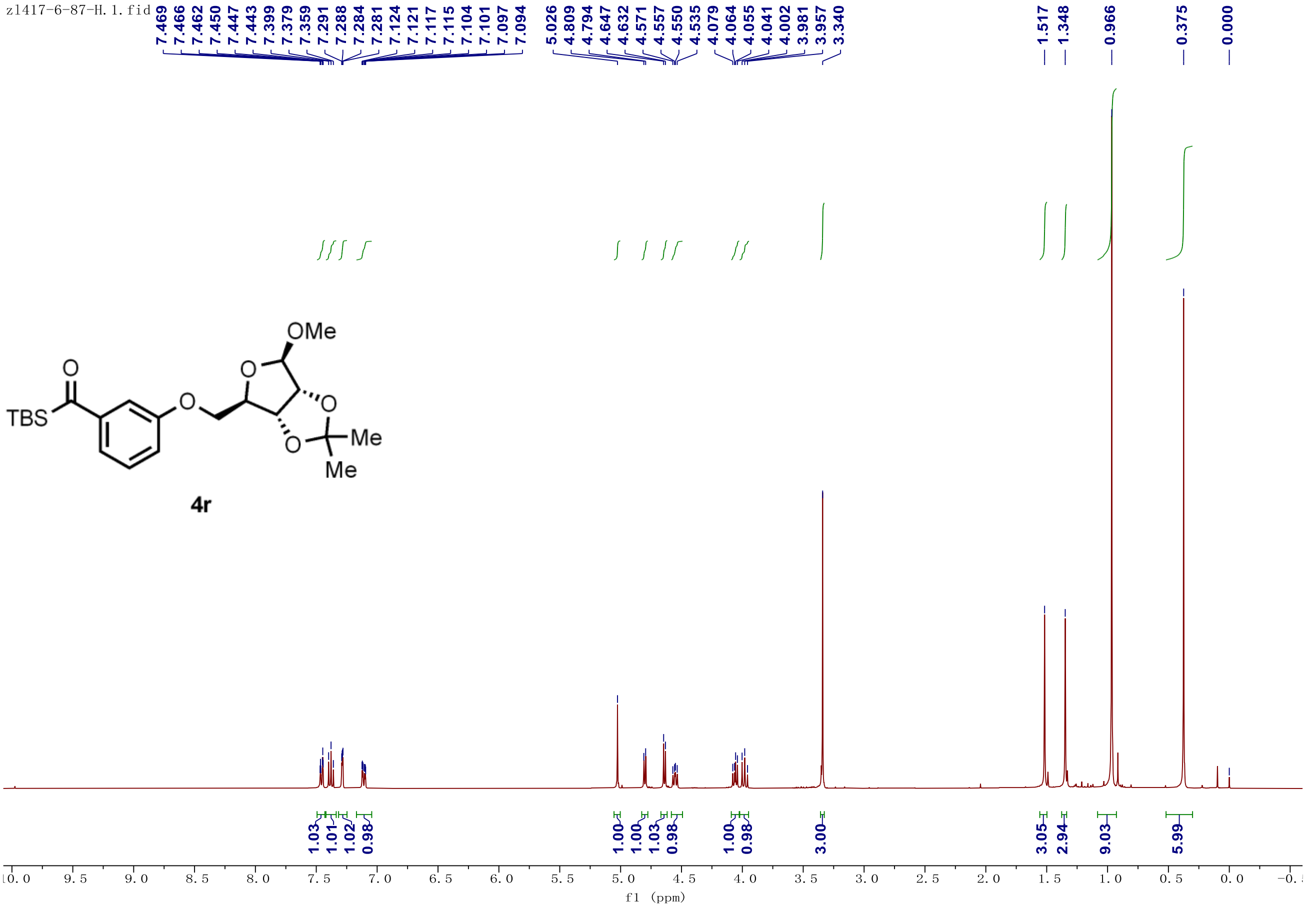
zl417-6-87-H.1.fid
7.469 7.466 7.462 7.450 7.447 7.443 7.399 7.379 7.359 7.291 7.288 7.284 7.281 7.124 7.121 7.117 7.115 7.104 7.101 7.097 7.094
5.026 4.809 4.794 4.647 4.632 4.571 4.557 4.550 4.535 4.079 4.064 4.055 4.041 4.002 3.981 3.957 3.340
1.517 1.348 0.966 0.375 0.000
TBS
O
OMe
Me
Me
4r
1.03 1.01 1.02 0.98 1.00 1.00 1.03 0.98 1.00 0.98 3.00 3.05 2.94 9.03 5.99
10.0 9.5 9.0 8.5 8.0 7.5 7.0 6.5 6.0 5.5 5.0 4.5 4.0 3.5 3.0 2.5 2.0 1.5 1.0 0.5 0.0
f1 (ppm)

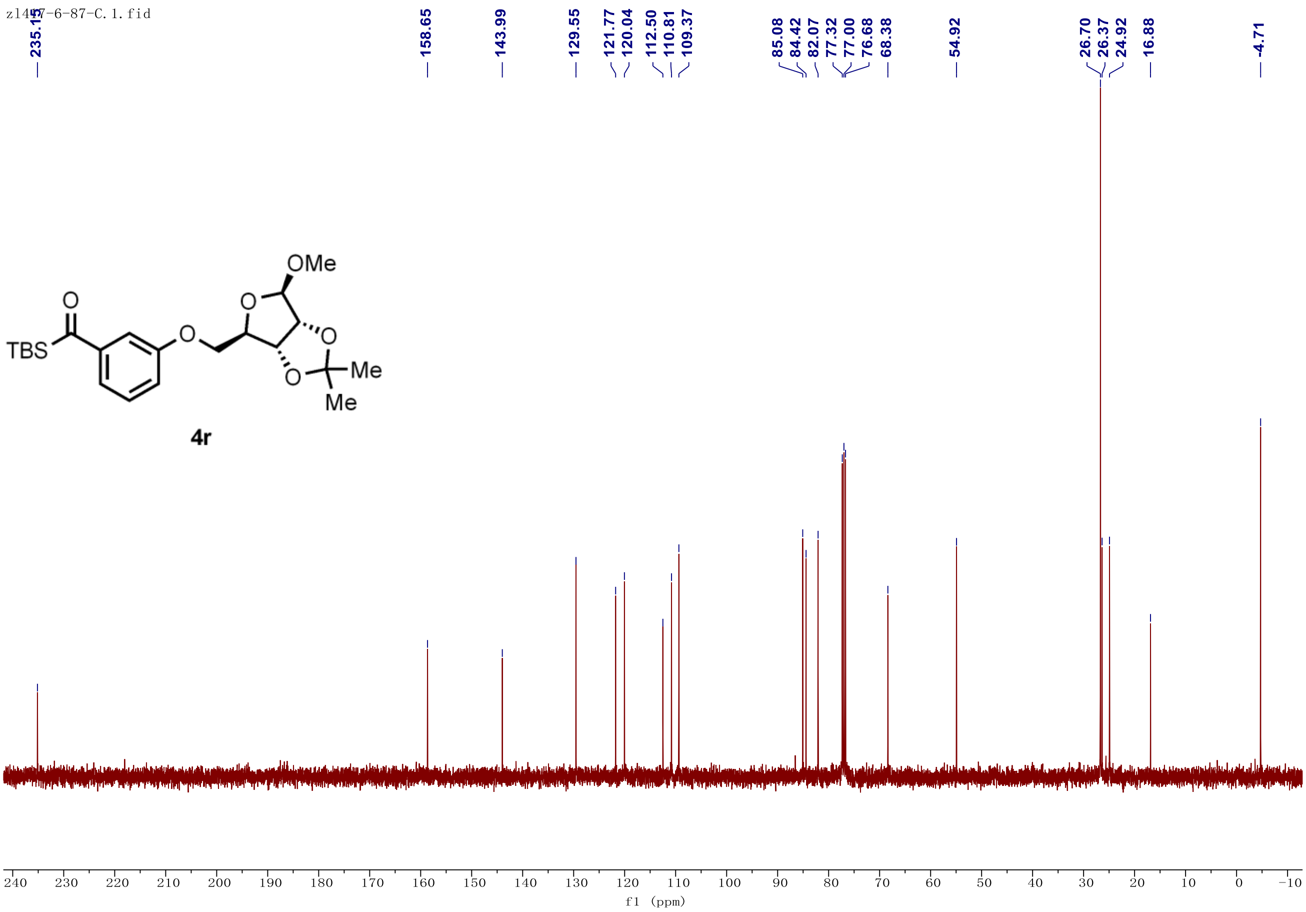
235.15
158.65
143.99
129.55
121.77
120.04
112.50
110.81
109.37
85.08
84.42
82.07
77.32
77.00
76.68
68.38
54.92
26.70
26.37
24.92
16.88
-4.71
OMe
O
TBS
4r
Me
f1 (ppm)

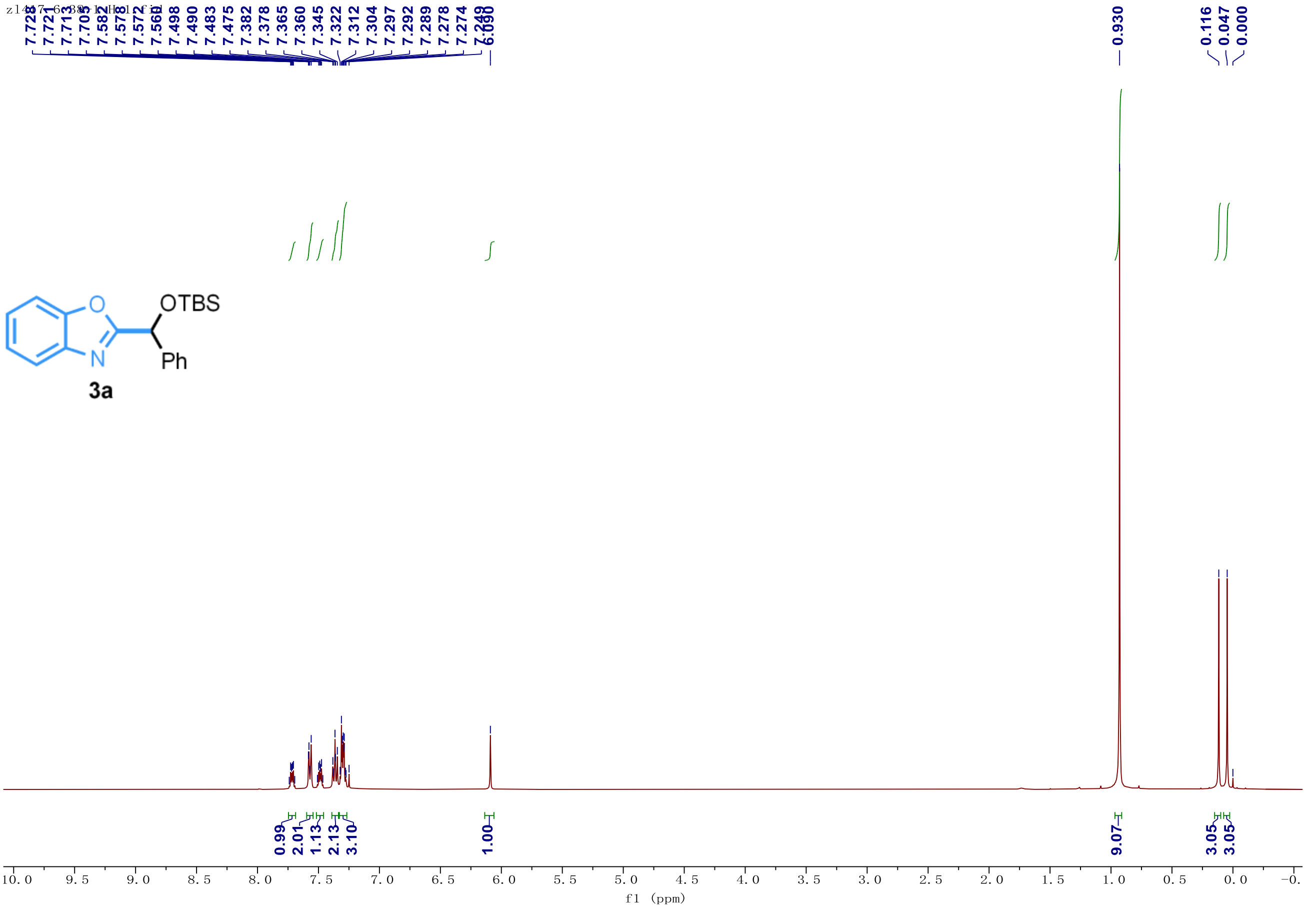
0.930
0.116
0.047
0.000
OTBS
Ph
3a
0.99
2.01
1.13
2.13
3.10
1.00
9.07
3.05
3.05
f1 (ppm)

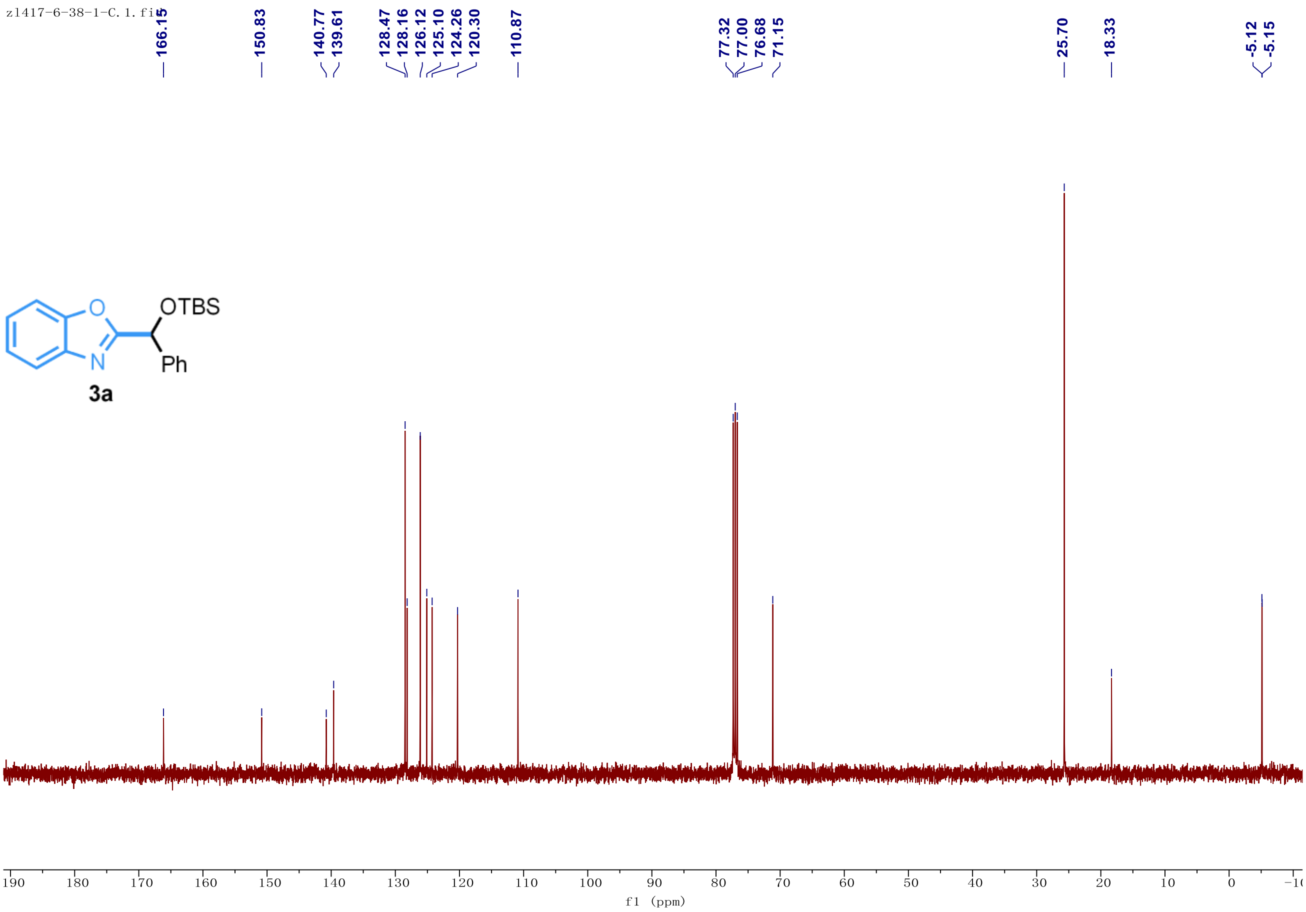
z1417-6-38-1-C.1.fid
166.15
150.83
140.77
139.61
128.47
128.16
126.12
125.10
124.26
120.30
110.87
77.32
77.00
76.68
71.15
25.70
18.33
-5.12
-5.15
OTBS
O
N
Ph
3a
f1 (ppm)

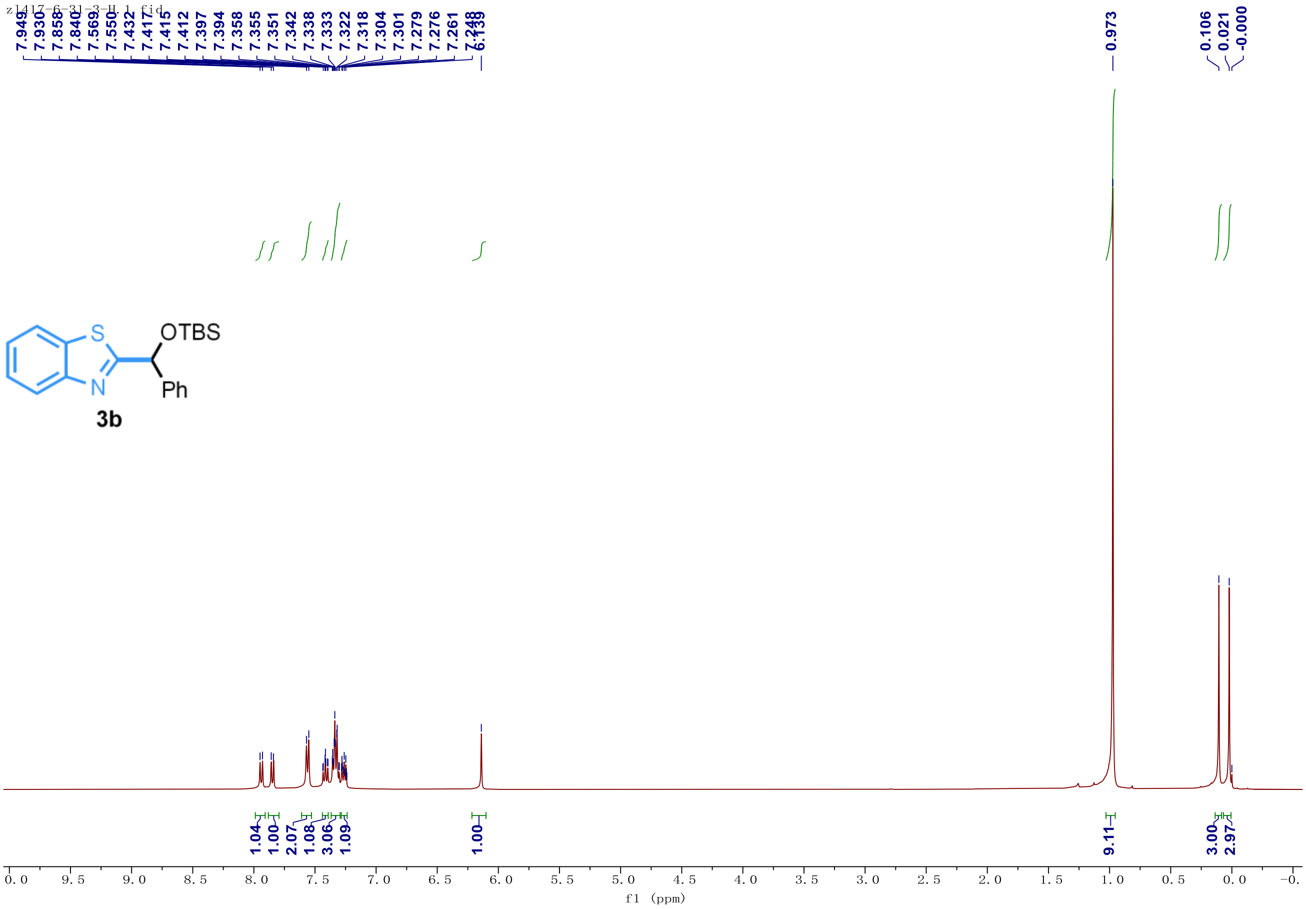
z1417-6-31-3-H.1.fid
7.949
7.930
7.858
7.840
7.569
7.550
7.432
7.417
7.415
7.412
7.397
7.394
7.358
7.355
7.351
7.342
7.338
7.333
7.322
7.318
7.304
7.301
7.279
7.276
7.261
7.248
6.139
0.973
0.106
0.021
-0.000
OTBS
S
N
Ph
3b
1.04
1.00
2.07
1.08
3.06
1.09
1.00
9.11
3.00
2.97
f1 (ppm)

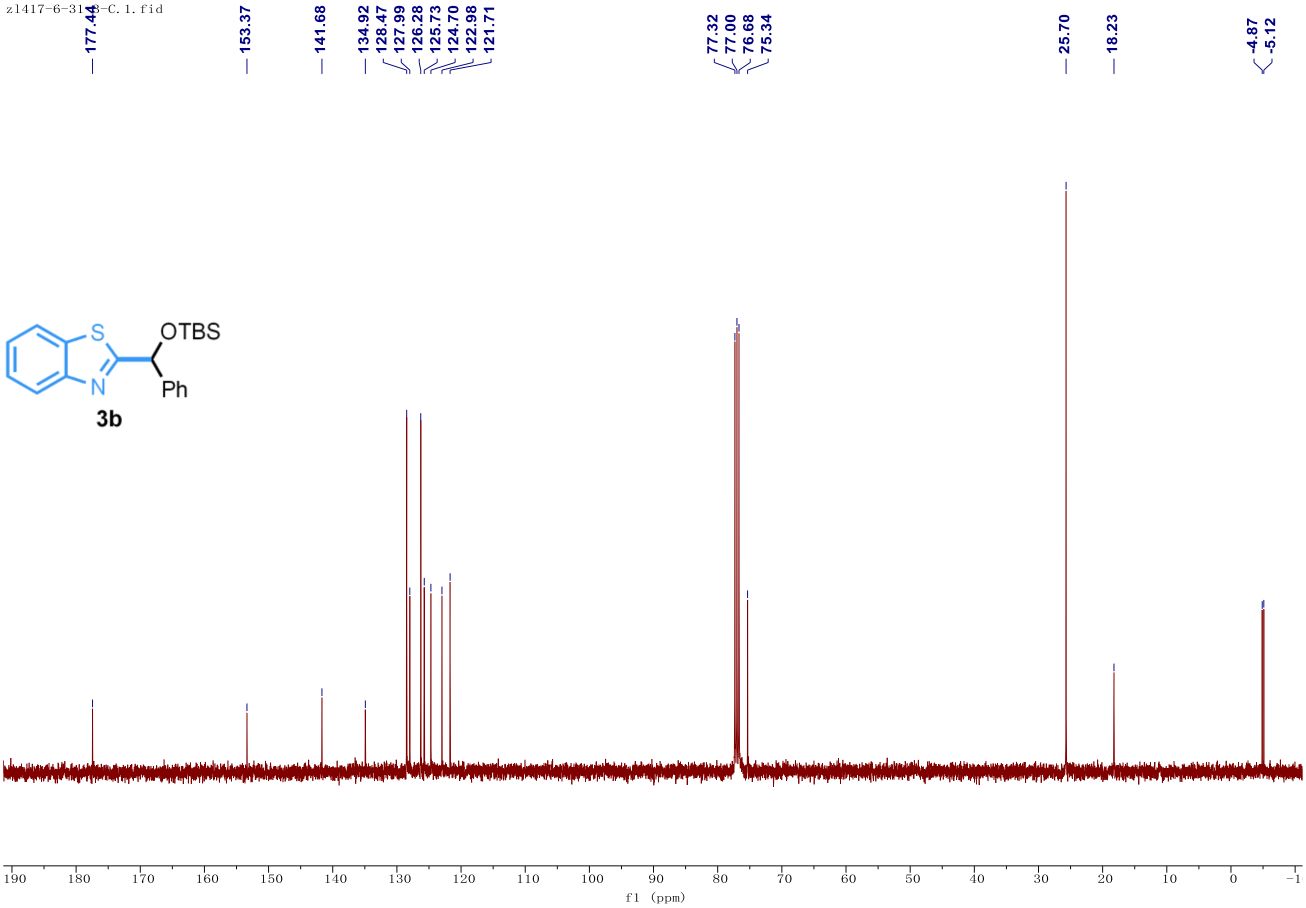
z1417-6-31-3-C.1.fid
177.44
153.37
141.68
134.92
128.47
127.99
126.28
125.73
124.70
122.98
121.71
77.32
77.00
76.68
75.34
25.70
18.23
-4.87
-5.12
OTBS
S
N
Ph
3b
f1 (ppm)

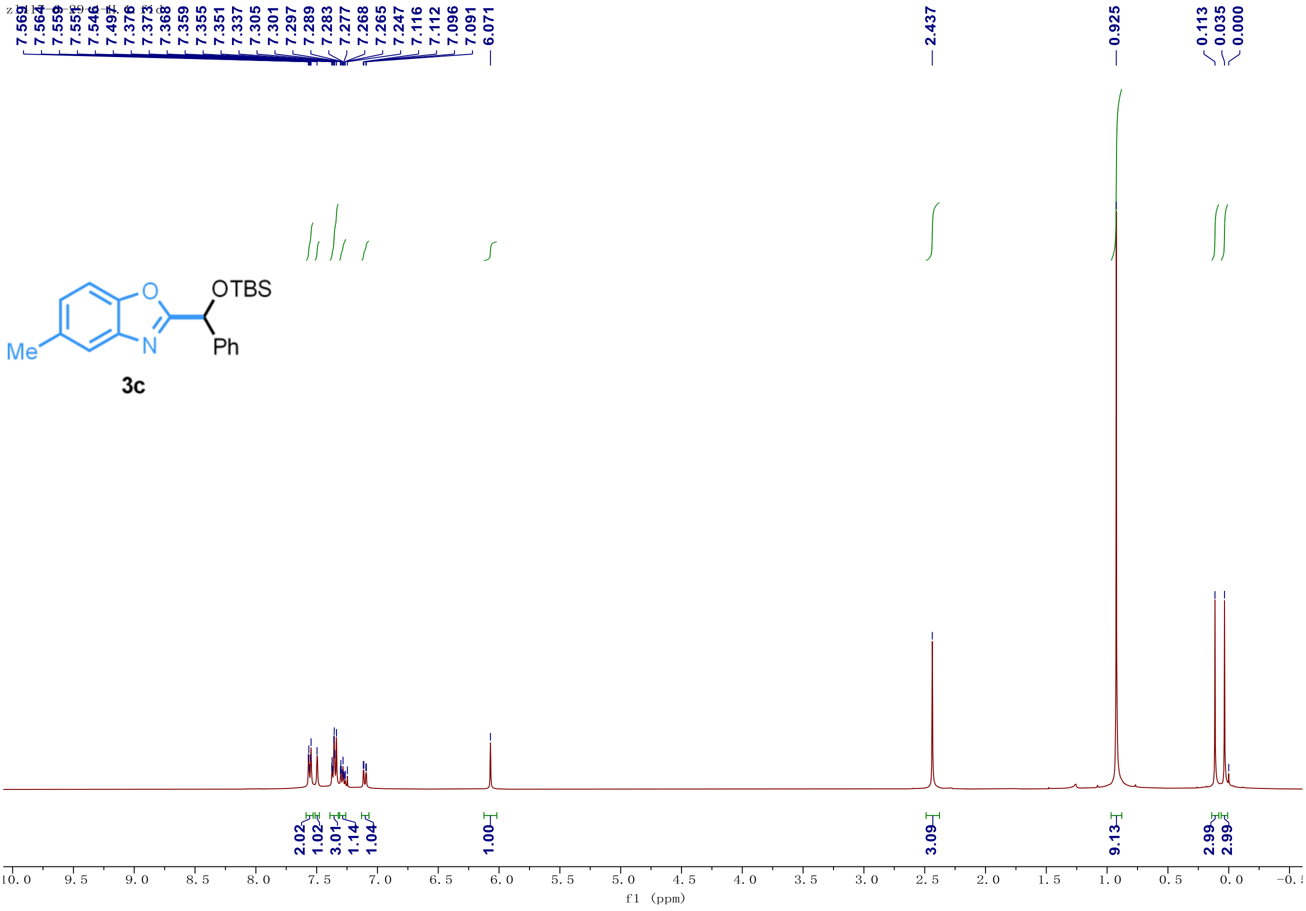
7.569
7.564
7.559
7.551
7.546
7.497
7.376
7.373
7.368
7.359
7.355
7.351
7.337
7.305
7.301
7.297
7.289
7.283
7.277
7.268
7.265
7.247
7.116
7.112
7.096
7.091
6.071
2.437
0.925
0.113
0.035
0.000
OTBS
O
N
Me
Ph
3c
2.02
1.02
3.01
1.14
1.04
1.00
3.09
9.13
2.99
2.99
f1 (ppm)

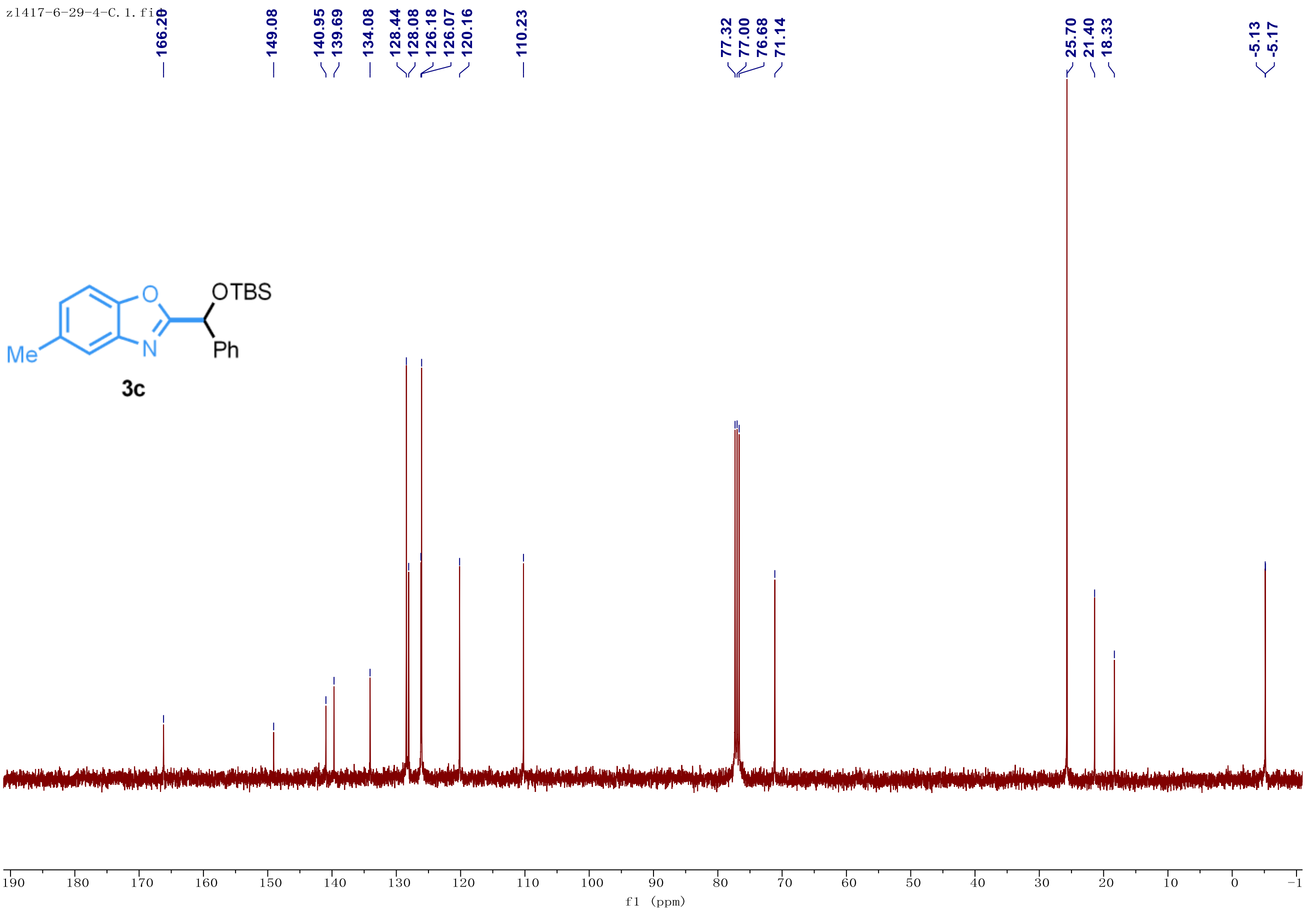

zl417-6-29-4-C.1.fid
OTBS
Me
O
N
Ph
3c
f1 (ppm)

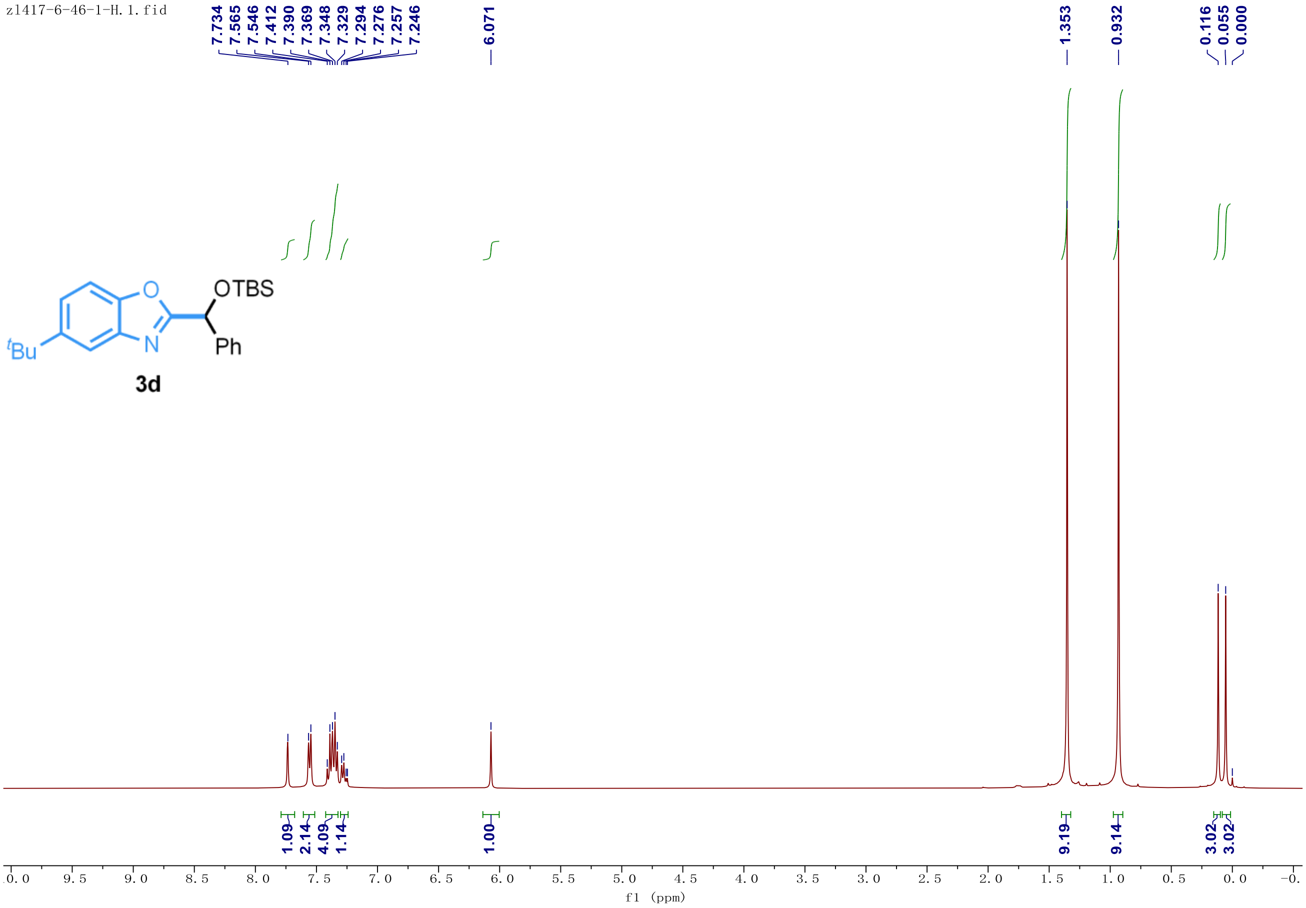

zl417-6-46-1-H.1.fid
OTBS
^{t}Bu
O
N
Ph
3d
f1 (ppm)

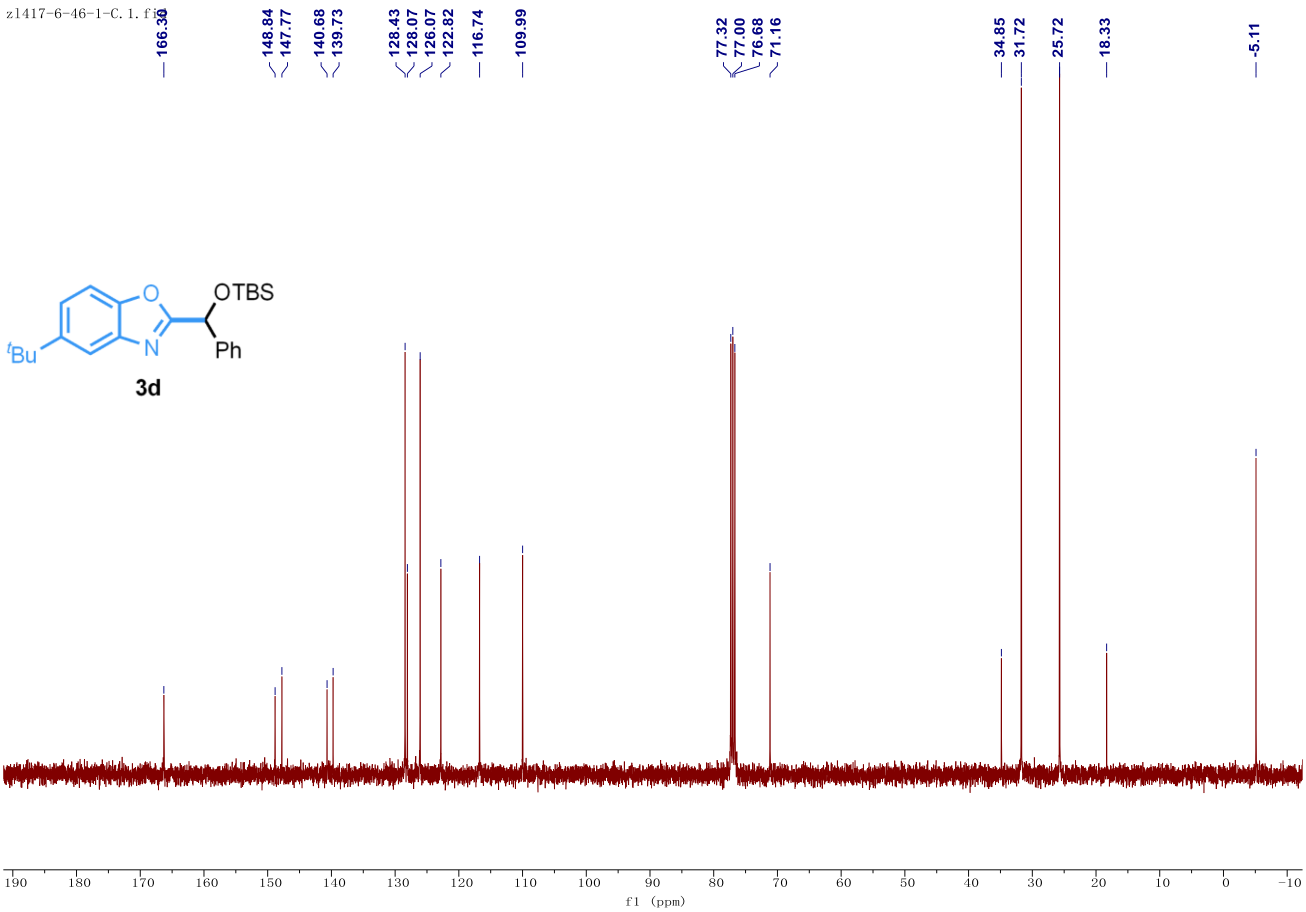

zl417-6-46-1-C.1.fid
166.30
148.84
147.77
140.68
139.73
128.43
128.07
126.07
122.82
116.74
109.99
77.32
77.00
76.68
71.16
34.85
31.72
25.72
18.33
-5.11
OTBS
Ph
3d
f1 (ppm)

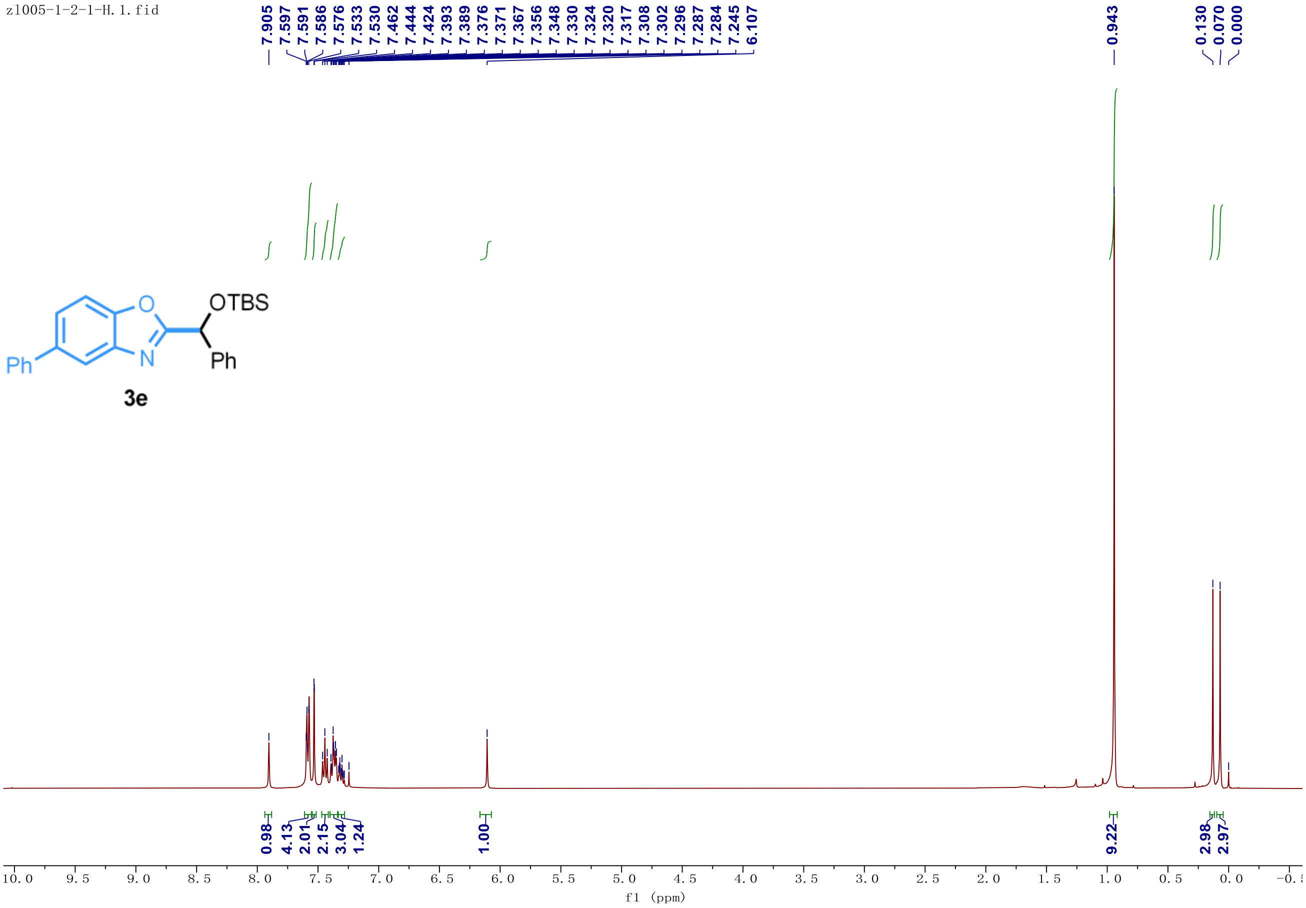

zl005-1-2-1-H.1.fid
7.905
7.597
7.591
7.586
7.576
7.533
7.530
7.462
7.444
7.424
7.393
7.389
7.376
7.371
7.367
7.356
7.348
7.330
7.324
7.320
7.317
7.308
7.302
7.296
7.287
7.284
7.245
6.107
0.943
0.130
0.070
0.000
OTBS
Ph
Ph
3e
0.98
4.13
2.01
2.15
3.04
1.24
1.00
9.22
2.98
2.97
f1 (ppm)

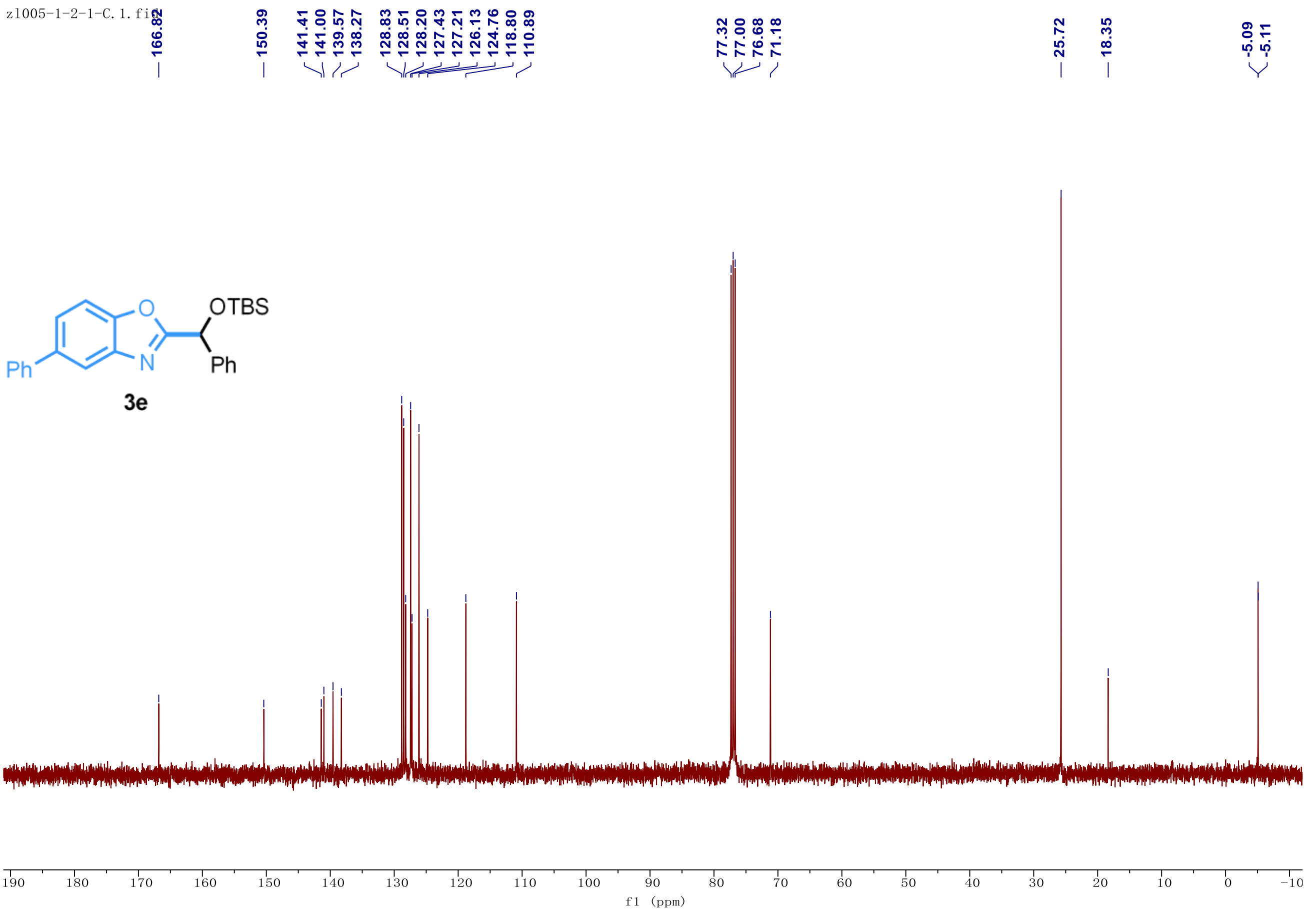
z1005-1-2-1-C.1.fid
166.82
150.39
141.41
141.00
139.57
138.27
128.83
128.51
128.20
127.43
127.21
126.13
124.76
118.80
110.89
77.32
77.00
76.68
71.18
25.72
18.35
-5.09
-5.11
OTBS
Ph
Ph
O
N
3e
f1 (ppm)

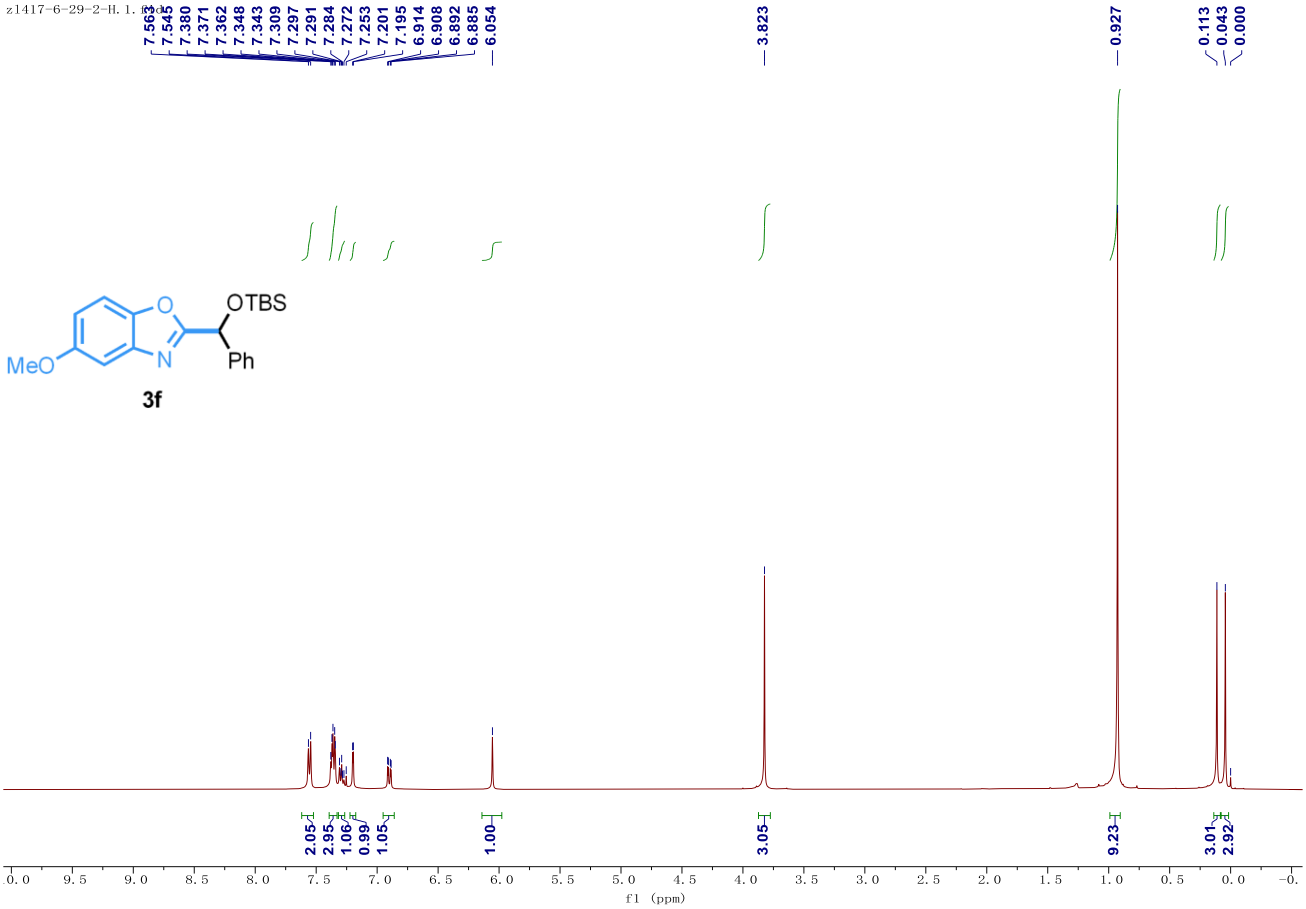
z1417-6-29-2-H.1.fid
7.563
7.545
7.380
7.371
7.362
7.348
7.343
7.309
7.297
7.291
7.284
7.272
7.253
7.201
7.195
6.914
6.908
6.892
6.885
6.054
3.823
0.927
0.113
0.043
0.000
OTBS
Ph
MeO
O
N
3f
2.05
2.95
1.06
0.99
1.05
1.00
3.05
9.23
3.01
2.92
f1 (ppm)

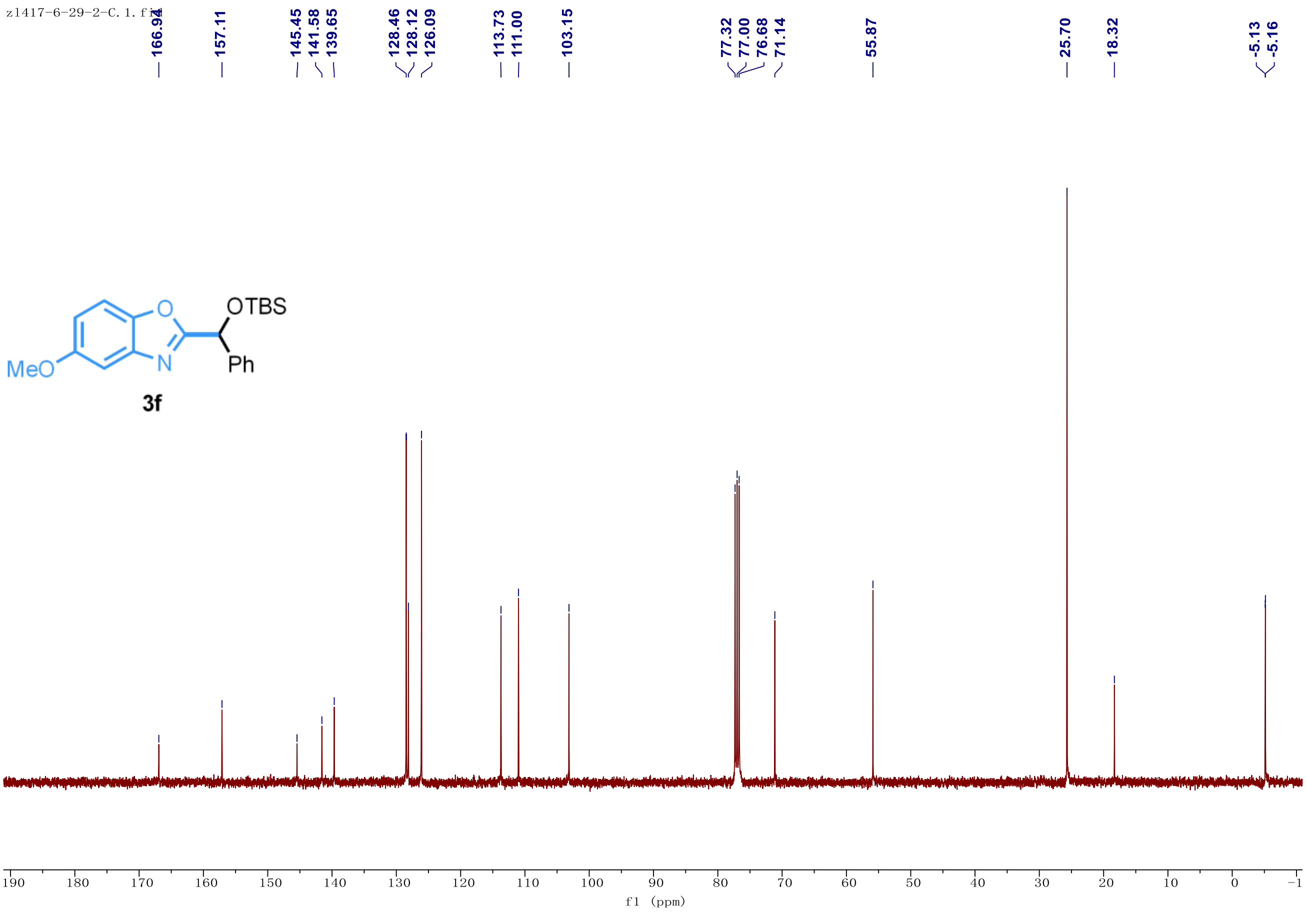

166.94
157.11
145.45
141.58
139.65
128.46
128.12
126.09
113.73
111.00
103.15
77.32
77.00
76.68
71.14
55.87
25.70
18.32
-5.13
-5.16
OTBS
MeO
O
N
Ph
3f
f1 (ppm)

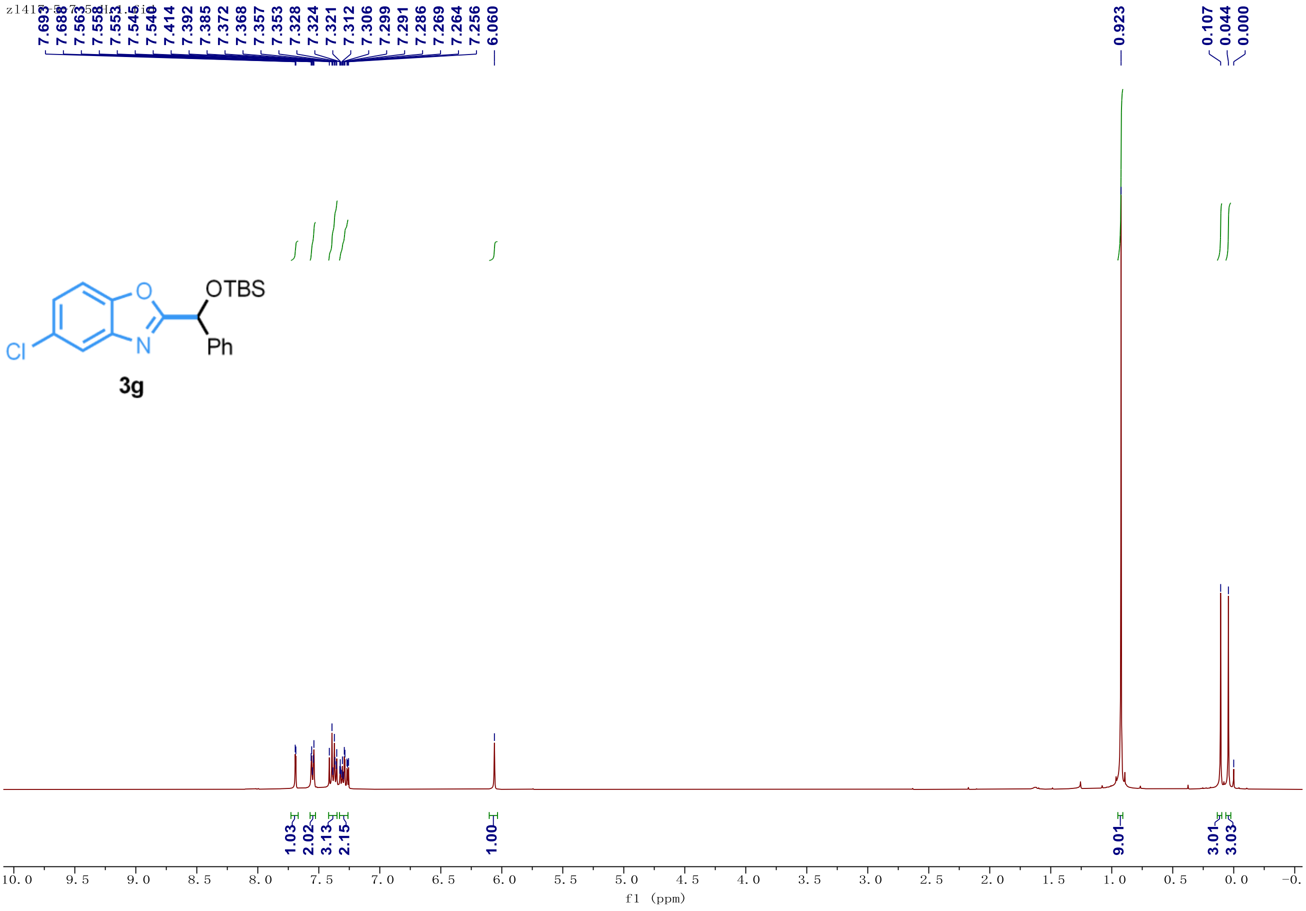

7.693
7.688
7.563
7.558
7.553
7.546
7.540
7.414
7.392
7.385
7.372
7.368
7.357
7.353
7.328
7.324
7.321
7.312
7.306
7.299
7.291
7.286
7.269
7.264
7.256
6.060
0.923
0.107
0.044
0.000
OTBS
Cl
O
N
Ph
3g
1.03
2.02
3.13
2.15
1.00
9.01
3.01
3.03
f1 (ppm)

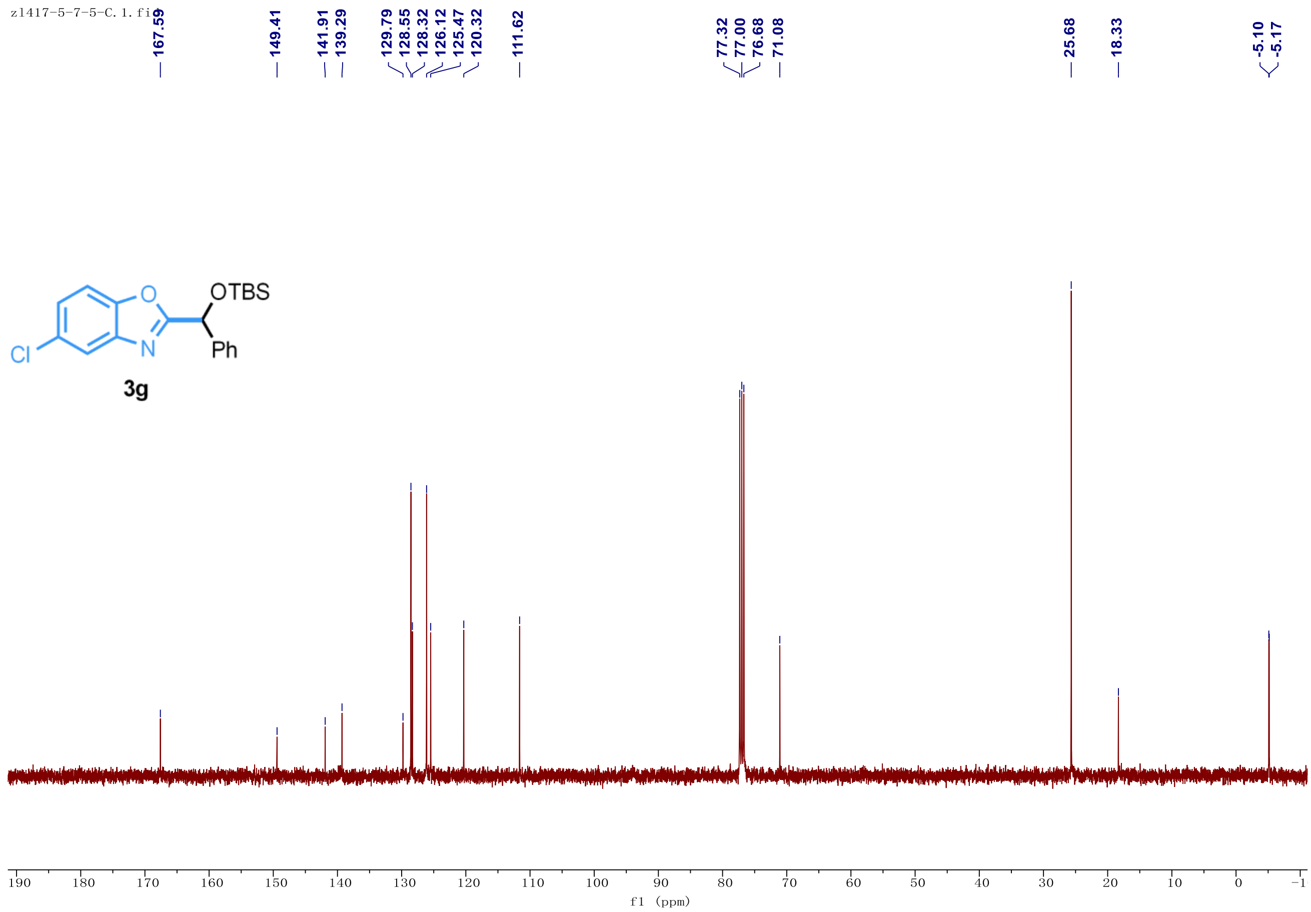
z1417-5-7-5-C.1.fid
167.59
149.41
141.91
139.29
129.79
128.55
128.32
126.12
125.47
120.32
111.62
77.32
77.00
76.68
71.08
25.68
18.33
-5.10
-5.17
OTBS
Cl
O
N
Ph
3g
f1 (ppm)

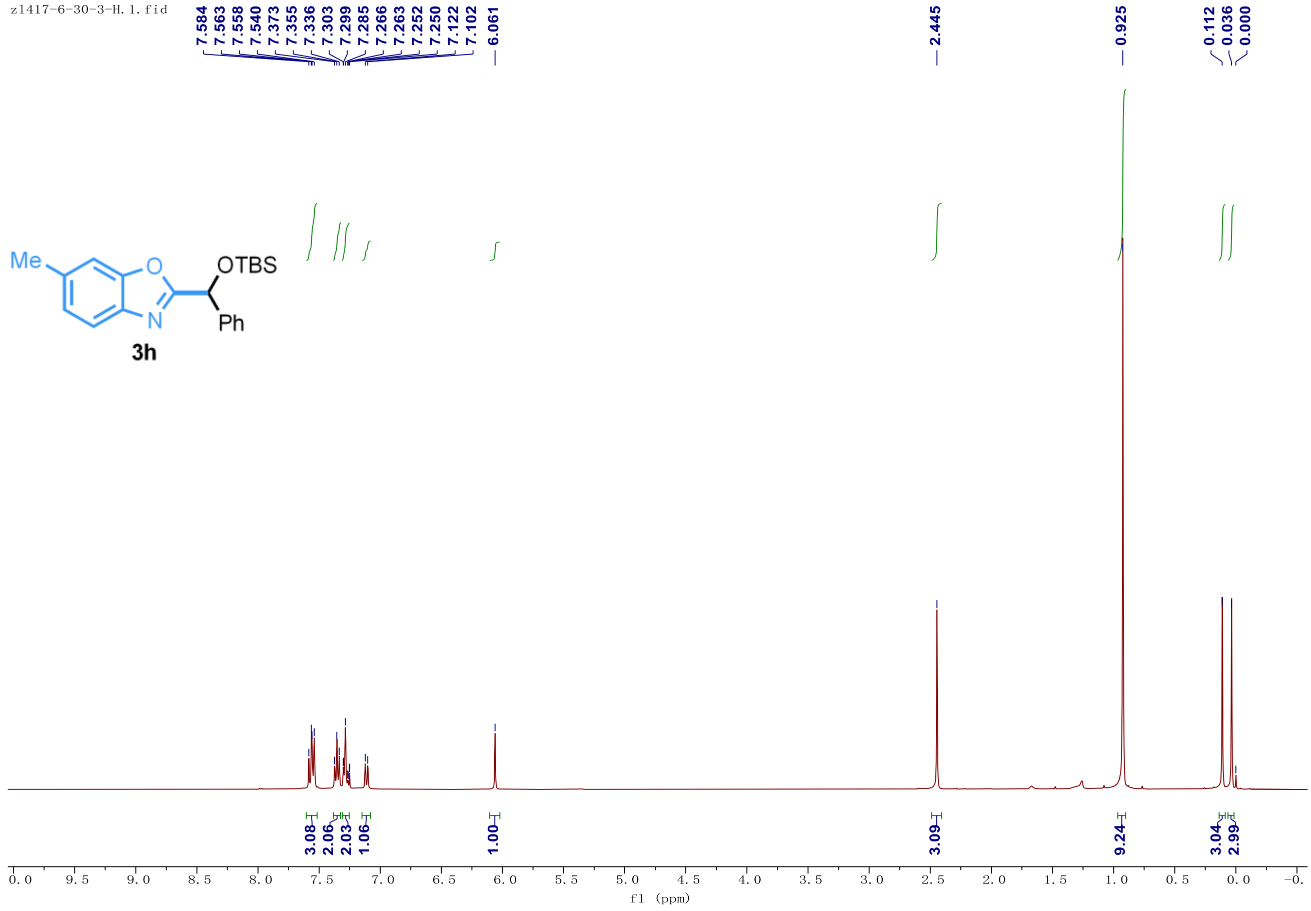
z1417-6-30-3-H.1.fid
7.584
7.563
7.558
7.540
7.373
7.355
7.336
7.303
7.299
7.285
7.266
7.263
7.252
7.250
7.122
7.102
6.061
2.445
0.925
0.112
0.036
0.000
Me
O
OTBS
N
Ph
3h
3.08
2.06
2.03
1.06
1.00
3.09
9.24
3.04
2.99
f1 (ppm)

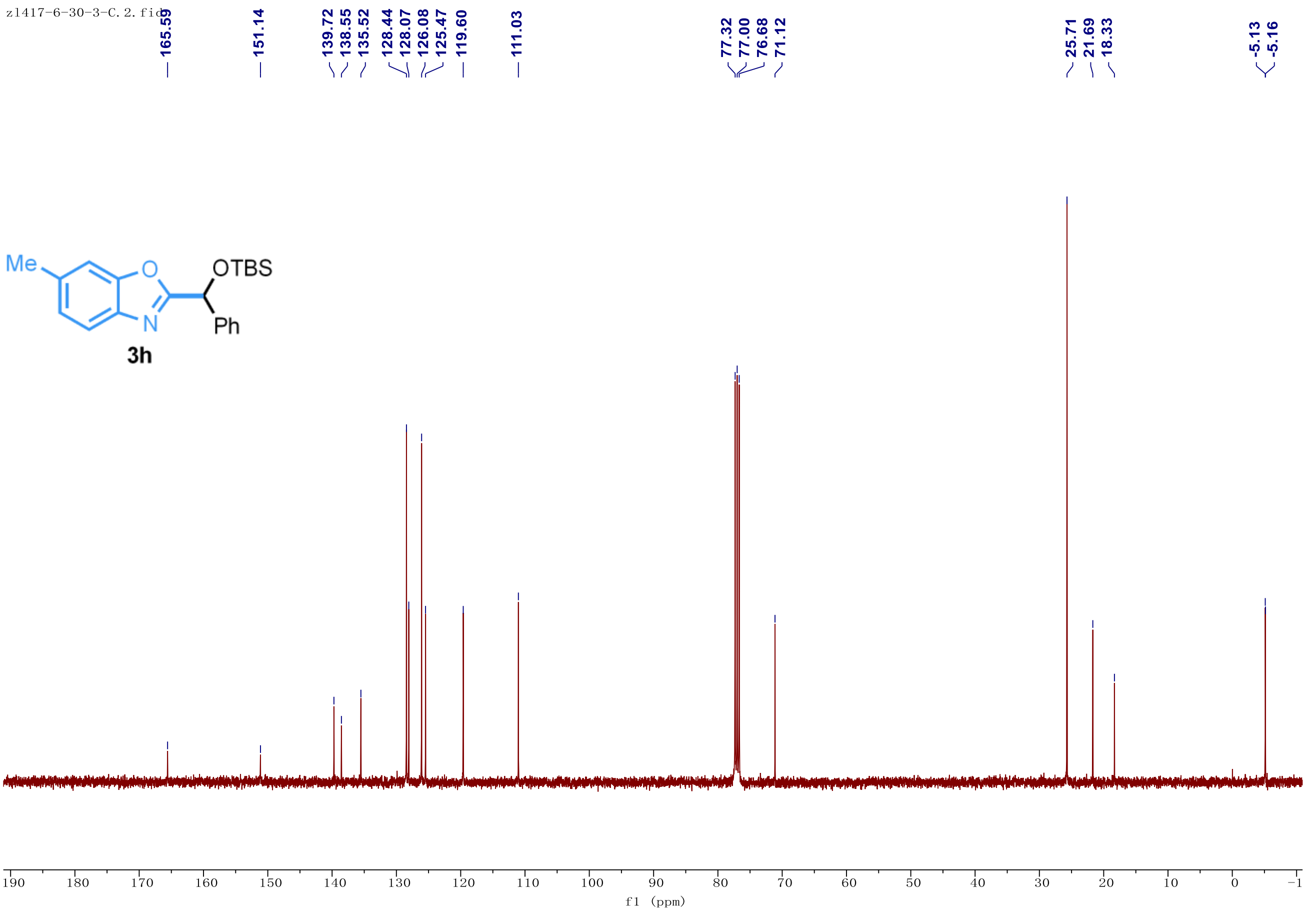

zl417-6-30-3-C.2.fid
165.59
151.14
139.72
138.55
135.52
128.44
128.07
126.08
125.47
119.60
111.03
77.32
77.00
76.68
71.12
25.71
21.69
18.33
-5.13
-5.16
Me
O
N
OTBS
Ph
3h
190 180 170 160 150 140 130 120 110 100 90 80 70 60 50 40 30 20 10 0 -1
f1 (ppm)

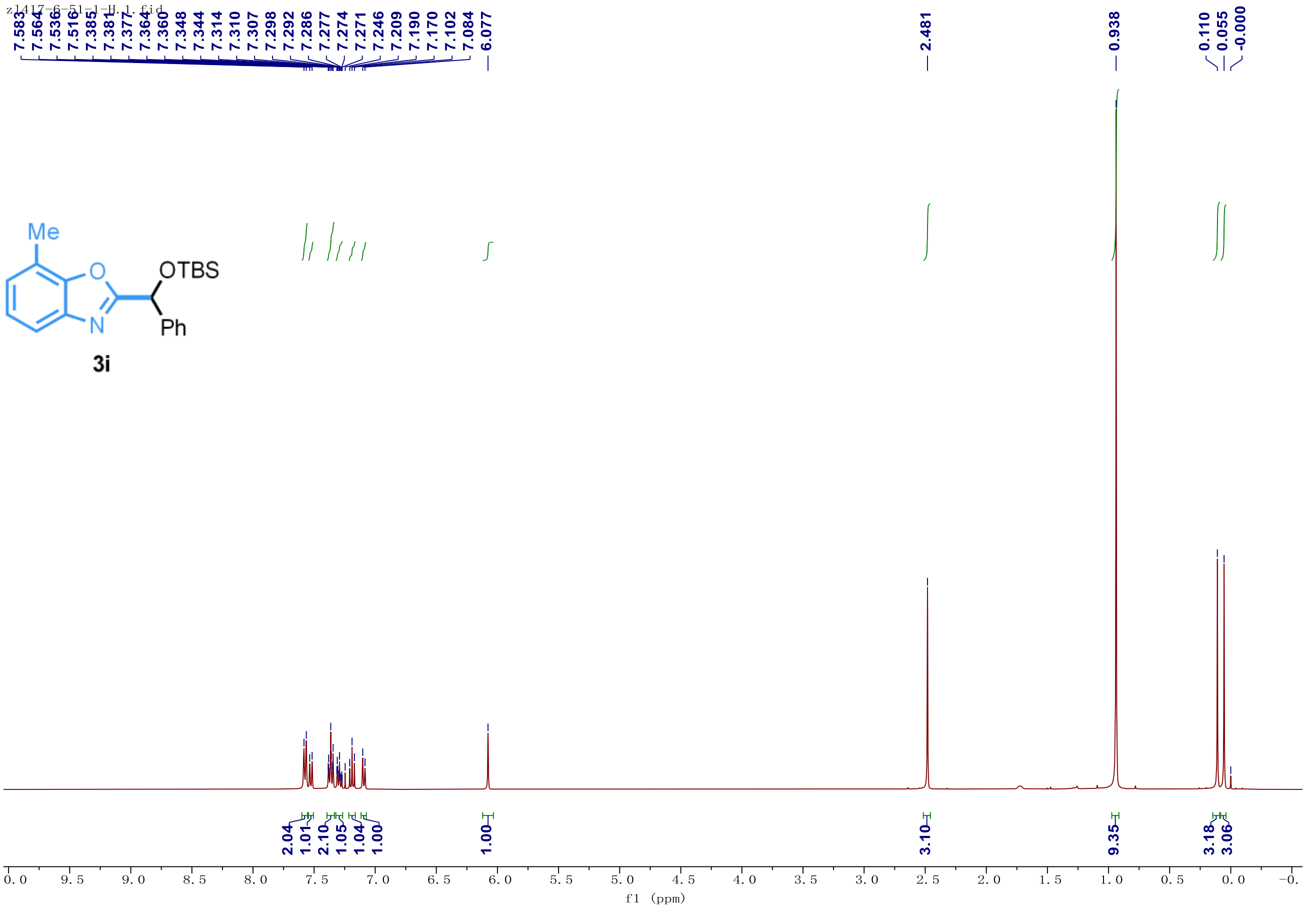

zl417-6-51-1-H.1.fid
7.583
7.564
7.536
7.516
7.385
7.381
7.377
7.364
7.360
7.348
7.344
7.314
7.310
7.307
7.298
7.292
7.286
7.277
7.274
7.271
7.246
7.209
7.190
7.170
7.102
7.084
6.077
2.481
0.938
0.110
0.055
-0.000
Me
O
N
OTBS
Ph
3i
2.04
1.01
2.10
1.05
1.04
1.00
1.00
3.10
9.35
3.18
3.06
0.0 9.5 9.0 8.5 8.0 7.5 7.0 6.5 6.0 5.5 5.0 4.5 4.0 3.5 3.0 2.5 2.0 1.5 1.0 0.5 0.0 -0.
f1 (ppm)

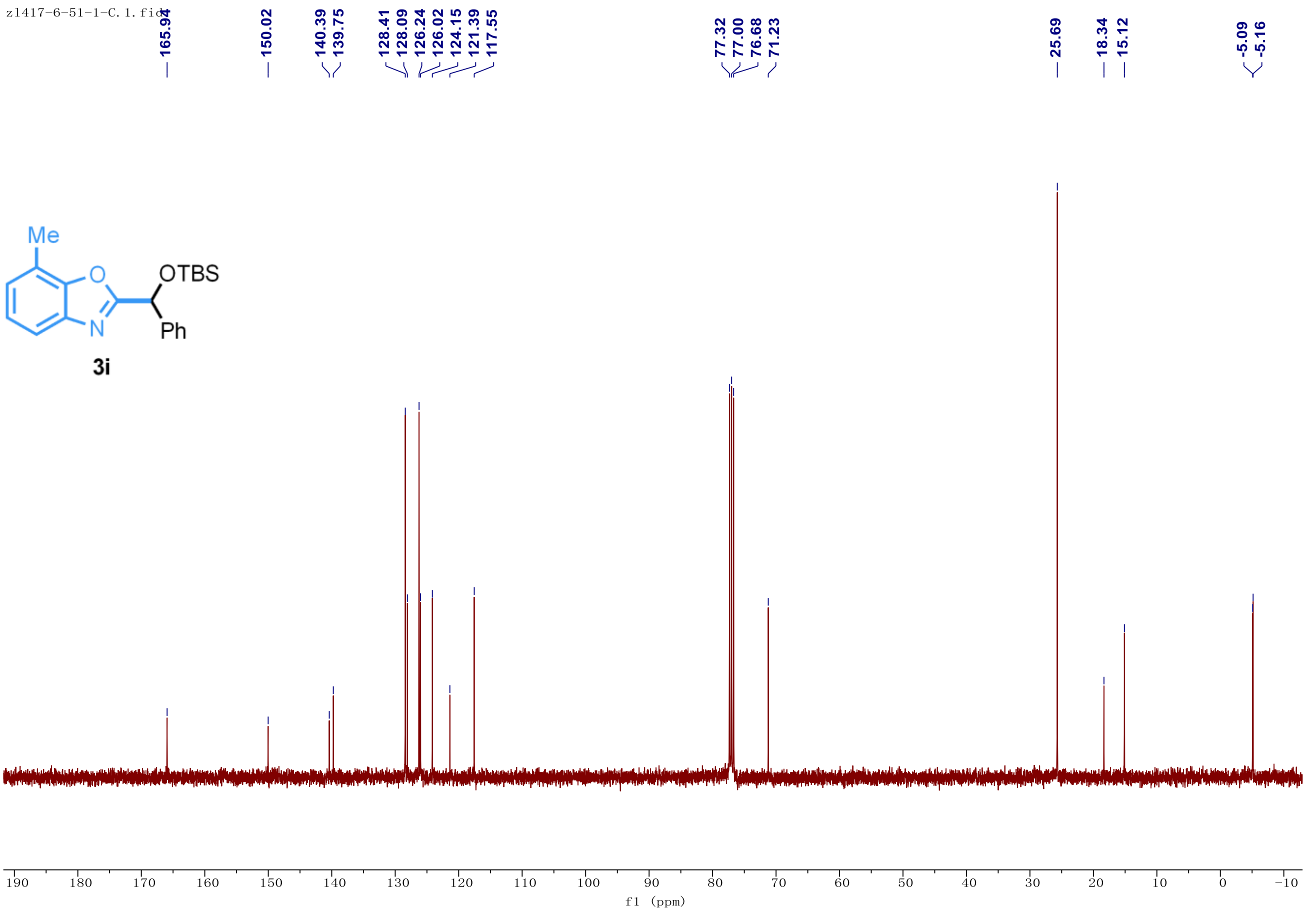
z1417-6-51-1-C.1.fid
165.94
150.02
140.39
139.75
128.41
128.09
126.24
126.02
124.15
121.39
117.55
77.32
77.00
76.68
71.23
25.69
18.34
15.12
-5.09
-5.16
Me
OTBS
O
N
Ph
3i
190 180 170 160 150 140 130 120 110 100 90 80 70 60 50 40 30 20 10 0 -10
f1 (ppm)

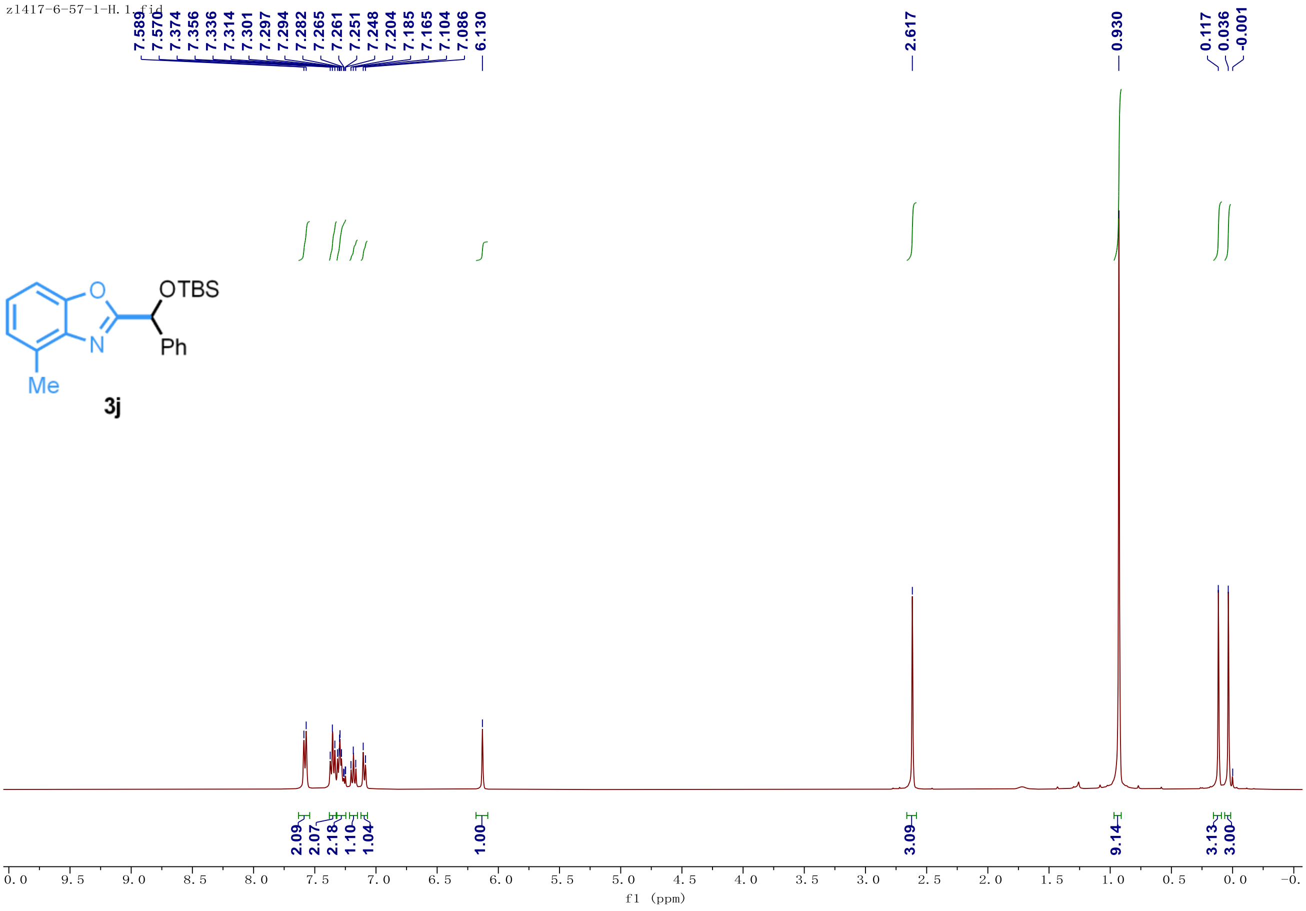
z1417-6-57-1-H.1.fid
7.589
7.570
7.374
7.356
7.336
7.314
7.301
7.297
7.294
7.282
7.265
7.261
7.251
7.248
7.204
7.185
7.165
7.104
7.086
6.130
2.617
0.930
0.117
0.036
-0.001
OTBS
O
N
Ph
Me
3j
2.09
2.07
2.18
1.10
1.04
1.00
3.09
9.14
3.13
3.00
f1 (ppm)

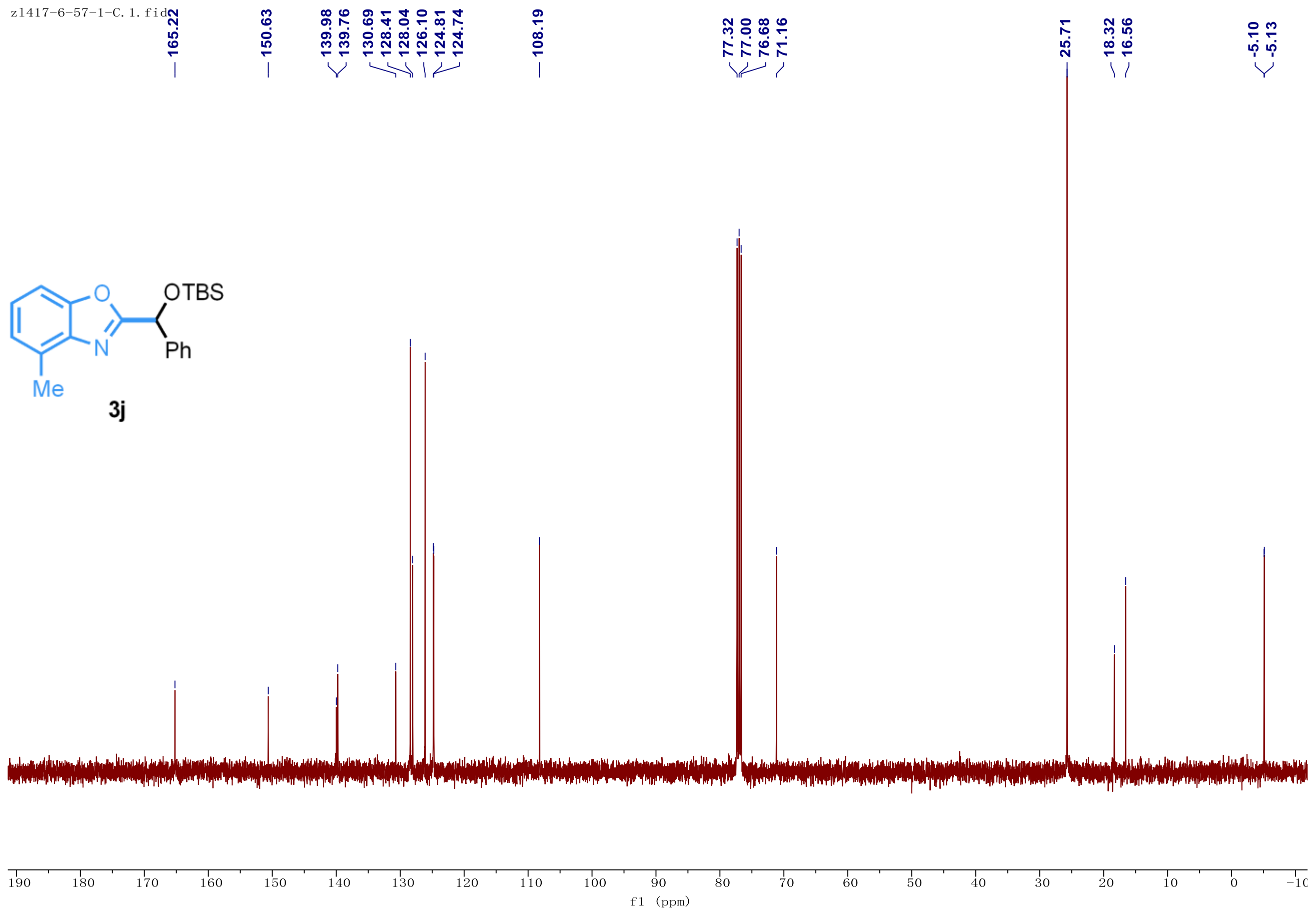
z1417-6-57-1-C.1.fid
165.22
150.63
139.98
139.76
130.69
128.41
128.04
126.10
124.81
124.74
108.19
77.32
77.00
76.68
71.16
25.71
18.32
16.56
-5.10
-5.13
OTBS
Ph
N
O
Me
3j
f1 (ppm)

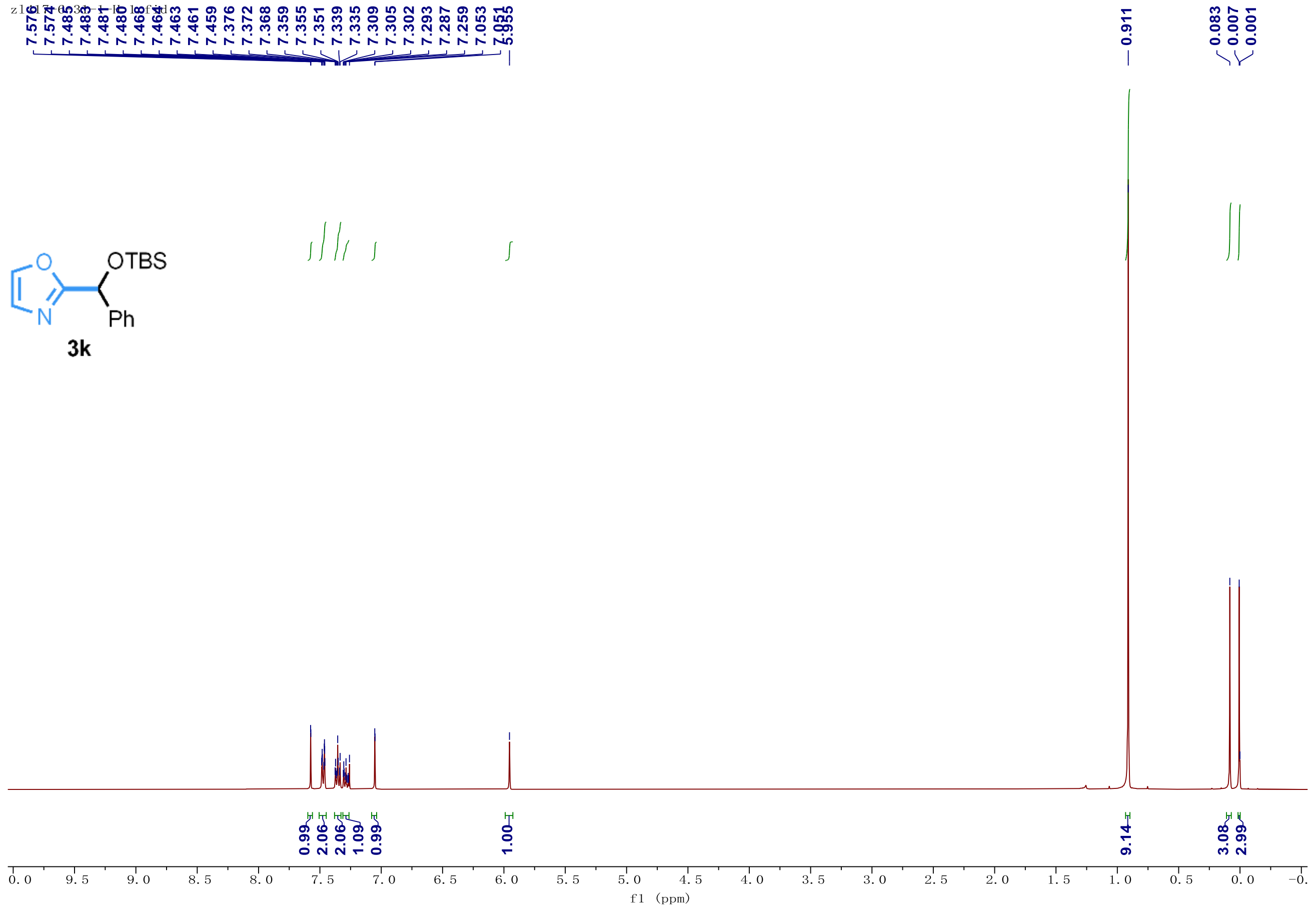
0.911
0.083
0.007
0.001
OTBS
Ph
N
O
3k
0.99
2.06
2.06
1.09
0.99
1.00
9.14
3.08
2.99
f1 (ppm)

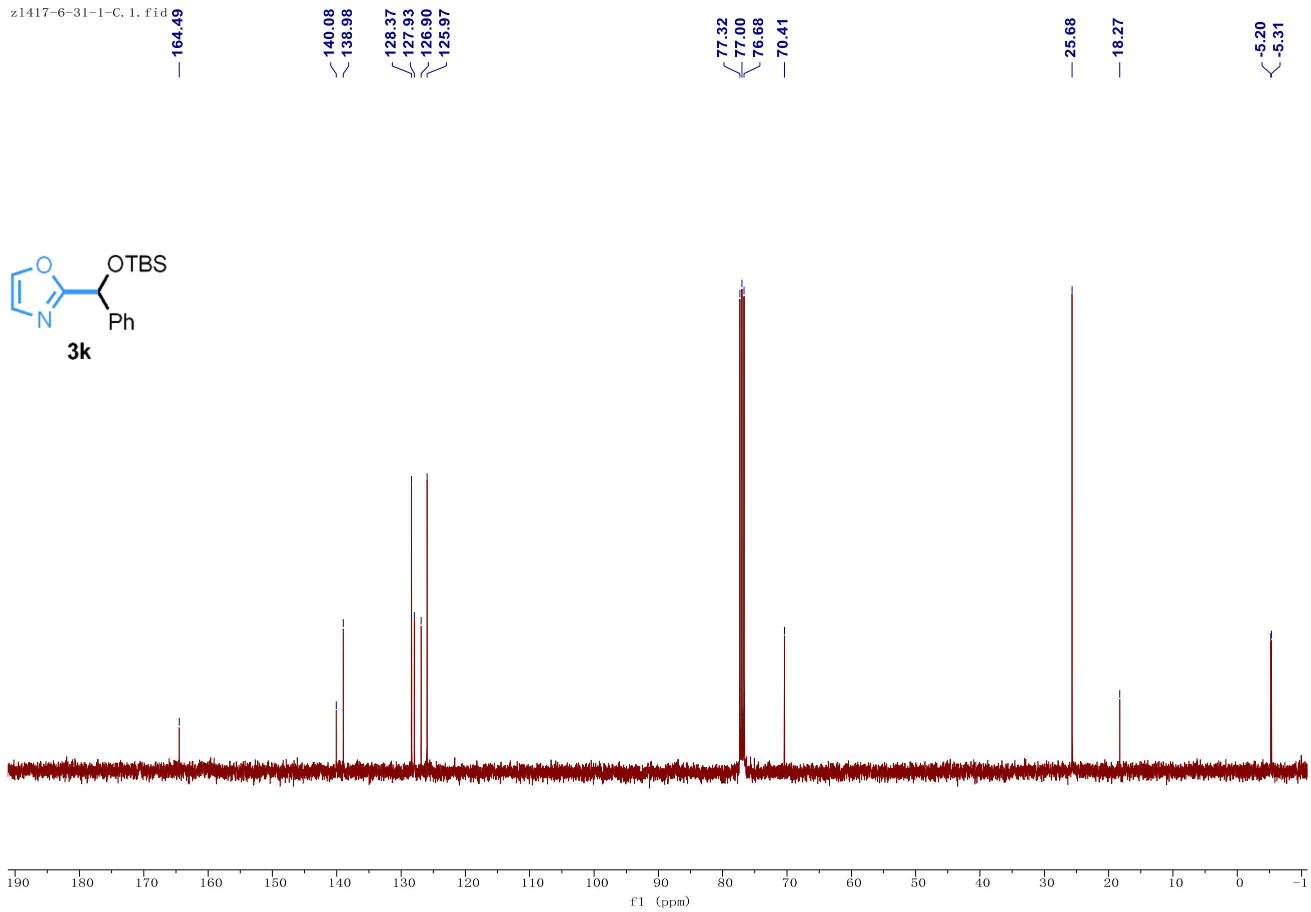

z1417-6-31-1-C.1.fid
164.49
140.08
138.98
128.37
127.93
126.90
125.97
77.32
77.00
76.68
70.41
25.68
18.27
-5.20
-5.31
OTBS
O
N
Ph
3k
190 180 170 160 150 140 130 120 110 100 90 80 70 60 50 40 30 20 10 0 -1
f1 (ppm)

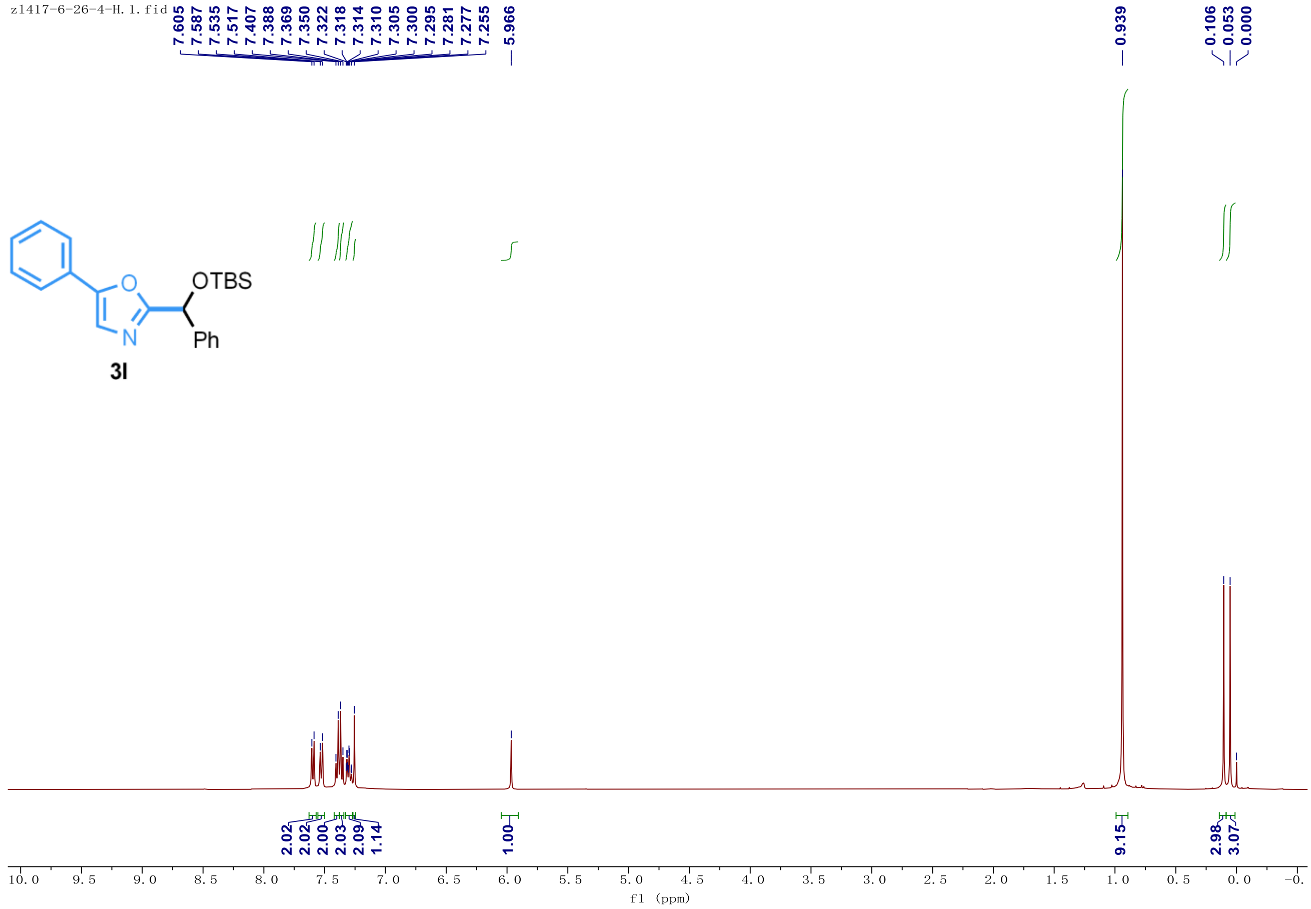

z1417-6-26-4-H.1.fid
7.605
7.587
7.535
7.517
7.407
7.388
7.369
7.350
7.322
7.318
7.314
7.310
7.305
7.300
7.295
7.281
7.277
7.255
5.966
0.939
0.106
0.053
0.000
OTBS
O
N
Ph
3l
2.02
2.02
2.00
2.03
2.09
1.14
1.00
9.15
2.98
3.07
10.0 9.5 9.0 8.5 8.0 7.5 7.0 6.5 6.0 5.5 5.0 4.5 4.0 3.5 3.0 2.5 2.0 1.5 1.0 0.5 0.0 -0.
f1 (ppm)

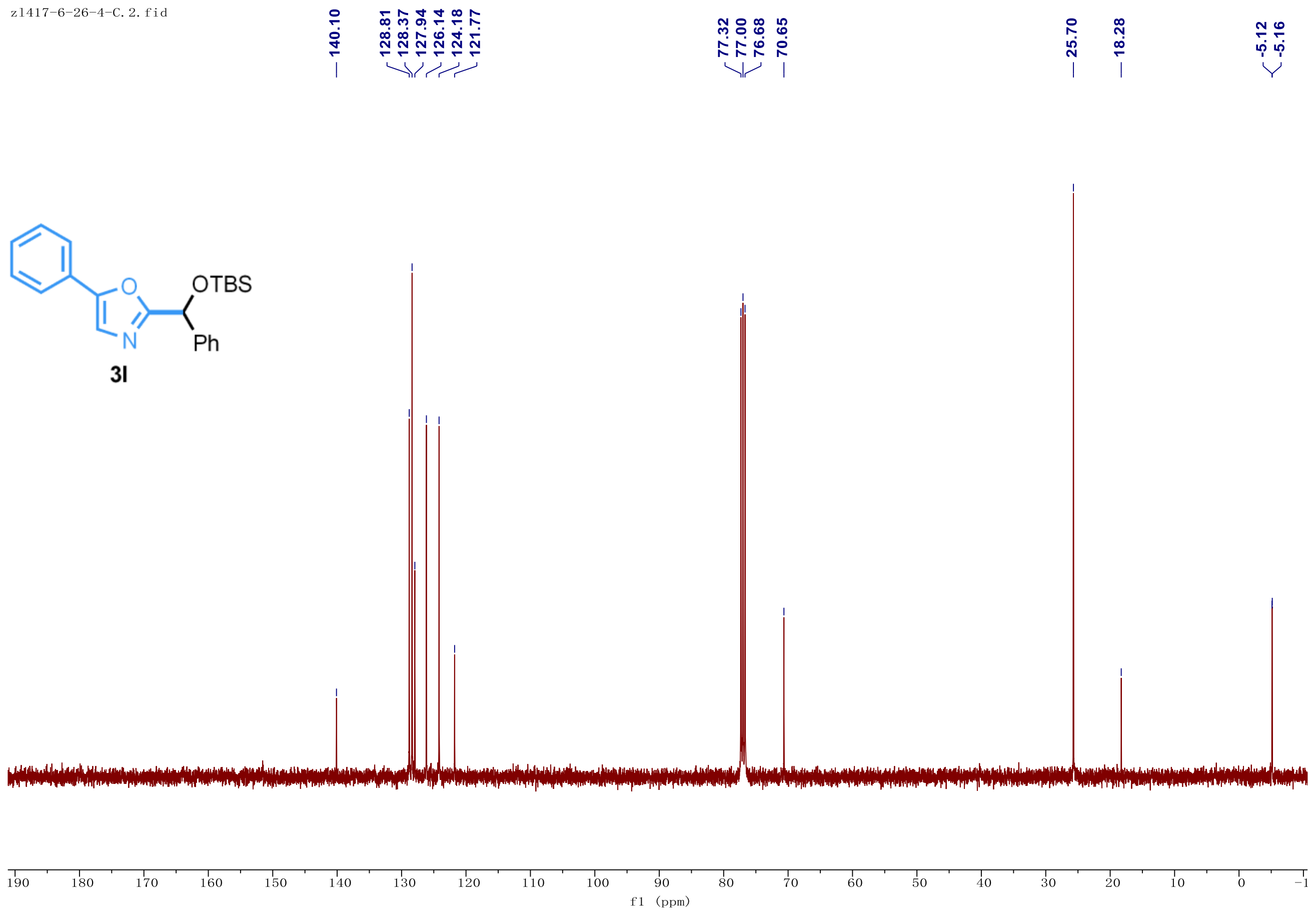
zl417-6-26-4-C.2.fid
140.10
128.81
128.37
127.94
126.14
124.18
121.77
77.32
77.00
76.68
70.65
25.70
18.28
-5.12
-5.16
OTBS
O
N
Ph
3l
f1 (ppm)

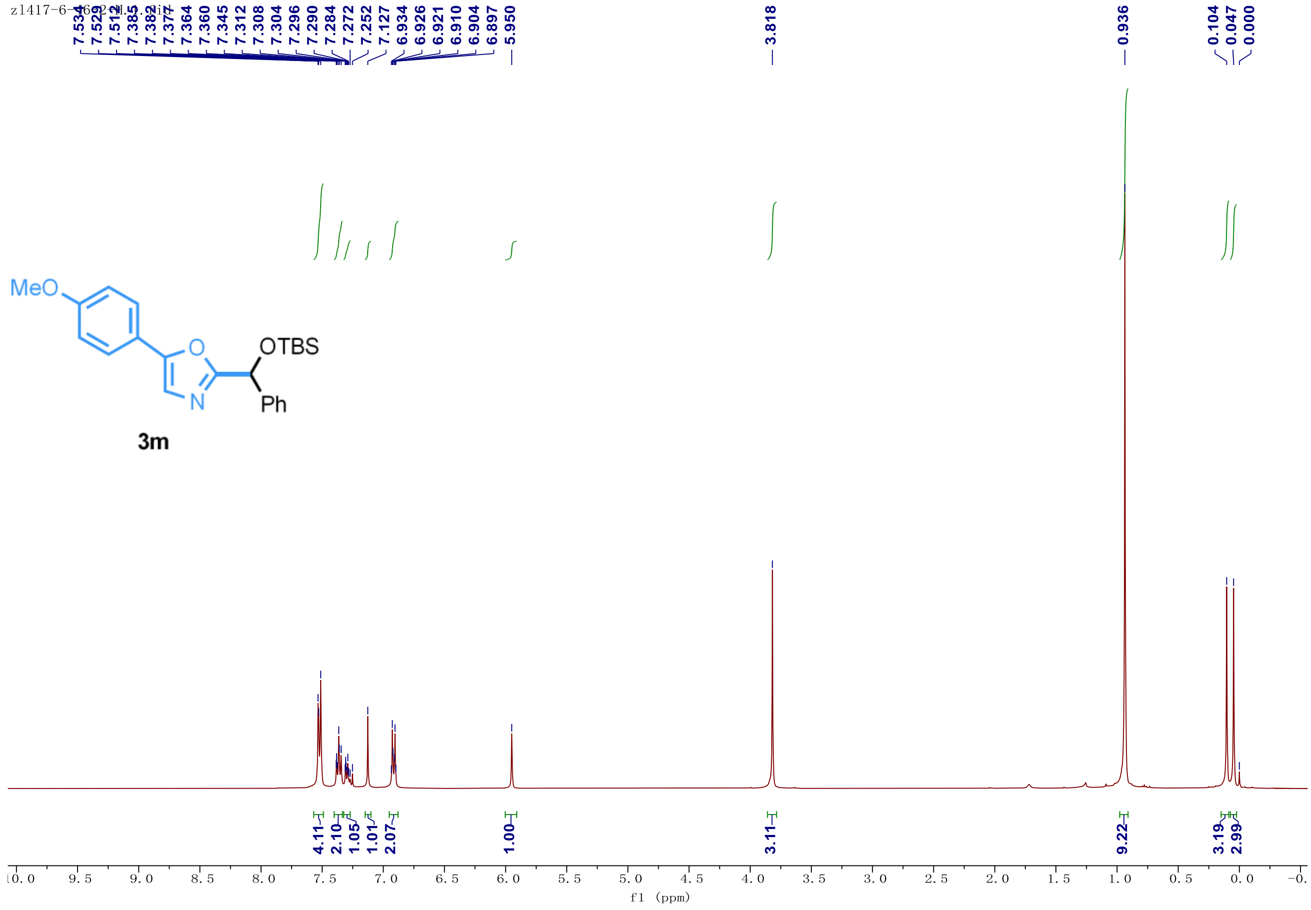
7.534
7.529
7.512
7.385
7.382
7.377
7.364
7.360
7.345
7.312
7.308
7.304
7.296
7.290
7.284
7.272
7.252
7.127
6.934
6.926
6.921
6.910
6.904
6.897
5.950
3.818
0.936
0.104
0.047
0.000
MeO
O
N
OTBS
Ph
3m
4.11
2.10
1.05
1.01
2.07
1.00
3.11
9.22
3.19
2.99
f1 (ppm)

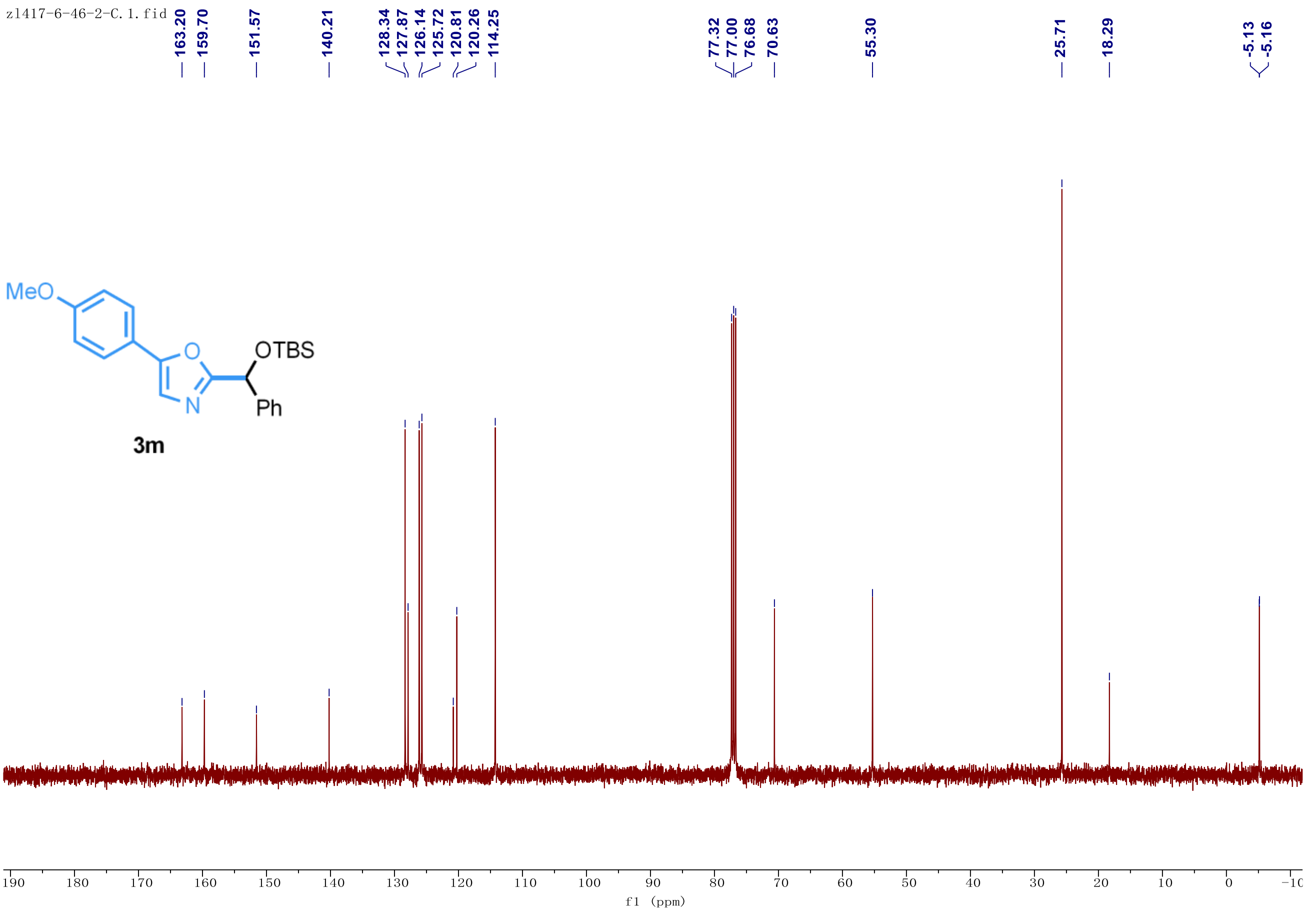
zl417-6-46-2-C.1.fid
163.20
159.70
151.57
140.21
128.34
127.87
126.14
125.72
120.81
120.26
114.25
77.32
77.00
76.68
70.63
55.30
25.71
18.29
-5.13
-5.16
MeO
O
N
OTBS
Ph
3m
190 180 170 160 150 140 130 120 110 100 90 80 70 60 50 40 30 20 10 0 -10
f1 (ppm)

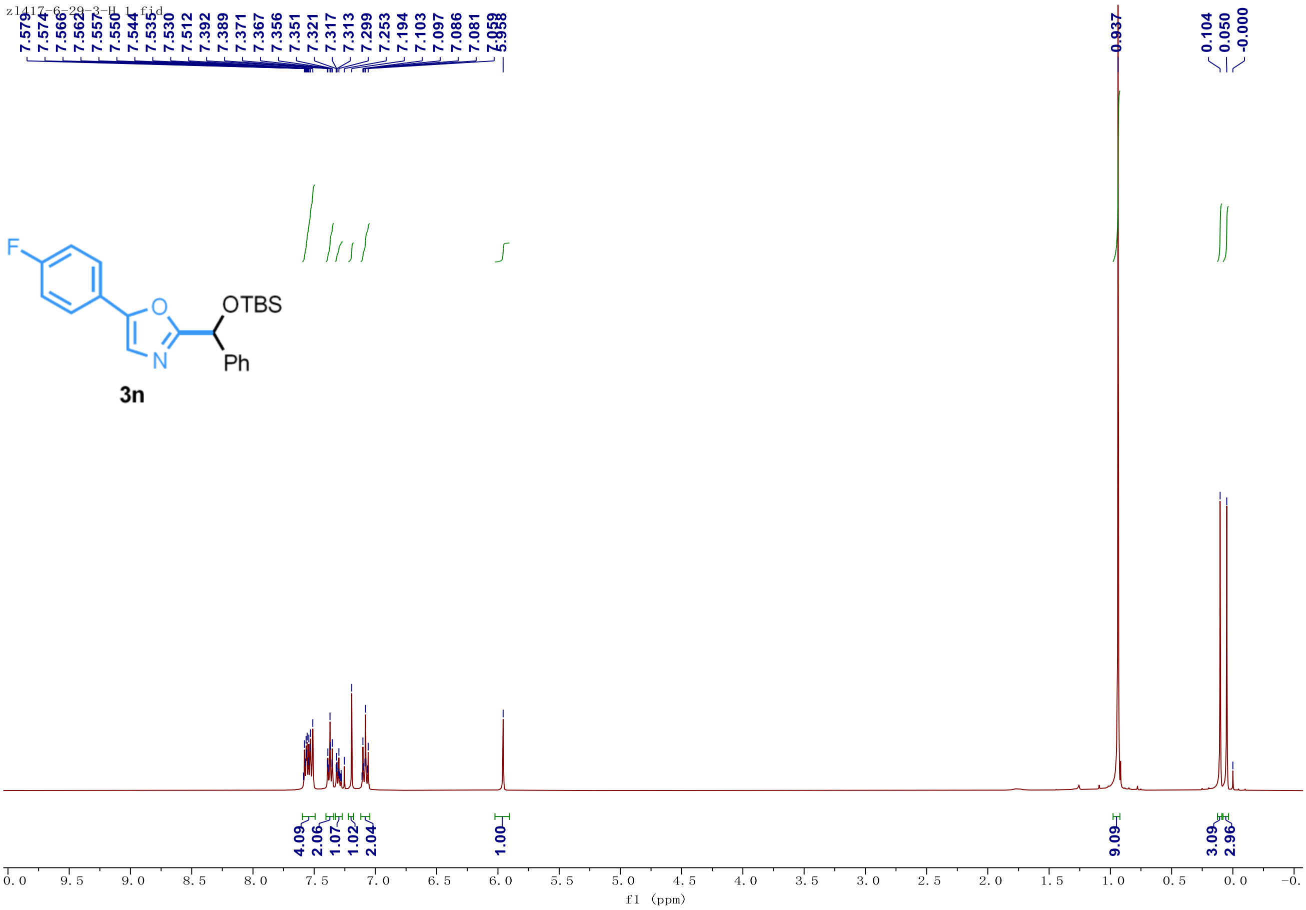
zl417-6-29-3-H.1.fid
7.579
7.574
7.566
7.562
7.557
7.550
7.544
7.535
7.530
7.512
7.392
7.389
7.371
7.367
7.356
7.351
7.321
7.317
7.313
7.299
7.253
7.194
7.103
7.097
7.086
7.081
7.059
5.958
0.937
0.104
0.050
-0.000
F
O
N
OTBS
Ph
3n
4.09
2.06
1.07
1.02
2.04
1.00
9.09
3.09
2.96
10.0 9.5 9.0 8.5 8.0 7.5 7.0 6.5 6.0 5.5 5.0 4.5 4.0 3.5 3.0 2.5 2.0 1.5 1.0 0.5 0.0 -0.
f1 (ppm)

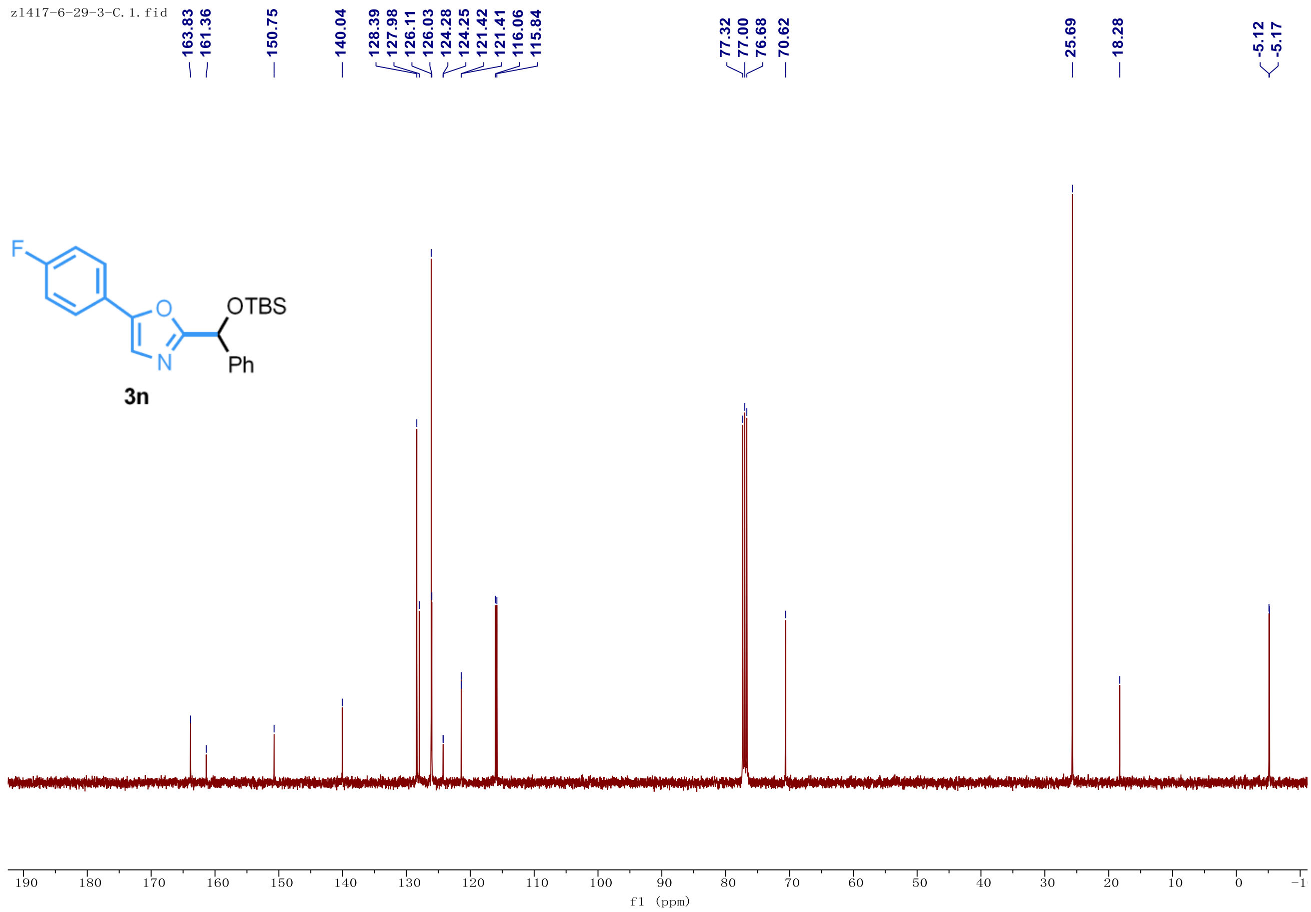

zl417-6-29-3-C.1.fid
163.83
161.36
150.75
140.04
128.39
127.98
126.11
126.03
124.28
124.25
121.42
121.41
116.06
115.84
77.32
77.00
76.68
70.62
25.69
18.28
-5.12
-5.17
F
O
OTBS
N
Ph
3n
f1 (ppm)

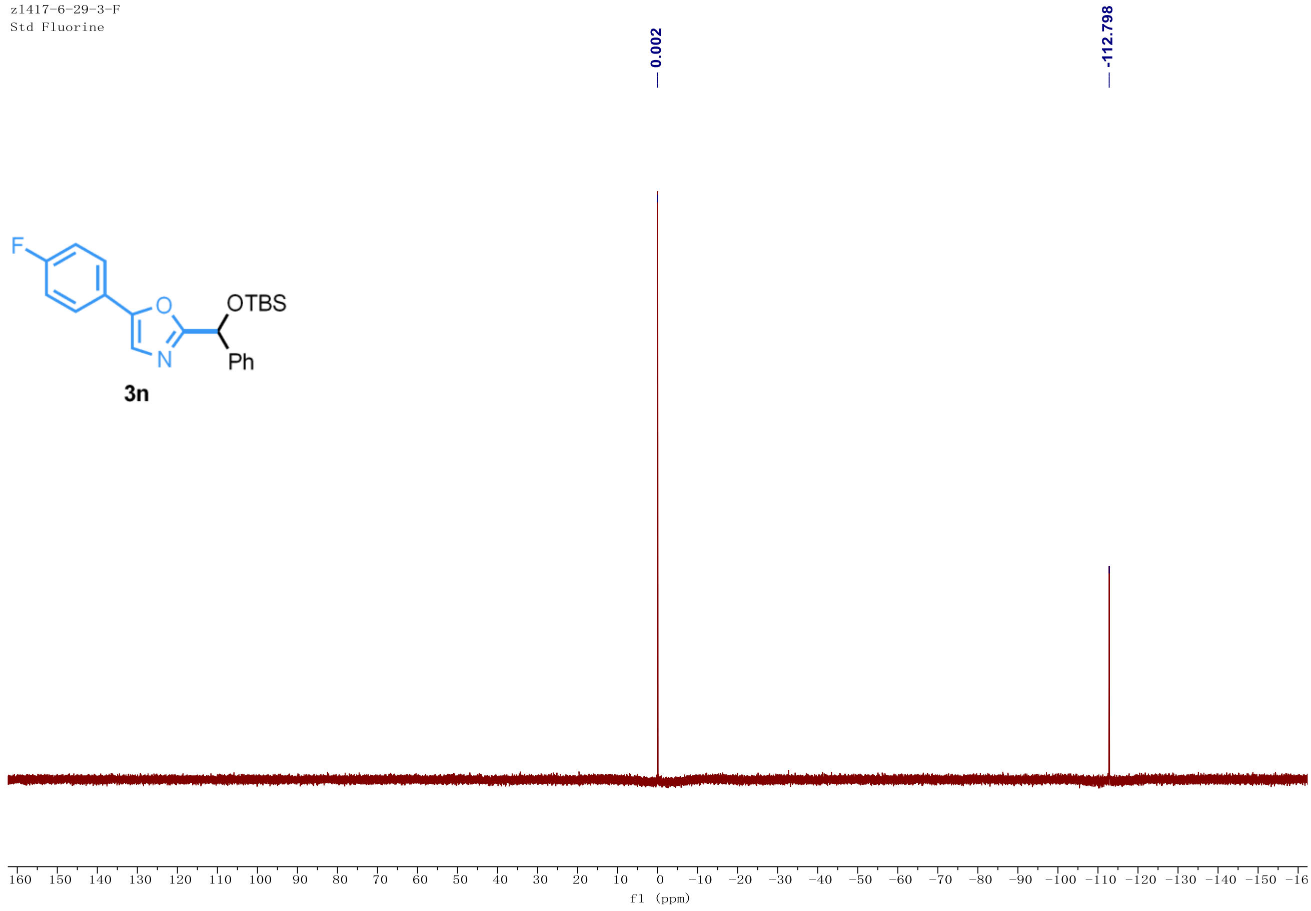

zl417-6-29-3-F
Std Fluorine
0.002
-112.798
F
O
OTBS
N
Ph
3n
f1 (ppm)

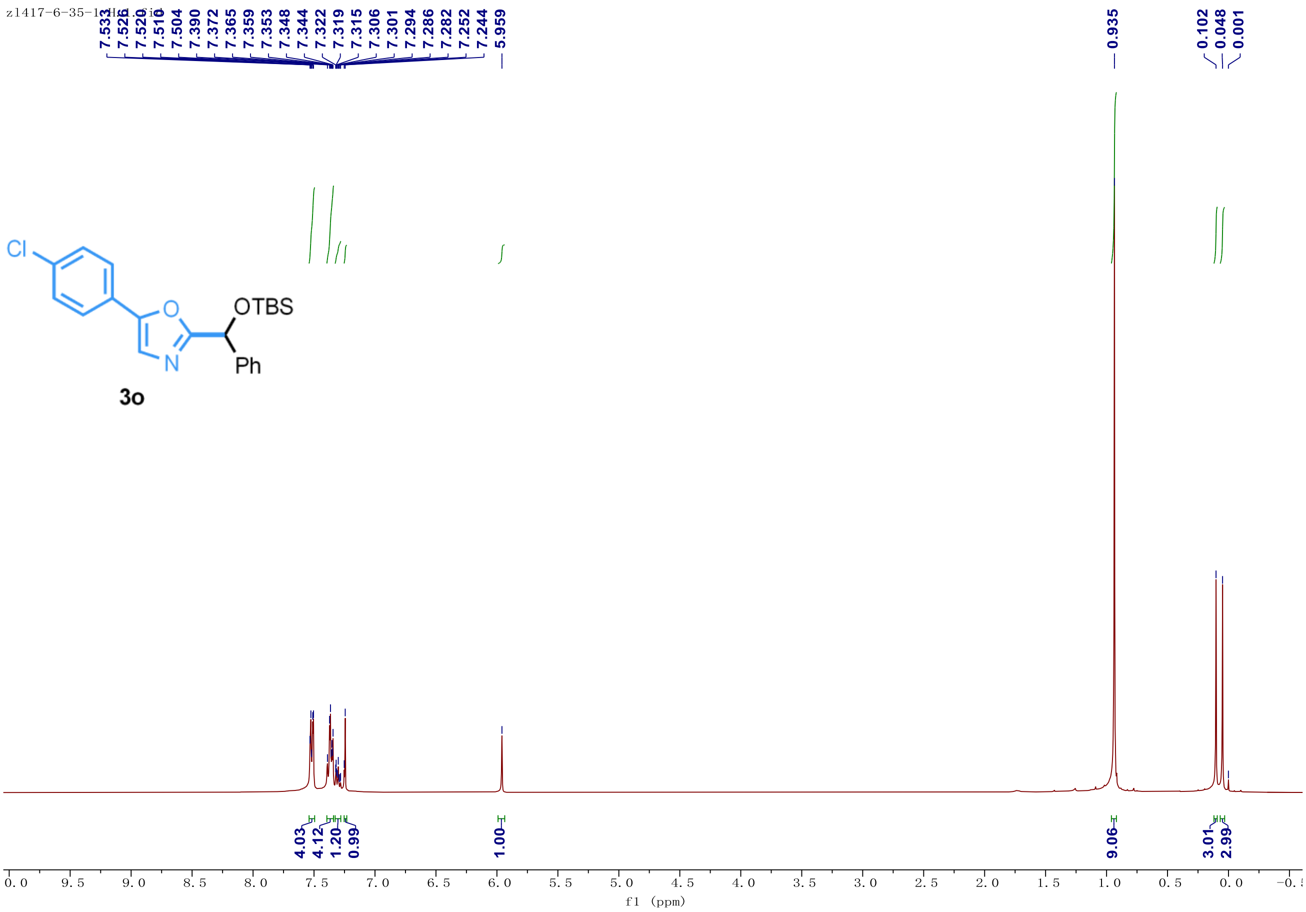
z1417-6-35-1H.fid
7.533
7.526
7.520
7.510
7.504
7.390
7.372
7.365
7.359
7.353
7.348
7.344
7.322
7.319
7.315
7.306
7.301
7.294
7.286
7.282
7.252
7.244
5.959
0.935
0.102
0.048
0.001
Cl
O
OTBS
N
Ph
3o
4.03
4.12
1.20
0.99
1.00
9.06
3.01
2.99
f1 (ppm)

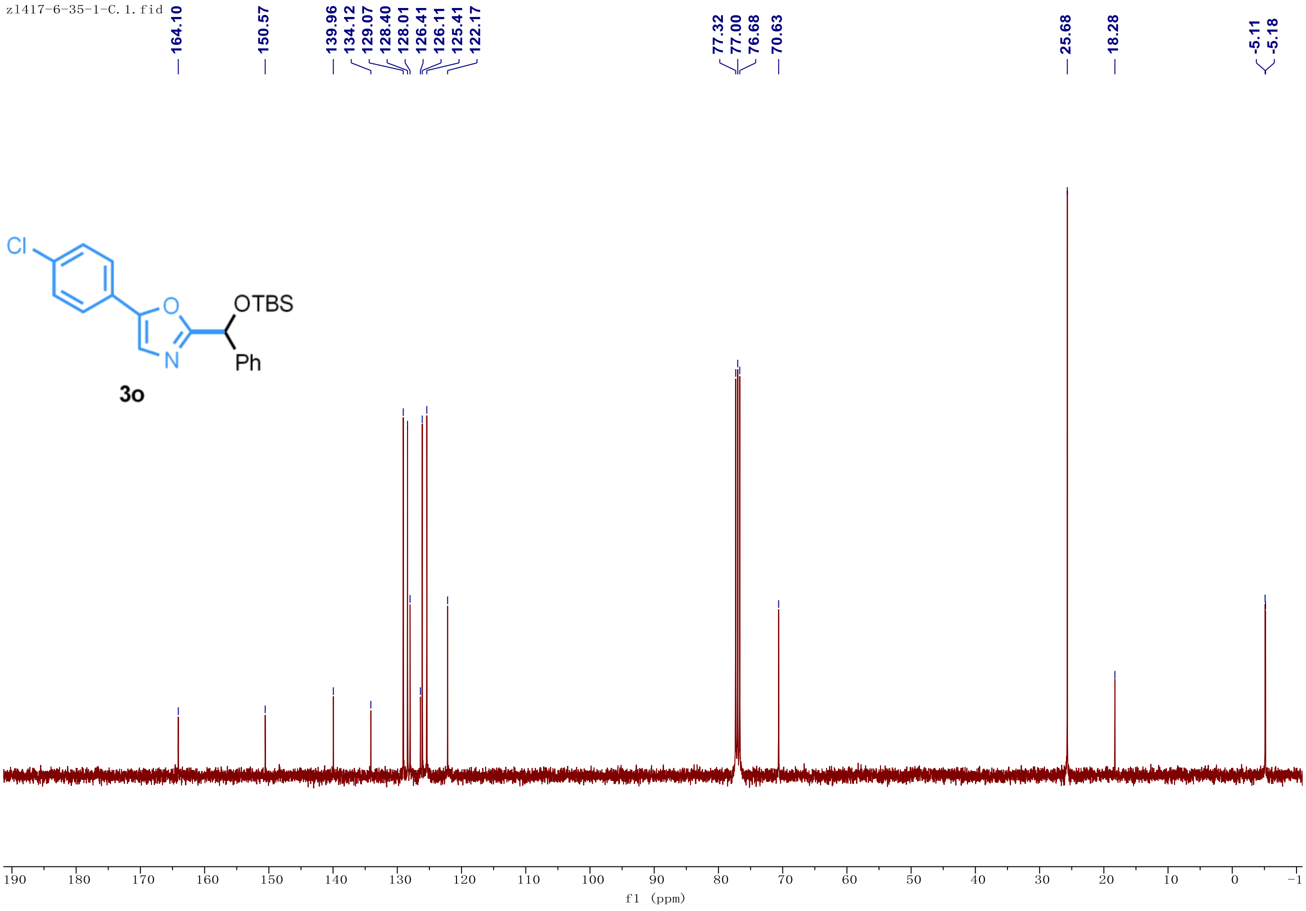
z1417-6-35-1-C.1.fid
164.10
150.57
139.96
134.12
129.07
128.40
128.01
126.41
126.11
125.41
122.17
77.32
77.00
76.68
70.63
25.68
18.28
-5.11
-5.18
Cl
O
OTBS
N
Ph
3o
f1 (ppm)

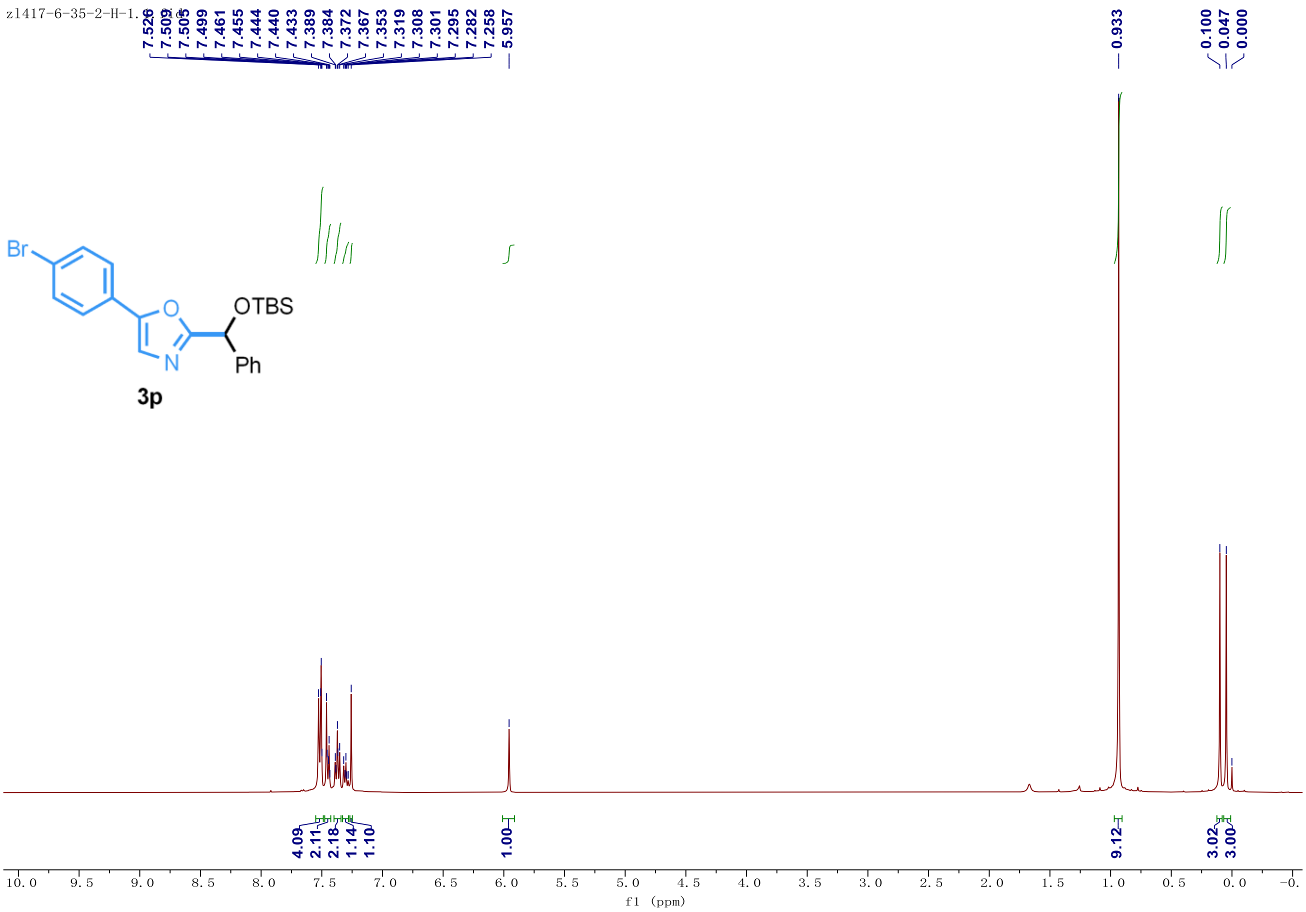
z1417-6-35-2-H-1.
7.526
7.509
7.505
7.499
7.461
7.455
7.444
7.440
7.433
7.389
7.384
7.372
7.367
7.353
7.319
7.308
7.301
7.295
7.282
7.258
5.957
0.933
0.100
0.047
0.000
Br
O
OTBS
N
Ph
3p
4.09
2.11
2.18
1.14
1.10
1.00
9.12
3.02
3.00
f1 (ppm)

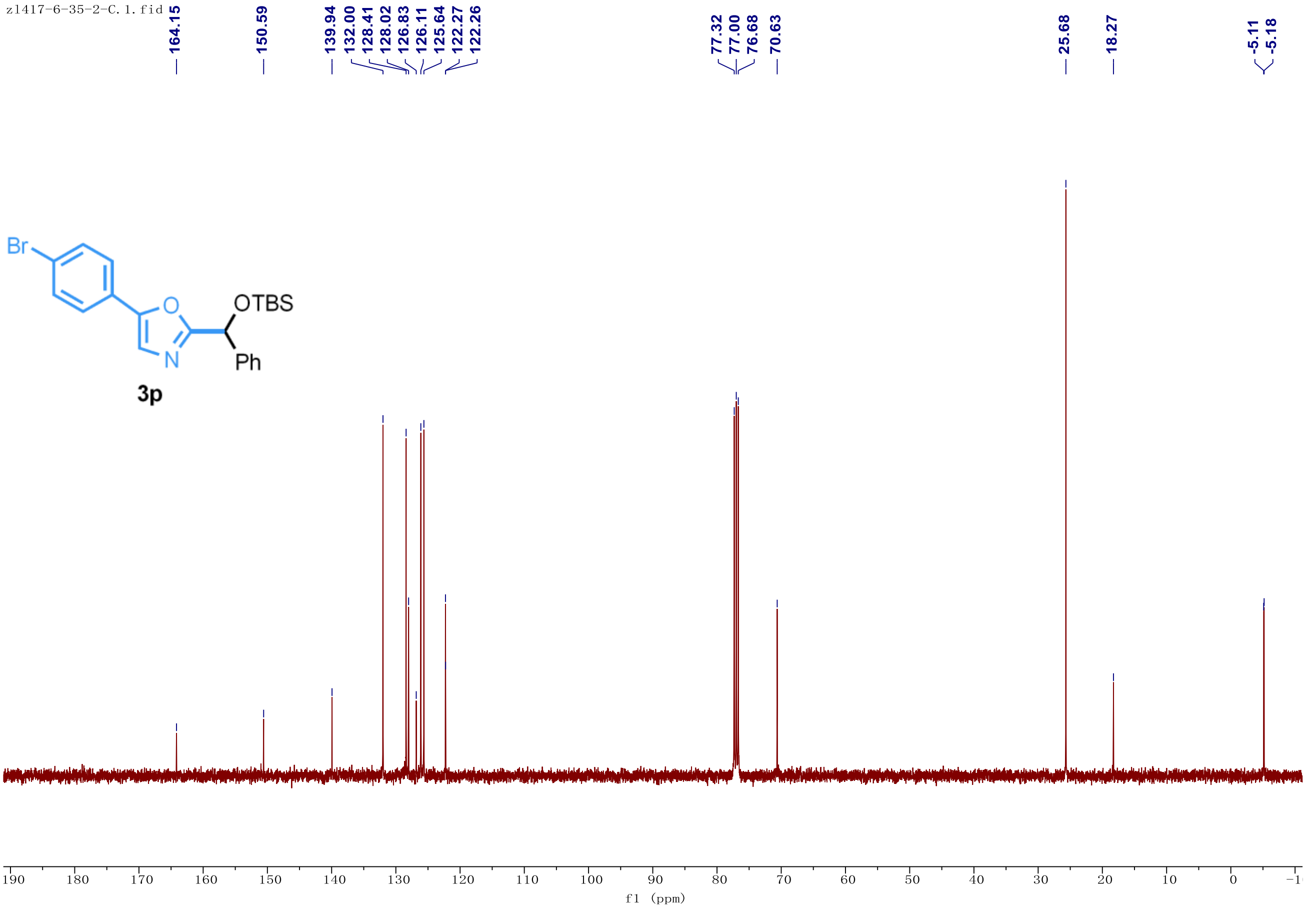
z1417-6-35-2-C.1.fid
164.15
150.59
139.94
132.00
128.41
128.02
126.83
126.11
125.64
122.27
122.26
77.32
77.00
76.68
70.63
25.68
18.27
-5.11
-5.18
Br
O
OTBS
N
Ph
3p
f1 (ppm)

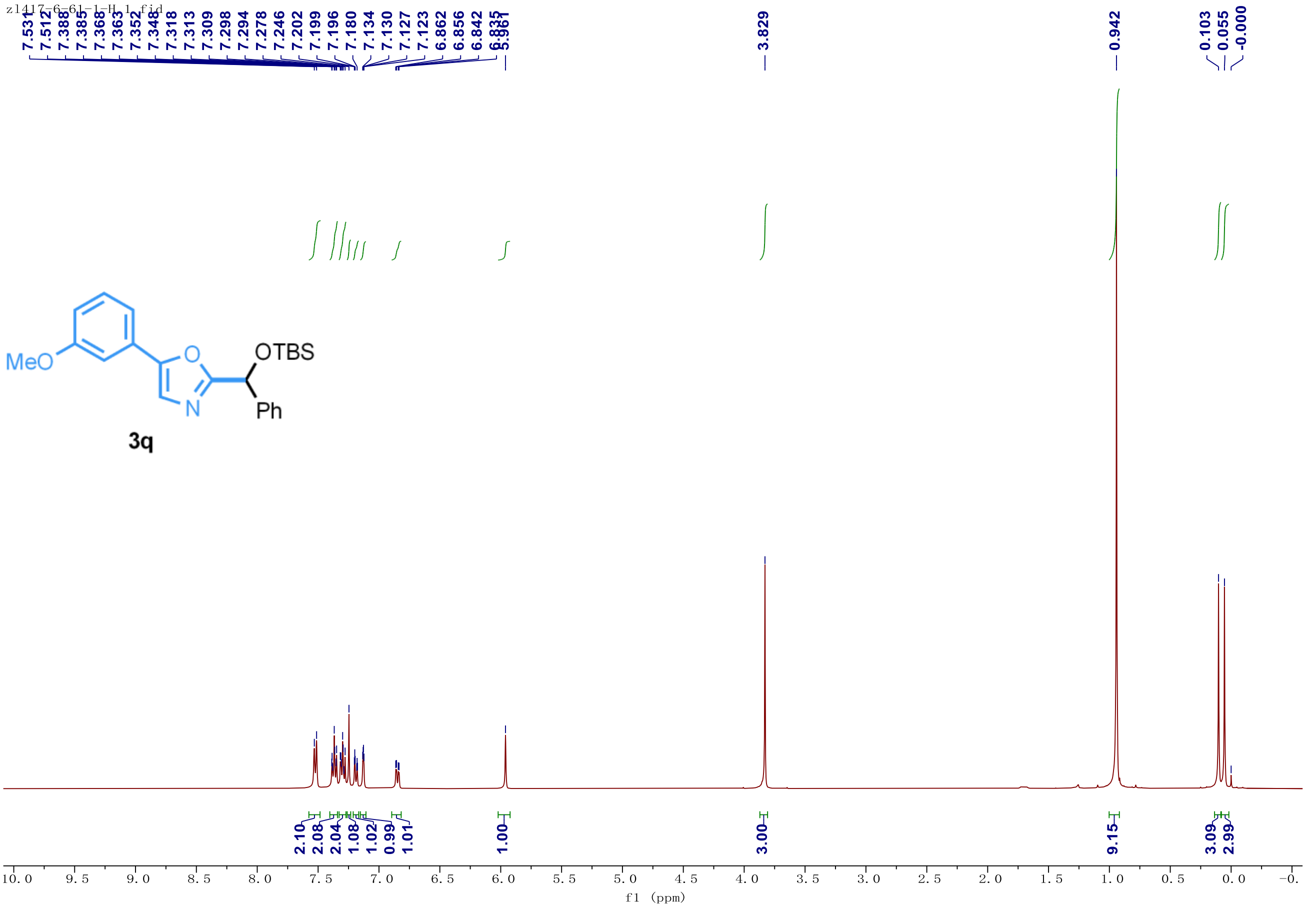
z1417-6-61-1-H.1.fid
7.531 7.512 7.388 7.385 7.368 7.363 7.352 7.348 7.318 7.313 7.309 7.298 7.294 7.278 7.246 7.202 7.199 7.196 7.180 7.134 7.130 7.127 7.123 6.862 6.856 6.842 6.835 5.961
3.829
0.942
0.103 0.055 -0.000
MeO
O
N
OTBS
Ph
3q
2.10 2.08 2.04 1.08 1.02 0.99 1.01
1.00
3.00
9.15
3.09 2.99
f1 (ppm)

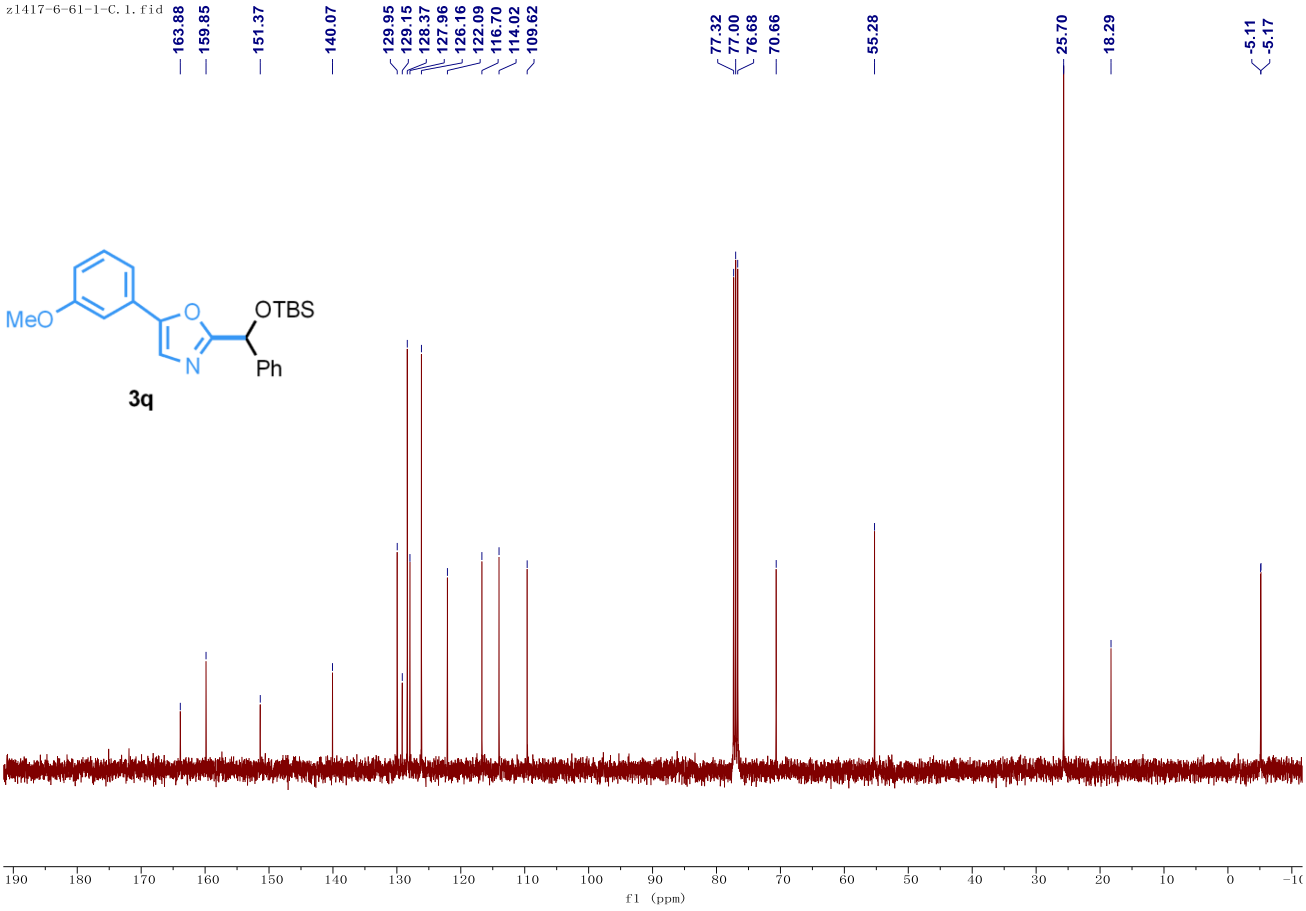
z1417-6-61-1-C.1.fid
163.88 159.85 151.37 140.07 129.95 129.15 128.37 127.96 126.16 122.09 116.70 114.02 109.62
77.32 77.00 76.68 70.66
55.28
25.70 18.29
-5.11 -5.17
MeO
O
N
OTBS
Ph
3q
f1 (ppm)

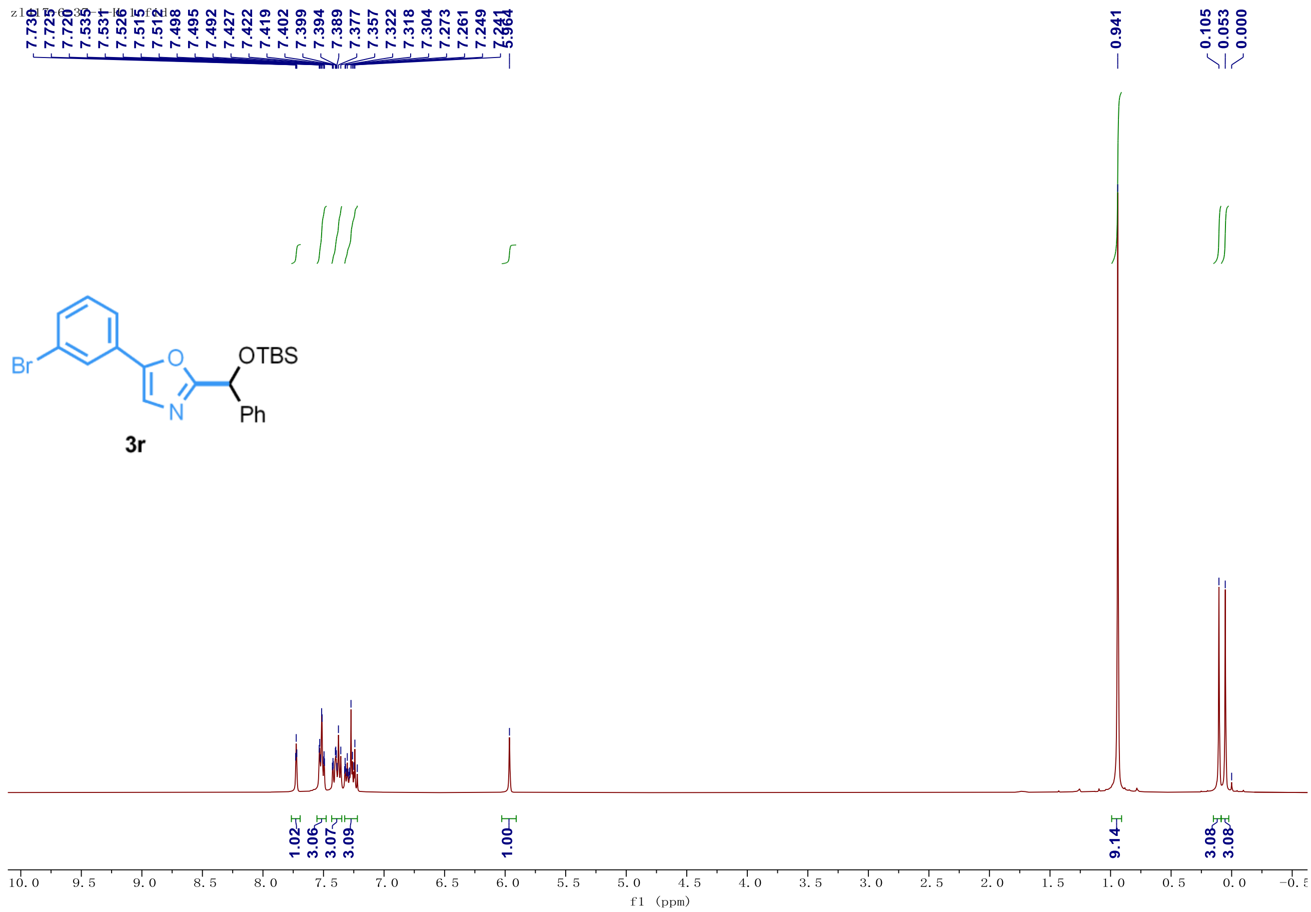
7.730
7.725
7.720
7.535
7.531
7.526
7.515
7.512
7.498
7.495
7.492
7.427
7.422
7.419
7.402
7.399
7.394
7.389
7.377
7.357
7.322
7.318
7.304
7.273
7.261
7.249
7.241
5.964
0.941
0.105
0.053
0.000
Br
OTBS
O
N
Ph
3r
1.02
3.06
3.07
3.09
1.00
9.14
3.08
3.08
f1 (ppm)

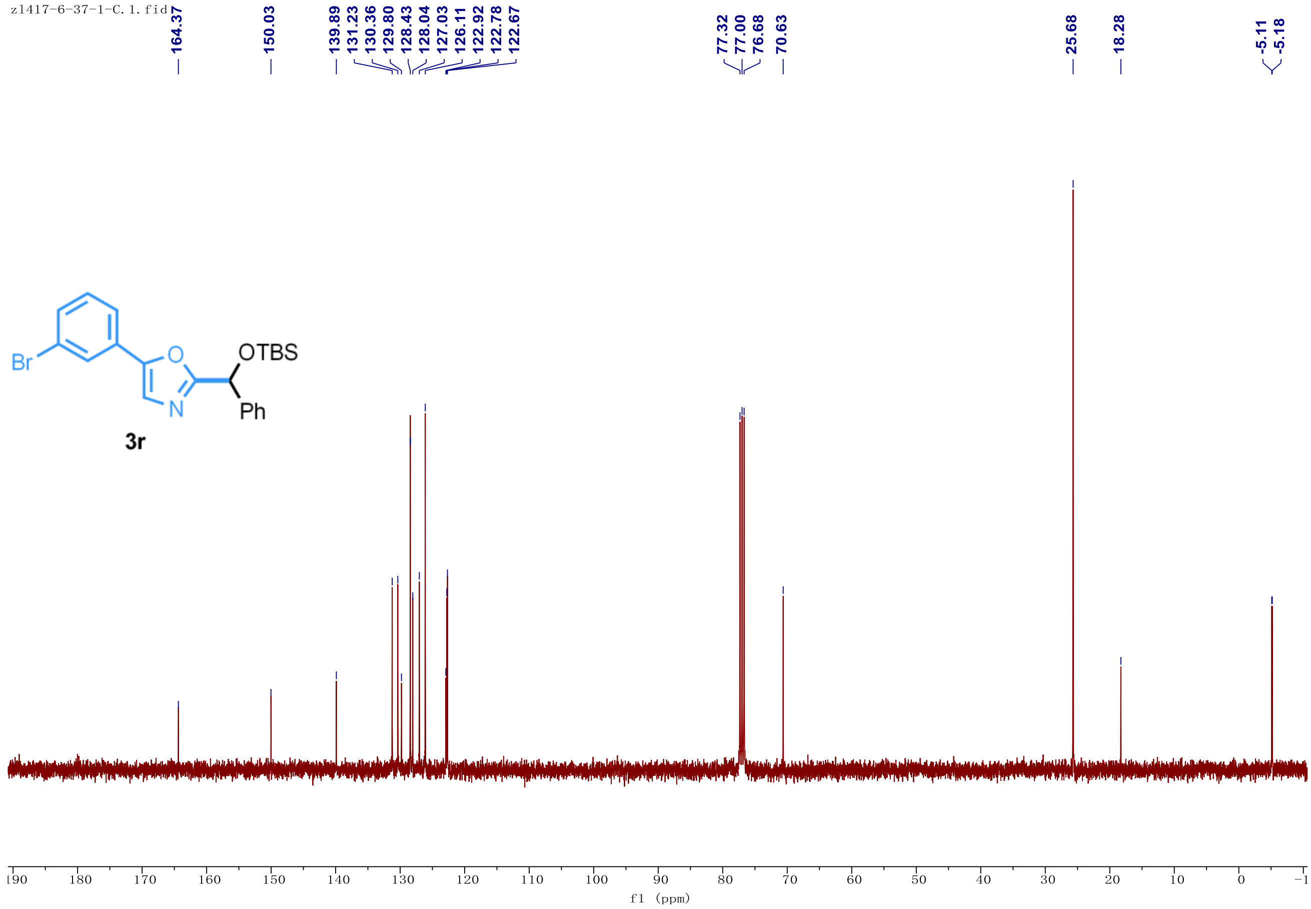
z1417-6-37-1-C.1.fid
164.37
150.03
139.89
131.23
130.36
129.80
128.43
128.04
127.03
126.11
122.92
122.78
122.67
77.32
77.00
76.68
70.63
25.68
18.28
-5.11
-5.18
Br
OTBS
O
N
Ph
3r
f1 (ppm)

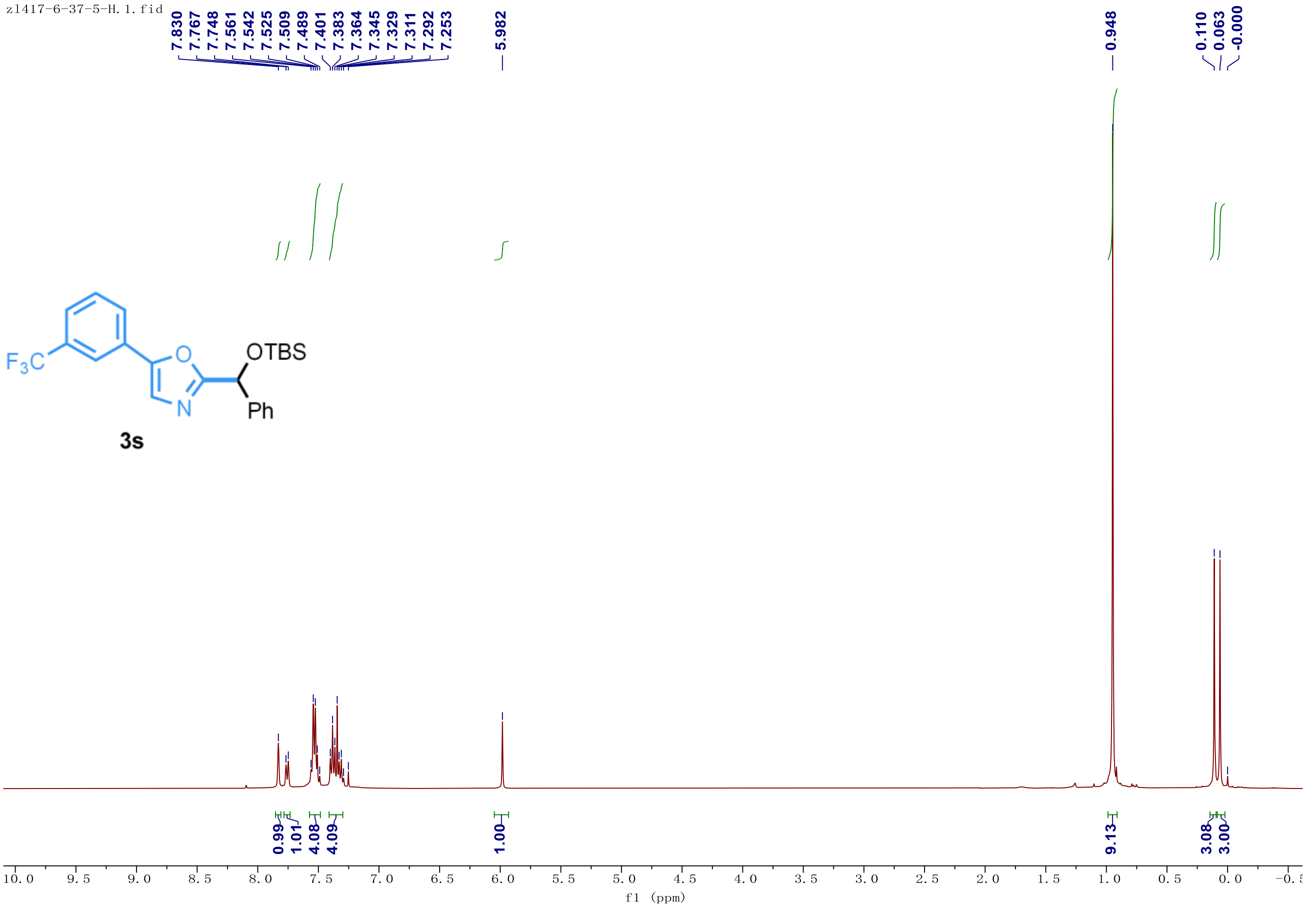

z1417-6-37-5-H.1.fid
7.830
7.767
7.748
7.561
7.542
7.525
7.509
7.489
7.401
7.383
7.364
7.345
7.329
7.311
7.292
7.253
5.982
0.948
0.110
0.063
-0.000
OTBS
Ph
3s
0.99
1.01
4.08
4.09
1.00
9.13
3.08
3.00
f1 (ppm)

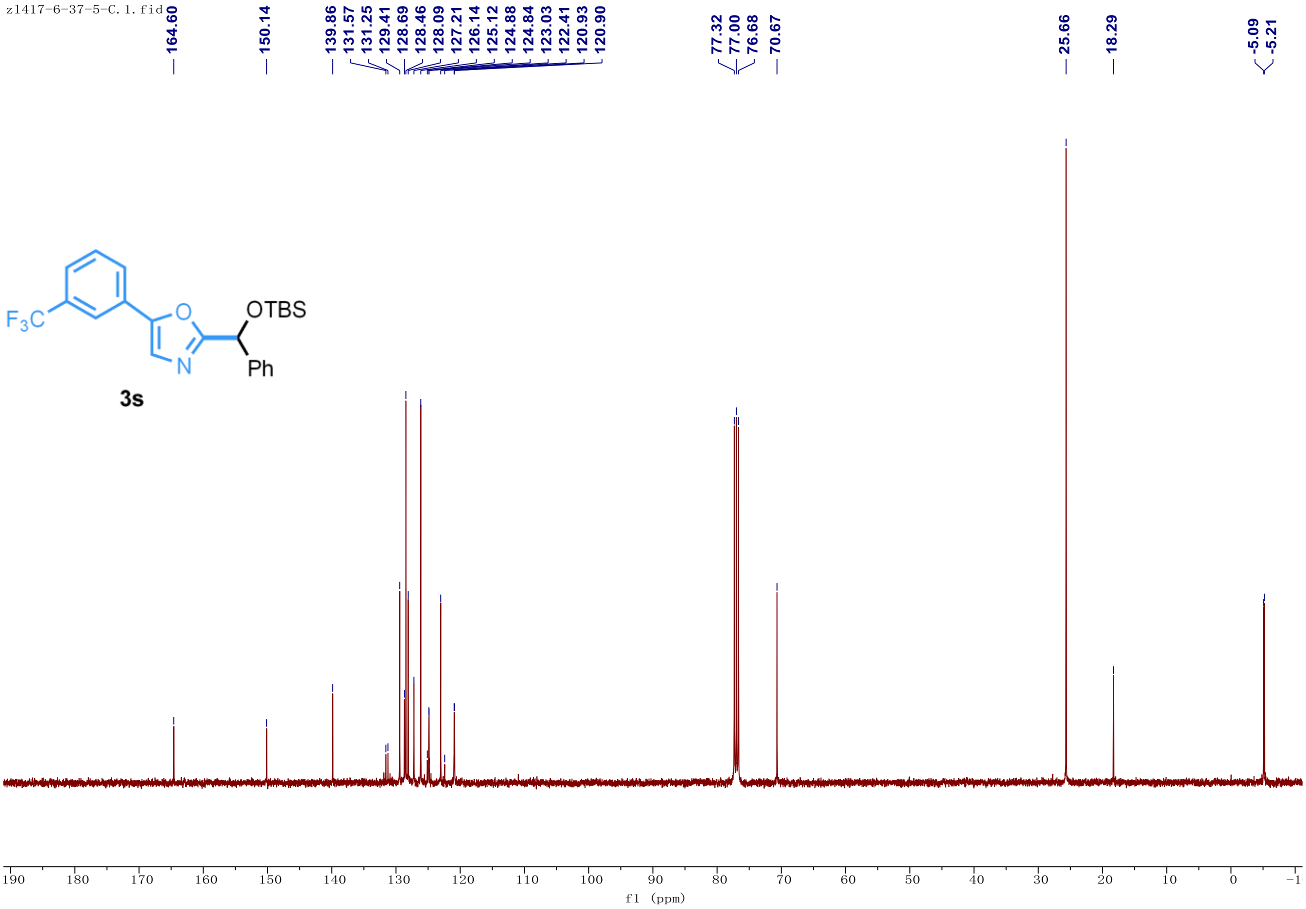

z1417-6-37-5-C.1.fid
164.60
150.14
139.86
131.57
131.25
129.41
128.69
128.46
128.09
127.21
126.14
125.12
124.88
124.84
123.03
122.41
120.93
120.90
77.32
77.00
76.68
70.67
25.66
18.29
-5.09
-5.21
OTBS
Ph
3s
f1 (ppm)

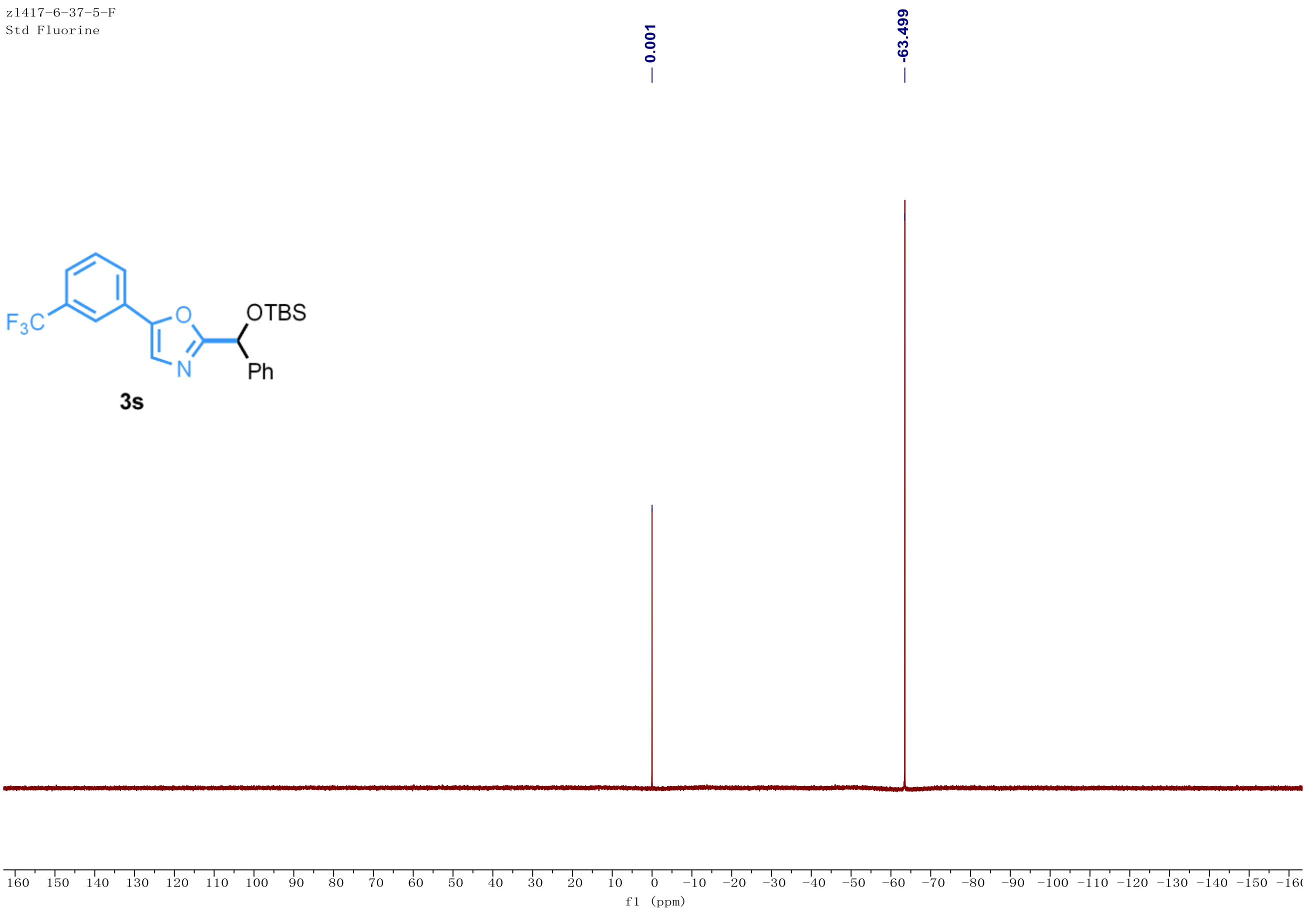

zl417-6-37-5-F
Std Fluorine
0.001
-63.499
F3C
OTBS
Ph
3s
f1 (ppm)

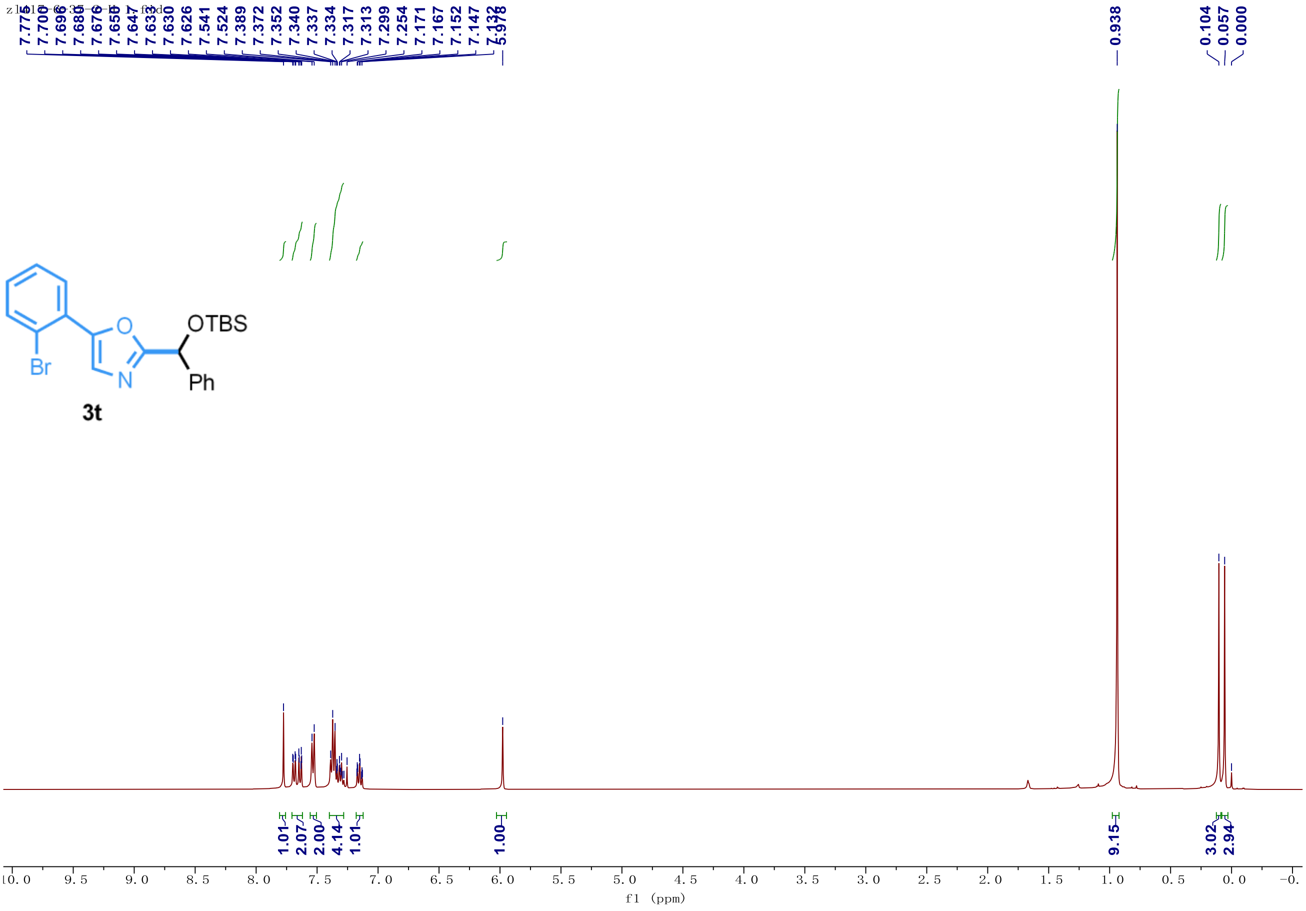

7.700
7.696
7.680
7.676
7.650
7.647
7.633
7.630
7.626
7.541
7.524
7.389
7.372
7.352
7.340
7.337
7.334
7.317
7.313
7.299
7.254
7.171
7.167
7.152
7.147
5.978
0.938
0.104
0.057
0.000
Br
OTBS
Ph
3t
1.01
2.07
2.00
4.14
1.01
1.00
9.15
3.02
2.94
f1 (ppm)

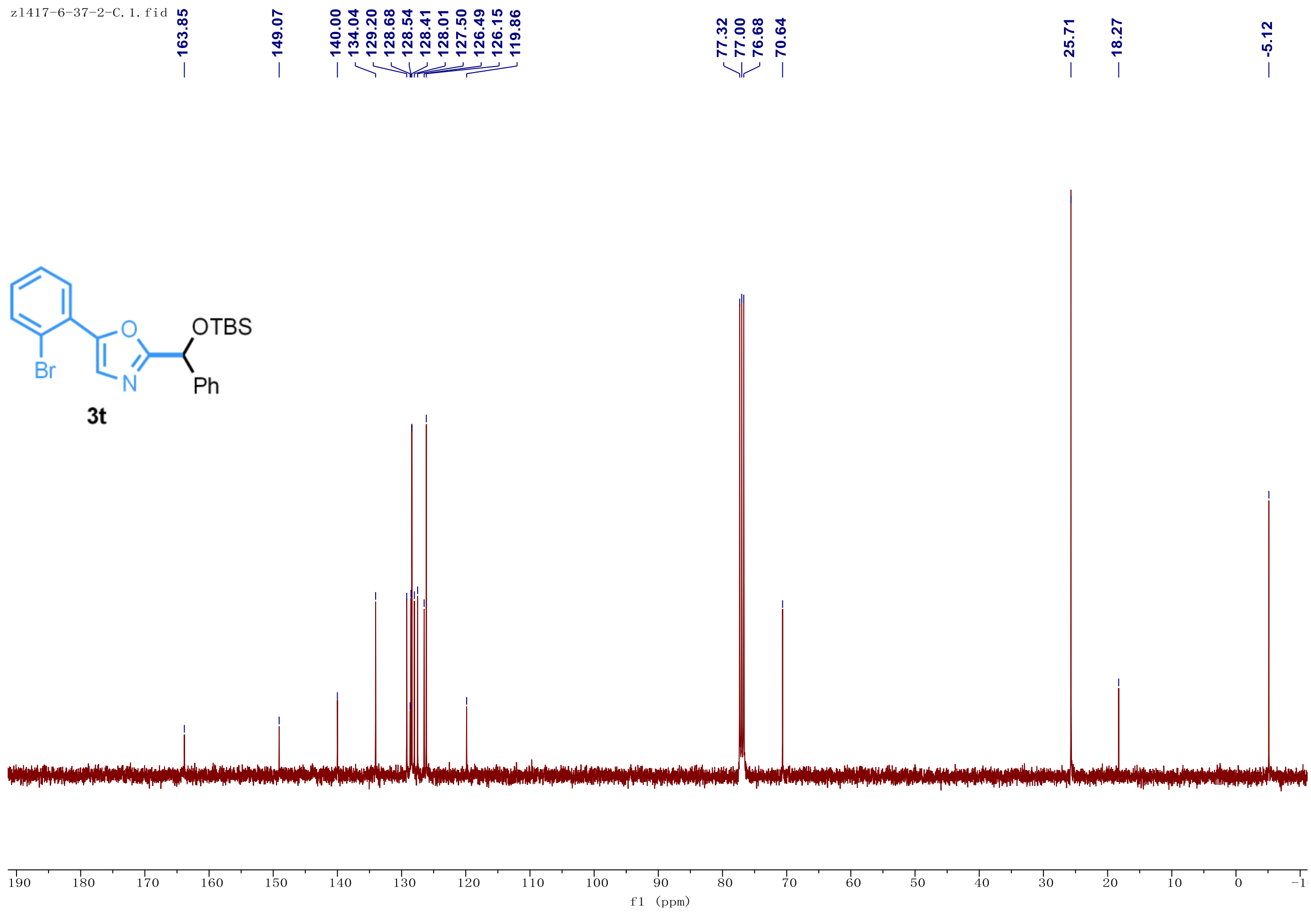
z1417-6-37-2-C.1.fid
163.85
149.07
140.00
134.04
129.20
128.68
128.54
128.41
128.01
127.50
126.49
126.15
119.86
77.32
77.00
76.68
70.64
25.71
18.27
-5.12
OTBS
O
N
Br
Ph
3t
190 180 170 160 150 140 130 120 110 100 90 80 70 60 50 40 30 20 10 0 -1
f1 (ppm)

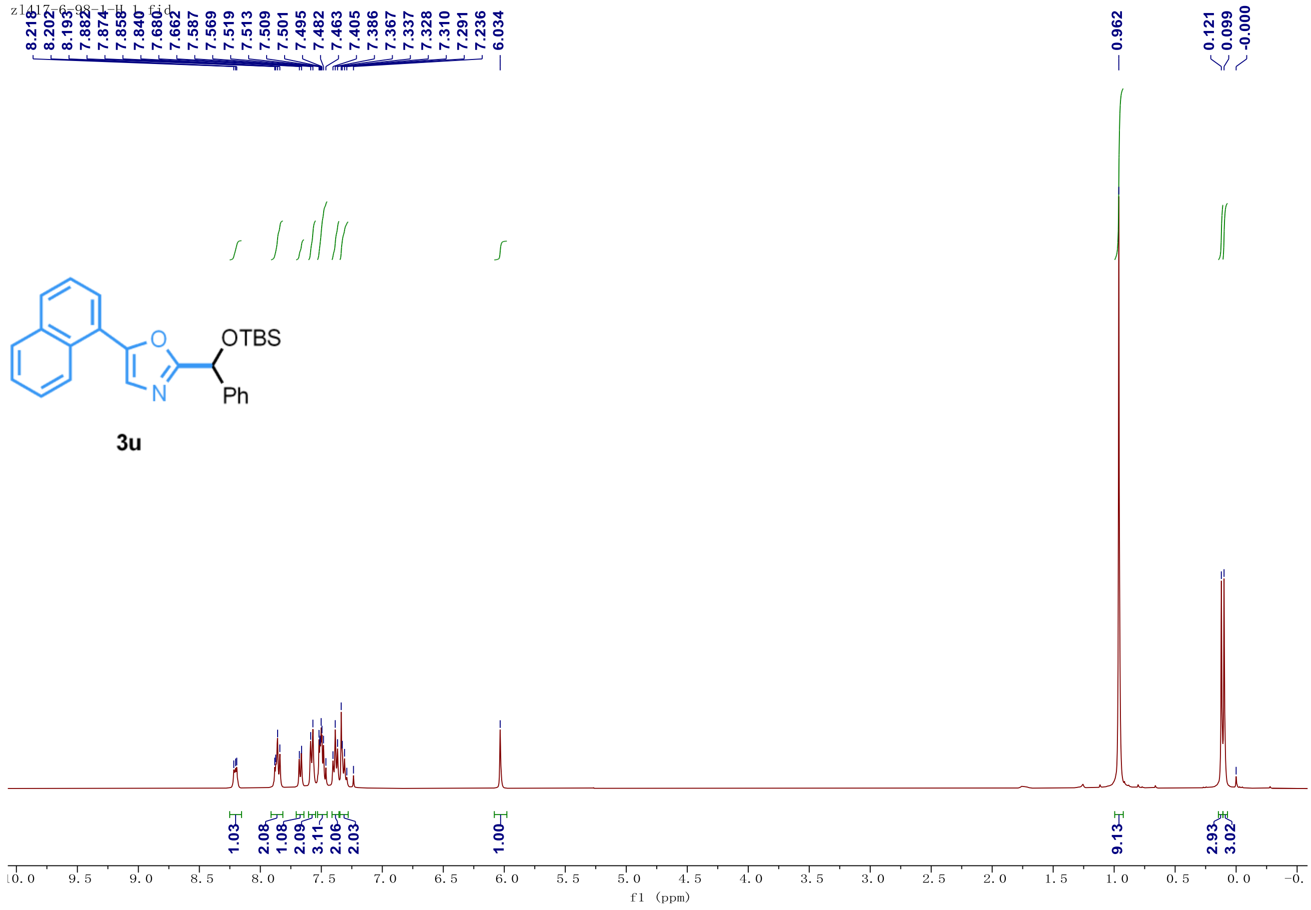
z1417-6-98-1-H.1.fid
8.218
8.202
8.193
7.882
7.874
7.858
7.840
7.680
7.662
7.587
7.569
7.519
7.513
7.509
7.501
7.495
7.482
7.463
7.405
7.386
7.367
7.337
7.328
7.310
7.291
7.236
6.034
0.962
0.121
0.099
-0.000
OTBS
O
N
Ph
3u
1.03
2.08
1.08
2.09
3.11
2.06
2.03
1.00
9.13
2.93
3.02
10.0 9.5 9.0 8.5 8.0 7.5 7.0 6.5 6.0 5.5 5.0 4.5 4.0 3.5 3.0 2.5 2.0 1.5 1.0 0.5 0.0 -0.
f1 (ppm)

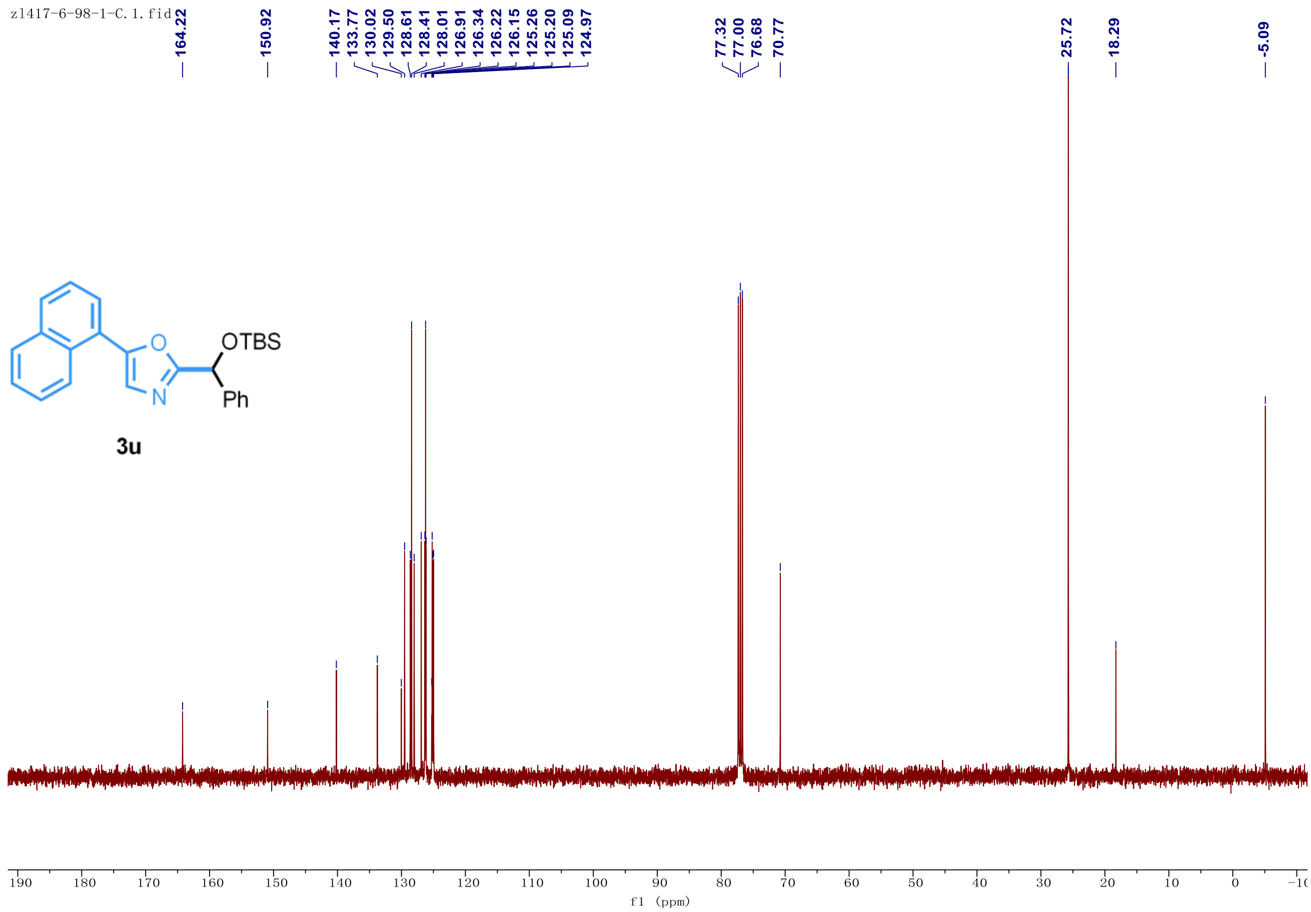

zl417-6-98-1-C.1.fid
164.22
150.92
140.17
133.77
130.02
129.50
128.61
128.41
128.01
126.91
126.34
126.22
126.15
125.26
125.20
125.09
124.97
77.32
77.00
76.68
70.77
25.72
18.29
-5.09
OTBS
O
N
Ph
3u
f1 (ppm)

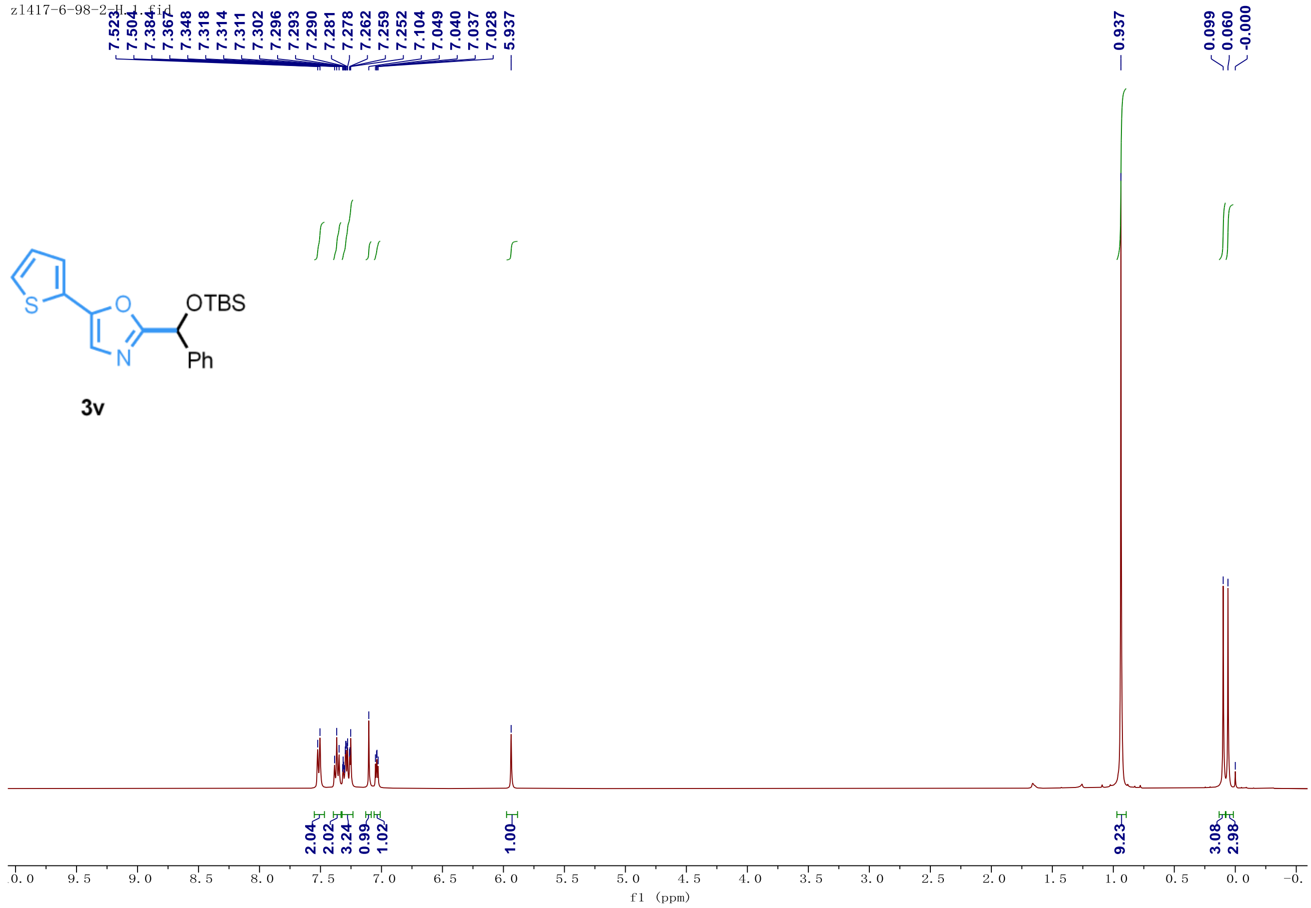

zl417-6-98-2-H.1.fid
7.523
7.504
7.384
7.367
7.348
7.318
7.314
7.311
7.302
7.296
7.293
7.290
7.281
7.278
7.262
7.259
7.252
7.104
7.049
7.040
7.037
7.028
5.937
0.937
0.099
0.060
-0.000
S
O
N
OTBS
Ph
3v
2.04
2.02
3.24
0.99
1.02
1.00
9.23
3.08
2.98
f1 (ppm)

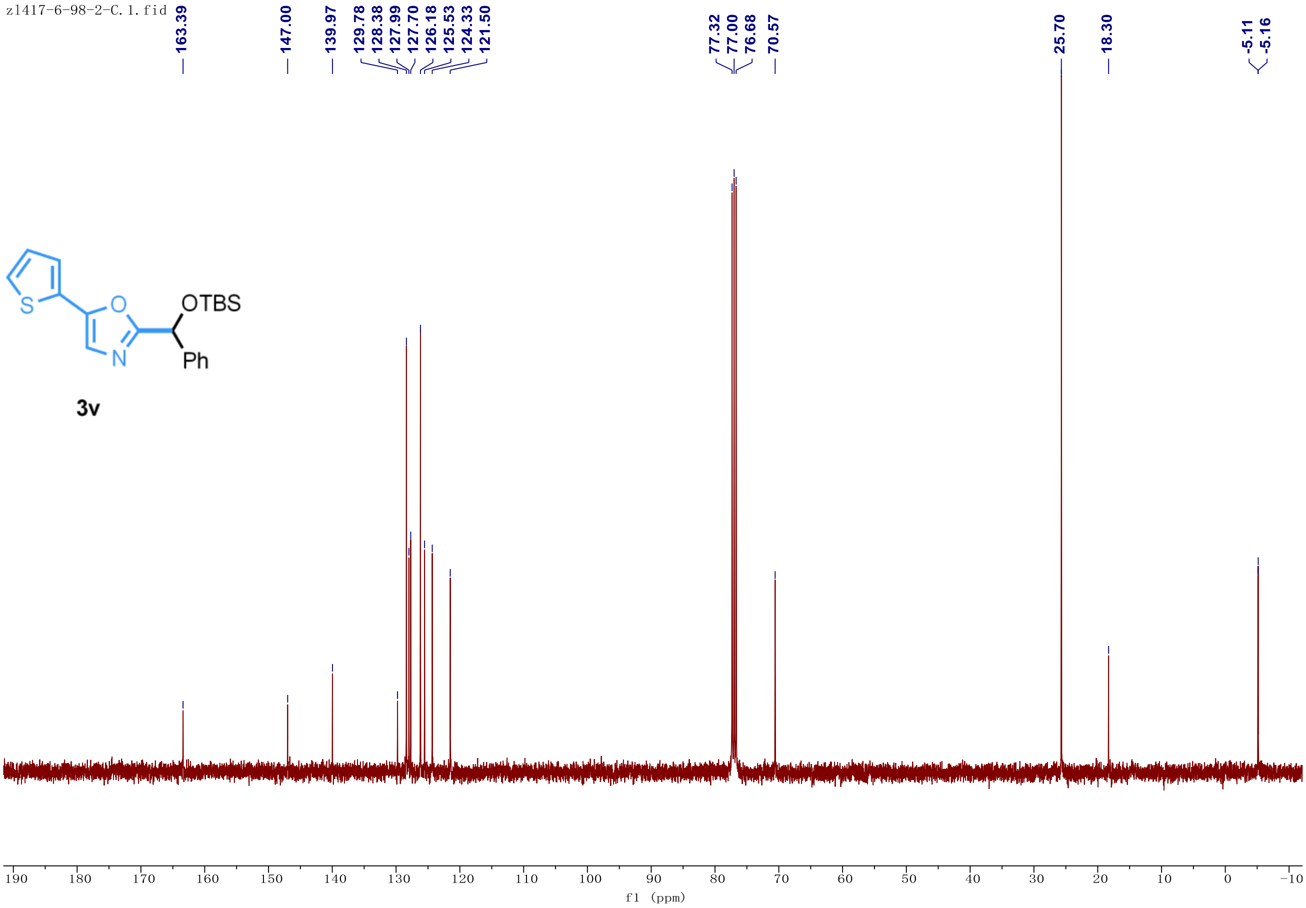
zl417-6-98-2-C.1.fid
163.39
147.00
139.97
129.78
128.38
127.99
127.70
126.18
125.53
124.33
121.50
77.32
77.00
76.68
70.57
25.70
18.30
-5.11
-5.16
OTBS
Ph
S
O
N
3v
f1 (ppm)

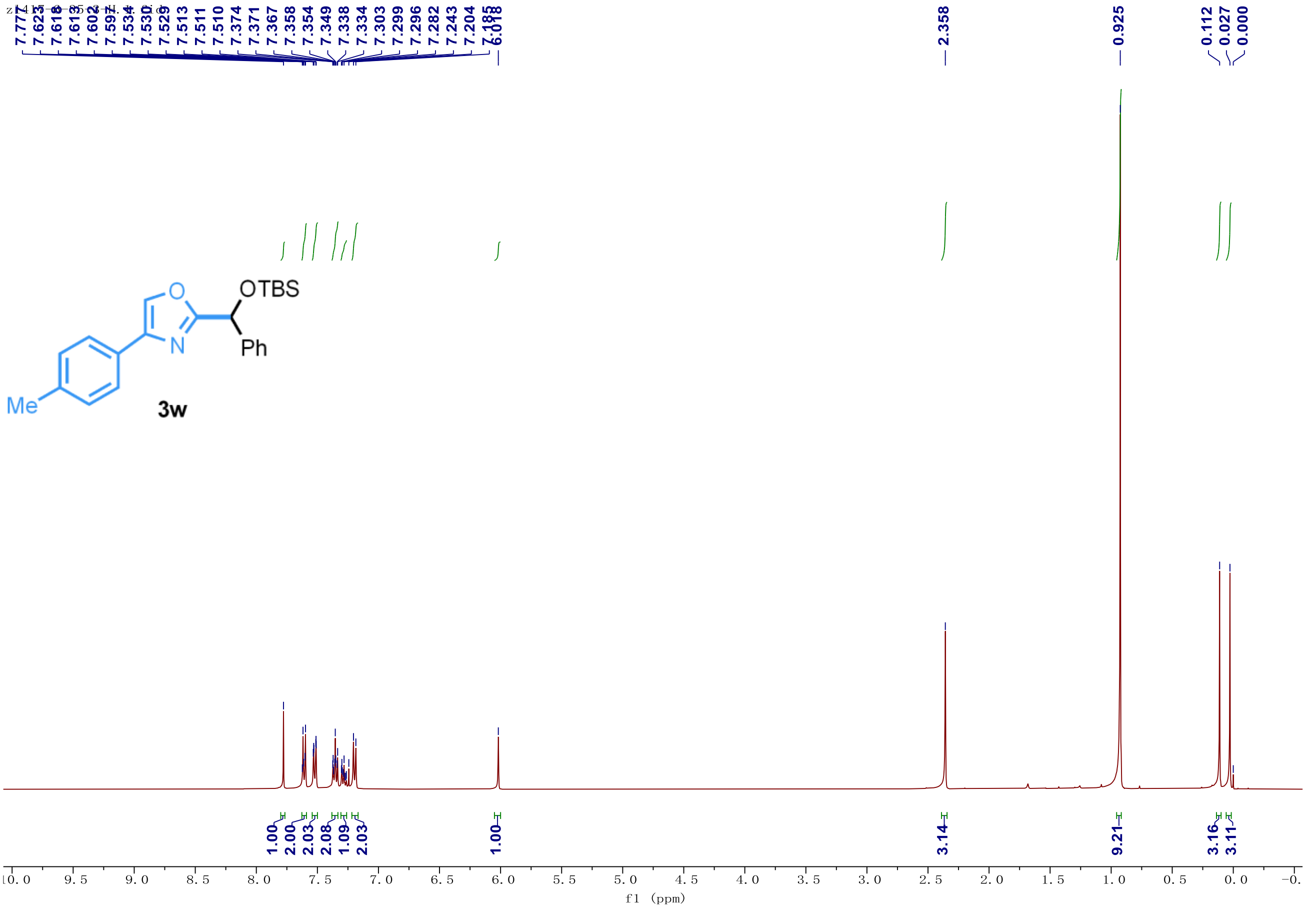
7.602
7.597
7.534
7.530
7.529
7.513
7.511
7.510
7.374
7.371
7.367
7.358
7.354
7.349
7.338
7.334
7.303
7.299
7.296
7.282
7.243
7.204
6.018
2.358
0.925
0.112
0.027
0.000
OTBS
Ph
O
N
Me
3w
1.00
2.00
2.03
2.08
1.09
2.03
1.00
3.14
9.21
3.16
3.11
f1 (ppm)

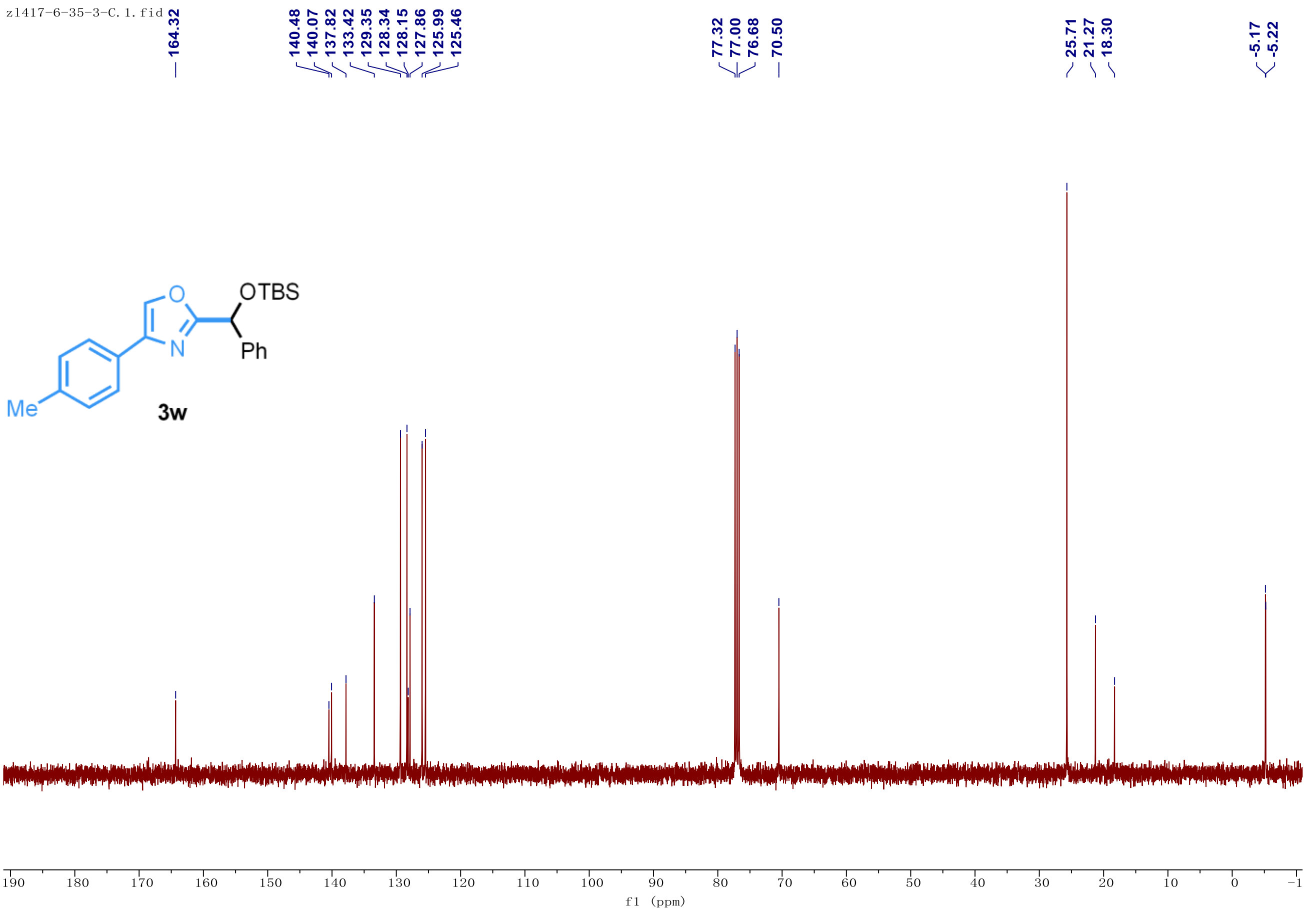
z1417-6-35-3-C.1.fid
164.32
140.48
140.07
137.82
133.42
129.35
128.34
128.15
127.86
125.99
125.46
77.32
77.00
76.68
70.50
25.71
21.27
18.30
-5.17
-5.22
OTBS
O
N
Ph
Me
3w
190 180 170 160 150 140 130 120 110 100 90 80 70 60 50 40 30 20 10 0 -1
f1 (ppm)

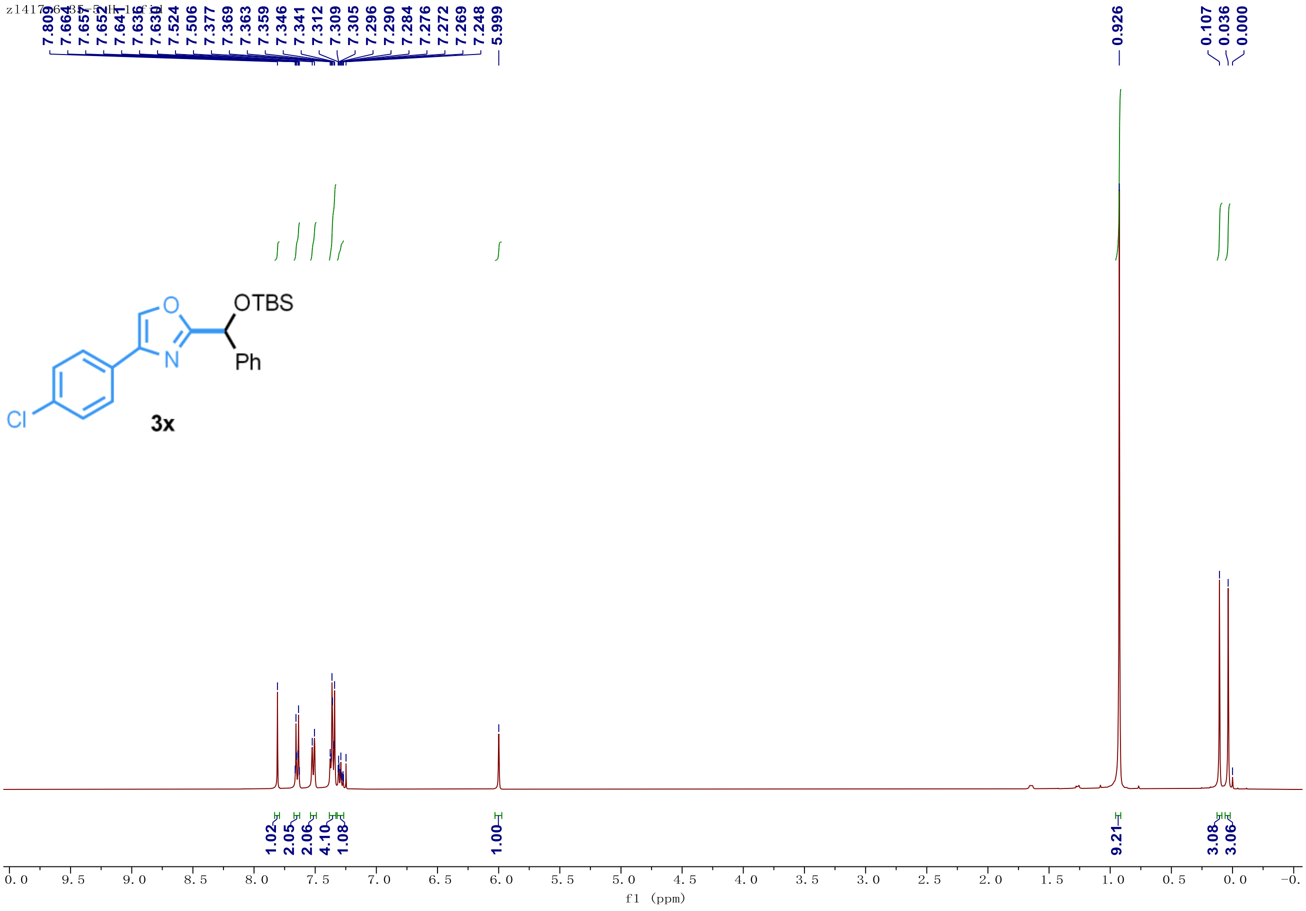
7.809
7.664
7.657
7.652
7.641
7.636
7.630
7.524
7.506
7.377
7.369
7.363
7.359
7.346
7.341
7.312
7.309
7.305
7.296
7.290
7.284
7.276
7.272
7.269
7.248
5.999
0.926
0.107
0.036
0.000
OTBS
O
N
Ph
Cl
3x
1.02
2.05
2.06
4.10
1.08
1.00
9.21
3.08
3.06
0.0 9.5 9.0 8.5 8.0 7.5 7.0 6.5 6.0 5.5 5.0 4.5 4.0 3.5 3.0 2.5 2.0 1.5 1.0 0.5 0.0 -0.
f1 (ppm)

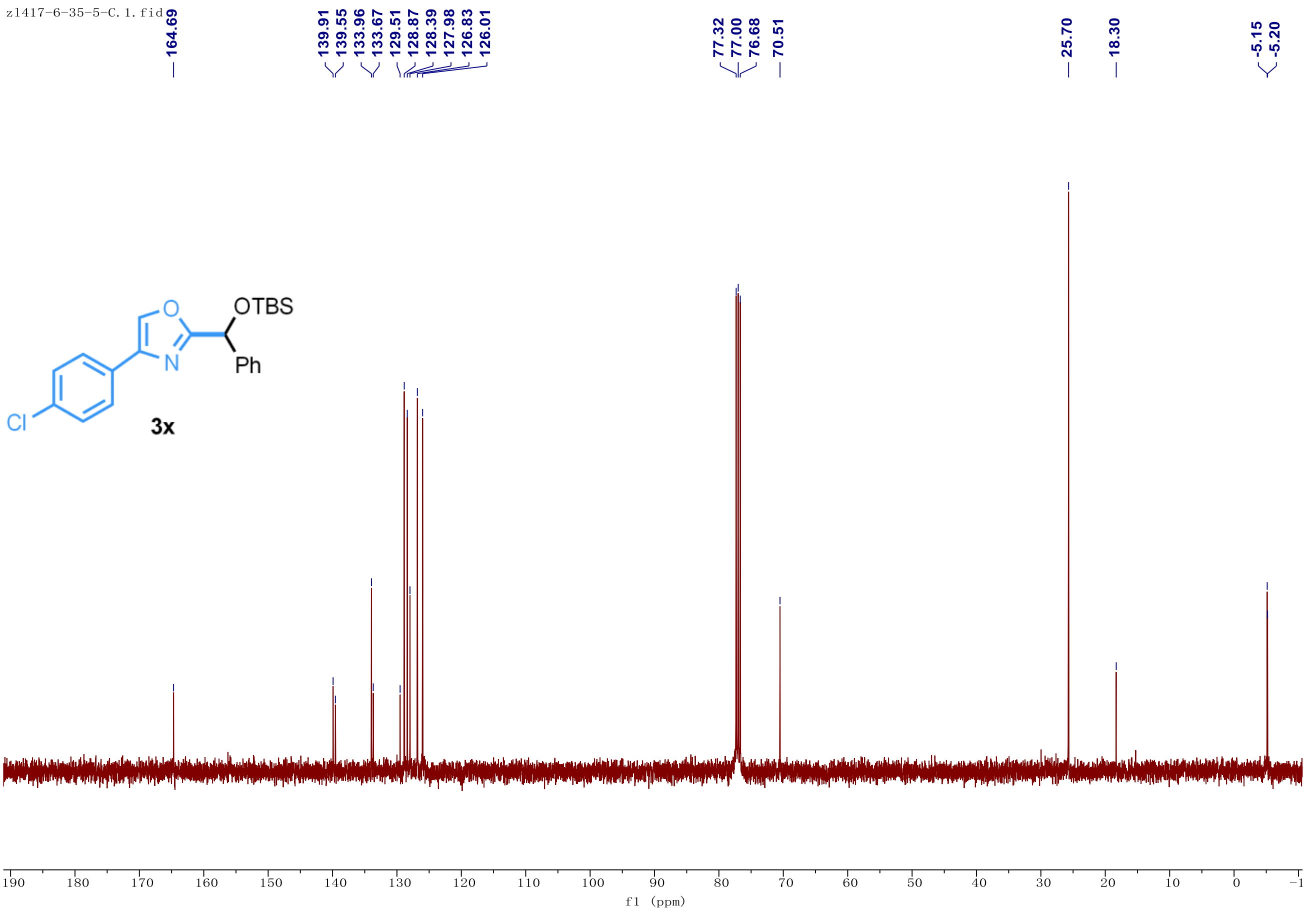
zl417-6-35-5-C.1.fid
164.69
139.91
139.55
133.96
133.67
129.51
128.87
128.39
127.98
126.83
126.01
77.32
77.00
76.68
70.51
25.70
18.30
-5.15
-5.20
OTBS
O
N
Ph
Cl
3x
190 180 170 160 150 140 130 120 110 100 90 80 70 60 50 40 30 20 10 0 -1
f1 (ppm)

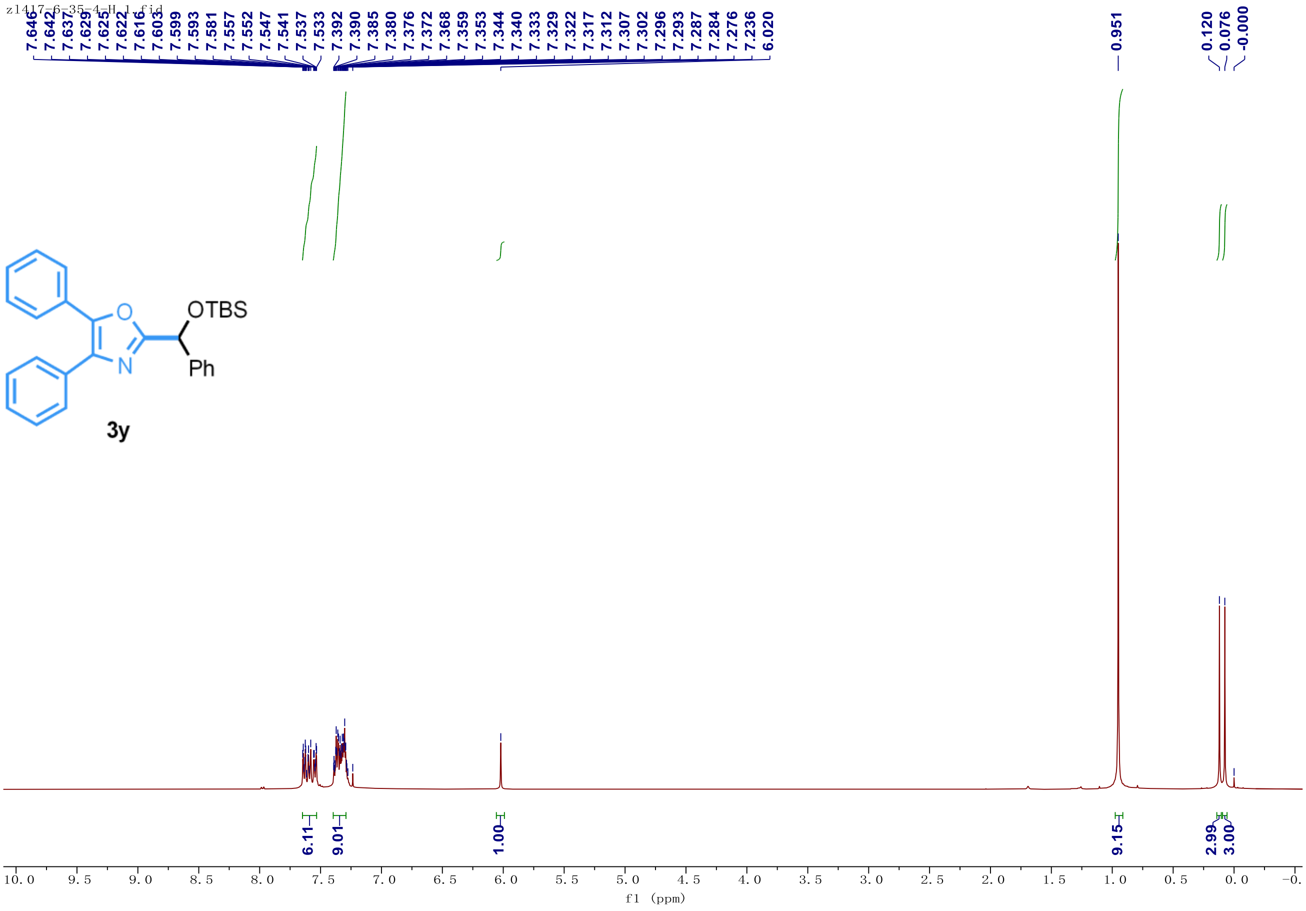
zl417-6-35-4-H.1.fid
7.646
7.642
7.637
7.629
7.625
7.622
7.616
7.603
7.599
7.593
7.581
7.557
7.552
7.547
7.541
7.537
7.533
7.392
7.390
7.385
7.380
7.376
7.372
7.368
7.359
7.353
7.344
7.340
7.333
7.329
7.322
7.317
7.312
7.307
7.302
7.296
7.293
7.287
7.284
7.276
7.236
6.020
0.951
0.120
0.076
-0.000
OTBS
O
N
Ph
3y
6.11
9.01
1.00
9.15
2.99
3.00
10.0 9.5 9.0 8.5 8.0 7.5 7.0 6.5 6.0 5.5 5.0 4.5 4.0 3.5 3.0 2.5 2.0 1.5 1.0 0.5 0.0 -0.
f1 (ppm)

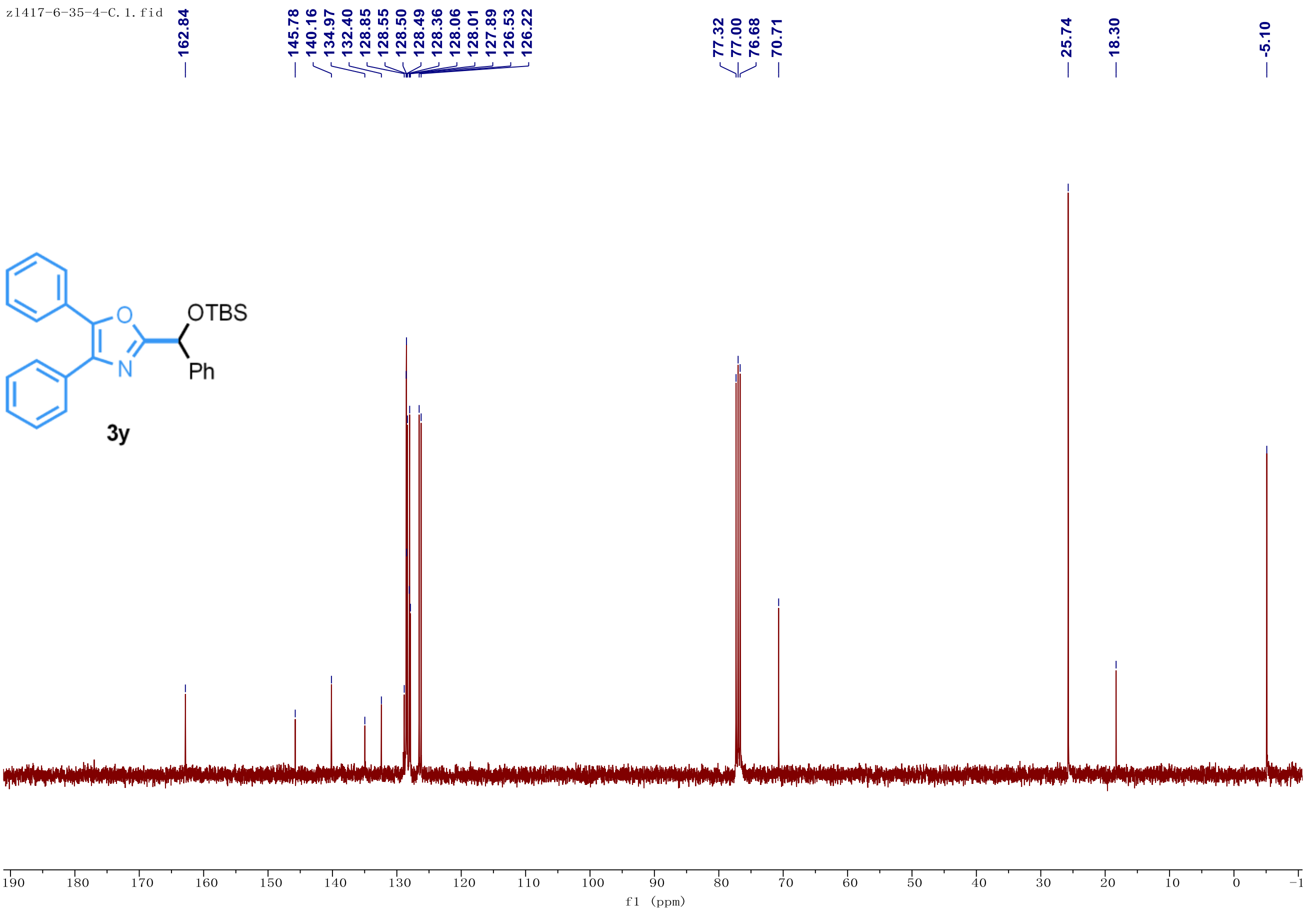
zl417-6-35-4-C.1.fid
162.84
145.78
140.16
134.97
132.40
128.85
128.55
128.50
128.49
128.36
128.06
128.01
127.89
126.53
126.22
77.32
77.00
76.68
70.71
25.74
18.30
-5.10
OTBS
O
N
Ph
3y
190 180 170 160 150 140 130 120 110 100 90 80 70 60 50 40 30 20 10 0 -1
f1 (ppm)

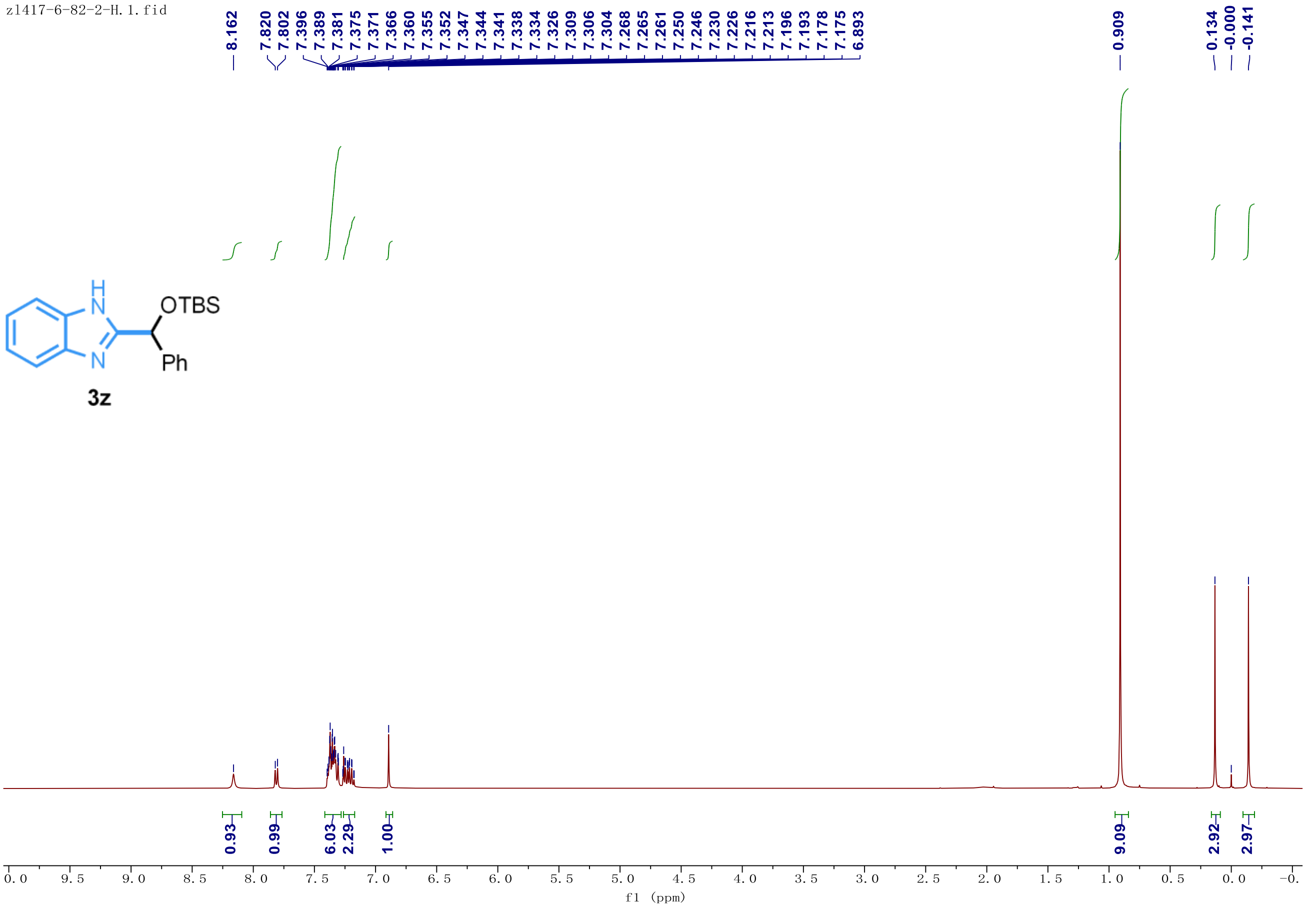
zl417-6-82-2-H.1.fid
8.162
7.820
7.802
7.396
7.389
7.381
7.375
7.371
7.366
7.360
7.355
7.352
7.347
7.344
7.341
7.338
7.334
7.326
7.309
7.306
7.304
7.268
7.265
7.261
7.250
7.246
7.230
7.226
7.216
7.213
7.196
7.193
7.178
7.175
6.893
0.909
0.134
-0.000
-0.141
H
N
N
OTBS
Ph
3z
0.93
0.99
6.03
2.29
1.00
9.09
2.92
2.97
0.0 9.5 9.0 8.5 8.0 7.5 7.0 6.5 6.0 5.5 5.0 4.5 4.0 3.5 3.0 2.5 2.0 1.5 1.0 0.5 0.0 -0.
f1 (ppm)

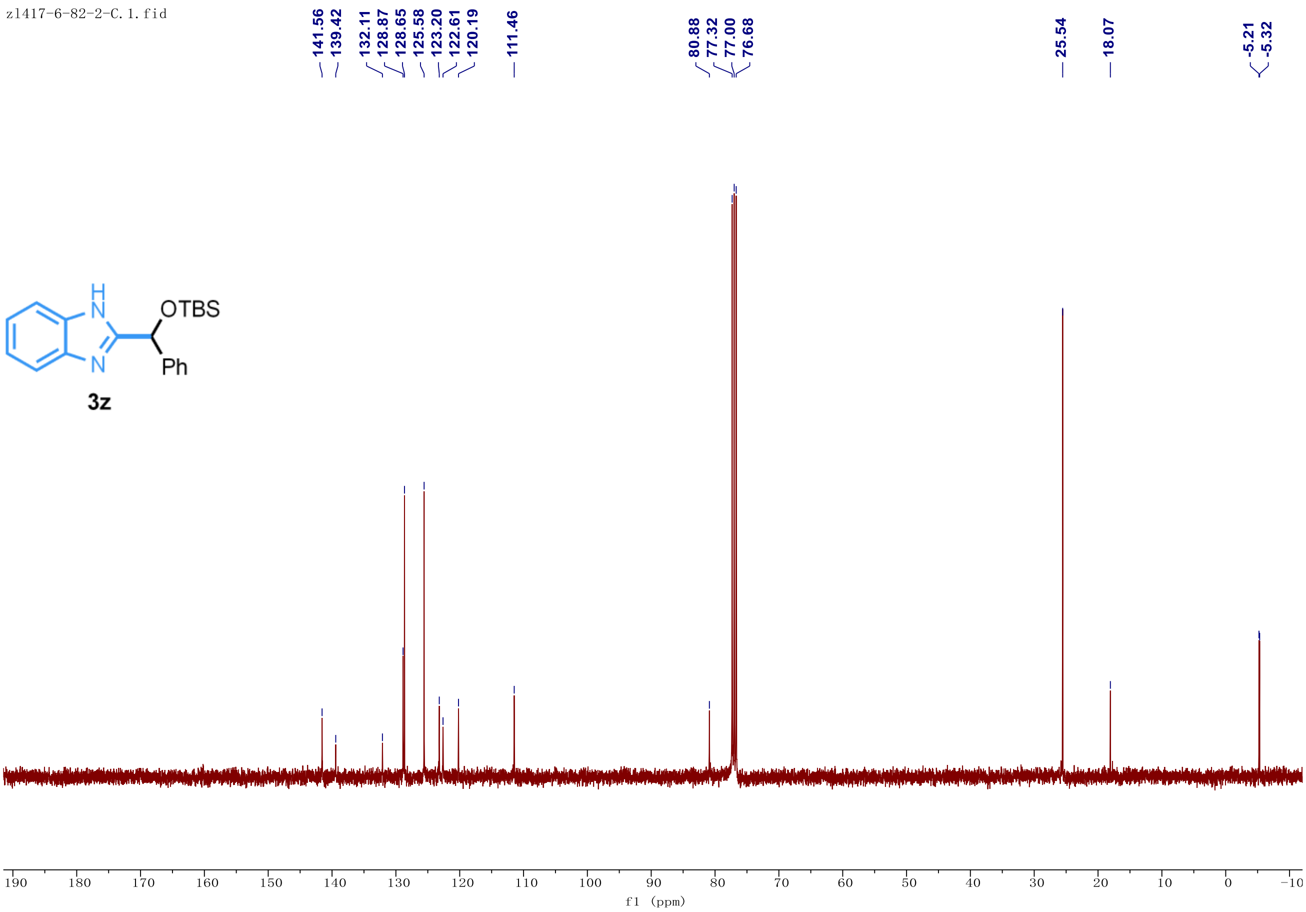
z1417-6-82-2-C.1.fid
141.56
139.42
132.11
128.87
128.65
125.58
123.20
122.61
120.19
111.46
80.88
77.32
77.00
76.68
25.54
18.07
-5.21
-5.32
OTBS
Ph
f1 (ppm)

3z

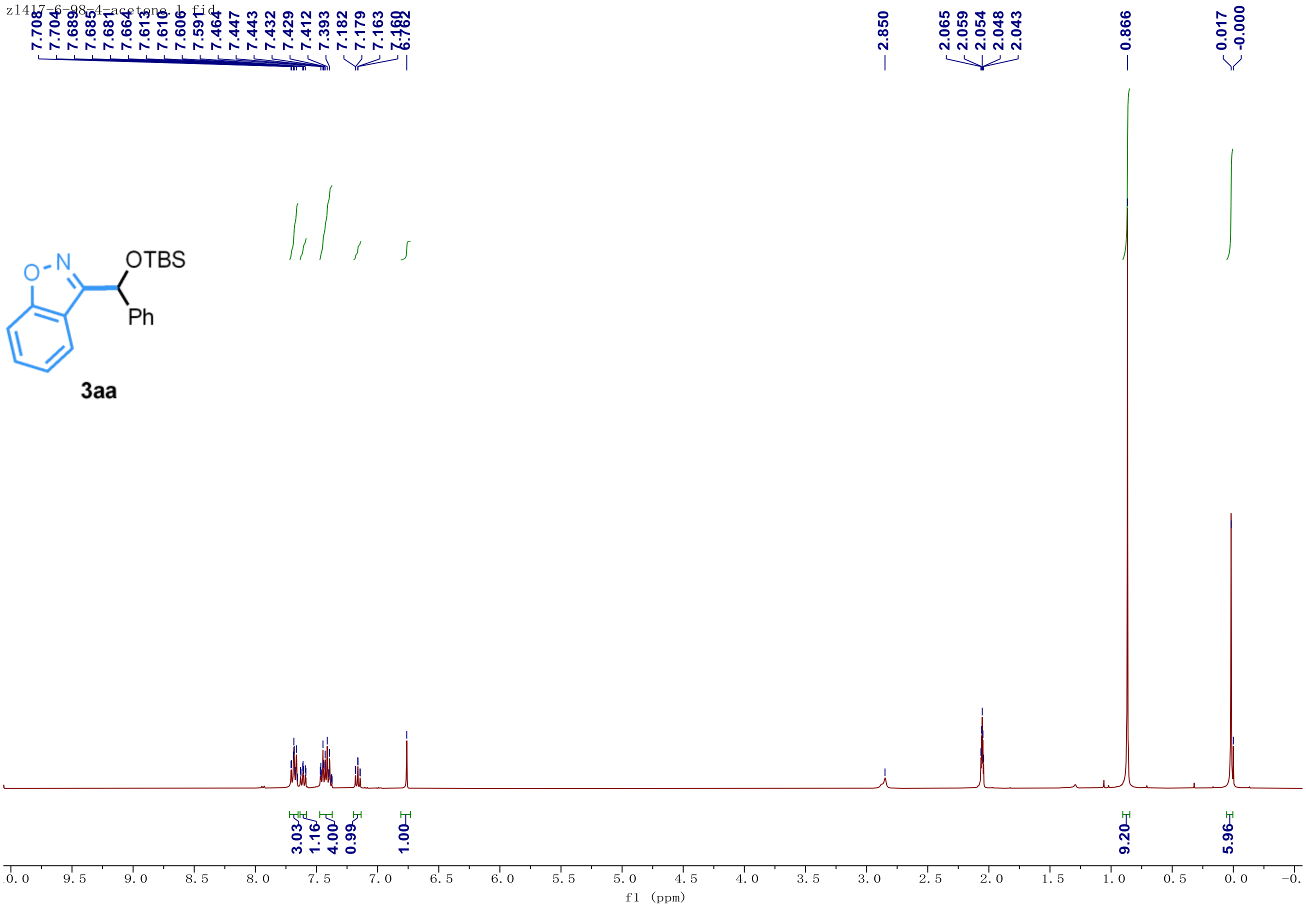
z1417-6-98-4-acetone.1.fid
7.708
7.704
7.689
7.685
7.681
7.664
7.613
7.610
7.606
7.591
7.464
7.447
7.443
7.432
7.429
7.412
7.393
7.182
7.179
7.163
7.160
6.782
2.850
2.065
2.059
2.054
2.048
2.043
0.866
0.017
-0.000
3.03
1.16
4.00
0.99
1.00
9.20
5.96
OTBS
Ph
f1 (ppm)

3aa

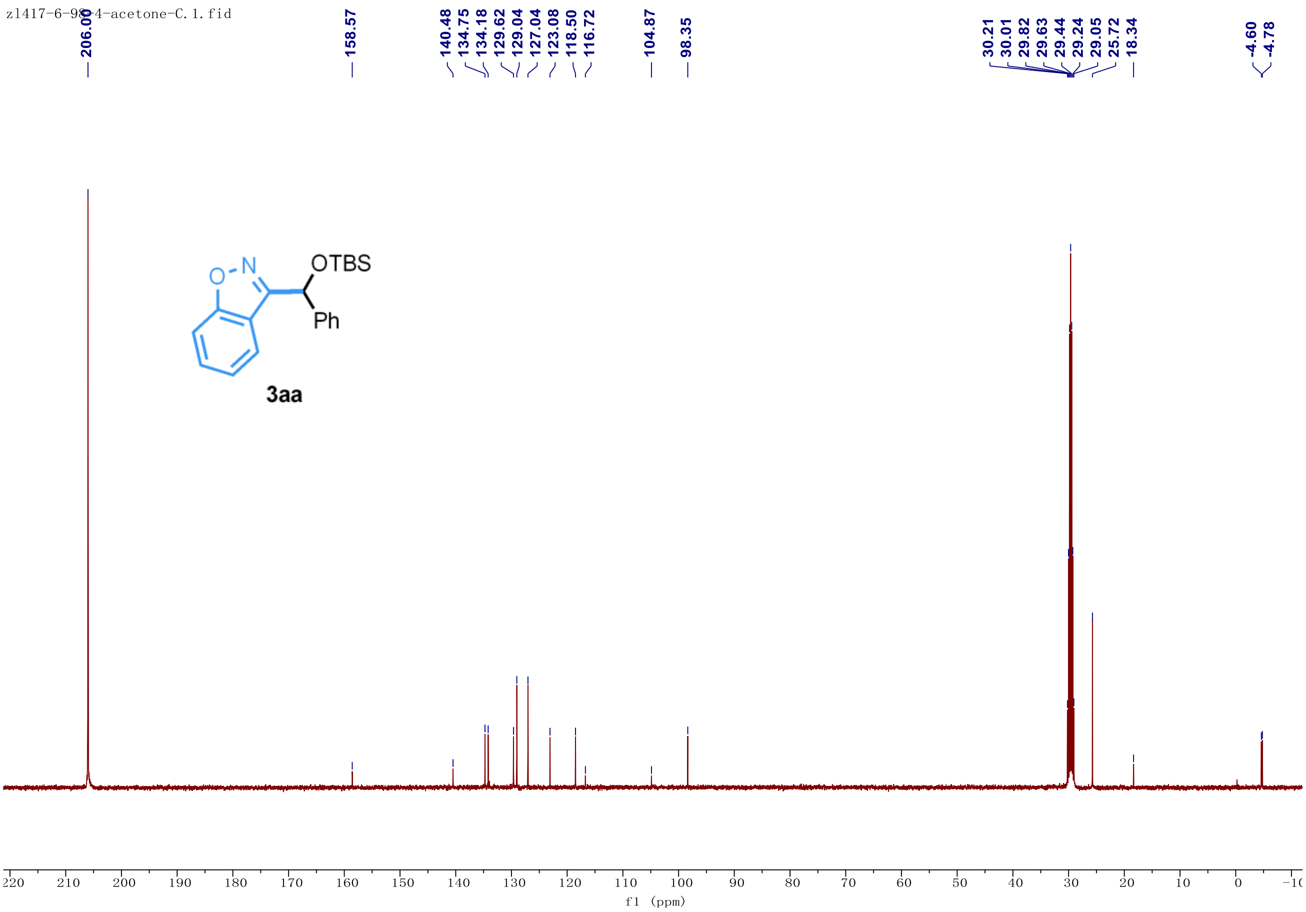
z1417-6-98-4-acetone-C.1.fid
158.57
140.48
134.75
134.18
129.62
129.04
127.04
123.08
118.50
116.72
104.87
98.35
30.21
30.01
29.82
29.63
29.44
29.24
29.05
25.72
18.34
-4.60
-4.78
OTBS
O-N
Ph
3aa
220 210 200 190 180 170 160 150 140 130 120 110 100 90 80 70 60 50 40 30 20 10 0
f1 (ppm)

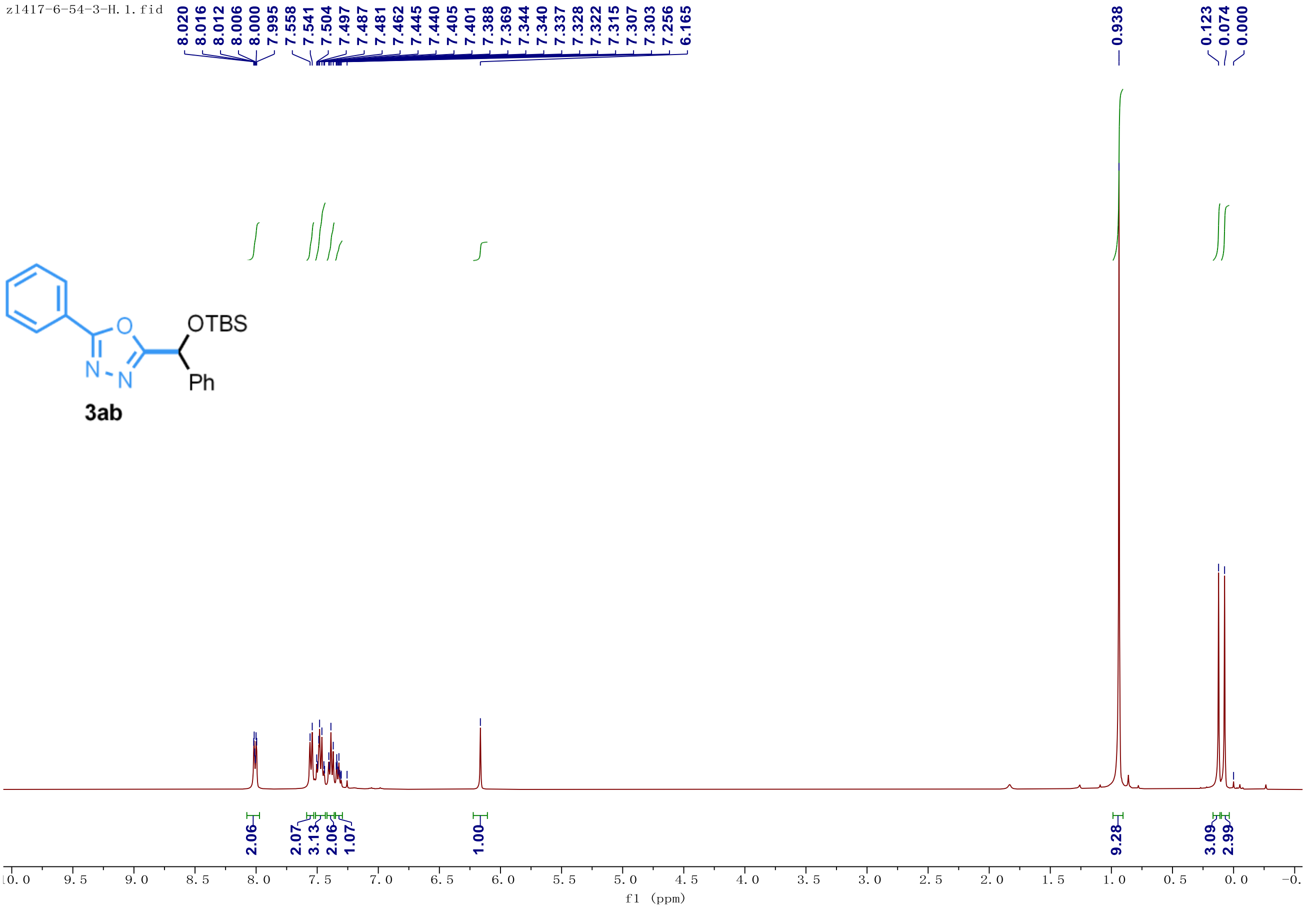
z1417-6-54-3-H.1.fid
8.020
8.016
8.012
8.006
8.000
7.995
7.558
7.541
7.504
7.497
7.487
7.481
7.462
7.445
7.440
7.405
7.401
7.388
7.369
7.344
7.340
7.337
7.328
7.322
7.315
7.307
7.303
7.256
6.165
0.938
0.123
0.074
0.000
OTBS
O
N-N
Ph
3ab
2.06
2.07
3.13
2.06
1.07
1.00
9.28
3.09
2.99
10.0 9.5 9.0 8.5 8.0 7.5 7.0 6.5 6.0 5.5 5.0 4.5 4.0 3.5 3.0 2.5 2.0 1.5 1.0 0.5 0.0
f1 (ppm)

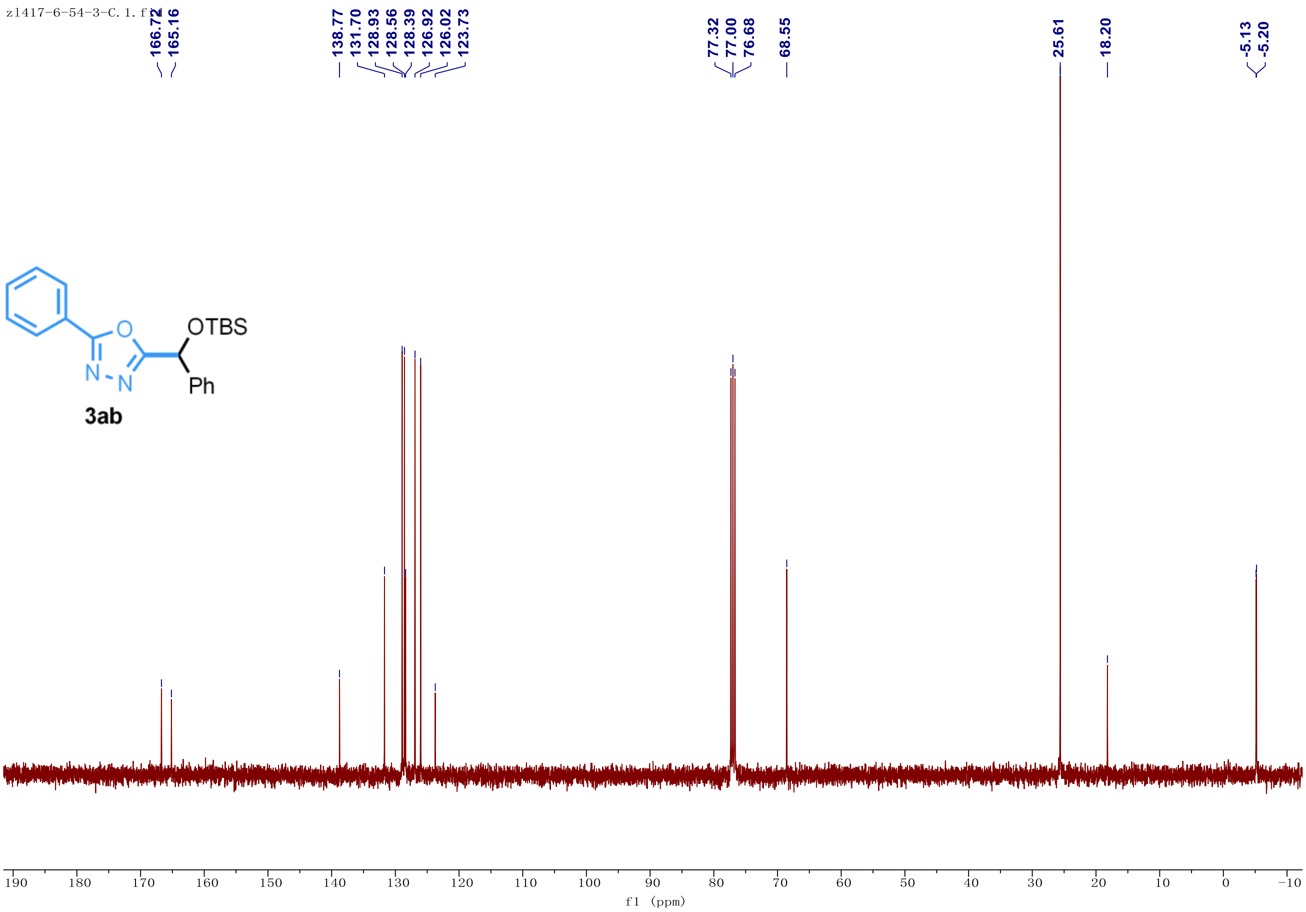
z1417-6-54-3-C.1.fid
166.72
165.16
138.77
131.70
128.93
128.56
128.39
126.92
126.02
123.73
77.32
77.00
76.68
68.55
25.61
18.20
-5.13
-5.20
OTBS
Ph
O
N
N
3ab
f1 (ppm)

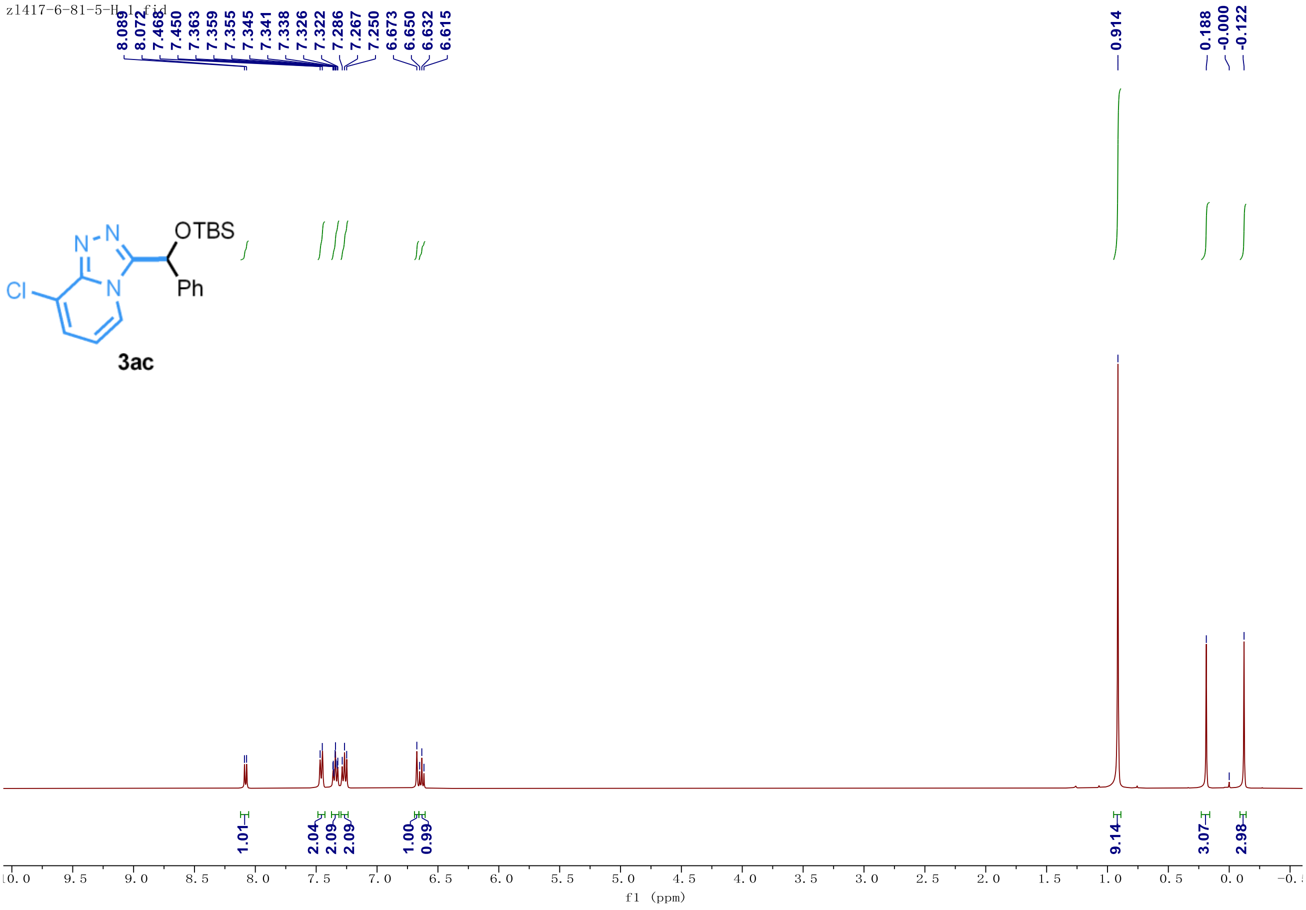
z1417-6-81-5-H.1.fid
8.089
8.072
7.468
7.450
7.363
7.359
7.355
7.345
7.341
7.338
7.326
7.322
7.286
7.267
7.250
6.673
6.650
6.632
6.615
0.914
0.188
-0.000
-0.122
OTBS
Ph
Cl
N
N
N
3ac
1.01
2.04
2.09
2.09
1.00
0.99
9.14
3.07
2.98
f1 (ppm)

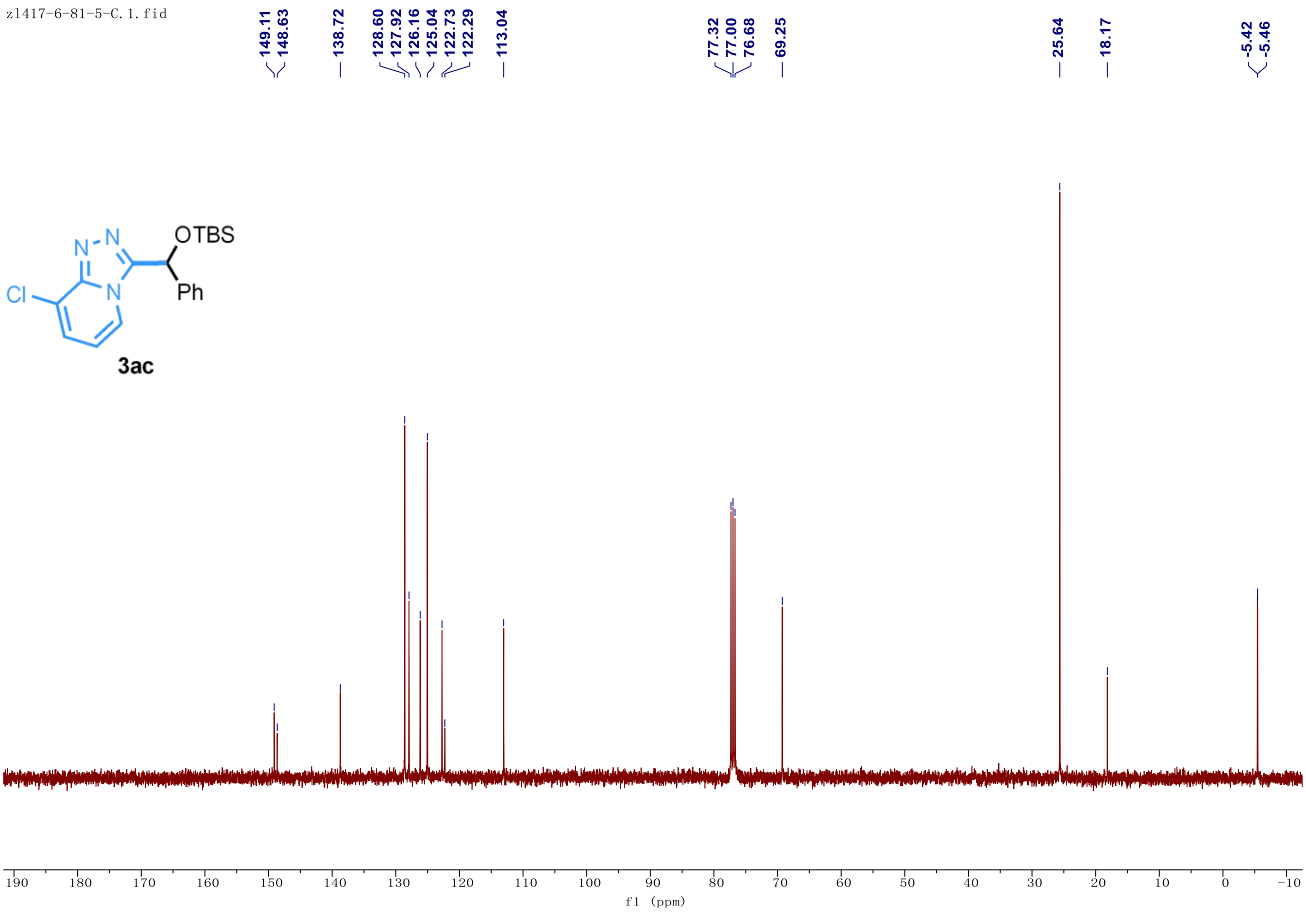

zl417-6-81-5-C.1.fid
149.11
148.63
138.72
128.60
127.92
126.16
125.04
122.73
122.29
113.04
77.32
77.00
76.68
69.25
25.64
18.17
-5.42
-5.46
N
N
OTBS
Cl
N
Ph
3ac
190 180 170 160 150 140 130 120 110 100 90 80 70 60 50 40 30 20 10 0 -10
f1 (ppm)

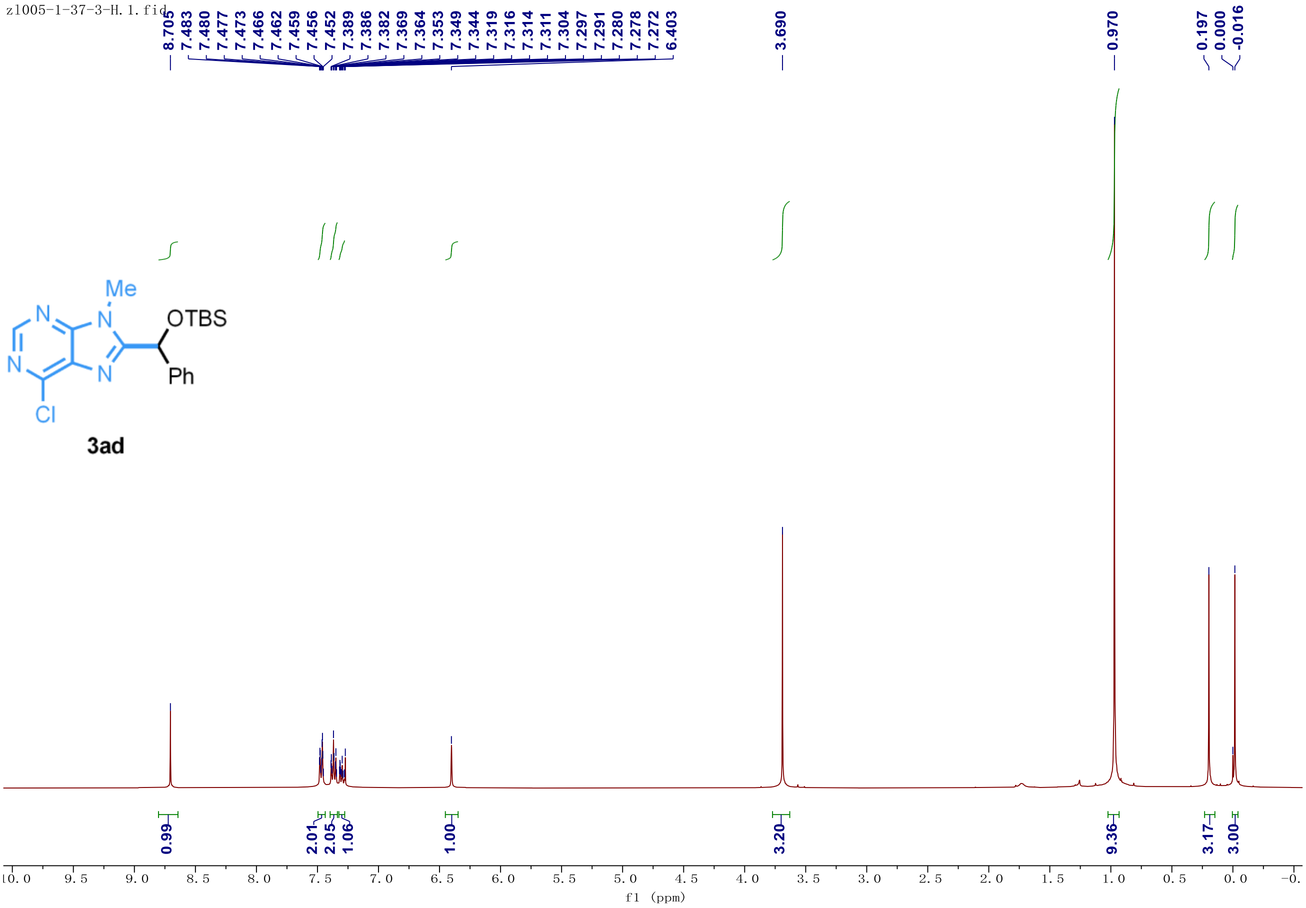

zl005-1-37-3-H.1.fid
8.705
7.483
7.480
7.477
7.473
7.466
7.462
7.459
7.456
7.452
7.389
7.386
7.382
7.369
7.364
7.353
7.349
7.344
7.319
7.316
7.314
7.311
7.304
7.297
7.291
7.280
7.278
7.272
6.403
3.690
0.970
0.197
0.000
-0.016
Me
N
N
OTBS
N
N
Ph
Cl
3ad
0.99
2.01
2.05
1.06
1.00
3.20
9.36
3.17
3.00
10.0 9.5 9.0 8.5 8.0 7.5 7.0 6.5 6.0 5.5 5.0 4.5 4.0 3.5 3.0 2.5 2.0 1.5 1.0 0.5 0.0 -0.
f1 (ppm)

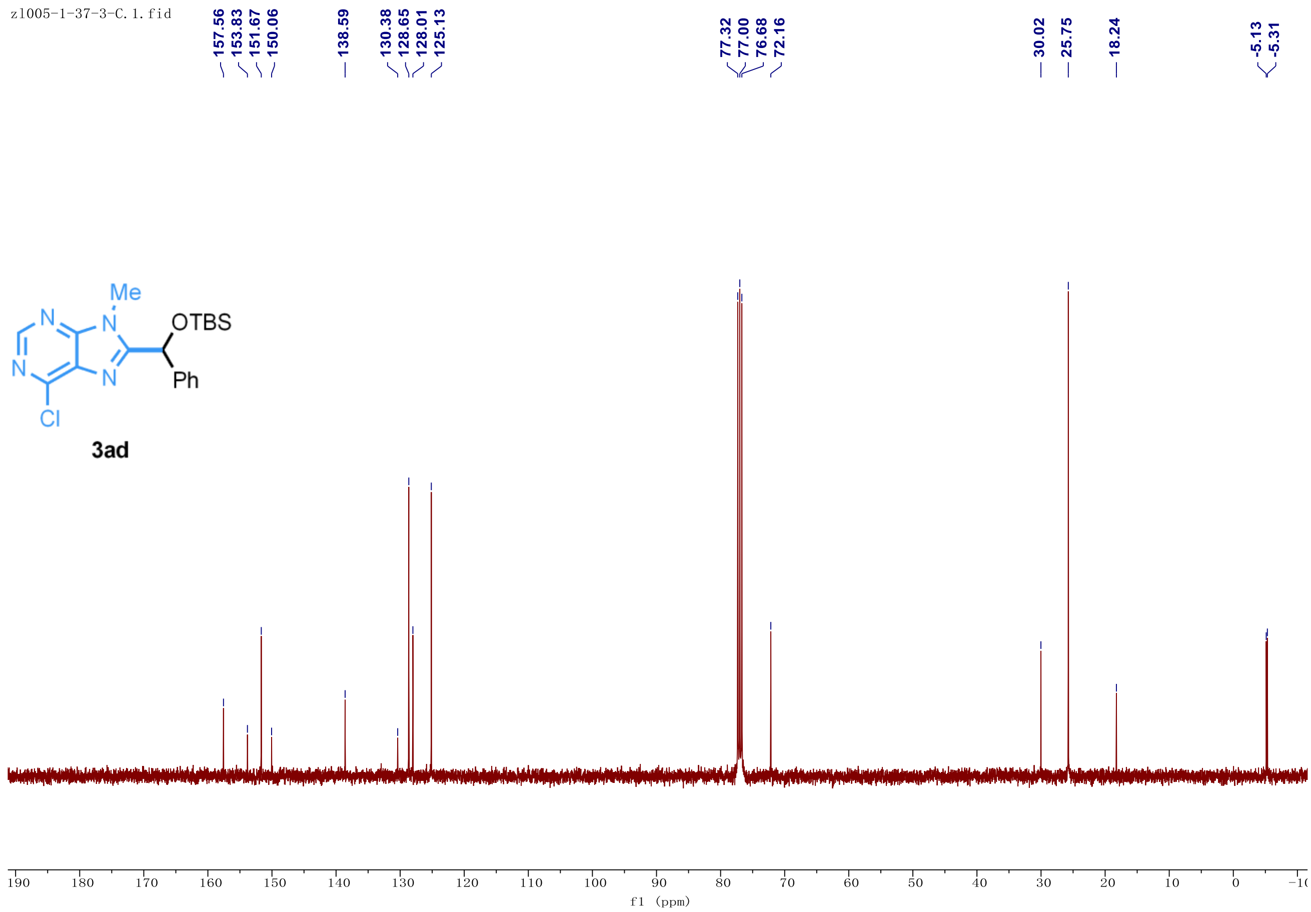
zl005-1-37-3-C.1.fid
157.56
153.83
151.67
150.06
138.59
130.38
128.65
128.01
125.13
77.32
77.00
76.68
72.16
30.02
25.75
18.24
-5.13
-5.31
f1 (ppm)

3ad

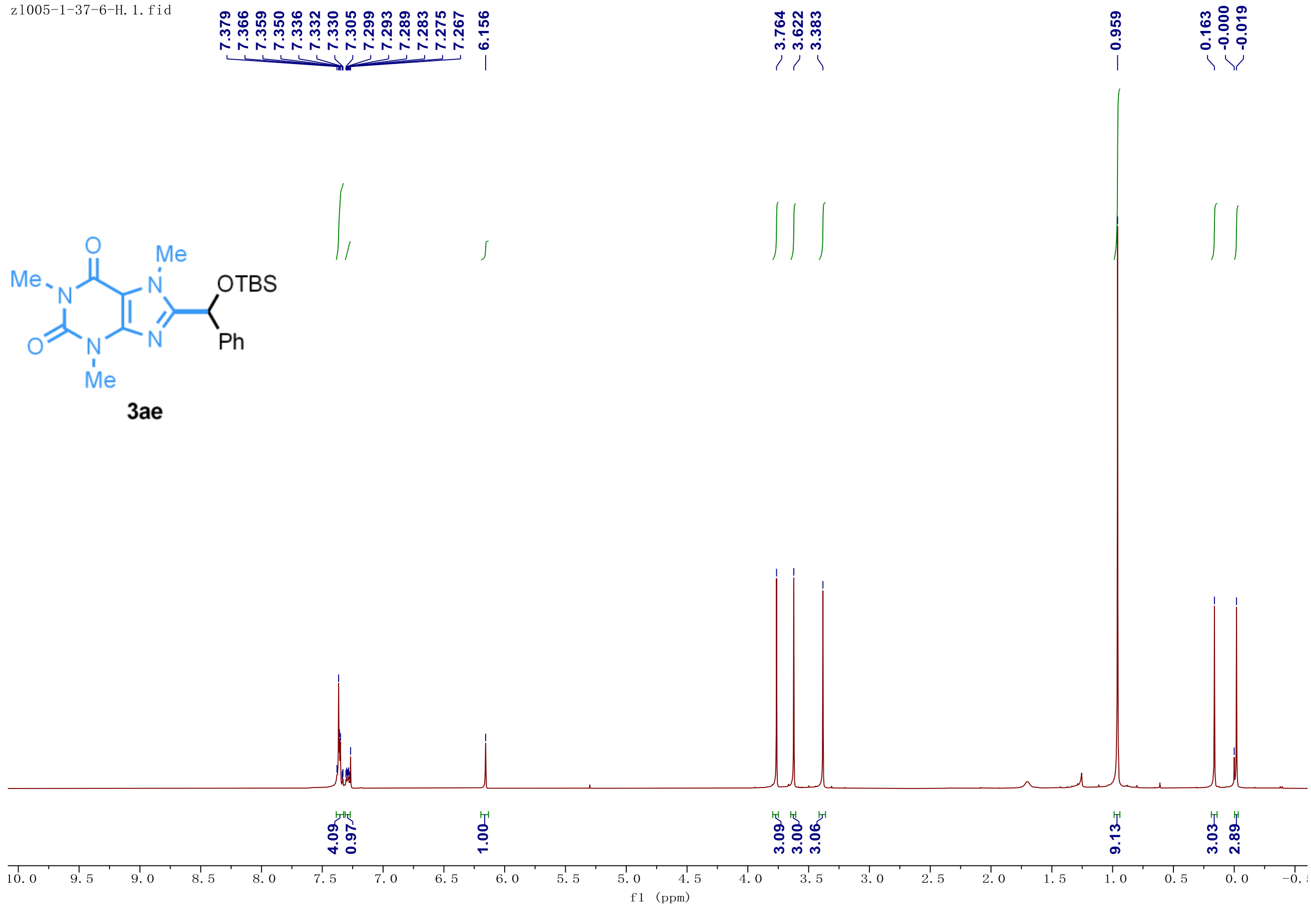
zl005-1-37-6-H.1.fid
7.379
7.366
7.359
7.350
7.336
7.332
7.330
7.305
7.299
7.293
7.289
7.283
7.275
7.267
6.156
3.764
3.622
3.383
0.959
0.163
-0.000
-0.019
4.09
0.97
1.00
3.09
3.00
3.06
9.13
3.03
2.89
f1 (ppm)

3ae

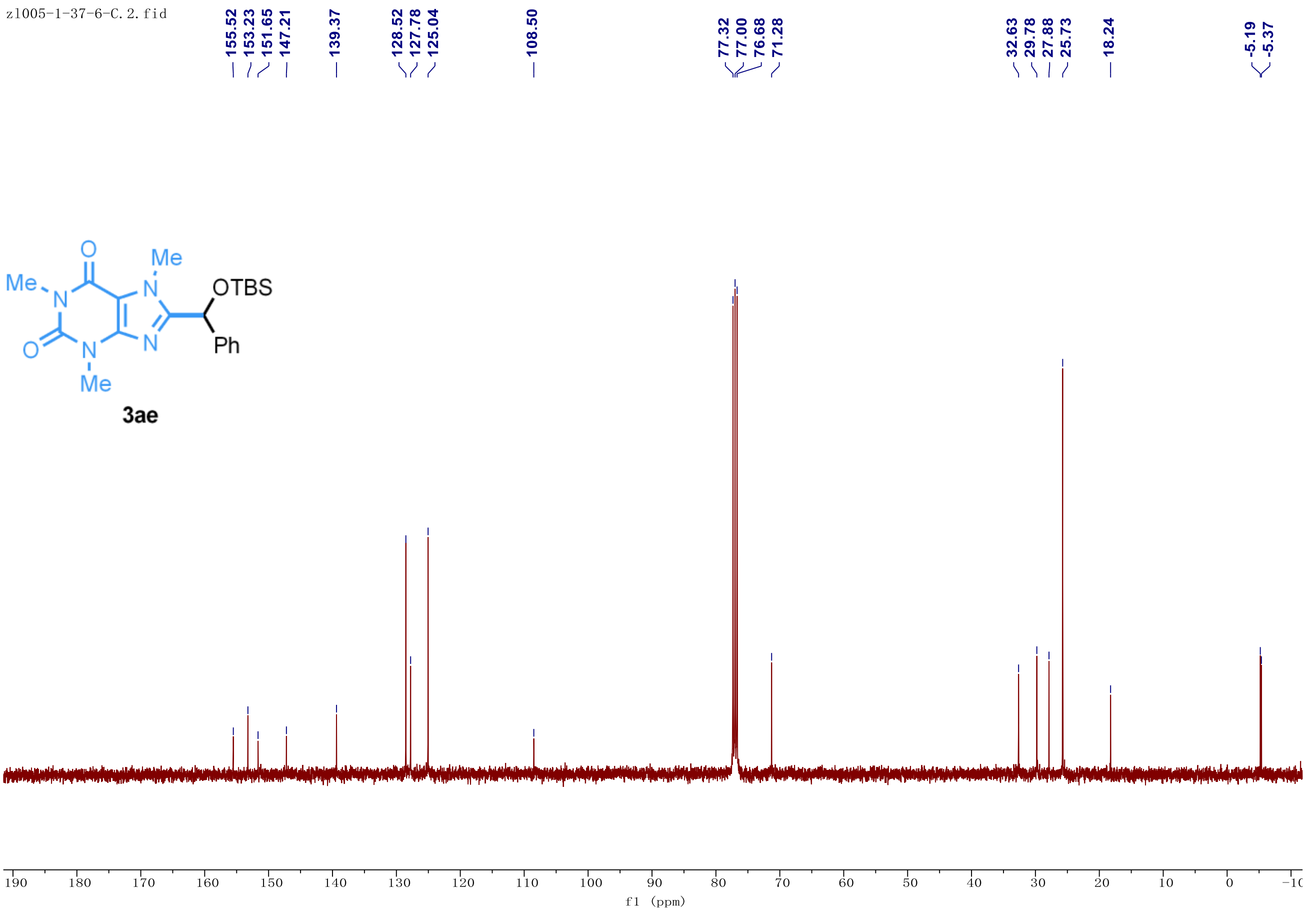
z1005-1-37-6-C.2.fid
155.52
153.23
151.65
147.21
139.37
128.52
127.78
125.04
108.50
77.32
77.00
76.68
71.28
32.63
29.78
27.88
25.73
18.24
-5.19
-5.37
Me
O
N
OTBS
Ph
3ae
f1 (ppm)

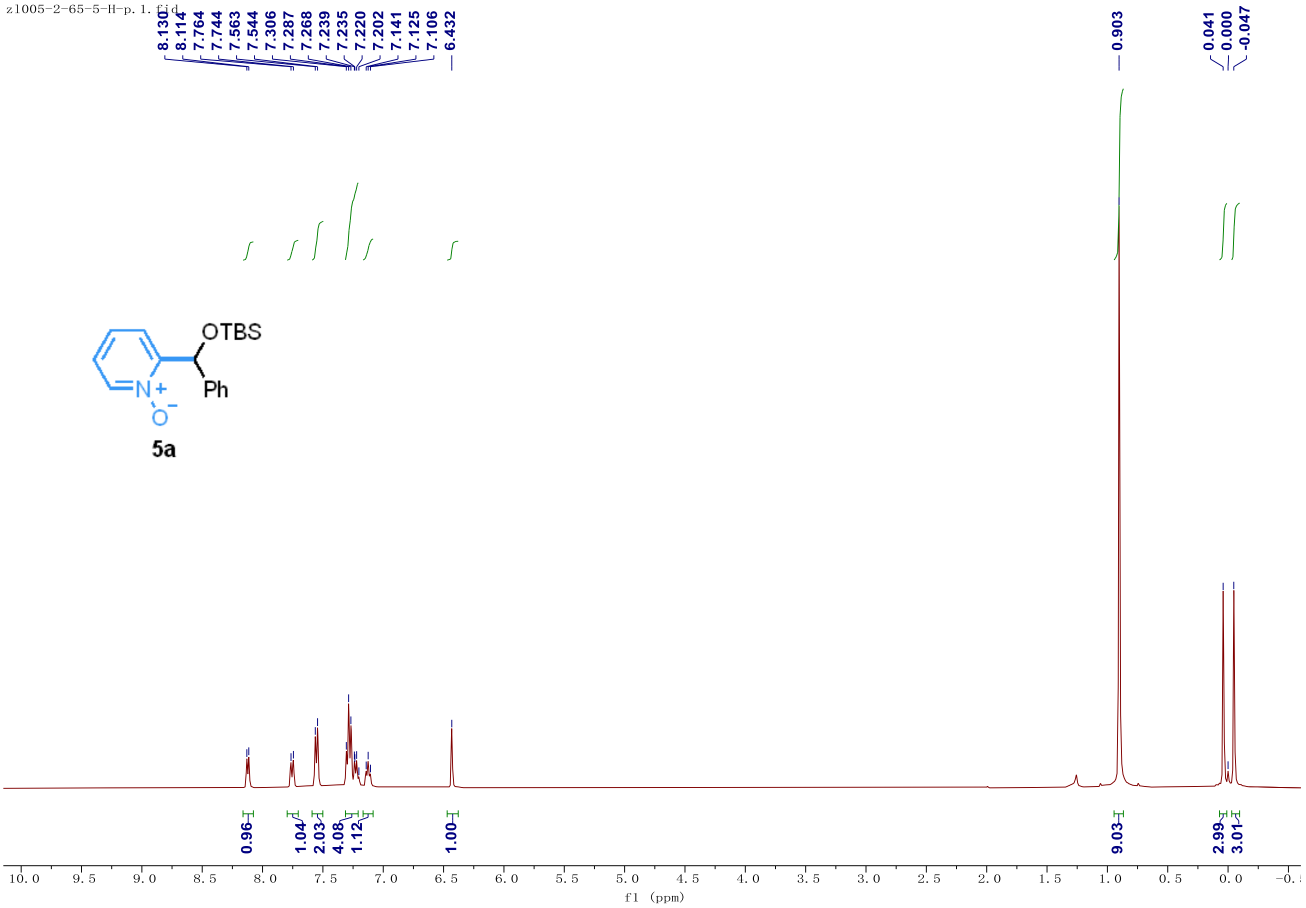
z1005-2-65-5-H-p.1.fid
8.130
8.114
7.764
7.744
7.563
7.544
7.306
7.287
7.268
7.239
7.235
7.220
7.202
7.141
7.125
7.106
6.432
0.903
0.041
0.000
-0.047
OTBS
Ph
N+
O-
5a
0.96
1.04
2.03
4.08
1.12
1.00
9.03
2.99
3.01
f1 (ppm)

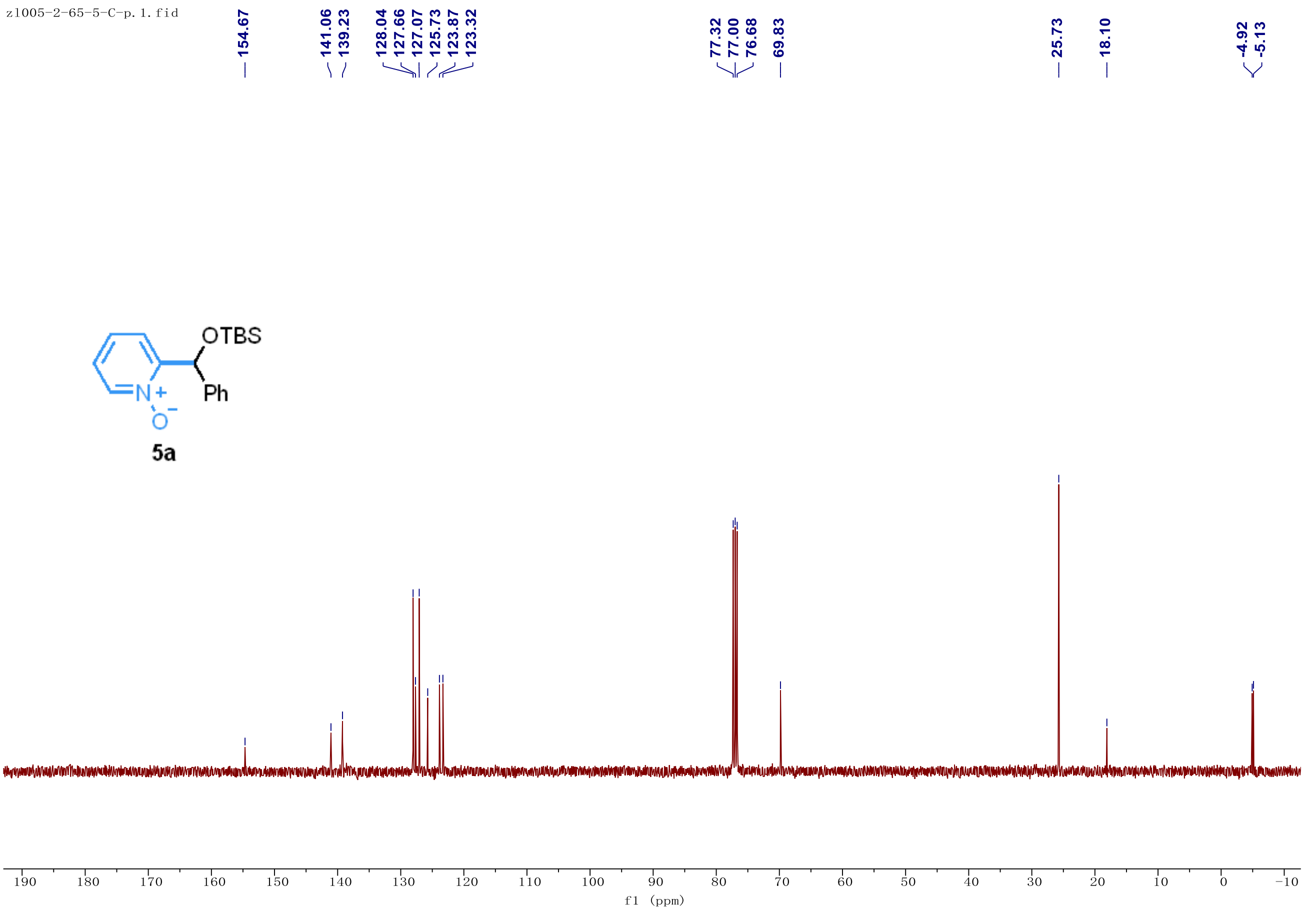
z1005-2-65-5-C-p.1.fid
154.67
141.06
139.23
128.04
127.66
127.07
125.73
123.87
123.32
77.32
77.00
76.68
69.83
25.73
18.10
-4.92
-5.13
OTBS
Ph
N+
O-
5a
f1 (ppm)

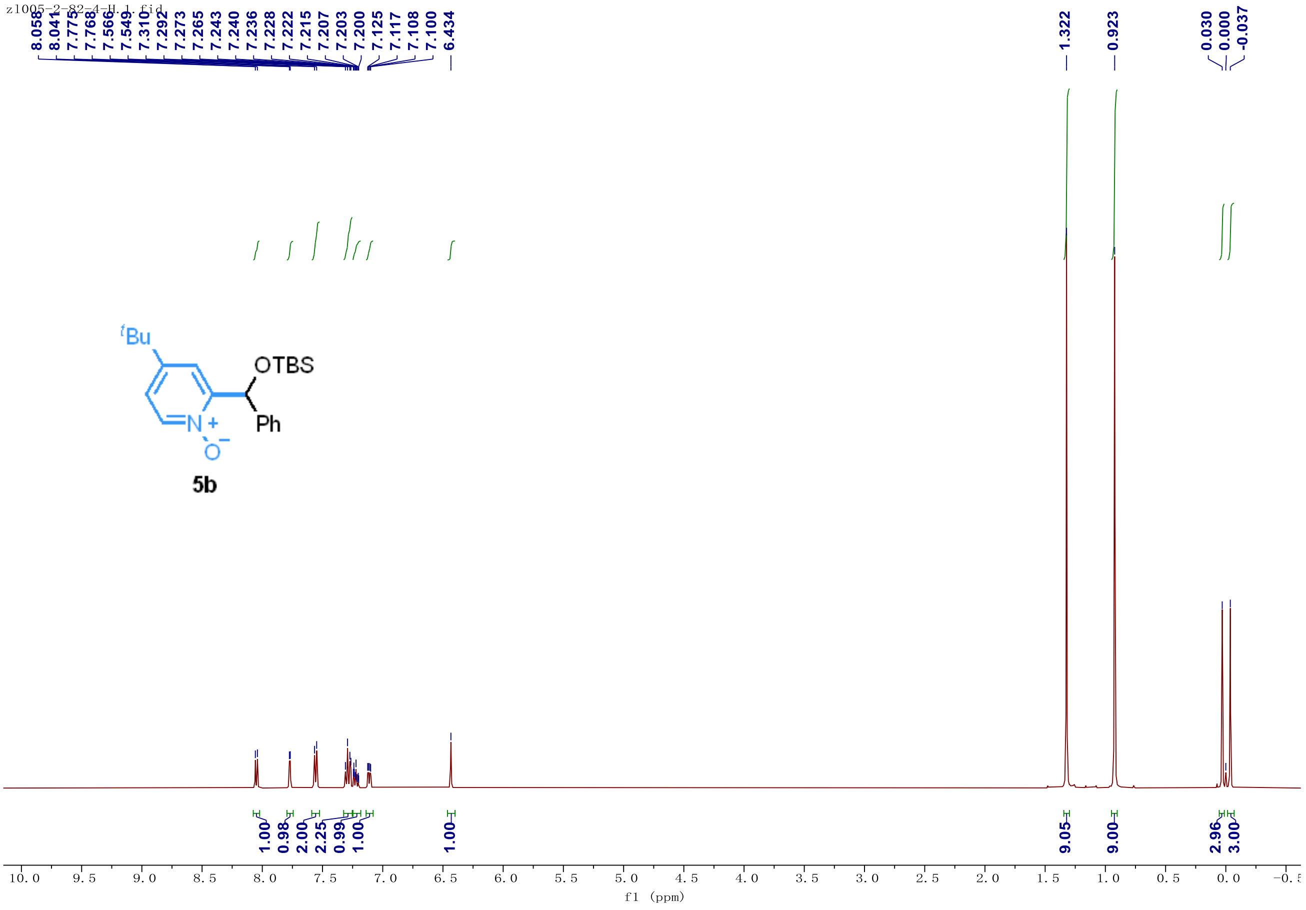
8.058
8.041
7.775
7.768
7.566
7.549
7.310
7.292
7.273
7.265
7.243
7.240
7.236
7.228
7.222
7.215
7.207
7.203
7.200
7.125
7.117
7.108
7.100
6.434
1.322
0.923
0.030
0.000
-0.037
1.00
0.98
2.00
2.25
0.99
1.00
1.00
9.05
9.00
2.96
3.00
tBu
OTBS
Ph
N+
O-
5b
f1 (ppm)

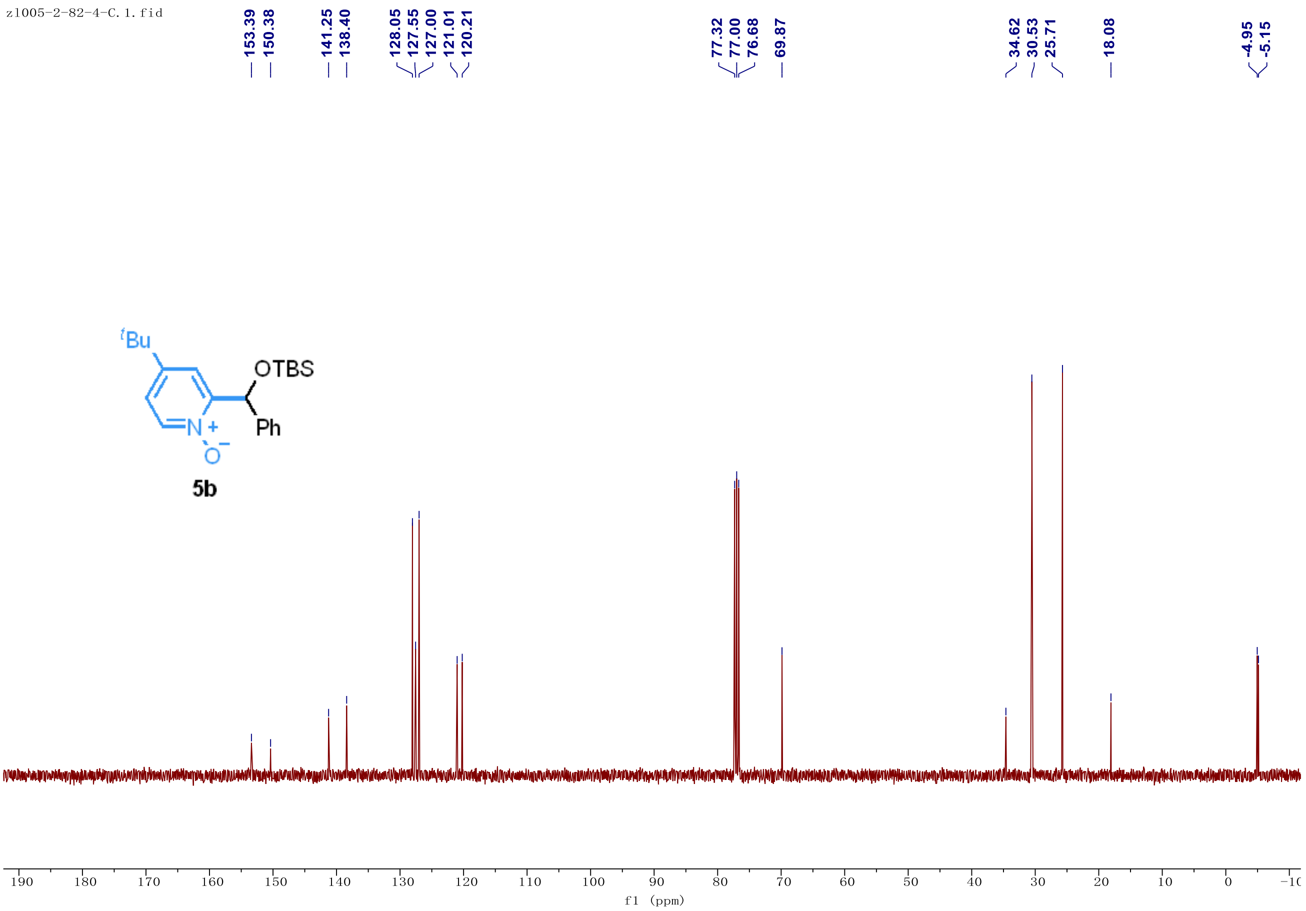
z1005-2-82-4-C.1.fid
153.39
150.38
141.25
138.40
128.05
127.55
127.00
121.01
120.21
77.32
77.00
76.68
69.87
34.62
30.53
25.71
18.08
-4.95
-5.15
tBu
OTBS
N+
O−
Ph
5b
190 180 170 160 150 140 130 120 110 100 90 80 70 60 50 40 30 20 10 0 -10
f1 (ppm)

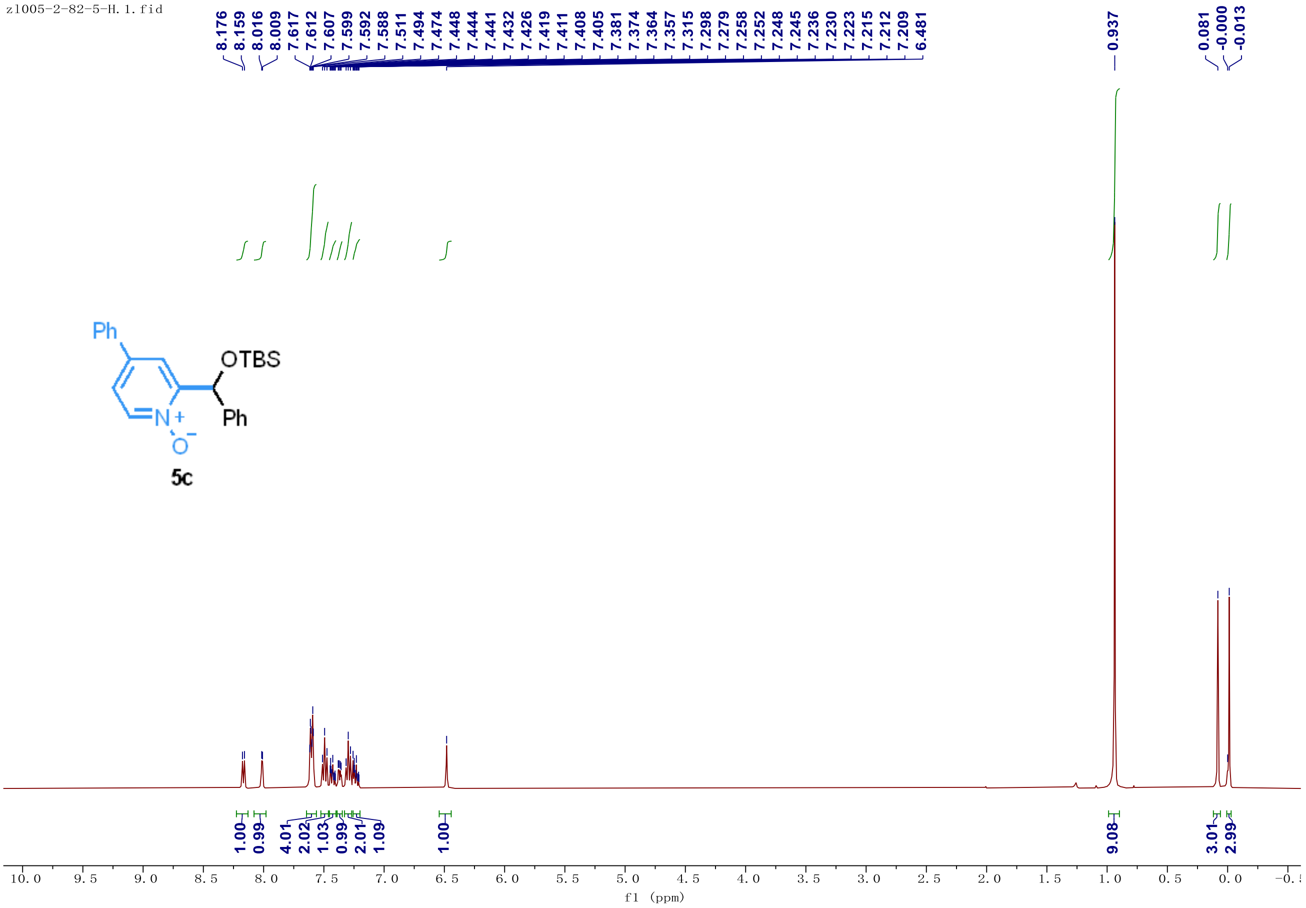
z1005-2-82-5-H.1.fid
8.176
8.159
8.016
8.009
7.617
7.612
7.607
7.599
7.592
7.588
7.511
7.494
7.474
7.448
7.444
7.441
7.432
7.426
7.419
7.411
7.408
7.405
7.381
7.374
7.364
7.357
7.315
7.298
7.279
7.258
7.252
7.248
7.245
7.236
7.230
7.223
7.215
7.212
7.209
6.481
0.937
0.081
-0.000
-0.013
Ph
OTBS
N+
O−
Ph
5c
1.00
0.99
4.01
2.02
1.03
0.99
2.01
1.09
1.00
9.08
3.01
2.99
10.0 9.5 9.0 8.5 8.0 7.5 7.0 6.5 6.0 5.5 5.0 4.5 4.0 3.5 3.0 2.5 2.0 1.5 1.0 0.5 0.0 -0.5
f1 (ppm)

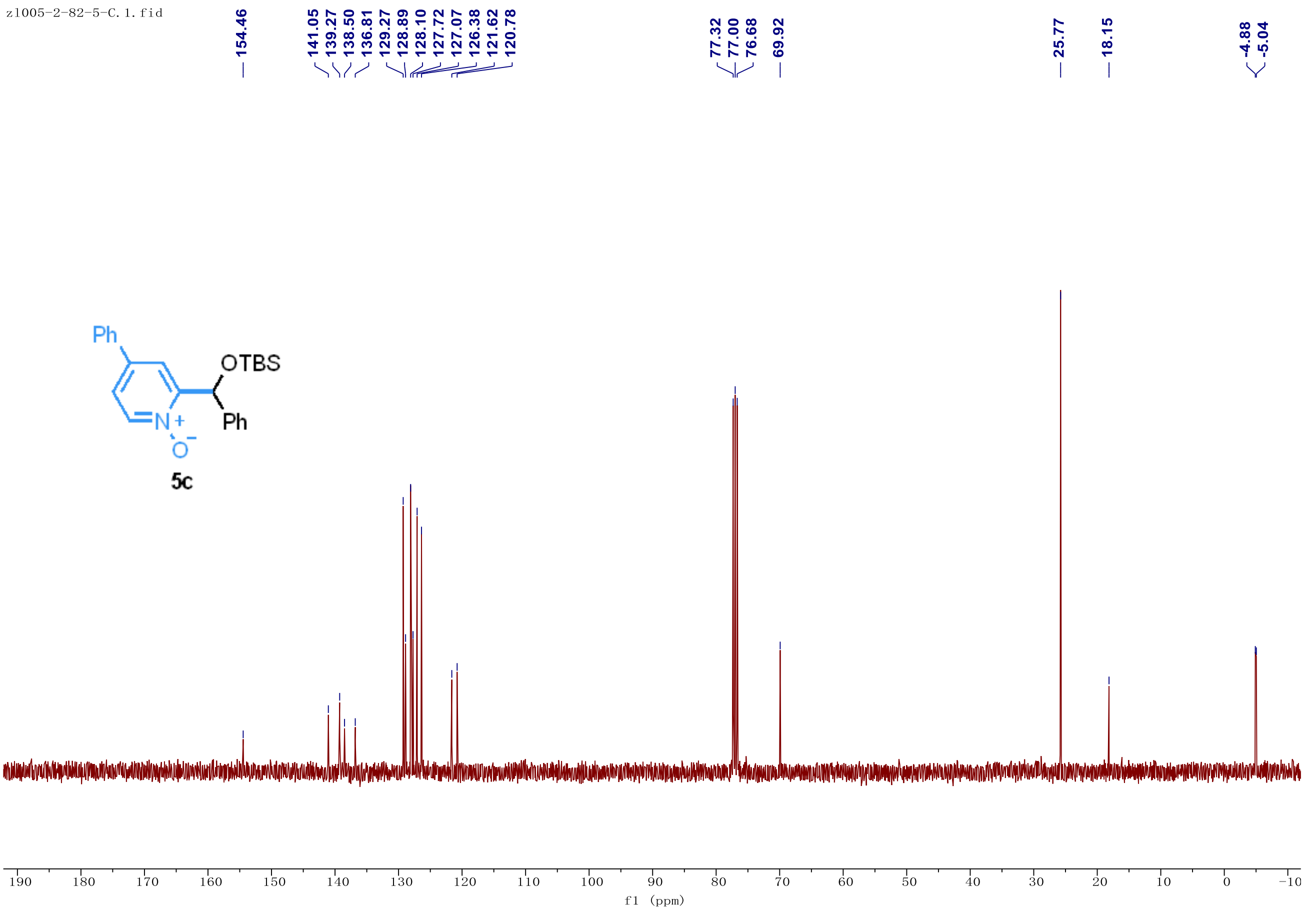
z1005-2-82-5-C.1.fid
154.46
141.05
139.27
138.50
136.81
129.27
128.89
128.10
127.72
127.07
126.38
121.62
120.78
77.32
77.00
76.68
69.92
25.77
18.15
-4.88
-5.04
Ph
OTBS
N+
O−
Ph
5c
f1 (ppm)

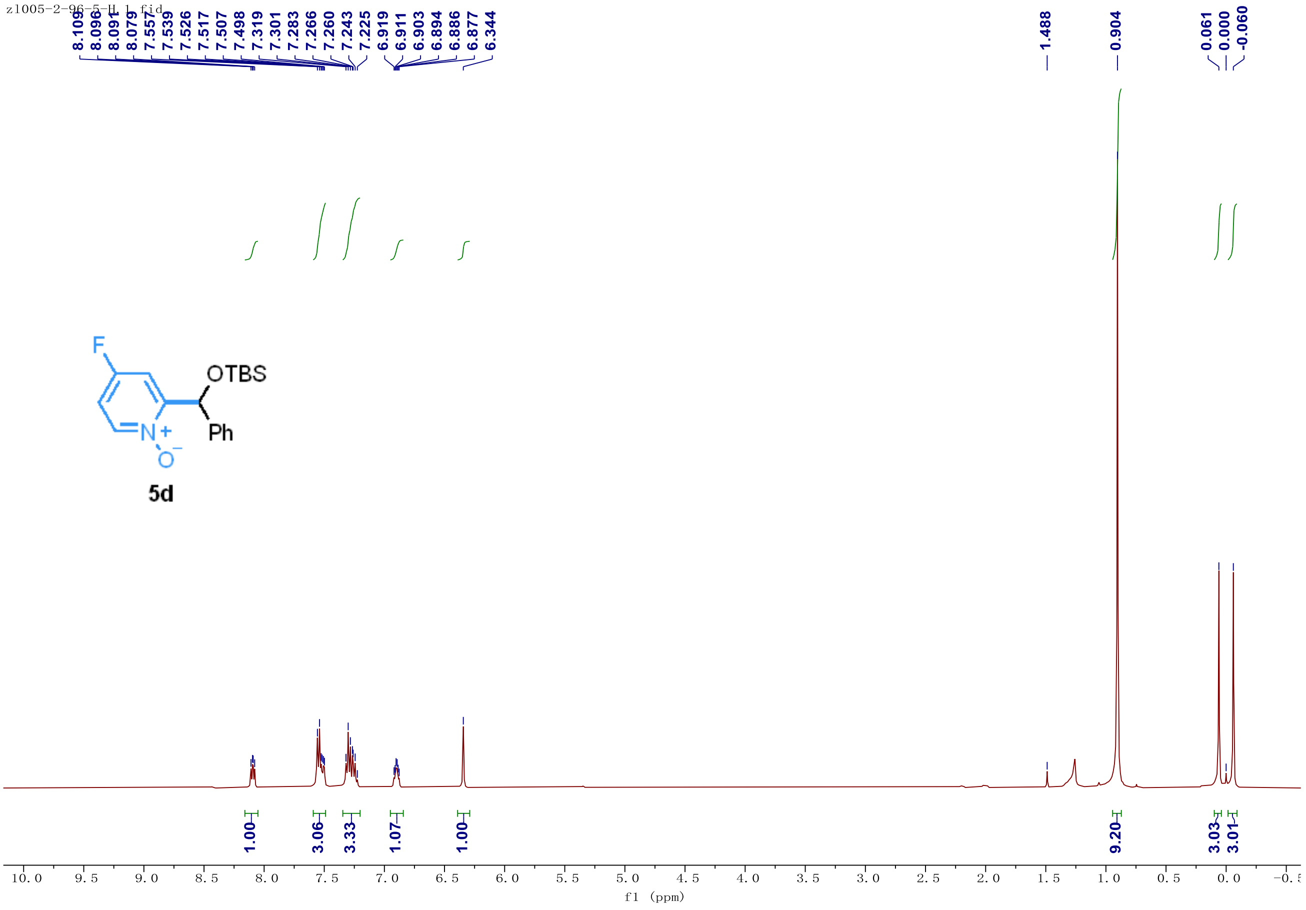
z1005-2-96-5-H.1.fid
8.109
8.096
8.091
8.079
7.557
7.539
7.526
7.517
7.507
7.498
7.319
7.301
7.283
7.266
7.260
7.243
7.225
6.919
6.911
6.903
6.894
6.886
6.877
6.344
1.488
0.904
0.061
0.000
-0.060
F
OTBS
N+
O−
Ph
5d
1.00
3.06
3.33
1.07
1.00
9.20
3.03
3.01
f1 (ppm)

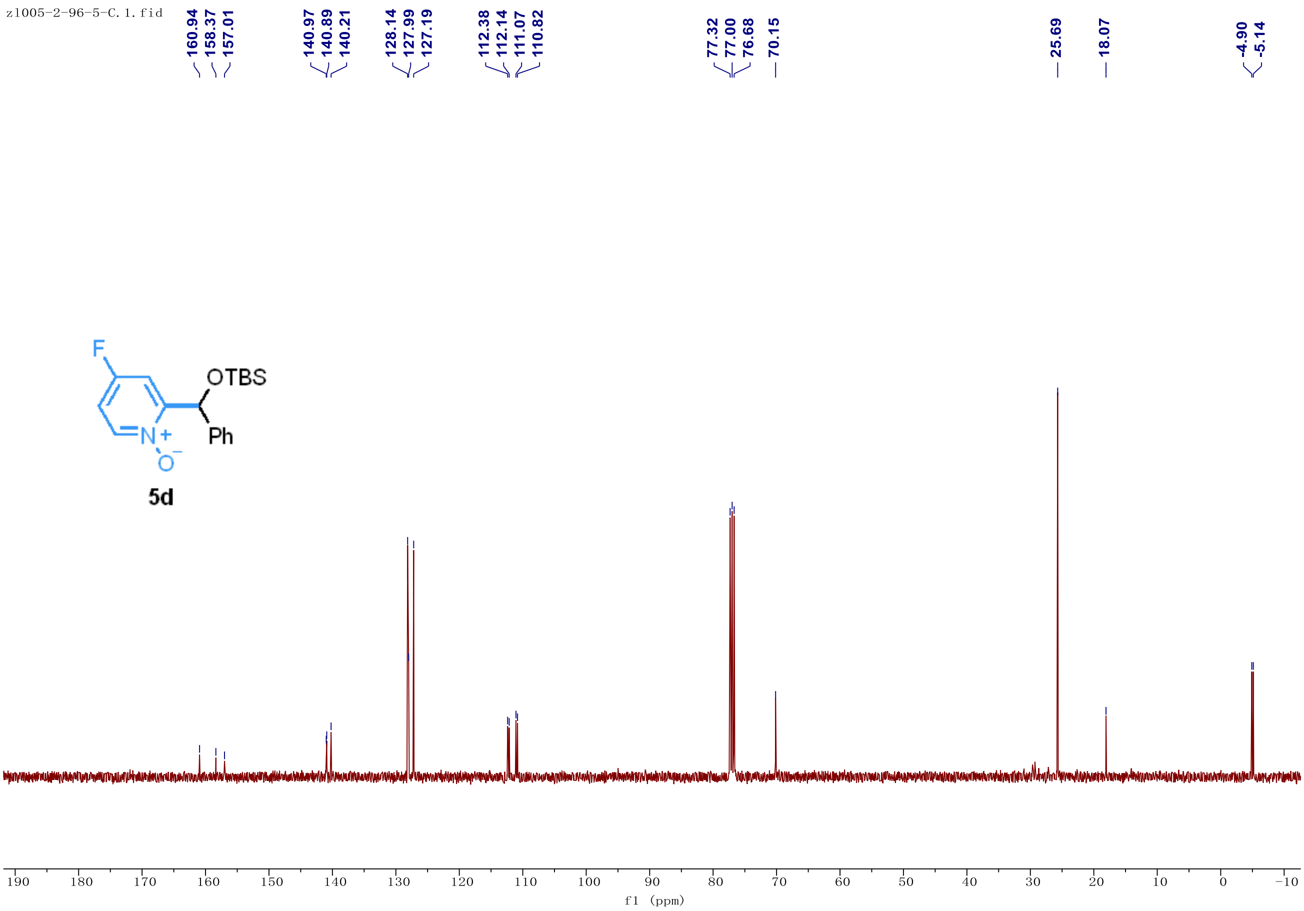
z1005-2-96-5-C.1.fid
160.94
158.37
157.01
140.97
140.89
140.21
128.14
127.99
127.19
112.38
112.14
111.07
110.82
77.32
77.00
76.68
70.15
25.69
18.07
-4.90
-5.14
F
OTBS
N+
O−
Ph
5d
190 180 170 160 150 140 130 120 110 100 90 80 70 60 50 40 30 20 10 0 −10
f1 (ppm)

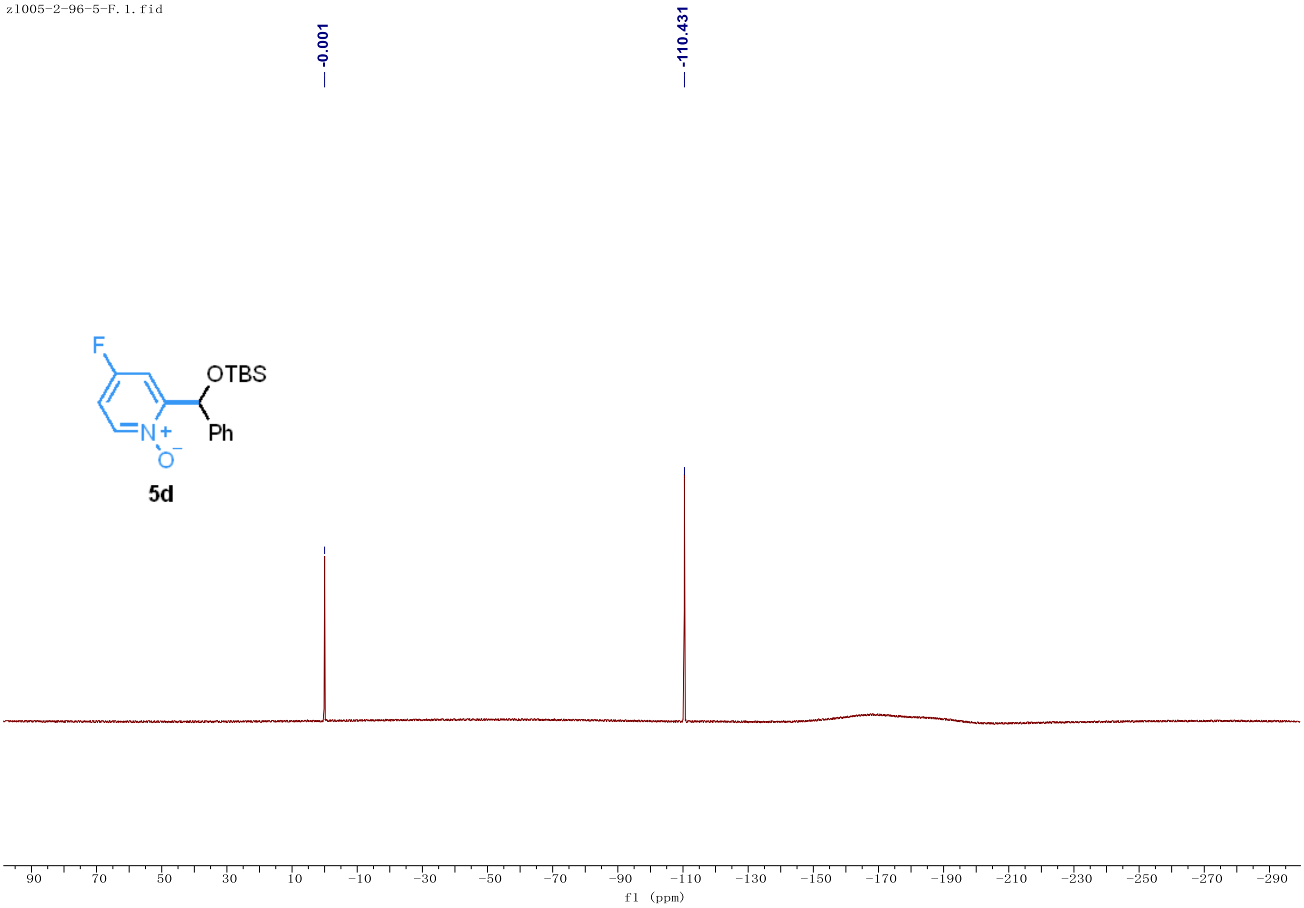
z1005-2-96-5-F.1.fid
-0.001
-110.431
F
OTBS
N+
O−
Ph
5d
90 70 50 30 10 −10 −30 −50 −70 −90 −110 −130 −150 −170 −190 −210 −230 −250 −270 −290
f1 (ppm)

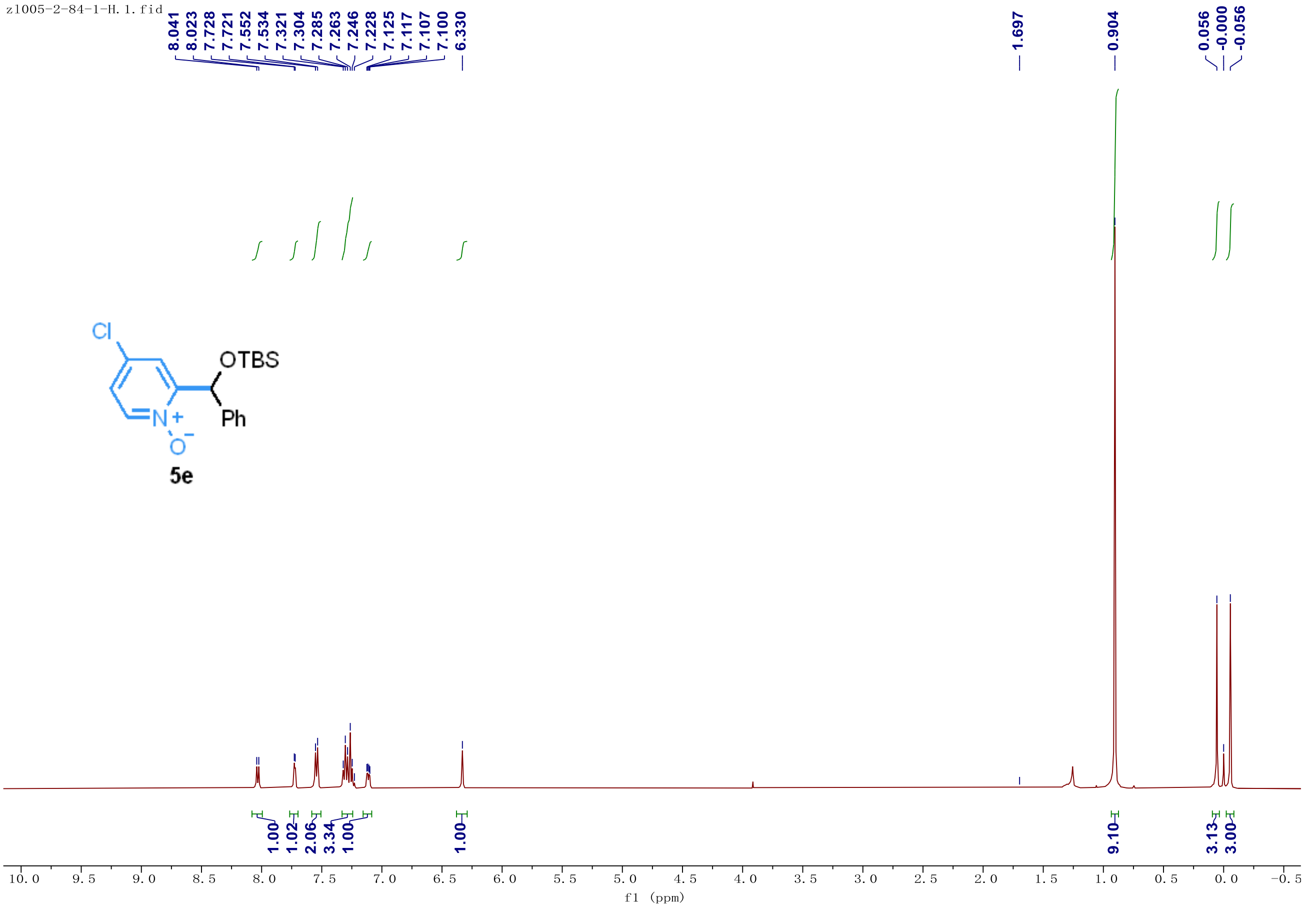
z1005-2-84-1-H.1.fid
8.041
8.023
7.728
7.721
7.552
7.534
7.321
7.304
7.285
7.263
7.246
7.228
7.125
7.117
7.107
7.100
6.330
1.697
0.904
0.056
-0.000
-0.056
Cl
OTBS
N+
O−
Ph
5e
1.00
1.02
2.06
3.34
1.00
1.00
9.10
3.13
3.00
f1 (ppm)

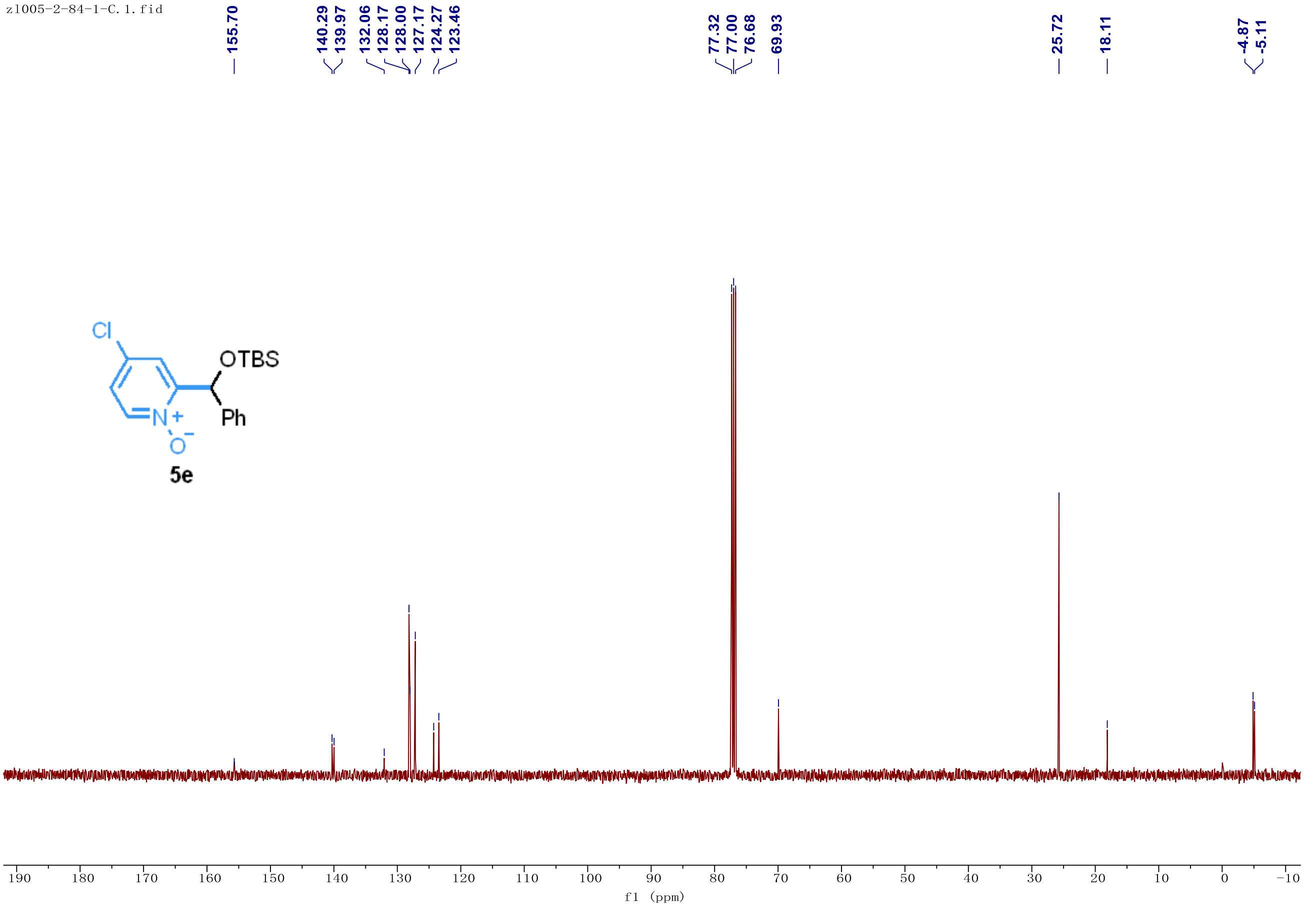
z1005-2-84-1-C.1.fid
155.70
140.29
139.97
132.06
128.17
128.00
127.17
124.27
123.46
77.32
77.00
76.68
69.93
25.72
18.11
-4.87
-5.11
Cl
OTBS
N+
O−
Ph
5e
f1 (ppm)

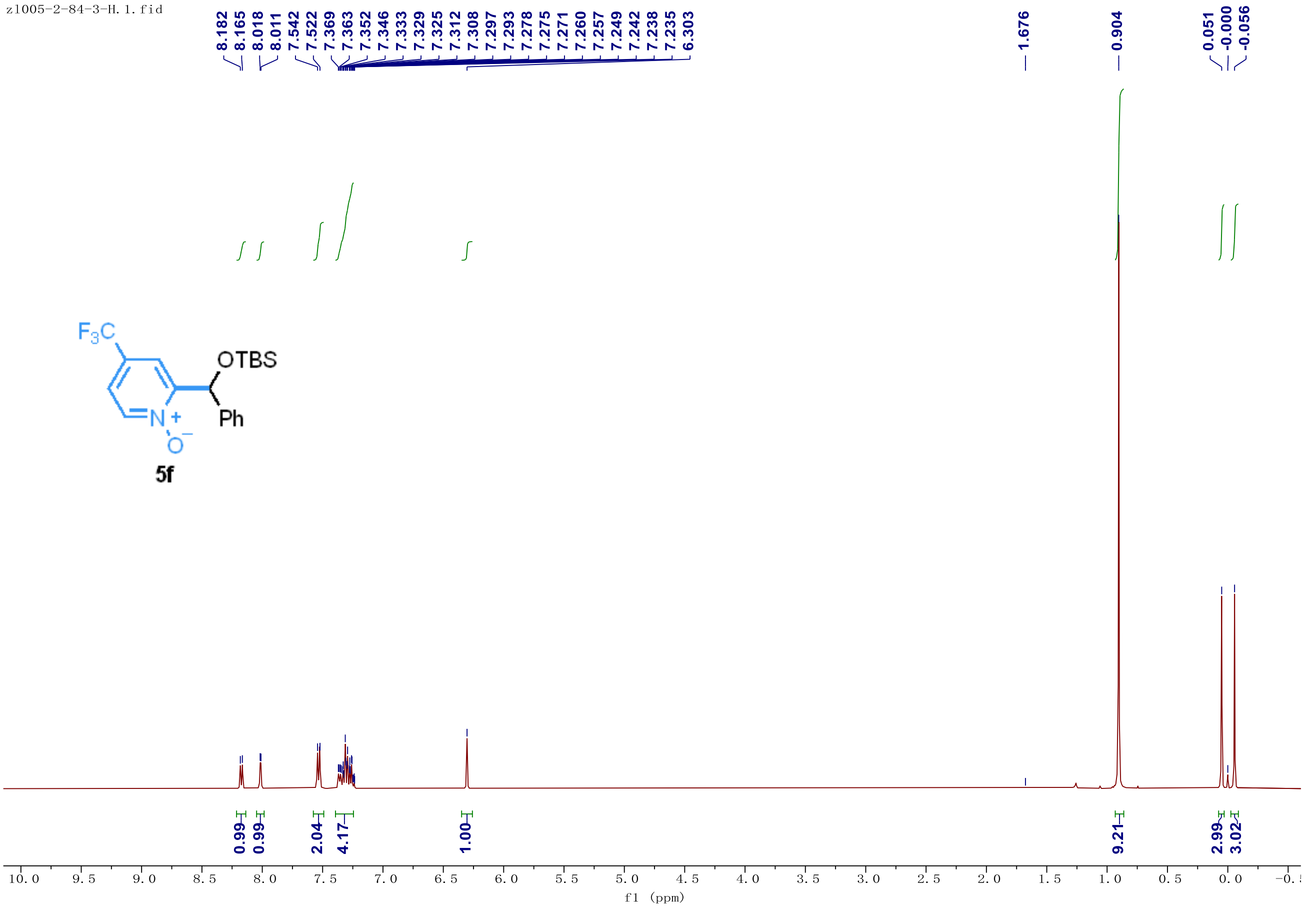
z1005-2-84-3-H.1.fid
8.182
8.165
8.018
8.011
7.542
7.522
7.369
7.363
7.352
7.346
7.333
7.329
7.325
7.312
7.308
7.297
7.293
7.278
7.275
7.271
7.260
7.257
7.249
7.242
7.238
7.235
6.303
1.676
0.904
0.051
-0.000
-0.056
F3C
OTBS
N+
O−
Ph
5f
0.99
0.99
2.04
4.17
1.00
9.21
2.99
3.02
f1 (ppm)

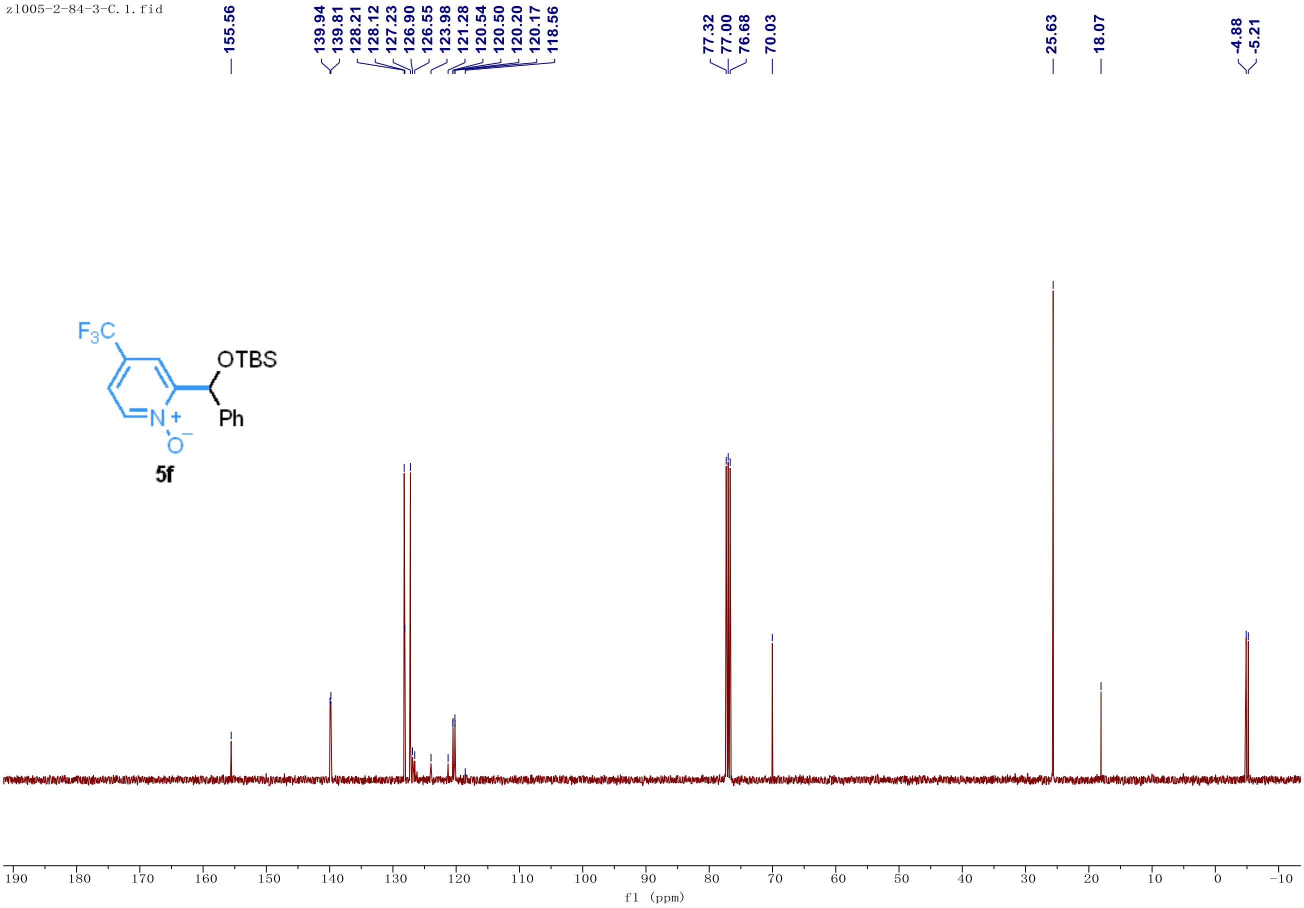
z1005-2-84-3-C.1.fid
155.56
139.94
139.81
128.21
128.12
127.23
126.90
126.55
123.98
121.28
120.54
120.50
120.20
120.17
118.56
77.32
77.00
76.68
70.03
25.63
18.07
-4.88
-5.21
F3C
OTBS
N+
O−
Ph
5f
f1 (ppm)

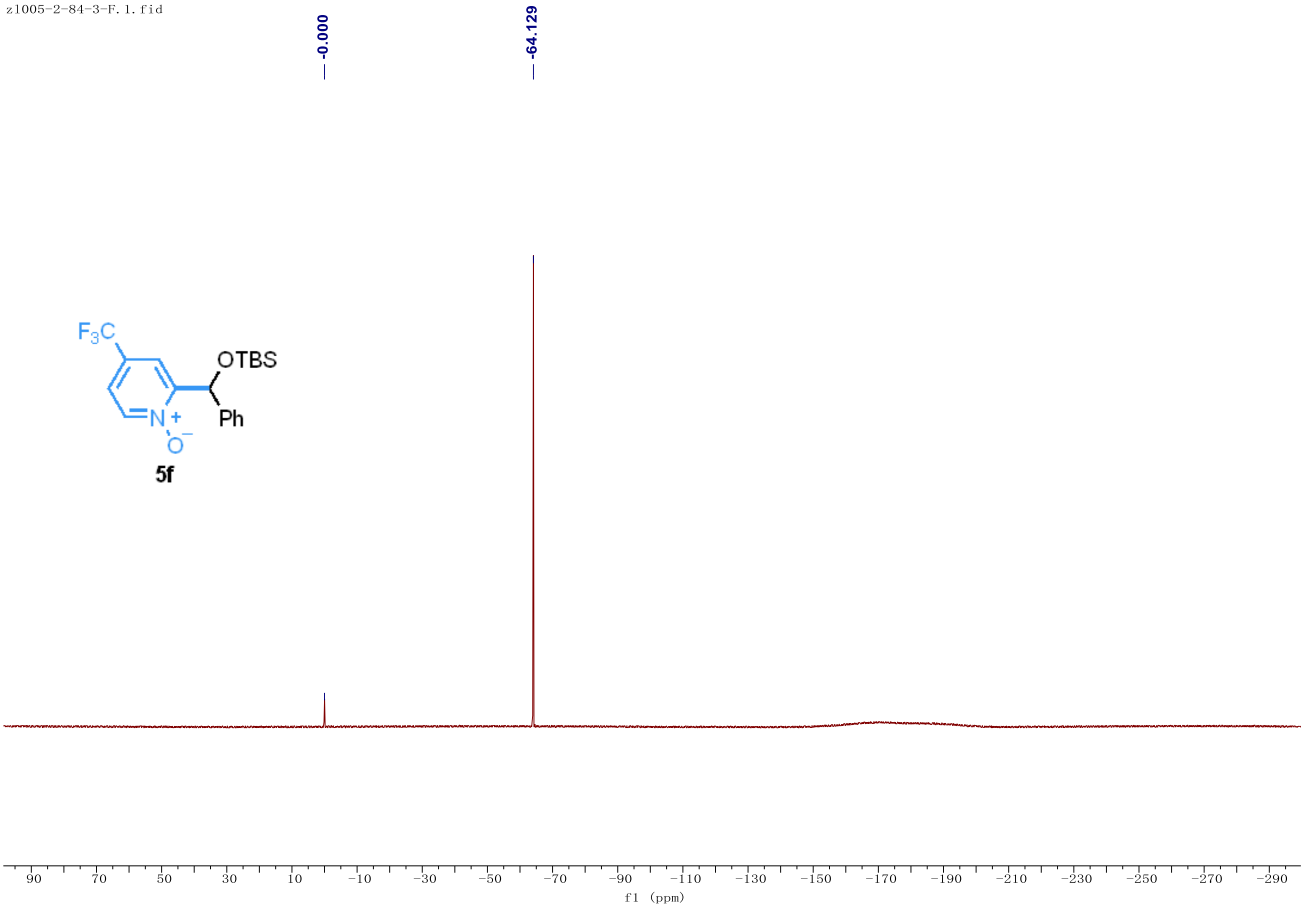
z1005-2-84-3-F.1.fid
-0.000
-64.129
F3C
OTBS
N+
O−
Ph
5f
90 70 50 30 10 −10 −30 −50 −70 −90 −110 −130 −150 −170 −190 −210 −230 −250 −270 −290
f1 (ppm)

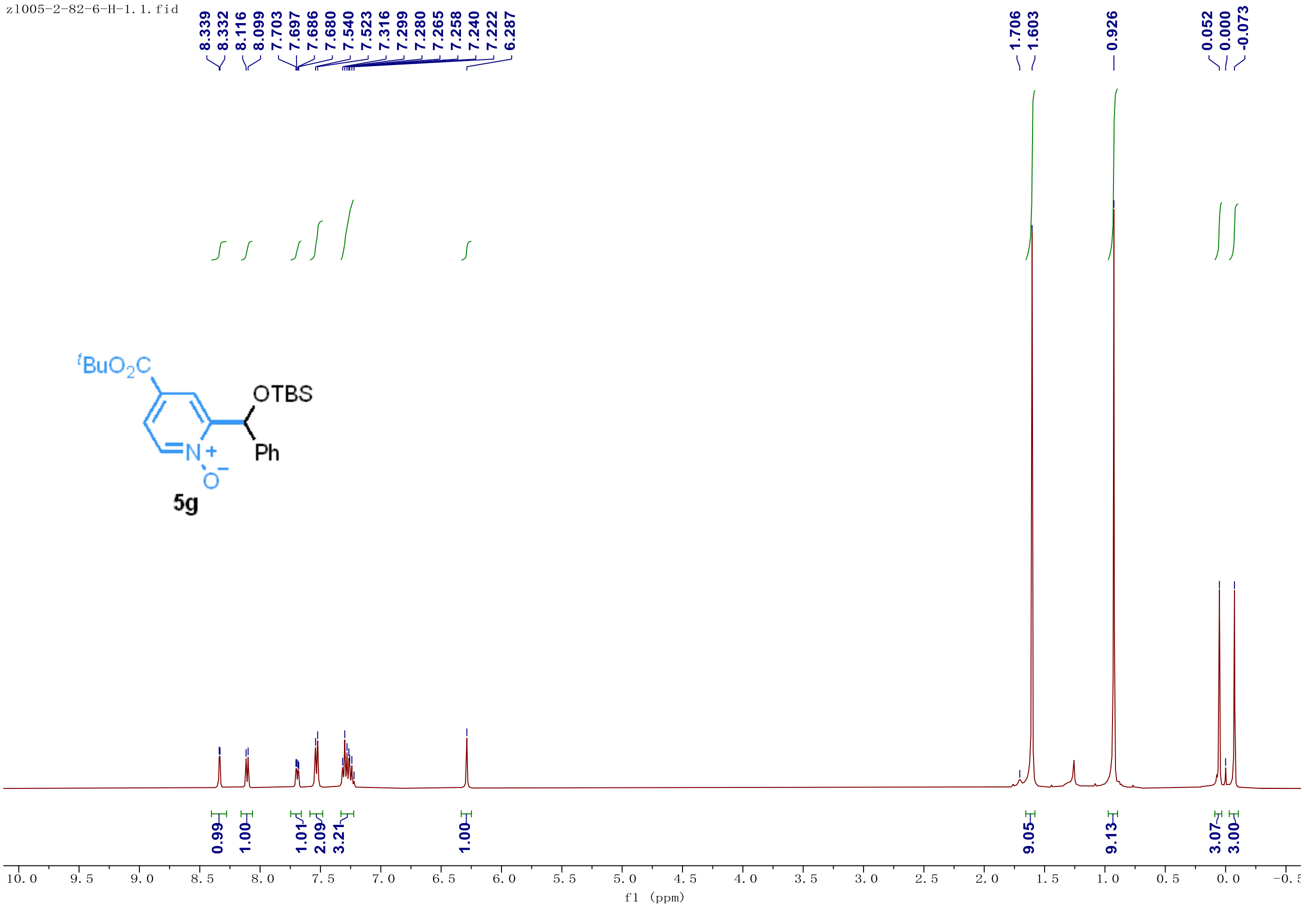
z1005-2-82-6-H-1.1.fid
8.339 8.332 8.116 8.099 7.703 7.697 7.686 7.680 7.540 7.523 7.316 7.299 7.280 7.265 7.258 7.240 7.222 6.287
1.706 1.603
0.926
0.052 0.000 -0.073
tBuO2C
OTBS
N+
O−
Ph
5g
0.99 1.00 1.01 2.09 3.21 1.00 9.05 9.13 3.07 3.00
10.0 9.5 9.0 8.5 8.0 7.5 7.0 6.5 6.0 5.5 5.0 4.5 4.0 3.5 3.0 2.5 2.0 1.5 1.0 0.5 0.0 -0.5
f1 (ppm)

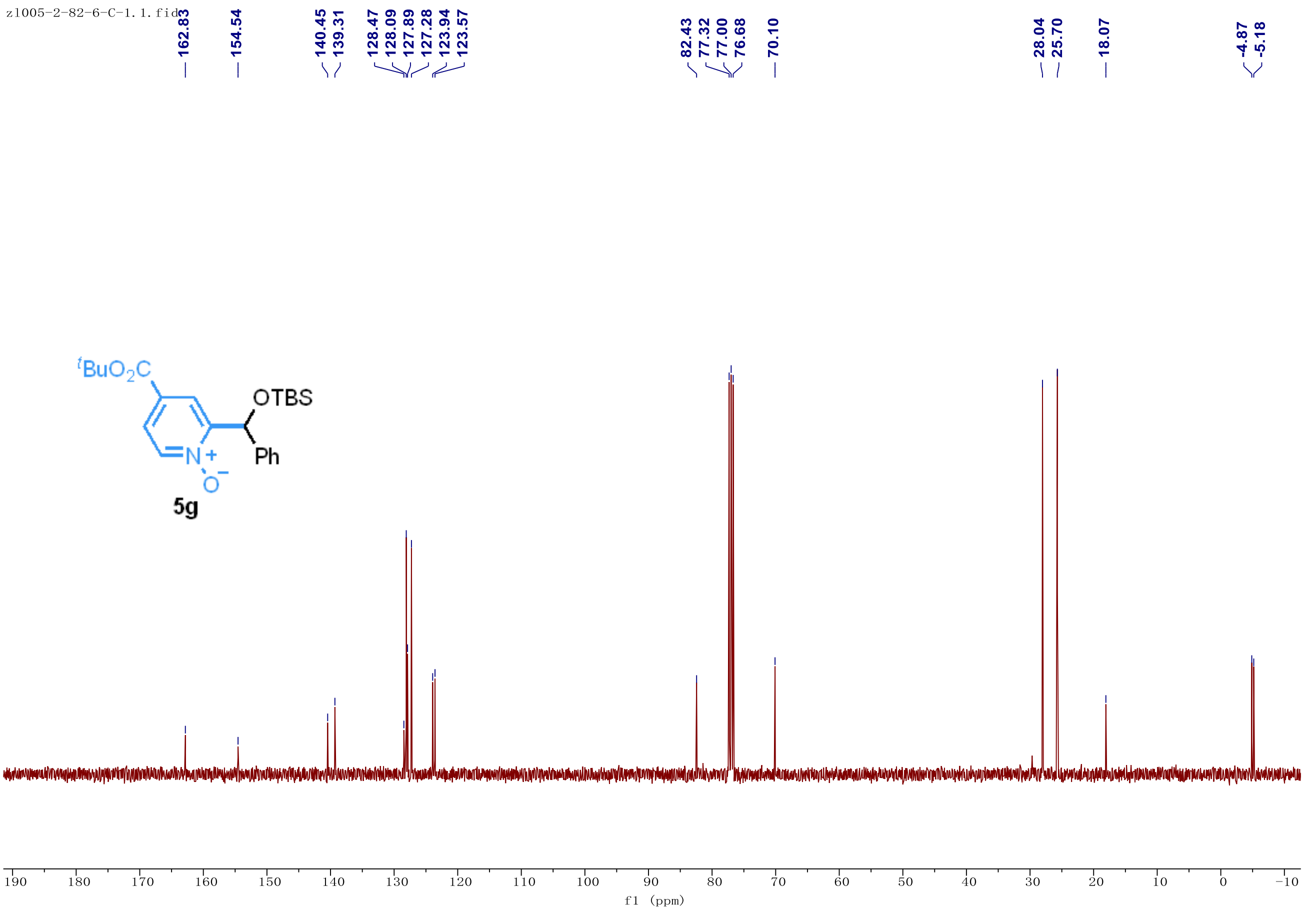

z1005-2-82-6-C-1.1.fid
162.83
154.54
140.45
139.31
128.47
128.09
127.89
127.28
123.94
123.57
82.43
77.32
77.00
76.68
70.10
28.04
25.70
18.07
-4.87
-5.18
$^{t}BuO_2C$
OTBS
N+
O−
Ph
5g
f1 (ppm)

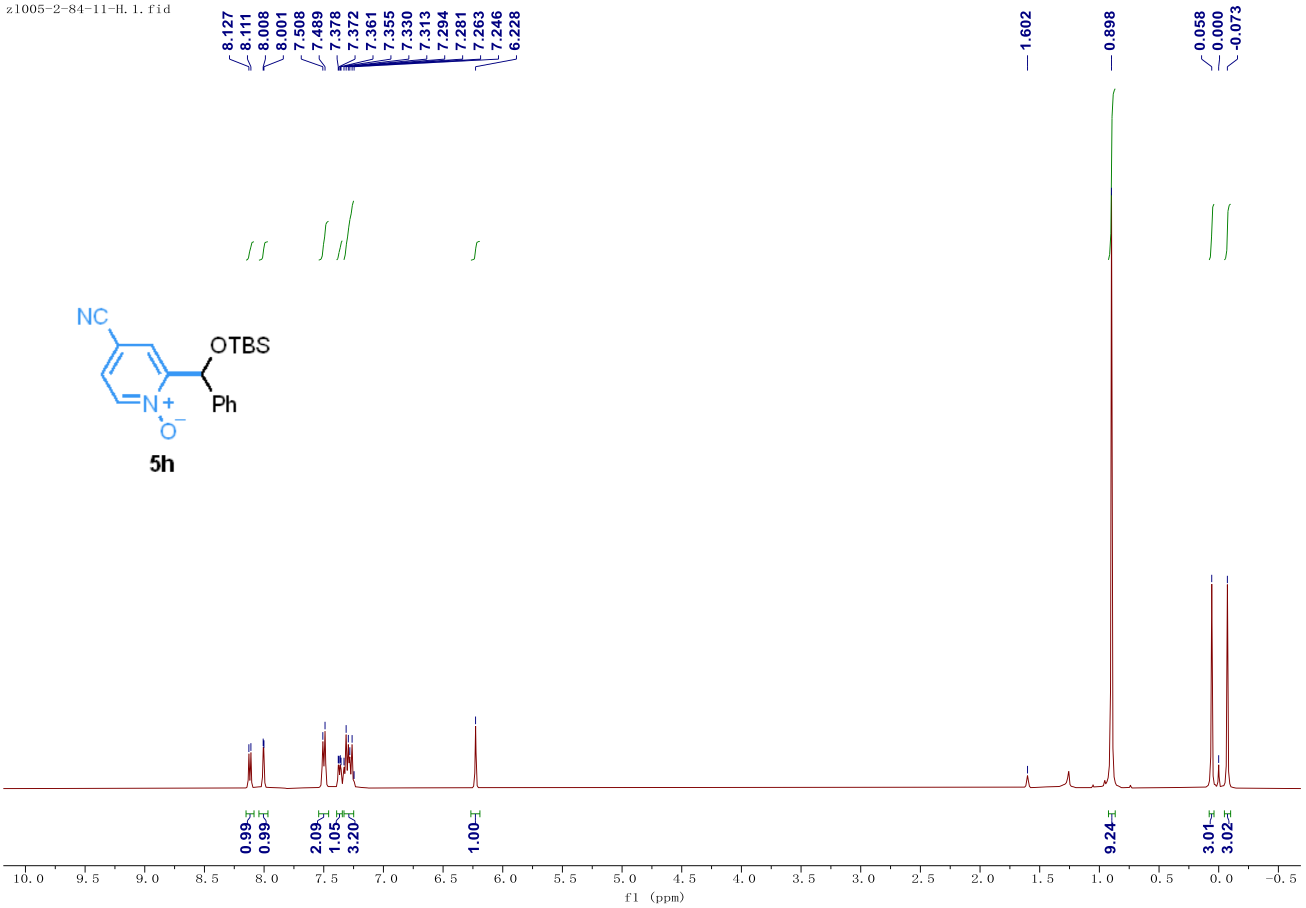

z1005-2-84-11-H.1.fid
8.127
8.111
8.008
8.001
7.508
7.489
7.378
7.372
7.361
7.355
7.330
7.313
7.294
7.281
7.263
7.246
6.228
1.602
0.898
0.058
0.000
-0.073
NC
OTBS
N+
O−
Ph
5h
0.99
0.99
2.09
1.05
3.20
1.00
9.24
3.01
3.02
f1 (ppm)

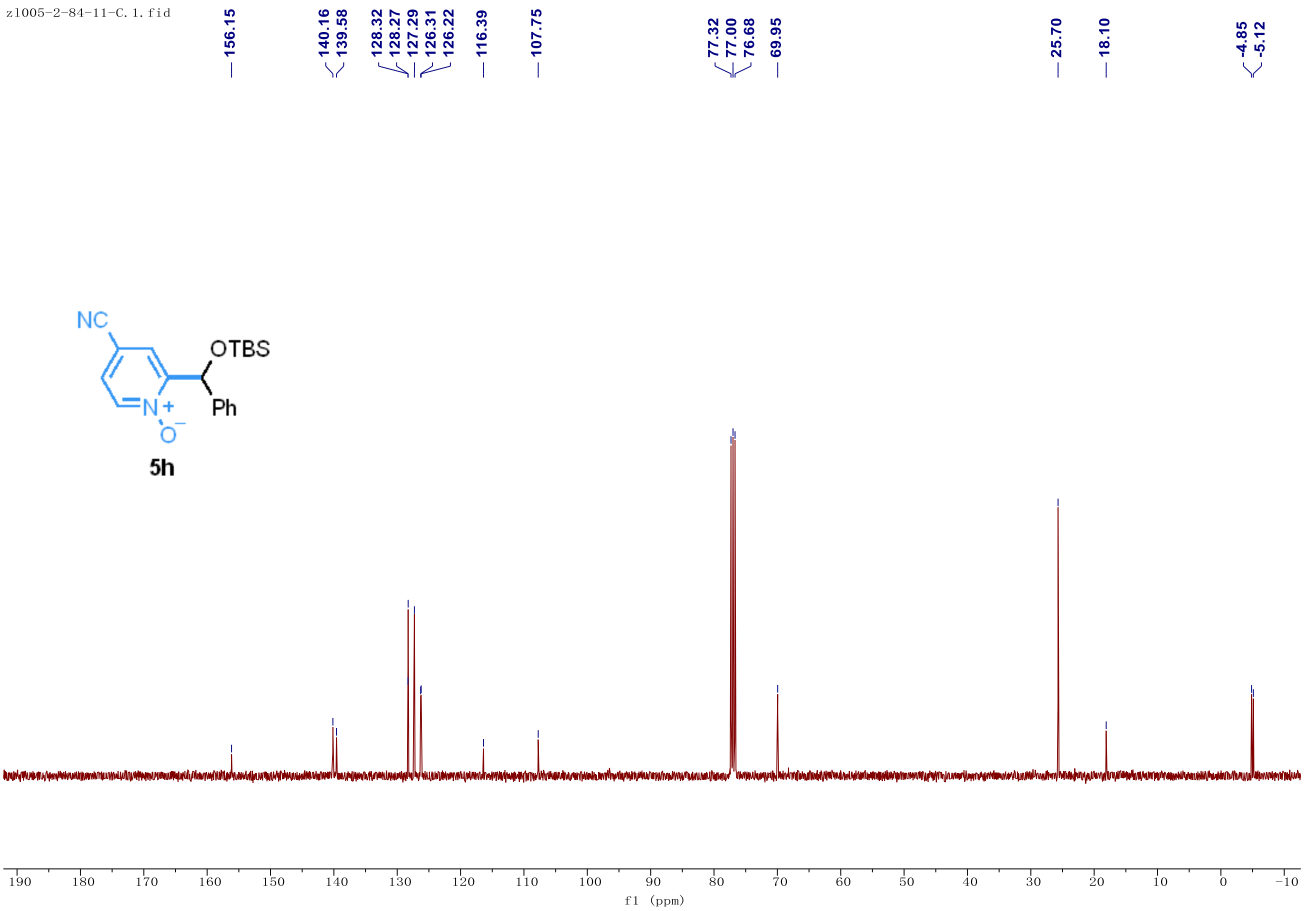
z1005-2-84-11-C.1.fid
156.15
140.16
139.58
128.32
128.27
127.29
126.31
126.22
116.39
107.75
77.32
77.00
76.68
69.95
25.70
18.10
-4.85
-5.12
NC
OTBS
N+
O−
Ph
5h
190 180 170 160 150 140 130 120 110 100 90 80 70 60 50 40 30 20 10 0 −10
f1 (ppm)

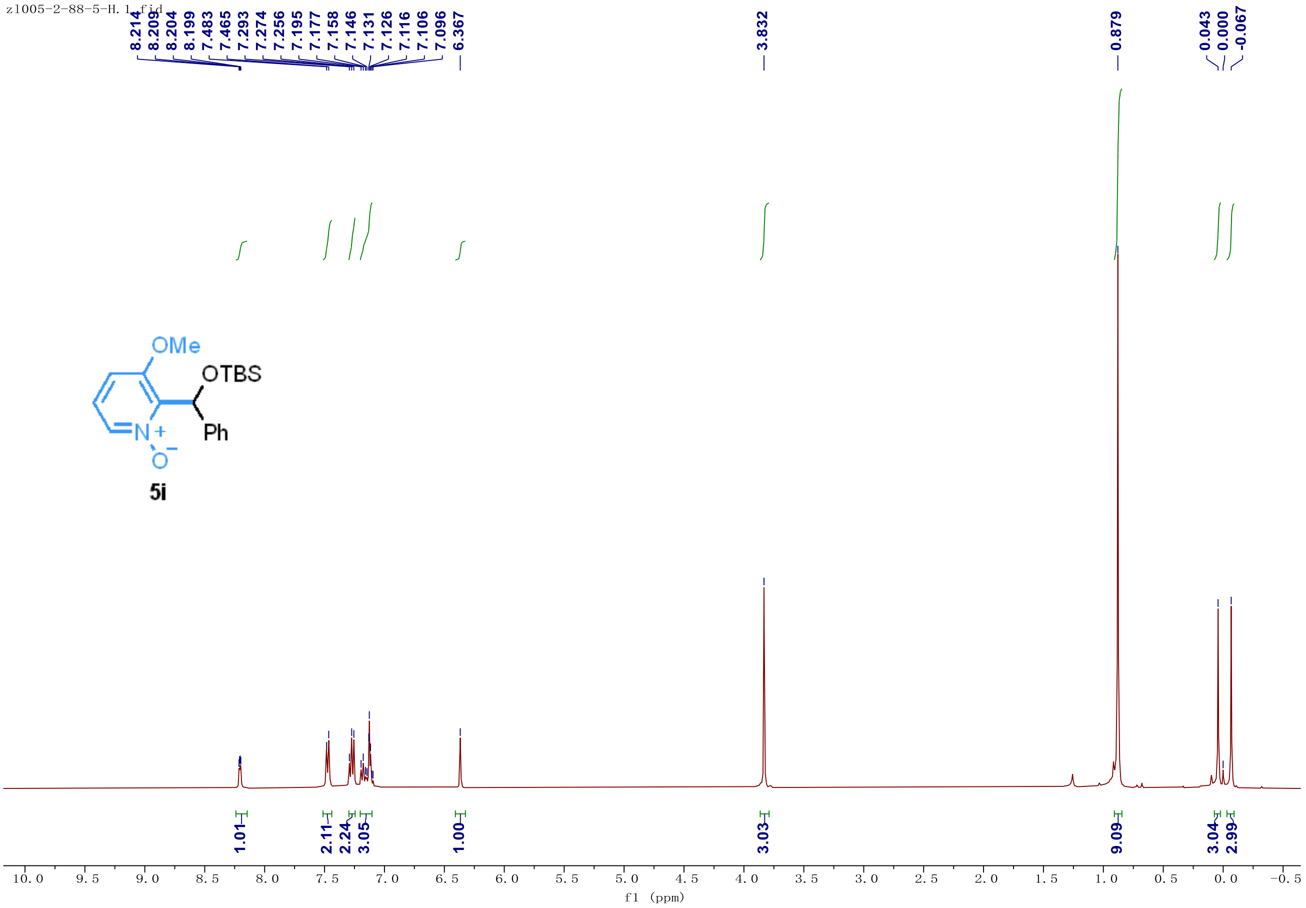
z1005-2-88-5-H.1.fid
8.214
8.209
8.204
8.199
7.483
7.465
7.293
7.274
7.256
7.195
7.177
7.158
7.146
7.131
7.126
7.116
7.106
7.096
6.367
3.832
0.879
0.043
0.000
-0.067
OMe
OTBS
N+
O−
Ph
5i
1.01
2.11
2.24
3.05
1.00
3.03
9.09
3.04
2.99
10.0 9.5 9.0 8.5 8.0 7.5 7.0 6.5 6.0 5.5 5.0 4.5 4.0 3.5 3.0 2.5 2.0 1.5 1.0 0.5 0.0 −0.5
f1 (ppm)

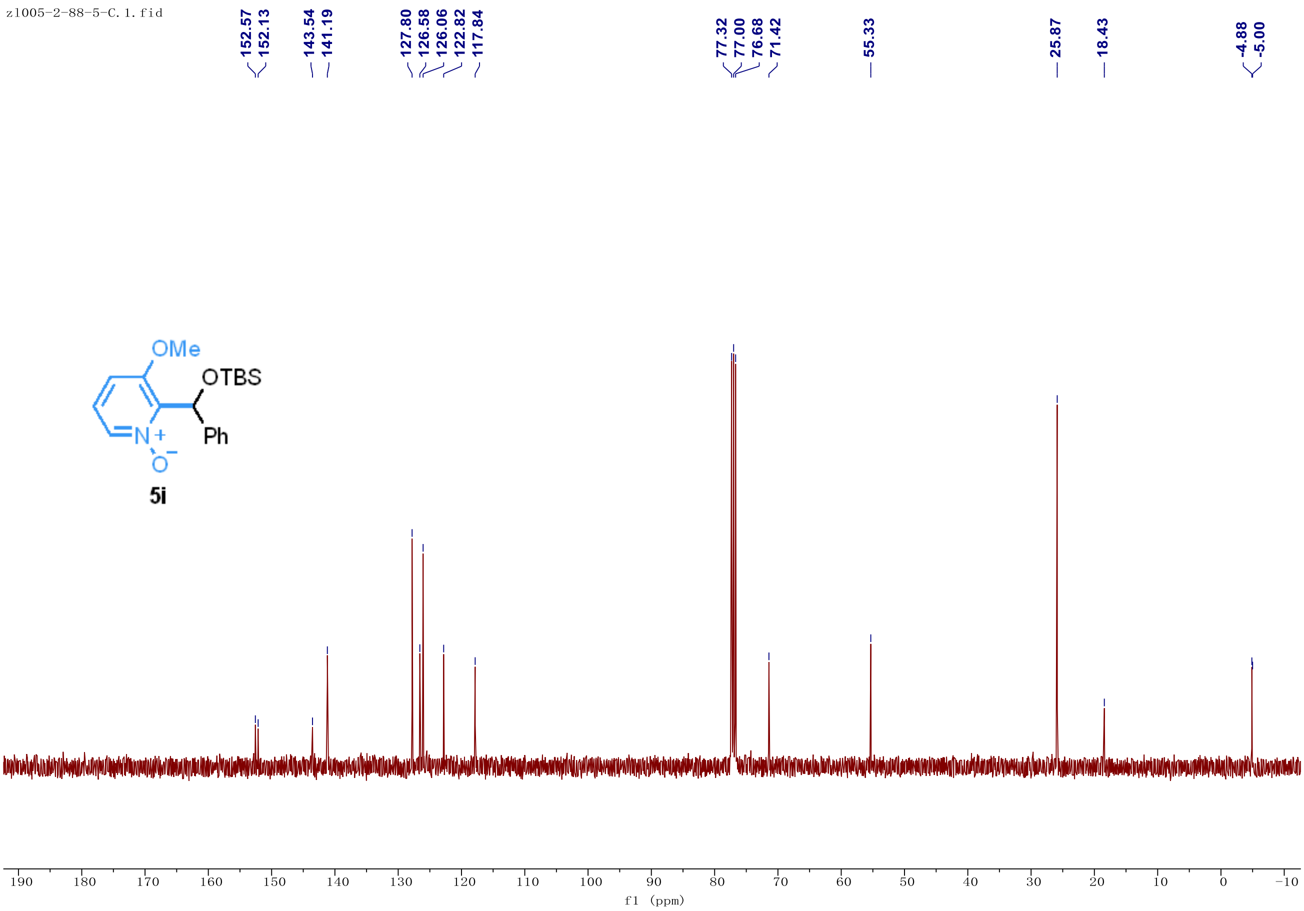
z1005-2-88-5-C.1.fid
152.57
152.13
143.54
141.19
127.80
126.58
126.06
122.82
117.84
77.32
77.00
76.68
71.42
55.33
25.87
18.43
-4.88
-5.00
OMe
OTBS
N+
O−
Ph
5i
f1 (ppm)

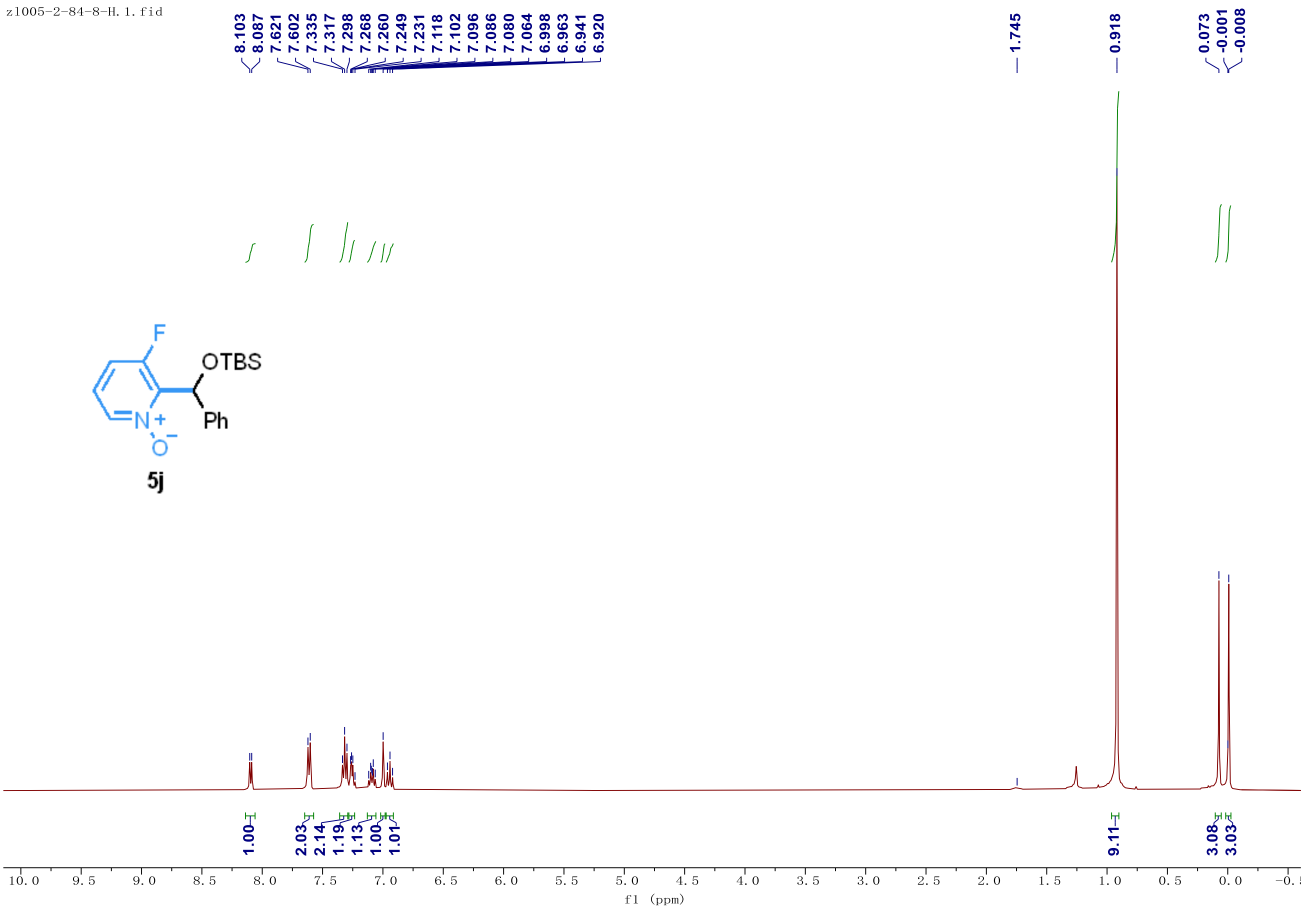
z1005-2-84-8-H.1.fid
8.103
8.087
7.621
7.602
7.335
7.317
7.298
7.268
7.260
7.249
7.231
7.118
7.102
7.096
7.086
7.080
7.064
6.998
6.963
6.941
6.920
1.745
0.918
0.073
-0.001
-0.008
F
OTBS
N+
O−
Ph
5j
1.00
2.03
2.14
1.19
1.13
1.00
1.01
9.11
3.08
3.03
f1 (ppm)

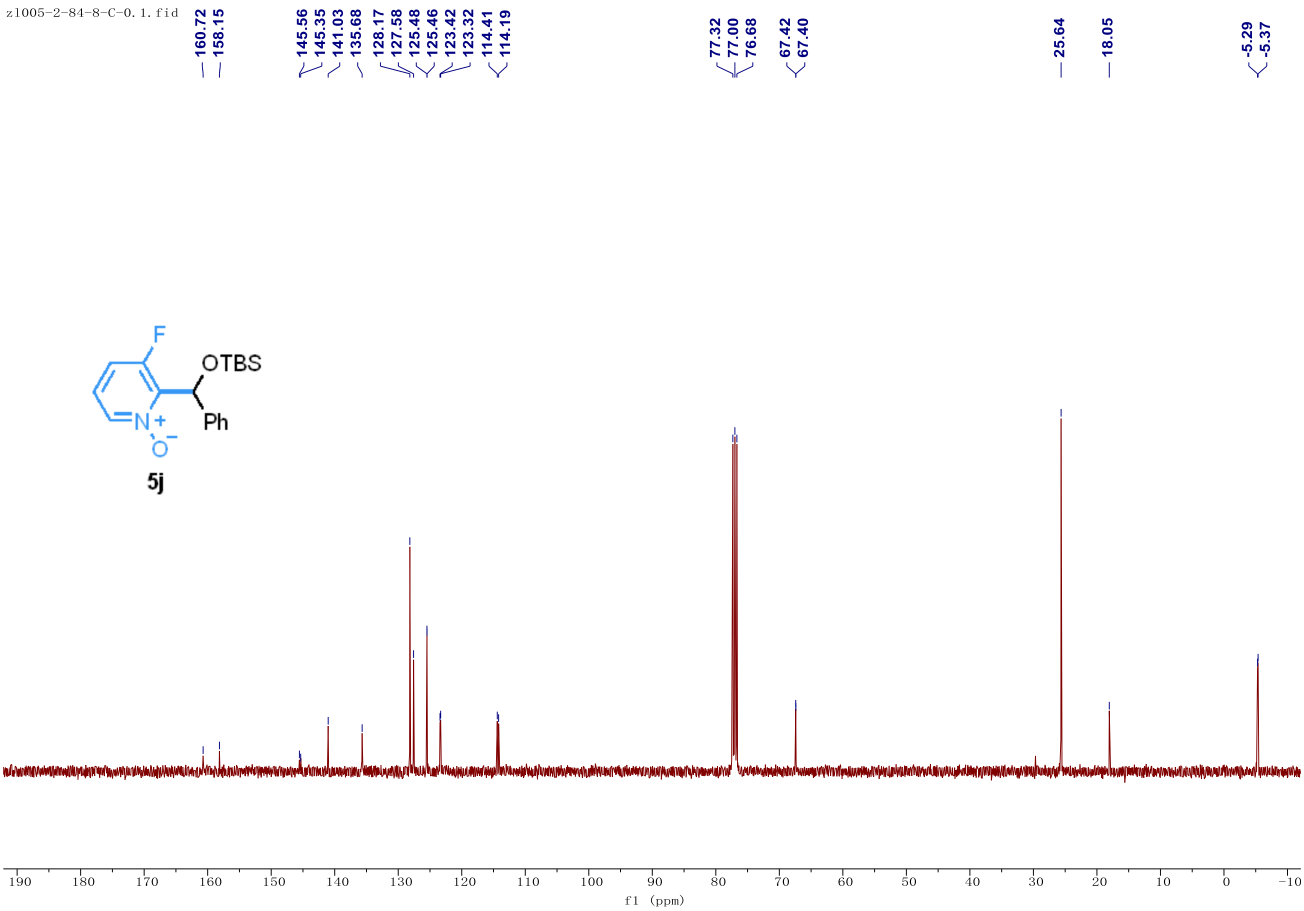

z1005-2-84-8-C-0.1.fid
160.72
158.15
145.56
145.35
141.03
135.68
128.17
127.58
125.48
125.46
123.42
123.32
114.41
114.19
77.32
77.00
76.68
67.42
67.40
25.64
18.05
-5.29
-5.37
F
OTBS
N+
O-
Ph
5j
190 180 170 160 150 140 130 120 110 100 90 80 70 60 50 40 30 20 10 0 −10
f1 (ppm)

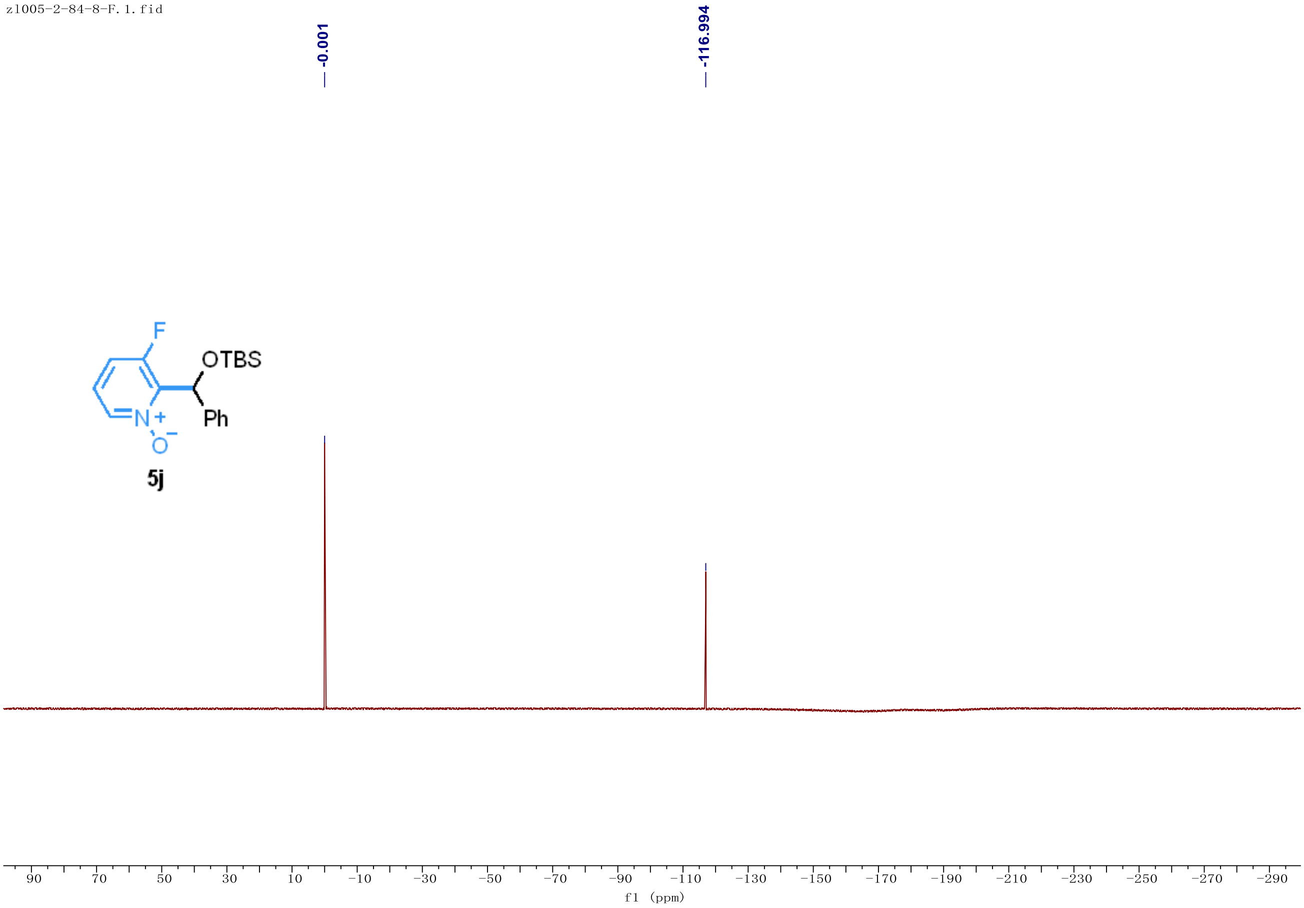

z1005-2-84-8-F.1.fid
-0.001
-116.994
F
OTBS
N+
O-
Ph
5j
90 70 50 30 10 −10 −30 −50 −70 −90 −110 −130 −150 −170 −190 −210 −230 −250 −270 −290
f1 (ppm)

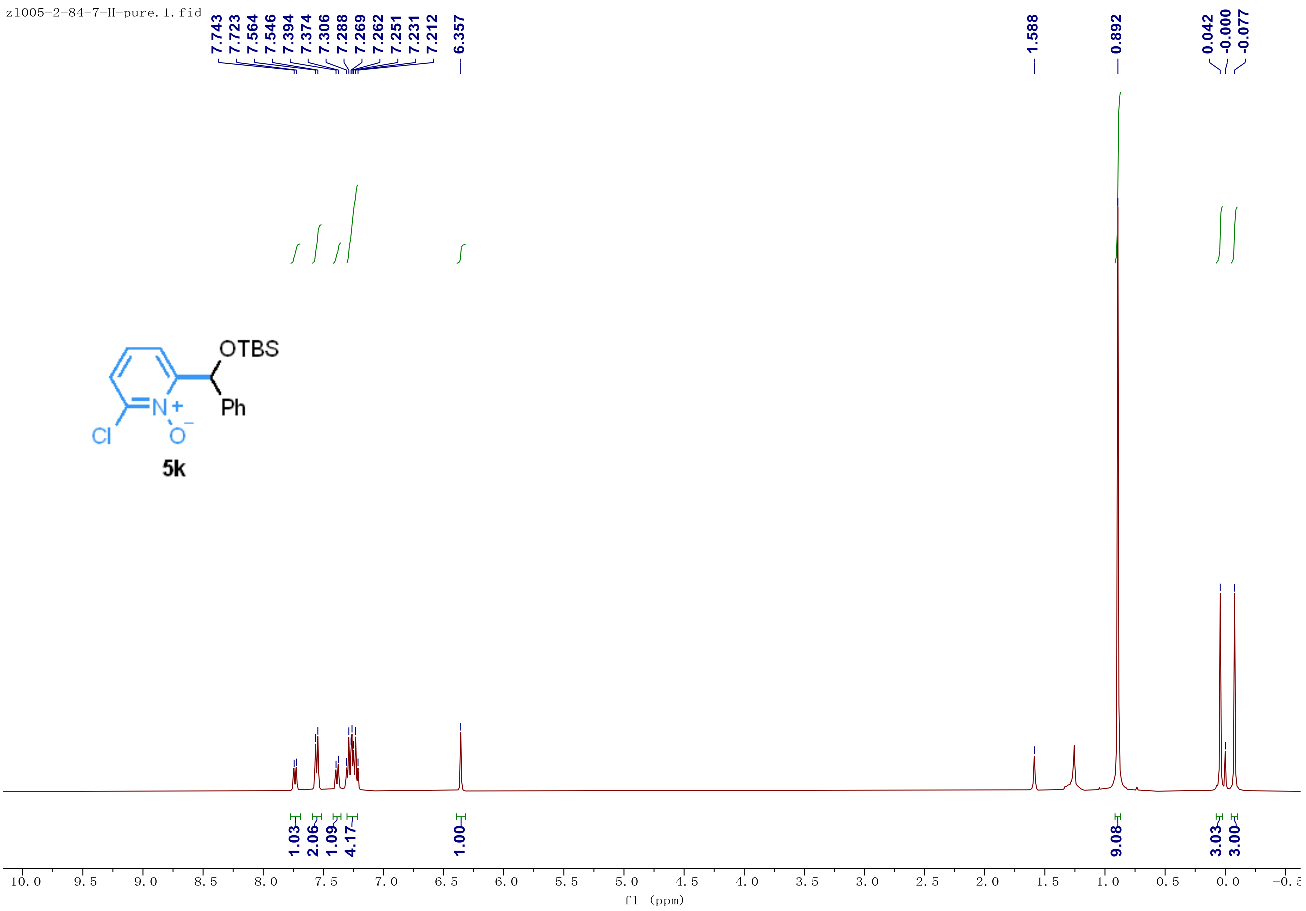
z1005-2-84-7-H-pure.1.fid
7.743
7.723
7.564
7.546
7.394
7.374
7.306
7.288
7.269
7.262
7.251
7.231
7.212
6.357
1.588
0.892
0.042
-0.000
-0.077
OTBS
Ph
N+
O−
Cl
5k
1.03
2.06
1.09
4.17
1.00
9.08
3.03
3.00
f1 (ppm)

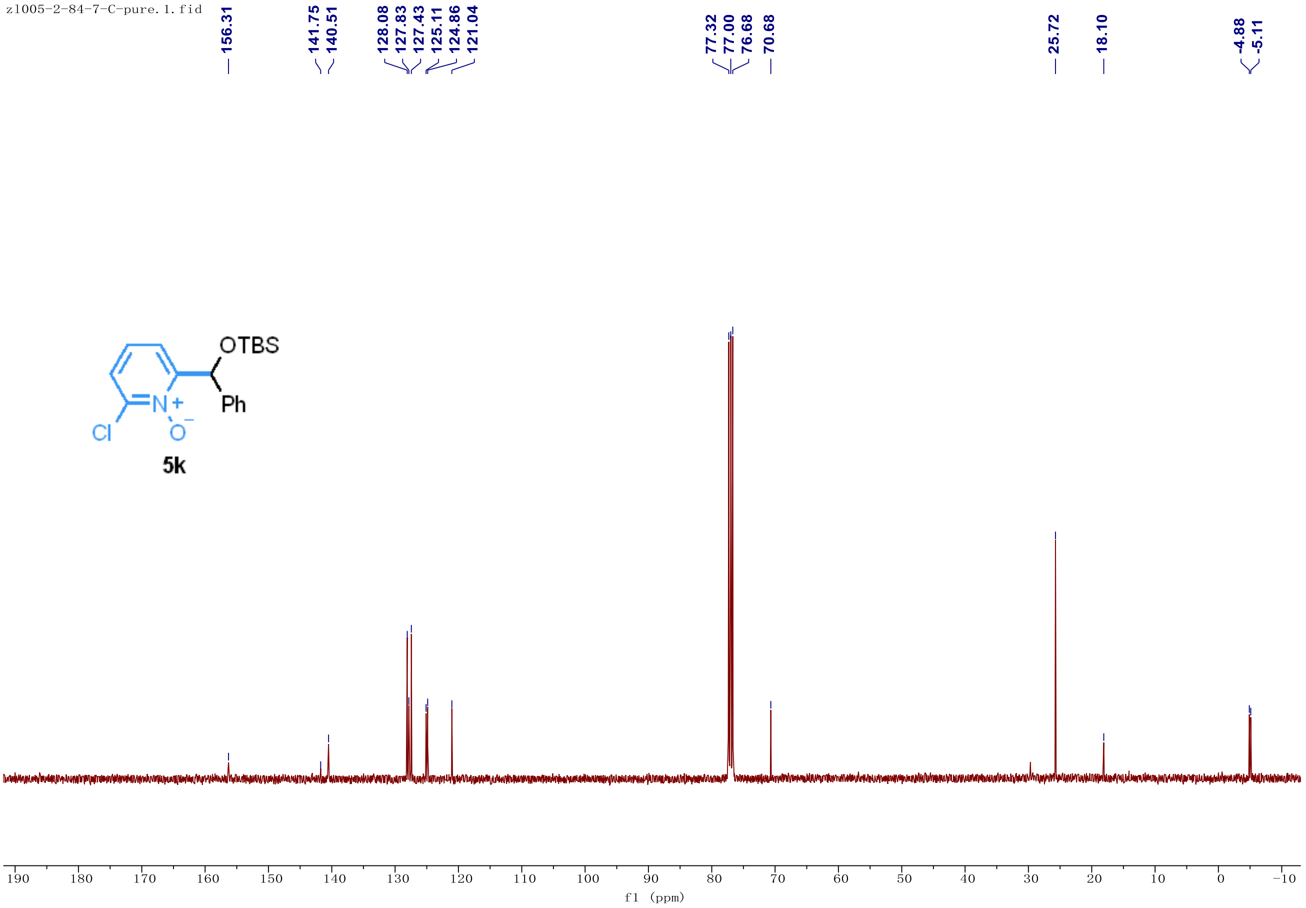
z1005-2-84-7-C-pure.1.fid
156.31
141.75
140.51
128.08
127.83
127.43
125.11
124.86
121.04
77.32
77.00
76.68
70.68
25.72
18.10
-4.88
-5.11
OTBS
Ph
N+
O−
Cl
5k
f1 (ppm)

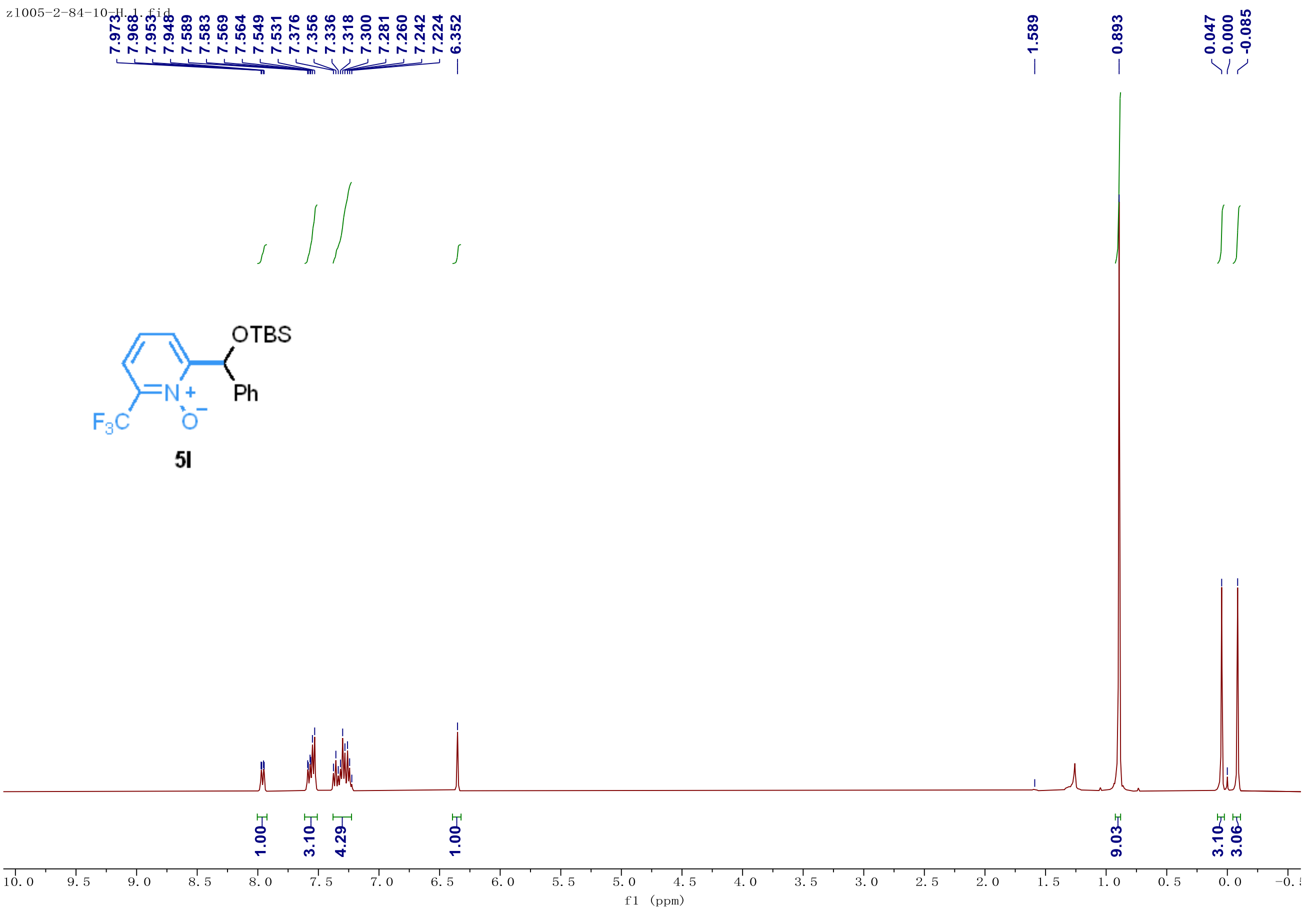
z1005-2-84-10-H.1.fid
7.973
7.968
7.953
7.948
7.589
7.583
7.569
7.564
7.549
7.531
7.376
7.356
7.336
7.318
7.300
7.281
7.260
7.242
7.224
6.352
1.589
0.893
0.047
0.000
-0.085
OTBS
Ph
F3C
N+
O−
5l
1.00
3.10
4.29
1.00
9.03
3.10
3.06
f1 (ppm)

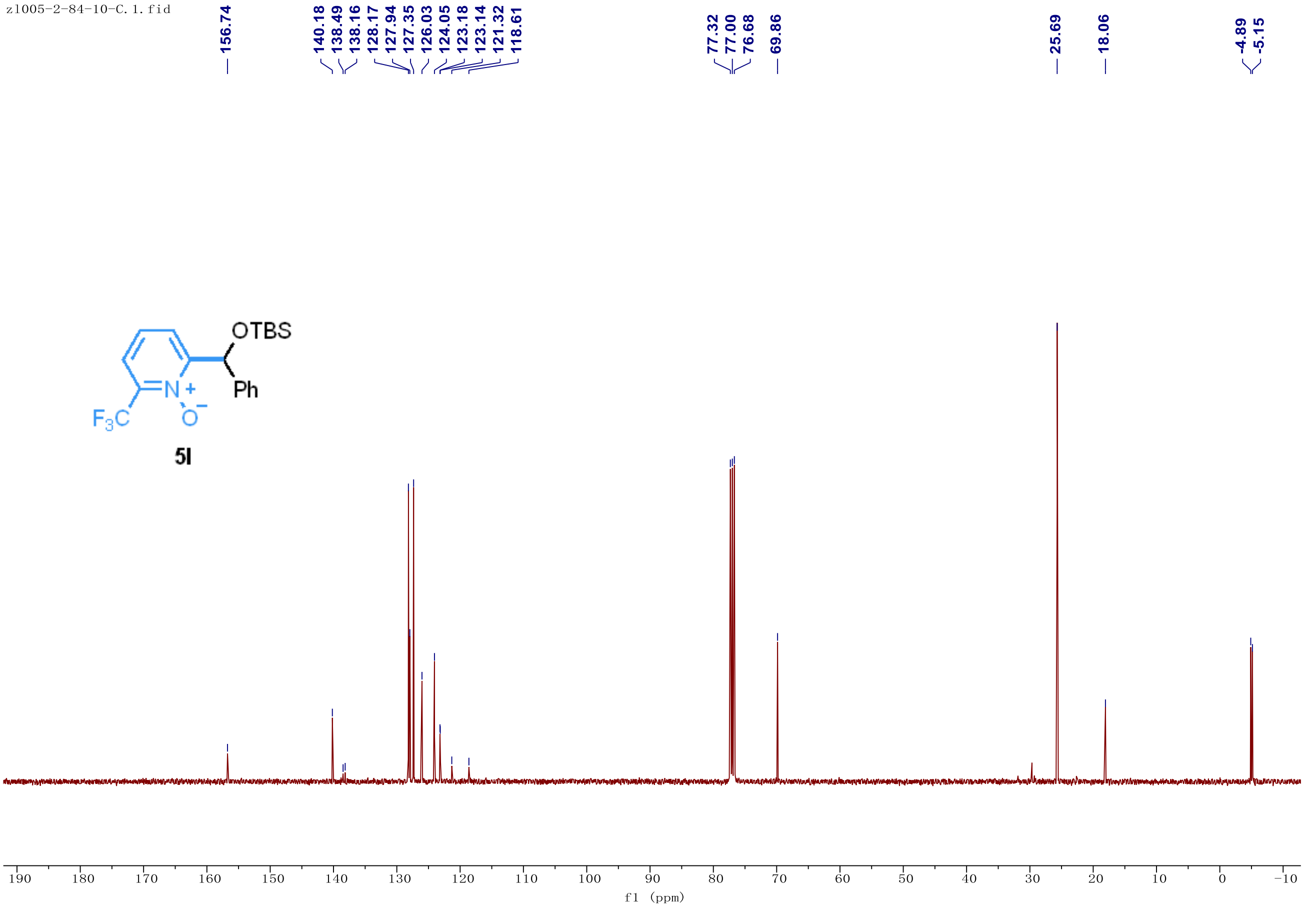
z1005-2-84-10-C.1.fid
156.74
140.18
138.49
138.16
128.17
127.94
127.35
126.03
124.05
123.18
123.14
121.32
118.61
77.32
77.00
76.68
69.86
25.69
18.06
-4.89
-5.15
OTBS
Ph
F3C
N+
O−
5l
f1 (ppm)

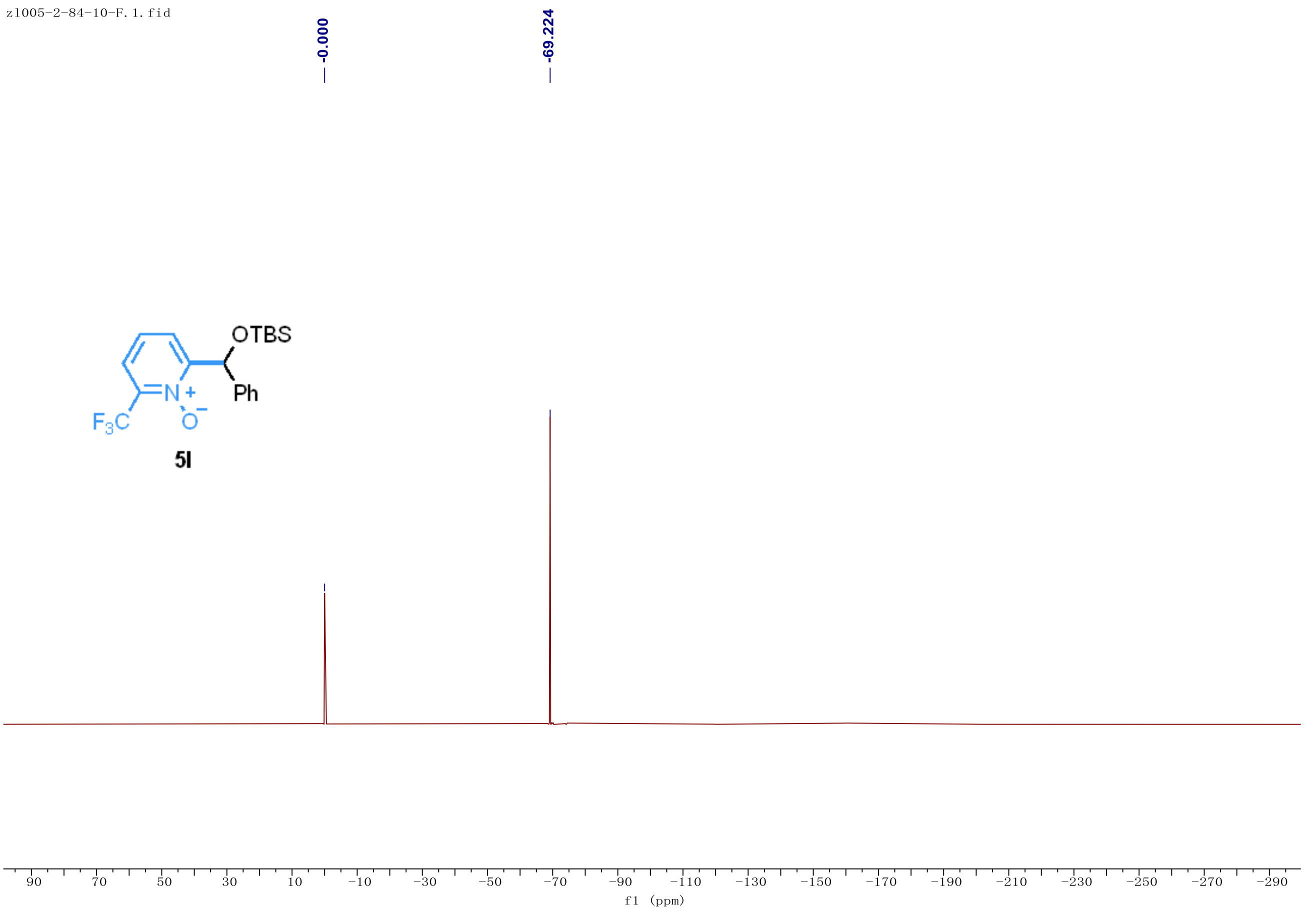
z1005-2-84-10-F.1.fid
-0.000
-69.224
OTBS
Ph
F3C
5l
f1 (ppm)

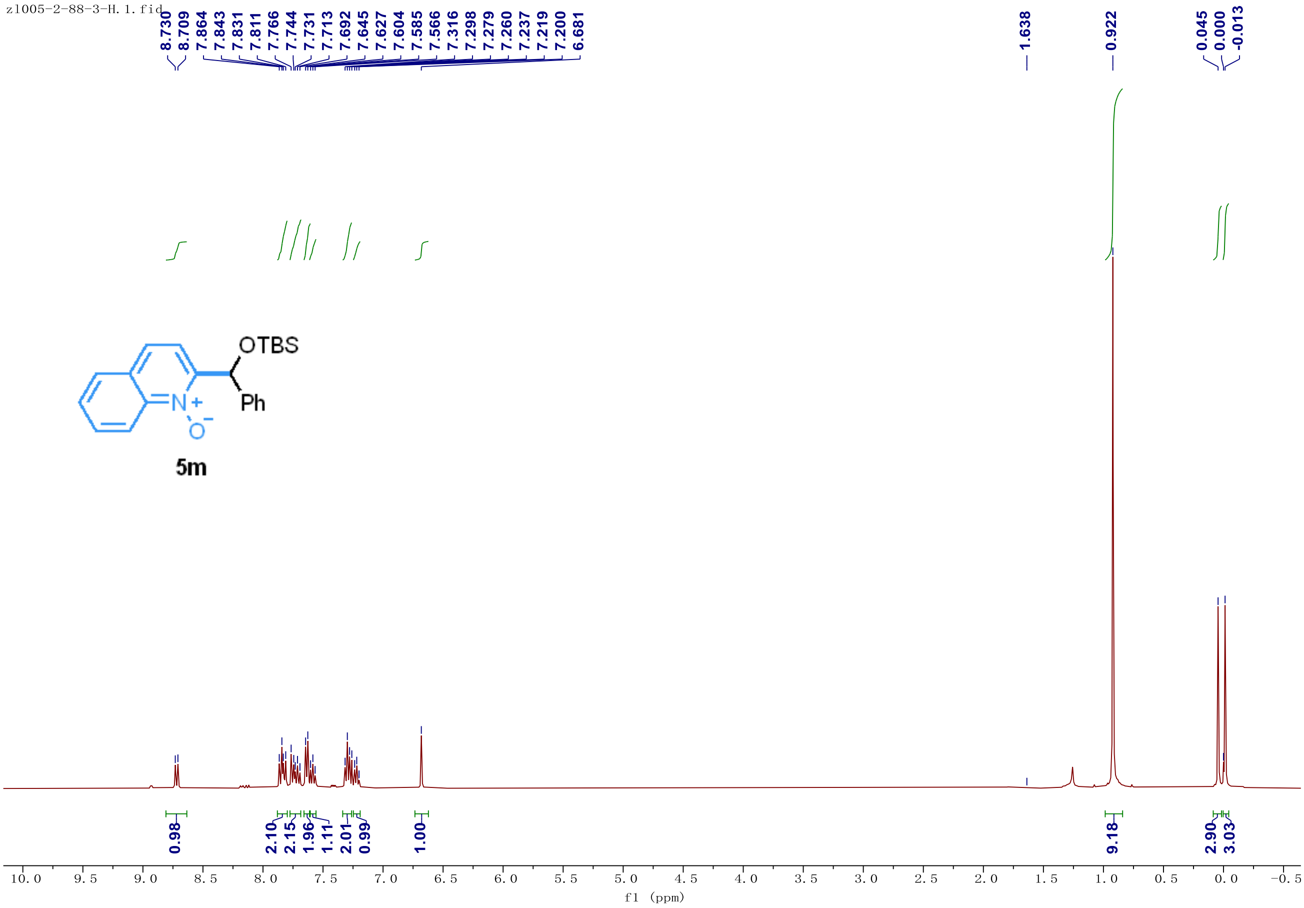
z1005-2-88-3-H.1.fid
8.730
8.709
7.864
7.843
7.831
7.811
7.766
7.744
7.731
7.713
7.692
7.645
7.627
7.604
7.585
7.566
7.316
7.298
7.279
7.260
7.237
7.219
7.200
6.681
1.638
0.922
0.045
0.000
-0.013
OTBS
Ph
5m
0.98
2.10
2.15
1.96
1.11
2.01
0.99
1.00
9.18
2.90
3.03
f1 (ppm)

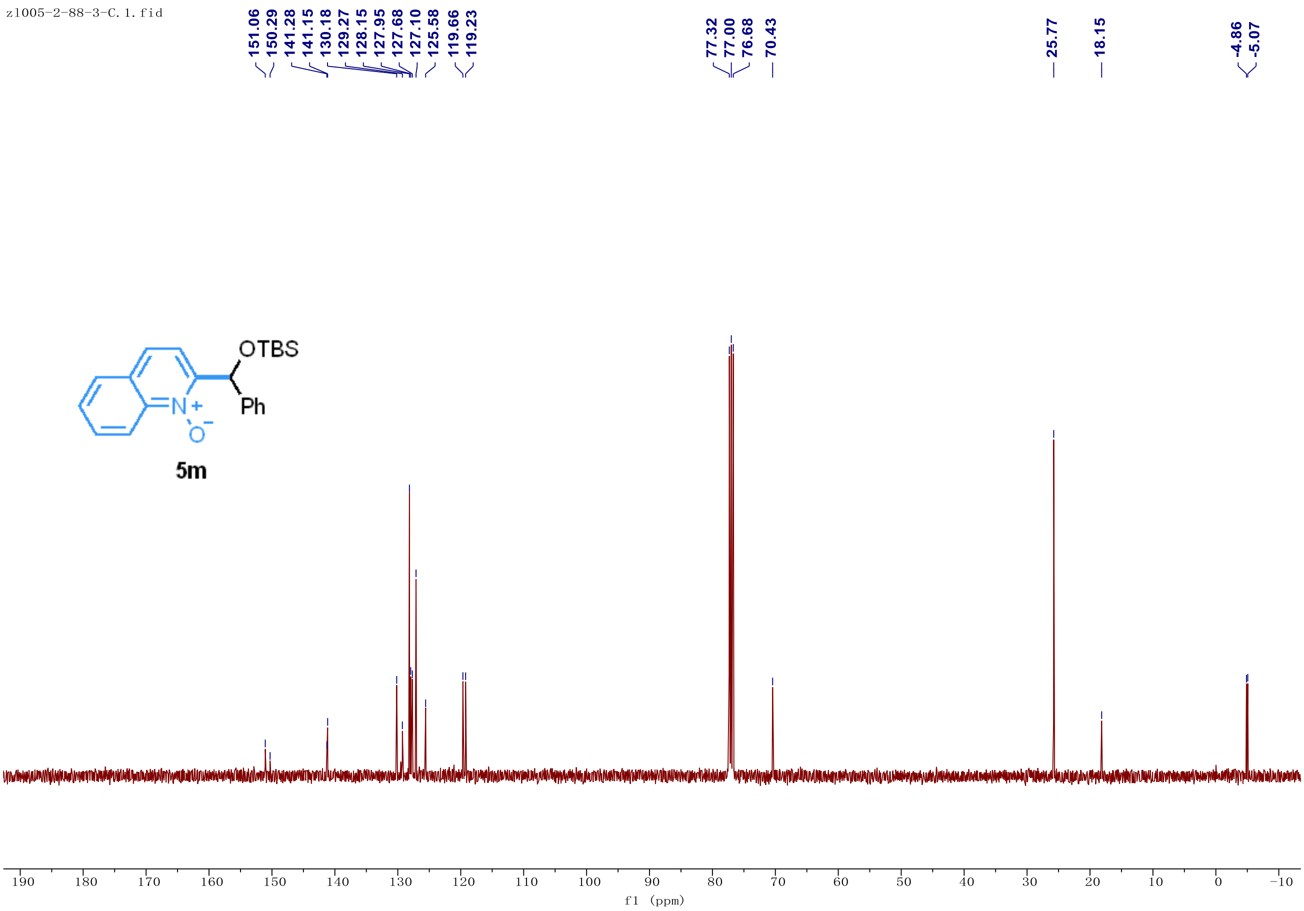

z1005-2-88-3-C.1.fid
151.06
150.29
141.28
141.15
130.18
129.27
128.15
127.95
127.68
127.10
125.58
119.66
119.23
77.32
77.00
76.68
70.43
25.77
18.15
-4.86
-5.07
OTBS
N+
O-
Ph
5m
190 180 170 160 150 140 130 120 110 100 90 80 70 60 50 40 30 20 10 0 -10
f1 (ppm)

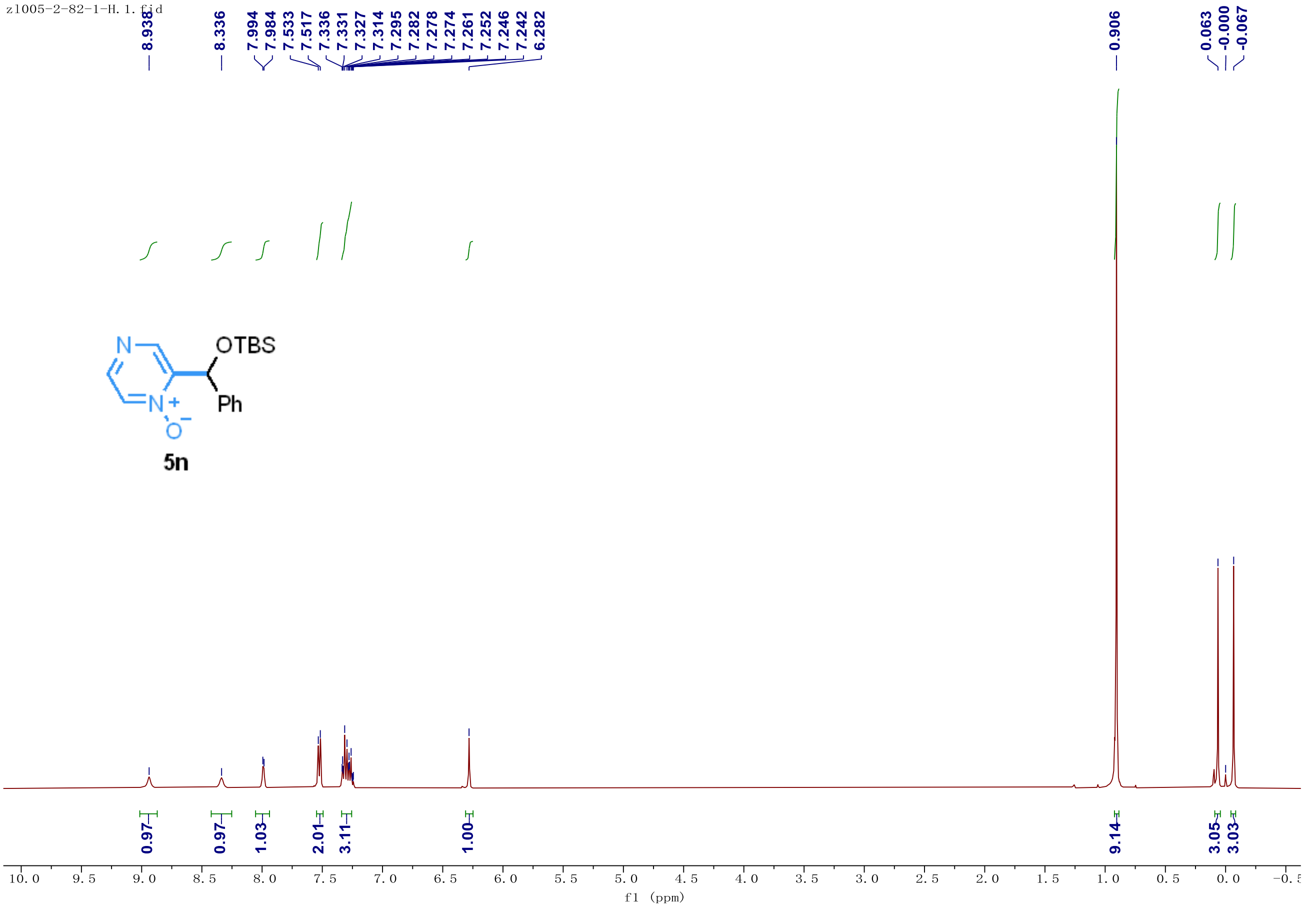

z1005-2-82-1-H.1.fid
8.938
8.336
7.994
7.984
7.533
7.517
7.336
7.331
7.327
7.314
7.295
7.282
7.278
7.274
7.261
7.252
7.246
7.242
6.282
0.906
0.063
-0.000
-0.067
N
OTBS
N+
O-
Ph
5n
0.97
0.97
1.03
2.01
3.11
1.00
9.14
3.05
3.03
10.0 9.5 9.0 8.5 8.0 7.5 7.0 6.5 6.0 5.5 5.0 4.5 4.0 3.5 3.0 2.5 2.0 1.5 1.0 0.5 0.0
f1 (ppm)

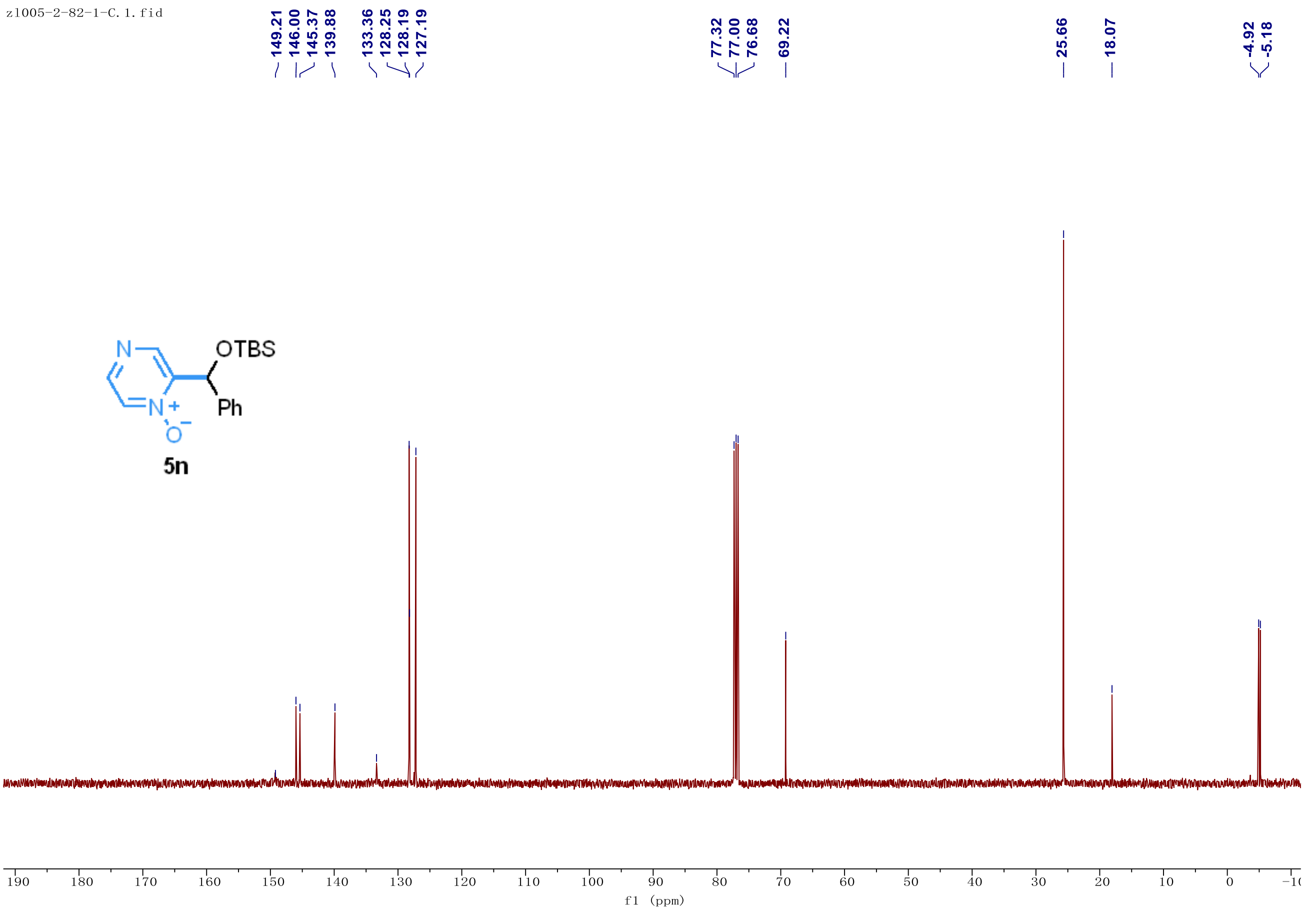

z1005-2-82-1-C.1.fid
149.21
146.00
145.37
139.88
133.36
128.25
128.19
127.19
77.32
77.00
76.68
69.22
25.66
18.07
-4.92
-5.18
OTBS
Ph
5n
190 180 170 160 150 140 130 120 110 100 90 80 70 60 50 40 30 20 10 0
f1 (ppm)

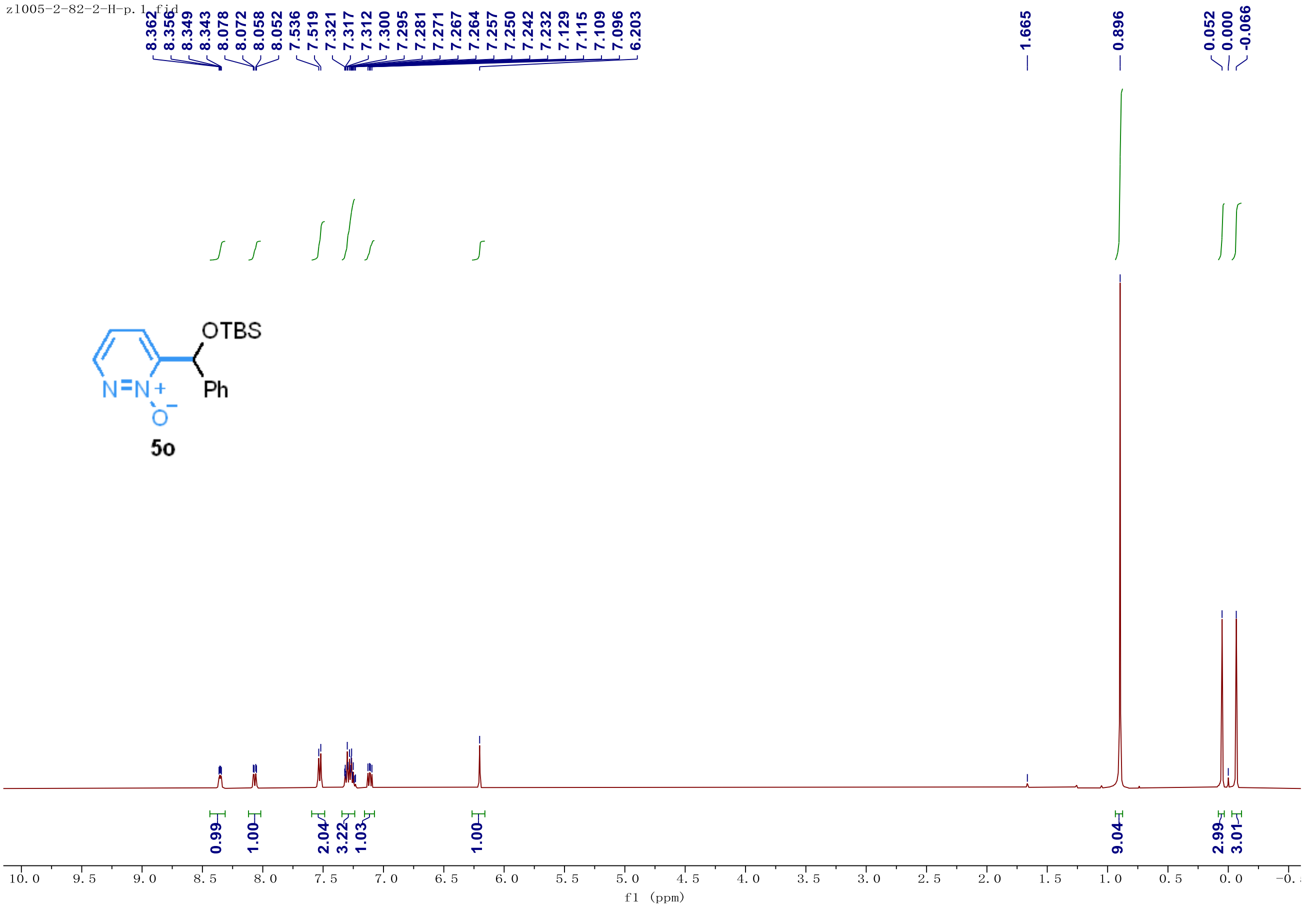

z1005-2-82-2-H-p.1.fid
8.362
8.356
8.349
8.343
8.078
8.072
8.058
8.052
7.536
7.519
7.321
7.317
7.312
7.300
7.295
7.281
7.271
7.267
7.264
7.257
7.250
7.242
7.232
7.129
7.115
7.109
7.096
6.203
1.665
0.896
0.052
0.000
-0.066
OTBS
Ph
5o
0.99
1.00
2.04
3.22
1.03
1.00
9.04
2.99
3.01
10.0 9.5 9.0 8.5 8.0 7.5 7.0 6.5 6.0 5.5 5.0 4.5 4.0 3.5 3.0 2.5 2.0 1.5 1.0 0.5 0.0
f1 (ppm)

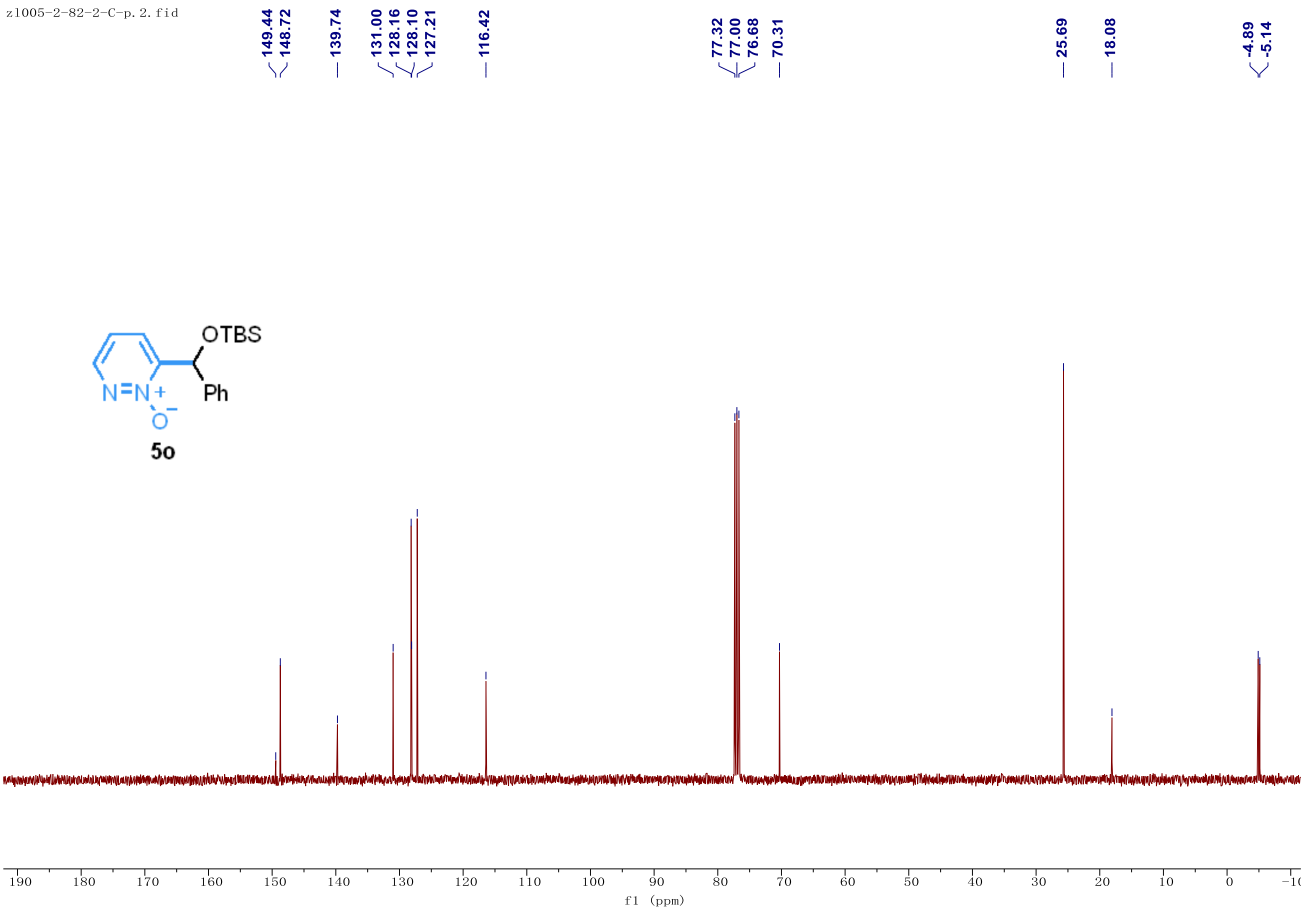
z1005-2-82-2-C-p.2.fid
149.44
148.72
139.74
131.00
128.16
128.10
127.21
116.42
77.32
77.00
76.68
70.31
25.69
18.08
-4.89
-5.14
OTBS
N
N+
O−
Ph
5o
f1 (ppm)

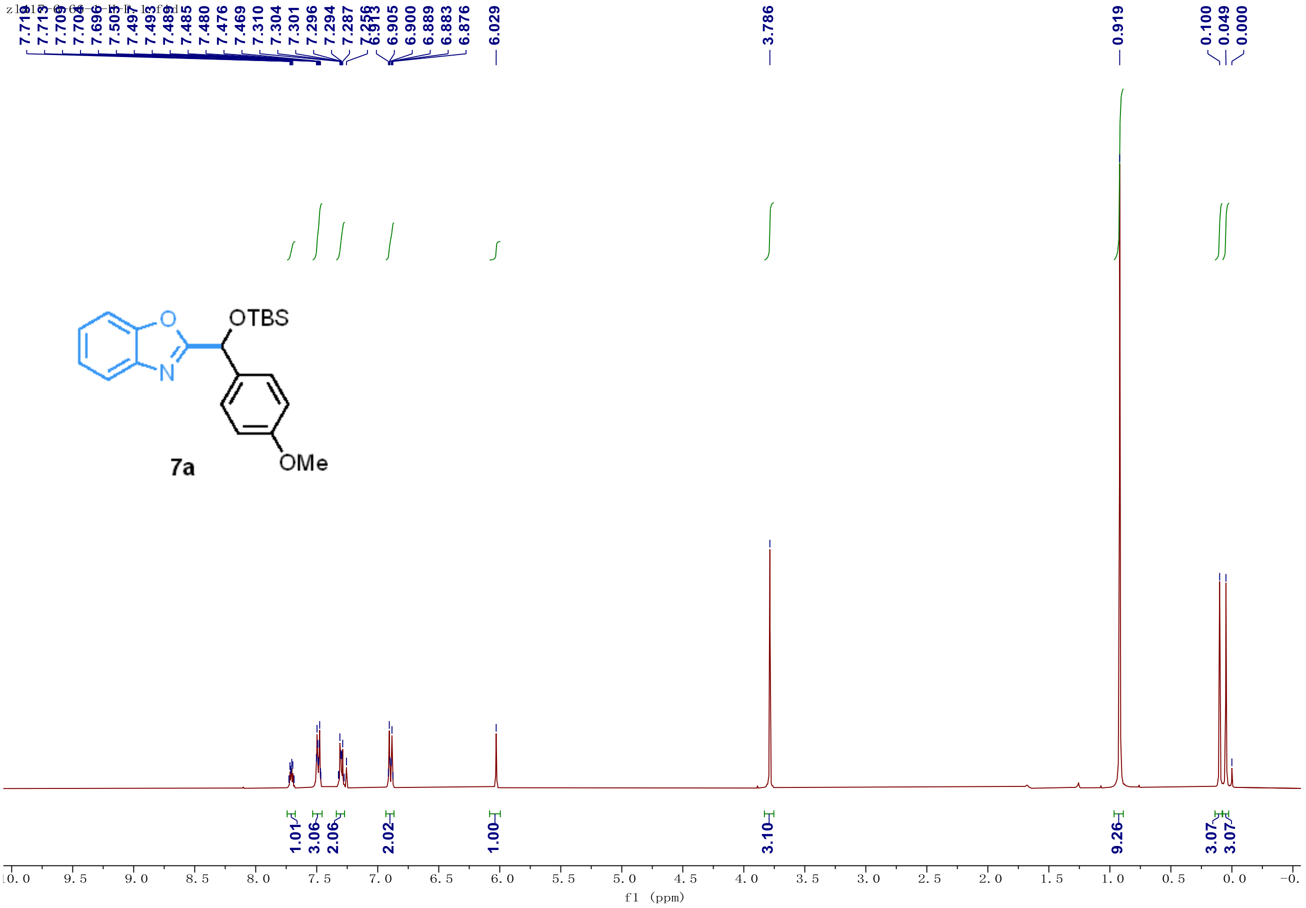
6.029
3.786
0.919
0.100
0.049
0.000
OTBS
O
N
OMe
7a
1.01
3.06
2.06
2.02
1.00
3.10
9.26
3.07
3.07
f1 (ppm)

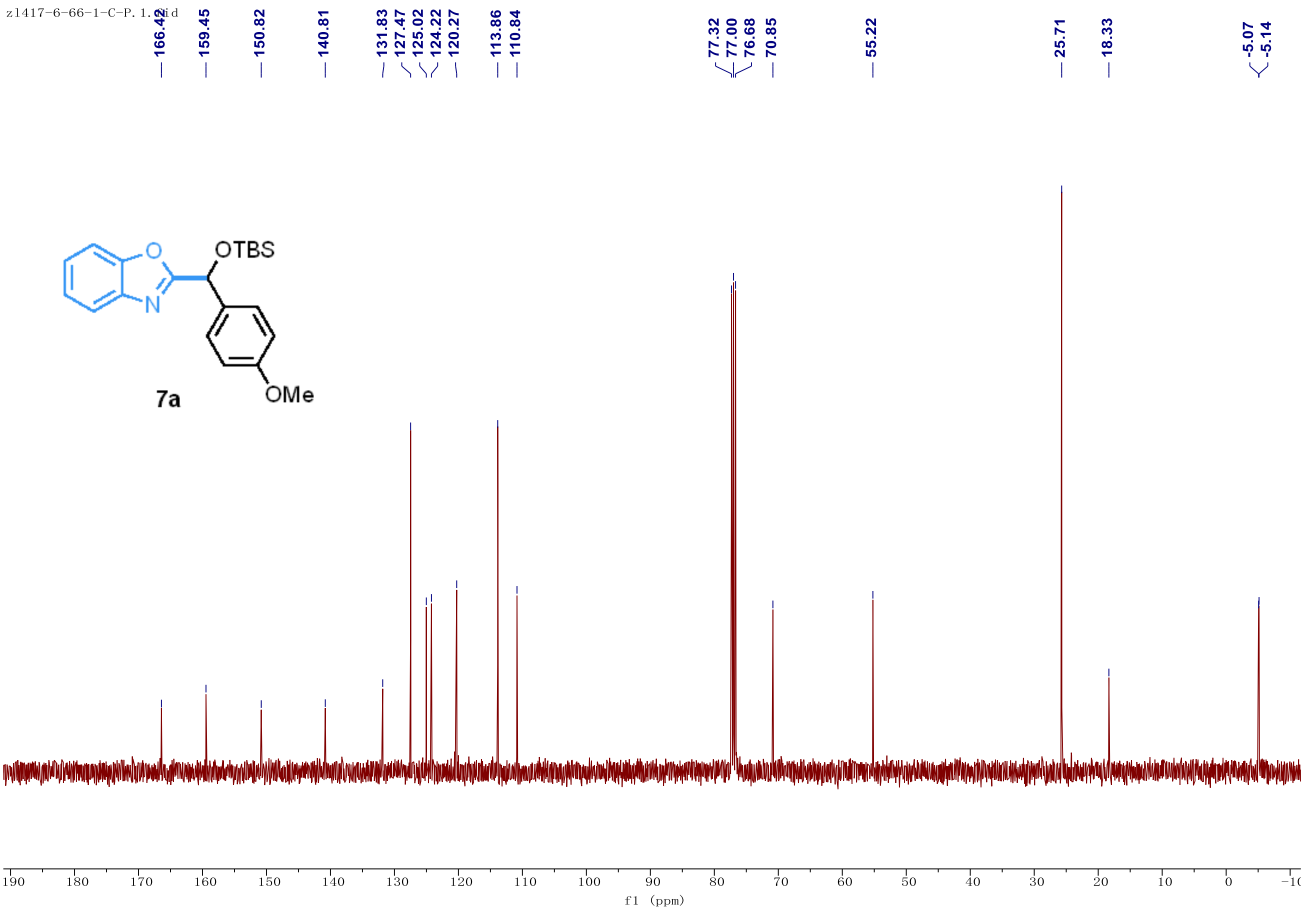
z1417-6-66-1-C-P.1.fid
166.42
159.45
150.82
140.81
131.83
127.47
125.02
124.22
120.27
113.86
110.84
77.32
77.00
76.68
70.85
55.22
25.71
18.33
-5.07
-5.14
OTBS
O
N
OMe
7a
f1 (ppm)

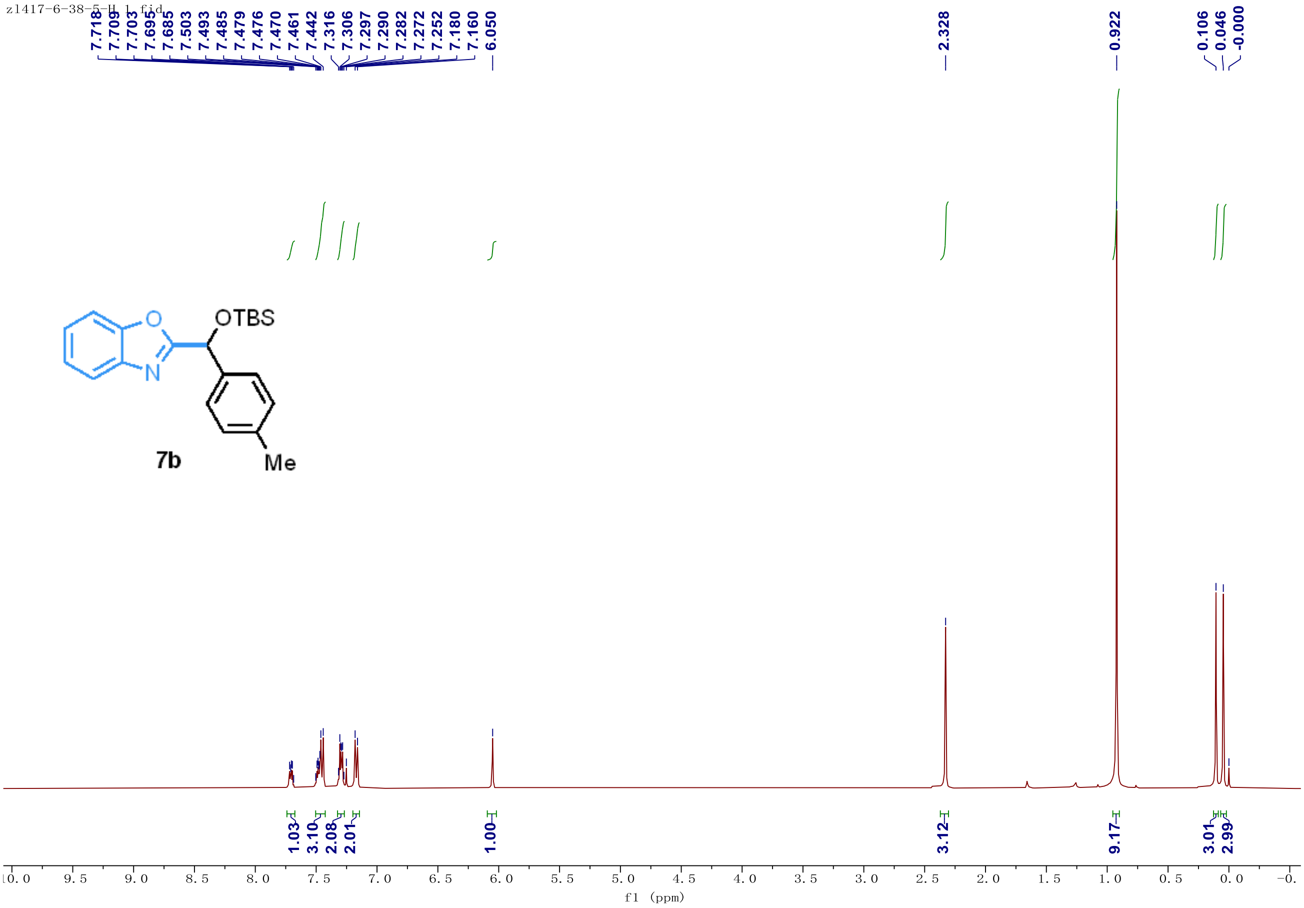
z1417-6-38-5-H.1.fid
7.718
7.709
7.703
7.695
7.685
7.503
7.493
7.485
7.479
7.476
7.470
7.461
7.442
7.316
7.306
7.297
7.290
7.282
7.272
7.252
7.180
7.160
6.050
2.328
0.922
0.106
0.046
-0.000
OTBS
O
N
Me
7b
1.03
3.10
2.08
2.01
1.00
3.12
9.17
3.01
2.99
f1 (ppm)

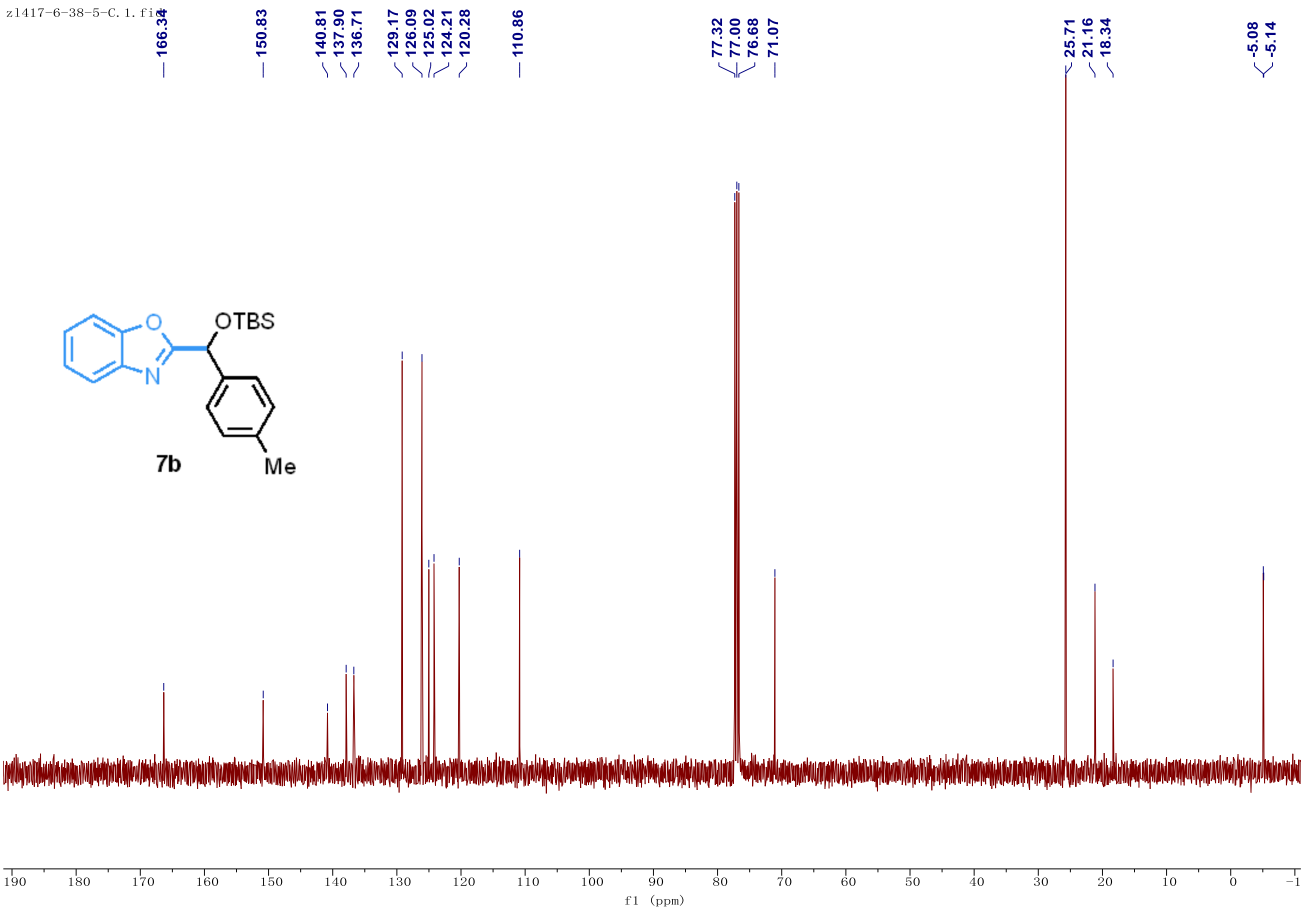
z1417-6-38-5-C.1.fid
166.34
150.83
140.81
137.90
136.71
129.17
126.09
125.02
124.21
120.28
110.86
77.32
77.00
76.68
71.07
25.71
21.16
18.34
-5.08
-5.14
OTBS
O
N
7b
Me
190 180 170 160 150 140 130 120 110 100 90 80 70 60 50 40 30 20 10 0 -1
f1 (ppm)

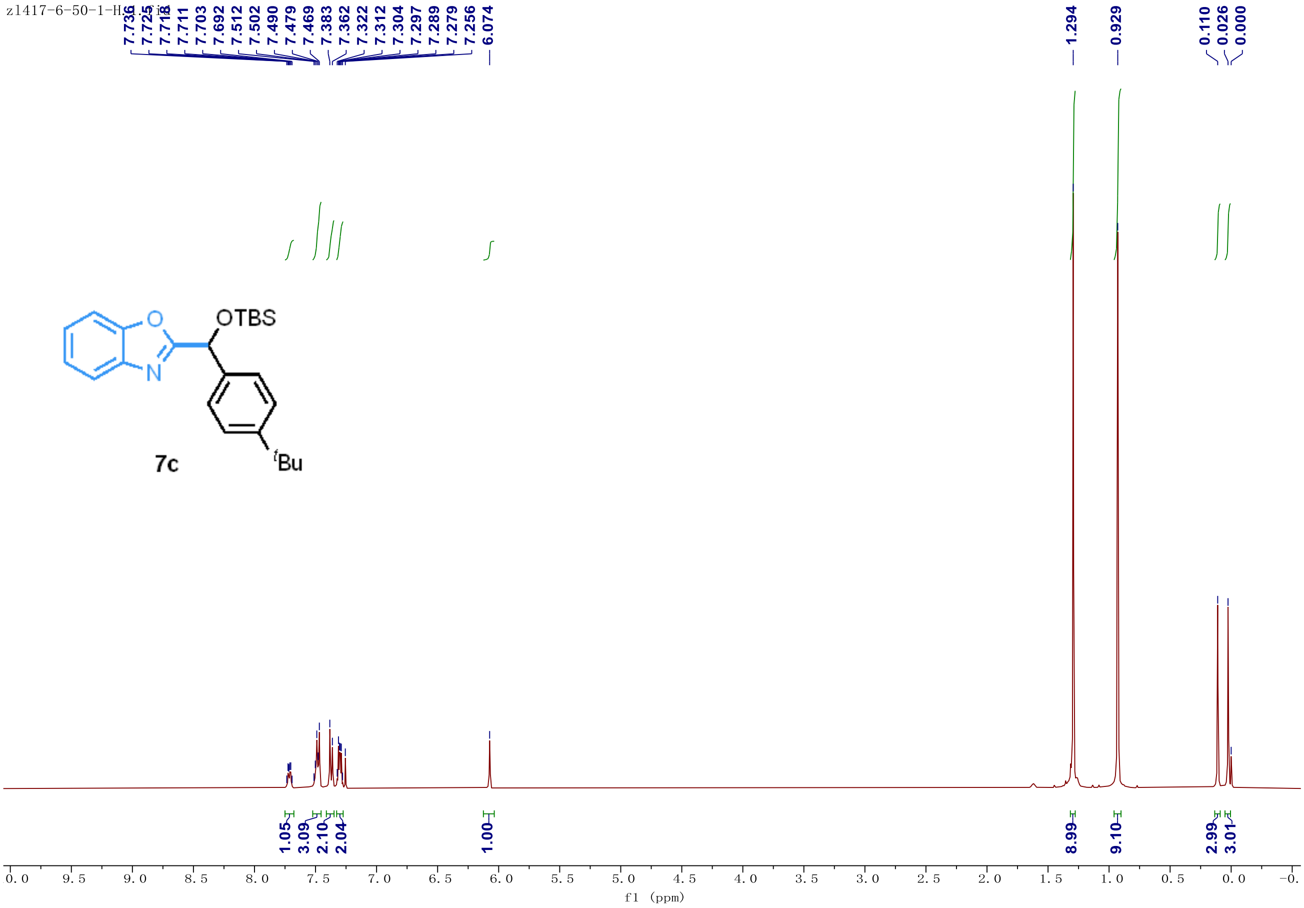
z1417-6-50-1-H
7.736
7.725
7.718
7.711
7.703
7.692
7.512
7.502
7.490
7.479
7.469
7.383
7.362
7.322
7.312
7.304
7.297
7.289
7.279
7.256
6.074
1.294
0.929
0.110
0.026
0.000
OTBS
O
N
7c
tBu
1.05
3.09
2.10
2.04
1.00
8.99
9.10
2.99
3.01
10.0 9.5 9.0 8.5 8.0 7.5 7.0 6.5 6.0 5.5 5.0 4.5 4.0 3.5 3.0 2.5 2.0 1.5 1.0 0.5 0.0 -0.
f1 (ppm)

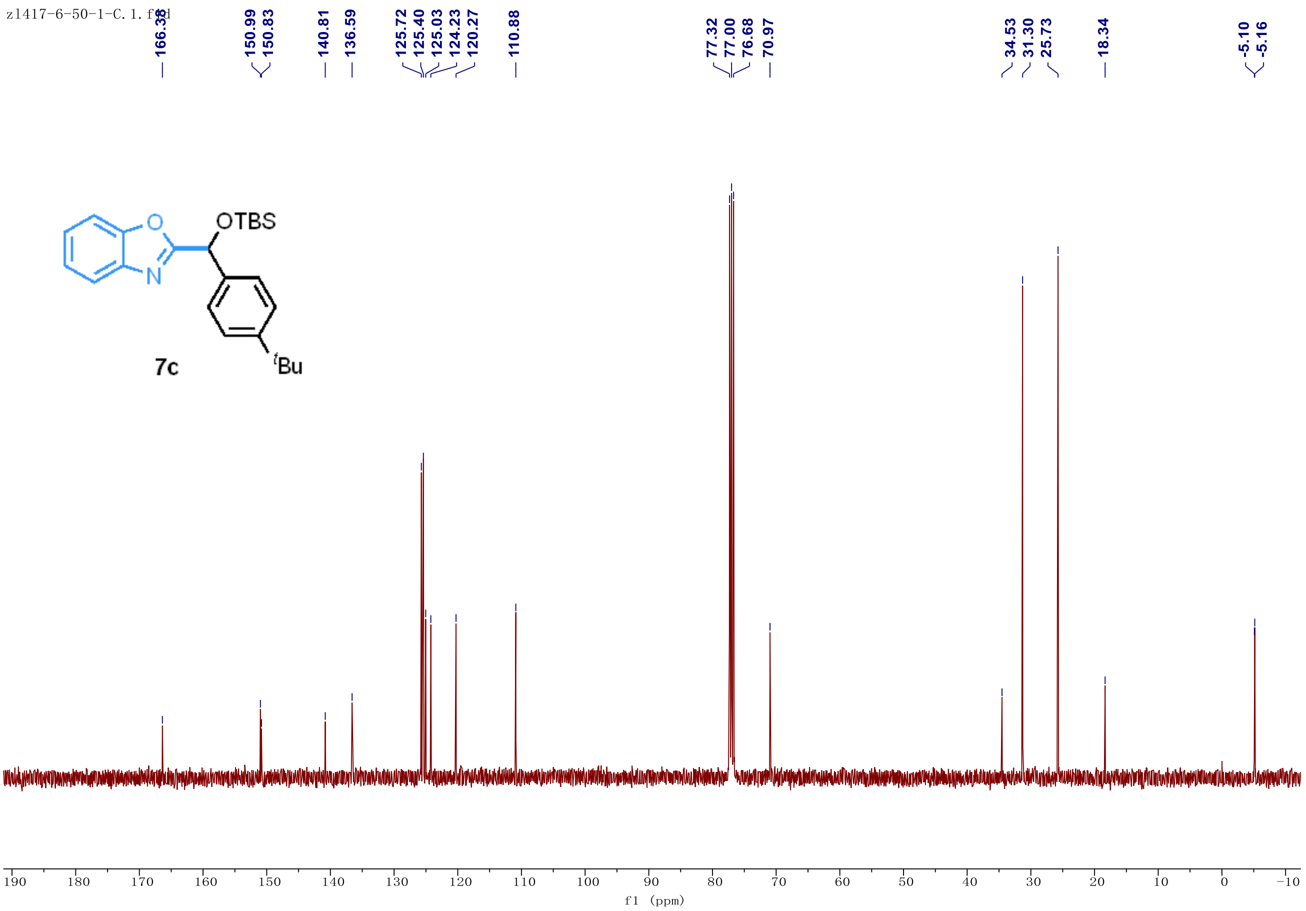
z1417-6-50-1-C.1.fid
166.38
150.99
150.83
140.81
136.59
125.72
125.40
125.03
124.23
120.27
110.88
77.32
77.00
76.68
70.97
34.53
31.30
25.73
18.34
-5.10
-5.16
OTBS
O
N
7c
tBu
190 180 170 160 150 140 130 120 110 100 90 80 70 60 50 40 30 20 10 0 -10
f1 (ppm)

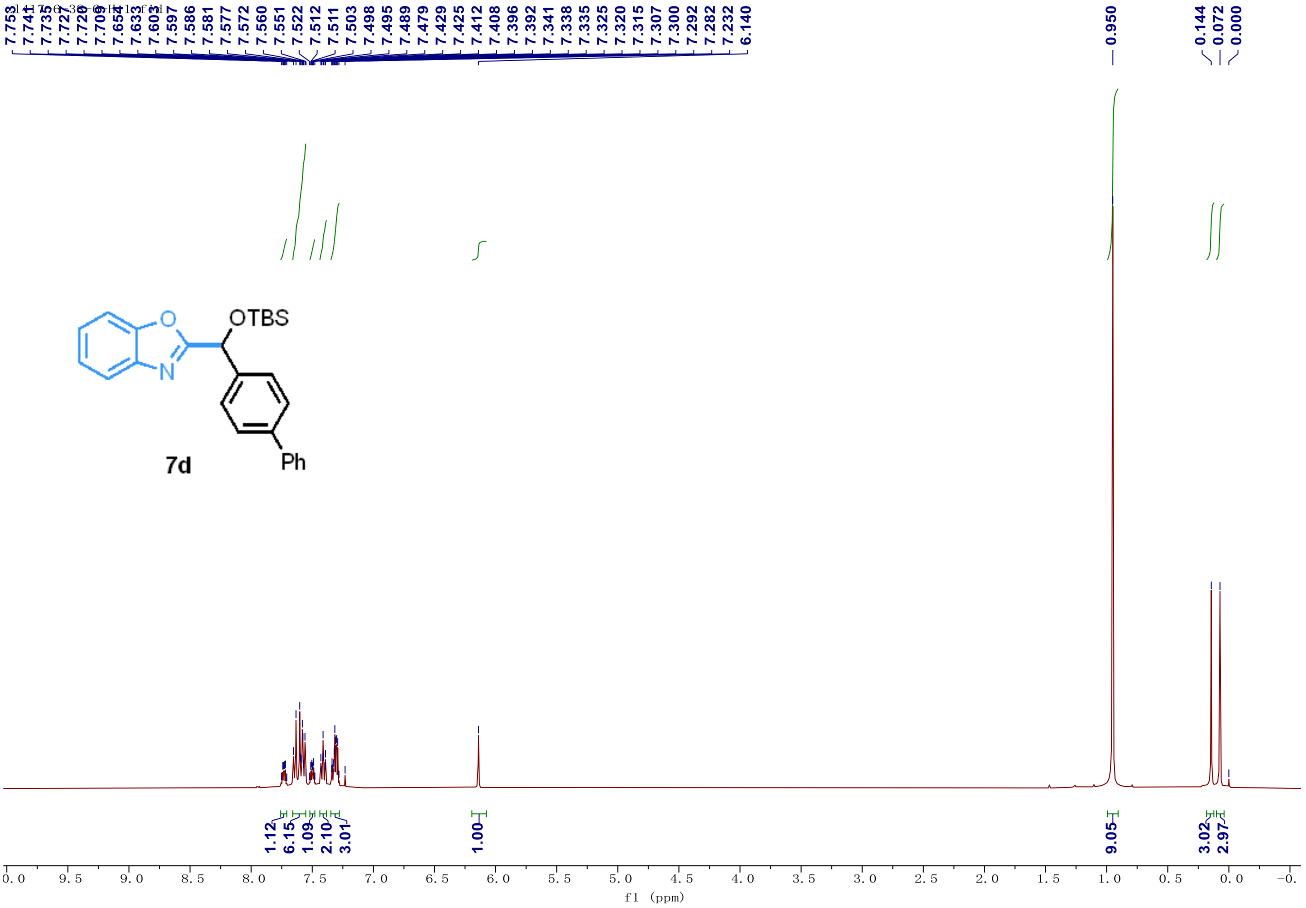
7.753
7.741
7.735
7.727
7.726
7.709
7.654
7.633
7.603
7.597
7.586
7.581
7.577
7.572
7.560
7.551
7.522
7.512
7.511
7.503
7.498
7.495
7.489
7.479
7.429
7.425
7.412
7.408
7.396
7.392
7.341
7.338
7.335
7.325
7.320
7.315
7.307
7.300
7.292
7.282
7.232
6.140
0.950
0.144
0.072
0.000
OTBS
O
N
7d
Ph
1.12
6.15
1.09
2.10
3.01
1.00
9.05
3.02
2.97
0.0 9.5 9.0 8.5 8.0 7.5 7.0 6.5 6.0 5.5 5.0 4.5 4.0 3.5 3.0 2.5 2.0 1.5 1.0 0.5 0.0 -0.
f1 (ppm)

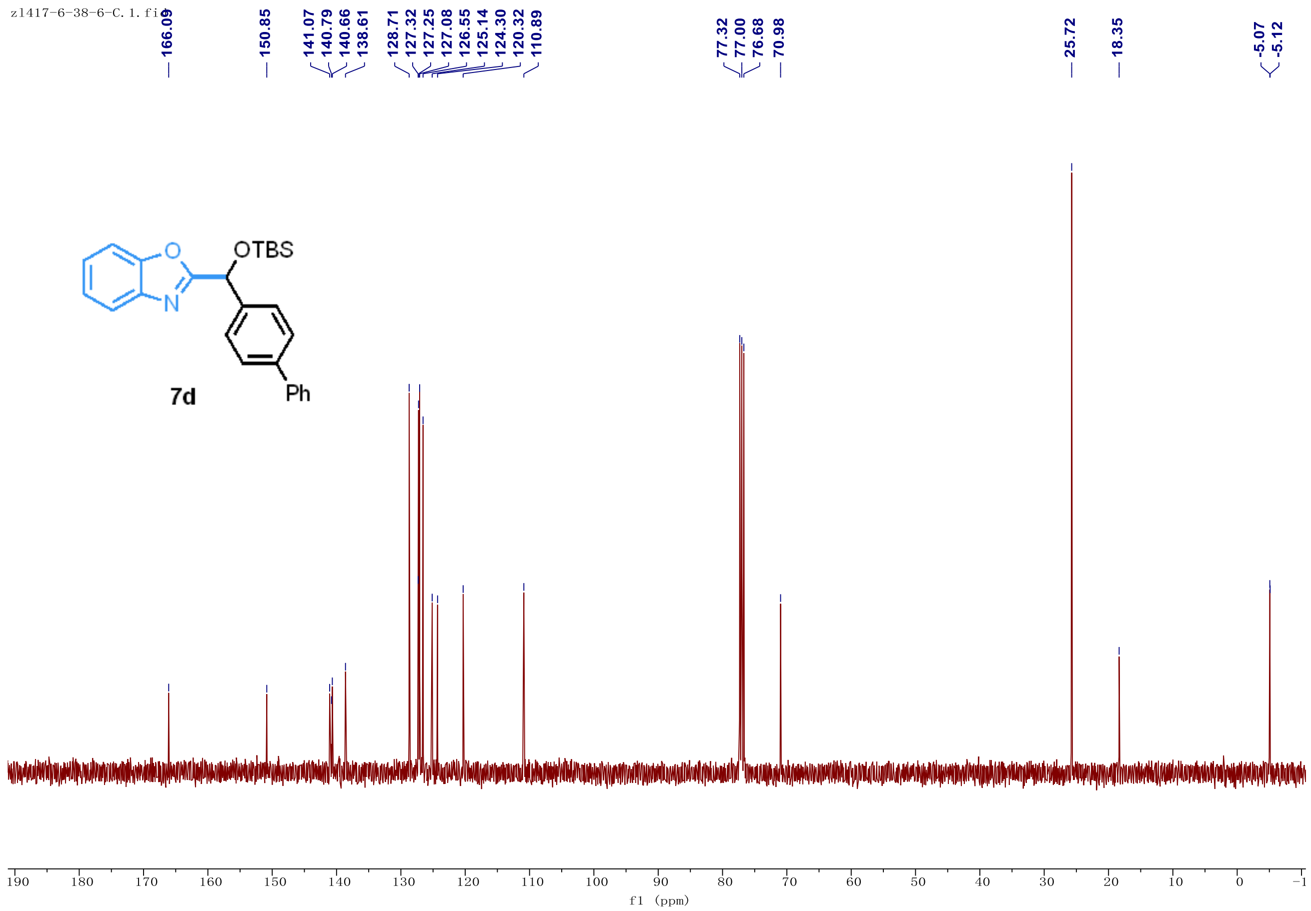
z1417-6-38-6-C.1.fid
166.09
150.85
141.07
140.79
140.66
138.61
128.71
127.32
127.25
127.08
126.55
125.14
124.30
120.32
110.89
77.32
77.00
76.68
70.98
25.72
18.35
-5.07
-5.12
OTBS
O
N
Ph
7d
f1 (ppm)

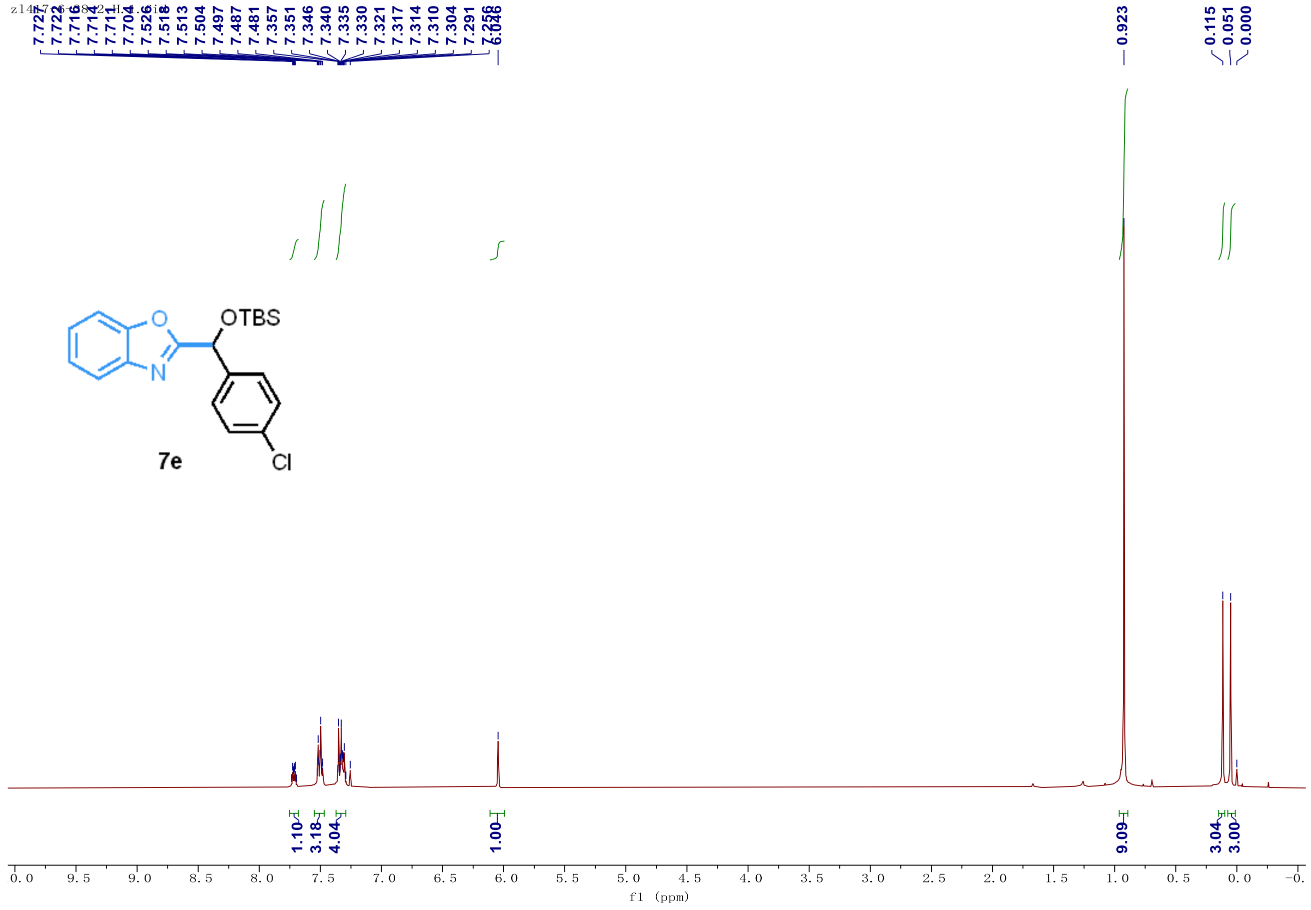
7.727
7.722
7.716
7.714
7.711
7.704
7.526
7.518
7.513
7.504
7.497
7.487
7.481
7.357
7.351
7.346
7.340
7.335
7.330
7.321
7.317
7.314
7.310
7.304
7.291
7.256
6.046
0.923
0.115
0.051
0.000
OTBS
O
N
Cl
7e
1.10
3.18
4.04
1.00
9.09
3.04
3.00
f1 (ppm)

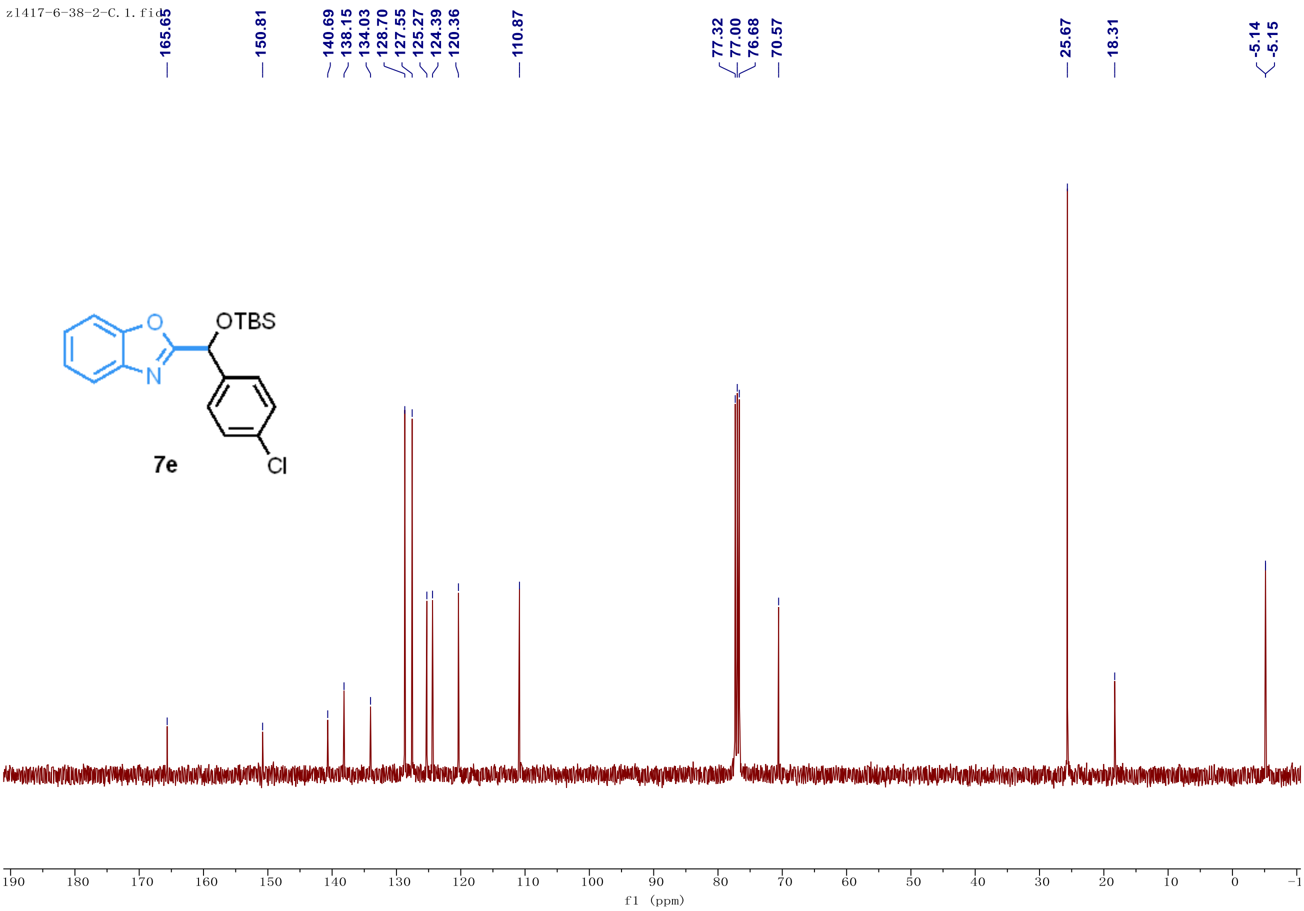

zl417-6-38-2-C.1.fid
165.65
150.81
140.69
138.15
134.03
128.70
127.55
125.27
124.39
120.36
110.87
77.32
77.00
76.68
70.57
25.67
18.31
-5.14
-5.15
OTBS
O
N
Cl
7e
190 180 170 160 150 140 130 120 110 100 90 80 70 60 50 40 30 20 10 0
f1 (ppm)

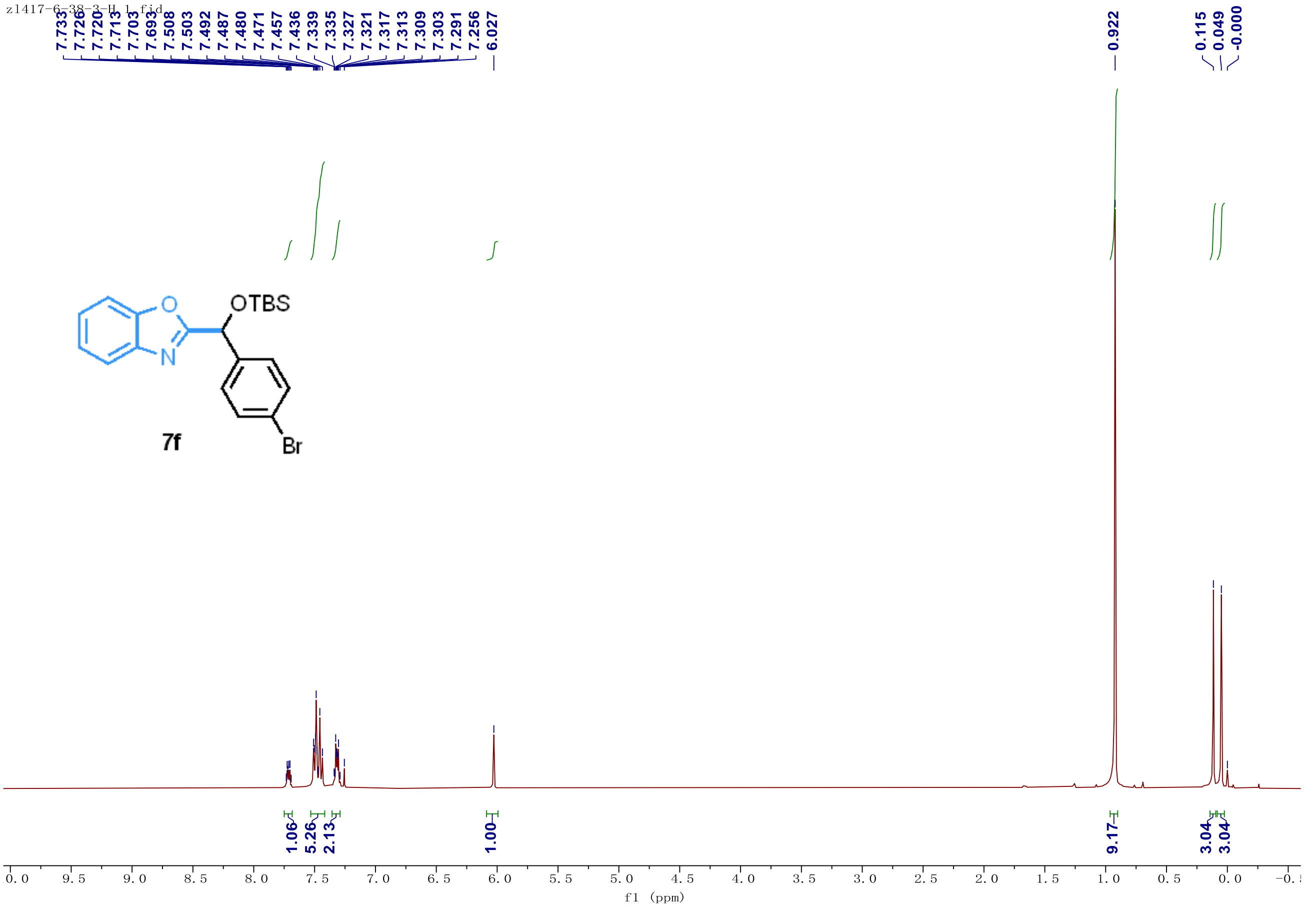

7.733
7.726
7.720
7.713
7.703
7.693
7.508
7.503
7.492
7.487
7.480
7.471
7.457
7.436
7.339
7.335
7.327
7.321
7.317
7.313
7.309
7.303
7.291
7.256
6.027
0.922
0.115
0.049
-0.000
OTBS
O
N
Br
7f
1.06
5.26
2.13
1.00
9.17
3.04
3.04
9.5 9.0 8.5 8.0 7.5 7.0 6.5 6.0 5.5 5.0 4.5 4.0 3.5 3.0 2.5 2.0 1.5 1.0 0.5 0.0
f1 (ppm)

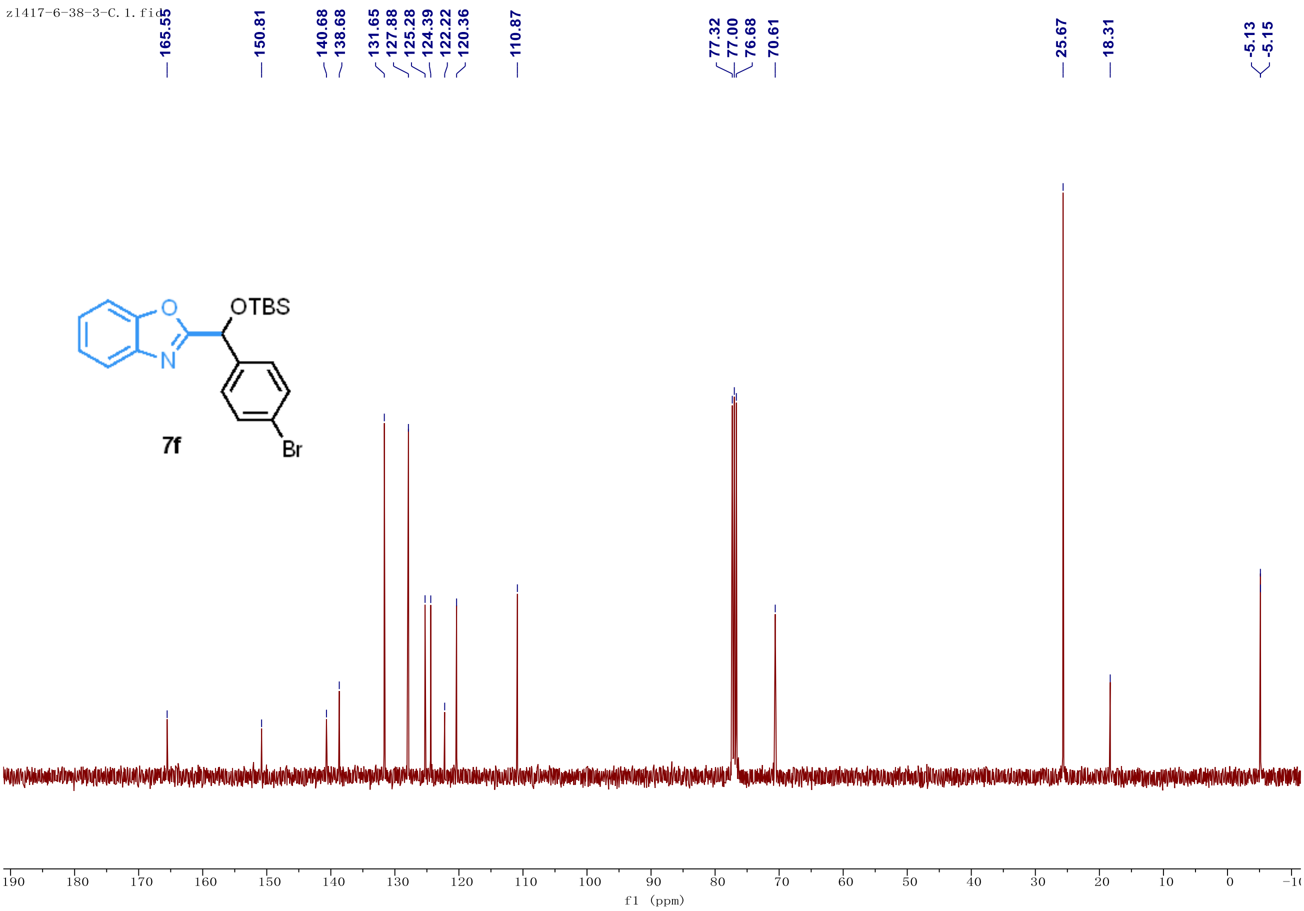
z1417-6-38-3-C.1.fid
165.55
150.81
140.68
138.68
131.65
127.88
125.28
124.39
122.22
120.36
110.87
77.32
77.00
76.68
70.61
25.67
18.31
-5.13
-5.15
O
N
OTBS
Br
7f
f1 (ppm)

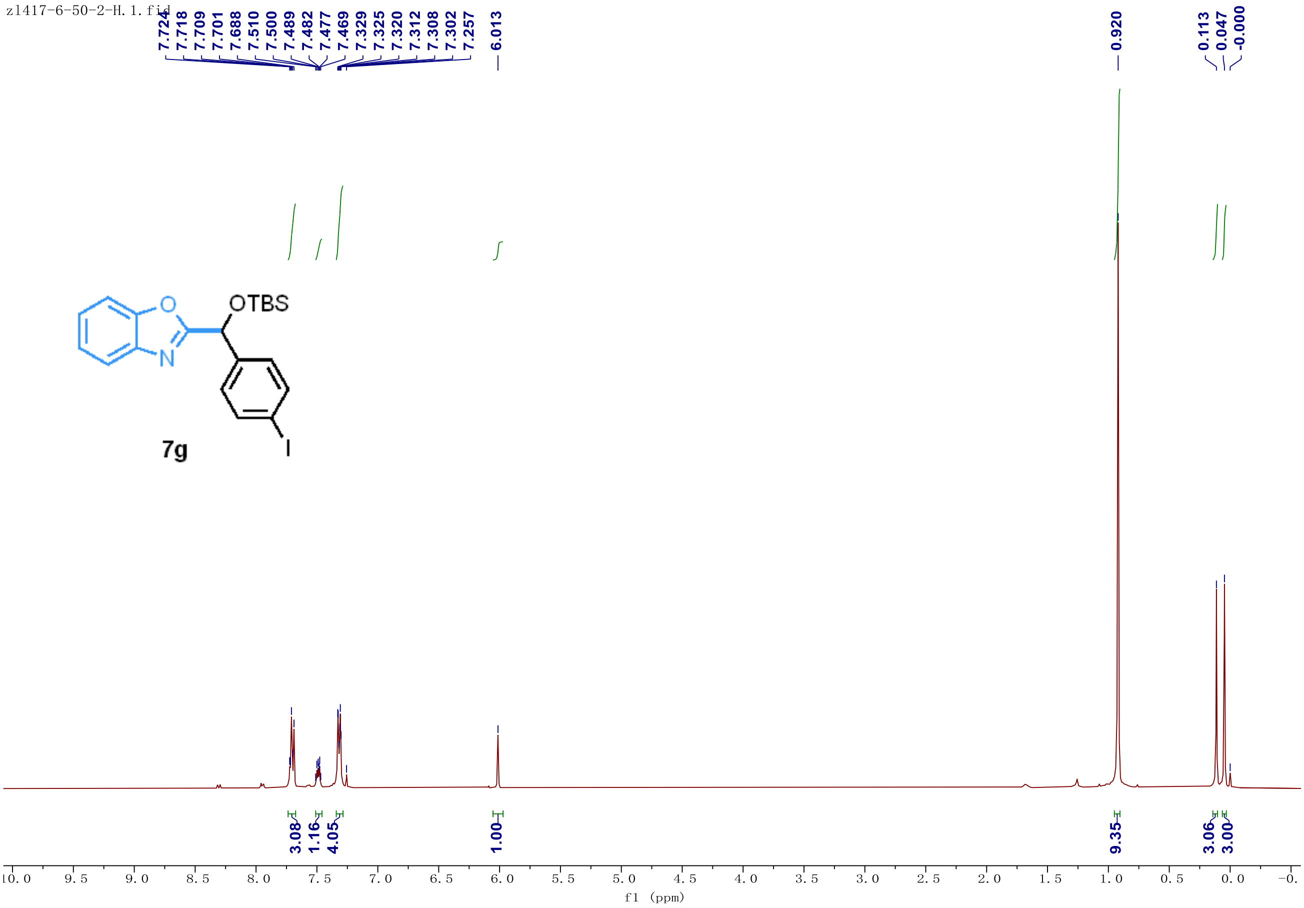
z1417-6-50-2-H.1.fid
7.724
7.718
7.709
7.701
7.688
7.510
7.500
7.489
7.482
7.477
7.469
7.329
7.325
7.320
7.312
7.308
7.302
7.257
6.013
0.920
0.113
0.047
-0.000
O
N
OTBS
I
7g
3.08
1.16
4.05
1.00
9.35
3.06
3.00
f1 (ppm)

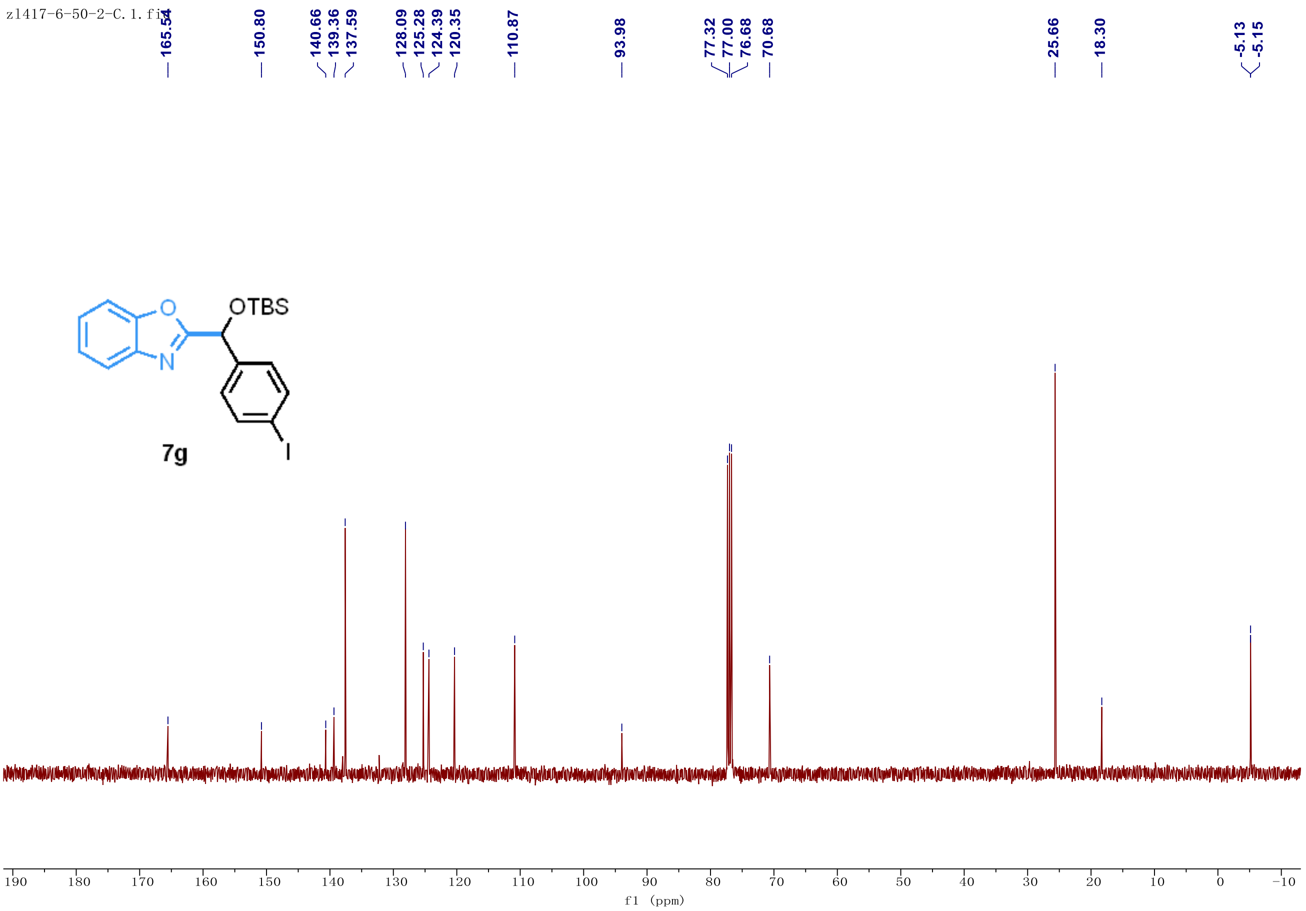

165.54
150.80
140.66
139.36
137.59
128.09
125.28
124.39
120.35
110.87
93.98
77.32
77.00
76.68
70.68
25.66
18.30
-5.13
-5.15
OTBS
O
N
I
7g
190 180 170 160 150 140 130 120 110 100 90 80 70 60 50 40 30 20 10 0 -10
f1 (ppm)

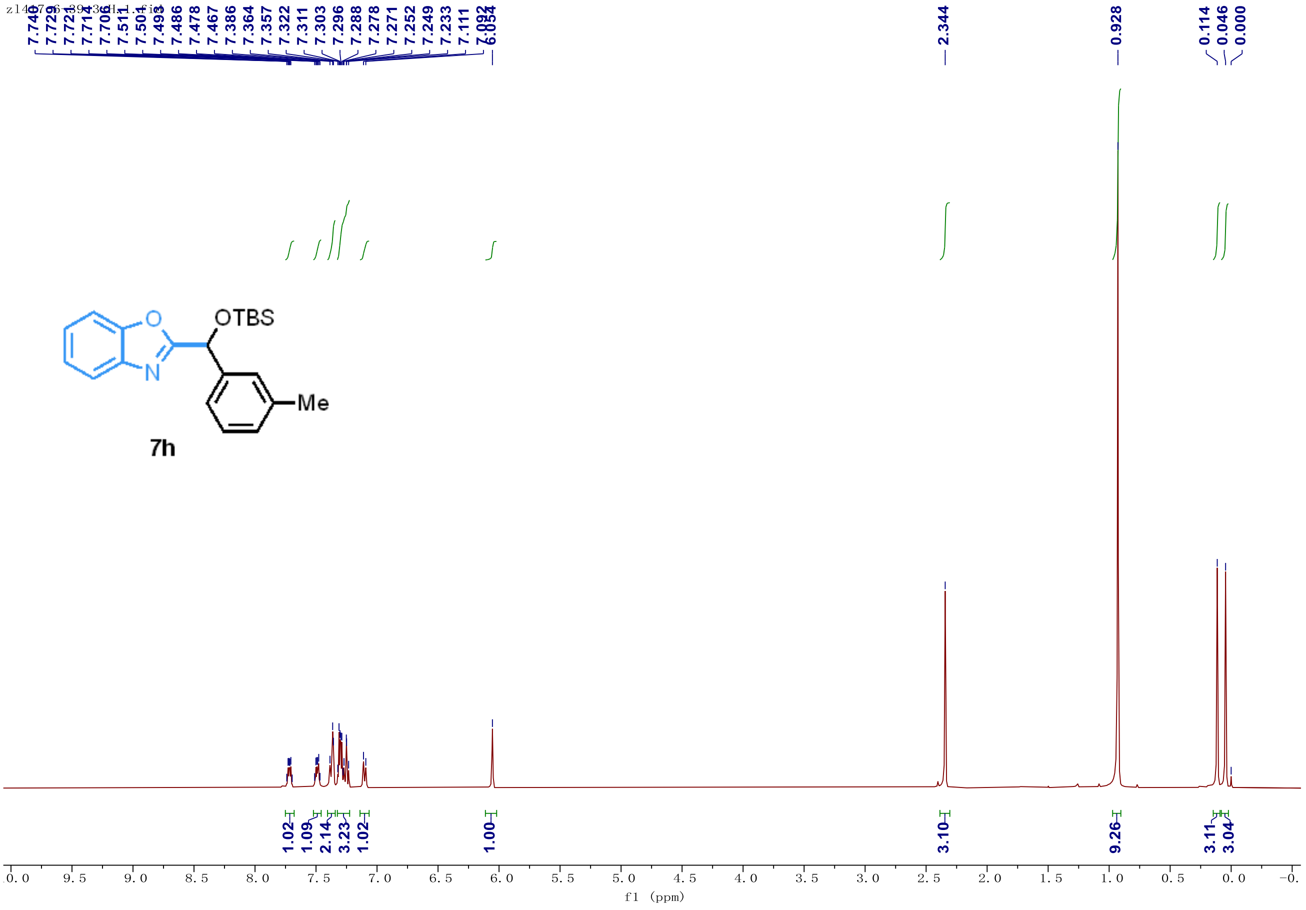

7.740
7.729
7.723
7.714
7.706
7.511
7.504
7.493
7.486
7.478
7.467
7.386
7.364
7.357
7.322
7.311
7.303
7.296
7.288
7.278
7.271
7.252
7.249
7.233
7.111
7.092
6.054
2.344
0.928
0.114
0.046
0.000
OTBS
O
N
Me
7h
1.02
1.09
2.14
3.23
1.02
1.00
3.10
9.26
3.11
3.04
f1 (ppm)

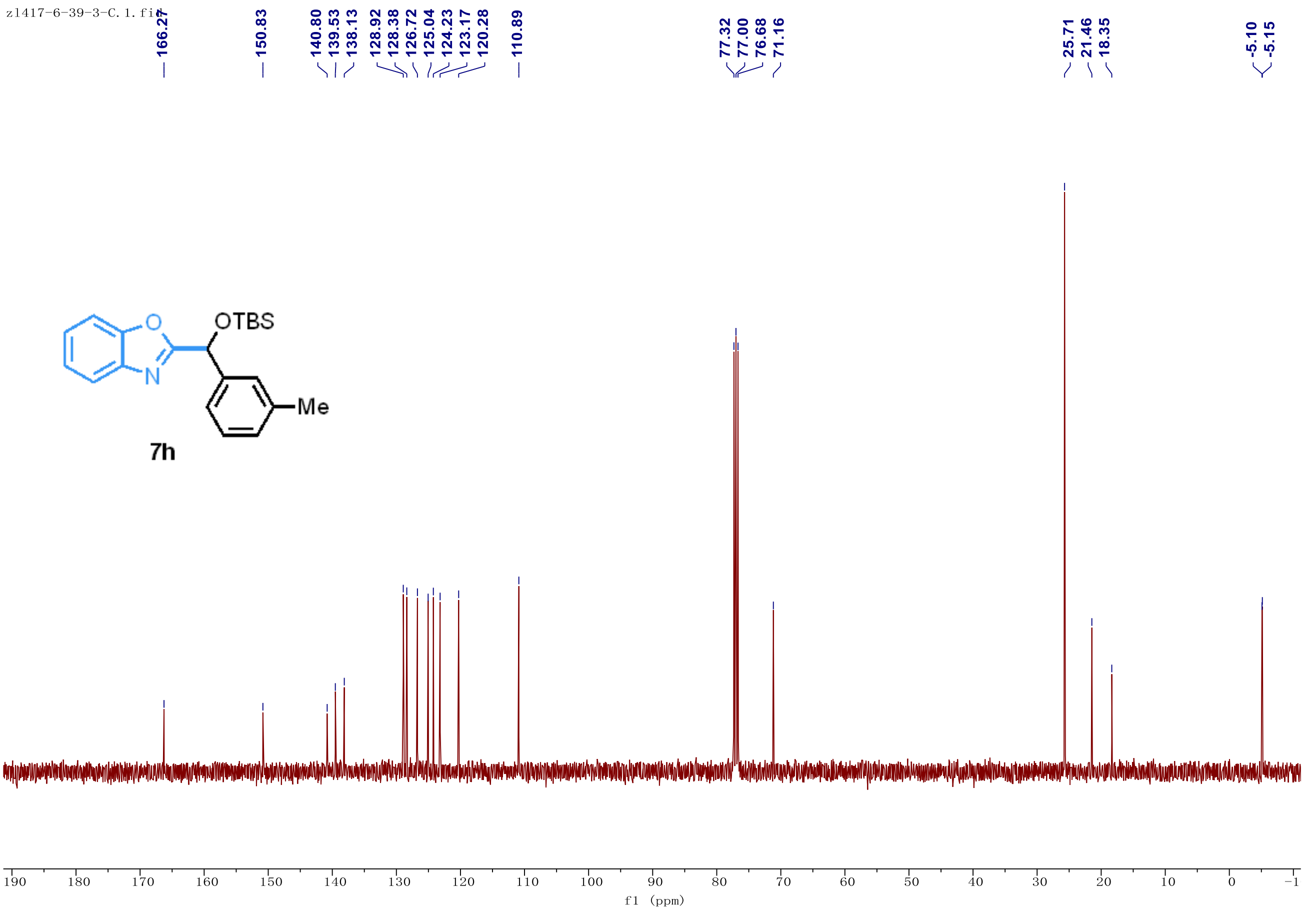
z1417-6-39-3-C.1.fid
166.27
150.83
140.80
139.53
138.13
128.92
128.38
126.72
125.04
124.23
123.17
120.28
110.89
77.32
77.00
76.68
71.16
25.71
21.46
18.35
-5.10
-5.15
O
N
OTBS
Me
7h
190 180 170 160 150 140 130 120 110 100 90 80 70 60 50 40 30 20 10 0 -1
f1 (ppm)

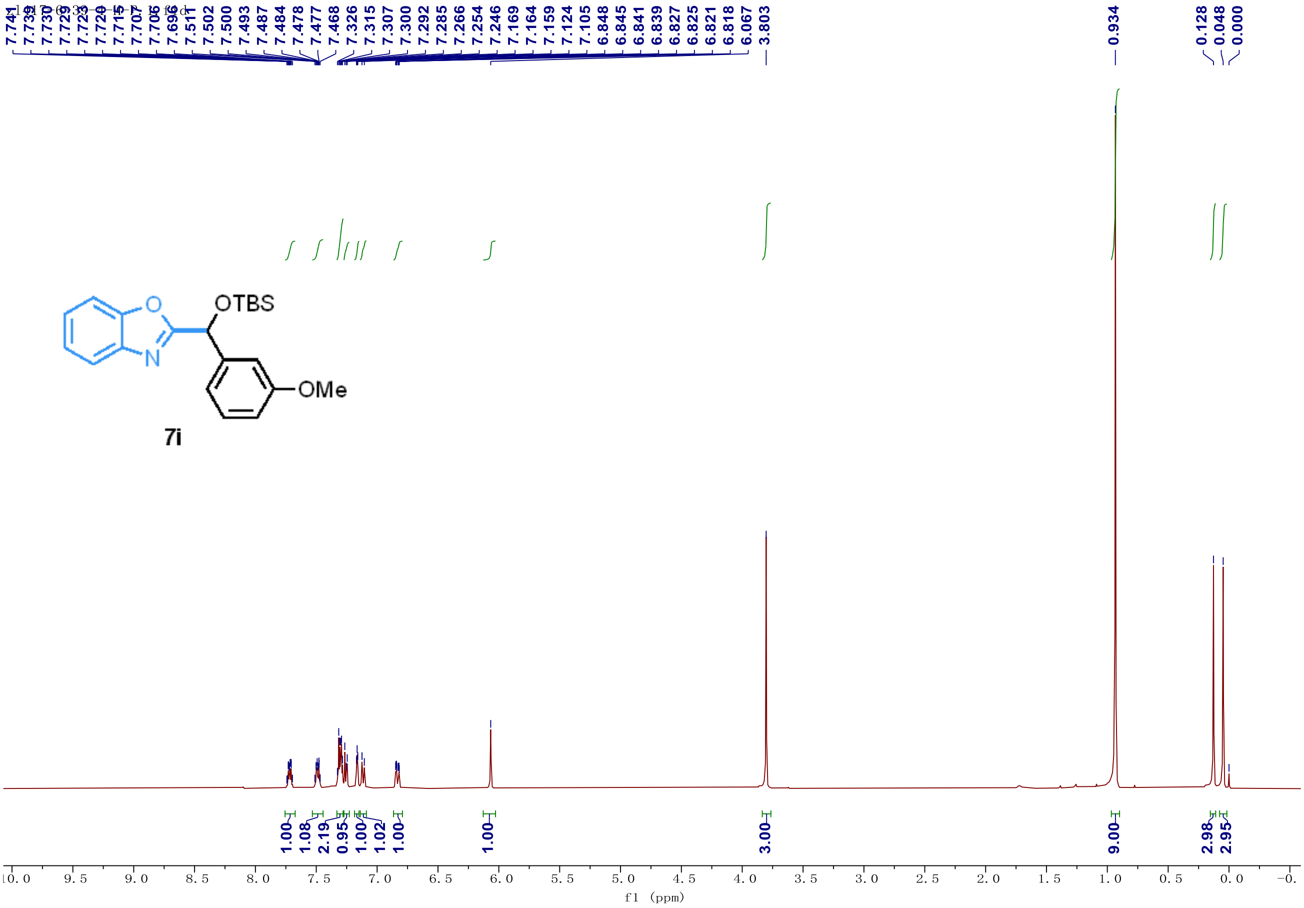
7.741
7.739
7.730
7.729
7.723
7.720
7.715
7.707
7.706
7.696
7.511
7.502
7.500
7.493
7.487
7.484
7.478
7.477
7.468
7.326
7.315
7.307
7.300
7.292
7.285
7.266
7.254
7.246
7.169
7.164
7.159
7.124
7.105
6.848
6.845
6.841
6.839
6.827
6.825
6.821
6.818
6.067
3.803
0.934
0.128
0.048
0.000
O
N
OTBS
OMe
7i
1.00
1.08
2.19
0.95
1.00
1.02
1.00
1.00
3.00
9.00
2.98
2.95
10.0 9.5 9.0 8.5 8.0 7.5 7.0 6.5 6.0 5.5 5.0 4.5 4.0 3.5 3.0 2.5 2.0 1.5 1.0 0.5 0.0 -0.
f1 (ppm)

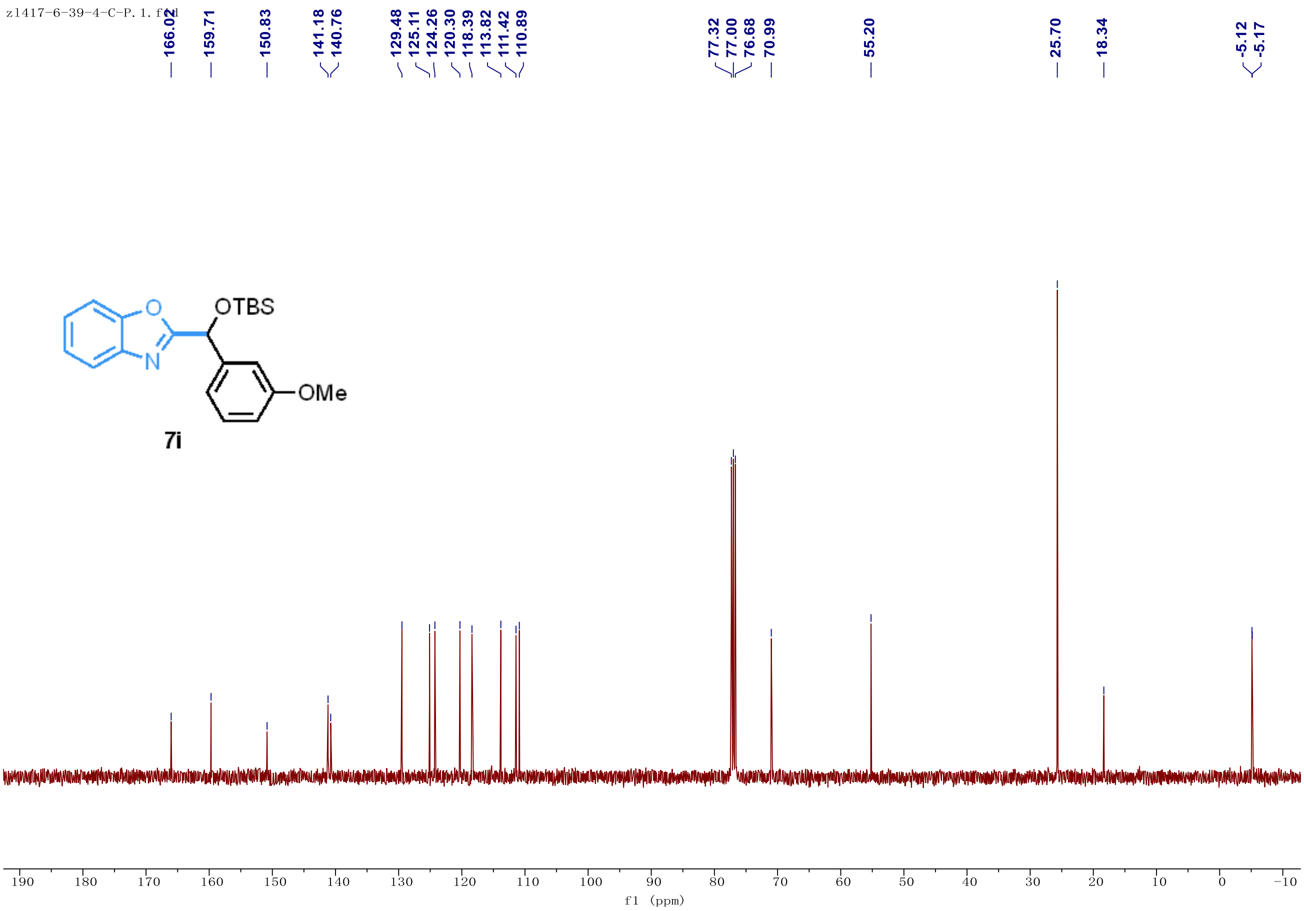
z1417-6-39-4-C-P.1.fid
166.02
159.71
150.83
141.18
140.76
129.48
125.11
124.26
120.30
118.39
113.82
111.42
110.89
77.32
77.00
76.68
70.99
55.20
25.70
18.34
-5.12
-5.17
OTBS
O
N
OMe
7i
f1 (ppm)

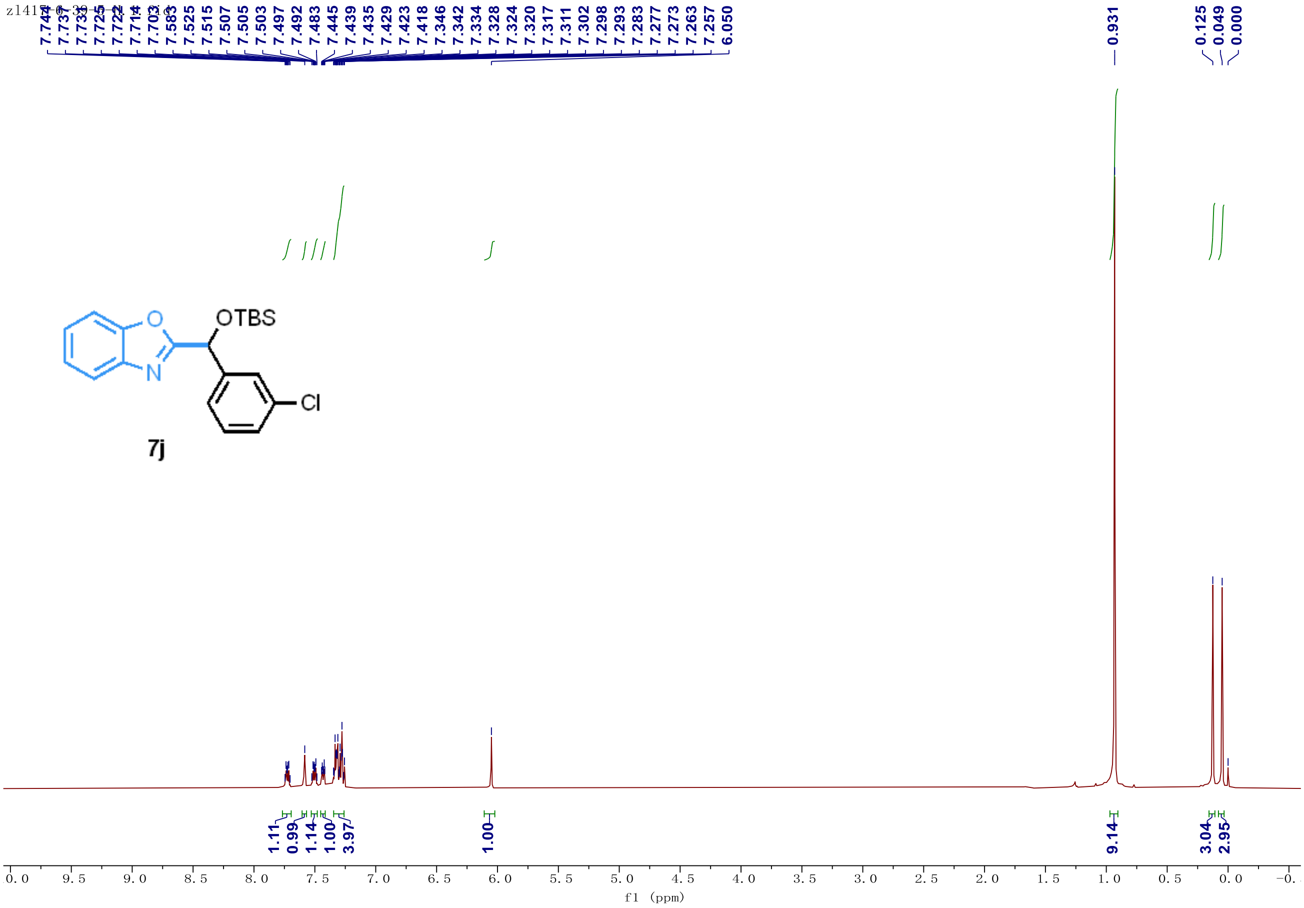
7.744
7.737
7.733
7.725
7.722
7.714
7.703
7.583
7.525
7.515
7.507
7.505
7.503
7.497
7.492
7.483
7.445
7.439
7.435
7.429
7.423
7.418
7.346
7.342
7.334
7.328
7.324
7.320
7.317
7.311
7.302
7.298
7.293
7.283
7.277
7.273
7.263
7.257
6.050
0.931
0.125
0.049
0.000
OTBS
O
N
Cl
7j
1.11
0.99
1.14
1.00
3.97
1.00
9.14
3.04
2.95
f1 (ppm)

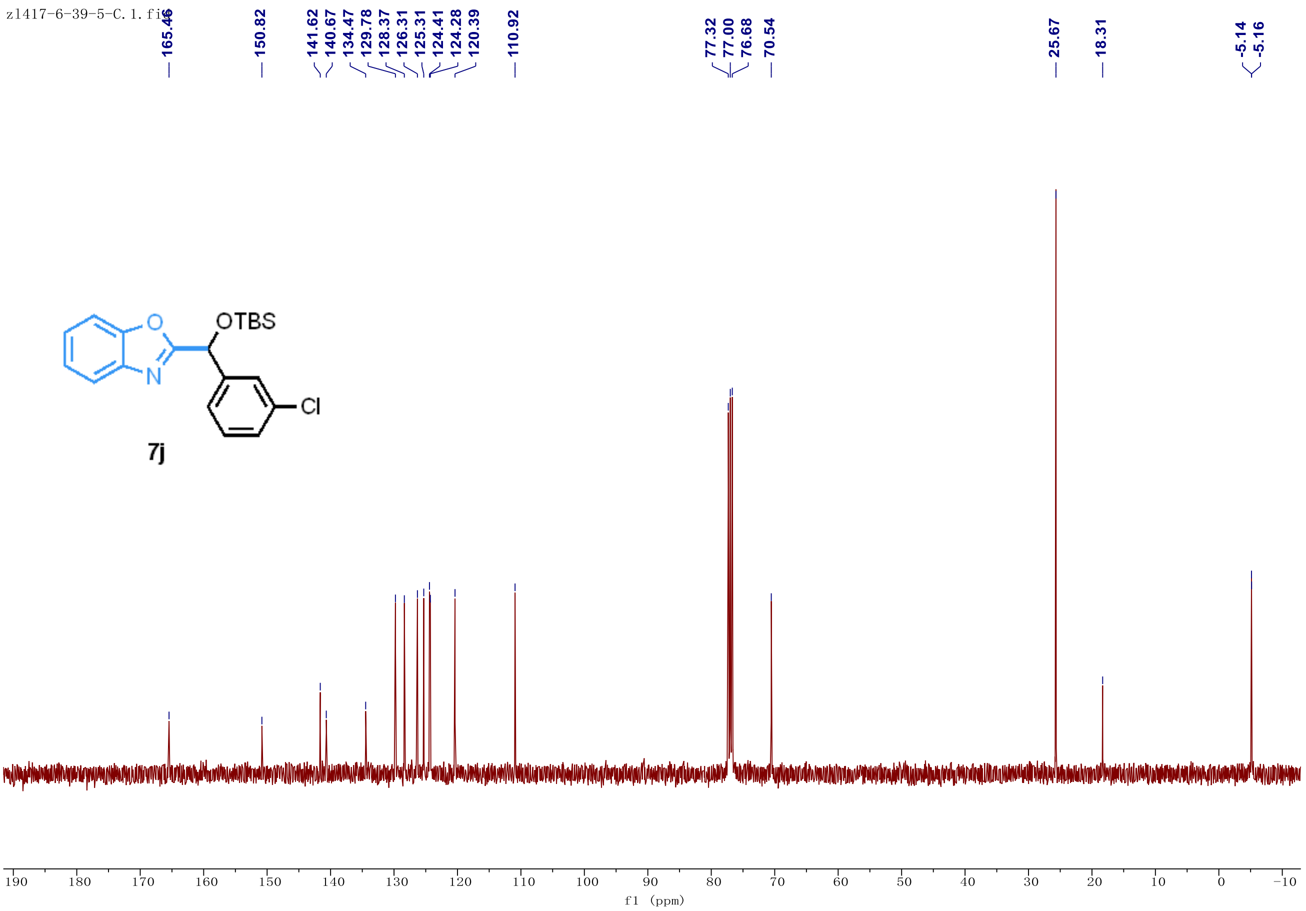
z1417-6-39-5-C.1.fid
165.46
150.82
141.62
140.67
134.47
129.78
128.37
126.31
125.31
124.41
124.28
120.39
110.92
77.32
77.00
76.68
70.54
25.67
18.31
-5.14
-5.16
OTBS
N
O
Cl
7j
f1 (ppm)

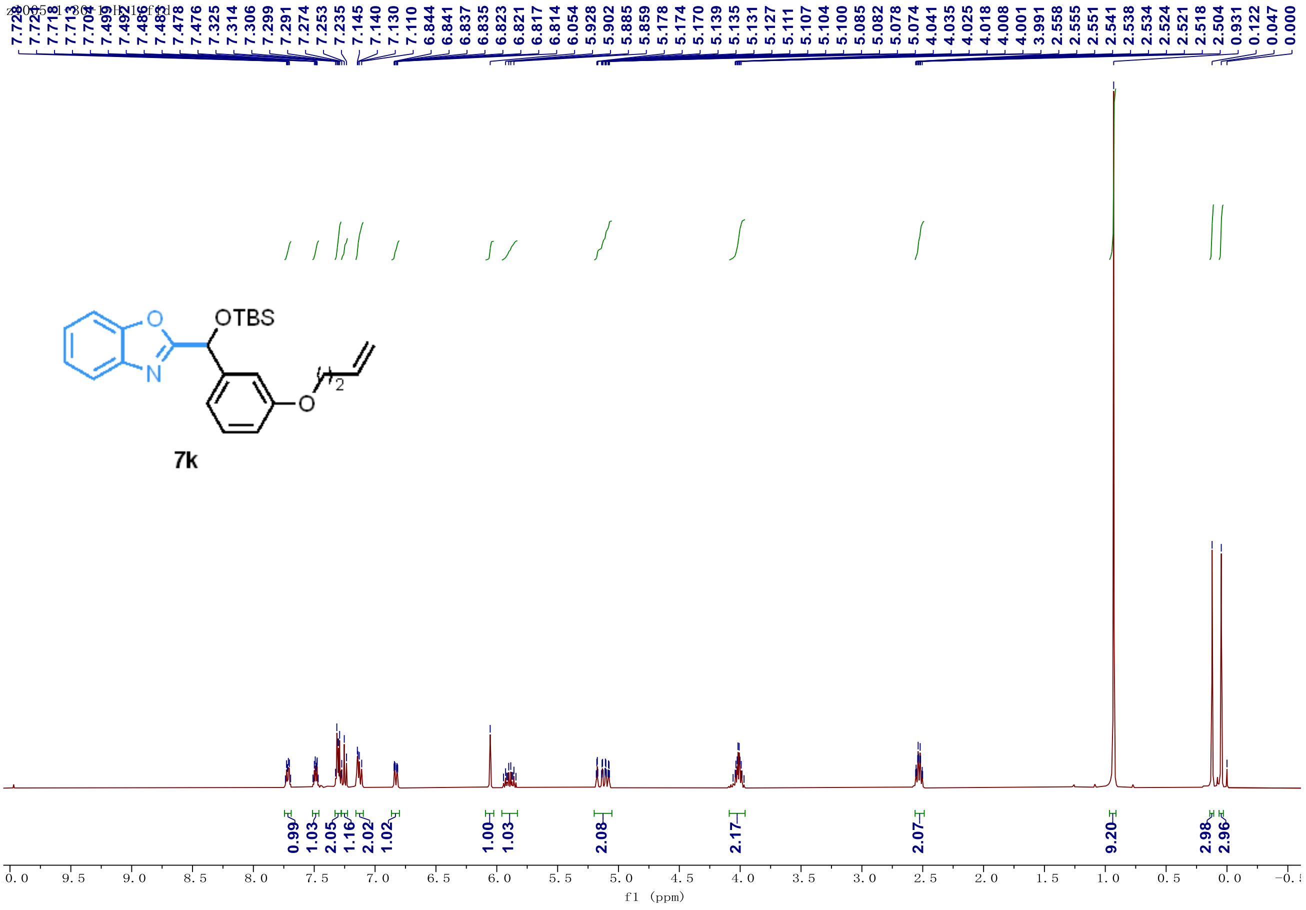
7.499
7.492
7.486
7.483
7.478
7.476
7.325
7.314
7.306
7.299
7.291
7.274
7.253
7.235
7.145
7.140
7.130
7.110
6.844
6.841
6.837
6.835
6.823
6.821
6.817
6.814
6.054
5.928
5.902
5.885
5.859
5.178
5.174
5.170
5.139
5.135
5.131
5.127
5.111
5.107
5.104
5.100
5.085
5.082
5.078
5.074
4.041
4.035
4.025
4.018
4.008
4.001
3.991
2.558
2.555
2.551
2.541
2.538
2.534
2.524
2.521
2.518
2.504
0.931
0.122
0.047
0.000
OTBS
N
O
O
2
7k
0.99
1.03
2.05
1.16
2.02
1.02
1.00
1.03
2.08
2.17
2.07
9.20
2.98
2.96
f1 (ppm)

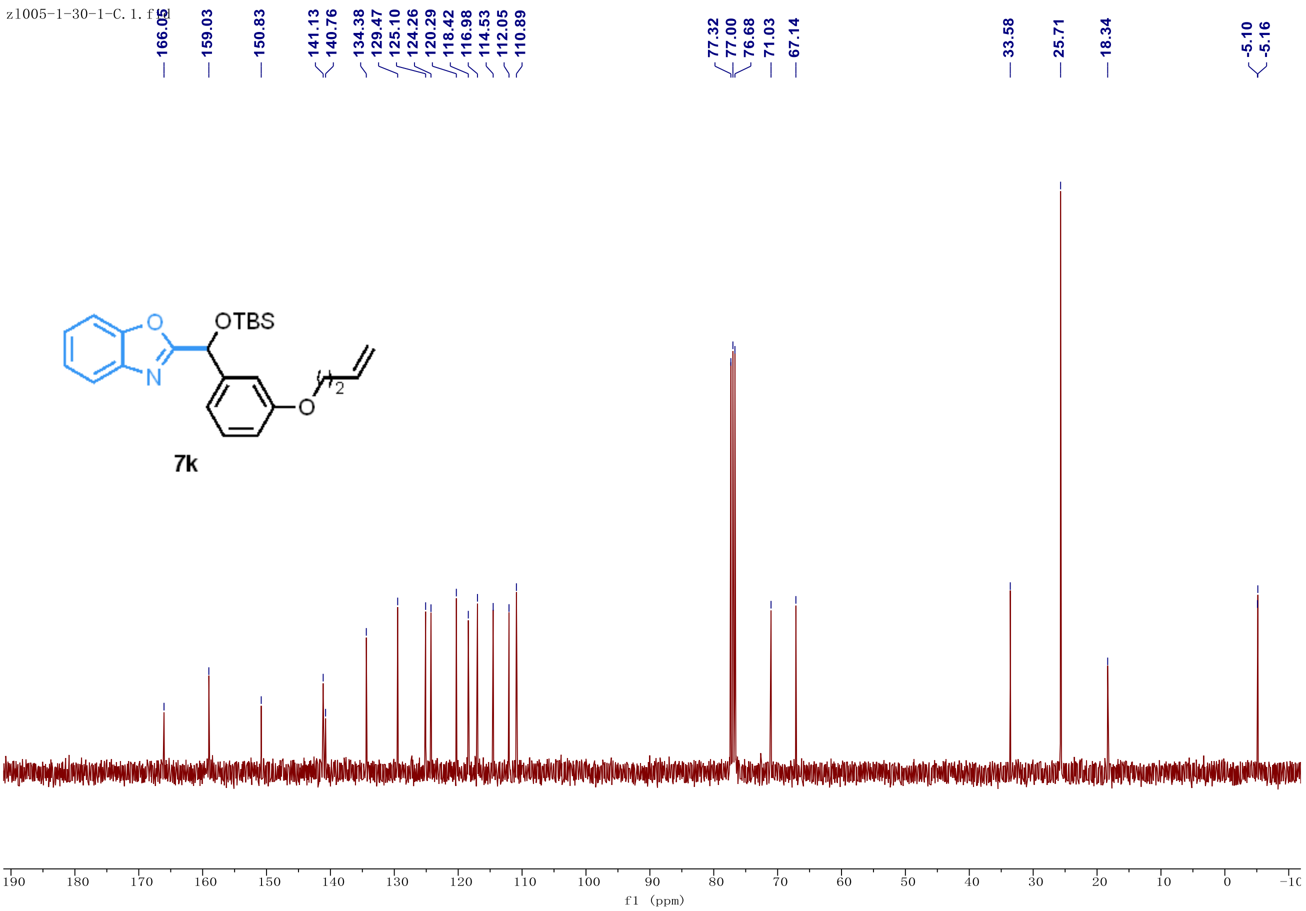
z1005-1-30-1-C.1.fid
166.05
159.03
150.83
141.13
140.76
134.38
129.47
125.10
124.26
120.29
118.42
116.98
114.53
112.05
110.89
77.32
77.00
76.68
71.03
67.14
33.58
25.71
18.34
-5.10
-5.16
OTBS
O
N
O
2
7k
f1 (ppm)

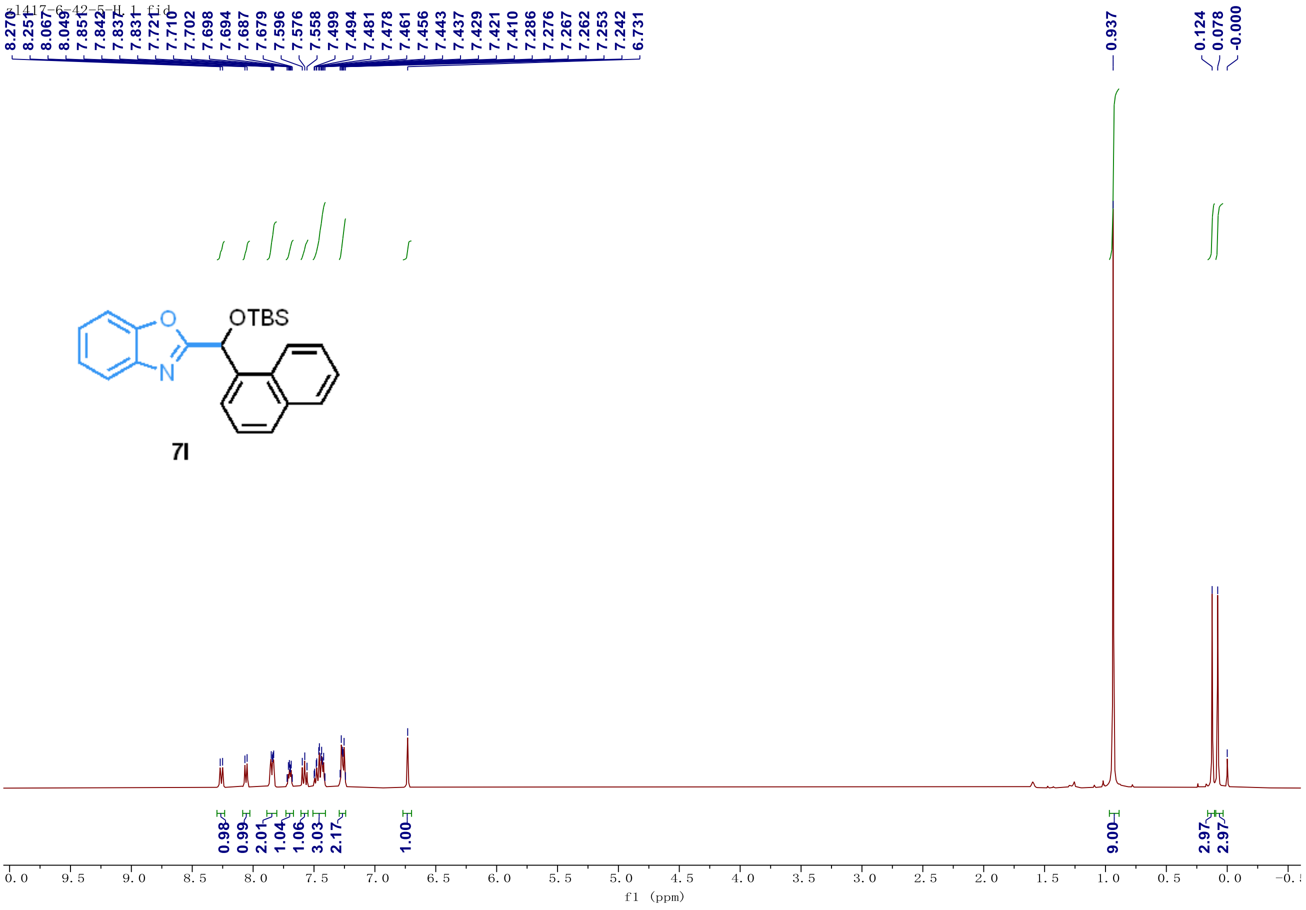
8.270
8.251
8.067
8.049
7.851
7.842
7.837
7.831
7.721
7.710
7.702
7.698
7.694
7.687
7.679
7.596
7.576
7.558
7.499
7.494
7.481
7.478
7.461
7.456
7.443
7.437
7.429
7.421
7.410
7.286
7.276
7.267
7.262
7.253
7.242
6.731
0.937
0.124
0.078
-0.000
OTBS
O
N
7l
0.98
0.99
2.01
1.04
1.06
3.03
2.17
1.00
9.00
2.97
2.97
f1 (ppm)

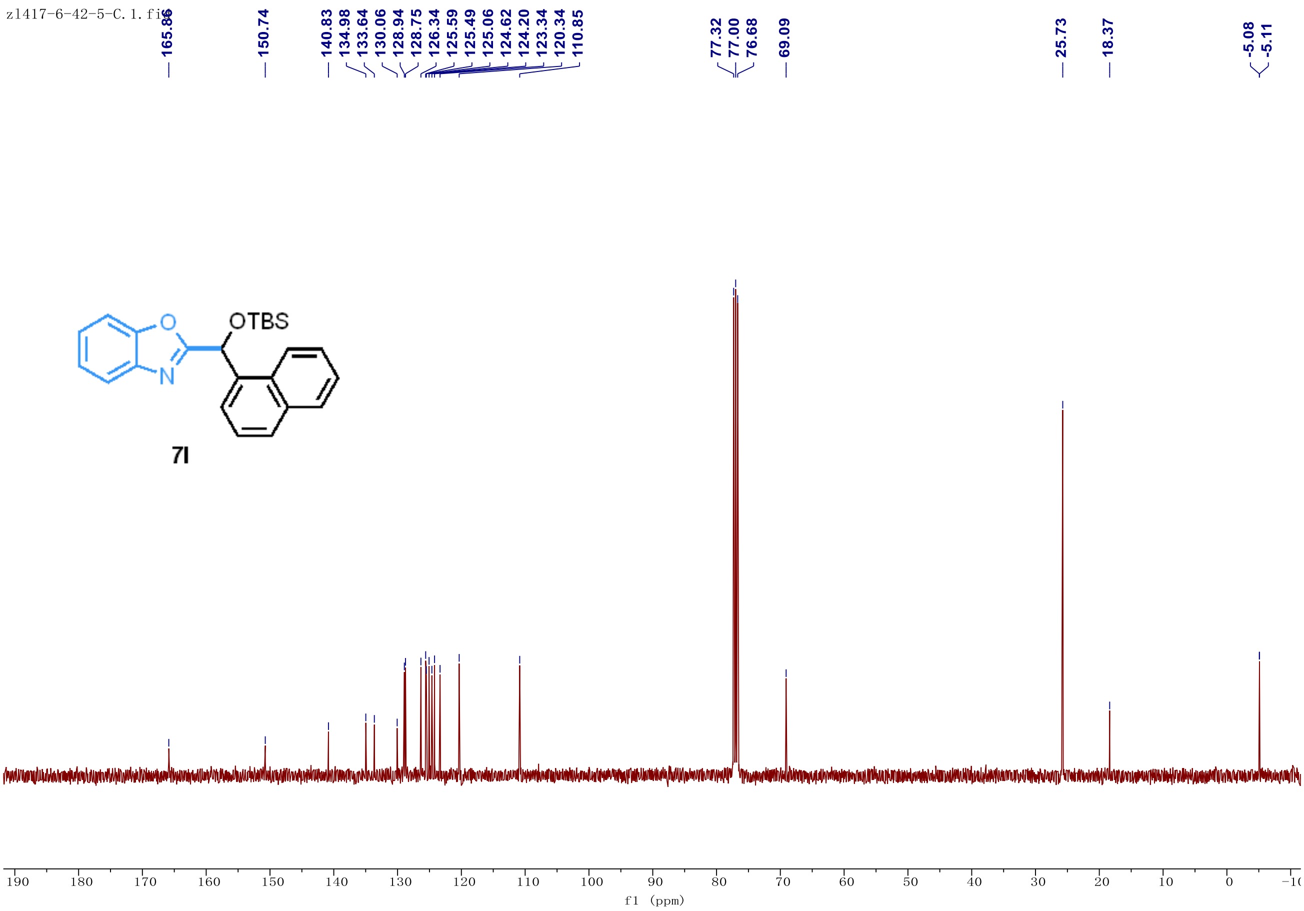
z1417-6-42-5-C.1.fid
165.86
150.74
140.83
134.98
133.64
130.06
128.94
128.75
126.34
125.59
125.49
125.06
124.62
124.20
123.34
120.34
110.85
77.32
77.00
76.68
69.09
25.73
18.37
-5.08
-5.11
OTBS
O
N
7l
190 180 170 160 150 140 130 120 110 100 90 80 70 60 50 40 30 20 10 0
f1 (ppm)

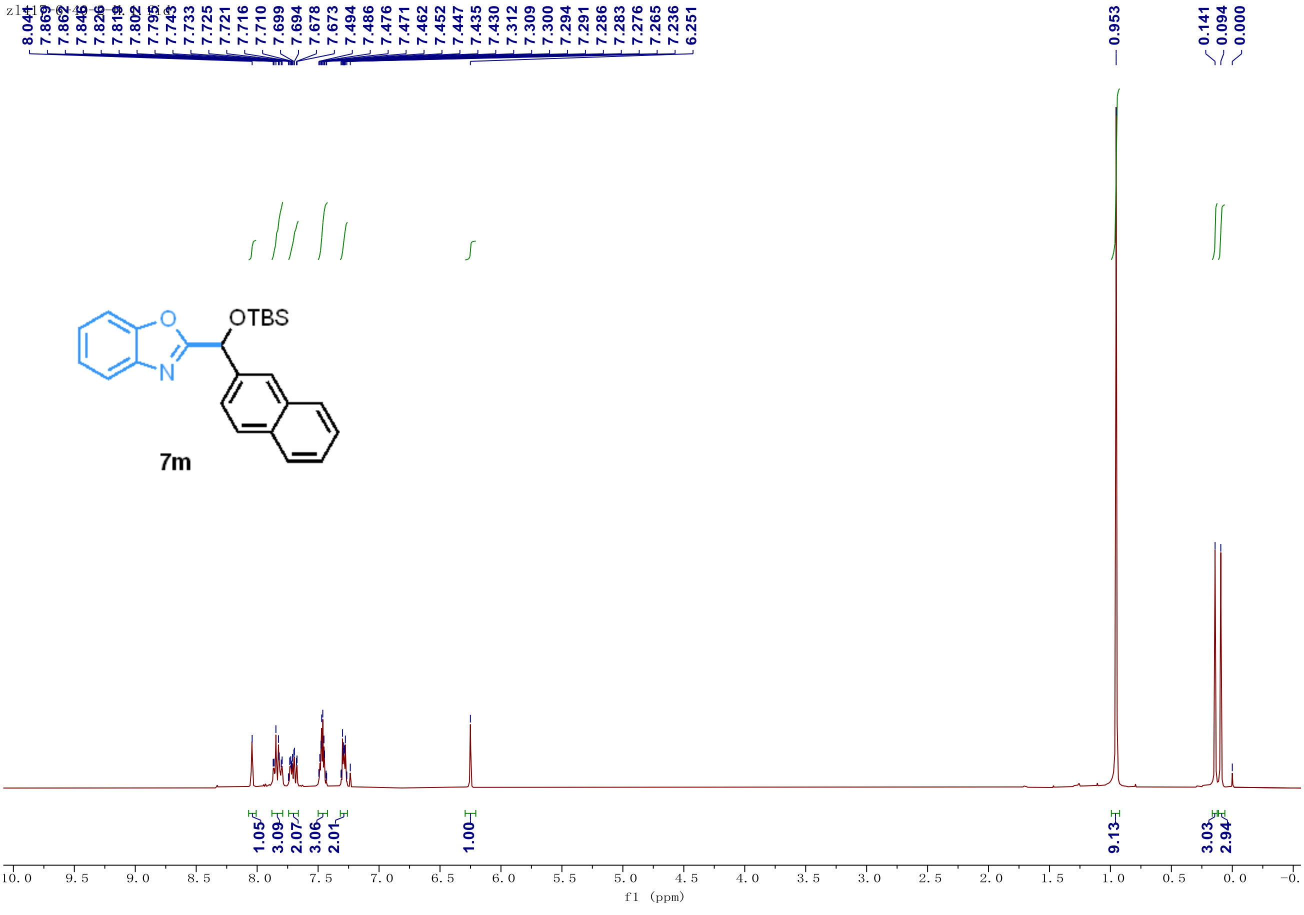
[illegible]
8.041
7.869
7.862
7.846
7.826
7.819
7.802
7.795
7.743
7.733
7.725
7.721
7.716
7.710
7.699
7.694
7.678
7.673
7.494
7.486
7.476
7.471
7.462
7.452
7.447
7.435
7.430
7.312
7.309
7.300
7.294
7.291
7.286
7.283
7.276
7.265
7.236
6.251
0.953
0.141
0.094
0.000
OTBS
O
N
7m
1.05
3.09
2.07
3.06
2.01
1.00
9.13
3.03
2.94
10.0 9.5 9.0 8.5 8.0 7.5 7.0 6.5 6.0 5.5 5.0 4.5 4.0 3.5 3.0 2.5 2.0 1.5 1.0 0.5 0.0
f1 (ppm)

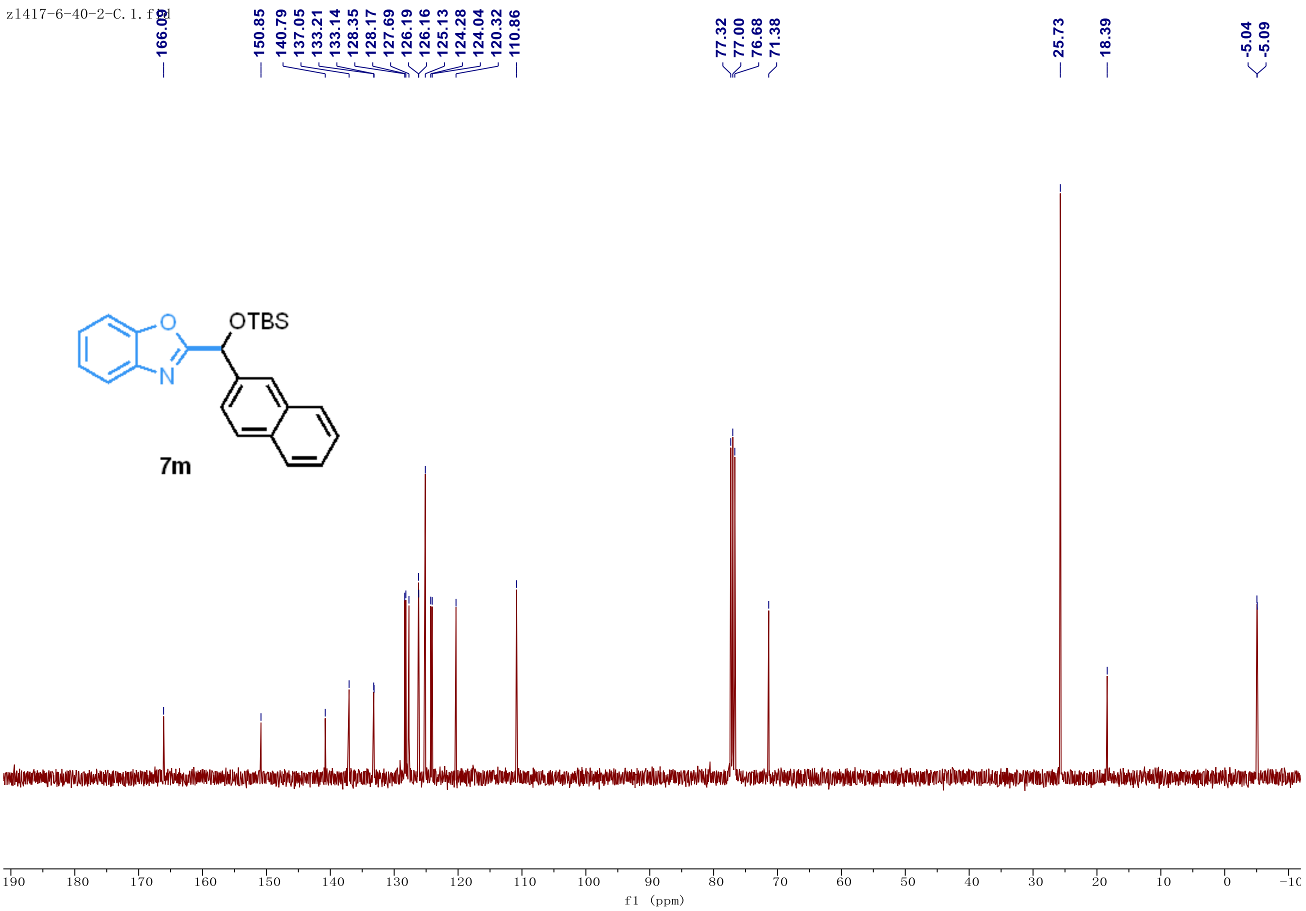
z1417-6-40-2-C.1.fid
166.09
150.85
140.79
137.05
133.21
133.14
128.35
128.17
127.69
126.19
126.16
125.13
124.28
124.04
120.32
110.86
77.32
77.00
76.68
71.38
25.73
18.39
-5.04
-5.09
OTBS
O
N
7m
f1 (ppm)

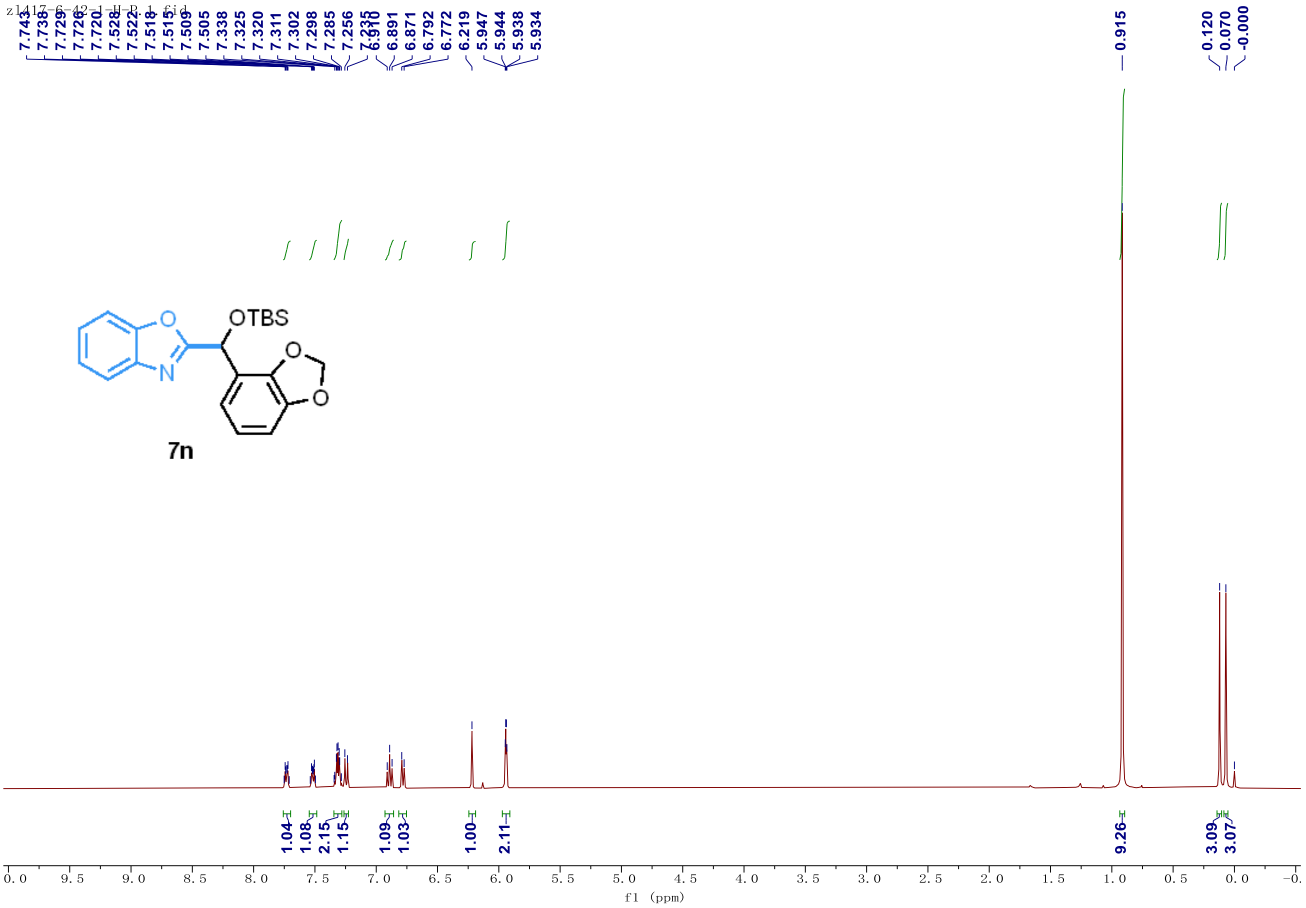
7.743
7.738
7.729
7.726
7.720
7.528
7.522
7.518
7.515
7.509
7.505
7.338
7.325
7.320
7.311
7.302
7.298
7.285
7.256
7.235
6.910
6.891
6.871
6.792
6.772
6.219
5.947
5.944
5.938
5.934
0.915
0.120
0.070
-0.000
OTBS
O
N
7n
1.04
1.08
2.15
1.15
1.09
1.03
1.00
2.11
9.26
3.09
3.07
f1 (ppm)

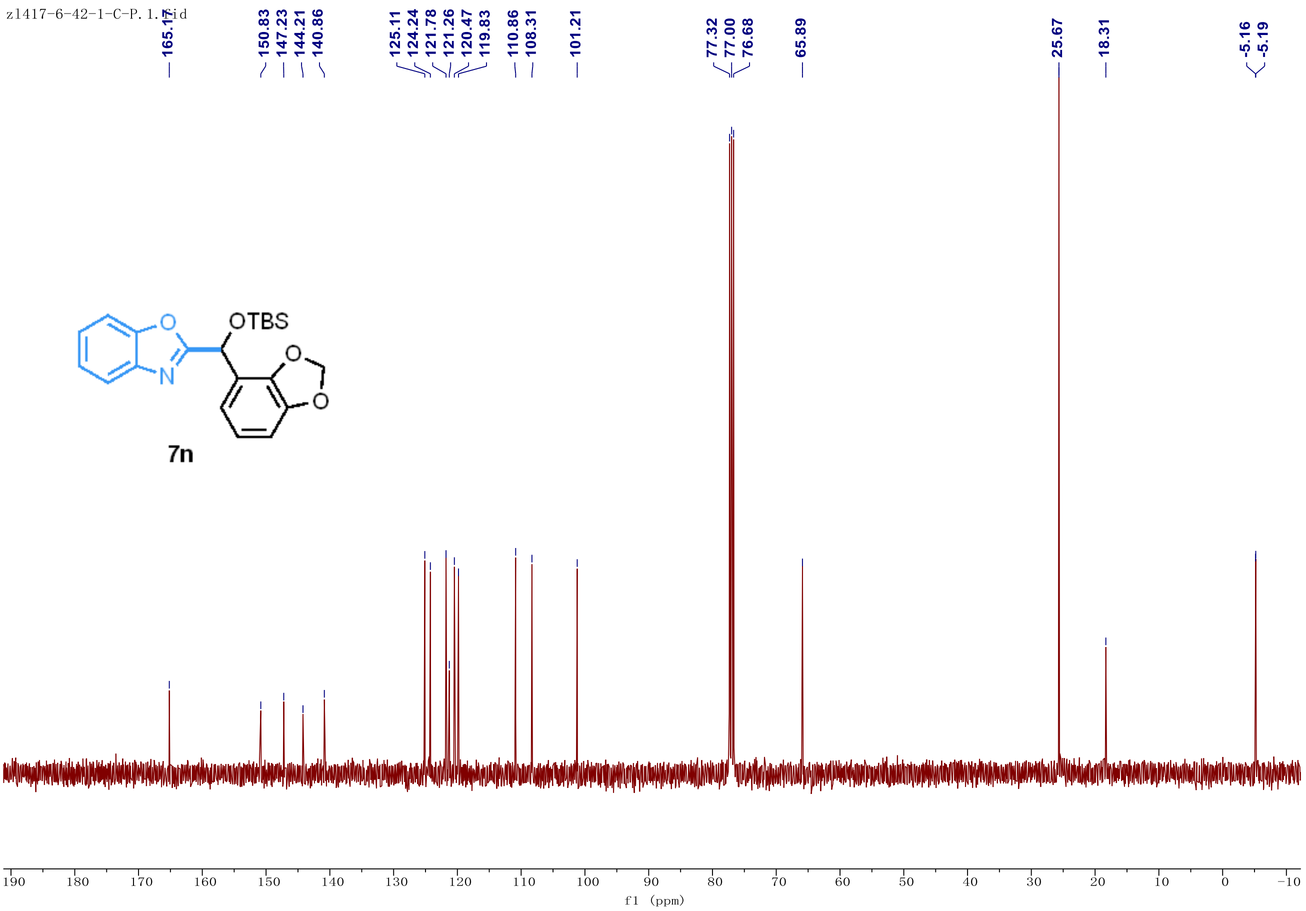
z1417-6-42-1-C-P.1.fid
165.17
150.83
147.23
144.21
140.86
125.11
124.24
121.78
121.26
120.47
119.83
110.86
108.31
101.21
77.32
77.00
76.68
65.89
25.67
18.31
-5.16
-5.19
OTBS
7n
f1 (ppm)

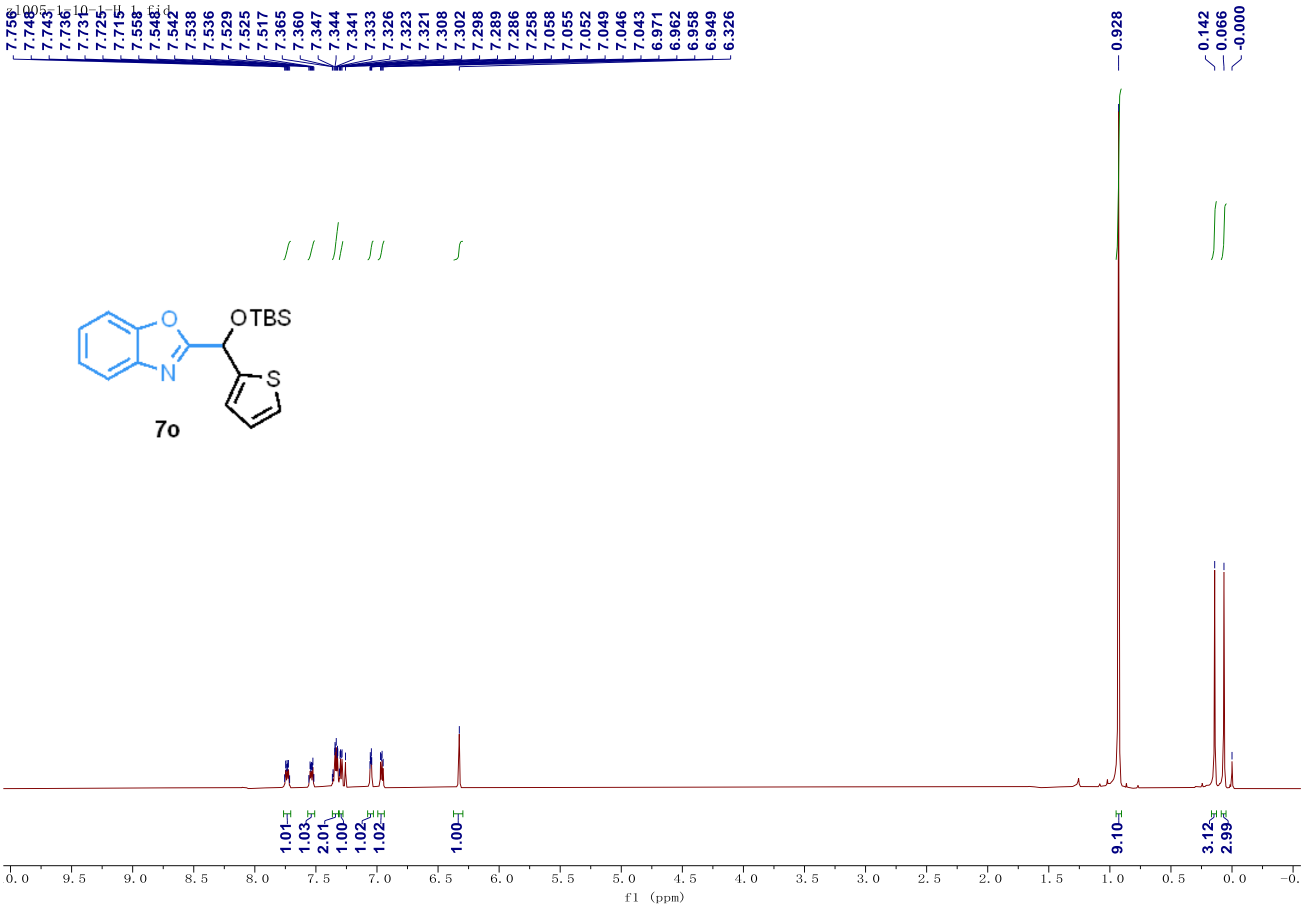
7.756
7.748
7.743
7.736
7.731
7.725
7.715
7.558
7.548
7.542
7.538
7.536
7.529
7.525
7.517
7.365
7.360
7.347
7.344
7.341
7.333
7.326
7.323
7.321
7.308
7.302
7.298
7.289
7.286
7.258
7.058
7.055
7.052
7.049
7.046
7.043
6.971
6.962
6.958
6.949
6.326
0.928
0.142
0.066
-0.000
OTBS
7o
1.01
1.03
2.01
1.00
1.02
1.02
1.00
9.10
3.12
2.99
f1 (ppm)

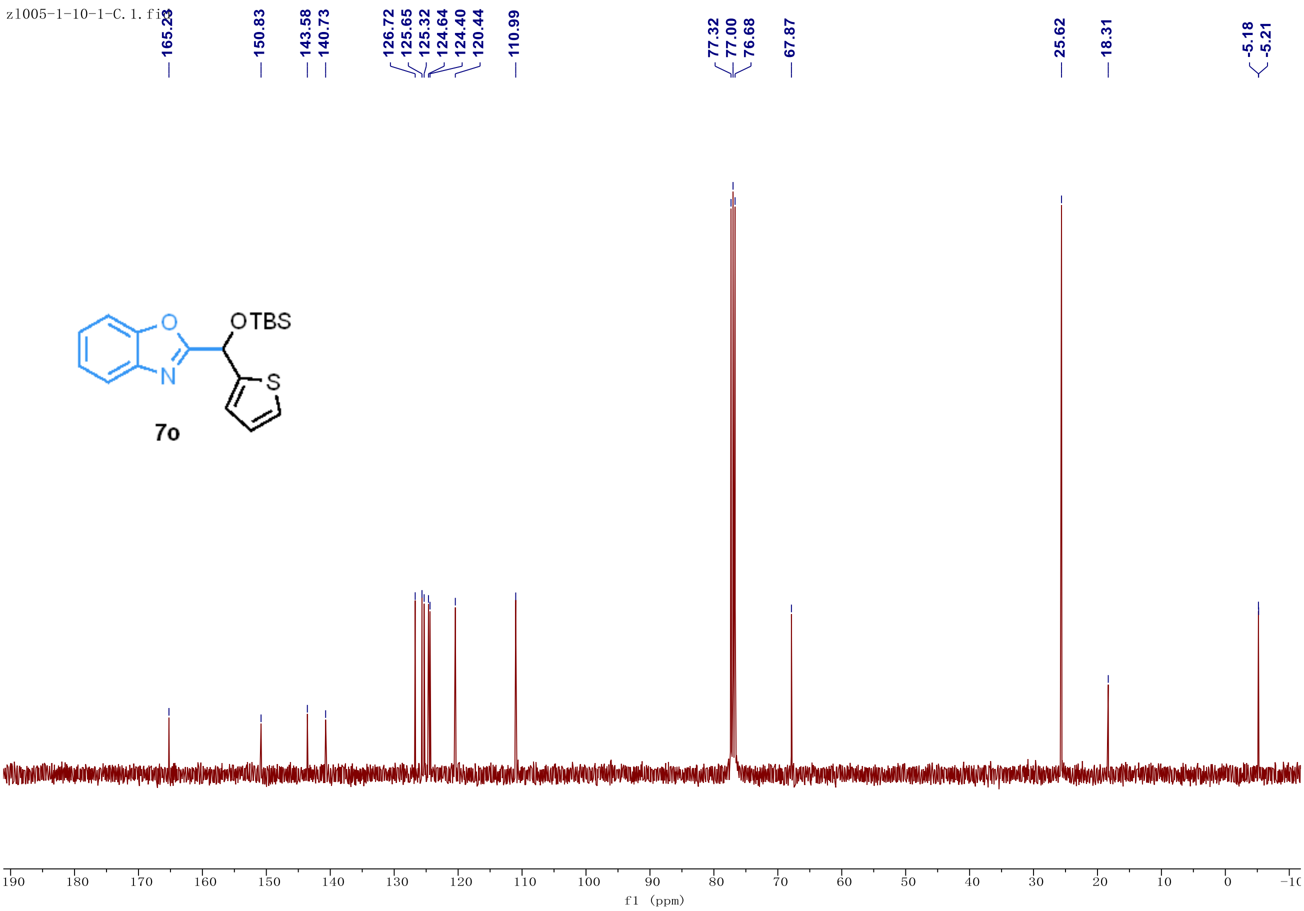
z1005-1-10-1-C.1.fid
165.23
150.83
143.58
140.73
126.72
125.65
125.32
124.64
124.40
120.44
110.99
77.32
77.00
76.68
67.87
25.62
18.31
-5.18
-5.21
OTBS
O
N
S
7o
190 180 170 160 150 140 130 120 110 100 90 80 70 60 50 40 30 20 10 0
f1 (ppm)

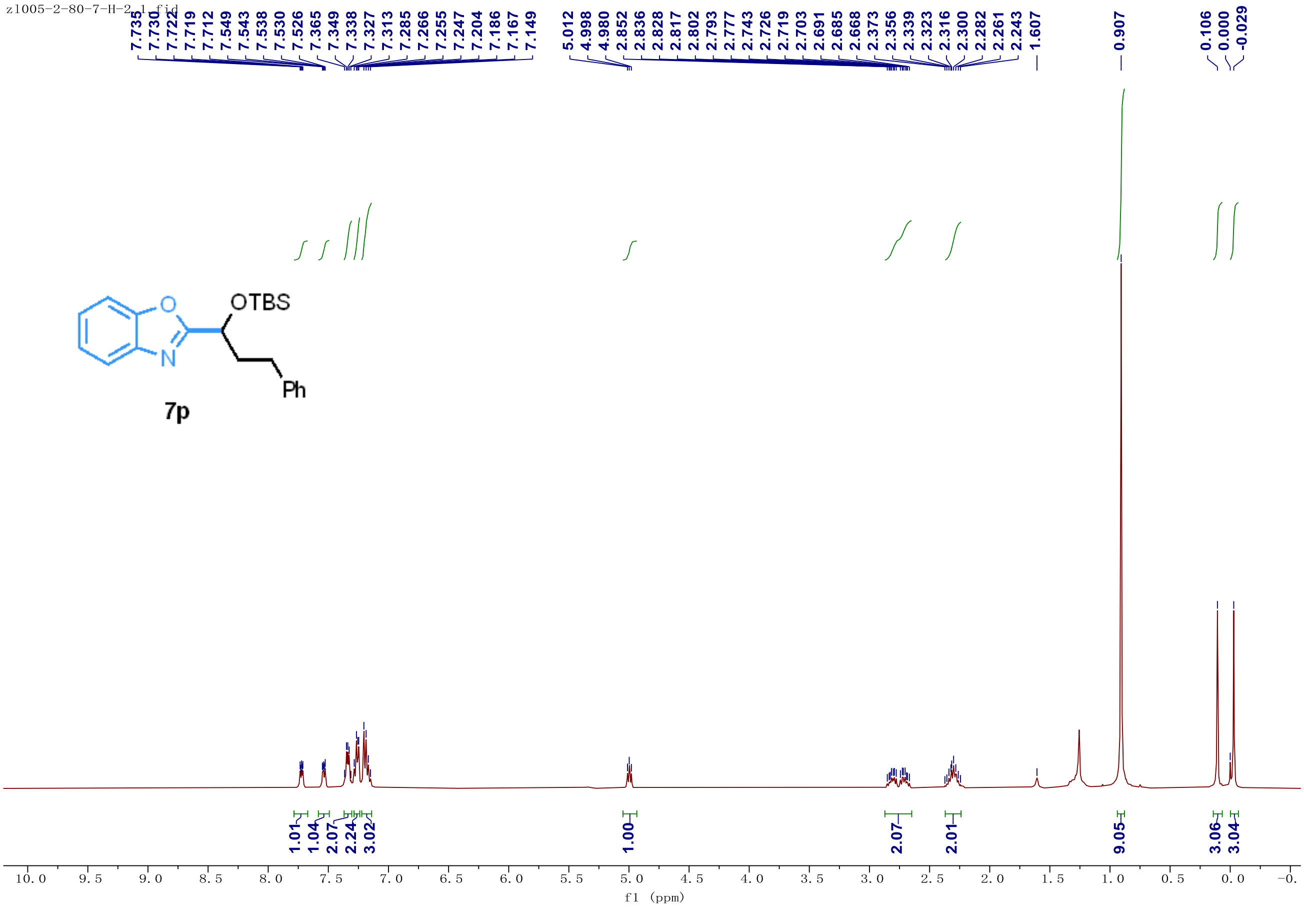
z1005-2-80-7-H-2.1.fid
7.735
7.730
7.722
7.719
7.712
7.549
7.543
7.538
7.530
7.526
7.365
7.349
7.338
7.327
7.313
7.285
7.266
7.255
7.247
7.204
7.186
7.167
7.149
5.012
4.998
4.980
2.852
2.836
2.828
2.817
2.802
2.793
2.777
2.743
2.726
2.719
2.703
2.691
2.685
2.668
2.373
2.356
2.339
2.323
2.316
2.300
2.282
2.261
2.243
1.607
0.907
0.106
0.000
-0.029
OTBS
O
N
Ph
7p
1.01
1.04
2.07
2.24
3.02
1.00
2.07
2.01
9.05
3.06
3.04
10.0 9.5 9.0 8.5 8.0 7.5 7.0 6.5 6.0 5.5 5.0 4.5 4.0 3.5 3.0 2.5 2.0 1.5 1.0 0.5 0.0
f1 (ppm)

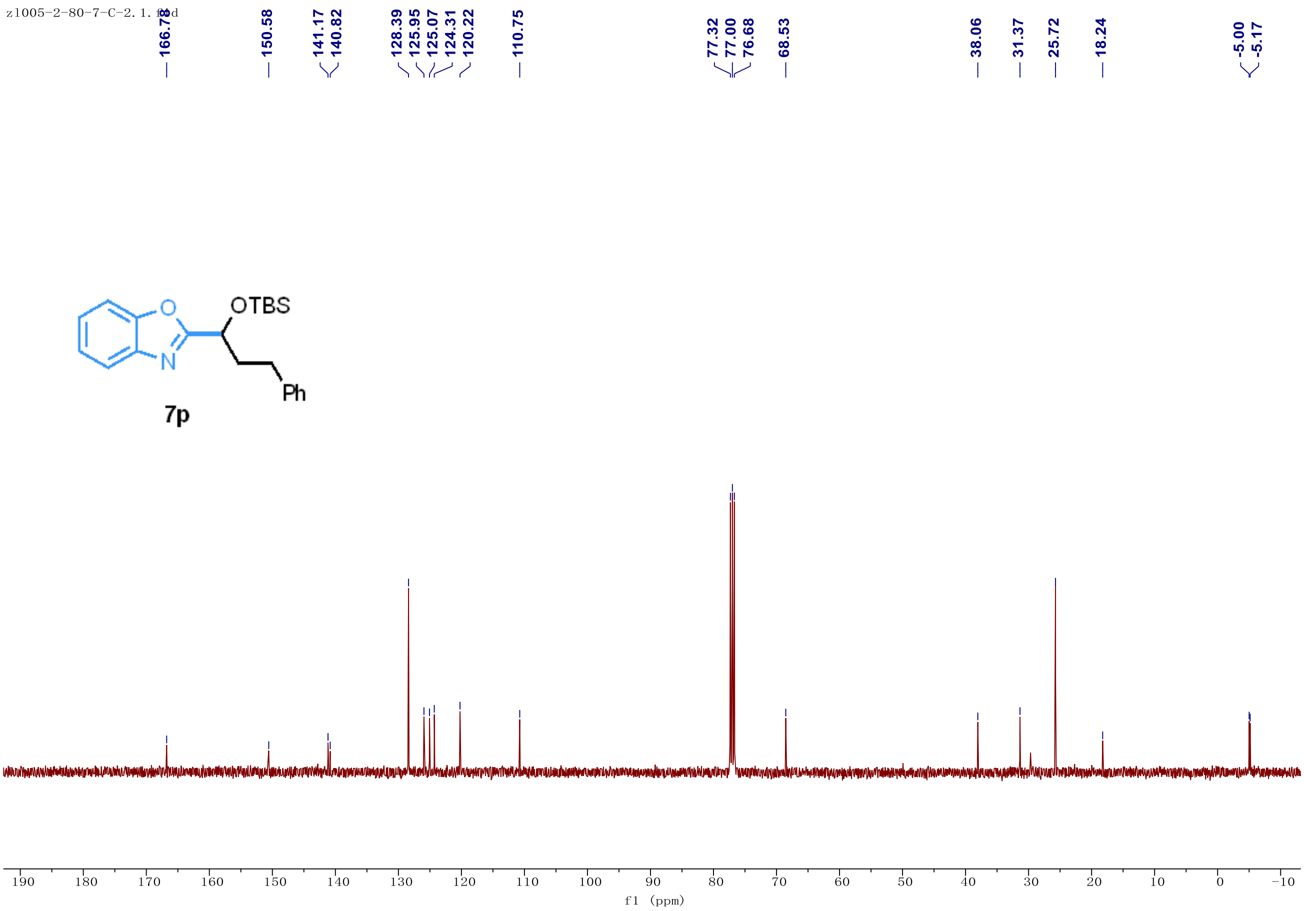
z1005-2-80-7-C-2.1.fid
166.78
150.58
141.17
140.82
128.39
125.95
125.07
124.31
120.22
110.75
77.32
77.00
76.68
68.53
38.06
31.37
25.72
18.24
-5.00
-5.17
OTBS
O
N
Ph
7p
190 180 170 160 150 140 130 120 110 100 90 80 70 60 50 40 30 20 10 0 -10
f1 (ppm)

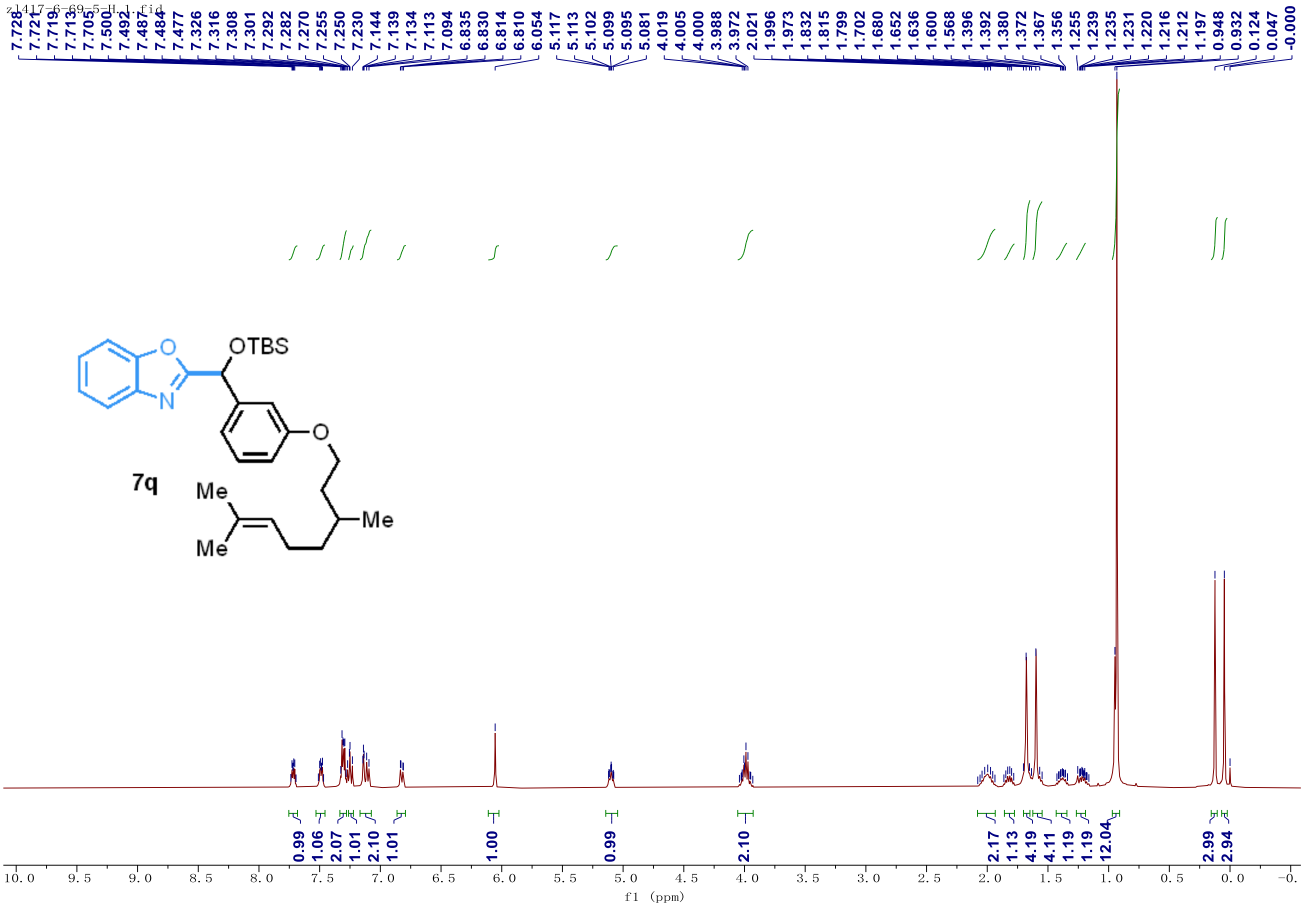
z1417-6-69-5-H.1.fid
7.728
7.721
7.719
7.713
7.705
7.500
7.492
7.487
7.484
7.477
7.326
7.316
7.308
7.301
7.292
7.282
7.270
7.255
7.250
7.230
7.144
7.139
7.134
7.113
7.094
6.835
6.830
6.814
6.810
6.054
5.117
5.113
5.102
5.099
5.095
5.081
4.019
4.005
4.000
3.988
3.972
2.021
1.996
1.973
1.832
1.815
1.799
1.702
1.680
1.652
1.636
1.600
1.568
1.396
1.392
1.380
1.372
1.367
1.356
1.255
1.239
1.235
1.231
1.220
1.216
1.212
1.197
0.948
0.932
0.124
0.047
-0.000
OTBS
O
N
O
Me
Me
Me
7q
0.99
1.06
2.07
1.01
2.10
1.01
1.00
0.99
2.10
2.17
1.13
4.19
4.11
1.19
1.19
12.04
2.99
2.94
10.0 9.5 9.0 8.5 8.0 7.5 7.0 6.5 6.0 5.5 5.0 4.5 4.0 3.5 3.0 2.5 2.0 1.5 1.0 0.5 0.0 -0.
f1 (ppm)

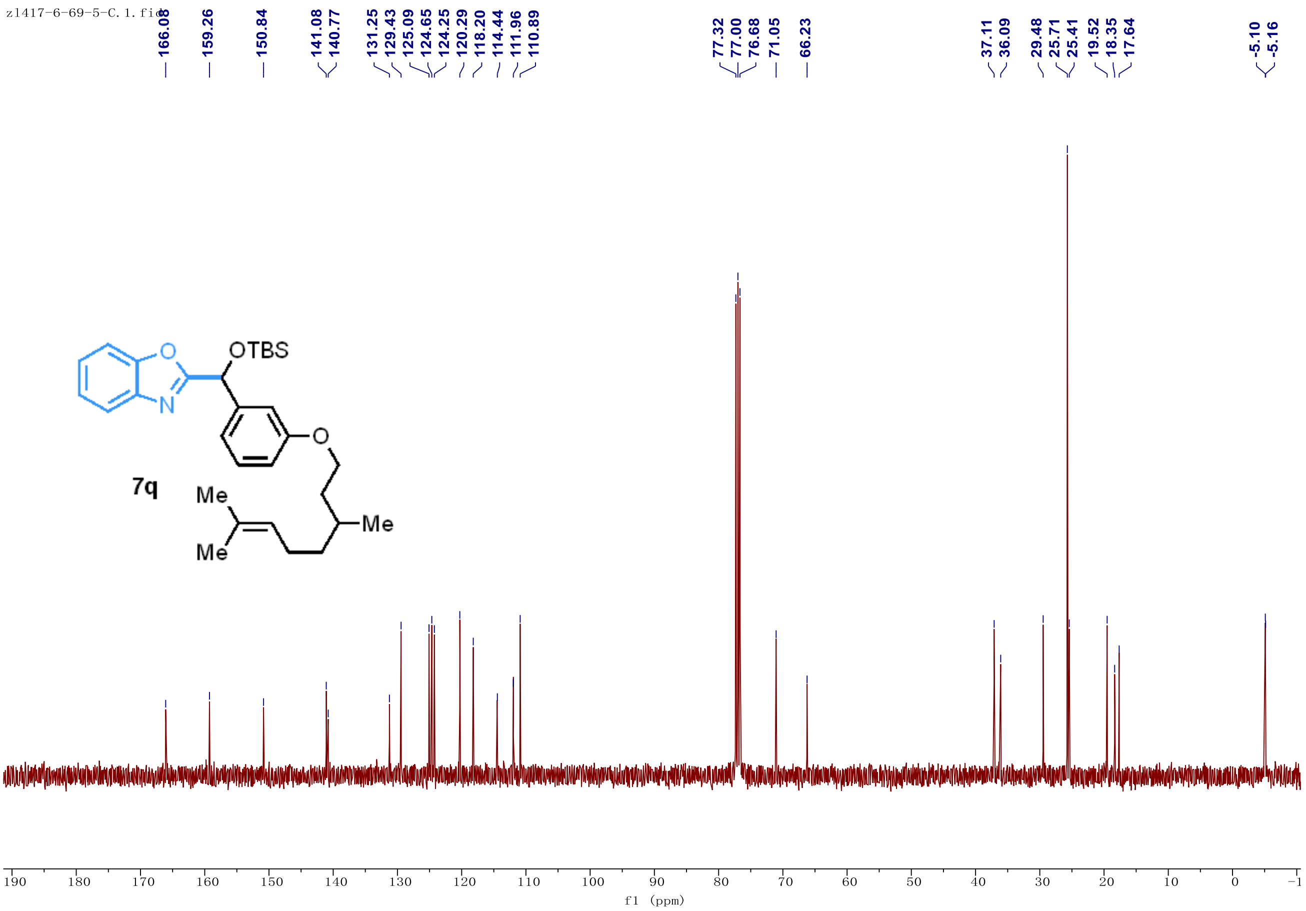
z1417-6-69-5-C.1.fid
166.08
159.26
150.84
141.08
140.77
131.25
129.43
125.09
124.65
124.25
120.29
118.20
114.44
111.96
110.89
77.32
77.00
76.68
71.05
66.23
37.11
36.09
29.48
25.71
25.41
19.52
18.35
17.64
-5.10
-5.16
OTBS
O
N
O
Me
Me
Me
7q
190 180 170 160 150 140 130 120 110 100 90 80 70 60 50 40 30 20 10 0 -1
f1 (ppm)

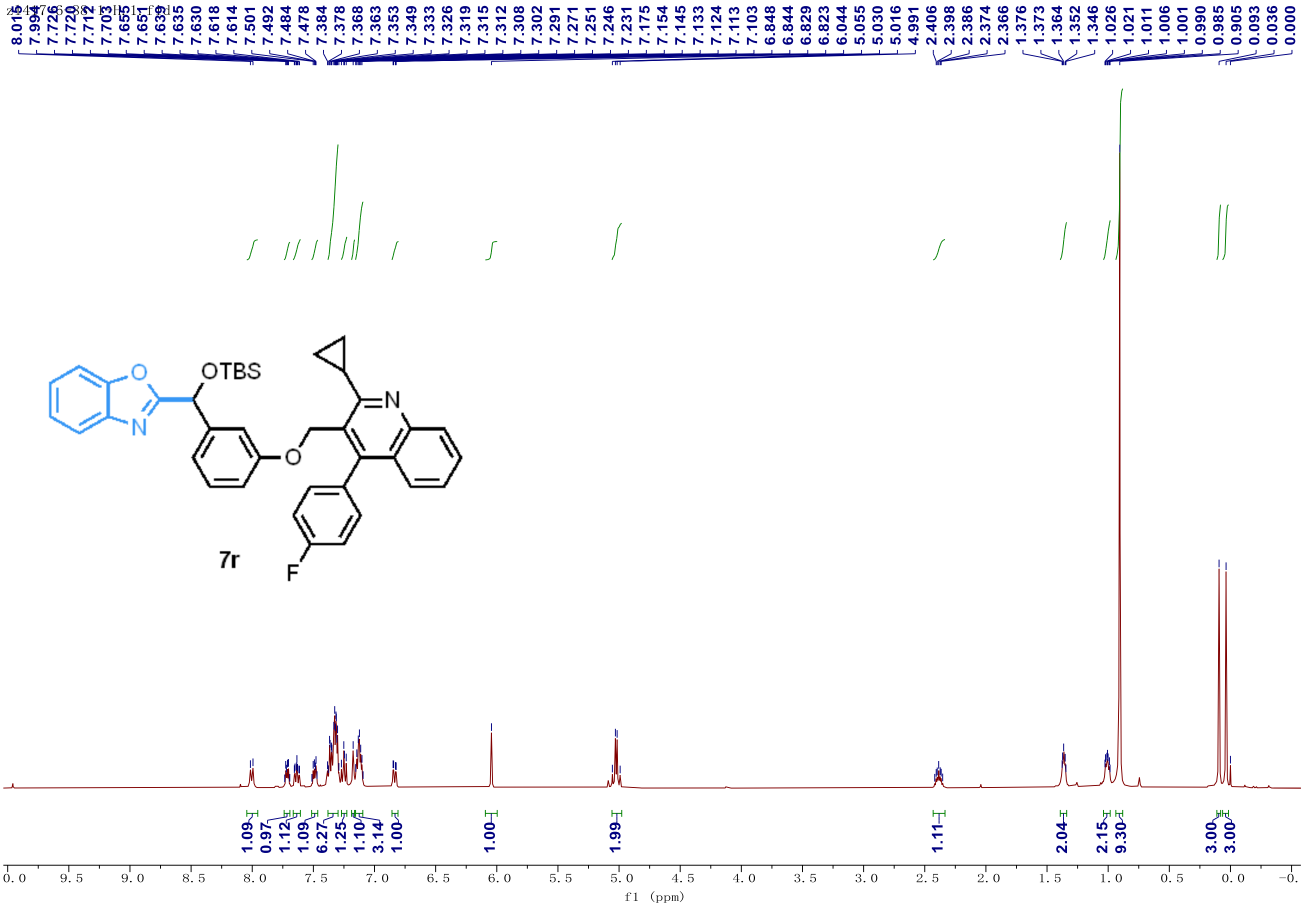
7.994
7.726
7.720
7.712
7.703
7.655
7.651
7.639
7.635
7.630
7.618
7.614
7.501
7.492
7.484
7.478
7.384
7.378
7.368
7.363
7.353
7.349
7.333
7.326
7.319
7.315
7.312
7.308
7.302
7.291
7.271
7.251
7.246
7.231
7.175
7.154
7.145
7.133
7.124
7.113
7.103
6.848
6.844
6.829
6.823
6.044
5.055
5.030
5.016
4.991
2.406
2.398
2.386
2.374
2.366
1.376
1.373
1.364
1.352
1.346
1.026
1.021
1.011
1.006
1.001
0.990
0.985
0.905
0.093
0.036
0.000
OTBS
O
N
O
N
F
7r
1.09
0.97
1.12
1.09
6.27
1.25
1.10
3.14
1.00
1.00
1.99
1.11
2.04
2.15
9.30
3.00
3.00
10.0 9.5 9.0 8.5 8.0 7.5 7.0 6.5 6.0 5.5 5.0 4.5 4.0 3.5 3.0 2.5 2.0 1.5 1.0 0.5 0.0 -0.
f1 (ppm)

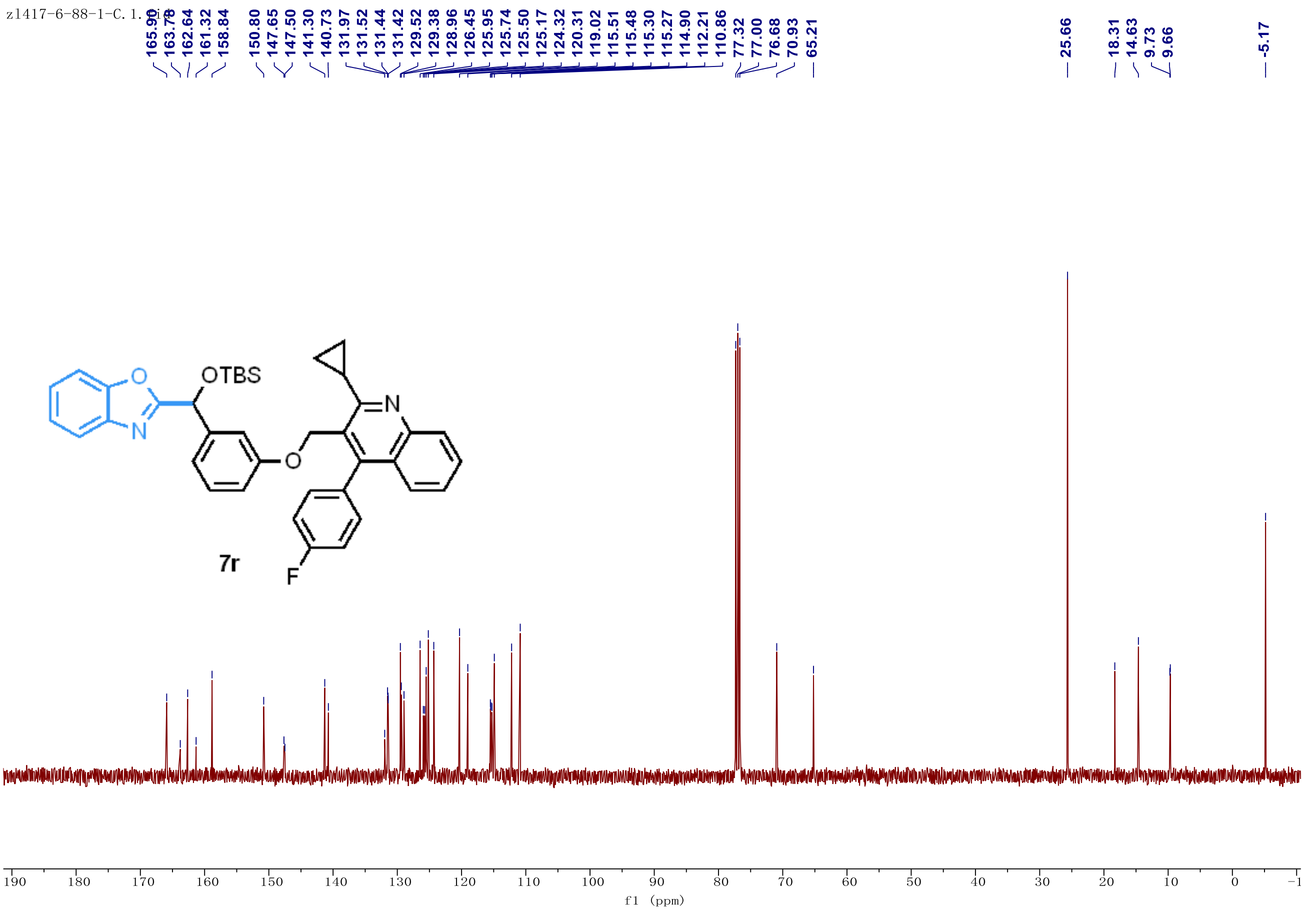
z1417-6-88-1-C.1.fid
165.90
163.78
162.64
161.32
158.84
150.80
147.65
147.50
141.30
140.73
131.97
131.52
131.44
131.42
129.52
129.38
128.96
126.45
125.95
125.74
125.50
125.17
124.32
120.31
119.02
115.51
115.48
115.30
115.27
114.90
112.21
110.86
77.32
77.00
76.68
70.93
65.21
25.66
18.31
14.63
9.73
9.66
-5.17
OTBS
O
N
N
O
F
7r
190 180 170 160 150 140 130 120 110 100 90 80 70 60 50 40 30 20 10 0
f1 (ppm)

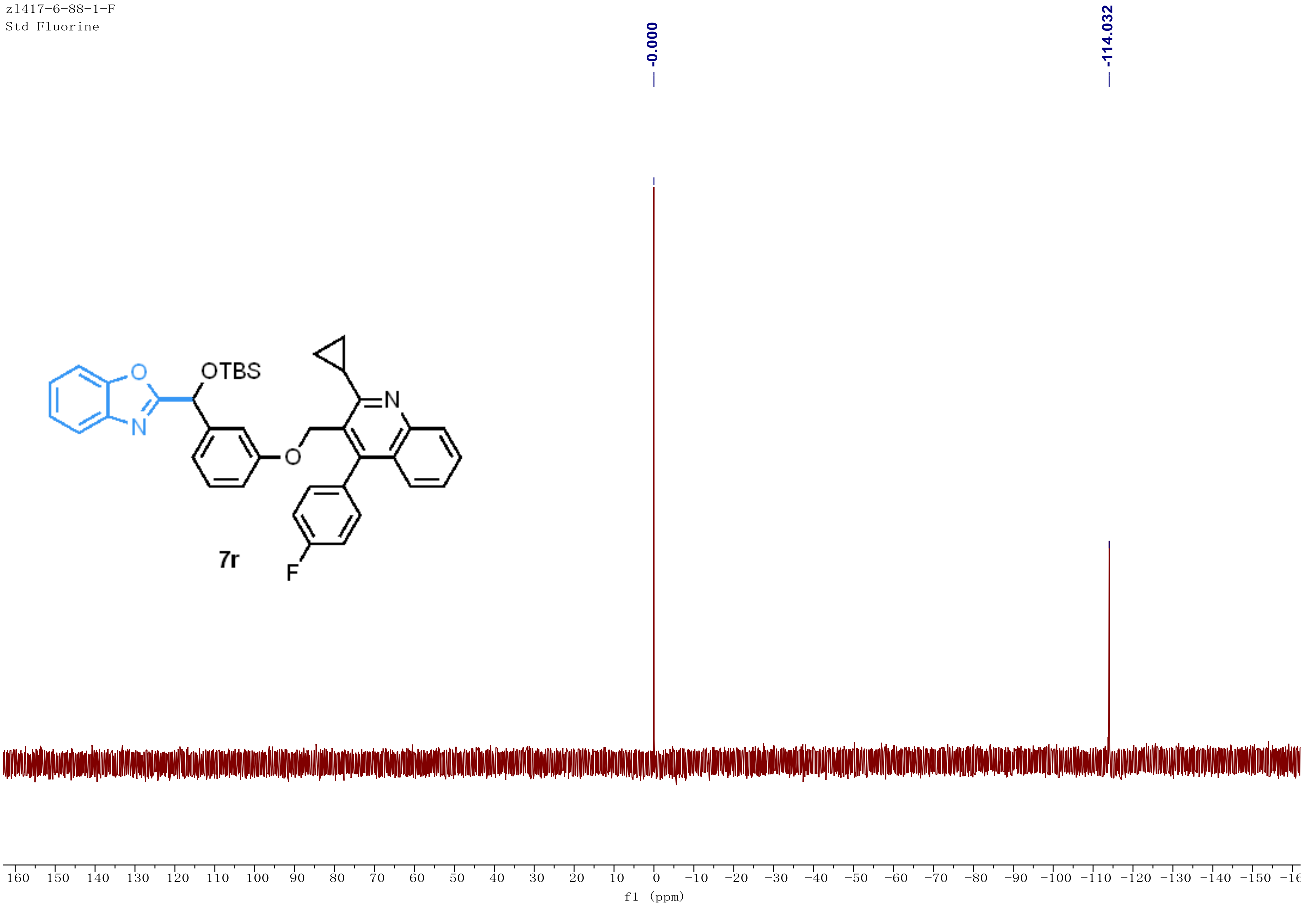
z1417-6-88-1-F
Std Fluorine
-0.000
-114.032
OTBS
O
N
N
O
F
7r
160 150 140 130 120 110 100 90 80 70 60 50 40 30 20 10 0 −10 −20 −30 −40 −50 −60 −70 −80 −90 −100 −110 −120 −130 −140 −150
f1 (ppm)

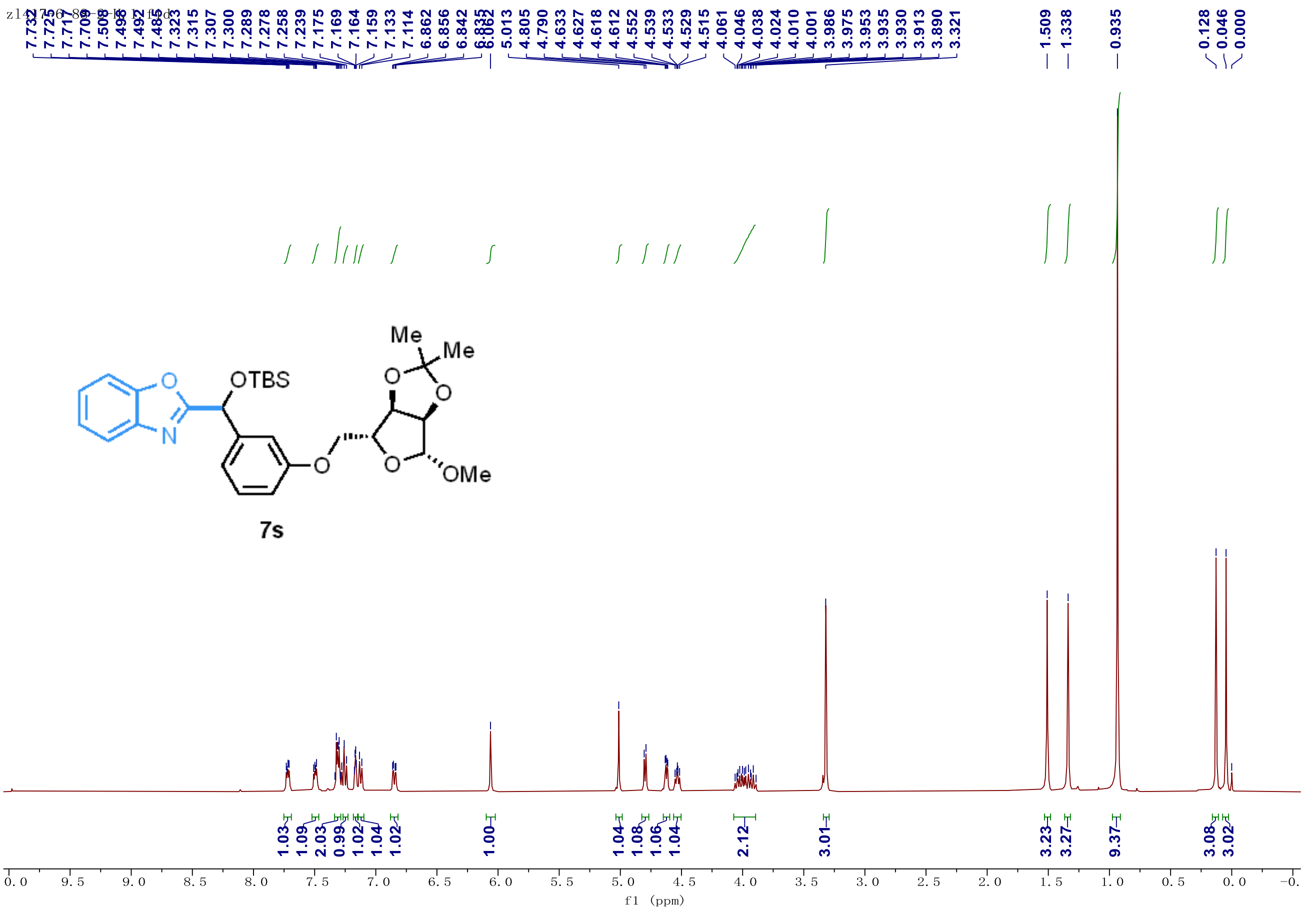
7.732
7.725
7.717
7.709
7.503
7.498
7.492
7.485
7.323
7.315
7.307
7.300
7.289
7.278
7.258
7.239
7.175
7.169
7.164
7.159
7.133
7.114
6.862
6.856
6.842
6.835
6.062
5.013
4.805
4.790
4.633
4.627
4.618
4.612
4.552
4.539
4.533
4.529
4.515
4.061
4.046
4.038
4.024
4.010
4.001
3.986
3.975
3.953
3.935
3.930
3.913
3.890
3.321
1.509
1.338
0.935
0.128
0.046
0.000
Me
Me
OTBS
O
N
O
O
O
O
OMe
7s
1.03
1.09
2.03
0.99
1.02
1.04
1.02
1.00
1.04
1.08
1.06
1.04
2.12
3.01
3.23
3.27
9.37
3.08
3.02
f1 (ppm)

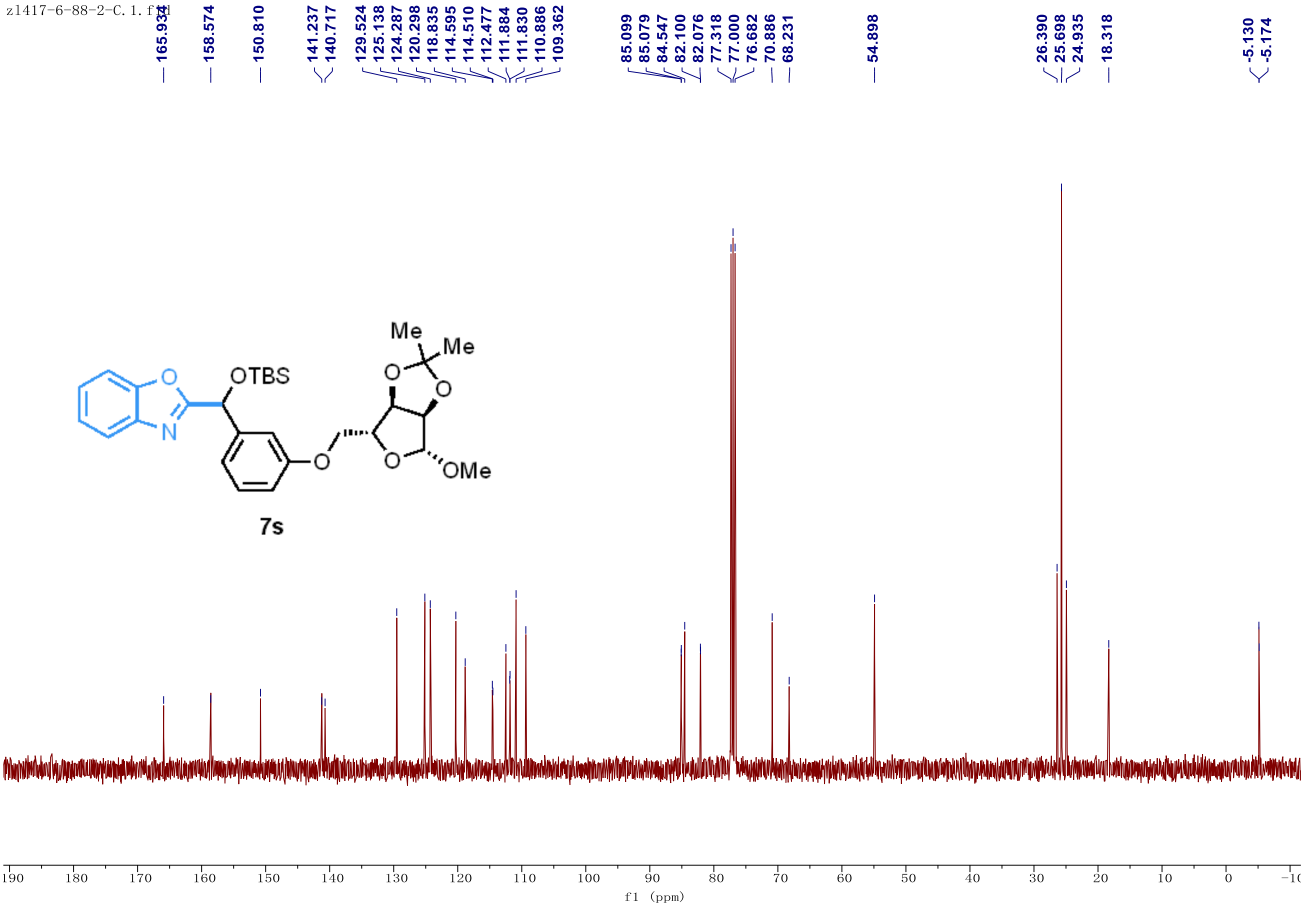
z1417-6-88-2-C.1.fid
165.934
158.574
150.810
141.237
140.717
129.524
125.138
124.287
120.298
118.835
114.595
114.510
112.477
111.884
111.830
110.886
109.362
85.099
85.079
84.547
82.100
82.076
77.318
77.000
76.682
70.886
68.231
54.898
26.390
25.698
24.935
18.318
-5.130
-5.174
Me
Me
OTBS
O
N
O
O
O
O
OMe
7s
f1 (ppm)

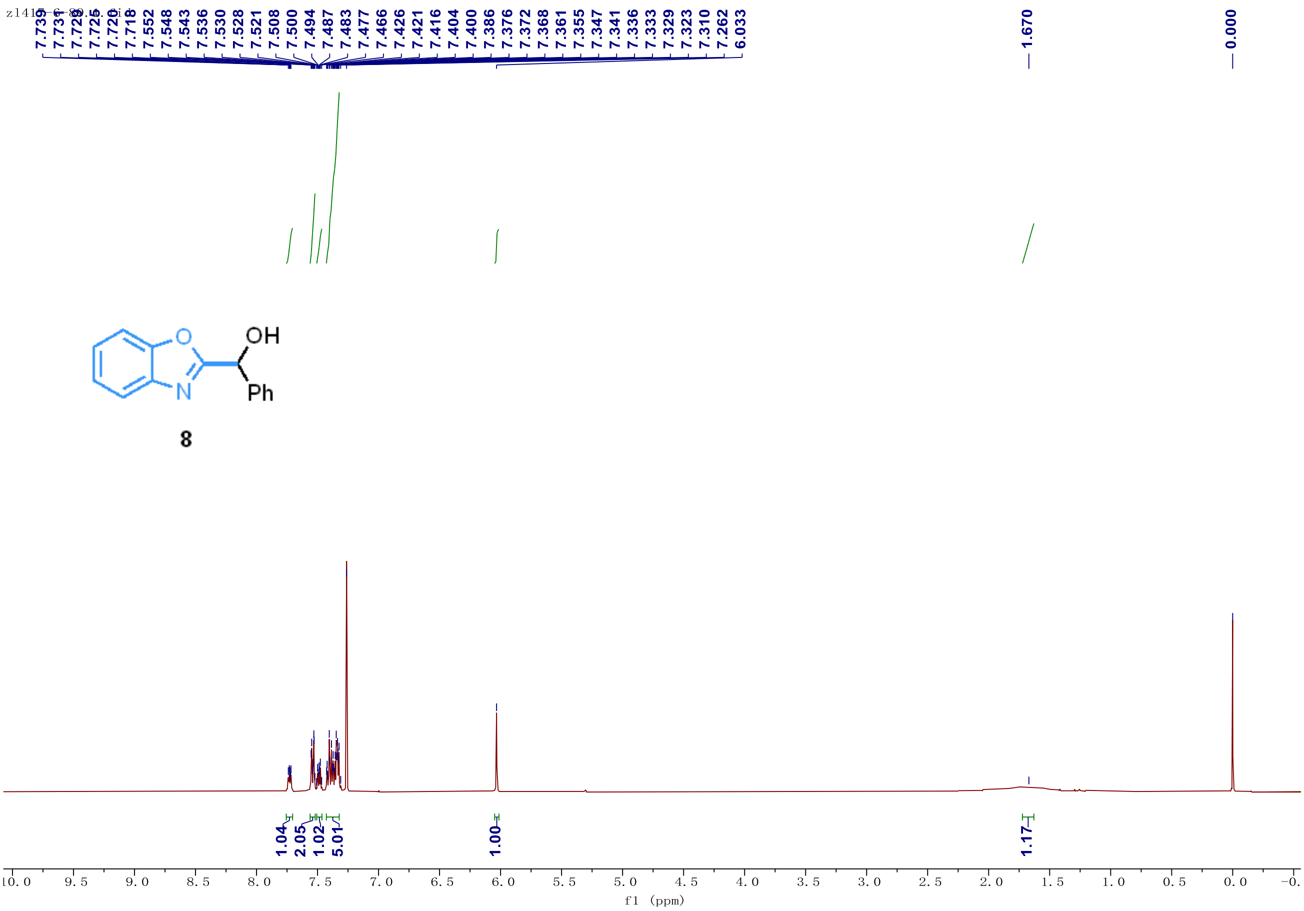
7.739
7.731
7.728
7.725
7.720
7.718
7.552
7.548
7.543
7.536
7.530
7.528
7.521
7.508
7.500
7.494
7.487
7.483
7.477
7.466
7.426
7.421
7.416
7.404
7.400
7.386
7.376
7.372
7.368
7.361
7.355
7.347
7.341
7.336
7.333
7.329
7.323
7.310
7.262
6.033
1.670
0.000
O
N
OH
Ph
8
1.04
2.05
1.02
5.01
1.00
1.17
10.0 9.5 9.0 8.5 8.0 7.5 7.0 6.5 6.0 5.5 5.0 4.5 4.0 3.5 3.0 2.5 2.0 1.5 1.0 0.5 0.0 -0.
f1 (ppm)

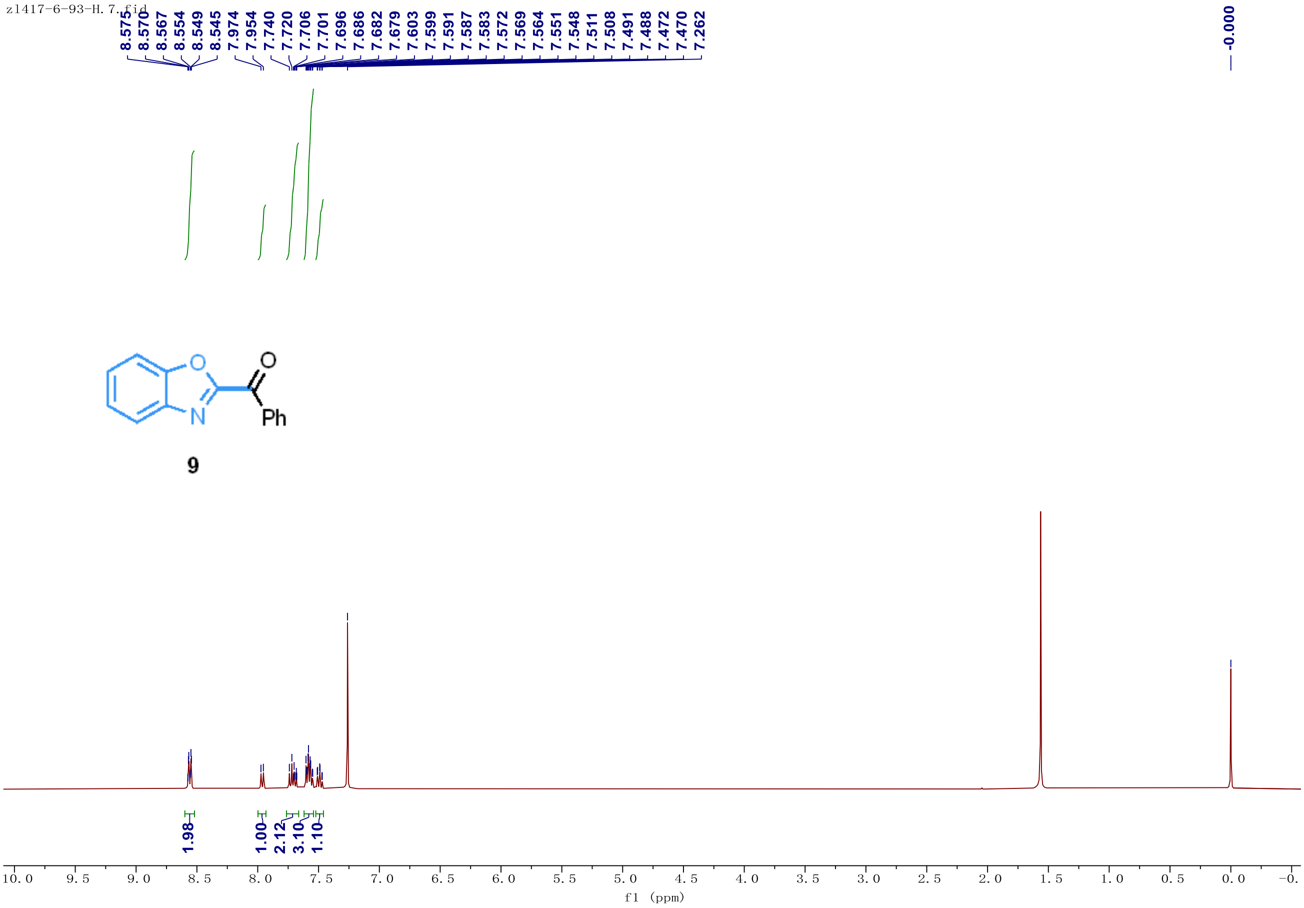
z1417-6-93-H.7.fid
8.575
8.570
8.567
8.554
8.549
8.545
7.974
7.954
7.740
7.720
7.706
7.701
7.696
7.686
7.682
7.679
7.603
7.599
7.591
7.587
7.583
7.572
7.569
7.564
7.551
7.548
7.511
7.508
7.491
7.488
7.472
7.470
7.262
-0.000
O
N
O
Ph
9
1.98
1.00
2.12
3.10
1.10
10.0 9.5 9.0 8.5 8.0 7.5 7.0 6.5 6.0 5.5 5.0 4.5 4.0 3.5 3.0 2.5 2.0 1.5 1.0 0.5 0.0 -0.
f1 (ppm)

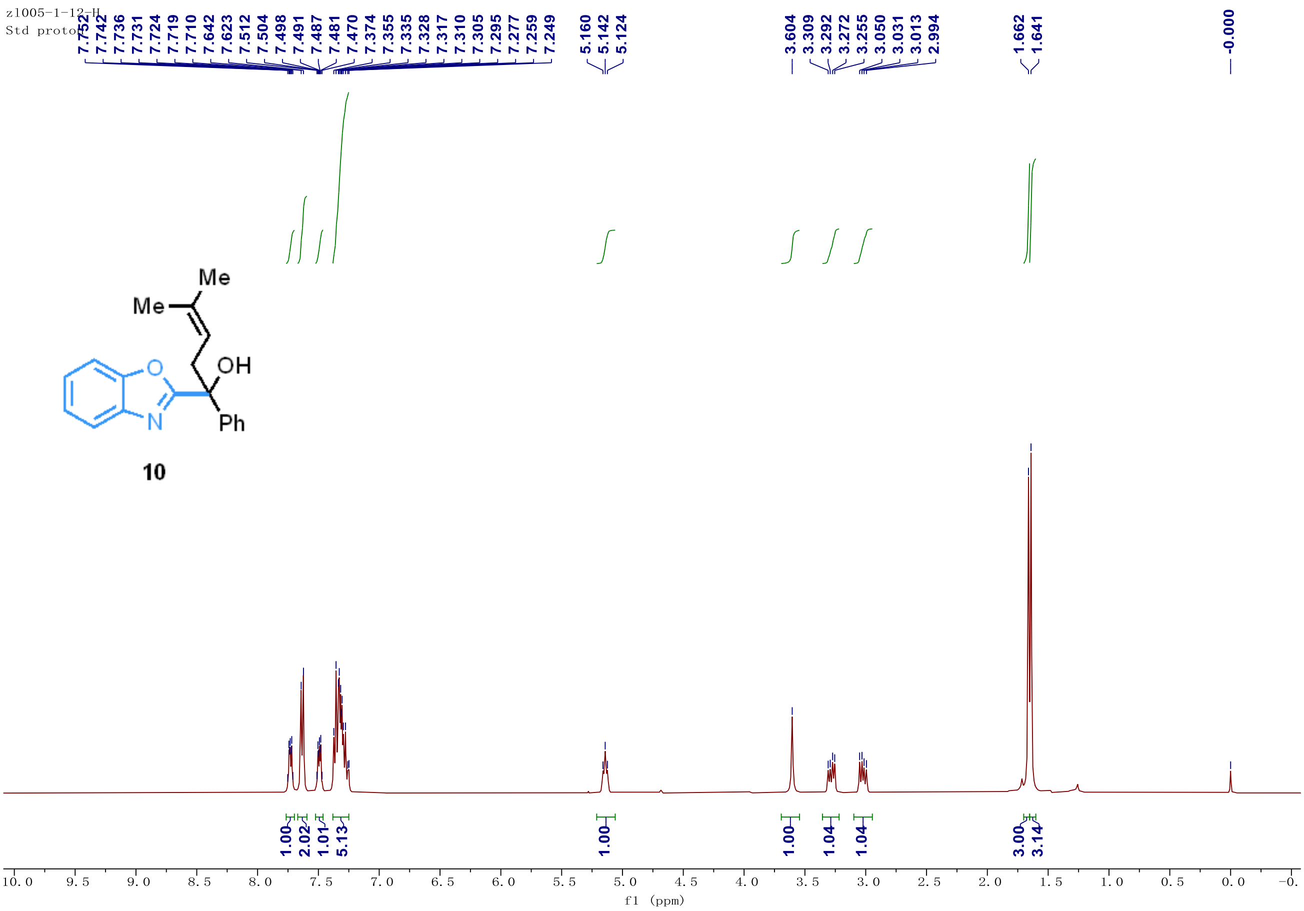
z1005-1-12-H
Std proton
7.752 7.742 7.736 7.731 7.724 7.719 7.710 7.642 7.623 7.512 7.504 7.498 7.491 7.487 7.481 7.470 7.374 7.355 7.335 7.328 7.317 7.310 7.305 7.295 7.277 7.259 7.249
5.160 5.142 5.124
3.604 3.309 3.292 3.272 3.255 3.050 3.031 3.013 2.994
1.662 1.641
-0.000
Me
Me
OH
O
N
Ph
1.00 2.02 1.01 5.13 1.00 1.00 1.04 1.04 3.00 3.14
f1 (ppm)

10

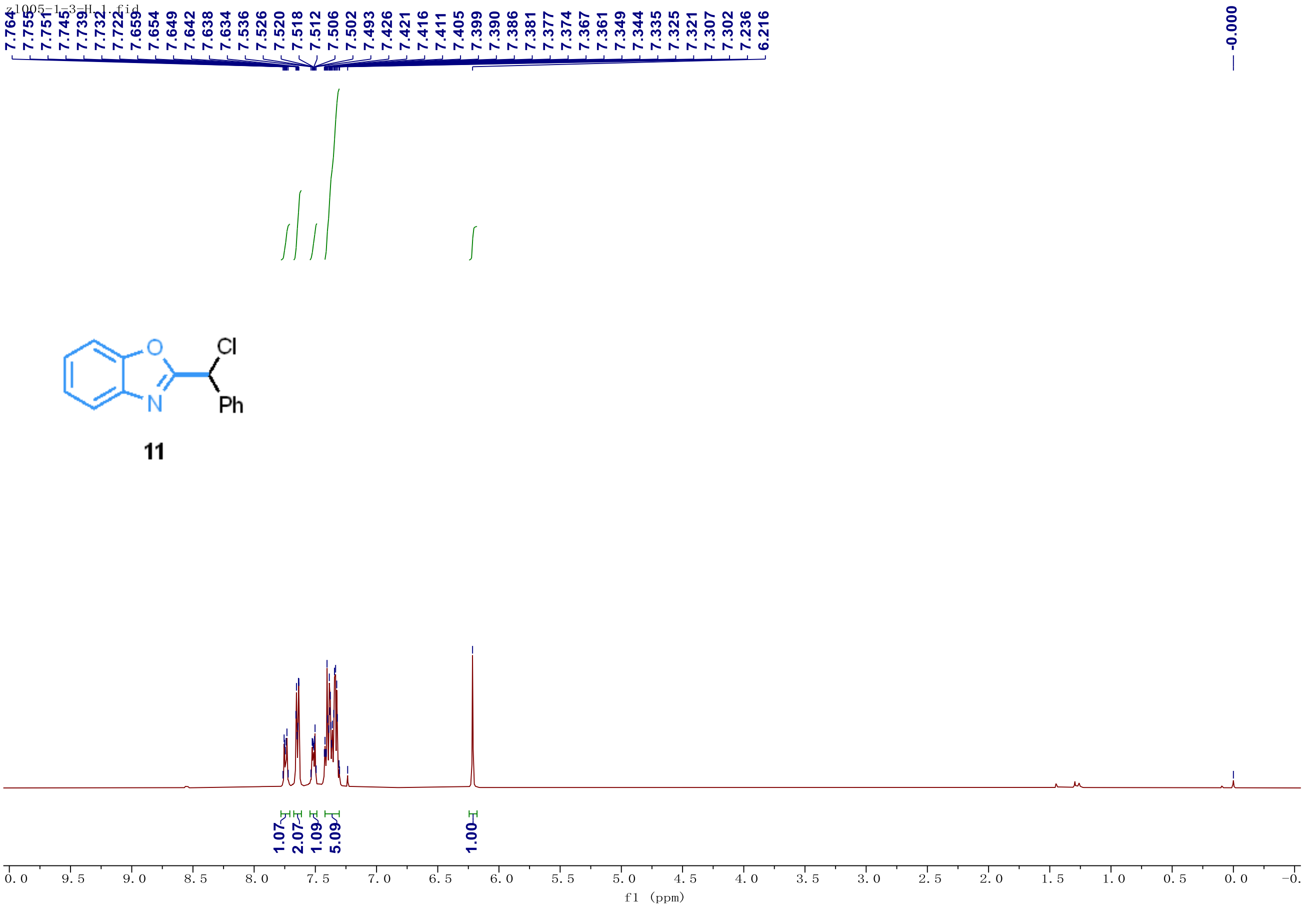
z1005-1-3-H.1.fid
7.764 7.755 7.751 7.745 7.739 7.732 7.722 7.659 7.654 7.649 7.642 7.638 7.634 7.536 7.526 7.520 7.518 7.512 7.506 7.502 7.493 7.426 7.421 7.416 7.411 7.405 7.399 7.390 7.386 7.381 7.377 7.374 7.367 7.361 7.349 7.344 7.335 7.325 7.321 7.307 7.302 7.236 6.216
-0.000
Cl
O
N
Ph
1.07 2.07 1.09 5.09 1.00
f1 (ppm)

11

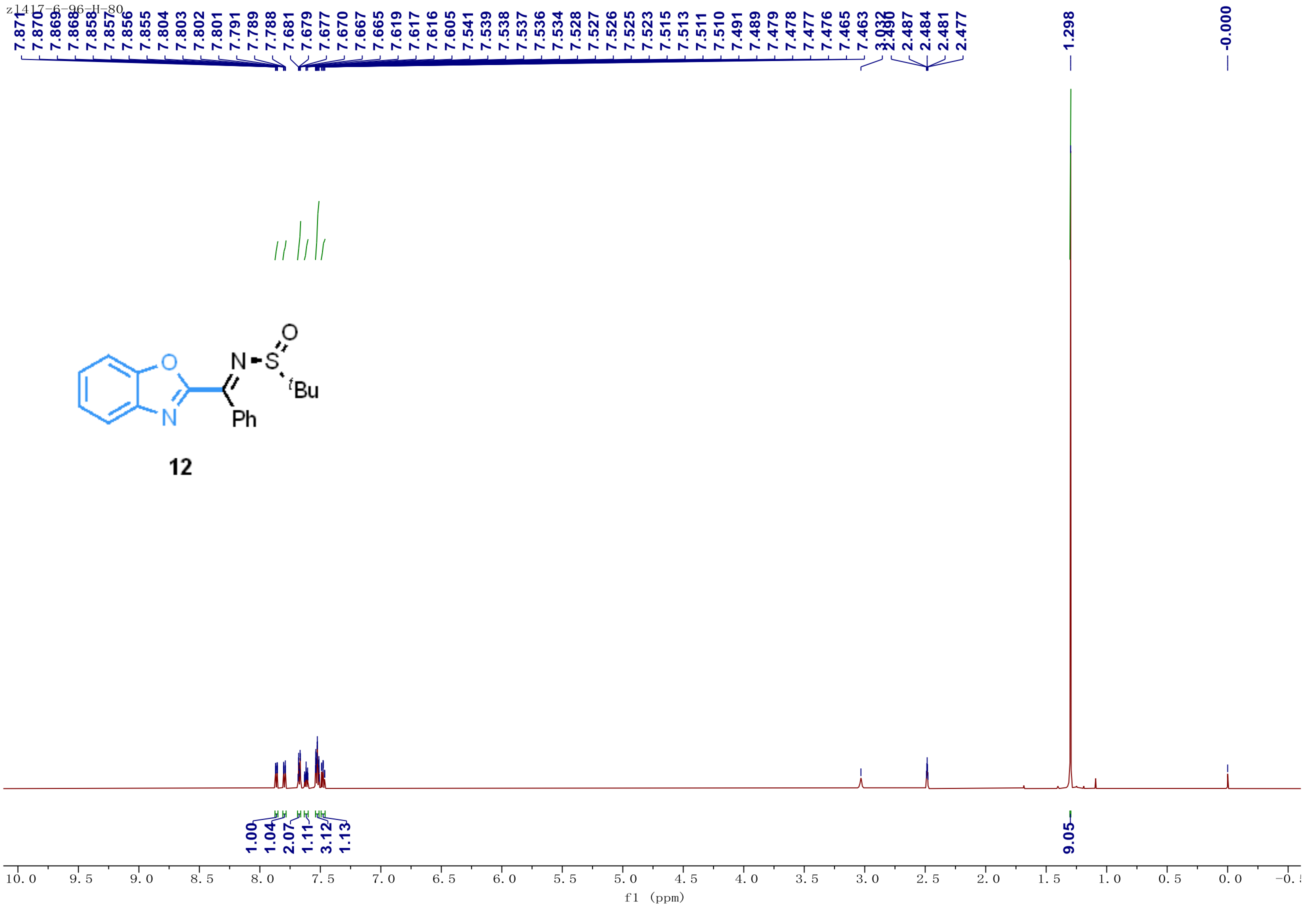
z1417-6-96-H-80
7.871
7.870
7.869
7.868
7.858
7.857
7.856
7.855
7.804
7.803
7.802
7.801
7.791
7.789
7.788
7.681
7.679
7.677
7.670
7.667
7.665
7.619
7.617
7.616
7.605
7.541
7.539
7.538
7.537
7.536
7.534
7.528
7.527
7.526
7.525
7.523
7.515
7.513
7.511
7.510
7.491
7.489
7.479
7.478
7.477
7.476
7.465
7.463
3.032
2.490
2.487
2.484
2.481
2.477
1.298
-0.000
N
S
O
tBu
O
N
Ph
12
1.00
1.04
2.07
1.11
3.12
1.13
9.05
f1 (ppm)

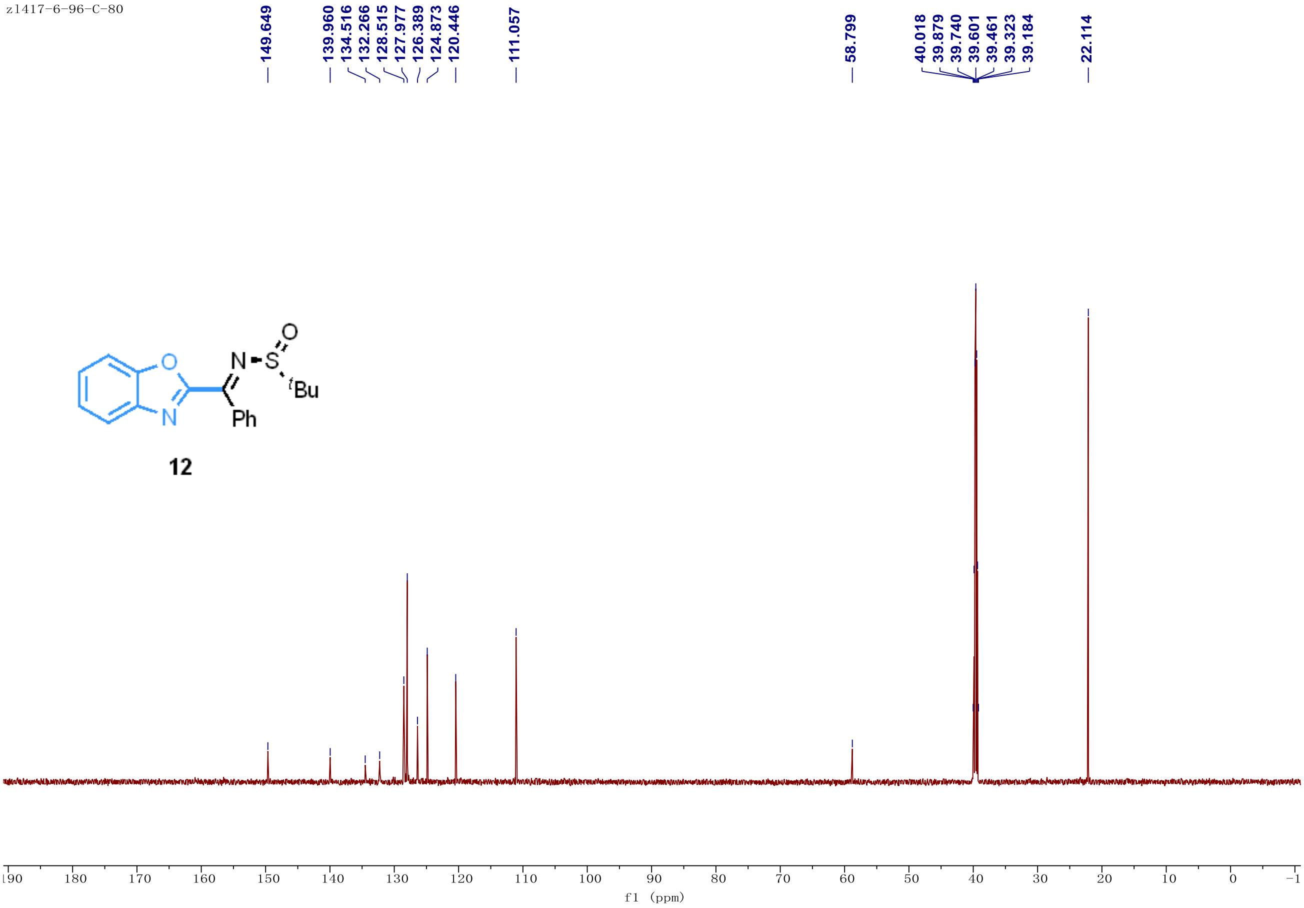
z1417-6-96-C-80
149.649
139.960
134.516
132.266
128.515
127.977
126.389
124.873
120.446
111.057
58.799
40.018
39.879
39.740
39.601
39.461
39.323
39.184
22.114
N
S
O
tBu
O
N
Ph
12
f1 (ppm)

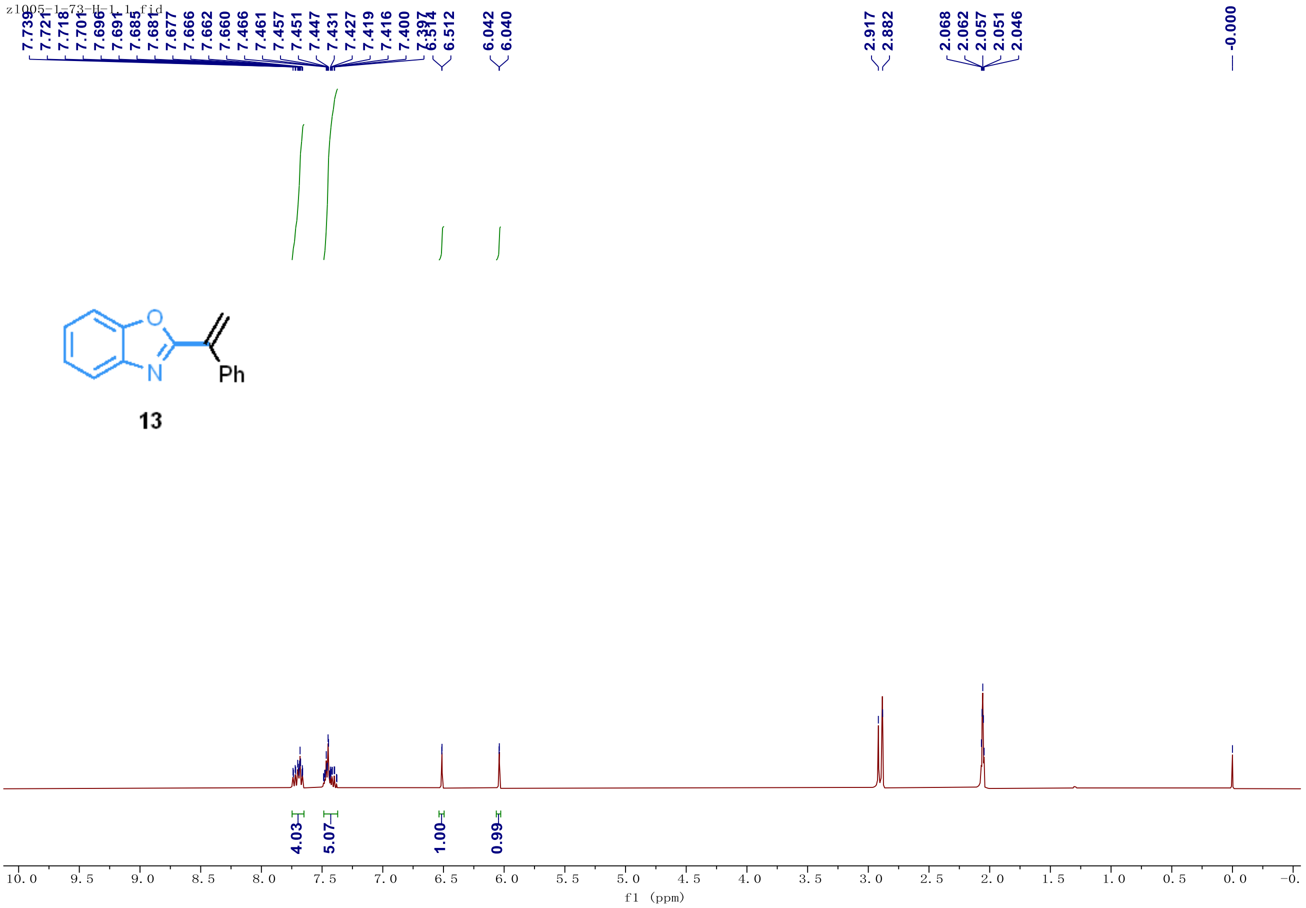

z1005-1-73-H-1.1.fid
7.739 7.721 7.718 7.701 7.696 7.691 7.685 7.681 7.677 7.666 7.662 7.660 7.466 7.461 7.457 7.451 7.447 7.431 7.427 7.419 7.416 7.400 7.397 6.514 6.512
6.042 6.040
2.917 2.882
2.068 2.062 2.057 2.051 2.046
-0.000
O
N
Ph
13
4.03 5.07 1.00 0.99
10.0 9.5 9.0 8.5 8.0 7.5 7.0 6.5 6.0 5.5 5.0 4.5 4.0 3.5 3.0 2.5 2.0 1.5 1.0 0.5 0.0 -0.
f1 (ppm)

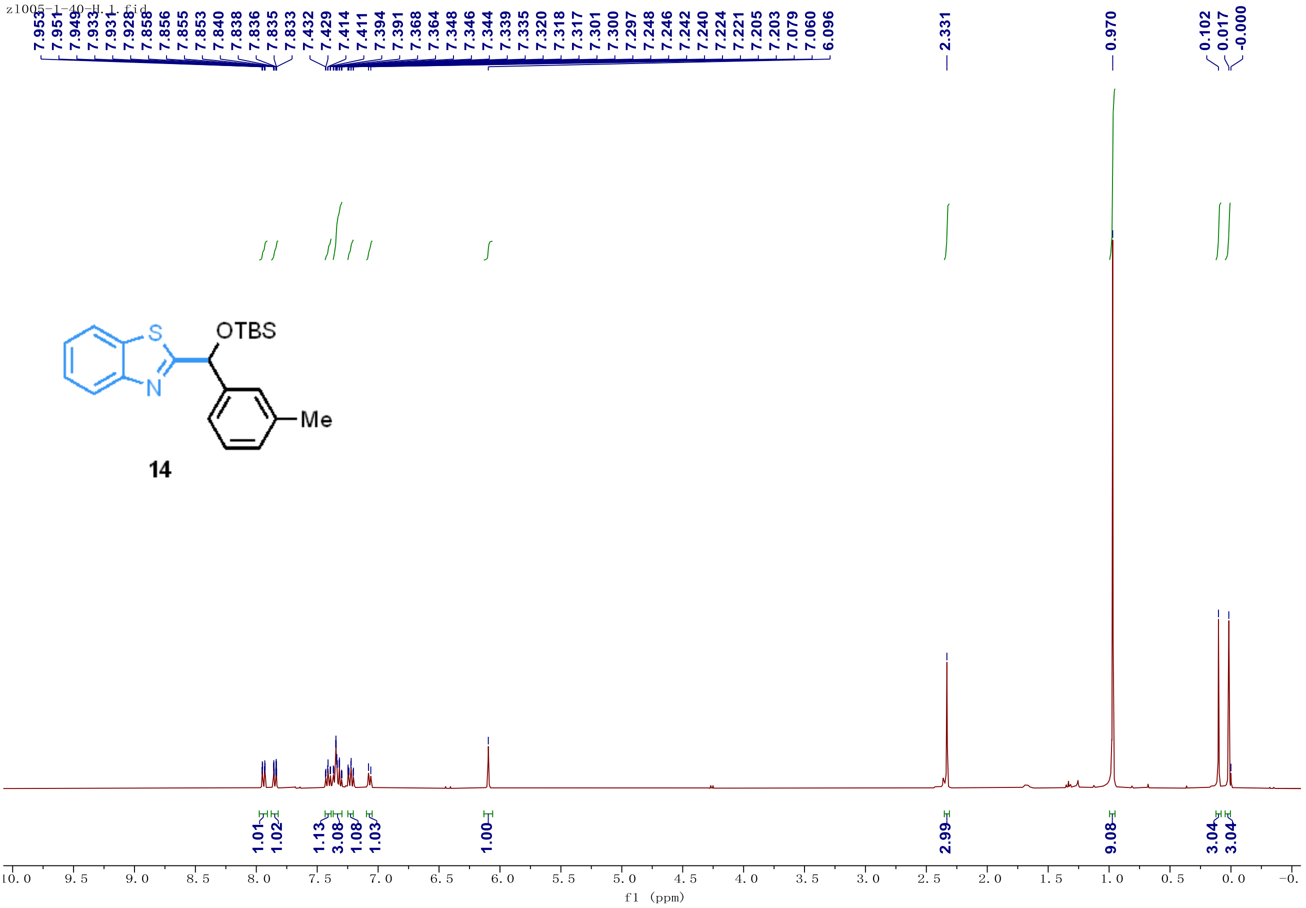

z1005-1-40-H.1.fid
7.953 7.951 7.949 7.933 7.931 7.928 7.858 7.856 7.855 7.853 7.840 7.838 7.836 7.835 7.833 7.432 7.429 7.414 7.411 7.394 7.391 7.368 7.364 7.348 7.346 7.344 7.339 7.335 7.320 7.318 7.317 7.301 7.300 7.297 7.248 7.246 7.242 7.240 7.224 7.221 7.205 7.203 7.079 7.060 6.096
2.331
0.970
0.102 0.017 -0.000
S
N
OTBS
Me
14
1.01 1.02 1.13 3.08 1.08 1.03 1.00 2.99 9.08 3.04 3.04
10.0 9.5 9.0 8.5 8.0 7.5 7.0 6.5 6.0 5.5 5.0 4.5 4.0 3.5 3.0 2.5 2.0 1.5 1.0 0.5 0.0 -0.
f1 (ppm)

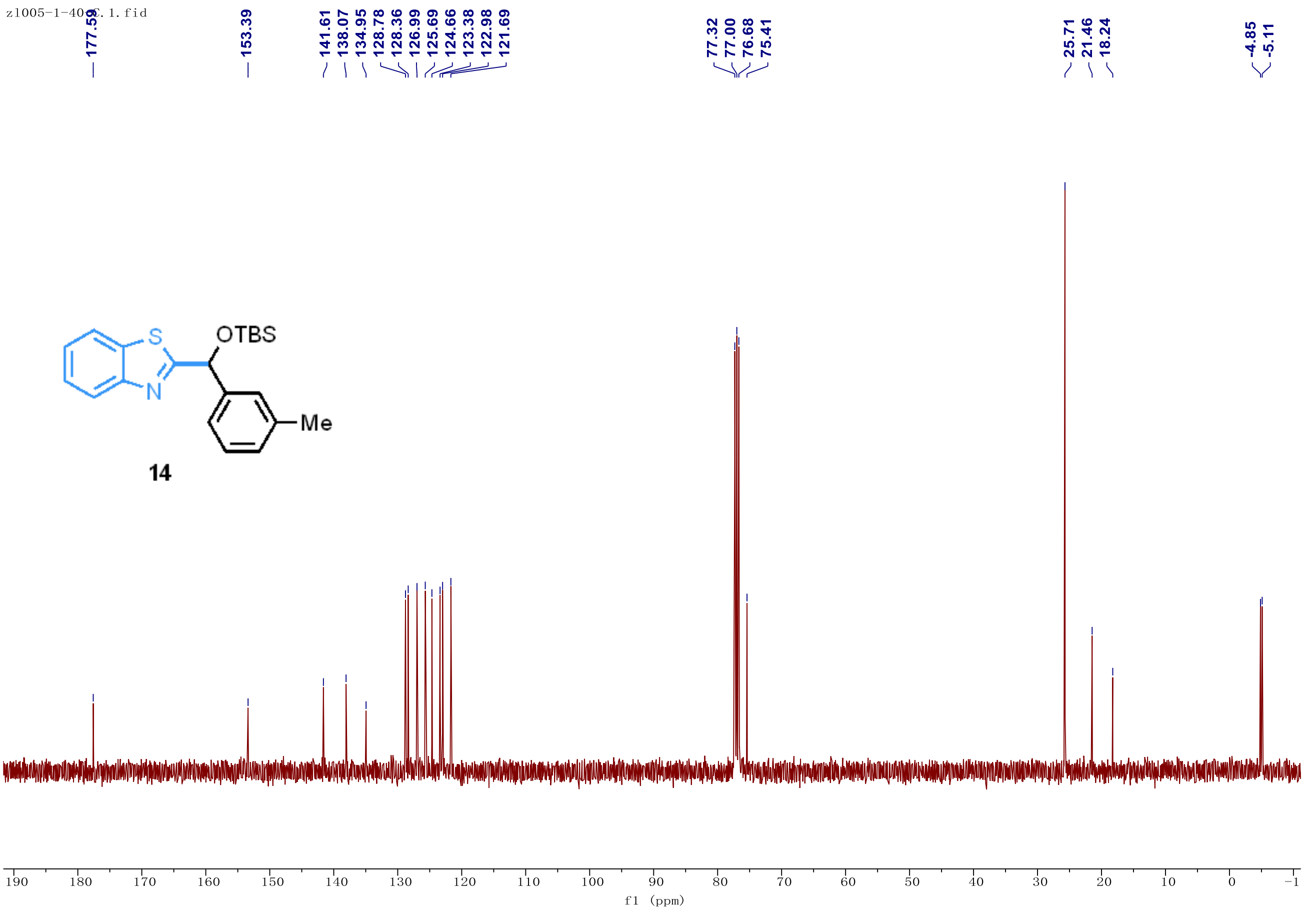

z1005-1-40-C.1.fid
177.59
153.39
141.61
138.07
134.95
128.78
128.36
126.99
125.69
124.66
123.38
122.98
121.69
77.32
77.00
76.68
75.41
25.71
21.46
18.24
-4.85
-5.11
OTBS
S
N
Me
14
190 180 170 160 150 140 130 120 110 100 90 80 70 60 50 40 30 20 10 0 -1
f1 (ppm)

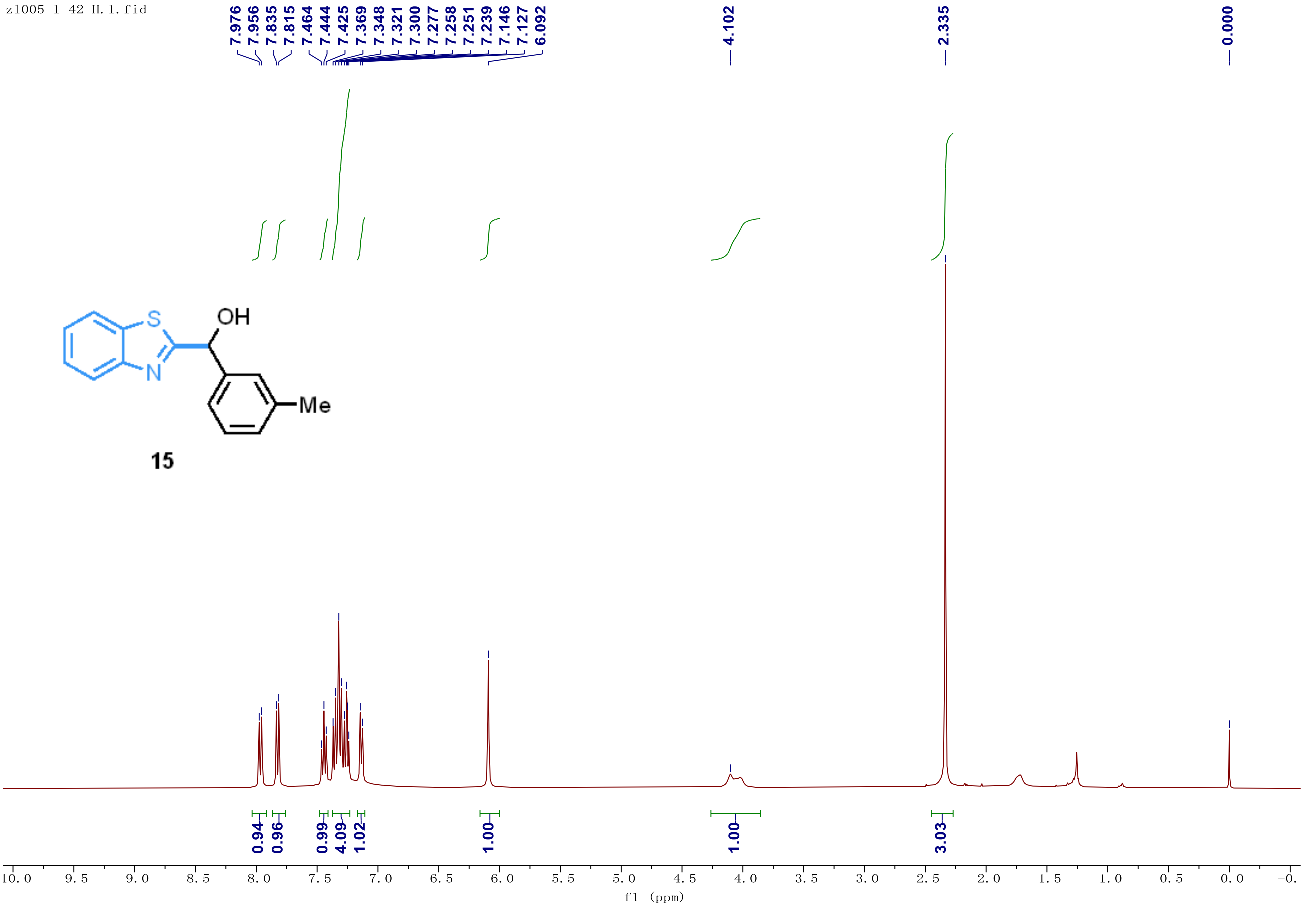

z1005-1-42-H.1.fid
7.976
7.956
7.835
7.815
7.464
7.444
7.425
7.369
7.348
7.321
7.300
7.277
7.258
7.251
7.239
7.146
7.127
6.092
4.102
2.335
0.000
OH
S
N
Me
15
0.94
0.96
0.99
4.09
1.02
1.00
1.00
3.03
10.0 9.5 9.0 8.5 8.0 7.5 7.0 6.5 6.0 5.5 5.0 4.5 4.0 3.5 3.0 2.5 2.0 1.5 1.0 0.5 0.0 -0.
f1 (ppm)

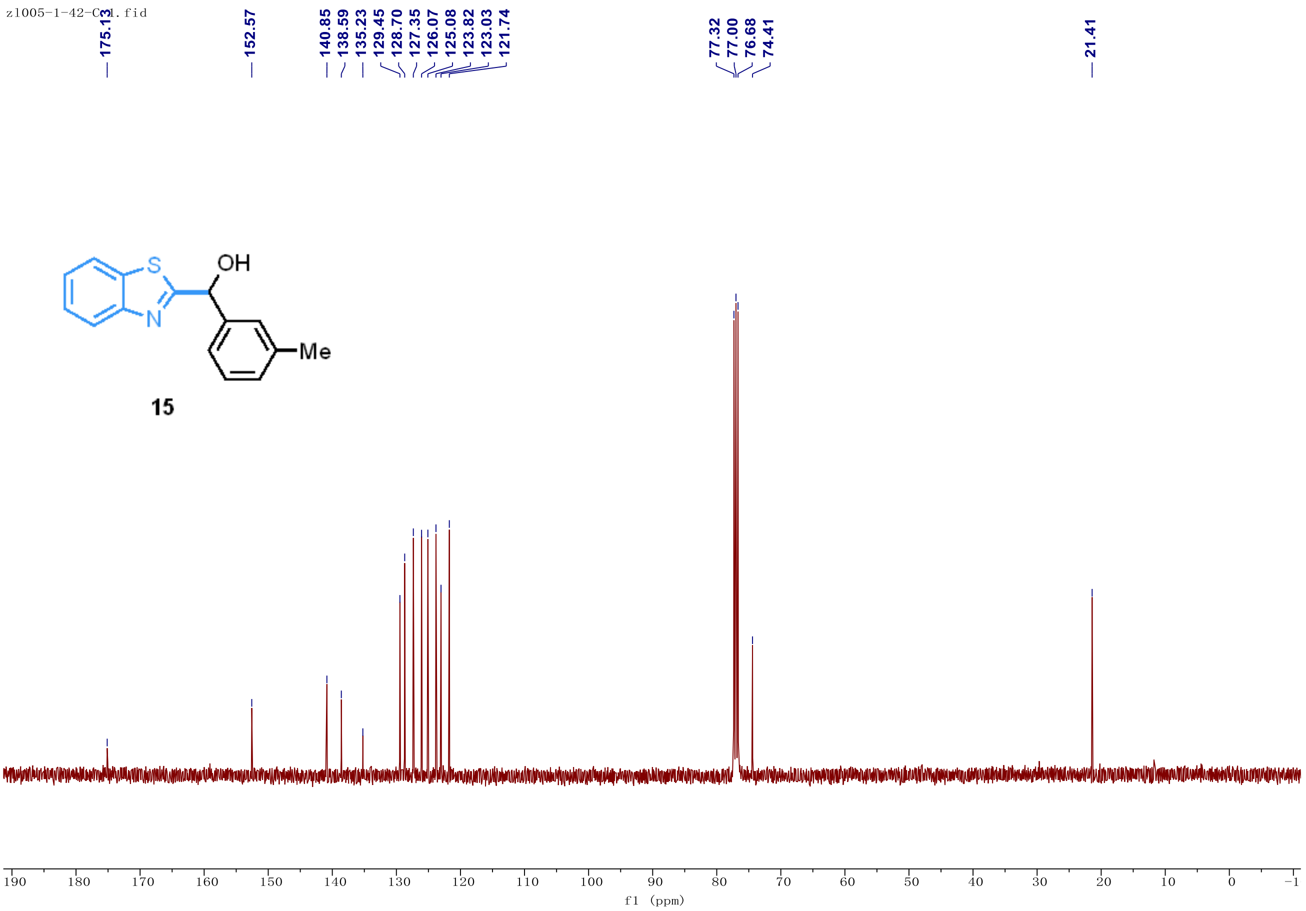
z1005-1-42-C13.fid
175.13
152.57
140.85
138.59
135.23
129.45
128.70
127.35
126.07
125.08
123.82
123.03
121.74
77.32
77.00
76.68
74.41
21.41
OH
S
N
Me
15
f1 (ppm)

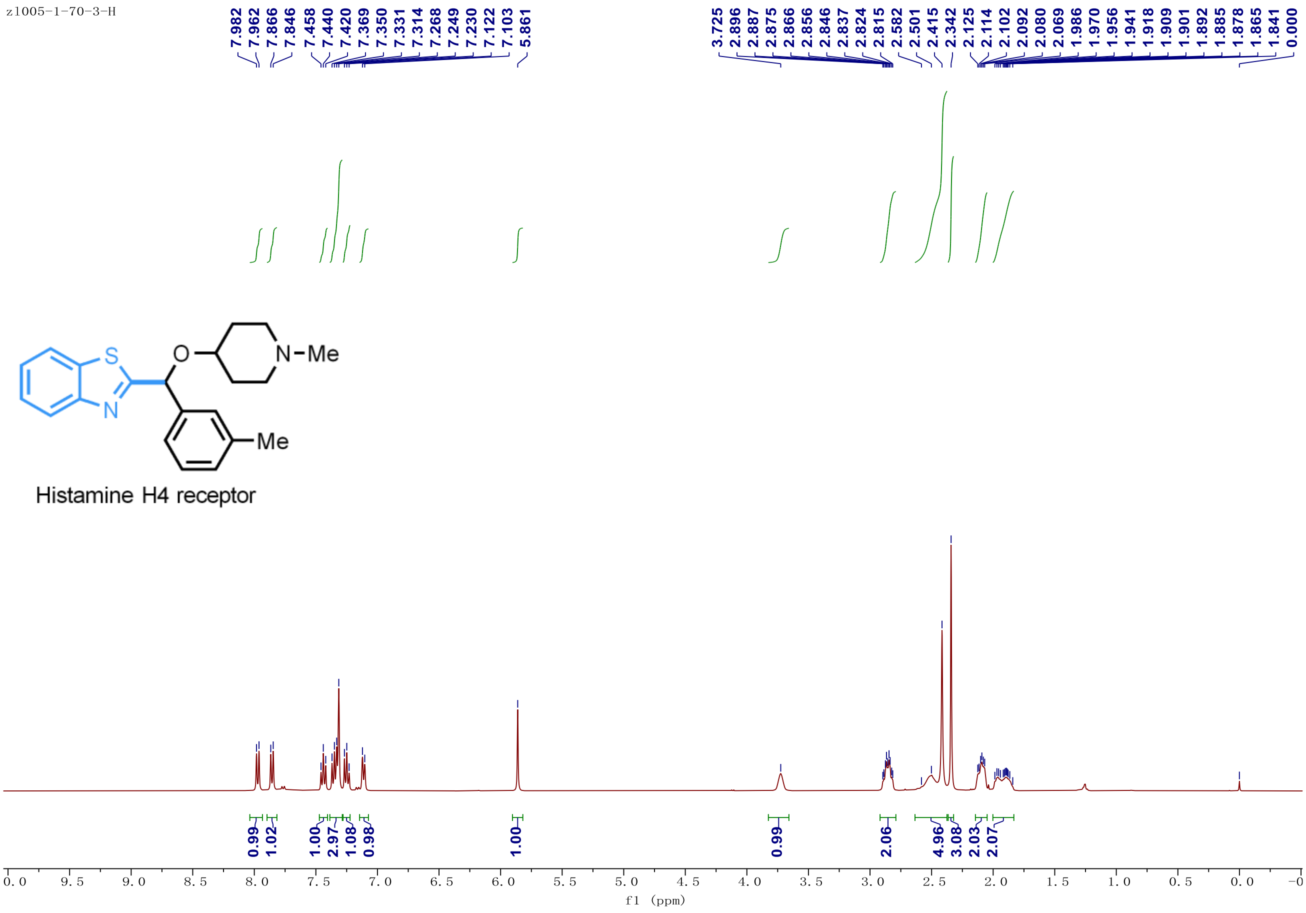
z1005-1-70-3-H
7.982
7.962
7.866
7.846
7.458
7.440
7.420
7.369
7.350
7.331
7.314
7.268
7.249
7.230
7.122
7.103
5.861
3.725
2.896
2.887
2.875
2.866
2.856
2.846
2.837
2.824
2.815
2.582
2.501
2.415
2.342
2.125
2.114
2.102
2.092
2.080
2.069
1.986
1.970
1.956
1.941
1.918
1.909
1.901
1.892
1.885
1.878
1.865
1.841
0.000
S
N
O
N-Me
Me
Histamine H4 receptor
0.99
1.02
1.00
2.97
1.08
0.98
1.00
0.99
2.06
4.96
3.08
2.03
2.07
f1 (ppm)

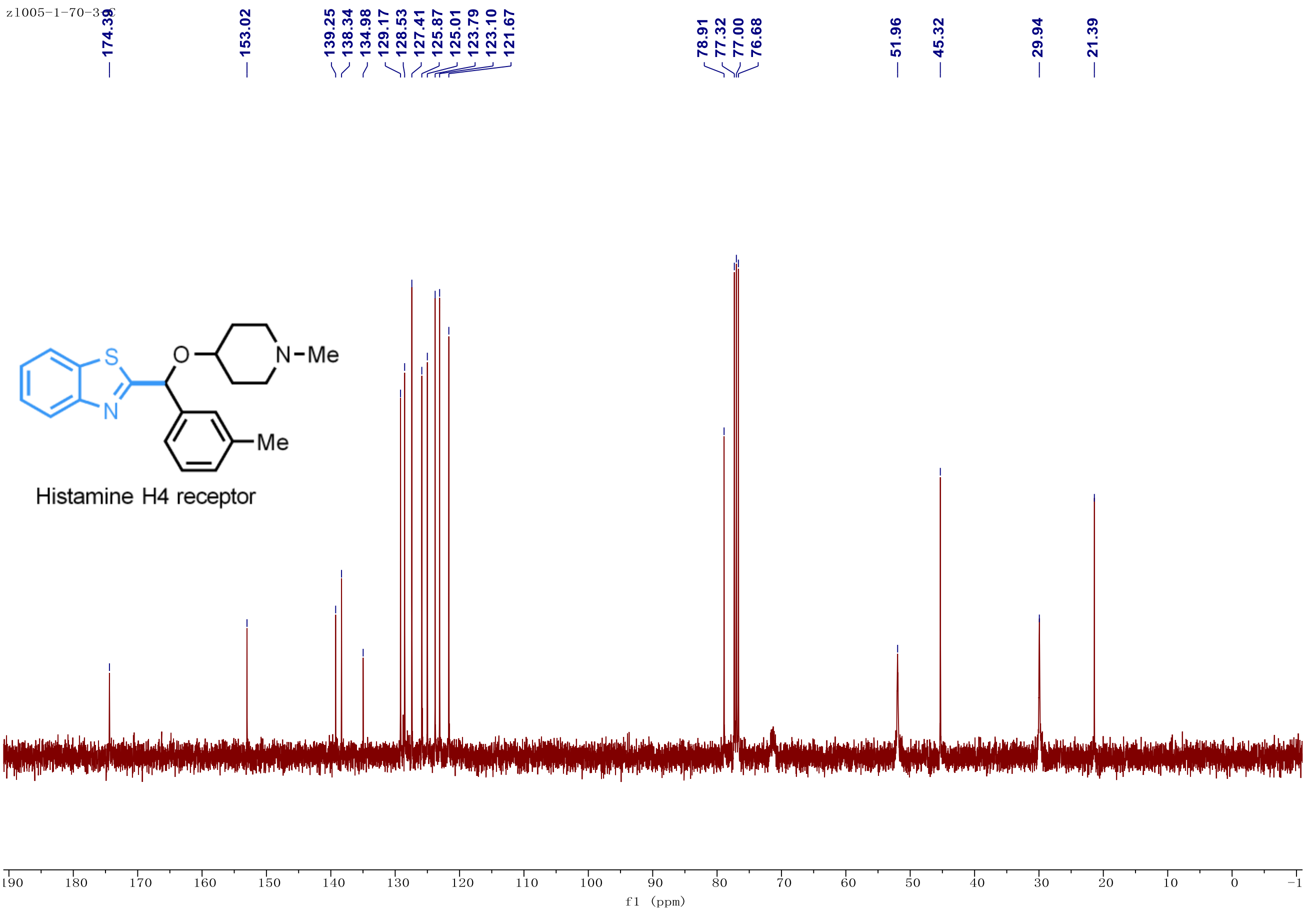
174.39
153.02
139.25
138.34
134.98
129.17
128.53
127.41
125.87
125.01
123.79
123.10
121.67
78.91
77.32
77.00
76.68
51.96
45.32
29.94
21.39
S
N
O
N-Me
Me
Histamine H4 receptor
f1 (ppm)

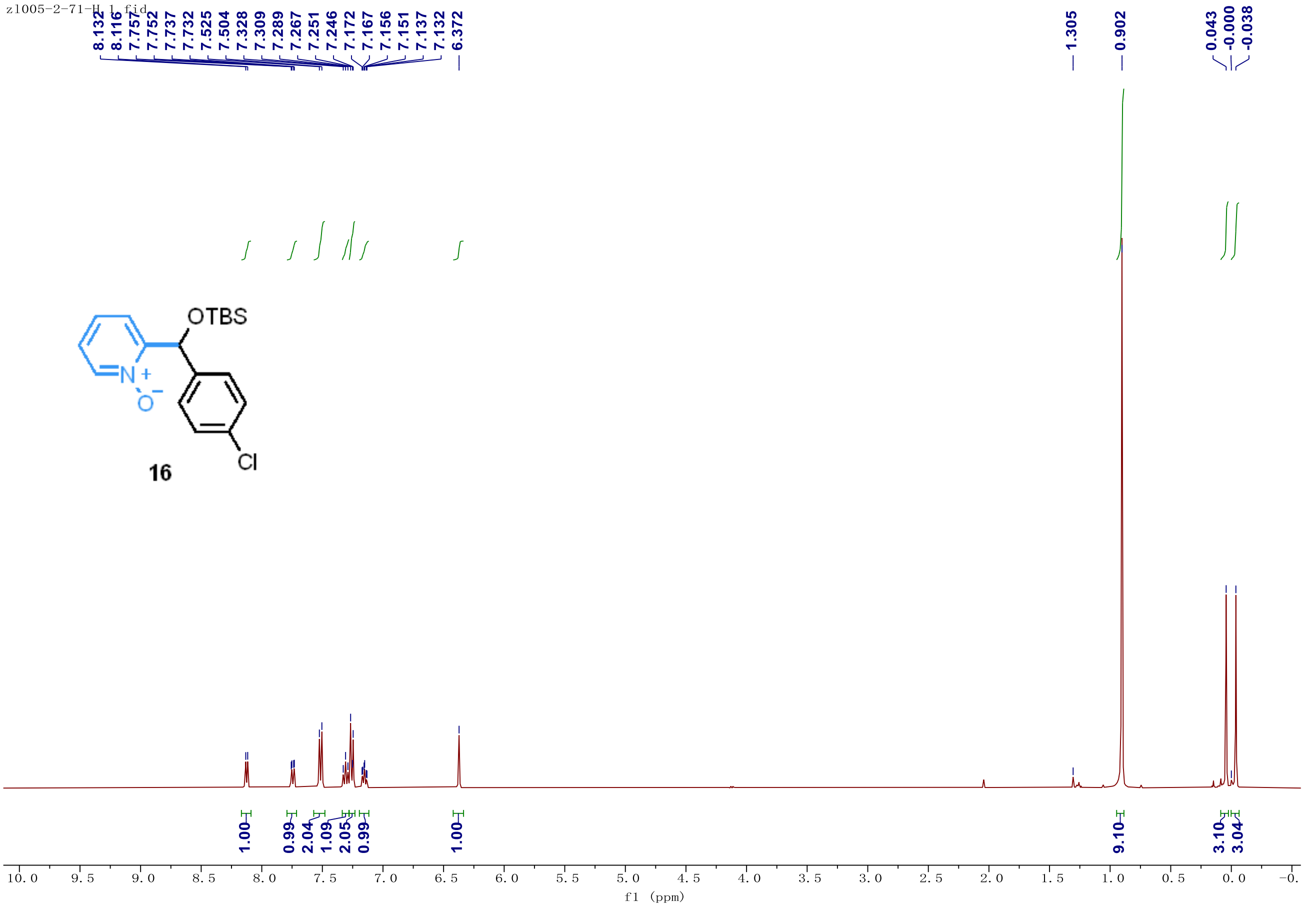
8.132
8.116
7.757
7.752
7.737
7.732
7.525
7.504
7.328
7.309
7.289
7.267
7.251
7.246
7.172
7.167
7.156
7.151
7.137
7.132
6.372
1.305
0.902
0.043
-0.000
-0.038
OTBS
N+
O-
Cl
16
1.00
0.99
2.04
1.09
2.05
0.99
1.00
9.10
3.10
3.04
f1 (ppm)

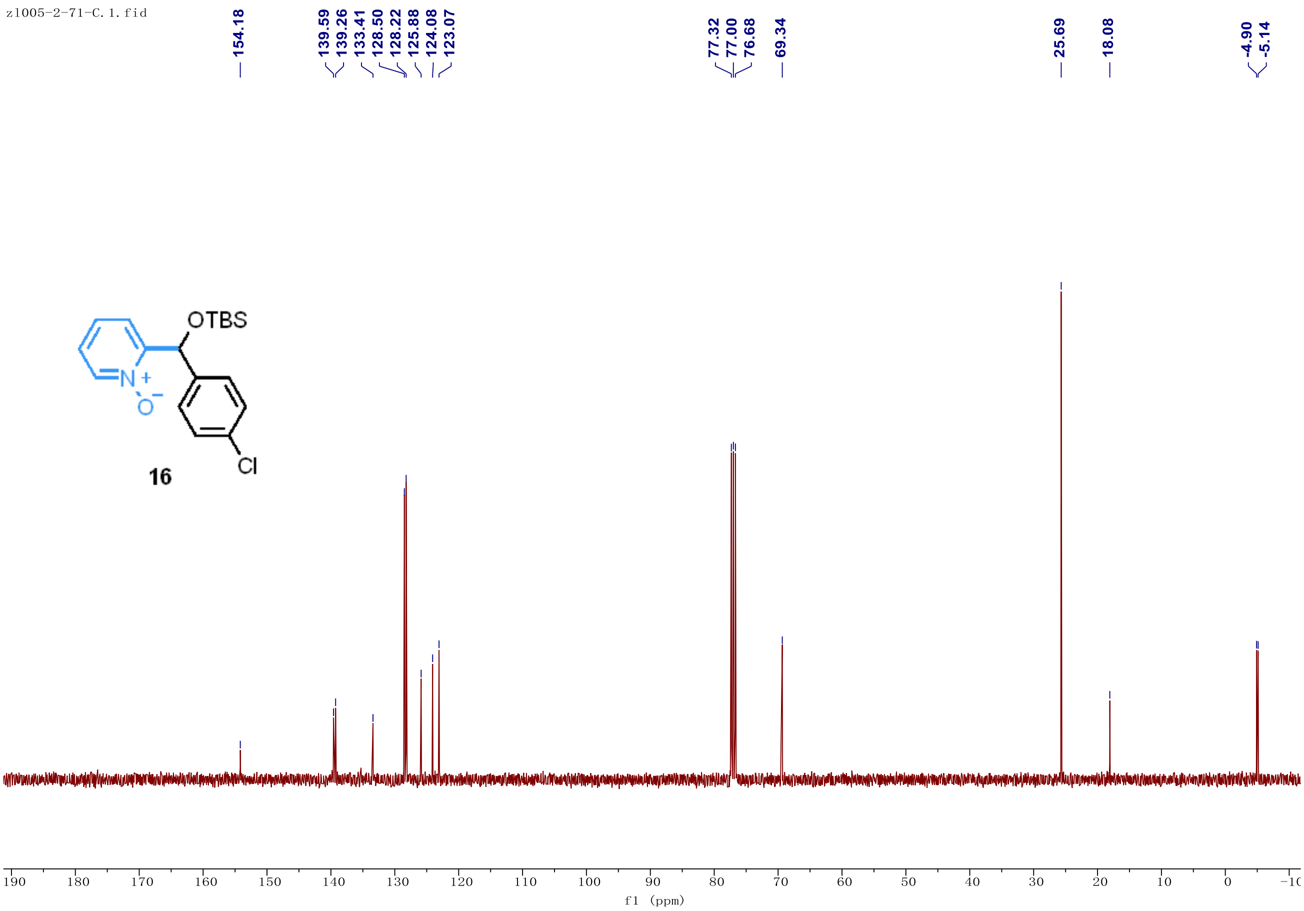
z1005-2-71-C.1.fid
154.18
139.59
139.26
133.41
128.50
128.22
125.88
124.08
123.07
77.32
77.00
76.68
69.34
25.69
18.08
-4.90
-5.14
OTBS
N+
O-
Cl
16
190 180 170 160 150 140 130 120 110 100 90 80 70 60 50 40 30 20 10 0
f1 (ppm)

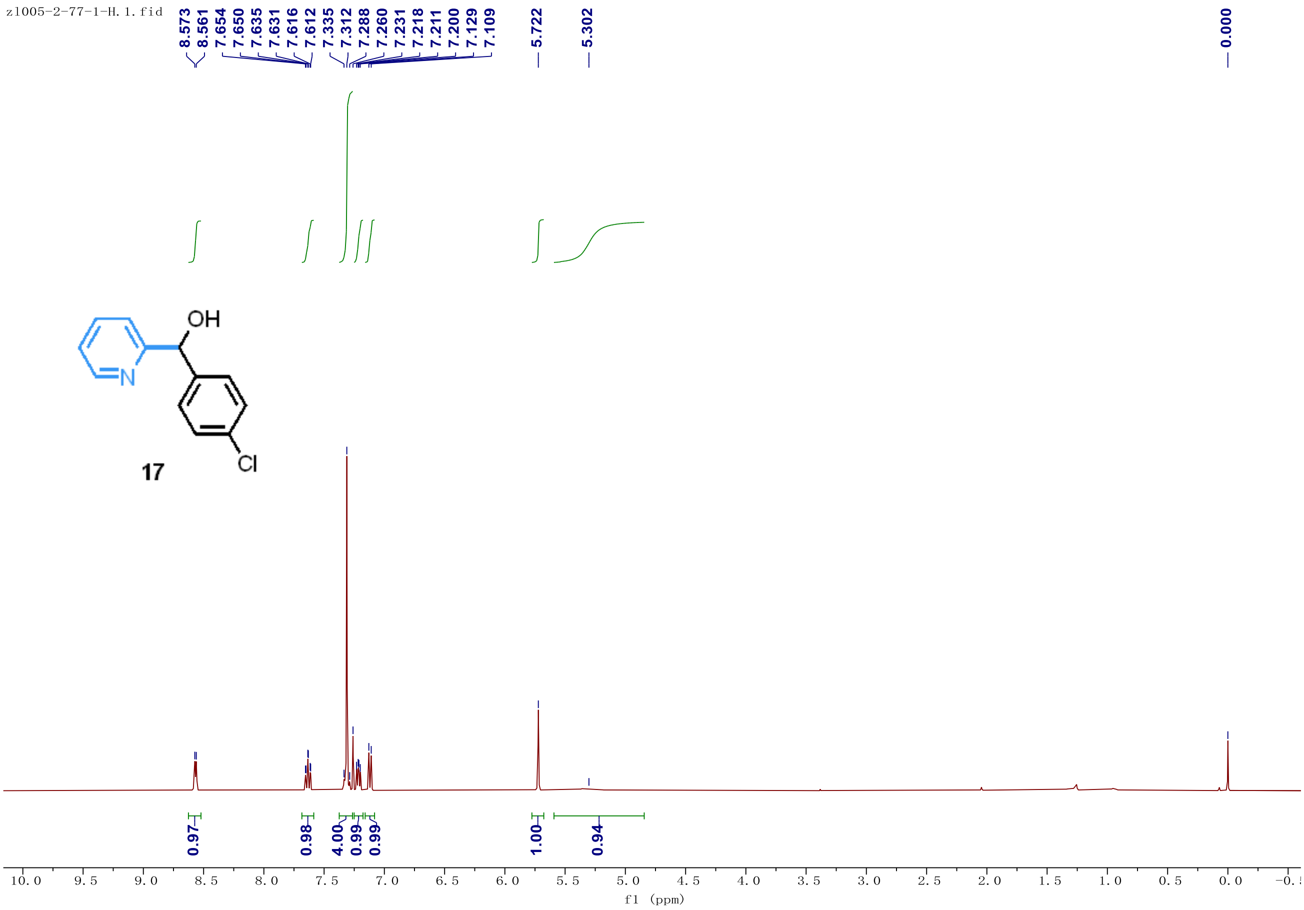
z1005-2-77-1-H.1.fid
8.573
8.561
7.654
7.650
7.635
7.631
7.616
7.612
7.335
7.312
7.288
7.260
7.231
7.218
7.211
7.200
7.129
7.109
5.722
5.302
0.000
OH
N
Cl
17
0.97
0.98
4.00
0.99
0.99
1.00
0.94
10.0 9.5 9.0 8.5 8.0 7.5 7.0 6.5 6.0 5.5 5.0 4.5 4.0 3.5 3.0 2.5 2.0 1.5 1.0 0.5 0.0
f1 (ppm)

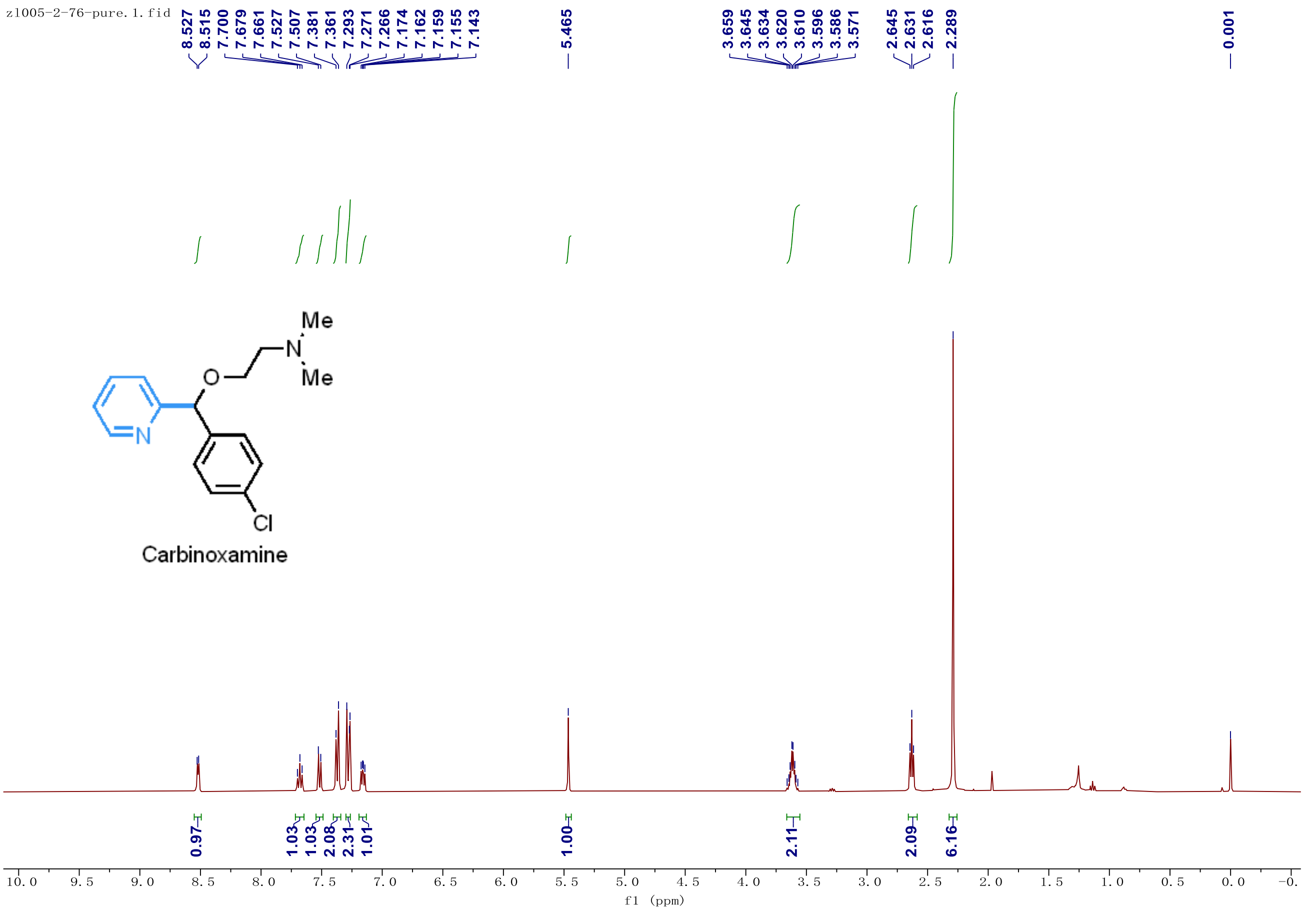
z1005-2-76-pure.1.fid
Carbinoxamine
f1 (ppm)

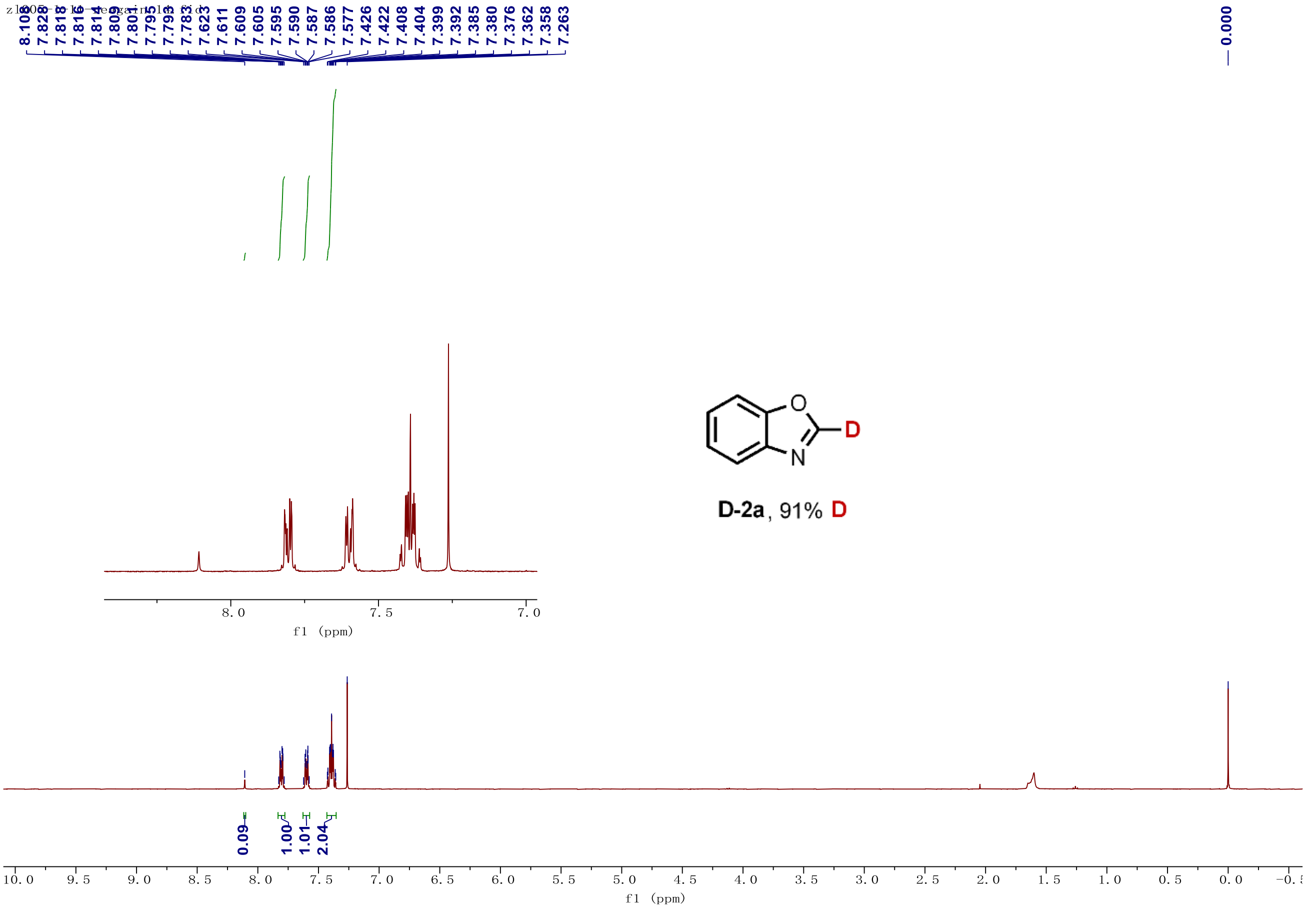
D-2a, 91% D
f1 (ppm)

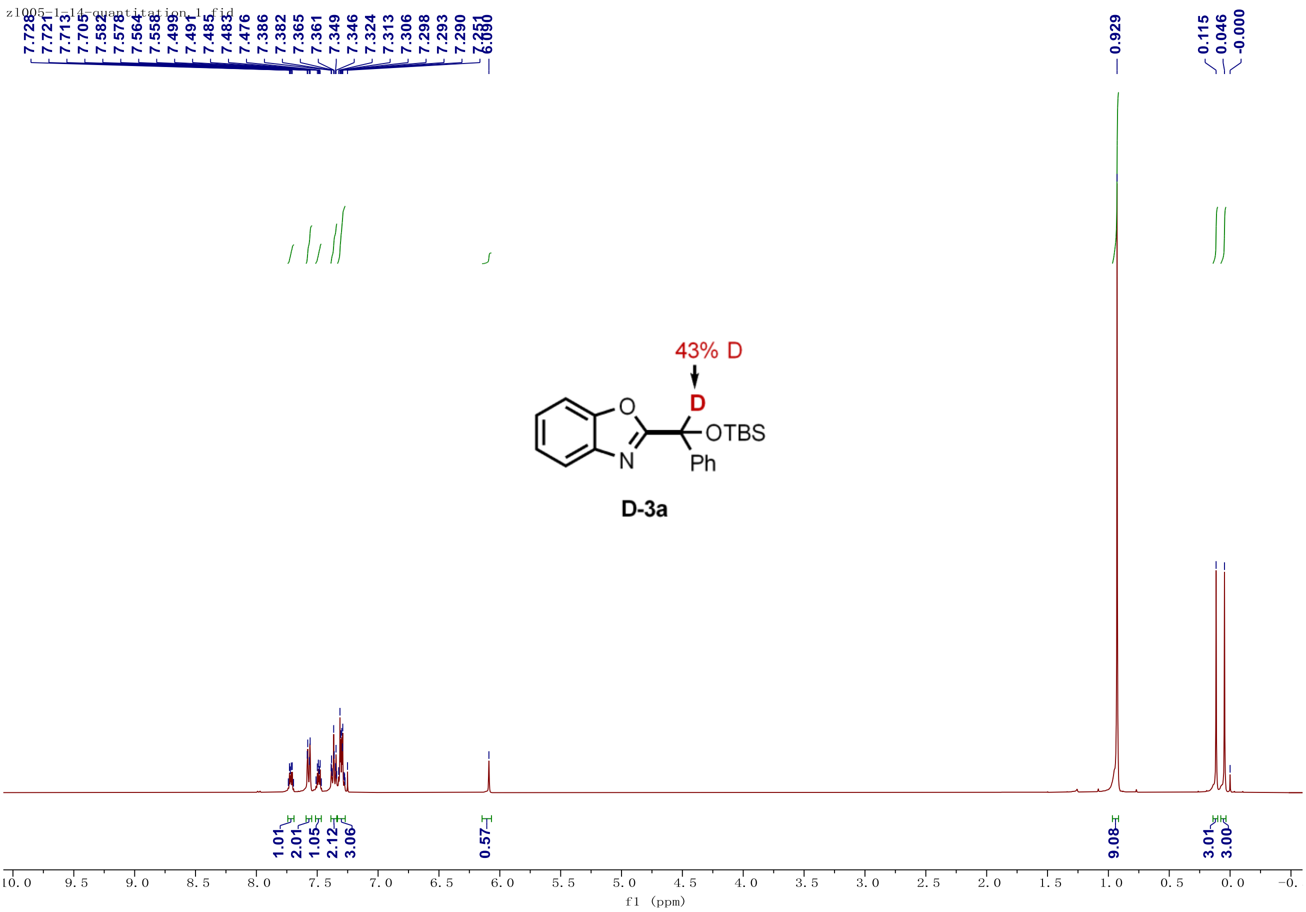

43% D
D
OTBS
Ph
D-3a
0.929
0.115
0.046
-0.000
1.01
2.01
1.05
2.12
3.06
0.57
9.08
3.01
3.00
f1 (ppm)

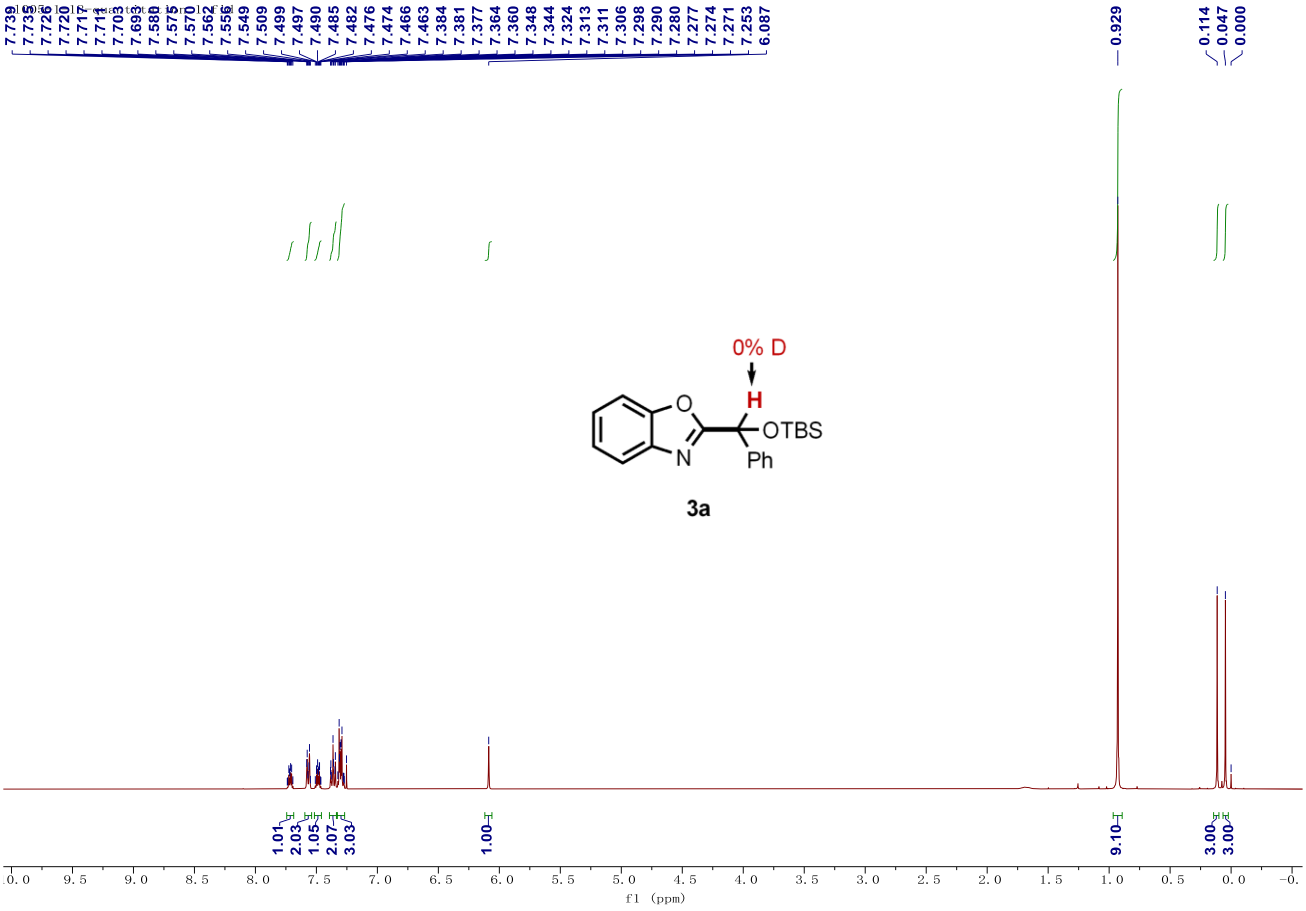

0% D
H
OTBS
Ph
3a
0.929
0.114
0.047
0.000
1.01
2.03
1.05
2.07
3.03
1.00
9.10
3.00
3.00
f1 (ppm)

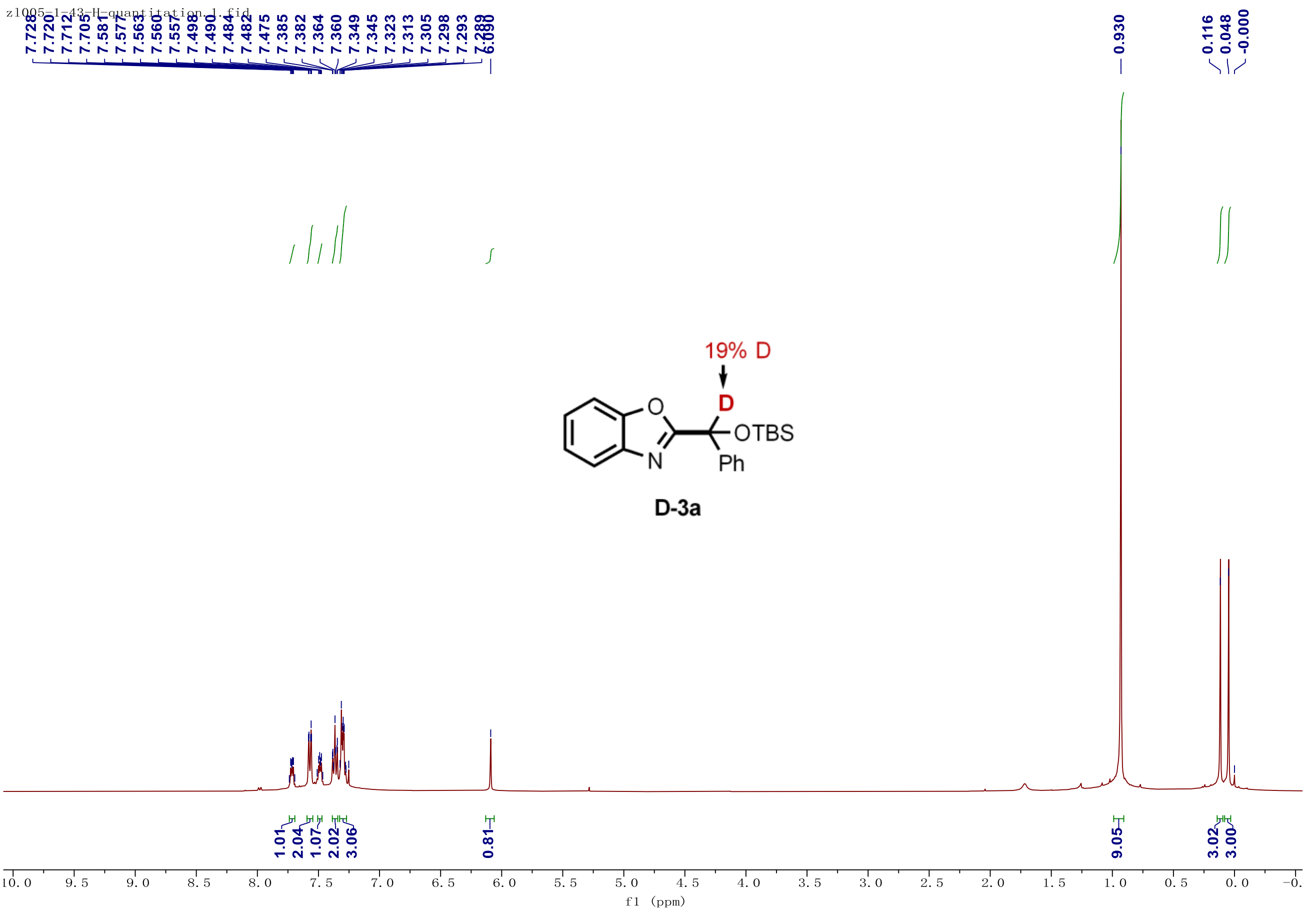
19% D
D
O
N
OTBS
Ph
D-3a
f1 (ppm)

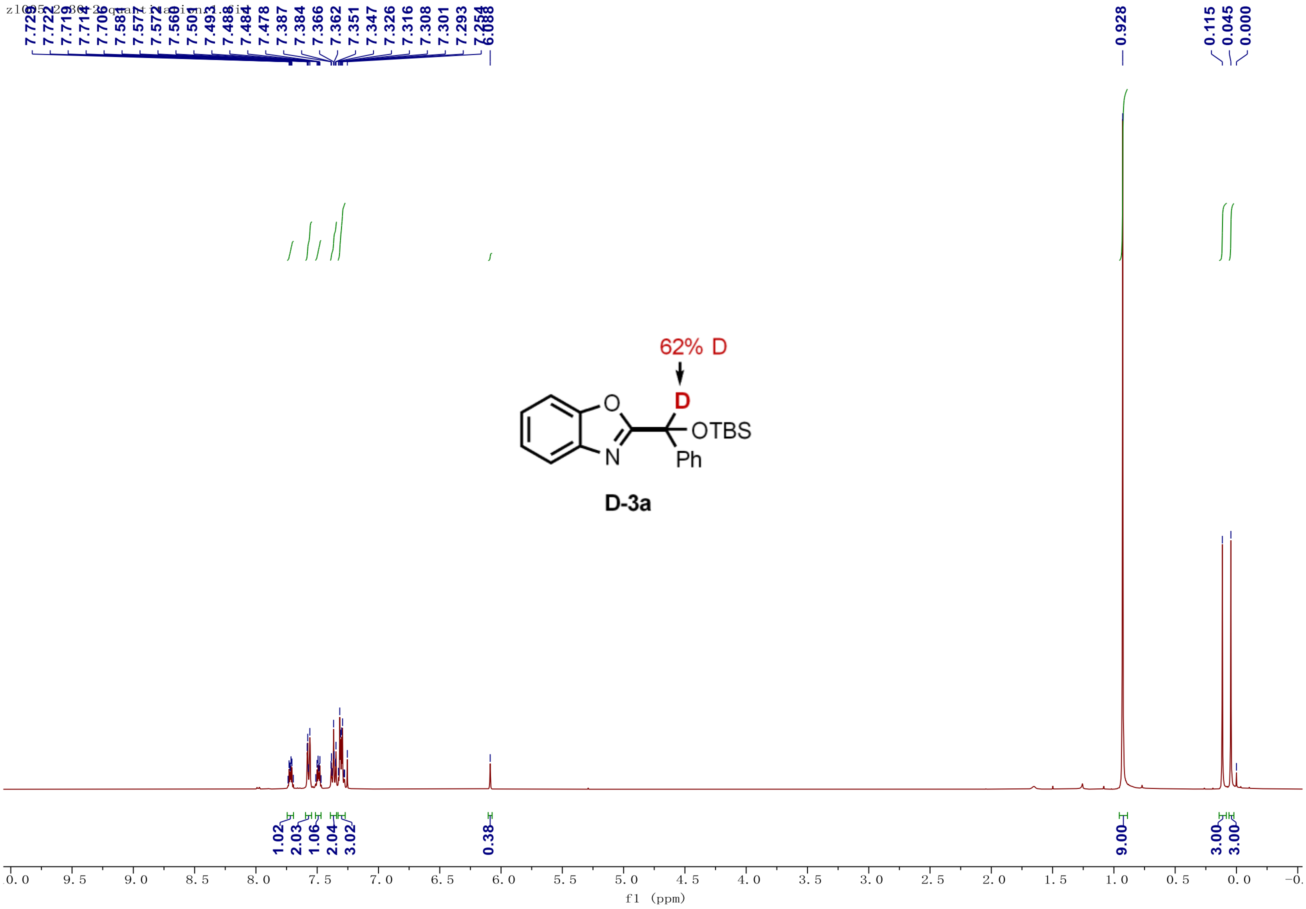
62% D
D
O
N
OTBS
Ph
D-3a
f1 (ppm)

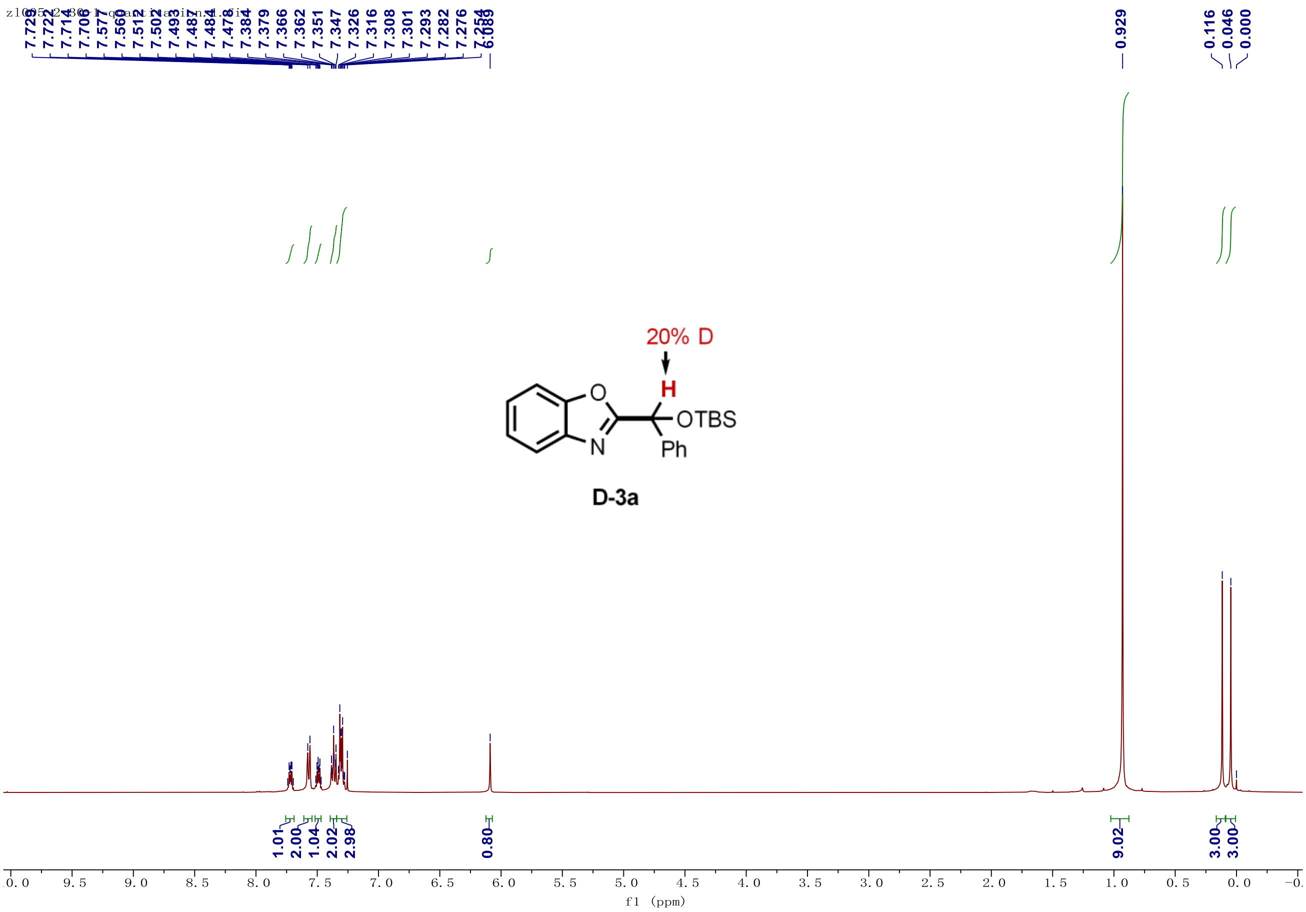
7.729
7.722
7.714
7.706
7.577
7.560
7.512
7.502
7.493
7.487
7.484
7.478
7.384
7.379
7.366
7.362
7.351
7.347
7.326
7.316
7.308
7.301
7.293
7.282
7.276
7.254
6.089
0.929
0.116
0.046
0.000
20% D
H
O
N
OTBS
Ph
D-3a
1.01
2.00
1.04
2.02
2.98
0.80
9.02
3.00
3.00
0.0 9.5 9.0 8.5 8.0 7.5 7.0 6.5 6.0 5.5 5.0 4.5 4.0 3.5 3.0 2.5 2.0 1.5 1.0 0.5 0.0 -0.
f1 (ppm)

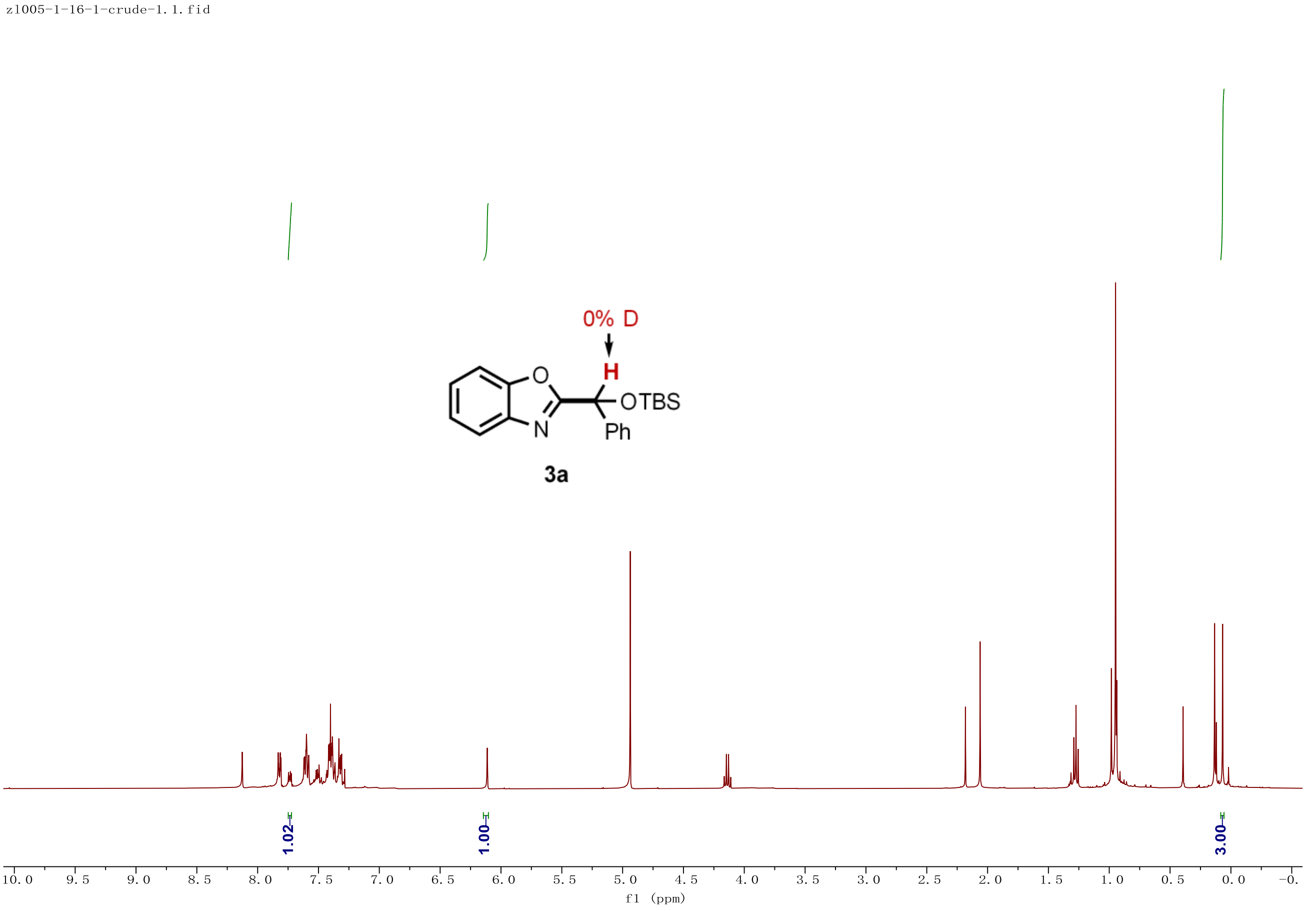
z1005-1-16-1-crude-1.1.fid
0% D
H
O
N
OTBS
Ph
3a
1.02
1.00
3.00
10.0 9.5 9.0 8.5 8.0 7.5 7.0 6.5 6.0 5.5 5.0 4.5 4.0 3.5 3.0 2.5 2.0 1.5 1.0 0.5 0.0 -0.
f1 (ppm)

z1005-1-16-2-crude.1.fid

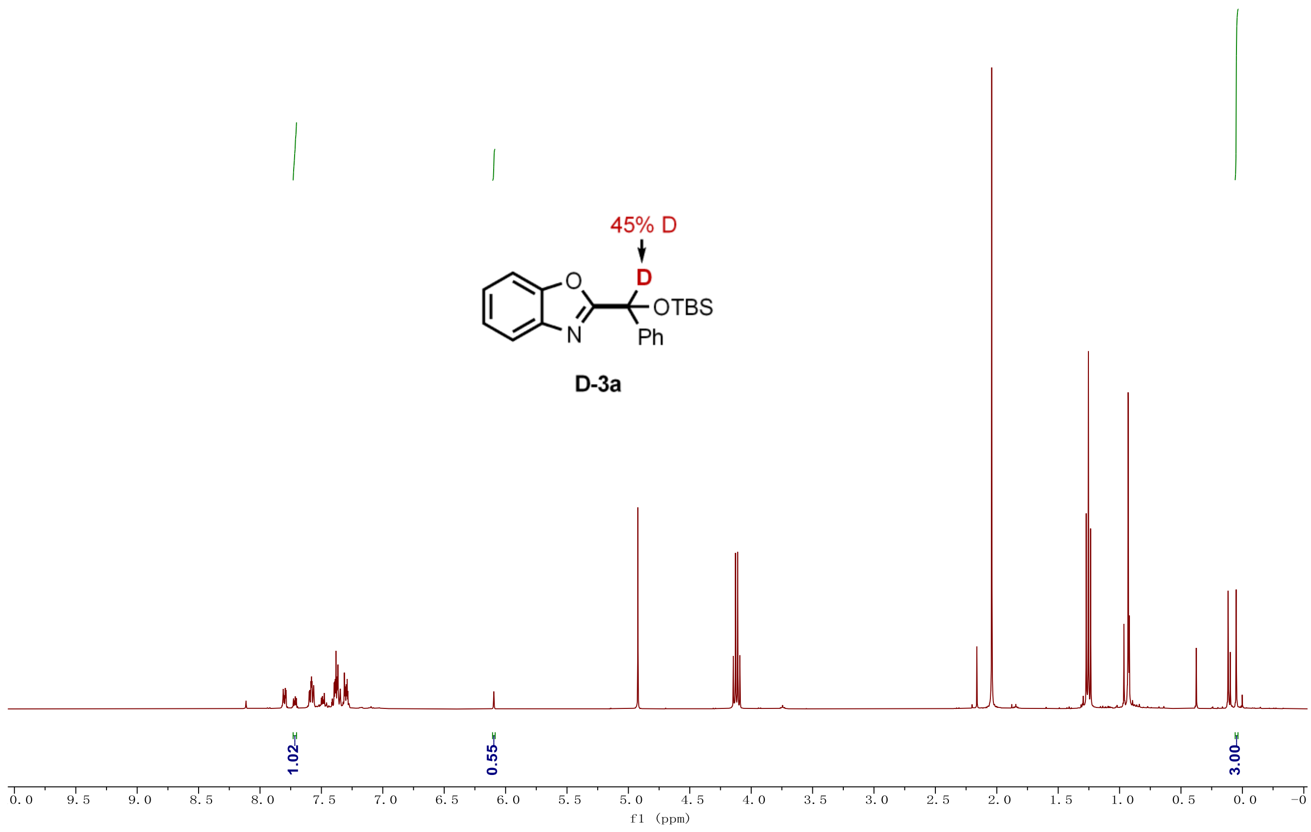

Supplement: Supplementary file 1 — Supporting Information [file ADVS-11-2409457-s001.pdf]
